# Supplementary material for: Cooperative chemoenzymatic and biocatalytic cascades to access chiral sulfur compounds bearing C(sp3)–S stereocentres
Source: Nat Commun. 2024 Sep 27;15:8332. doi: 10.1038/s41467-024-52608-8 (PMC11436715; doi:10.1038/s41467-024-52608-8)
Supplement: Supplementary file 1 — Supplementary Information [file 41467_2024_52608_MOESM1_ESM.pdf]

Supplementary Information

Cooperative chemoenzymatic and biocatalytic cascades to access chiral sulfur compounds bearing C(sp^3)–S stereocentres

Fei Zhao[1], Ariane Mattana[1], Ruqaiya Alam[1], Sarah Montgomery[2], Akash Pandya[2], Fabrizio Manetti[3], Beatriz Dominguez[2]* & Daniele Castagnolo[1]*

[1] Department of Chemistry, University College London, 20 Gordon Street, London WC1H 0AJ, UK.

[2] Johnson Matthey, 28 Cambridge Science Park, Milton Road, Cambridge CB4 0FP, UK.

[3] Department of Biotechnology, Chemistry and Pharmacy, University of Siena, via Aldo Moro 2, Siena 53100, Italy.

*Corresponding authors. Emails: d.castagnolo@ucl.ac.uk, beatriz.dominguez@matthey.com

Table of Contents

I. General Information……………………………………………………………………4

II. Biology…………………………………………………………………………………...4

III. Chemistry……………………………………………………………………………..7

General Procedure for the Synthesis of α-Thio/Amino/Oxy/Selanyl Fumarate and Maleate **1aa-1aw** and **1ba-1bd**……………………………………………………………………..7

Procedure for the Synthesis of *E*-**1ax**……………………………………………..…………..16

General Procedure for the Synthesis of α-Sulfonyl Fumarates **1ay** and **1az**……..…………..16

General Procedure for the Synthesis of Racemic α-Thio Succinates **2aa-2as** and **2au-2aw**…17

Procedure for the Synthesis of Racemic α-Thio Succinate **2at**…………………..………..22

Procedure for the Synthesis of Racemic **2ax**……………………………………..………..23

General Procedure for the Synthesis of Racemic α-Sulfonyl Succinates **2ay** and **2az**………..23

General Procedure for the Synthesis of Racemic α-Amino/Oxy/Selanyl Succinates **2ba-2bd**.24

General Procedure for the Synthesis of **9aa-9aj**………………………………..…………..25

General Procedure for the Synthesis of **5aa-5aj**……………………………...……………..28

Procedure for the Synthesis of **5ak**……………………………..………...……………..31

Procedure for the Synthesis of **5al**……………………………..………...……………..32

Procedure for the Synthesis of *Z*-**5am**……………………………..………...……………..32

General Procedure for the Synthesis of Racemic **7aa-7af**………..…………..………..………..33

General Procedure for the Synthesis of Racemic **7ag-7ah**……..…………..………..………..34

Procedure for the Synthesis of Racemic **7ai**……………………...…………..……….…..35

Procedure for the Synthesis of Racemic **7aj**……………………...…………..……….…..35

Procedure for the Synthesis of Racemic **7ak**……………………...…………..……….…..36

Procedure for the Synthesis of Racemic **7al**……………………...…………..……….…..37

Procedure for the Synthesis of Racemic **7am**……………………....…………..……….…..37

General Procedure for the Synthesis of Racemic **10aa-10ae**……....…………..……….…..37

Procedure for the Synthesis of **6aa**……………………...…………………………..……….…..39

Procedure for the Synthesis of **6ab**……………………...……………………………….…..40

Procedure for the Synthesis of Racemic **8aa**…………...………..…………………….…..40

Procedure for the Synthesis of Racemic **8ab**…………...………..…………………….…..41

Procedure for the Synthesis of Racemic **11aa** and (*S*)-**11aa**…….…………………….…..41

Reaction Condition Optimization for ENE Biocatalysed Enantioselective Reduction of **1aa**..42

General Procedure for ENE-101 Biocatalysed Enantioselective Reduction of (*Z*)-**1** and/or (*E*)-**1** into (*S*)-**2**…………………………………………………………..………………..43

Reaction Condition Optimization for Two-step One-pot Chemoenzymatic Cascade for the Enantioselective Synthesis of (*S*)-**2aa**……………………………………………………..45

General Procedure for Two-step One-pot Chemoenzymatic Cascade for the Enantioselective Synthesis of (*S*)-**2**………..…………………………………………...………………....45

Reaction Condition Optimization for One-Step One-pot Chemoenzymatic Cascade for the Enantioselective Synthesis of (*S*)-**2aa**……………………………………………………..47

General Procedure for One-Step One-pot Chemoenzymatic Cascade for the Enantioselective Synthesis of (*S*)-**2**……..……………….………………………...………………......47

Reaction Condition Optimization for ENE Biocatalysed Enantioselective Reduction of **5aa**..49

Investigation of the Configurational Stability of (*S*)-**7aa**…………………………….…..50

General Procedure for ENE-101 Biocatalysed Enantioselective Reduction of **5** or **6** into (*S*)-**7** or **8**……………………..………………………………………………..………………….....50
The time course experiment on ENE-101 biocatalysed reduction of **5aj**……………………..…52
Screening of ADHs for the Oxidation of **9aa** into **5aa**…………………..…………………….....52
General Procedure for One-Step One-Pot Hydrogen-Borrowing Cascade for the Enantioselective Synthesis of (*S*)-**7** from **9**……………..…………..……..………………….....53
Screening of ADHs for the Reduction of **7ad** into **9ad**.…………..……...……………….......54
General Procedure for Two-Step One-Pot Biocatalytic-Chemical (Bio-Chem) or Biocatalytic-Biocatalytic (Bio-Bio) Cascade for the Enantioselective Synthesis of **10** from **5**……………..54
General Procedure for One-Step One-Pot Biocatalytic-Biocatalytic (Bio-Bio) Cascade for the Enantioselective Synthesis of **10** from **5**…….……………..………..……...…………………....55
General Procedure for Three-Step One-Pot Biocatalytic-Biocatalytic-Biocatalytic (Bio-Bio-Bio) Cascade for the Enantioselective Synthesis of **10** from **9**……..……...………………......56
Deuterium-Labelling Experiments…………………..…………………...…………….…......58
Optical Rotations of Chiral Sulfide Products…..……………………...……………….….......61
IV. Computational Study……………………………………………………………...……....61
V. Copies of ^{1}H NMR, ^{13}C NMR, ^{19}F NMR and NOESY Spectra……………………………..63
VI. Conditions for HPLC Analysis and Copies of HPLC Spectra……………………..…......227
VII. Supplementary References………………………………………………………………...328

I. General Information

If not otherwise specified, the reagents were obtained from commercial sources and used directly without purification. Analytical thin-layer chromatography (TLC) was performed using commercially available pre-coated plates and visualized with UV light at 254 nm. Column chromatography was carried out using Sigma Aldrich silica gel particle size, 40-63 µm particle size 60 Å. ^{1}H NMR, ^{13}C NMR, ^{19}F NMR and NOESY were measured with Bruker (Germany) Ascend400 Spectrometer, Bruker (Germany) Avance III 400 or Bruker (Germany) Avance Neo 500 at room temperature operating at the frequencies indicated. Chemical shifts (δ) are given in ppm, referenced to tetramethylsilane. Coupling constants (*J*) are reported in Hertz (Hz), proton coupling patterns were recorded as singlet (s), doublet (d), triplet (t), quartet (q), and multiplet (m). Mass spectra were recorded at the EPSRC National Mass Spectrometry Service Centre on a Thermo Scientific LTQ Orbitrap XL Mass Spectrometer using low-resolution ESI or high-resolution nano ESI techniques. HPLC analysis was carried out using a Perkin-Elmer 1100 HPLC system coupled with UV/Vis set to the appropriate wavelength (214 nm, 230 nm or 254 nm). The chiral columns used for HPLC analysis included Chiralpak® IG (4.6 mm × 250 mm, 5 µm), Chiralpak® ID column (4.6 mm × 250 mm, 5 µm), Chiralcel® OJ-H column (4.6 mm × 250 mm, 5 µm), Chiralcel® OD-H column (4.6 mm × 250 mm, 5 µm) and Chiralpak® IC column (4.6 mm × 250 mm, 5 µm) supplied by Daicel. Hexane, heptane, isopropanol (IPA) and ethanol (EtOH) were used as the eluent system for all columns. The conversion was determined by integration of the product peak in HPLC (uncorrected area under curve), the yield was determined after isolation by flash chromatography, and the enantiomeric excess was determined through chiral HPLC analysis. α_D measurements were taken using a Bellingham and Stanley ADP440+ Polarimeter with a cell length of 0.5 dm.

II. Biology

All the enzymes including ENEs, GDHs, ADHs used in this study are cell-free extracts (CFE), which are commercially available via https://matthey.com from Johnson Matthey for the replication of this work.[1] The product code for the ENE kit is EZK002, which includes 7 ENEs, 3 GDHs, 1 FDH, NAD^+ and $NADP^+$. The product code for the ADH kit is EZK003, which includes 17 ADHs, 3 GDHs, 1 FDH, NAD^+ and $NADP^+$. All the enzymes are also available from Johnson Matthey for cooperative research purposes, and a material transfer agreement (MTA) needs to be signed in this case. Please contact Dr Beatriz Dominguez (beatriz.dominguez@matthey.com) for information on ENE and ADH enzymes.

The enzyme NOX-009 was provided in kind by Prozomix Ltd.

The ENE biocatalysts NCR, pQR1440 and pQR1907, used as cell free extract (CFE) for comparison experiments were kindly provided by Prof. Helen Hailes at UCL.

ENE-101 was expressed in *E. coli* using standard expression techniques. The nucleic acid sequence optimized for expression of ENE-101 in *E. coli* was cloned into a standard expression vector for IPTG-inducible expression. ENE-101 preparations were produced by harvesting cells, recovering biomass and resuspending the biomass in buffer (0.1 M potassium phosphate, pH 7.0), lysing the suspension and removing the cell debris to provide a clarified cell lysate. The clarified cell lysate was filtered first through filters of 0.5 µm and 0.2 µm pore size, and then further concentrated by ultrafiltration to provide an ENE-101 preparation (circa 20% protein by weight). Such ENE-101 preparations may be used directly or lyophilised for subsequent reconstitution and use. The powder activity (U/mg) of ENE-101 was determined by the assay described in Supplementary Table 1, using 1-octene-3-one as the reference substrate.

The protein sequence of ENE-101[1]
MSHTLFDPVQAGDLQLANRIAMAPLTRNRSPNAVPKDITATYYAQRATAGLLITEATAIS
HQGQGYADVPGLYSTEQLDGWKKVTAAVHERGGRIVTQLWHVGRISHNDLQPDGGAPV
APSAIAAKSKTYLIDKATGQGHFAATSEPRALDAEELPGIVHDYAAAARNAVETAGFDGV
EIHGANGYLLDQFLKTGANRRTDDYGGSIENRARLLLEATRAVVDAIGGGKVGIRLSPVT
PANDIVDADPQPLFDYVIRQLAPLGLAYVHVIEGSTGGPRELEDRPFDYEALKTAYREAG
GKGAWMVNNAYDRALAMEAVASGRADIVAFGKAFISNPDLVERLRQDAPLNPWDSKTF
YGGGEKGYTDYPTLGESAKG**

The gene sequence of ENE-101[1]
ATGAGCCATACCCTGTTTGATCCGGTTCAGGCAGGCGATCTGCAGCTGGCAAATCGTA
TTGCAATGGCACCGCTGACCCGTAATCGTAGCCCGAATGCAGTTCCGAAAGATATTAC
CGCAACCTATTATGCACAGCGTGCAACCGCAGGTCTGCTGATTACCGAAGCAACCGC
AATTAGCCATCAGGGTCAGGGTTATGCAGATGTTCCGGGTCTGTATAGCACCGAACA
GCTGGATGGTTGGAAAAAAGTTACCGCAGCAGTGCATGAACGTGGTGGTCGTATTGT
TACCCAGCTGTGGCATGTGGGTCGTATTAGCCATAATGATCTGCAACCGGATGGTGGT
GCTCCGGTGGCACCGAGCGCAATTGCAGCAAAAAGCAAAACCTATCTGATTGATAAA
GCAACCGGTCAGGGTCATTTTGCAGCAACCAGCGAACCGCGTGCACTGGATGCAGAA
GAACTGCCTGGTATTGTTCATGATTATGCAGCAGCAGCACGTAATGCAGTTGAAACCG
CAGGCTTTGATGGTGTTGAAATTCATGGTGCAAATGGCTATCTGCTGGATCAGTTTCT
GAAAACCGGTGCAAATCGTCGTACCGATGATTATGGTGGTAGCATTGAAAATCGTGC
CCGTCTGCTGCTGGAAGCAACCCGTGCAGTTGTTGATGCAATTGGTGGTGGTAAAGTT
GGTATTCGTCTGAGTCCGGTTACACCGGCAAATGATATTGTGGATGCCGATCCGCAGC
CGCTGTTTGATTATGTTATTCGTCAGCTGGCTCCGCTGGGTCTGGCCTATGTTCATGTT
ATTGAAGGTAGCACCGGTGGTCCTCGTGAACTGGAAGATCGTCCGTTCGATTATGAA
GCACTGAAAACAGCATATCGTGAAGCAGGCGGTAAAGGTGCATGGATGGTTAATAAT
GCCTATGATCGTGCCCTGGCAATGGAAGCAGTTGCAAGCGGTCGTGCAGATATTGTTG
CATTTGGTAAAGCCTTTATTAGCAATCCGGATCTGGTTGAACGTCTGCGTCAGGATGC
TCCGCTGAATCCGTGGGATAGTAAAACCTTTTATGGTGGCGGTGAAAAAGGCTATAC
CGATTATCCGACCCTGGGTGAAAGCGCAAAAGGTTAATAA

ADH-19, ADH-153 and ADH-159 are short chain dehydrogenases which are the intellectual property (IP) of Johnson Matthey, which means their gene sequences are not publicly available here.

Supplementary Table 1. Enzyme activity assays of ENEs and GDHs.

Enzyme	Enzyme Activity Assay	Powder Appearance	Powder Activity (U/mg)
ENE-101<br>ENE-102<br>ENE-103<br>ENE-107<br>ENE-108<br>ENE-109	One unit will reduce 1.0 µmol of 1-octen-3-one at pH 7 and 40 °C in the presence of NADH (25 mM). The double bond reduction of 1-octen-3-one can be measured by analysing the formation of 3-octanone using GC.	yellow<br>yellow<br>yellow<br>yellow<br>yellow<br>yellow	65.8<br>5.4<br>3.0<br>101.0<br>6.0<br>6.1
ENE-105	One unit will reduce 1.0 µmol of 1-octen-3-one at pH 7 and 40 °C in the presence of NADPH (25 mM). The double bond reduction of 1-octen-3-one can be measured by analysing the formation of 3-octanone using GC.	yellow	9.0
GDH-101	One unit will reduce 1.0 µmol of NAD to NADH per minute at pH 7 and 25 °C while oxidising glucose (100 mM) to glucono-1,5-lactone. NADH formation can be measured spectrophotometrically via an increase of absorbance at 340 nm. The extinction coefficient is 6220 $M^{-1}cm^{-1}$.	off white	32.0
GDH-5	One unit will reduce 1.0 µmol of NADP to NADPH per minute at pH 7 and 25 °C while oxidising glucose (100 mM) to glucono-1,5-lactone. NADPH formation can be measured spectrophotometrically via an increase of absorbance at 340 nm. The extinction coefficient is 6220 $M^{-1}cm^{-1}$.	off white	51.2
GDH-8	One unit will reduce 1.0 µmol of NADP to NADPH per minute at pH 10 and 25 °C while oxidising glucose (100 mM) to glucono-1,5-lactone. NADPH formation can be measured spectrophotometrically via an increase of absorbance at 340 nm. The extinction coefficient is 6220 $M^{-1}cm^{-1}$.	off white	1.6

Supplementary Table 2. Enzyme activity assays of ADHs.

Enzyme	Enzyme Activity Assay	Powder Appearance	Powder Activity (U/mg)
ADH-101	One unit will convert 1.0 μmol of 4'-chloroacetophenone per minute at pH 7 at 25 °C in the presence of NADPH (0.1 mM). NADPH depletion can be measured spectrophotometrically at 340 nm. The extinction coefficient is 6220 $M^{-1}cm^{-1}$.	off white	27.4
ADH-110		off white	8.5
ADH-104	One unit will reduce 1.0 μmol of ethyl-2-oxo-4-phenylbutyrate per minute at pH 7 at 25 °C in the presence of NADH (0.1 mM). NADH depletion can be measured spectrophotometrically at 340 nm. The extinction coefficient is 6220 $M^{-1}cm^{-1}$.	off white	62.0
ADH-105	One unit will convert 1.0 μmol of 4'-chloroacetophenone per minute at pH 7 at 25 °C in the presence of NADH (0.1 mM). NADH depletion can be measured spectrophotometrically at 340 nm. The extinction coefficient is 6220 $M^{-1}cm^{-1}$.	off white	4.9
ADH-19	One unit will reduce 1.0 μmol of 2,3-pentanedione at pH 7 at 25 °C in the presence of NADPH (0.1 mM). NADPH depletion was measured spectrophotometrically at 340 nm, extinction coefficient is 6220 $M^{-1}cm^{-1}$.	off white	2.8
ADH-20		off white	1.0
ADH-27		off white	1.6
ADH-61		off white	1.9
ADH-62		off white	3.2
ADH-150		off white	4.1
ADH-153		off white	3.3
ADH-159		off white	0.2
ADH-160		off white	0.5
ADH-171		off white	10.9
ADH-220		off white	1.2
ADH-230		off white	0.2
ADH-244		off white	2.1

III. Chemistry

General Procedure for the Synthesis of α-Thio/Amino/Oxy/Selanyl Fumarate and Maleate 1aa-1aw and 1ba-1bd

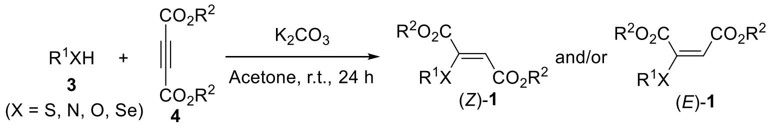

General Procedure A: To a solution of thiophenols/thiols/anilines/phenols/phenylselenols **3** (3 mmol) and dimethyl/diethyl/diisopropyl/di-*tert*-butyl acetylenedicarboxylate **4** (6 mmol) in acetone (30 mL) was added K_2CO_3 (6 mmol), and the resulting mixture was stirred at room temperature for 24 h. After filtration, an aliquot of the filtrate was taken for ^1H NMR analysis to determine the ratio of *Z* and *E* isomers of the products. Then the filtrate was concentrated, and the residue obtained was purified by flash chromatography (Hexane/EtOAc: 16/1→Hexane/EtOAc: 8/1) on silica gel to give

the *Z* and/or *E* products **1**.

According to ***General Procedure A***, the reaction gave two stereoisomers (*Z*-**1aa**/*E*-**1aa** = 4.1/1, 741.1 mg, 98% combined yield), which were separable by chromatography.

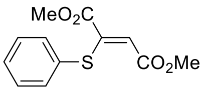

dimethyl 2-(phenylthio)fumarate (*Z*-1aa): Yellow oil. ^{1}H NMR (400 MHz, $CDCl_3$) δ 7.49-7.41 (m, 2H), 7.35-7.30 (m, 3H), 6.37 (s, 1H), 3.80 (s, 3H), 3.33 (s, 3H); ^{13}C NMR (101 MHz, $CDCl_3$) δ 165.67, 164.92, 150.07, 133.49, 132.16, 129.18, 129.06, 118.83, 52.73, 52.08; LRMS (ESI) m/z: 253 $[M+H]^+$. The characterization data of this compound were in accordance with the published ones.[2]

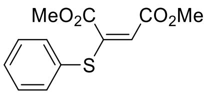

dimethyl 2-(phenylthio)maleate (*E*-1aa): Pale yellow oil. ^{1}H NMR (400 MHz, $CDCl_3$) δ 7.60-7.52 (m, 2H), 7.50-7.38 (m, 3H), 5.50 (s, 1H), 3.73 (s, 3H), 3.67 (s, 3H); ^{13}C NMR (101 MHz, $CDCl_3$) δ 165.53, 164.28, 151.44, 135.81, 130.65, 129.98, 127.30, 113.88, 53.12, 52.00; LRMS (ESI) m/z: 253 $[M+H]^+$.

According to ***General Procedure A***, the reaction gave two stereoisomers (*Z*-**1ab**/*E*-**1ab** = 2.5/1, 763.6 mg, 94% combined yield), which were separable by chromatography.

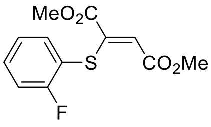

dimethyl 2-((2-fluorophenyl)thio)fumarate (*Z*-1ab): Yellow oil. ^{1}H NMR (400 MHz, $CDCl_3$) δ 7.50-7.42 (m, 1H), 7.39-7.31 (m, 1H), 7.16-7.05 (m, 2H), 6.46 (s, 1H), 3.80 (s, 3H), 3.41 (s, 3H); ^{13}C NMR (101 MHz, $CDCl_3$) δ 165.64, 164.44, 162.23 (d, J_{C-F} = 249.2 Hz), 148.53, 135.83, 131.66 (d, J_{C-F} = 7.9 Hz), 124.72 (d, J_{C-F} = 3.9 Hz), 119.76, 119.31 (d, J_{C-F} = 18.3 Hz), 116.16 (d, J_{C-F} = 22.6 Hz), 52.89, 52.16; ^{19}F NMR (376 MHz, $CDCl_3$) δ -106.71; LRMS (ESI) m/z: 271 $[M+H]^+$.

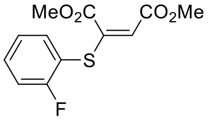

dimethyl 2-((2-fluorophenyl)thio)maleate (*E*-1ab): Pale yellow oil. ^{1}H NMR (400 MHz, $CDCl_3$) δ 7.58-7.52 (m, 1H), 7.52-7.45 (m, 1H), 7.24-7.15 (m, 2H), 5.57 (s, 1H), 3.71 (s, 3H), 3.67 (s, 3H); ^{13}C NMR (101 MHz, $CDCl_3$) δ 165.06, 164.07, 162.94 (d, J_{C-F} = 251.9 Hz), 148.87, 137.74, 133.44 (d, J_{C-F} = 8.1 Hz), 125.36 (d, J_{C-F} = 4.1 Hz), 116.79 (d, J_{C-F} = 22.4 Hz), 114.87, 114.58 (d, J_{C-F} = 18.5 Hz), 53.11, 52.05; ^{19}F NMR (376 MHz, $CDCl_3$) δ -104.88; LRMS (ESI) m/z: 271 $[M+H]^+$.

According to ***General Procedure A***, the reaction gave two stereoisomers (*Z*-**1ac**/*E*-**1ac** = 3.5/1, 803.4 mg, 93% combined yield), which were separable by chromatography.

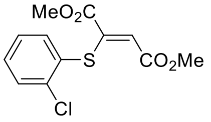

dimethyl 2-((2-chlorophenyl)thio)fumarate (*Z*-1ac): Yellow oil. ^{1}H NMR (400 MHz, $CDCl_3$) δ 7.49-7.40 (m, 2H), 7.31-7.19 (m, 2H), 6.56 (s, 1H), 3.81 (s, 3H), 3.42 (s, 3H); ^{13}C NMR (101 MHz, $CDCl_3$) δ 165.55, 164.50, 147.86, 137.66, 135.20, 131.79, 130.37, 130.23, 127.32, 121.22, 52.95, 52.21; LRMS (ESI) m/z: 289 ($[M+H]^+$, (Cl^{37})), 287 ($[M+H]^+$, (Cl^{35})).

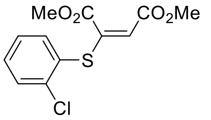

dimethyl 2-((2-chlorophenyl)thio)maleate (*E*-1ac): Pale yellow oil. ^{1}H NMR (400 MHz, $CDCl_3$) δ 7.64 (dd, J = 7.7, 1.7 Hz, 1H), 7.54 (d, J = 8.0 Hz, 1H), 7.45-7.38 (m, 1H), 7.35-7.29 (m, 1H), 5.49 (s, 1H), 3.74 (s, 3H), 3.67 (s, 3H); ^{13}C NMR (101 MHz, $CDCl_3$) δ 165.16, 164.12, 148.36, 139.83, 137.82, 132.21, 130.87, 128.03, 126.73, 115.20, 53.19, 52.07; LRMS (ESI) m/z: 289 ($[M+H]^+$, (Cl^{37})), 287 ($[M+H]^+$, (Cl^{35})).

According to ***General Procedure A***, the reaction gave two stereoisomers (*Z*-**1ad**/*E*-**1ad** = 1.2/1, 709.1 mg, 82% combined yield), which were separable by chromatography.

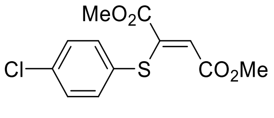

dimethyl 2-((4-chlorophenyl)thio)fumarate (*Z*-**1ad**): White solid. ^{1}H NMR (400 MHz, $CDCl_3$) δ 7.40-7.35 (m, 2H), 7.33-7.27 (m, 2H), 6.42 (s, 1H), 3.80 (s, 3H), 3.41 (s, 3H); ^{13}C NMR (101 MHz, $CDCl_3$) δ 165.55, 164.62, 148.90, 135.44, 134.64, 130.70, 129.39, 119.88, 52.93, 52.17; LRMS (ESI) m/z: 289 ($[M+H]^+$, (Cl^{37})), 287 ($[M+H]^+$, (Cl^{35})).

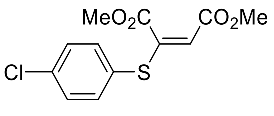

dimethyl 2-((4-chlorophenyl)thio)maleate (*E*-**1ad**): White solid. ^{1}H NMR (400 MHz, $CDCl_3$) δ 7.46 (d, *J* = 8.5 Hz, 2H), 7.38 (d, *J* = 8.5 Hz, 2H), 5.53 (s, 1H), 3.70 (s, 3H), 3.65 (s, 3H); ^{13}C NMR (101 MHz, $CDCl_3$) δ 165.09, 164.00, 150.37, 137.11, 136.85, 130.13, 125.73, 114.55, 53.08, 51.99; LRMS (ESI) m/z: 289 ($[M+H]^+$, (Cl^{37})), 287 ($[M+H]^+$, (Cl^{35})).

According to ***General Procedure A***, the reaction gave two stereoisomers (*Z*-**1ae**/*E*-**1ae** = 2.7/1, 897.0 mg, 90% combined yield), which were separable by chromatography.

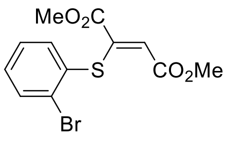

dimethyl 2-((2-bromophenyl)thio)fumarate (*Z*-**1ae**): Yellow oil. ^{1}H NMR (400 MHz, $CDCl_3$) δ 7.61 (d, *J* = 7.9 Hz, 1H), 7.45 (d, *J* = 7.7 Hz, 1H), 7.28-7.24 (m, 1H), 7.20-7.14 (m, 1H), 6.58 (s, 1H), 3.80 (s, 3H), 3.41 (s, 3H); ^{13}C NMR (101 MHz, $CDCl_3$) δ 165.51, 164.49, 147.72, 134.95, 134.12, 133.55, 130.31, 128.06, 127.96, 121.57, 52.93, 52.20; LRMS (ESI) m/z: 333 ($[M+H]^+$, (Br^{81})), 331 ($[M+H]^+$, (Br^{79})).

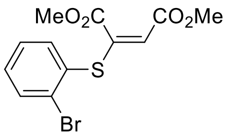

dimethyl 2-((2-bromophenyl)thio)maleate (*E*-**1ae**): Pale yellow oil. ^{1}H NMR (400 MHz, $CDCl_3$) δ 7.72 (d, *J* = 7.8 Hz, 1H), 7.66 (d, *J* = 7.6 Hz, 1H), 7.40-7.34 (m, 1H), 7.34-7.28 (m, 1H), 5.49 (s, 1H), 3.75 (s, 3H), 3.68 (s, 3H); ^{13}C NMR (101 MHz, $CDCl_3$) δ 165.10, 164.13, 148.32, 137.73, 134.25, 132.12, 130.53, 129.09, 128.69, 115.46, 53.16, 52.06; LRMS (ESI) m/z: 333 ($[M+H]^+$, (Br^{81})), 331 ($[M+H]^+$, (Br^{79})).

According to ***General Procedure A***, the reaction gave two stereoisomers (*Z*-**1af**/*E*-**1af** = 1.3/1, 872.1 mg, 88% combined yield), which were separable by chromatography.

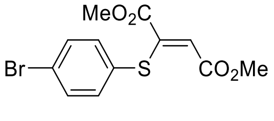

dimethyl 2-((4-bromophenyl)thio)fumarate (*Z*-**1af**): White solid. ^{1}H NMR (400 MHz, $CDCl_3$) δ 7.46 (d, *J* = 8.4 Hz, 2H), 7.30 (d, *J* = 8.4 Hz, 2H), 6.44 (s, 1H), 3.80 (s, 3H), 3.42 (s, 3H); ^{13}C NMR (101 MHz, $CDCl_3$) δ 165.55, 164.63, 148.65, 134.78, 132.37, 131.42, 123.56, 120.16, 52.98, 52.21; LRMS (ESI) m/z: 333 ($[M+H]^+$, (Br^{81})), 331 ($[M+H]^+$, (Br^{79})).

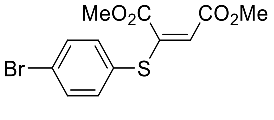

dimethyl 2-((4-bromophenyl)thio)maleate (*E*-**1af**): White solid. ^{1}H NMR (400 MHz, $CDCl_3$) δ 7.56 (d, *J* = 8.4 Hz, 2H), 7.41 (d, *J* = 8.4 Hz, 2H), 5.55 (s, 1H), 3.73 (s, 3H), 3.68 (s, 3H); ^{13}C NMR (101 MHz, $CDCl_3$) δ 165.20, 164.10, 150.25, 137.09, 133.19, 126.46, 125.49, 114.75, 53.20, 52.09; LRMS (ESI) m/z: 333 ($[M+H]^+$, (Br^{81})), 331 ($[M+H]^+$, (Br^{79})).

According to ***General Procedure A***, the reaction gave two stereoisomers (*Z*-**1ag**/*E*-**1ag** = 0.9/1, 565.9 mg, 71% combined yield), which were separable by chromatography.

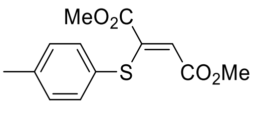

dimethyl 2-(*p*-tolylthio)fumarate (*Z*-1ag): White solid. ^{1}H NMR (400 MHz, CDCl3) δ 7.34 (d, *J* = 8.1 Hz, 2H), 7.13 (d, *J* = 8.0 Hz, 2H), 6.30 (s, 1H), 3.79 (s, 3H), 3.35 (s, 3H), 2.33 (s, 3H); ^{13}C NMR (101 MHz, CDCl3) δ 165.74, 164.98, 150.92, 139.43, 133.78, 129.92, 128.28, 117.82, 52.69, 52.02, 21.36; LRMS (ESI) m/z: 267 $[M+H]^+$. The characterization data of this compound were in accordance with the published ones.[2]

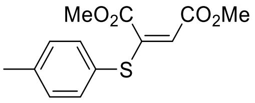

dimethyl 2-(*p*-tolylthio)maleate (*E*-1ag): White solid. ^{1}H NMR (400 MHz, CDCl3) δ 7.42 (d, *J* = 8.1 Hz, 2H), 7.23 (d, *J* = 7.9 Hz, 2H), 5.43 (s, 1H), 3.74 (s, 3H), 3.65 (s, 3H), 2.38 (s, 3H); ^{13}C NMR (101 MHz, CDCl3) δ 165.62, 164.27, 152.11, 141.13, 135.71, 130.76, 123.53, 113.24, 53.07, 51.89, 21.47; LRMS (ESI) m/z: 267 $[M+H]^+$.

According to ***General Procedure A***, the reaction gave two stereoisomers (*Z*-**1ah**/*E*-**1ah** = 2.8/1, 613.6 mg, 72% combined yield), which were separable by chromatography.

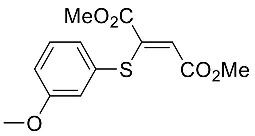

dimethyl 2-((3-methoxyphenyl)thio)fumarate (*Z*-1ah): Yellow oil. ^{1}H NMR (400 MHz, CDCl3) δ 7.26-7.19 (m, 1H), 7.00 (d, *J* = 7.7 Hz, 1H), 6.97 (s, 1H), 6.86 (d, *J* = 8.3 Hz, 1H), 6.40 (s, 1H), 3.79 (s, 3H), 3.78 (s, 3H), 3.39 (s, 3H); ^{13}C NMR (101 MHz, CDCl3) δ 165.62, 165.00, 159.87, 149.55, 133.40, 129.99, 125.31, 119.48, 117.92, 115.28, 55.52, 52.83, 52.07; LRMS (ESI) m/z: 283 $[M+H]^+$.

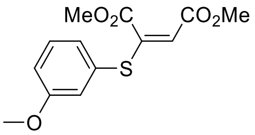

dimethyl 2-((3-methoxyphenyl)thio)maleate (*E*-1ah): Pale yellow oil. ^{1}H NMR (400 MHz, CDCl3) δ 7.36-7.30 (m, 1H), 7.13 (d, *J* = 7.5 Hz, 1H), 7.07 (s, 1H), 6.99 (dd, *J* = 8.3, 2.2 Hz, 1H), 5.51 (s, 1H), 3.81 (s, 3H), 3.75 (s, 3H), 3.66 (s, 3H); ^{13}C NMR (101 MHz, CDCl3) δ 165.52, 164.25, 160.42, 151.12, 130.72, 128.16, 127.71, 120.26, 116.92, 114.06, 55.61, 53.14, 51.95; LRMS (ESI) m/z: 283 $[M+H]^+$.

According to ***General Procedure A***, the reaction gave two stereoisomers (*Z*-**1ai**/*E*-**1ai** = 4.4/1, 739.5 mg, 87% combined yield), which were separable by chromatography.

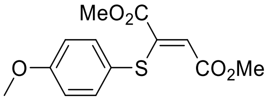

dimethyl 2-((4-methoxyphenyl)thio)fumarate (*Z*-1ai): White solid. ^{1}H NMR (400 MHz, CDCl3) δ 7.40 (d, *J* = 8.6 Hz, 2H), 6.85 (d, *J* = 8.6 Hz, 2H), 6.21 (s, 1H), 3.80 (s, 3H), 3.79 (s, 3H), 3.35 (s, 3H); ^{13}C NMR (101 MHz, CDCl3) δ 165.83, 164.96, 160.69, 152.03, 136.12, 121.85, 116.45, 114.65, 55.48, 52.70, 52.00; LRMS (ESI) m/z: 283 $[M+H]^+$.

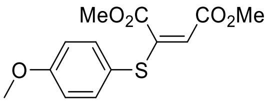

dimethyl 2-((4-methoxyphenyl)thio)maleate (*E*-1ai): White solid. ^{1}H NMR (400 MHz, CDCl3) δ 7.46 (d, *J* = 8.6 Hz, 2H), 6.94 (d, *J* = 8.6 Hz, 2H), 5.40 (s, 1H), 3.83 (s, 3H), 3.74 (s, 3H), 3.65 (s, 3H); ^{13}C NMR (101 MHz, CDCl3) δ 165.67, 164.30, 161.60, 152.81, 137.57, 117.35, 115.54, 112.85, 55.56, 53.11, 51.91; LRMS (ESI) m/z: 283 $[M+H]^+$.

According to ***General Procedure A***, the reaction gave two stereoisomers (*Z*-**1aj**/*E*-**1aj** = 4.4/1, 693.6 mg, 82% combined yield), which were separable by chromatography.

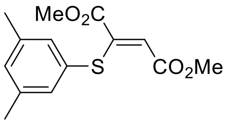

dimethyl 2-((3,5-dimethylphenyl)thio)fumarate (*Z*-1aj): Yellow oil. ^{1}H NMR (400 MHz, $CDCl_3$) δ 7.05 (s, 2H), 6.94 (s, 1H), 6.35 (s, 1H), 3.79 (s, 3H), 3.37 (s, 3H), 2.28 (s, 6H); ^{13}C NMR (101 MHz, $CDCl_3$) δ 165.69, 165.06, 150.34, 138.85, 131.61, 130.78, 130.74, 118.64, 52.68, 52.03, 21.21; LRMS (ESI) m/z: 281 $[M+H]^+$.

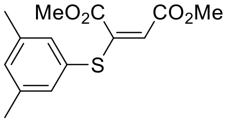

dimethyl 2-((3,5-dimethylphenyl)thio)maleate (*E*-1aj): White solid. ^{1}H NMR (400 MHz, $CDCl_3$) δ 7.16 (s, 2H), 7.07 (s, 1H), 5.44 (s, 1H), 3.77 (s, 3H), 3.66 (s, 3H), 2.32 (s, 6H); ^{13}C NMR (101 MHz, $CDCl_3$) δ 165.74, 164.34, 151.94, 139.79, 133.15, 132.39, 126.50, 113.49, 53.10, 51.92, 21.22; LRMS (ESI) m/z: 281 $[M+H]^+$.

According to ***General Procedure A***, the reaction gave two stereoisomers (*Z*-**1ak**/*E*-**1ak** = 0.9/1, 589.4 mg, 65% combined yield), which were separable by chromatography.

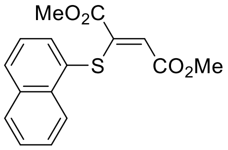

dimethyl 2-(naphthalen-1-ylthio)fumarate (*Z*-1ak): Pale yellow solid. ^{1}H NMR (400 MHz, $CDCl_3$) δ 8.40 (d, *J* = 8.4 Hz, 1H), 7.90-7.82 (m, 2H), 7.72 (d, *J* = 7.2 Hz, 1H), 7.62-7.56 (m, 1H), 7.56-7.50 (m, 1H), 7.46-7.39 (m, 1H), 6.40 (s, 1H), 3.84 (s, 3H), 2.98 (s, 3H); ^{13}C NMR (101 MHz, $CDCl_3$) δ 165.91, 164.78, 150.92, 134.39, 134.23, 134.05, 130.51, 129.20, 128.49, 127.25, 126.70, 126.07, 125.50, 118.28, 52.34, 52.07; LRMS (ESI) m/z: 303 $[M+H]^+$.

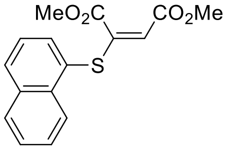

dimethyl 2-(naphthalen-1-ylthio)maleate (*E*-1ak): Pale yellow solid. ^{1}H NMR (400 MHz, $CDCl_3$) δ 8.35 (d, *J* = 8.3 Hz, 1H), 8.00 (d, *J* = 8.3 Hz, 1H), 7.91 (d, *J* = 8.0 Hz, 1H), 7.87 (d, *J* = 7.1 Hz, 1H), 7.66-7.60 (m, 1H), 7.60-7.54 (m, 1H), 7.54-7.48 (m, 1H), 5.20 (s, 1H), 3.75 (s, 3H), 3.59 (s, 3H); ^{13}C NMR (101 MHz, $CDCl_3$) δ 165.74, 164.18, 150.79, 136.39, 134.46, 134.12, 132.21, 128.90, 128.15, 127.10, 125.97, 125.52, 124.29, 114.01, 53.14, 51.85; LRMS (ESI) m/z: 303 $[M+H]^+$.

According to ***General Procedure A***, the reaction gave two stereoisomers (*Z*-**1al**/*E*-**1al** = 1.1/1, 559.0 mg, 62% combined yield), which were separable by chromatography.

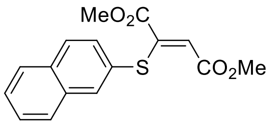

dimethyl 2-(naphthalen-2-ylthio)fumarate (*Z*-1al): Pale yellow solid. ^{1}H NMR (400 MHz, $CDCl_3$) δ 7.97 (s, 1H), 7.85-7.76 (m, 3H), 7.54-7.44 (m, 3H), 6.46 (s, 1H), 3.81 (s, 3H), 3.26 (s, 3H); ^{13}C NMR (101 MHz, $CDCl_3$) δ 165.66, 164.96, 149.63, 133.51, 133.04, 132.90, 129.87, 129.52, 128.83, 127.90, 127.88, 127.18, 126.98, 119.61, 52.79, 52.10; LRMS (ESI) m/z: 303 $[M+H]^+$. The characterization data of this compound were in accordance with the published ones.[2]

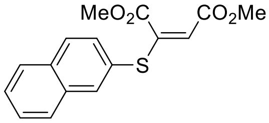

dimethyl 2-(naphthalen-2-ylthio)maleate (*E*-1al): White solid. ^{1}H NMR (400 MHz, $CDCl_3$) δ 8.11 (s, 1H), 7.92-7.82 (m, 3H), 7.62-7.52 (m, 3H), 5.52 (s, 1H), 3.73 (s, 3H), 3.65 (s, 3H); ^{13}C NMR (101 MHz, $CDCl_3$) δ 165.58, 164.24, 151.25, 136.06, 133.81, 131.22, 129.78, 128.19, 127.98, 127.17, 124.51, 114.33, 53.15, 51.95; LRMS (ESI) m/z: 303 $[M+H]^+$.

According to ***General Procedure A***, the reaction gave two stereoisomers (*Z*-**1am**/*E*-**1am** = 0.4/1, 623.2 mg, 81% combined yield), which were separable by chromatography.

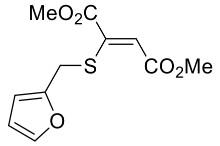

dimethyl 2-((furan-2-ylmethyl)thio)fumarate (*Z*-1am): White solid. ^{1}H NMR (400 MHz, $CDCl_3$) δ 7.31 (s, 1H), 6.45 (s, 1H), 6.27 (s, 1H), 6.15 (d, *J* = 2.8 Hz, 1H), 4.16 (s, 2H), 3.83 (s, 3H), 3.75 (s, 3H); ^{13}C NMR (101 MHz, $CDCl_3$) δ 165.57, 164.53, 150.35, 147.17, 142.67, 121.32, 110.66, 108.54, 53.19, 51.99, 29.36; LRMS (ESI) m/z: 257 $[M+H]^+$.

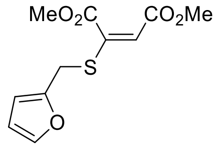

dimethyl 2-((furan-2-ylmethyl)thio)maleate (*E*-1am): Pale yellow oil. ^{1}H NMR (400 MHz, $CDCl_3$) δ 7.37 (s, 1H), 6.32 (s, 1H), 6.30-6.26 (m, 1H), 5.87 (s, 1H), 4.07 (s, 2H), 3.87 (s, 3H), 3.71 (s, 3H); ^{13}C NMR (101 MHz, $CDCl_3$) δ 165.68, 164.11, 148.67, 148.26, 142.97, 114.61, 110.91, 109.20, 53.27, 52.05, 29.07; LRMS (ESI) m/z: 257 $[M+H]^+$.

According to ***General Procedure A***, the reaction gave two stereoisomers (*Z*-**1an**/*E*-**1an** = 0.5/1, 768.5 mg, 96% combined yield), which were separable by chromatography.

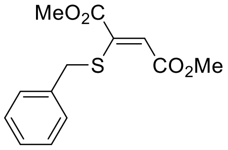

dimethyl 2-(benzylthio)fumarate (*Z*-1an): Pale yellow oil. ^{1}H NMR (400 MHz, $CDCl_3$) δ 7.32-7.27 (m, 4H), 7.26-7.21 (m, 1H), 6.34 (s, 1H), 4.11 (s, 2H), 3.75 (s, 6H); ^{13}C NMR (101 MHz, $CDCl_3$) δ 165.65, 164.78, 148.34, 136.37, 129.32, 128.73, 127.70, 119.98, 53.10, 51.91, 37.13; LRMS (ESI) m/z: 267 $[M+H]^+$.

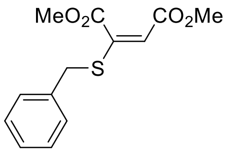

dimethyl 2-(benzylthio)maleate (*E*-1an): White solid. ^{1}H NMR (400 MHz, $CDCl_3$) δ 7.39-7.27 (m, 5H), 5.79 (s, 1H), 4.05 (s, 2H), 3.86 (s, 3H), 3.71 (s, 3H); ^{13}C NMR (101 MHz, $CDCl_3$) δ 165.87, 164.18, 149.97, 134.29, 129.08, 129.00, 128.16, 113.53, 53.26, 52.00, 36.71; LRMS (ESI) m/z: 267 $[M+H]^+$.

According to ***General Procedure A***, the reaction gave two stereoisomers (*Z*-**1ao**/*E*-**1ao** = 0.5/1, 806.9 mg, 96% combined yield), which were separable by chromatography.

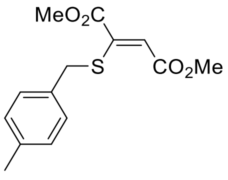

dimethyl 2-((4-methylbenzyl)thio)fumarate (*Z*-1ao): Pale yellow oil. ^{1}H NMR (400 MHz, $CDCl_3$) δ 7.17 (d, *J* = 7.7 Hz, 2H), 7.09 (d, *J* = 7.7 Hz, 2H), 6.33 (s, 1H), 4.07 (s, 2H), 3.76 (s, 3H), 3.74 (s, 3H), 2.31 (s, 3H); ^{13}C NMR (101 MHz, $CDCl_3$) δ 165.65, 164.83, 148.56, 137.41, 133.23, 129.41, 129.20, 119.77, 53.09, 51.88, 36.89, 21.23; LRMS (ESI) m/z: 281 $[M+H]^+$.

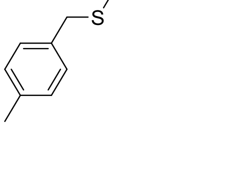

dimethyl 2-((4-methylbenzyl)thio)maleate (*E*-1ao): White solid. ^{1}H NMR (400 MHz, $CDCl_3$) δ 7.23 (d, *J* = 7.8 Hz, 2H), 7.14 (d, *J* = 7.8 Hz, 2H), 5.78 (s, 1H), 4.01 (s, 2H), 3.86 (s, 3H), 3.70 (s, 3H), 2.33 (s, 3H); ^{13}C NMR (101 MHz, $CDCl_3$) δ 165.92, 164.20, 150.26, 137.99, 131.07, 129.68, 128.99, 113.23, 53.24, 51.97, 36.50, 21.26; LRMS (ESI) m/z: 281 $[M+H]^+$.

According to ***General Procedure A***, the reaction gave two stereoisomers (*Z*-**1ap**/*E*-**1ap** = 0.5/1, 877.8 mg, 99% combined yield), which were separable by chromatography.

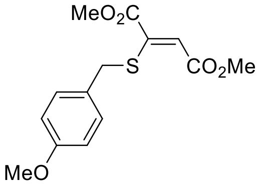

dimethyl 2-((4-methoxybenzyl)thio)fumarate (*Z*-1ap): Pale yellow solid. ^{1}H NMR (400 MHz, $CDCl_3$) δ 7.21 (d, *J* = 8.2 Hz, 2H), 6.82 (d, *J* = 8.2 Hz, 2H), 6.33 (s, 1H), 4.06 (s, 2H), 3.78 (s, 3H), 3.77 (s, 3H), 3.74 (s, 3H); ^{13}C NMR (101 MHz, $CDCl_3$) δ 165.66, 164.84, 159.15, 148.59, 130.49, 128.22, 119.71, 114.14, 55.39, 53.11, 51.88, 36.63; LRMS (ESI) m/z: 297 $[M+H]^+$.

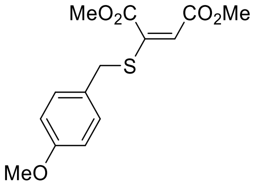

dimethyl 2-((4-methoxybenzyl)thio)maleate (*E*-1ap): White solid. ^{1}H NMR (400 MHz, $CDCl_3$) δ 7.26 (d, *J* = 8.3 Hz, 2H), 6.86 (d, *J* = 8.3 Hz, 2H), 5.78 (s, 1H), 4.01 (s, 2H), 3.86 (s, 3H), 3.80 (s, 3H), 3.71 (s, 3H); ^{13}C NMR (101 MHz, $CDCl_3$) δ 165.93, 164.21, 159.51, 150.28, 130.32, 125.96, 114.41, 113.23, 55.43, 53.25, 51.98, 36.27; LRMS (ESI) m/z: 297 $[M+H]^+$.

According to ***General Procedure A***, the reaction gave two stereoisomers (*Z*-**1aq**/*E*-**1aq** = 0.6/1, 832.1 mg, 99% combined yield), which were separable by chromatography.

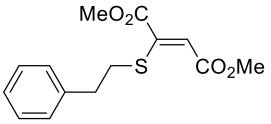

dimethyl 2-(phenethylthio)fumarate (*Z*-1aq): Pale yellow oil. ^{1}H NMR (400 MHz, $CDCl_3$) δ 7.33-7.26 (m, 2H), 7.25-7.19 (m, 1H), 7.17 (d, *J* = 7.6 Hz, 2H), 6.38 (s, 1H), 3.84 (s, 3H), 3.77 (s, 3H), 3.14-3.05 (m, 2H), 2.92-2.84 (m, 2H); ^{13}C NMR (101 MHz, $CDCl_3$) δ 165.68, 164.93, 148.64, 139.60, 128.73, 128.61, 126.77, 119.82, 53.23, 51.91, 36.51, 34.12; LRMS (ESI) m/z: 281 $[M+H]^+$.

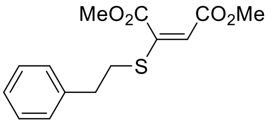

dimethyl 2-(phenethylthio)maleate (*E*-1aq): Colourless oil. ^{1}H NMR (400 MHz, $CDCl_3$) δ 7.36-7.29 (m, 2H), 7.28-7.23 (m, 1H), 7.20 (d, *J* = 7.6 Hz, 2H), 5.79 (s, 1H), 3.89 (s, 3H), 3.72 (s, 3H), 3.07-3.00 (m, 2H), 3.00-2.93 (m, 2H); ^{13}C NMR (101 MHz, $CDCl_3$) δ 166.04, 164.18, 150.08, 139.11, 128.86, 128.61, 127.03, 113.46, 53.28, 52.00, 34.78, 33.34; LRMS (ESI) m/z: 281 $[M+H]^+$.

According to ***General Procedure A***, the reaction gave two stereoisomers (*Z*-**1ar**/*E*-**1ar** = 0.7/1, 716.5 mg, 97% combined yield), which were separable by chromatography.

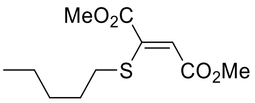

dimethyl 2-(pentylthio)fumarate (*Z*-1ar): Pale yellow oil. ^{1}H NMR (400 MHz, $CDCl_3$) δ 6.31 (s, 1H), 3.84 (s, 3H), 3.75 (s, 3H), 2.81 (t, *J* = 7.5 Hz, 2H), 1.60-1.53 (m, 2H), 1.40-1.24 (m, 4H), 0.87 (t, *J* = 7.0 Hz, 3H); ^{13}C NMR (101 MHz, $CDCl_3$) δ 165.77, 165.11, 149.62, 118.81, 53.11, 51.84, 32.77, 30.97, 29.47, 22.30, 13.99; LRMS (ESI) m/z: 247 $[M+H]^+$. The characterization data of this compound were in accordance with the published ones.[2]

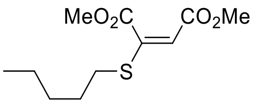

dimethyl 2-(pentylthio)maleate (*E*-1ar): Colourless oil. ^{1}H NMR (400 MHz, $CDCl_3$) δ 5.71 (s, 1H), 3.88 (s, 3H), 3.70 (s, 3H), 2.79 (t, *J* = 7.4 Hz, 2H), 1.74-1.63 (m, 2H), 1.45-1.27 (m, 4H), 0.90 (t, *J* = 7.0 Hz, 3H); ^{13}C NMR (101 MHz, $CDCl_3$) δ 166.20, 164.25, 150.95, 112.46, 53.22, 51.93, 31.97, 31.06, 27.84, 22.26, 13.98; LRMS (ESI) m/z: 247 $[M+H]^+$.

According to ***General Procedure A***, the reaction gave two stereoisomers (*Z*-**1as**/*E*-**1as** = 1.1/1, 764.8 mg, 99% combined yield), which were separable by chromatography.

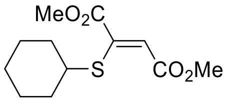

dimethyl 2-(cyclohexylthio)fumarate (*Z*-1as): Yellow oil. ^{1}H NMR (400 MHz, $CDCl_3$) δ 6.37 (s, 1H), 3.85 (s, 3H), 3.75 (s, 3H), 3.31-3.19 (m, 1H), 1.93-1.84 (m, 2H), 1.80-1.71 (m, 2H), 1.61-1.55 (m, 1H), 1.43-1.17 (m, 5H); ^{13}C NMR (101 MHz, $CDCl_3$) δ 165.66, 165.42, 148.11, 120.44, 53.17, 51.85, 45.11, 33.94, 26.06, 25.49; LRMS (ESI) m/z: 259 $[M+H]^+$. The characterization data of this compound were in accordance with the published ones.[2]

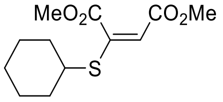

dimethyl 2-(cyclohexylthio)maleate (*E*-1as): Pale yellow oil. ^{1}H NMR (400 MHz, $CDCl_3$) δ 5.77 (s, 1H), 3.87 (s, 3H), 3.70 (s, 3H), 3.17-3.05 (m, 1H), 2.09-1.97 (m, 2H), 1.84-1.70 (m, 2H), 1.63-1.56 (m, 1H), 1.50-1.23 (m, 5H); ^{13}C NMR (101 MHz, $CDCl_3$) δ 166.34, 164.30, 149.92, 113.41, 53.17, 51.92, 44.93, 32.84, 25.83, 25.56; LRMS (ESI) m/z: 259 $[M+H]^+$.

According to ***General Procedure A***, the reaction only gave the *E* stereoisomer.

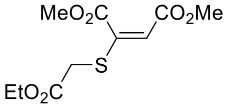

dimethyl 2-((2-ethoxy-2-oxoethyl)thio)maleate (*E*-1at): Colourless oil (231.9 mg, 29% yield). ^{1}H NMR (400 MHz, $CDCl_3$) δ 5.93 (s, 1H), 4.22 (q, J = 7.1 Hz, 2H), 3.87 (s, 3H), 3.71 (s, 3H), 3.59 (s, 2H), 1.29 (t, J = 7.1 Hz, 3H); ^{13}C NMR (101 MHz, $CDCl_3$) δ 167.64, 165.37, 164.02, 147.45, 115.58, 62.39, 53.33, 52.12, 34.12, 14.19; LRMS (ESI) m/z: 263 $[M+H]^+$. The *E* configuration of this compound was confirmed by comparing its characterization data with those of its *Z* isomer.[3]

According to ***General Procedure A***, the reaction gave two stereoisomers (*Z*-**1au**/*E*-**1au** = 4/1, 833.4 mg, 99% combined yield), which were separable by chromatography.

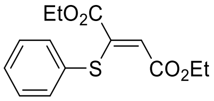

diethyl 2-(phenylthio)fumarate (*Z*-1au): Yellow oil. ^{1}H NMR (400 MHz, $CDCl_3$) δ 7.50-7.41 (m, 2H), 7.36-7.27 (m, 3H), 6.39 (s, 1H), 4.26 (q, J = 7.1 Hz, 2H), 3.79 (q, J = 7.1 Hz, 2H), 1.32 (t, J = 7.1 Hz, 3H), 0.90 (t, J = 7.1 Hz, 3H); ^{13}C NMR (101 MHz, $CDCl_3$) δ 165.28, 164.58, 149.61, 133.35, 132.51, 129.15, 128.91, 119.69, 62.30, 61.06, 14.38, 13.63; LRMS (ESI) m/z: 281 $[M+H]^+$. The characterization data of this compound were in accordance with the published ones.[2]

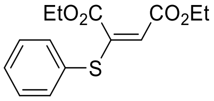

diethyl 2-(phenylthio)maleate (*E*-1au): Pale yellow oil. ^{1}H NMR (400 MHz, $CDCl_3$) δ 7.56 (d, J = 7.5 Hz, 2H), 7.48-7.37 (m, 3H), 5.53 (s, 1H), 4.19-4.08 (m, 4H), 1.26-1.17 (m, 6H); ^{13}C NMR (101 MHz, $CDCl_3$) δ 164.96, 163.77, 150.84, 135.80, 130.48, 129.85, 127.59, 114.58, 62.34, 60.88, 14.24, 13.93; LRMS (ESI) m/z: 281 $[M+H]^+$.

According to ***General Procedure A***, the reaction gave two stereoisomers (*Z*-**1av**/*E*-**1av** = 5/1, 910.4 mg, 98% combined yield), which were separable by chromatography.

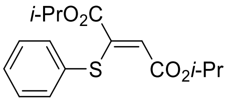

diisopropyl 2-(phenylthio)fumarate (*Z*-1av): Yellow oil. ^{1}H NMR (400 MHz, $CDCl_3$) δ 7.49-7.40 (m, 2H), 7.34-7.27 (m, 3H), 6.36 (s, 1H), 5.18-5.07 (m, 1H), 4.73-4.62 (m, 1H), 1.30 (d, J = 6.2 Hz, 6H), 0.89 (d, J = 6.2 Hz, 6H); ^{13}C NMR (101 MHz, $CDCl_3$) δ 164.85, 164.18, 149.32, 133.23, 132.69, 129.18, 128.78, 120.38, 70.43, 68.67, 22.03, 21.22; LRMS (ESI) m/z: 309 $[M+H]^+$.

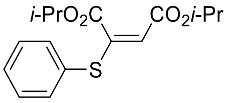

diisopropyl 2-(phenylthio)maleate (*E*-1av): Pale yellow oil. ^{1}H NMR (400 MHz, $CDCl_3$) δ 7.56 (d, J = 7.5 Hz, 2H), 7.48-7.37 (m, 3H), 5.56 (s, 1H), 5.05-4.93 (m, 2H), 1.20 (d, J = 6.2 Hz, 6H), 1.17 (d, J = 6.3 Hz, 6H); ^{13}C NMR (101 MHz, $CDCl_3$) δ 164.39, 163.26, 150.33, 135.77, 130.32, 129.77, 127.80, 115.26, 70.24, 68.35, 21.90, 21.54; LRMS (ESI) m/z: 309 $[M+H]^+$.

According to ***General Procedure A***, the reaction only gave the *Z* stereoisomer.

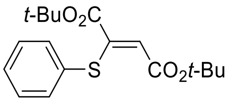

di-*tert*-butyl 2-(phenylthio)fumarate (*Z*-1aw): Pale yellow oil. (367.1 mg, 36% yield). ^{1}H NMR (400 MHz, $CDCl_3$) δ 7.51-7.42 (m, 2H), 7.35-7.28 (m, 3H), 6.27 (s, 1H), 1.52 (s, 9H), 1.09 (s, 9H); ^{13}C NMR (101 MHz, $CDCl_3$) δ 164.86, 163.70, 149.31, 133.54, 132.96, 129.14, 128.70, 121.14, 83.26, 81.72, 28.33, 27.43; LRMS (ESI) m/z: 337 $[M+H]^+$.

According to ***General Procedure A***, the reaction only gave the *E* stereoisomer.

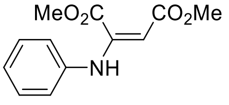

dimethyl 2-(phenylamino)maleate (*E*-1ba): Yellow oil. (342.0 mg, 48% yield). ^{1}H NMR (400 MHz, $CDCl_3$) δ 9.67 (s, 1H), 7.31-7.25 (m, 2H), 7.13-7.05 (m, 1H), 6.90 (d, J = 7.9 Hz, 2H), 5.39 (s, 1H), 3.74 (s, 3H), 3.69 (s, 3H); ^{13}C NMR (101 MHz, $CDCl_3$) δ 170.00, 164.98, 148.14, 140.40, 129.27, 124.37, 120.83, 93.70, 52.89, 51.33; LRMS (ESI) m/z: 236 $[M+H]^+$. The characterization data of this compound were in accordance with the published ones.[4]

According to ***General Procedure A***, the reaction gave two stereoisomers (*Z*-**1bb**/*E*-**1bb** = 0.8/1, 683.8 mg, 96% combined yield), which were inseparable by chromatography.

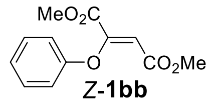

Z-**1bb**

+

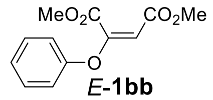

E-**1bb**

Z-**1bb**/*E*-**1bb** = 0.8/1

Colourless oil. ^{1}H NMR (400 MHz, $CDCl_3$) δ 7.45-7.37 (m, 2.12H), 7.34-7.23 (m, 3.05H), 7.11 (d, J = 8.2 Hz, 2.04H), 7.10-7.04 (m, 0.92H), 6.95 (d, J = 8.3 Hz, 1.71H), 6.59 (s, 0.83H), 5.14 (s, 1.00H), 3.92 (s, 3.19H), 3.73 (s, 2.67H), 3.70 (s, 2.69H), 3.67 (s, 3.23H); ^{13}C NMR (101 MHz, $CDCl_3$) δ 166.02, 164.01, 163.45, 162.79, 161.02, 156.71, 153.10, 149.96, 130.33, 129.77, 126.50, 123.53, 120.94, 116.25, 115.11, 98.98, 53.21, 53.12, 52.09, 51.85; LRMS (ESI) m/z: 237 $[M+H]^+$.

According to ***General Procedure A***, the reaction gave two stereoisomers (*Z*-**1bc**/*E*-**1bc** = 1/1, 692.6 mg, 87% combined yield), which were inseparable by chromatography.

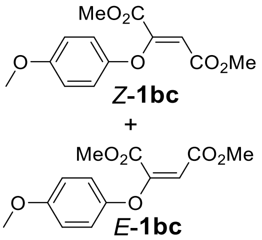

Z-**1bc** + *E*-**1bc**

Z-**1bc**/*E*-**1bc** = 1/1

Yellow oil. ^{1}H NMR (400 MHz, $CDCl_3$) δ 7.06-7.00 (m, 2.10H), 6.94-6.87 (m, 4.15H), 6.85-6.79 (m, 2.08H), 6.50 (s, 0.98H), 5.08 (s, 1.00H), 3.92 (s, 3.14H), 3.80 (s, 3.13H), 3.76 (s, 3.17H), 3.72 (s, 3.12H), 3.71 (s, 3.14H), 3.66 (s, 3.16H); ^{13}C NMR (101 MHz, $CDCl_3$) δ 166.17, 164.18, 163.59, 162.88, 161.93, 157.81, 155.85, 150.53, 150.50, 146.35, 121.95, 117.52, 115.23, 114.76, 114.11, 98.01, 55.76, 55.73, 53.21, 53.08, 52.09, 51.82; LRMS (ESI) m/z: 267 $[M+H]^+$.

According to ***General Procedure A***, the reaction gave two stereoisomers (*Z*-**1bd**/*E*-**1bd** = 5.9/1, 801.6 mg, 89% combined yield), which were separable by chromatography.

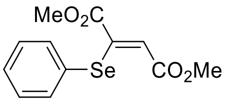

dimethyl 2-(phenylselanyl)fumarate (*Z*-1bd): Pale yellow solid. ^{1}H NMR (400 MHz, $CDCl_3$) δ 7.60 (d, *J* = 7.1 Hz, 2H), 7.40-7.35 (m, 1H), 7.35-7.30 (m, 2H), 6.52 (s, 1H), 3.81 (s, 3H), 3.21 (s, 3H); ^{13}C NMR (101 MHz, $CDCl_3$) δ 166.65, 165.87, 151.27, 136.34, 129.35, 129.14, 129.11, 118.17, 52.40, 52.22; LRMS (ESI) m/z: LRMS (ESI) m/z: 301 ($[M+H]^+$, (Se^{80})), 299 ($[M+H]^+$, (Se^{78})).

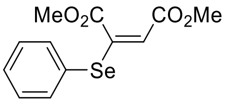

dimethyl 2-(phenylselanyl)maleate (*E*-1bd): Pale yellow solid. ^{1}H NMR (400 MHz, $CDCl_3$) δ 7.64 (d, *J* = 7.4 Hz, 2H), 7.48-7.43 (m, 1H), 7.42-7.36 (m, 2H), 5.75 (s, 1H), 3.67 (s, 3H), 3.65 (s, 3H); ^{13}C NMR (101 MHz, $CDCl_3$) δ 166.41, 164.01, 147.50, 137.13, 130.29, 129.96, 124.54, 118.20, 52.86, 52.05; LRMS (ESI) m/z: LRMS (ESI) m/z: 301 ($[M+H]^+$, (Se^{80})), 299 ($[M+H]^+$, (Se^{78})).

Procedure for the Synthesis of *E*-1ax

E-**1ax** was prepared similarly according to the literature procedure.[5] To a solution of thiophenol (3 mmol), *N*-methylmaleimide (3.3 mmol) and HBF_4 (3 mmol) in DMSO (15 mL) was added CuI (0.6 mmol), and the resulting mixture was stirred at 120 °C under air atmosphere for 18 h. Then water (45 mL) was added, and the mixture was extracted with EtOAc (15 mL × 3), and the combined organic phase was washed with brine, dried over $MgSO_4$, and concentrated. The residue was purified by flash chromatography (Hexane/EtOAc: 8/1→Hexane/EtOAc: 4/1) on silica gel to give the desired product *E*-**1ax** (171.3 mg, 26% yield) and **2ax** (159.6 mg, 24% yield).

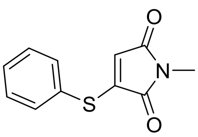

1-methyl-3-(phenylthio)-1*H*-pyrrole-2,5-dione (*E*-1ax): Yellow solid (171.3 mg, 26% yield). ^{1}H NMR (400 MHz, $CDCl_3$) δ 7.58-7.52 (m, 2H), 7.50-7.43 (m, 3H), 5.65 (s, 1H), 3.01 (s, 3H); ^{13}C NMR (101 MHz, $CDCl_3$) δ 169.67, 168.09, 152.67, 134.46, 130.56, 130.33, 127.45, 119.00, 24.12; LRMS (ESI) m/z: 220 $[M+H]^+$.

General Procedure for the Synthesis of α-Sulfonyl Fumarates 1ay and 1az

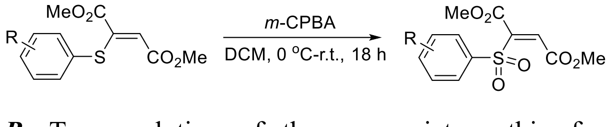

General Procedure B: To a solution of the appropriate α-thio fumarates (1 mmol) in dichloromethane (20 mL) at 0 °C was added *m*-CPBA (2.1 mmol). After addition, the resulting

mixture was stirred at room temperature for 18 h. Then the reaction mixture was washed with saturated aqueous Na_2CO_3 solution, dried over $MgSO_4$, and concentrated. The residue was purified by flash chromatography (Hexane/EtOAc: 4/1→Hexane/EtOAc: 2/1) on silica gel to give the desired products.

According to ***General Procedure B***.

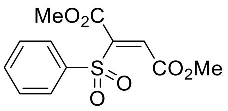

dimethyl 2-(phenylsulfonyl)fumarate (*Z*-1ay): Colourless oil (70.1 mg, 25% yield). ^{1}H NMR (400 MHz, $CDCl_3$) δ 7.91 (d, *J* = 8.0 Hz, 2H), 7.73-7.65 (m, 1H), 7.61-7.53 (m, 2H), 7.11 (s, 1H), 3.80 (s, 3H), 3.79 (s, 3H); ^{13}C NMR (101 MHz, $CDCl_3$) δ 163.23, 161.50, 147.44, 137.92, 134.78, 131.45, 129.47, 129.15, 53.59, 53.16; LRMS (ESI) m/z: 285 $[M+H]^+$.

According to ***General Procedure B***.

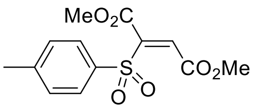

dimethyl 2-tosylfumarate (*Z*-1az): Colourless oil (95.2 mg, 32% yield). ^{1}H NMR (400 MHz, $CDCl_3$) δ 7.78 (d, *J* = 8.0 Hz, 2H), 7.36 (d, *J* = 8.0 Hz, 2H), 7.07 (s, 1H), 3.80 (s, 3H), 3.79 (s, 3H), 2.45 (s, 3H); ^{13}C NMR (101 MHz, $CDCl_3$) δ 163.30, 161.67, 147.86, 146.16, 134.83, 130.80, 130.14, 129.22, 53.59, 53.13, 21.91; LRMS (ESI) m/z: 299 $[M+H]^+$.

General Procedure for the Synthesis of Racemic α-Thio Succinates 2aa-2as and 2au-2aw

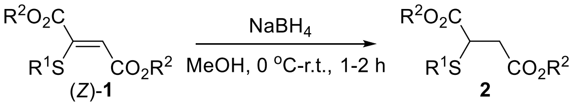

General Procedure C: To a solution of the appropriate α-thio fumarates (*Z*)-**1** (1 mmol) in MeOH (10 mL) at 0 °C was added $NaBH_4$ (1 mmol), then the reaction mixture was stirred at room temperature for 1 h and monitored by TLC [If the reaction did not complete in 1 h in some cases, another portion of $NaBH_4$ (1~4 mmol) was added and the reaction mixture was allowed to stir for another 1 h]. Upon completion as indicated by TLC, the reaction mixture was concentrated and a saturated aqueous solution of NH_4Cl (10 mL) was added. Then the resulting mixture was extracted with EtOAc (15 mL × 3), and the combined organic phase was washed with brine, dried over $MgSO_4$, and concentrated. The residue was purified by flash chromatography (Hexane/EtOAc: 16/1→Hexane/EtOAc: 8/1) on silica gel to give the desired products **2**.

According to ***General Procedure C***

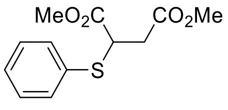

dimethyl 2-(phenylthio)succinate (2aa): Colourless oil (189.2 mg, 74% yield). ^{1}H NMR (400 MHz, $CDCl_3$) δ 7.52-7.43 (m, 2H), 7.36-7.30 (m, 3H), 4.02 (dd, *J* = 9.6, 5.7 Hz, 1H), 3.69 (s, 3H), 3.67 (s, 3H), 2.95 (dd, *J* = 17.0, 9.6 Hz, 1H), 2.74 (dd, *J* = 17.0, 5.7 Hz, 1H); ^{13}C NMR (101 MHz, $CDCl_3$) δ 171.60, 171.11, 134.27, 131.75, 129.21, 128.93, 52.60, 52.13, 45.76, 36.53; HRMS (ESI) m/z: $[M + H]^+$ Calcd for $C_{12}H_{15}O_4S^+$ 255.0686; Found 255.0680.

According to ***General Procedure C***

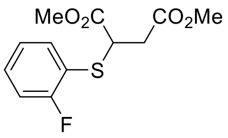

dimethyl 2-((2-fluorophenyl)thio)succinate (2ab): Colourless oil (228.4 mg, 84% yield). ^{1}H NMR (400 MHz, $CDCl_3$) δ 7.52-7.45 (m, 1H), 7.41-7.32 (m, 1H), 7.15-7.07 (m, 2H), 4.01 (dd, J = 9.8, 5.4 Hz, 1H), 3.67 (s, 6H), 2.94 (dd, J = 17.1, 9.9 Hz, 1H), 2.76 (dd, J = 17.1, 5.3 Hz, 1H); ^{13}C NMR (101 MHz, $CDCl_3$) δ 171.27, 171.00, 163.26 (d, J_{C-F} = 247.7 Hz), 137.37, 131.82 (d, J_{C-F} = 8.1 Hz), 124.71 (d, J_{C-F} = 4.0 Hz), 118.22 (d, J_{C-F} = 18.3 Hz), 116.18 (d, J_{C-F} = 23.3 Hz), 52.66, 52.17, 44.75 (d, J_{C-F} = 1.3 Hz), 36.23; ^{19}F NMR (376 MHz, $CDCl_3$) δ -106.13; HRMS (ESI) m/z: $[M + H]^+$ Calcd for $C_{12}H_{14}FO_4S^+$ 273.0591; Found 273.0587.

According to ***General Procedure C***

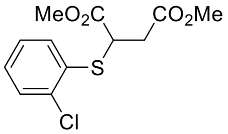

dimethyl 2-((2-chlorophenyl)thio)succinate (2ac): Colourless oil (230.9 mg, 80% yield). ^{1}H NMR (400 MHz, $CDCl_3$) δ 7.58 (dd, J = 7.2, 2.2 Hz, 1H), 7.44 (dd, J = 7.5, 1.9 Hz, 1H), 7.31-7.21 (m, 2H), 4.13 (dd, J = 10.0, 5.1 Hz, 1H), 3.68 (s, 3H), 3.68 (s, 3H), 3.02 (dd, J = 17.1, 10.0 Hz, 1H), 2.81 (dd, J = 17.0, 5.1 Hz, 1H); ^{13}C NMR (101 MHz, $CDCl_3$) δ 171.37, 170.99, 137.98, 135.45, 131.36, 130.26, 130.05, 127.44, 52.74, 52.24, 44.73, 36.11; HRMS (ESI) m/z: $[M + H]^+$ Calcd for $C_{12}H_{14}{}^{35}ClO_4S^+$ 289.0296; Found 289.0286.

According to ***General Procedure C***

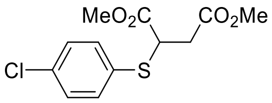

dimethyl 2-((4-chlorophenyl)thio)succinate (2ad): Colourless oil (276.1 mg, 96% yield). ^{1}H NMR (400 MHz, $CDCl_3$) δ 7.40 (d, J = 8.4 Hz, 2H), 7.30 (d, J = 8.4 Hz, 2H), 3.97 (dd, J = 9.3, 5.9 Hz, 1H), 3.70 (s, 3H), 3.68 (s, 3H), 2.93 (dd, J = 17.0, 9.4 Hz, 1H), 2.72 (dd, J = 17.0, 5.9 Hz, 1H); ^{13}C NMR (101 MHz, $CDCl_3$) δ 171.41, 170.98, 135.63, 135.40, 130.15, 129.43, 52.70, 52.23, 45.78, 36.34; HRMS (ESI) m/z: $[M + H]^+$ Calcd for $C_{12}H_{14}{}^{35}ClO_4S^+$ 289.0296; Found 289.0293.

According to ***General Procedure C***

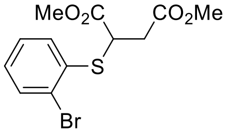

dimethyl 2-((2-bromophenyl)thio)succinate (2ae): Colourless oil (267.5 mg, 80% yield). ^{1}H NMR (400 MHz, $CDCl_3$) δ 7.65-7.53 (m, 2H), 7.31-7.25 (m, 1H), 7.19-7.12 (m, 1H), 4.13 (dd, J = 9.9, 5.1 Hz, 1H), 3.67 (s, 6H), 3.02 (dd, J = 17.1, 10.0 Hz, 1H), 2.81 (dd, J = 17.1, 5.1 Hz, 1H); ^{13}C NMR (101 MHz, $CDCl_3$) δ 171.28, 170.88, 134.80, 133.70, 133.52, 129.85, 128.32, 128.06, 52.70, 52.18, 45.04, 36.03; HRMS (ESI) m/z: $[M + H]^+$ Calcd for $C_{12}H_{14}{}^{79}BrO_4S^+$ 332.9791; Found 332.9791.

According to ***General Procedure C***

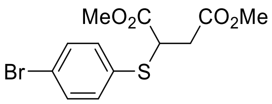

dimethyl 2-((4-bromophenyl)thio)succinate (2af): Colourless oil (311.8 mg, 94% yield). ^{1}H NMR (400 MHz, $CDCl_3$) δ 7.45 (d, *J* = 8.4 Hz, 2H), 7.33 (d, *J* = 8.4 Hz, 2H), 3.98 (dd, *J* = 9.4, 5.9 Hz, 1H), 3.70 (s, 3H), 3.68 (s, 3H), 2.93 (dd, *J* = 17.2, 9.2 Hz, 1H), 2.72 (dd, *J* = 17.0, 5.9 Hz, 1H); ^{13}C NMR (101 MHz, $CDCl_3$) δ 171.40, 170.96, 135.72, 132.38, 130.87, 123.53, 52.73, 52.24, 45.67, 36.35; HRMS (ESI) m/z: $[M + H]^+$ Calcd for $C_{12}H_{14}{}^{79}BrO_4S^+$ 332.9791; Found 332.9785.

According to ***General Procedure C***

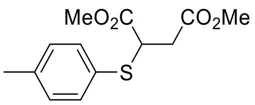

dimethyl 2-(*p*-tolylthio)succinate (2ag): Colourless oil (197.3 mg, 74% yield). ^{1}H NMR (400 MHz, $CDCl_3$) δ 7.36 (d, *J* = 7.9 Hz, 2H), 7.14 (d, *J* = 7.7 Hz, 2H), 3.94 (dd, *J* = 9.6, 5.7 Hz, 1H), 3.71 (s, 3H), 3.67 (s, 3H), 2.92 (dd, *J* = 17.0, 9.6 Hz, 1H), 2.72 (dd, *J* = 17.0, 5.6 Hz, 1H), 2.34 (s, 3H); ^{13}C NMR (101 MHz, $CDCl_3$) δ 171.67, 171.24, 139.45, 134.94, 130.02, 127.69, 52.59, 52.14, 45.86, 36.46, 21.37; HRMS (ESI) m/z: $[M + H]^+$ Calcd for $C_{13}H_{17}O_4S^+$ 269.0842; Found 269.0839.

According to ***General Procedure C***

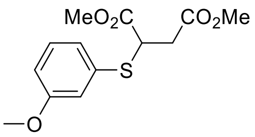

dimethyl 2-((3-methoxyphenyl)thio)succinate (2ah): Colourless oil (245.9 mg, 86% yield). ^{1}H NMR (400 MHz, $CDCl_3$) δ 7.26-7.20 (m, 1H), 7.08-7.00 (m, 2H), 6.87 (dd, *J* = 8.3, 2.2 Hz, 1H), 4.04 (dd, *J* = 9.5, 5.6 Hz, 1H), 3.80 (s, 3H), 3.71 (s, 3H), 3.68 (s, 3H), 2.97 (dd, *J* = 17.0, 9.6 Hz, 1H), 2.75 (dd, *J* = 17.0, 5.6 Hz, 1H); ^{13}C NMR (101 MHz, $CDCl_3$) δ 171.65, 171.14, 159.89, 132.96, 129.99, 126.05, 119.00, 114.83, 55.46, 52.69, 52.16, 45.70, 36.56; HRMS (ESI) m/z: $[M + H]^+$ Calcd for $C_{13}H_{17}O_5S^+$ 285.0791; Found 285.0788.

According to ***General Procedure C***

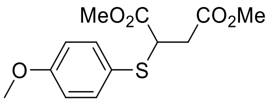

dimethyl 2-((4-methoxyphenyl)thio)succinate (2ai): Colourless oil (174.0 mg, 61% yield). ^{1}H NMR (400 MHz, $CDCl_3$) δ 7.40 (d, *J* = 8.3 Hz, 2H), 6.85 (d, *J* = 8.4 Hz, 2H), 3.87 (dd, *J* = 9.5, 5.8 Hz, 1H), 3.80 (s, 3H), 3.70 (s, 3H), 3.67 (s, 3H), 2.88 (dd, *J* = 17.0, 9.6 Hz, 1H), 2.70 (dd, *J* = 17.0, 5.7 Hz, 1H); ^{13}C NMR (101 MHz, $CDCl_3$) δ 171.64, 171.25, 160.80, 137.30, 121.42, 114.74, 55.45, 52.54, 52.13, 46.11, 36.33; HRMS (ESI) m/z: $[M + H]^+$ Calcd for $C_{13}H_{17}O_5S^+$ 285.0791; Found 285.0786.

According to ***General Procedure C***

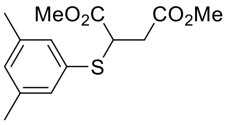

dimethyl 2-((3,5-dimethylphenyl)thio)succinate (2aj): Colourless oil (193.2 mg, 68% yield). ^{1}H NMR (400 MHz, $CDCl_3$) δ 7.09 (s, 2H), 6.95 (s, 1H), 4.00 (dd, *J* = 9.8, 5.4 Hz, 1H), 3.71 (s, 3H), 3.67 (s, 3H), 2.96 (dd, *J* = 17.0, 9.8 Hz, 1H), 2.73 (dd, *J* = 17.0, 5.4 Hz, 1H), 2.29 (s, 6H); ^{13}C NMR (101 MHz, $CDCl_3$) δ 171.75, 171.27, 138.83, 131.76, 131.15, 130.73, 52.59, 52.13, 45.76, 36.63, 21.27; HRMS (ESI) m/z: $[M + H]^+$ Calcd for $C_{14}H_{19}O_4S^+$ 283.0999; Found 283.0998.

According to ***General Procedure C***

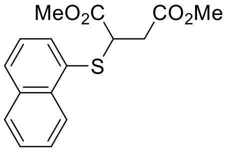

dimethyl 2-(naphthalen-1-ylthio)succinate (2ak): Pale yellow oil (250.7 mg, 82% yield). ^{1}H NMR (400 MHz, $CDCl_3$) δ 8.50 (d, *J* = 8.4 Hz, 1H), 7.92-7.84 (m, 2H), 7.80 (d, *J* = 7.1 Hz, 1H), 7.64-7.57 (m, 1H), 7.56-7.50 (m, 1H), 7.47-7.41 (m, 1H), 4.06 (dd, *J* = 9.7, 5.4 Hz, 1H), 3.65 (s, 3H), 3.54 (s, 3H), 3.00 (dd, *J* = 17.1, 9.8 Hz, 1H), 2.79 (dd, *J* = 17.1, 5.4 Hz, 1H); ^{13}C NMR (101 MHz, $CDCl_3$) δ 171.51, 171.12, 135.21, 134.99, 134.28, 130.56, 129.07, 128.78, 127.22, 126.54, 125.73, 125.60, 52.45, 52.11, 46.02, 36.49; HRMS (ESI) m/z: $[M + H]^+$ Calcd for $C_{16}H_{17}O_4S^+$ 305.0842; Found 305.0841.

According to ***General Procedure C***

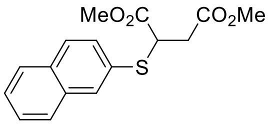

dimethyl 2-(naphthalen-2-ylthio)succinate (2al): White solid (256.0 mg, 84% yield). ^{1}H NMR (400 MHz, $CDCl_3$) δ 8.00 (s, 1H), 7.86-7.76 (m, 3H), 7.56-7.47 (m, 3H), 4.13 (dd, *J* = 9.4, 5.7 Hz, 1H), 3.70 (s, 3H), 3.68 (s, 3H), 3.01 (dd, *J* = 17.0, 9.6 Hz, 1H), 2.80 (dd, *J* = 17.0, 5.6 Hz, 1H); ^{13}C NMR (101 MHz, $CDCl_3$) δ 171.67, 171.14, 133.61, 133.11, 130.67, 129.15, 128.87, 127.84, 126.99, 126.79, 52.67, 52.15, 45.80, 36.56; HRMS (ESI) m/z: $[M + H]^+$ Calcd for $C_{16}H_{17}O_4S^+$ 305.0842; Found 305.0846.

According to ***General Procedure C***

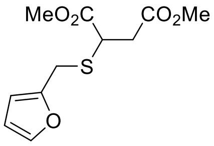

dimethyl 2-((furan-2-ylmethyl)thio)succinate (2am): Yellow oil (203.2 mg, 79% yield). ^{1}H NMR (400 MHz, $CDCl_3$) δ 7.36 (s, 1H), 6.30 (s, 1H), 6.24 (d, *J* = 2.9 Hz, 1H), 3.94 (d, *J* = 14.8 Hz, 1H), 3.81 (d, *J* = 14.8 Hz, 1H), 3.74 (s, 3H), 3.68 (dd, *J* = 10.1 Hz, 5.2 Hz, 1H), 3.66 (s, 3H), 2.97 (dd, *J* = 17.1, 10.1 Hz, 1H), 2.62 (dd, *J* = 17.1, 5.4 Hz, 1H); ^{13}C NMR (101 MHz, $CDCl_3$) δ 172.03, 171.07, 150.38, 142.57, 110.61, 108.46, 52.70, 52.14, 41.13, 36.06, 28.42; HRMS (ESI) m/z: $[M + H]^+$ Calcd for $C_{11}H_{15}O_5S^+$ 259.0635; Found 259.0629.

According to ***General Procedure C***

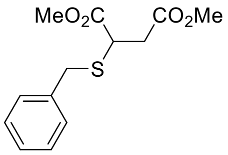

dimethyl 2-(benzylthio)succinate (2an): White solid (213.4 mg, 80% yield). ^{1}H NMR (400 MHz, $CDCl_3$) δ 7.38-7.28 (m, 4H), 7.28-7.21 (m, 1H), 3.86 (q, J = 13.4 Hz, 2H), 3.73 (s, 3H), 3.64 (s, 3H), 3.60 (dd, J = 10.0, 5.5 Hz, 1H), 2.96 (dd, J = 17.0, 10.0 Hz, 1H), 2.59 (dd, J = 17.0, 5.5 Hz, 1H); ^{13}C NMR (101 MHz, $CDCl_3$) δ 172.15, 171.05, 137.15, 129.15, 128.67, 127.45, 52.58, 52.08, 40.99, 36.15, 36.13; HRMS (ESI) m/z: $[M + H]^+$ Calcd for $C_{13}H_{17}O_4S^+$ 269.0842; Found 269.0839.

According to ***General Procedure C***

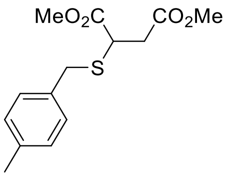

dimethyl 2-((4-methylbenzyl)thio)succinate (2ao): Colourless oil (228.1 mg, 81% yield). ^{1}H NMR (400 MHz, $CDCl_3$) δ 7.22 (d, J = 7.8 Hz, 2H), 7.12 (d, J = 7.7 Hz, 2H), 3.82 (q, J = 13.3 Hz, 2H), 3.74 (s, 3H), 3.65 (s, 3H), 3.60 (dd, J = 10.1, 5.4 Hz, 1H), 2.96 (dd, J = 17.0, 10.2 Hz, 1H), 2.60 (dd, J = 17.0, 5.3 Hz, 1H), 2.33 (s, 3H); ^{13}C NMR (101 MHz, $CDCl_3$) δ 172.23, 171.12, 137.14, 134.01, 129.37, 129.06, 52.58, 52.08, 40.96, 36.16, 35.88, 21.21; HRMS (ESI) m/z: $[M + H]^+$ Calcd for $C_{14}H_{19}O_4S^+$ 283.0999; Found 283.0995.

According to ***General Procedure C***

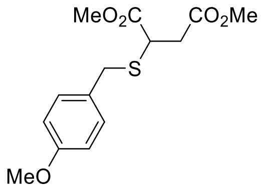

dimethyl 2-((4-methoxybenzyl)thio)succinate (2ap): Colourless oil (248.1 mg, 83% yield). ^{1}H NMR (400 MHz, $CDCl_3$) δ 7.25 (d, J = 8.3 Hz, 2H), 6.84 (d, J = 8.4 Hz, 2H), 3.87-3.76 (m, 5H), 3.74 (s, 3H), 3.64 (s, 3H), 3.58 (dd, J = 10.1, 5.4 Hz, 1H), 2.96 (dd, J = 17.0, 10.2 Hz, 1H), 2.59 (dd, J = 17.0, 5.4 Hz, 1H); ^{13}C NMR (101 MHz, $CDCl_3$) δ 172.23, 171.12, 158.99, 130.30, 129.03, 114.09, 55.38, 52.60, 52.09, 40.92, 36.16, 35.61; HRMS (ESI) m/z: $[M + H]^+$ Calcd for $C_{14}H_{19}O_5S^+$ 299.0948; Found 299.0940.

According to ***General Procedure C***

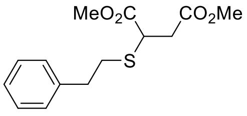

dimethyl 2-(phenethylthio)succinate (2aq): Colourless oil (197.5 mg, 70% yield). ^{1}H NMR (400 MHz, $CDCl_3$) δ 7.34-7.27 (m, 2H), 7.25-7.16 (m, 3H), 3.76 (s, 3H), 3.74-3.66 (m, 4H), 3.01 (dd, J = 17.0, 9.9 Hz, 1H), 2.97-2.82 (m, 4H), 2.67 (dd, J = 17.0, 5.6 Hz, 1H); ^{13}C NMR (101 MHz, $CDCl_3$) δ 172.24, 171.20, 140.02, 128.62, 126.61, 52.65, 52.15, 41.57, 36.37, 35.88, 33.10; HRMS (ESI) m/z: $[M + H]^+$ Calcd for $C_{14}H_{19}O_4S^+$ 283.0999; Found 283.0997.

According to ***General Procedure C***

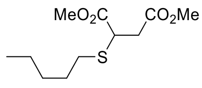

dimethyl 2-(pentylthio)succinate (2ar): Colourless oil (124.9 mg, 50% yield). ^{1}H NMR (400 MHz, $CDCl_3$) δ 3.75 (s, 3H), 3.71-3.61 (m, 4H), 2.99 (dd, J = 17.0, 9.9 Hz, 1H), 2.72-2.56 (m, 3H), 1.63-1.50 (m, 2H), 1.40-1.24 (m, 4H), 0.88 (t, J = 6.8 Hz, 3H); ^{13}C NMR (101 MHz, $CDCl_3$) δ 172.38, 171.29, 52.59, 52.12, 41.53, 36.49, 31.63, 31.05, 28.98, 22.33, 14.04; HRMS (ESI) m/z: $[M + H]^+$ Calcd for $C_{11}H_{21}O_4S^+$ 249.1155; Found 249.1148.

According to ***General Procedure C***

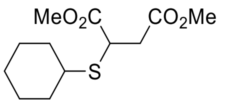

dimethyl 2-(cyclohexylthio)succinate (2as): Colourless oil (150.9 mg, 58% yield). ^{1}H NMR (400 MHz, CDCl$_3$) δ 3.76-3.67 (m, 4H), 3.65 (s, 3H), 2.95 (dd, *J* = 17.0, 9.9 Hz, 1H), 2.90-2.78 (m, 1H), 2.63 (dd, *J* = 17.1, 5.7 Hz, 1H), 2.06-1.96 (m, 1H), 1.94-1.82 (m, 1H), 1.79-1.65 (m, 2H), 1.62-1.52 (m, 1H), 1.38-1.13 (m, 5H); ^{13}C NMR (101 MHz, CDCl$_3$) δ 172.79, 171.26, 52.54, 52.04, 44.34, 40.38, 36.94, 33.70, 33.53, 26.05, 25.89, 25.70; HRMS (ESI) m/z: [M + H]$^+$ Calcd for $C_{12}H_{21}O_4S^+$ 261.1155; Found 261.1152.

According to ***General Procedure C***

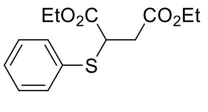

diethyl 2-(phenylthio)succinate (2au): Colourless oil (163.5 mg, 58% yield). ^{1}H NMR (400 MHz, CDCl$_3$) δ 7.56-7.42 (m, 2H), 7.37-7.28 (m, 3H), 4.19-4.08 (m, 4H), 4.00 (dd, *J* = 9.6, 5.6 Hz, 1H), 2.94 (dd, *J* = 16.9, 9.7 Hz, 1H), 2.72 (dd, *J* = 16.9, 5.6 Hz, 1H), 1.23 (t, *J* = 7.9 Hz, 3H), 1.19 (t, *J* = 7.6 Hz, 1H); ^{13}C NMR (101 MHz, CDCl$_3$) δ 171.21, 170.67, 134.23, 132.02, 129.15, 128.80, 61.56, 61.10, 45.90, 36.74, 14.25, 14.13; HRMS (ESI) m/z: [M + H]$^+$ Calcd for $C_{14}H_{19}O_4S^+$ 283.0999; Found 283.0995.

According to ***General Procedure C***

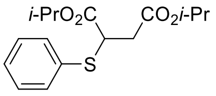

diisopropyl 2-(phenylthio)succinate (2av): Colourless oil (194.3 mg, 63% yield). ^{1}H NMR (400 MHz, CDCl$_3$) δ 7.53-7.45 (m, 2H), 7.33-7.27 (m, 3H), 5.05-4.88 (m, 2H), 3.95 (dd, *J* = 9.7, 5.6 Hz, 1H), 2.89 (dd, *J* = 16.8, 9.8 Hz, 1H), 2.68 (dd, *J* = 16.9, 5.6 Hz, 1H), 1.24-1.17 (m, 9H), 1.14 (d, *J* = 6.2 Hz, 3H); ^{13}C NMR (101 MHz, CDCl$_3$) δ 170.68, 170.11, 134.07, 132.16, 129.06, 128.62, 69.07, 68.59, 45.97, 36.95, 21.85, 21.75, 21.60; HRMS (ESI) m/z: [M + H]$^+$ Calcd for $C_{16}H_{23}O_4S^+$ 311.1312; Found 311.1310.

According to ***General Procedure C***

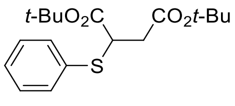

di-*tert*-butyl 2-(phenylthio)succinate (2aw): Colourless oil (173.8 mg, 51% yield). ^{1}H NMR (400 MHz, CDCl$_3$) δ 7.55-7.45 (m, 2H), 7.35-7.27 (m, 3H), 3.88 (dd, *J* = 9.7, 5.6 Hz, 1H), 2.81 (dd, *J* = 16.7, 9.9 Hz, 1H), 2.60 (dd, *J* = 16.7, 5.6 Hz, 1H), 1.43 (s, 9H), 1.37 (s, 9H); ^{13}C NMR (101 MHz, CDCl$_3$) δ 170.35, 169.93, 133.77, 132.81, 128.99, 128.33, 81.67, 81.39, 46.77, 37.82, 28.16, 27.94; HRMS (ESI) m/z: [M + Na]$^+$ Calcd for $C_{18}H_{26}NaO_4S^+$ 361.1444; Found 361.1443.

Procedure for the Synthesis of Racemic α-Thio Succinate 2at

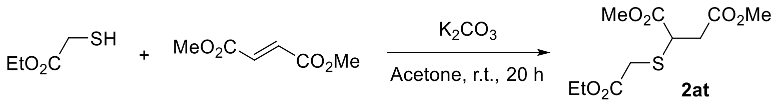

To a solution of ethyl 2-mercaptoacetate (2 mmol) and dimethyl fumarate (3 mmol) in acetone (20 mL) was added K_2CO_3 (4 mmol), and the resulting mixture was stirred at room temperature for 20 h. After filtration, the filtrate was concentrated, and the residue obtained was purified by flash

chromatography (Hexane/EtOAc: 8/1→Hexane/EtOAc: 4/1) on silica gel to give the desired product **2at**.

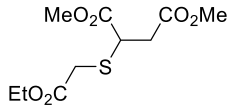

dimethyl 2-((2-ethoxy-2-oxoethyl)thio)succinate (2at): Colourless oil (519.6 mg, 98% yield). ^{1}H NMR (400 MHz, $CDCl_3$) δ 4.19 (q, J = 7.1 Hz, 2H), 3.84 (dd, J = 9.9, 5.4 Hz, 1H), 3.75 (s, 3H), 3.68 (s, 3H), 3.49 (d, J = 15.7 Hz, 1H), 3.35 (d, J = 15.7 Hz, 1H), 2.99 (dd, J = 17.1, 9.9 Hz, 1H), 2.73 (dd, J = 17.1, 5.4 Hz, 1H), 1.28 (t, J = 7.1 Hz, 3H); ^{13}C NMR (101 MHz, $CDCl_3$) δ 171.72, 170.93, 169.65, 61.80, 52.78, 52.21, 41.70, 36.06, 33.53, 14.23; HRMS (ESI) m/z: $[M + H]^+$ Calcd for $C_{10}H_{17}O_6S^+$ 265.0740; Found 265.0737.

Procedure for the Synthesis of Racemic 2ax

2ax was prepared similarly according to the literature procedure.[5] To a solution of thiophenol (3 mmol), *N*-methylmaleimide (3.3 mmol) and HBF_4 (3 mmol) in DMSO (15 mL) was added CuI (0.6 mmol), and the resulting mixture was stirred at 120 °C under air atmosphere for 18 h. Then water (45 mL) was added, and the mixture was extracted with EtOAc (15 mL × 3), and the combined organic phase was washed with brine, dried over $MgSO_4$, and concentrated. The residue was purified by flash chromatography (Hexane/EtOAc: 8/1→Hexane/EtOAc: 4/1) on silica gel to give the desired product *E*-**1ax** (171.3 mg, 26% yield) and **2ax** (159.6 mg, 24% yield).

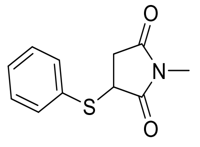

1-methyl-3-(phenylthio)pyrrolidine-2,5-dione (2ax): White solid (159.6 mg, 24% yield). ^{1}H NMR (400 MHz, $CDCl_3$) δ 7.55-7.46 (m, 2H), 7.40-7.29 (m, 3H), 4.03 (dd, J = 9.1, 3.8 Hz, 1H), 3.14 (dd, J = 18.7, 9.1 Hz, 1H), 2.89 (s, 3H), 2.70 (dd, J = 18.7, 3.8 Hz, 1H); ^{13}C NMR (101 MHz, $CDCl_3$) δ 175.72, 174.60, 134.43, 130.49, 129.49, 129.44, 44.25, 36.38, 25.22; HRMS (ESI) m/z: $[M + H]^+$ Calcd for $C_{11}H_{12}NO_2S^+$ 222.0583; Found 222.0582.

General Procedure for the Synthesis of Racemic α-Sulfonyl Succinates 2ay and 2az

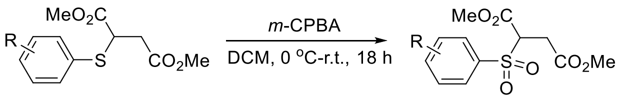

2ay (R = H)
2az (R = 4-Me)

General Procedure D: To a solution of the appropriate α-thio succinates (1 mmol) in dichloromethane (20 mL) at 0 °C was added *m*-CPBA (2.1 mmol). After addition, the resulting mixture was stirred at room temperature for 18 h. Then the reaction mixture was washed with saturated aqueous Na_2CO_3 solution, dried over $MgSO_4$, and concentrated. The residue was purified by flash chromatography (Hexane/EtOAc: 2/1→Hexane/EtOAc: 1/1) on silica gel to give the desired products.

According to ***General Procedure D***.

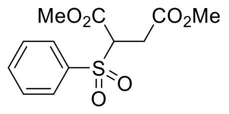

dimethyl 2-(phenylsulfonyl)succinate (2ay): Colourless oil (223.0 mg, 78% yield). ^{1}H NMR (400 MHz, $CDCl_3$) δ 7.87 (d, J = 7.9 Hz, 2H), 7.75-7.67 (m, 1H), 7.63-7.55 (m, 2H), 4.44 (dd, J = 9.6, 5.2 Hz, 1H), 3.68 (s, 3H), 3.67 (s, 3H), 3.19-3.04 (m, 2H); ^{13}C NMR (101 MHz, $CDCl_3$) δ 170.16, 165.53, 137.16, 134.72, 129.39, 129.20, 66.43, 53.40, 52.65, 31.09; HRMS (ESI) m/z: $[M + H]^+$ Calcd for $C_{12}H_{15}O_6S^+$ 287.0584; Found 287.0583.

According to ***General Procedure D***.

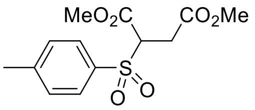

dimethyl 2-tosylsuccinate (2az): Colourless oil (274.9 mg, 92% yield). ^{1}H NMR (400 MHz, $CDCl_3$) δ 7.74 (d, *J* = 8.0 Hz, 2H), 7.37 (d, *J* = 8.0 Hz, 2H), 4.41 (dd, *J* = 9.5, 5.3 Hz, 1H), 3.69 (s, 3H), 3.68 (s, 3H), 3.17-3.02 (m, 2H), 2.46 (s, 3H); ^{13}C NMR (101 MHz, $CDCl_3$) δ 170.21, 165.68, 145.96, 134.12, 130.02, 129.25, 66.48, 53.39, 52.61, 31.27, 21.88; HRMS (ESI) m/z: $[M + H]^+$ Calcd for $C_{13}H_{17}O_6S^+$ 301.0740; Found 301.0737.

General Procedure for the Synthesis of Racemic α-Amino/Oxy/Selanyl Succinates 2ba-2bd

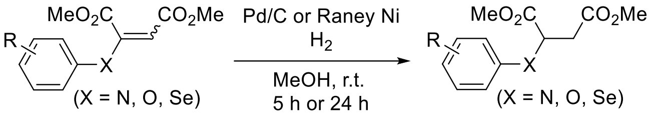

1ba-1bd **2ba-2bd**

General Procedure E: To a solution of the appropriate α-amino/oxy/selanyl fumarate and/or maleate (1 mmol) in MeOH (15 mL) was added Pd/C or Raney Ni (20 % w/w), and the mixture was stirred at room temperature for 5 h or 24 h under hydrogen. After the removal of the metal catalyst by filtration with celite, the filtrate was concentrated and the residue obtained was purified by flash chromatography (Hexane/EtOAc: 16/1→Hexane/EtOAc: 8/1) on silica gel to give the desired products.

Following ***General Procedure E***, *E*-**1ba** was hydrogenated in 5 h under the catalysis of Pd/C to give **2ba**.

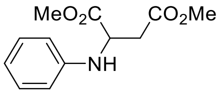

dimethyl phenylaspartate (2ba): Colourless oil (174.2 mg, 73% yield). ^{1}H NMR (400 MHz, DMSO-d_6) δ 7.14-7.04 (m, 2H), 6.65-6.56 (m, 3H), 5.99 (d, *J* = 9.2 Hz, 1H), 4.46-4.36 (m, 1H), 3.62 (s, 3H), 3.61 (s, 3H), 2.87 (dd, *J* = 16.1, 5.8 Hz, 1H), 2.76 (dd, *J* = 16.1, 7.5 Hz, 1H); ^{13}C NMR (101 MHz, DMSO-d_6) δ 172.68, 170.59, 147.17, 128.92, 116.87, 112.67, 52.32, 51.99, 51.67, 36.59; HRMS (ESI) m/z: $[M + H]^+$ Calcd for $C_{12}H_{16}NO_4^+$ 238.1074; Found 238.1072.

Following ***General Procedure E***, a mixture of *Z*-**1bb** and *E*-**1bb** (*Z*-**1bb**/*E*-**1bb** = 0.8/1) was hydrogenated in 5 h under the catalysis of Pd/C to give **2bb**.

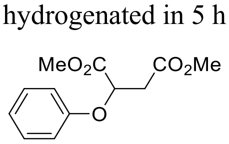

dimethyl 2-phenoxysuccinate (2bb): Colourless oil (210.5 mg, 88% yield). ^{1}H NMR (400 MHz, $CDCl_3$) δ 7.31-7.24 (m, 2H), 7.03-6.97 (m, 1H), 6.94 (d, *J* = 8.2 Hz, 2H), 5.17-5.08 (m, 1H), 3.77 (s, 3H), 3.72 (s, 3H), 3.04-2.91 (m, 2H); ^{13}C NMR (101 MHz, $CDCl_3$) δ 170.99, 170.13, 157.68, 129.69, 122.35, 115.94, 73.65, 52.75, 52.30, 37.72; HRMS (ESI) m/z: $[M + H]^+$ Calcd for $C_{12}H_{15}O_5^+$ 239.0914; Found 239.0916.

Following ***General Procedure E***, a mixture of *Z*-**1bc** and *E*-**1bc** (*Z*-**1bc**/*E*-**1bc** = 1/1) was hydrogenated in 5 h under the catalysis of Pd/C to give **2bc**.

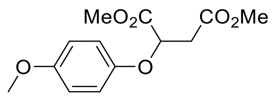

dimethyl 2-(4-methoxyphenoxy)succinate (2bc): Colourless oil (250.9 mg, 94% yield). ^{1}H NMR (400 MHz, DMSO-d_6) δ 6.92-6.80 (m, 4H), 5.05 (dd, *J* = 7.8, 4.4 Hz, 1H), 3.69 (s, 3H), 3.67 (s, 3H), 3.63 (s, 3H), 2.99 (dd, *J* = 16.3, 4.3 Hz, 1H), 2.90 (dd, *J* = 16.3, 7.8 Hz, 1H); ^{13}C NMR (101 MHz, DMSO-d_6) δ 170.31, 169.82, 154.22, 151.25, 116.81, 114.56, 73.69, 55.34, 52.25, 51.83, 37.02; HRMS (ESI) m/z: $[M + H]^+$ Calcd for $C_{13}H_{17}O_6^+$ 269.1020; Found 269.1016.

Following ***General Procedure E***, *Z*-**1bd** was hydrogenated in 24 h under the catalysis of Raney Ni to give **2bd** (Note: no reaction occurred in this case when Pd/C was employed as the metal catalyst).

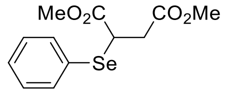

dimethyl 2-(phenylselanyl)succinate (2bd): Colourless oil (51.3 mg, 17% yield). ^{1}H NMR (400 MHz, $CDCl_3$) δ 7.60 (d, *J* = 7.3 Hz, 2H), 7.41-7.35 (m, 1H), 7.34-7.28 (m, 2H), 3.99 (dd, *J* = 9.9, 5.6 Hz, 1H), 3.67 (s, 3H), 3.66 (s, 3H), 2.99 (dd, *J* = 17.2, 9.9 Hz, 1H), 2.78 (dd, *J* = 17.2, 5.5 Hz, 1H); ^{13}C NMR (101 MHz, $CDCl_3$) δ 172.58, 171.55, 136.38, 129.30, 129.22, 126.66, 52.51, 52.14, 37.02, 36.92; HRMS (ESI) m/z: $[M + H]^+$ Calcd for $C_{12}H_{15}O_4{}^{80}Se^+$ 303.0131; Found 303.0128.

General Procedure for the Synthesis of 9aa-9aj

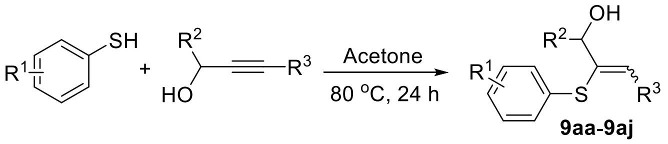

General Procedure F: To a solution of the appropriate non-terminal propargyl alcohols (4 mmol) in acetone (40 mL) were added the appropriate thiophenols (6.0 mmol), and the resulting mixture was stirred at 80 °C in an oil bath for 24 h. An aliquot of the reaction mixture was taken for ^{1}H NMR analysis to determine the ratio of *Z* and *E* isomers of the products. After removal of the solvent, the residue was purified by flash chromatography (Hexane/EtOAc: 16/1→Hexane/EtOAc: 8/1) on silica gel to give the desired products **9**. Of note, this procedure is a modified procedure based on the process reported in the literature.[6]

According to ***General Procedure F***, the reaction only gave the *Z* stereoisomer.

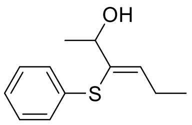

(*Z*)-3-(phenylthio)hex-3-en-2-ol (*Z*-9aa): Yellow oil (427.2 mg, 51% yield). ^{1}H NMR (400 MHz, DMSO-d_6) δ 7.34-7.24 (m, 2H), 7.24-7.18 (m, 2H), 7.18-7.10 (m, 1H), 6.39 (t, *J* = 7.1 Hz, 1H), 5.11 (d, *J* = 4.4 Hz, 1H), 4.21-4.01 (m, 1H), 2.35-2.11 (m, 2H), 1.18 (d, *J* = 6.3 Hz, 3H), 0.93 (t, *J* = 7.5 Hz, 3H); ^{13}C NMR (101 MHz, DMSO-d_6) δ 138.11, 136.11, 135.98, 129.00, 127.01, 125.28, 69.45, 23.09, 22.56, 13.49; LRMS (ESI) m/z: 209 $[M+H]^+$. The configuration of the carbon-carbon double bond of this compound was confirmed by NOESY analysis.

According to ***General Procedure F***, the reaction only gave the *Z* stereoisomer.

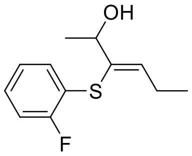

(*Z*)-3-((2-fluorophenyl)thio)hex-3-en-2-ol (*Z*-9ab): Yellow oil (416.9 mg, 46% yield). ^{1}H NMR (400 MHz, $CDCl_3$) δ 7.24-7.19 (m, 1H), 7.18-7.11 (m, 1H), 7.07-7.00 (m, 2H), 6.34 (t, J = 7.1 Hz, 1H), 4.29 (q, J = 6.3 Hz, 1H), 2.39-2.28 (m, 2H), 1.92 (s, 1H), 1.35 (d, J = 6.4 Hz, 3H), 1.01 (t, J = 7.5 Hz, 3H); ^{13}C NMR (101 MHz, $CDCl_3$) δ 160.43 (d, $J_{C\text{-}F}$ = 244.9 Hz), 140.54, 134.59, 130.08, 127.62 (d, $J_{C\text{-}F}$ = 7.6 Hz), 124.62 (d, $J_{C\text{-}F}$ = 3.6 Hz), 123.07 (d, $J_{C\text{-}F}$ = 17.1 Hz), 115.76 (d, $J_{C\text{-}F}$ = 21.8 Hz), 71.29, 23.29, 22.62, 13.53; LRMS (ESI) m/z: 227 [M+H]$^{+}$. ^{19}F NMR (376 MHz, $CDCl_3$) δ -111.19. The configuration of the carbon-carbon double bond of this compound was confirmed by NOESY analysis.

According to ***General Procedure F***, the reaction only gave the *Z* stereoisomer.

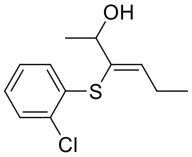

(*Z*)-3-((2-chlorophenyl)thio)hex-3-en-2-ol (*Z*-9ac): Pale yellow oil (542.9 mg, 56% yield). ^{1}H NMR (400 MHz, $CDCl_3$) δ 7.36-7.31 (m, 1H), 7.17-7.13 (m, 2H), 7.10-7.04 (m, 1H), 6.48-6.42 (m, 1H), 4.36-4.28 (m, 1H), 2.35-2.25 (m, 2H), 1.86 (s, 1H), 1.37 (d, J = 6.4 Hz, 3H), 1.00 (t, J = 7.5 Hz, 3H); ^{13}C NMR (101 MHz, $CDCl_3$) δ 142.08, 135.42, 134.73, 132.19, 129.87, 127.67, 127.17, 126.34, 71.65, 23.35, 22.83, 13.49; LRMS (ESI) m/z: 245 ([M+H]$^{+}$, (Cl^{37})), 243 ([M+H]$^{+}$, (Cl^{35})). The configuration of the carbon-carbon double bond of this compound was confirmed by NOESY analysis.

According to ***General Procedure F***, the reaction only gave the *Z* stereoisomer.

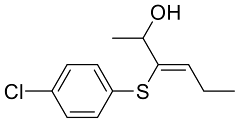

(*Z*)-3-((4-chlorophenyl)thio)hex-3-en-2-ol (*Z*-9ad): Pale yellow oil (483.9 mg, 50% yield). ^{1}H NMR (400 MHz, $CDCl_3$) δ 7.24-7.12 (m, 4H), 6.33 (t, J = 7.1 Hz, 1H), 4.37-4.20 (m, 1H), 2.37-2.19 (m, 2H), 2.03 (s, 1H), 1.33 (d, J = 6.3 Hz, 3H), 0.99 (t, J = 7.6 Hz, 3H); ^{13}C NMR (101 MHz, $CDCl_3$) δ 140.80, 135.62, 134.83, 131.57, 129.16, 129.02, 71.45, 23.32, 22.75, 13.56; LRMS (ESI) m/z: 245 ([M+H]$^{+}$, (Cl^{37})), 243 ([M+H]$^{+}$, (Cl^{35})). The configuration of the carbon-carbon double bond of this compound was confirmed by NOESY analysis.

According to ***General Procedure F***, the reaction only gave the *Z* stereoisomer.

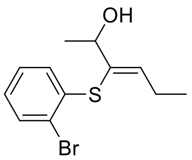

(*Z*)-3-((2-bromophenyl)thio)hex-3-en-2-ol (*Z*-9ae): Pale yellow oil (873.4 mg, 76% yield). ^{1}H NMR (400 MHz, $CDCl_3$) δ 7.51 (d, J = 7.9 Hz, 1H), 7.23-7.17 (m, 1H), 7.16-7.10 (m, 1H), 7.03-6.94 (m, 1H), 6.46 (t, J = 7.1 Hz, 1H), 4.39-4.27 (m, 1H), 2.36-2.22 (m, 2H), 1.94 (d, J = 5.5 Hz, 1H), 1.37 (d, J = 6.4 Hz, 3H), 1.00 (t, J = 7.5 Hz, 3H); ^{13}C NMR (101 MHz, $CDCl_3$) δ 142.15, 137.44, 135.22, 133.14, 127.78, 127.55, 126.51, 121.97, 71.66, 23.39, 22.87, 13.50; LRMS (ESI) m/z: 289 ([M+H]$^{+}$, (Br^{81})), 287 ([M+H]$^{+}$, (Br^{79})). The configuration of the carbon-carbon double bond of this compound was confirmed by NOESY analysis.

According to ***General Procedure F***, the reaction only gave the *Z* stereoisomer.

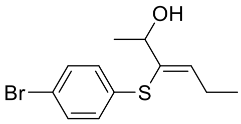

(*Z*)-3-((4-bromophenyl)thio)hex-3-en-2-ol (*Z*-9af): Yellow oil (514.6 mg, 45% yield). ^{1}H NMR (400 MHz, $CDCl_3$) δ 7.38-7.32 (m, 2H), 7.15-7.07 (m, 2H), 6.34 (t, *J* = 7.1 Hz, 1H), 4.35-4.25 (m, 1H), 2.35-2.24 (m, 2H), 1.90 (d, *J* = 5.1 Hz, 1H), 1.33 (d, *J* = 6.4 Hz, 3H), 1.00 (t, *J* = 7.5 Hz, 3H); ^{13}C NMR (101 MHz, $CDCl_3$) δ 141.05, 135.59, 135.47, 132.09, 129.26, 119.38, 71.54, 23.36, 22.78, 13.57; LRMS (ESI) m/z: 289 ($[M+H]^+$, (Br^{81})), 287 ($[M+H]^+$, (Br^{79})). The configuration of the carbon-carbon double bond of this compound was confirmed by NOESY analysis.

According to ***General Procedure F*** but with 3 equivalents of thiophenol, the reaction gave two stereoisomers (*Z*-**9ag**/*E*-**9ag** = 4.3/1, 741.8 mg, 72% combined yield), which were separable by chromatography.

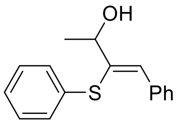

(*Z*)-4-phenyl-3-(phenylthio)but-3-en-2-ol (*Z*-9ag): Pale yellow oil. ^{1}H NMR (400 MHz, DMSO-d_6) δ 7.59 (d, *J* = 7.5 Hz, 2H), 7.38 (s, 1H), 7.36-7.21 (m, 7H), 7.21-7.15 (m, 1H), 5.39 (d, *J* = 4.4 Hz, 1H), 4.27-4.08 (m, 1H), 1.29 (d, *J* = 6.3 Hz, 3H); ^{13}C NMR (101 MHz, DMSO-d_6) δ 137.94, 135.70, 134.74, 132.38, 129.19, 129.05, 128.09, 128.05, 127.66, 125.99, 69.54, 23.51; LRMS (ESI) m/z: 257 $[M+H]^+$. The configuration of the carbon-carbon double bond of this compound was confirmed by NOESY analysis.

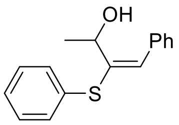

(*E*)-4-phenyl-3-(phenylthio)but-3-en-2-ol (*E*-9ag): Pale yellow oil. ^{1}H NMR (400 MHz, DMSO-d_6) δ 7.50 (d, *J* = 7.6 Hz, 2H), 7.43-7.37 (m, 2H), 7.37-7.28 (m, 3H), 7.27-7.22 (m, 1H), 7.20 (d, *J* = 7.6 Hz, 2H), 6.25 (s, 1H), 5.28 (d, *J* = 3.8 Hz, 1H), 4.97-4.84 (m, 1H), 1.39 (d, *J* = 6.3 Hz, 3H); ^{13}C NMR (101 MHz, DMSO-d_6) δ 145.64, 136.06, 135.00, 132.12, 129.43, 128.94, 128.37, 128.31, 127.40, 127.02, 64.24, 22.48; LRMS (ESI) m/z: 257 $[M+H]^+$. The configuration of the carbon-carbon double bond of this compound was confirmed by NOESY analysis.

According to ***General Procedure F*** but with 3 equivalents of 4-chlorothiophenol, the reaction gave two stereoisomers (*Z*-**9ah**/*E*-**9ah** = 3.5/1, 605.5 mg, 52% combined yield), which were separable by chromatography.

(*Z*)-3-((4-chlorophenyl)thio)-4-phenylbut-3-en-2-ol (*Z*-9ah): Pale yellow oil. ^{1}H NMR (400 MHz, $CDCl_3$) δ 7.60 (d, *J* = 7.3 Hz, 2H), 7.36-7.25 (m, 4H), 7.25-7.16 (m, 4H), 4.47-4.33 (m, 1H), 1.99 (d, *J* = 5.1 Hz, 1H), 1.45 (d, *J* = 6.3 Hz, 3H); ^{13}C NMR (101 MHz, $CDCl_3$) δ 137.15, 135.52, 134.59, 133.66, 132.34, 130.14, 129.54, 129.33, 128.33, 128.29, 71.75, 23.28; LRMS (ESI) m/z: 293 ($[M+H]^+$, (Cl^{37})), 291 ($[M+H]^+$, (Cl^{35})). The configuration of the carbon-carbon double bond of this compound was confirmed by NOESY analysis.

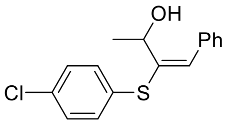

(*E*)-3-((4-chlorophenyl)thio)-4-phenylbut-3-en-2-ol (*E*-9ah): Colourless oil. ^{1}H NMR (400 MHz, $CDCl_3$) δ 7.45 (d, *J* = 8.3 Hz, 2H), 7.39-7.27 (m, 5H), 7.21 (d, *J* = 7.5 Hz, 2H), 6.61 (s, 1H), 5.13-5.01 (m, 1H), 1.98 (d, *J* = 7.1 Hz, 1H), 1.46 (d, *J* = 6.4 Hz, 3H); ^{13}C NMR (101 MHz, $CDCl_3$) δ 142.50, 135.99, 134.34, 133.90, 133.55, 132.95, 129.59, 128.62, 127.75, 66.52, 23.03; LRMS (ESI) m/z: 293 ($[M+H]^+$, (Cl^{37})), 291 ($[M+H]^+$, (Cl^{35})). The configuration of the carbon-carbon double bond of this compound was confirmed by NOESY analysis.

According to ***General Procedure F***, the reaction gave two stereoisomers (*Z*-**9ai**/*E*-**9ai** = 11/1, 207.4 mg, 20% combined yield), which were inseparable by chromatography.

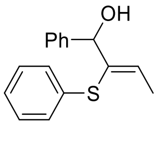

(*Z*)-1-phenyl-2-(phenylthio)but-2-en-1-ol (*Z*-9ai): Pale yellow oil. ^{1}H NMR (400 MHz, $CDCl_3$) δ 7.38-7.28 (m, 5H), 7.26-7.20 (m, 4H), 7.19-7.12 (m, 1H), 6.44 (q, *J* = 6.7 Hz, 1H), 5.24 (d, *J* = 4.9 Hz, 1H), 2.47 (d, *J* = 5.1 Hz, 1H), 1.89 (d, *J* = 6.7 Hz, 3H); ^{13}C NMR (101 MHz, $CDCl_3$) δ 141.71, 136.37, 135.51, 134.83, 129.10, 128.43, 128.24, 127.91, 126.79, 125.94, 71.44, 15.75; LRMS (ESI) m/z: 257 $[M+H]^+$. The configuration of the carbon-carbon double bond of this compound was confirmed by NOESY analysis.

According to ***General Procedure F***, the reaction gave two stereoisomers (*Z*-**9aj**/*E*-**9aj** = 11/1, 438.7 mg, 56% combined yield), which were inseparable by chromatography.

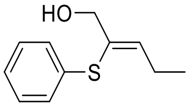

(*Z*)-2-(phenylthio)pent-2-en-1-ol (*Z*-9aj): Yellow oil. ^{1}H NMR (400 MHz, $CDCl_3$) δ 7.31-7.22 (m, 4H), 7.20-7.13 (m, 1H), 6.24 (t, *J* = 7.2 Hz, 1H), 4.06 (d, *J* = 6.3 Hz, 2H), 2.43-2.31 (m, 2H), 1.87 (t, *J* = 6.4 Hz, 1H), 1.02 (t, *J* = 7.5 Hz, 3H); ^{13}C NMR (101 MHz, $CDCl_3$) δ 140.43, 134.71, 131.84, 129.16, 129.14, 126.35, 66.04, 23.14, 13.79; LRMS (ESI) m/z: 195 $[M+H]^+$.

General Procedure for the Synthesis of 5aa-5aj

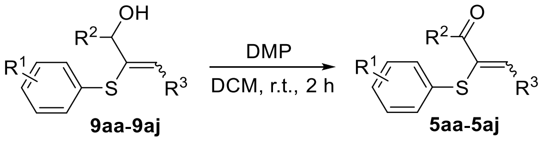

General Procedure G: To a solution of the appropriate α-thio allyl alcohols **9** (2 mmol) in dichloromethane (20 mL) was added Dess–Martin periodinane (2.4 mmol), and the resulting mixture was stirred at room temperature for 2 h. After that, the reaction mixture was washed with saturated aqueous $NaHCO_3$ solution, dried over $MgSO_4$, and concentrated. The residue was purified by flash chromatography (Hexane/EtOAc: 32/1→Hexane/EtOAc: 16/1) on silica gel to give the desired products **5**.

According to ***General Procedure G***.

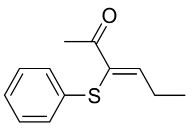

(*Z*)-3-(phenylthio)hex-3-en-2-one (*Z*-5aa): Yellow oil (331.8 mg, 80% yield). ^{1}H NMR (500 MHz, $CDCl_3$) δ 7.28-7.22 (m, 3H), 7.19-7.11 (m, 3H), 2.57-2.47 (m, 2H), 2.28 (s, 3H), 1.10 (t, J = 7.5 Hz, 3H); ^{13}C NMR (126 MHz, $CDCl_3$) δ 197.91, 153.46, 135.93, 133.91, 129.32, 127.58, 126.09, 27.60, 24.32, 13.02; LRMS (ESI) m/z: 207 $[M+H]^+$. The configuration of the carbon-carbon double bond of this compound was confirmed by NOESY analysis. The characterization data of this compound were also in accordance with the published ones.[7]

According to ***General Procedure G***.

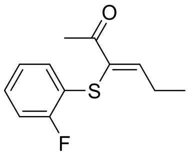

(*Z*)-3-((2-fluorophenyl)thio)hex-3-en-2-one (*Z*-5ab): Pale yellow oil (363.6 mg, 81% yield). ^{1}H NMR (400 MHz, $CDCl_3$) δ 7.27 (t, J = 7.3 Hz, 1H), 7.20-7.12 (m, 1H), 7.10-7.00 (m, 3H), 2.60-2.49 (m, 2H), 2.32 (s, 3H), 1.11 (t, J = 7.6 Hz, 3H); ^{13}C NMR (101 MHz, $CDCl_3$) δ 197.14, 160.28 (d, J_{C-F} = 245.6 Hz), 154.27, 132.90, 129.91 (d, J_{C-F} = 1.8 Hz), 128.02 (d, J_{C-F} = 7.6 Hz), 124.82 (d, J_{C-F} = 3.9 Hz), 122.97 (d, J_{C-F} = 17.0 Hz), 115.92 (d, J_{C-F} = 21.6 Hz), 27.32, 24.45, 12.92; ^{19}F NMR (376 MHz, $CDCl_3$) δ -111.21; LRMS (ESI) m/z: 225 $[M+H]^+$. The configuration of the carbon-carbon double bond of this compound was confirmed by NOESY analysis.

According to ***General Procedure G***.

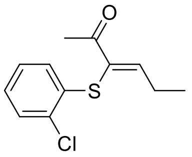

(*Z*)-3-((2-chlorophenyl)thio)hex-3-en-2-one (*Z*-5ac): Pale yellow oil (375.1 mg, 78% yield). ^{1}H NMR (400 MHz, $CDCl_3$) δ 7.40-7.34 (m, 2H), 7.16-7.06 (m, 2H), 6.94-6.89 (m, 1H), 2.56-2.45 (m, 2H), 2.31 (s, 3H), 1.10 (t, J = 7.6 Hz, 3H); ^{13}C NMR (101 MHz, $CDCl_3$) δ 197.51, 155.36, 135.33, 132.65, 132.17, 130.04, 127.49, 127.48, 126.79, 27.38, 24.45, 12.96; LRMS (ESI) m/z: 243 ($[M+H]^+$, (Cl^{37})), 241 ($[M+H]^+$, (Cl^{35})).

According to ***General Procedure G***.

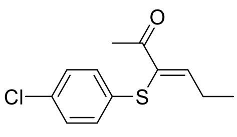

(*Z*)-3-((4-chlorophenyl)thio)hex-3-en-2-one (*Z*-5ad): Yellow oil (360.7 mg, 75% yield). ^{1}H NMR (400 MHz, $CDCl_3$) δ 7.27 (t, J = 7.4 Hz, 1H), 7.24-7.20 (m, 2H), 7.12-7.06 (m, 2H), 2.58-2.46 (m, 2H), 2.30 (s, 3H), 1.11 (t, J = 7.6 Hz, 3H); ^{13}C NMR (101 MHz, $CDCl_3$) δ 197.31, 154.19, 134.51, 133.85, 132.12, 129.45, 128.96, 27.50, 24.47, 13.04; LRMS (ESI) m/z: 243 ($[M+H]^+$, (Cl^{37})), 241 ($[M+H]^+$, (Cl^{35})).

According to ***General Procedure G***.

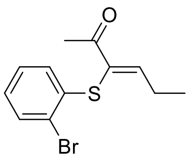

(*Z*)-3-((2-bromophenyl)thio)hex-3-en-2-one (*Z*-5ae): White solid (484.1 mg, 85% yield). ^{1}H NMR (400 MHz, $CDCl_3$) δ 7.53 (d, J = 7.9 Hz, 1H), 7.39 (t, J = 7.4 Hz, 1H), 7.21-7.15 (m, 1H), 7.04-6.98 (m, 1H), 6.88 (dd, J = 7.9, 1.1 Hz, 1H), 2.56-2.45 (m, 2H), 2.31 (s, 3H), 1.10 (t, J = 7.5 Hz, 3H); ^{13}C NMR (101 MHz, $CDCl_3$) δ 197.57, 155.35, 137.37, 133.31, 133.02, 128.11, 127.30, 126.93, 121.82, 27.42, 24.46, 12.97; LRMS (ESI) m/z: 287 ($[M+H]^+$, (Br^{81})), 285 ($[M+H]^+$, (Br^{79})).

According to ***General Procedure G***.

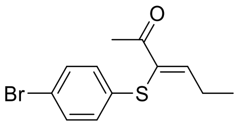

(*Z*)-3-((4-bromophenyl)thio)hex-3-en-2-one (*Z*-5af): Yellow oil (478.9 mg, 84% yield). ^{1}H NMR (400 MHz, $CDCl_3$) δ 7.40-7.34 (m, 2H), 7.31-7.26 (m, 1H), 7.05-6.99 (m, 2H), 2.58-2.46 (m, 2H), 2.30 (s, 3H), 1.11 (t, *J* = 7.6 Hz, 3H); ^{13}C NMR (101 MHz, $CDCl_3$) δ 197.26, 154.35, 135.23, 133.68, 132.34, 129.16, 119.92, 27.49, 24.48, 13.03; LRMS (ESI) m/z: 287 ($[M+H]^+$, (Br^{81})), 285 ($[M+H]^+$, (Br^{79})).

According to ***General Procedure G***.

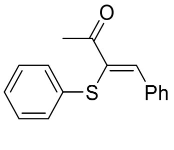

(*Z*)-4-phenyl-3-(phenylthio)but-3-en-2-one (*Z*-5ag): Yellow oil (445.0 mg, 87% yield). ^{1}H NMR (400 MHz, $CDCl_3$) δ 7.95 (s, 1H), 7.89-7.81 (m, 2H), 7.45-7.36 (m, 3H), 7.31-7.21 (m, 4H), 7.21-7.15 (m, 1H), 2.35 (s, 3H); ^{13}C NMR (101 MHz, $CDCl_3$) δ 199.15, 144.41, 135.47, 134.40, 132.50, 131.03, 130.26, 129.53, 128.53, 127.94, 126.54, 27.82; LRMS (ESI) m/z: 255 $[M+H]^+$. The configuration of the carbon-carbon double bond of this compound was confirmed by NOESY analysis.

According to ***General Procedure G***.

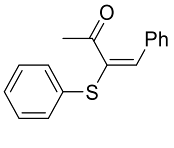

(*E*)-4-phenyl-3-(phenylthio)but-3-en-2-one (*E*-5ag): Yellow solid (363.7 mg, 72% yield). ^{1}H NMR (400 MHz, $CDCl_3$) δ 7.43 (d, *J* = 7.7 Hz, 2H), 7.37-7.23 (m, 8H), 7.08 (s, 1H), 2.16 (s, 3H); ^{13}C NMR (101 MHz, $CDCl_3$) δ 201.63, 136.84, 136.45, 135.06, 132.94, 131.43, 129.43, 129.02, 128.83, 128.76, 127.96, 30.48; LRMS (ESI) m/z: 255 $[M+H]^+$. The configuration of the carbon-carbon double bond of this compound was confirmed by NOESY analysis.

According to ***General Procedure G***.

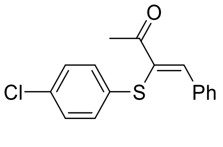

(*Z*)-3-((4-chlorophenyl)thio)-4-phenylbut-3-en-2-one (*Z*-5ah): Yellow solid (444.1 mg, 77% yield). ^{1}H NMR (400 MHz, $CDCl_3$) δ 7.96 (s, 1H), 7.86-7.78 (m, 2H), 7.45-7.36 (m, 3H), 7.24 (d, *J* = 8.5 Hz, 2H), 7.15 (d, *J* = 8.4 Hz, 2H), 2.36 (s, 3H); ^{13}C NMR (101 MHz, $CDCl_3$) δ 198.53, 145.14, 134.18, 134.02, 132.58, 132.16, 131.04, 130.50, 129.66, 129.23, 128.62, 27.74; LRMS (ESI) m/z: 291 ($[M+H]^+$, (Cl^{37})), 289 ($[M+H]^+$, (Cl^{35})). The configuration of the carbon-carbon double bond of this compound was confirmed by NOESY analysis.

According to ***General Procedure G***.

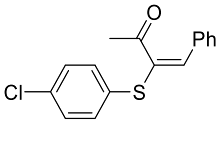

(*E*)-3-((4-chlorophenyl)thio)-4-phenylbut-3-en-2-one (*E*-5ah): White solid (416.2 mg, 72% yield). ^{1}H NMR (400 MHz, $CDCl_3$) δ 7.39-7.24 (m, 9H), 7.10 (s, 1H), 2.16 (s, 3H); ^{13}C NMR (101 MHz, $CDCl_3$) δ 201.32, 137.24, 136.10, 134.84, 134.12, 132.54, 131.61, 129.62, 129.25, 128.87, 128.84, 30.42; LRMS (ESI) m/z: 291 ($[M+H]^+$, (Cl^{37})), 289 ($[M+H]^+$, (Cl^{35})). The configuration of the carbon-carbon double bond of this compound was confirmed by NOESY analysis.

According to ***General Procedure G***.

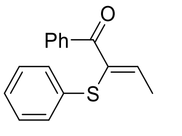

(*Z*)-1-phenyl-2-(phenylthio)but-2-en-1-one (*Z*-5ai): Pale yellow solid (326.6 mg, 64% yield). ^{1}H NMR (400 MHz, $CDCl_3$) δ 7.70 (d, *J* = 7.7 Hz, 2H), 7.54-7.46 (m, 1H), 7.42-7.34 (m, 2H), 7.26-7.21 (m, 2H), 7.21-7.15 (m, 2H), 7.15-7.09 (m, 1H), 6.84 (q, *J* = 6.9 Hz, 1H), 2.14 (d, *J* = 6.9 Hz, 3H); ^{13}C NMR (101 MHz, $CDCl_3$) δ 194.17, 144.70, 137.53, 136.99, 134.42, 132.44, 129.91, 129.49, 129.07, 128.25, 126.73, 16.60; LRMS (ESI) m/z: 255 $[M+H]^+$.

According to ***General Procedure G***.

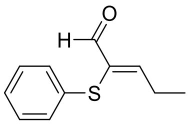

(*Z*)-2-(phenylthio)pent-2-enal (*Z*-5aj): Yellow oil (336.9 mg, 88% yield). ^{1}H NMR (400 MHz, DMSO-d_6) δ 9.53 (s, 1H), 7.58 (t, *J* = 7.3 Hz, 1H), 7.32-7.22 (m, 2H), 7.21-7.10 (m, 3H), 2.63-2.53 (m, 2H), 1.08 (t, *J* = 7.5 Hz, 3H); ^{13}C NMR (101 MHz, DMSO-d_6) δ 190.87, 165.98, 134.59, 134.47, 129.08, 127.56, 125.92, 24.11, 12.41; LRMS (ESI) m/z: 193 $[M+H]^+$. The configuration of the carbon-carbon double bond of this compound was confirmed by NOESY analysis.

Procedure for the Synthesis of 5ak

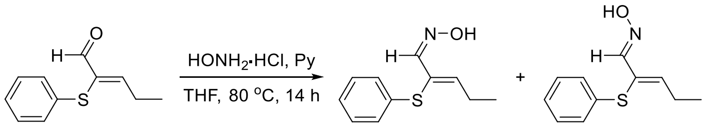

To a solution of *Z*-**5aj** (1 mmol) in THF (10 mL) were added hydroxylamine hydrochloride (2 mmol) and pyridine (4 mmol), and the resulting mixture was stirred at 80 °C in an oil bath for 14 h. After removal of the solvent, water (20 mL) was added to the residue obtained, and the resulting mixture was extracted with EtOAc (15 mL × 3). Then the combined organic phase was washed with 1 mol/L HCl, saturated aqueous $NaHCO_3$ solution, and brine, and dried over $MgSO_4$. An aliquot of the organic phase was taken for ^{1}H NMR analysis to determine the ratio of *Z* and *E* isomers of the products (the *Z* and *E* isomerization herein refers to the isomerization on the C=N bond). After removal of the solvent, the residue obtained was purified by flash chromatography (Hexane/EtOAc: 16/1→Hexane/EtOAc: 8/1) on silica gel to give the desired product **5ak**. The reaction gave two stereoisomers (*1Z,2Z*-**5ak**/*1E,2Z*-**5ak** = 0.4/1, 105.6 mg, 51% combined yield), which were inseparable by chromatography.

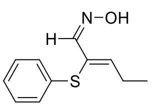

(1*Z*,2*Z*)-**5ak**
+

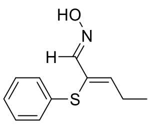

(1*E*,2*Z*)-**5ak**
(1*Z*,2*Z*)-**5ak**/(1*E*,2*Z*)-**5ak** = 0.4/1

Pale yellow oil. ^{1}H NMR (400 MHz, $CDCl_3$) δ 8.94 (s, 0.39H), 8.76 (s, 1.00H), 8.16 (s, 0.40H), 7.83 (s, 1.01H), 7.33-7.15 (m, 6.30H), 7.15-7.08 (m, 1.02H), 6.53 (t, *J* = 7.3 Hz, 1.02H), 6.14 (t, *J* = 7.9 Hz, 0.39H), 2.57-2.44 (m, 2.07H), 2.40-2.28 (m, 0.83H), 1.04 (t, *J* = 7.5 Hz, 4.29H); ^{13}C NMR (101 MHz, $CDCl_3$) δ 151.39, 150.99, 146.28, 145.81, 135.44, 134.49, 130.28, 129.16, 129.05, 127.55, 126.90, 126.45, 126.04, 125.77, 23.83, 22.73, 13.99, 13.30; LRMS (ESI) m/z: 208 $[M+H]^+$. The configuration of the carbon-nitrogen double bond was confirmed by NOESY analysis of (1*E*,2*Z*)-2-(phenylthio)pent-2-enal oxime.

Procedure for the Synthesis of 5al

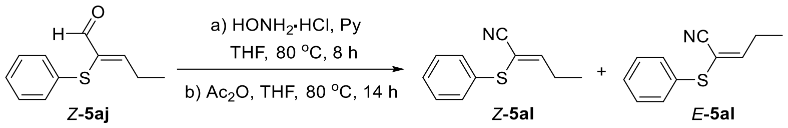

To a solution of *Z*-**5aj** (1 mmol) in THF (10 mL) were added hydroxylamine hydrochloride (2 mmol) and pyridine (4 mmol), and the mixture was stirred at 80 °C in an oil bath for 8 h. Then Ac_2O (5 mmol) was added, and the resulting mixture was stirred at 80 °C for 14 h. After removal of the solvent, water (20 mL) was added to the residue obtained, and the resulting mixture was extracted with EtOAc (15 mL × 3). Then the combined organic phase was washed with 1 mol/L HCl, saturated aqueous $NaHCO_3$ solution, and brine, and dried over $MgSO_4$. An aliquot of the organic phase was taken for ^{1}H NMR analysis to determine the ratio of *Z* and *E* isomers of the products. After removal of the solvent, the residue obtained was purified by flash chromatography (Hexane/EtOAc: 32/1→Hexane/EtOAc: 16/1) on silica gel to give the desired product **5al**. Although *Z*-**5aj** was used as the substrate, the reaction gave two stereoisomers (*Z*-**5al**/*E*-**5al** = 1/1, 100.9 mg, 53% combined yield), which were separable by chromatography.

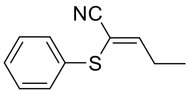

(*Z*)-2-(phenylthio)pent-2-enenitrile (*Z*-5al): Yellow oil. ^{1}H NMR (400 MHz, $CDCl_3$) δ 7.46-7.30 (m, 5H), 6.79 (t, *J* = 7.2 Hz, 1H), 2.56-2.46 (m, 2H), 1.12 (t, *J* = 7.6 Hz, 3H); ^{13}C NMR (101 MHz, $CDCl_3$) δ 155.08, 131.73, 131.48, 129.59, 128.59, 116.79, 109.19, 23.89, 12.77; LRMS (ESI) m/z: 190 $[M+H]^+$.

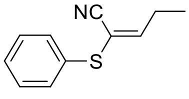

(*E*)-2-(phenylthio)pent-2-enenitrile (*E*-5al): Yellow oil. ^{1}H NMR (400 MHz, $CDCl_3$) δ 7.45-7.29 (m, 5H), 6.77 (t, *J* = 7.7 Hz, 1H), 2.57-2.44 (m, 2H), 1.14 (t, *J* = 7.5 Hz, 3H); ^{13}C NMR (101 MHz, $CDCl_3$) δ 157.04, 132.05, 131.21, 129.58, 128.46, 115.08, 107.65, 26.29, 13.03; LRMS (ESI) m/z: 190 $[M+H]^+$.

Procedure for the Synthesis of *Z*-5am

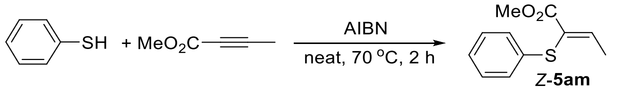

According to a procedure reported in the literature.[8] A mixture of thiophenol (3 mmol), methyl 2-butynoate (3 mmol) and AIBN (0.6 mmol) was stirred at 70 °C in an oil bath for 2 h under solvent-free conditions. Then water (20 mL) was added, and the resulting mixture was extracted with EtOAc (15 mL × 3). The combined organic phase was washed with brine and dried over $MgSO_4$. After removal of the solvent, the residue obtained was purified by flash chromatography (Hexane/EtOAc: 16/1→Hexane/EtOAc: 8/1) on silica gel to give the desired product *Z*-**5am**. Of note, *E*-**5am** was not observed in this case, however, both *Z* and *E* isomers of the Michael addition product, together with phenyl disulfide, were obtained as the side products.

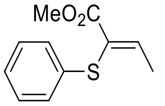

methyl (*Z*)-2-(phenylthio)but-2-enoate (*Z*-5am): Pale yellow oil. (143.3 mg, 23% yield). ^{1}H NMR (400 MHz, $CDCl_3$) δ 7.55 (q, *J* = 7.0 Hz, 1H), 7.28-7.18 (m, 4H), 7.18-7.11 (m, 1H), 3.68 (s, 3H), 2.10 (d, *J* = 7.0 Hz, 3H); ^{13}C NMR (101 MHz, $CDCl_3$) δ 166.07, 149.64, 135.82, 129.08, 127.91, 127.31, 126.06, 52.73, 16.96; LRMS (ESI) m/z: 209 $[M+H]^+$. The characterization data of this compound were in accordance with the published ones.[9]

General Procedure for the Synthesis of Racemic 7aa-7af

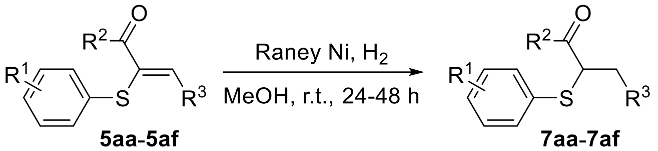

General Procedure H: To a solution of the appropriate α-thio enones **5** (1 mmol) in MeOH (15 mL) was added Raney Ni (20 % w/w), and the mixture was stirred at room temperature for 24 h under hydrogen and monitored by TLC [If the reaction did not complete in 24 h in some cases, another portion of Raney Ni (20 % w/w) was added and the reaction mixture was allowed to stir under hydrogen for another 24 h and monitored by TLC]. After removal of the Raney Ni by filtration with celite, the filtrate was concentrated and the residue obtained was purified by flash chromatography (Hexane/EtOAc: 32/1→Hexane/EtOAc: 16/1) on silica gel to give the desired products **7**. Of note, the high loading of the Raney Ni catalyst is essential because the sulfur atoms in the substrates and products were able to poison the Raney Ni catalyst by coordinating with the Ni. In addition, TLC monitoring is important for this reaction because the C–S bond in the products could also be cleaved under the standard conditions. By contrast, only less than 15% conversions were observed for this process in most cases when Pd/C was used as the catalyst.

According to ***General Procedure H***.

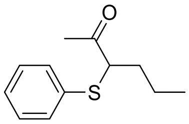

3-(phenylthio)hexan-2-one (7aa): Colourless oil (93.7 mg, 45% yield). ^{1}H NMR (400 MHz, $CDCl_3$) δ 7.32-7.26 (m, 2H), 7.25-7.17 (m, 3H), 3.56 (t, J = 7.5 Hz, 1H), 2.17 (s, 3H), 1.79-1.57 (m, 2H), 1.49-1.30 (m, 2H), 0.87 (t, J = 7.3 Hz, 3H); ^{13}C NMR (101 MHz, $CDCl_3$) δ 205.70, 133.33, 132.38, 129.22, 127.95, 57.68, 32.57, 26.48, 20.67, 13.89; HRMS (ESI) m/z: [M + H]$^+$ Calcd for $C_{12}H_{17}OS^+$ 209.0995; Found 209.1001.

According to ***General Procedure H***.

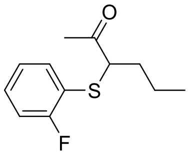

3-((2-fluorophenyl)thio)hexan-2-one (7ab): Colourless oil (84.2 mg, 37% yield). ^{1}H NMR (400 MHz, $CDCl_3$) δ 7.43-7.37 (m, 1H), 7.33-7.26 (m, 1H), 7.11-7.04 (m, 2H), 3.64 (t, J = 7.5 Hz, 1H), 2.26 (s, 3H), 1.85-1.64 (m, 2H), 1.57-1.34 (m, 2H), 0.94 (t, J = 7.3 Hz, 3H); ^{13}C NMR (101 MHz, $CDCl_3$) δ 205.31, 162.66 (d, J_{C-F} = 246.8 Hz), 135.64 (d, J_{C-F} = 1.0 Hz), 130.69 (d, J_{C-F} = 8.0 Hz), 124.73 (d, J_{C-F} = 3.9 Hz), 119.81 (d, J_{C-F} = 18.2 Hz), 116.16 (d, J_{C-F} = 23.0 Hz), 56.99 (d, J_{C-F} = 1.6 Hz), 32.56, 26.31 (d, J_{C-F} = 1.2 Hz), 20.62, 13.87; ^{19}F NMR (376 MHz, $CDCl_3$) δ -106.77. HRMS (ESI) m/z: [M + H]$^+$ Calcd for $C_{12}H_{16}FOS^+$ 227.0900; Found 227.0901.

According to ***General Procedure H***.

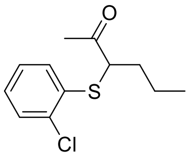

3-((2-chlorophenyl)thio)hexan-2-one (7ac): Colourless oil (118.3 mg, 49% yield). ^{1}H NMR (400 MHz, $CDCl_3$) δ 7.41-7.35 (m, 2H), 7.23-7.15 (m, 2H), 3.76 (t, J = 7.5 Hz, 1H), 2.23 (s, 3H), 1.93-1.72 (m, 2H), 1.58-1.40 (m, 2H), 0.96 (t, J = 7.3 Hz, 3H); ^{13}C NMR (101 MHz, $CDCl_3$) δ 206.06, 135.59, 133.25, 132.22, 130.18, 128.51, 127.50, 56.61, 32.81, 25.77, 20.75, 13.89; HRMS (ESI) m/z: [M + H]$^+$ Calcd for $C_{12}H_{16}{}^{35}ClOS^+$ 243.0605; Found 243.0600.

According to ***General Procedure H***.

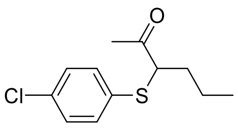

3-((4-chlorophenyl)thio)hexan-2-one (7ad): Colourless oil (101.0 mg, 42% yield). ^{1}H NMR (400 MHz, $CDCl_3$) δ 7.30-7.24 (m, 4H), 3.59 (t, J = 7.5 Hz, 1H), 2.25 (s, 3H), 1.84-1.60 (m, 2H), 1.55-1.33 (m, 2H), 0.94 (t, J = 7.3 Hz, 3H); ^{13}C NMR (101 MHz, $CDCl_3$) δ 205.28, 134.33, 133.86, 131.54, 129.41, 57.66, 32.39, 26.44, 20.64, 13.89; HRMS (ESI) m/z: $[M + H]^+$ Calcd for $C_{12}H_{16}{}^{35}ClOS^+$ 243.0605; Found 243.0598.

According to ***General Procedure H***.

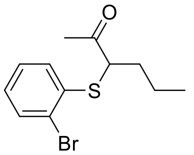

3-((2-bromophenyl)thio)hexan-2-one (7ae): Colourless oil (137.8 mg, 48% yield). ^{1}H NMR (400 MHz, $CDCl_3$) δ 7.57 (d, J = 8.0 Hz, 1H), 7.35 (d, J = 7.9 Hz, 1H), 7.28-7.24 (m, 1H), 7.11-7.05 (m, 1H), 3.76 (t, J = 7.6 Hz, 1H), 2.23 (s, 3H), 1.95-1.72 (m, 2H), 1.55-1.37 (m, 2H), 0.97 (t, J = 7.3 Hz, 3H); ^{13}C NMR (101 MHz, $CDCl_3$) δ 206.22, 135.49, 133.46, 131.50, 128.40, 128.16, 125.52, 56.90, 32.78, 25.69, 20.78, 13.90; HRMS (ESI) m/z: $[M + H]^+$ Calcd for $C_{12}H_{16}{}^{79}BrOS^+$ 287.0100; Found 287.0099.

According to ***General Procedure H***.

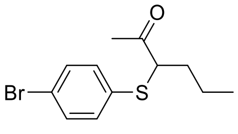

3-((4-bromophenyl)thio)hexan-2-one (7af): Colourless oil (115.8 mg, 40% yield). ^{1}H NMR (400 MHz, $CDCl_3$) δ 7.41 (d, J = 8.1 Hz, 2H), 7.21 (d, J = 8.2 Hz, 2H), 3.60 (t, J = 7.5 Hz, 1H), 2.24 (s, 3H), 1.84-1.61 (m, 2H), 1.55-1.33 (m, 2H), 0.94 (t, J = 7.3 Hz, 3H); ^{13}C NMR (101 MHz, $CDCl_3$) δ 205.29, 133.92, 132.34, 132.29, 122.33, 57.53, 32.40, 26.40, 20.65, 13.88; HRMS (ESI) m/z: $[M + H]^+$ Calcd for $C_{12}H_{16}{}^{79}BrOS^+$ 287.0100; Found 287.0098.

General Procedure for the Synthesis of Racemic 7ag-7ah

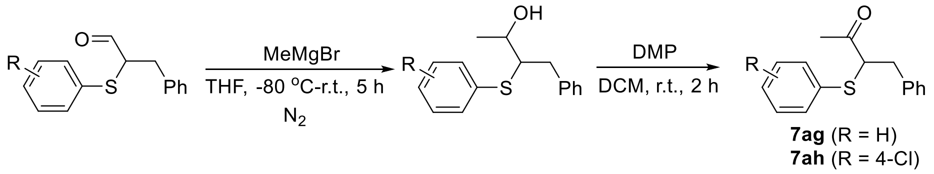

General Procedure I: The appropriate α-thio aldehydes (2 mmol) were dissolved in anhydrous THF (20 mL). To the resulting mixture was added dropwise MeMgBr (2.4 mmol, 1.5 mol/L in THF) at -80 °C under nitrogen. The mixture was stirred at -80 °C for 1 h, then the reaction mixture was moved to room temperature and allowed to stir at the same temperature for 4 h. After that, a saturated aqueous solution of NH_4Cl (10 mL) was added to quench the reaction. After removal of most of the solvent, water (10 mL) was added, and the resulting mixture was extracted with EtOAc (15 mL × 3), and the combined organic phase was washed with brine, dried over $MgSO_4$, and concentrated to give the crude β-hydroxysulfide intermediates. To a solution of the appropriate crude β-hydroxysulfides in dichloromethane (20 mL) was added Dess–Martin periodinane (2 mmol), and the resulting mixture was stirred at room temperature for 2 h. After that, the reaction mixture was washed with saturated aqueous $NaHCO_3$ solution, dried over $MgSO_4$, and concentrated. The residue was purified by flash chromatography (Hexane/EtOAc: 32/1→Hexane/EtOAc: 16/1) on silica gel to give the desired products.

According to ***General Procedure I***.

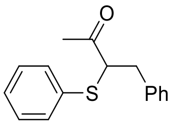

4-phenyl-3-(phenylthio)butan-2-one (7ag): Pale yellow oil (321.4 mg, 63% yield over two steps). ^{1}H NMR (400 MHz, $CDCl_3$) δ 7.38-7.32 (m, 2H), 7.32-7.26 (m, 5H), 7.25-7.17 (m, 3H), 3.91 (dd, *J* = 8.4, 6.8 Hz, 1H), 3.18 (dd, *J* = 14.2, 8.4 Hz, 1H), 3.00 (dd, *J* = 14.2, 6.8 Hz, 1H), 2.21 (s, 3H); ^{13}C NMR (101 MHz, $CDCl_3$) δ 204.38, 138.15, 133.13, 132.82, 129.27, 129.20, 128.68, 128.38, 126.91, 58.88, 36.79, 27.94; HRMS (ESI) m/z: [M + H]$^+$ Calcd for $C_{16}H_{17}OS^+$ 257.0995; Found 257.0994.

According to ***General Procedure I***.

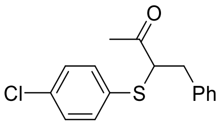

3-((4-chlorophenyl)thio)-4-phenylbutan-2-one (7ah): Pale yellow oil (377.6 mg, 65% yield over two steps). ^{1}H NMR (400 MHz, $CDCl_3$) δ 7.32-7.20 (m, 7H), 7.18 (d, *J* = 7.6 Hz, 2H), 3.90-3.81 (m, 1H), 3.15 (dd, *J* = 14.2, 8.3 Hz, 1H), 2.97 (dd, *J* = 14.1, 6.9 Hz, 1H), 2.21 (s, 3H); ^{13}C NMR (101 MHz, $CDCl_3$) δ 204.02, 137.88, 134.82, 134.62, 131.04, 129.46, 129.18, 128.75, 127.02, 58.85, 36.65, 27.88; HRMS (ESI) m/z: [M + H]$^+$ Calcd for $C_{16}H_{16}{}^{35}ClOS^+$ 291.0605; Found 291.0591.

Procedure for the Synthesis of Racemic 7ai

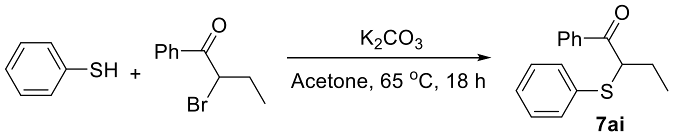

7ai

To a solution of 2-bromo-1-phenylbutan-1-one (3 mmol) in acetone (30 mL) were added thiophenol (3 mmol) and K_2CO_3 (6 mmol), then the reaction mixture was stirred at 65 °C for 18 h. After filtration, the filtrate was concentrated, and the residue obtained was purified by flash chromatography (Hexane/EtOAc: 64/1→Hexane/EtOAc: 32/1) on silica gel to give the desired product **7ai**.

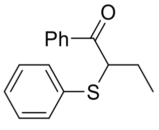

1-phenyl-2-(phenylthio)butan-1-one (7ai): White solid (630.1 mg, 82% yield). ^{1}H NMR (400 MHz, $CDCl_3$) δ 7.93 (d, *J* = 7.9 Hz, 2H), 7.59-7.52 (m, 1H), 7.48-7.40 (m, 2H), 7.37-7.31 (m, 2H), 7.31-7.22 (m, 3H), 4.44-4.32 (m, 1H), 2.12-1.96 (m, 1H), 1.94-1.80 (m, 1H), 1.06 (t, *J* = 7.3 Hz, 3H); ^{13}C NMR (101 MHz, $CDCl_3$) δ 196.13, 136.43, 134.62, 133.14, 132.17, 129.04, 128.70, 128.67, 53.44, 24.38, 12.08; HRMS (ESI) m/z: [M + H]$^+$ Calcd for $C_{16}H_{17}OS^+$ 257.0995; Found 257.0990.

Procedure for the Synthesis of Racemic 7aj

7aj was prepared according to our previous method.[10] To a solution of pentanal (3.0 mmol) in THF (15.0 mL) were added DL-proline (0.9 mmol) and NBS (6.0 mmol), then the resulting mixture was stirred at room temperature for 3 h. After that, the reaction mixture was evaporated under reduced pressure (200 mbar) at 35 °C to remove most of the solvent until solids began to precipitate out. After filtration, the crude 2-bromopentanal was obtained in the filtrate, which was transferred to a microwave tube. THF was added into the tube to make the whole volume 15.0

mL, followed by the addition of H_2O (15.0 mL). Then thiophenols (3.0 mmol) and K_2CO_3 (9.0 mmol) were added into the tube and the resulting mixture was stirred vigorously at room temperature for 10 min to release CO_2. After that, the reaction mixture was irradiated with microwave at 110 °C for 0.5 h. The reaction mixture was then diluted with H_2O (30.0 mL) and extracted with ethyl acetate (20 mL × 3). The combined organic layers were washed with brine and dried with Na_2SO_4. After filtration and removal of the solvent *in vacuo*, the residue was purified by flash chromatography (Hexane/EtOAc: 64/1→Hexane/EtOAc: 32/1) on silica gel to give **7aj**.

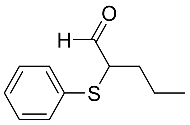

2-(phenylthio)pentanal (7aj): Yellow oil (355.8 mg, 61% yield). ^{1}H NMR (400 MHz, $CDCl_3$) δ 9.36 (d, J = 4.3 Hz, 1H), 7.44-7.34 (m, 2H), 7.34-7.24 (m, 3H), 3.62-3.43 (m, 1H), 1.86-1.73 (m, 1H), 1.72-1.60 (m, 1H), 1.60-1.42 (m, 2H), 0.96 (t, J = 7.3 Hz, 3H); ^{13}C NMR (101 MHz, $CDCl_3$) δ 195.42, 132.91, 132.02, 129.29, 128.24, 56.71, 29.98, 20.33, 13.90; HRMS (ESI) m/z: $[M + H]^+$ Calcd for $C_{11}H_{15}OS^+$ 195.0838; Found 195.0840. The characterization data of this compound were in accordance with the published ones.[10]

Procedure for the Synthesis of Racemic 7ak

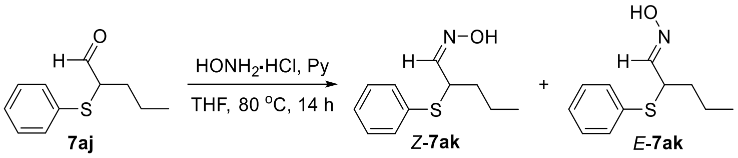

To a solution of **7aj** (1 mmol) in THF (10 mL) were added hydroxylamine hydrochloride (2 mmol) and pyridine (4 mmol), and the resulting mixture was stirred at 80 °C in an oil bath for 14 h. After removal of the solvent, water (20 mL) was added to the residue obtained, and the resulting mixture was extracted with EtOAc (15 mL × 3). Then the combined organic phase was washed with 1 mol/L HCl, saturated aqueous $NaHCO_3$ solution, and brine, and dried over $MgSO_4$. An aliquot of the organic phase was taken for ^{1}H NMR analysis to determine the ratio of Z and E isomers of the products. After removal of the solvent, the residue obtained was purified by flash chromatography (Hexane/EtOAc: 16/1→Hexane/EtOAc: 8/1) on silica gel to give the desired product **7ak**. The reaction gave two stereoisomers (*Z*-**7ak**/*E*-**7ak** = 0.5/1, 132.9 mg, 64% combined yield), which were inseparable by chromatography.

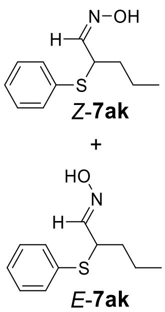

Z-**7ak**/*E*-**7ak** = 0.5/1

Colourless oil. ^{1}H NMR (400 MHz, $CDCl_3$) δ 8.10 (s, 0.51H), 7.66 (s, 1.00H), 7.44-7.36 (m, 3.08H), 7.32-7.19 (m, 5.83H), 6.60 (d, J = 8.4 Hz, 0.53H), 4.69-4.57 (m, 0.53H), 3.80-3.67 (m, 1.03H), 1.78-1.56 (m, 3.49H), 1.56-1.41 (m, 3.19H), 0.99-0.92 (m, 4.65H); ^{13}C NMR (101 MHz, $CDCl_3$) δ 152.70, 152.06, 133.94, 133.25, 132.99, 131.61, 129.01, 128.97, 127.70, 127.21, 47.32, 40.29, 34.31, 34.25, 20.51, 20.43, 13.86, 13.80; HRMS (ESI) m/z: $[M + H]^+$ Calcd for $C_{11}H_{16}NOS^+$ 210.0947; Found 210.0944. The configuration of the carbon-nitrogen double bond was confirmed by NOESY analysis.

Procedure for the Synthesis of Racemic 7al

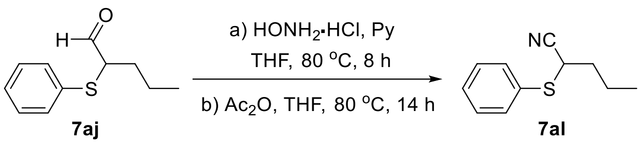

To a solution of **7aj** (1 mmol) in THF (10 mL) were added hydroxylamine hydrochloride (2 mmol) and pyridine (4 mmol), and the mixture was stirred at 80 °C in an oil bath for 8 h. Then Ac_2O (5 mmol) was added, and the resulting mixture was stirred at 80 °C for 14 h. After removal of the solvent, water (20 mL) was added to the residue obtained, and the resulting mixture was extracted with EtOAc (15 mL × 3). Then the combined organic phase was washed with 1 mol/L HCl, saturated aqueous $NaHCO_3$ solution, and brine, and dried over $MgSO_4$. After removal of the solvent, the residue obtained was purified by flash chromatography (Hexane/EtOAc: 32/1→Hexane/EtOAc: 16/1) on silica gel to give the desired product **7al**.

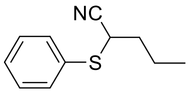

2-(phenylthio)pentanenitrile (7al): Pale yellow oil (99.1 mg, 52% yield). ^{1}H NMR (400 MHz, $CDCl_3$) δ 7.67-7.55 (m, 2H), 7.46-7.34 (m, 3H), 3.70 (t, J = 7.4 Hz, 1H), 1.89-1.77 (m, 2H), 1.69-1.56 (m, 2H), 0.97 (t, J = 7.3 Hz, 3H); ^{13}C NMR (101 MHz, $CDCl_3$) δ 134.63, 130.95, 129.56, 119.45, 37.01, 34.50, 20.49, 13.41; HRMS (ESI) m/z: [M + H]$^+$ Calcd for $C_{11}H_{14}NS^+$ 192.0841; Found 192.0838. The characterization data of this compound were in accordance with the published ones.[11]

Procedure for the Synthesis of Racemic 7am

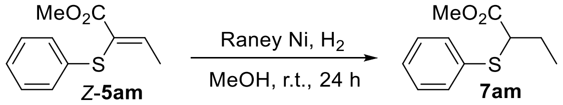

To a solution of Z-**5am** (1 mmol) in MeOH (15 mL) was added Raney Ni (20 % w/w), and the mixture was stirred at room temperature for 24 h under hydrogen and monitored by TLC. After removal of the Raney Ni by filtration with celite, the filtrate was concentrated and the residue obtained was purified by flash chromatography (Hexane/EtOAc: 16/1→Hexane/EtOAc: 8/1) on silica gel to give the desired product **7am**.

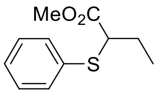

methyl 2-(phenylthio)butanoate (7am): Colourless oil (52.1 mg, 25% yield). ^{1}H NMR (400 MHz, $CDCl_3$) δ 7.48-7.42 (m, 2H), 7.35-7.27 (m, 3H), 3.67 (s, 3H), 3.59 (dd, J = 8.0, 6.9 Hz, 1H), 1.98-1.85 (m, 1H), 1.85-1.73 (m, 1H), 1.03 (t, J = 7.4 Hz, 3H); ^{13}C NMR (101 MHz, $CDCl_3$) δ 172.83, 133.57, 132.92, 129.07, 128.01, 52.60, 52.28, 25.28, 12.00; HRMS (ESI) m/z: [M + H]$^+$ Calcd for $C_{11}H_{15}O_2S^+$ 211.0787; Found 211.0783.

General Procedure for the Synthesis of Racemic 10aa-10ae

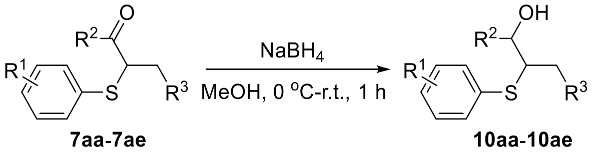

General Procedure J: To a solution of the appropriate α-thio ketones **7** (0.2 mmol) in MeOH (5 mL)

at 0 °C was added $NaBH_4$ (0.2 mmol), then the reaction mixture was stirred at room temperature for 1 h. After removal of most of the solvent, a saturated aqueous solution of NH_4Cl (10 mL) was added. The resulting mixture was extracted with EtOAc (10 mL × 3), and the combined organic phase was washed with brine, dried over $MgSO_4$, and concentrated. The residue obtained was directly used for ^{1}HNMR analysis to determine the *syn*/*anti* ratios and then purified by flash chromatography (Hexane/EtOAc: 16/1→Hexane/EtOAc: 8/1) on silica gel to give the desired products **10**.

According to ***General Procedure J***, the reaction gave two pairs of diastereoisomers (*syn*-**10aa**/*anti*-**10aa** = 7.8/1, 35.3 mg, 84% combined yield), which were inseparable by chromatography.

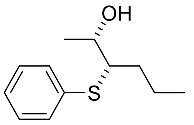

***syn*-3-(phenylthio)hexan-2-ol (*syn*-10aa)**: Colourless oil. ^{1}H NMR (400 MHz, $CDCl_3$) δ 7.45 (d, *J* = 7.9 Hz, 2H), 7.32-7.22 (m, 3H), 3.80-3.65 (m, 1H), 2.99-2.87 (m, 1H), 2.63 (d, *J* = 3.0 Hz, 1H), 1.76-1.63 (m, 2H), 1.56-1.37 (m, 2H), 1.26 (d, *J* = 6.2 Hz, 3H), 0.92 (t, *J* = 6.9 Hz, 3H); ^{13}C NMR (101 MHz, $CDCl_3$) δ 134.69, 132.51, 129.09, 127.30, 69.25, 58.99, 33.35, 20.61, 20.21, 14.07; HRMS (ESI) m/z: [M + H]$^+$ Calcd for $C_{12}H_{19}OS^+$ 211.1151; Found 211.1149.

According to ***General Procedure J***, the reaction gave two pairs of diastereoisomers (*syn*-**10ab**/*anti*-**10ab** = 5.1/1, 38.6 mg, 85% combined yield), which were separable by chromatography.

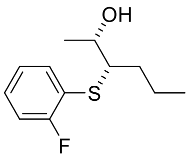

***syn*-3-((2-fluorophenyl)thio)hexan-2-ol (*syn*-10ab)**: Colourless oil. ^{1}H NMR (400 MHz, $CDCl_3$) δ 7.52-7.45 (m, 1H), 7.31-7.23 (m, 1H), 7.13-7.03 (m, 2H), 3.78-3.67 (m, 1H), 2.96 (ddd, *J* = 9.6, 6.3, 3.8 Hz, 1H), 2.31 (s, 1H), 1.76-1.60 (m, 2H), 1.57-1.37 (m, 2H), 1.25 (d, *J* = 6.2 Hz, 3H), 0.92 (t, *J* = 7.1 Hz, 3H); ^{13}C NMR (101 MHz, $CDCl_3$) δ 162.74 (d, J_{C-F} = 245.5 Hz), 135.75, 129.91 (d, J_{C-F} = 8.1 Hz), 124.66 (d, J_{C-F} = 3.8 Hz), 121.41 (d, J_{C-F} = 18.0 Hz), 116.08 (d, J_{C-F} = 23.5 Hz), 69.45, 58.49, 33.34, 20.55, 20.14, 14.05; ^{19}F NMR (376 MHz, $CDCl_3$) δ -107.02. HRMS (ESI) m/z: [M - OH]$^+$ Calcd for $C_{12}H_{16}FS^+$ 211.0951; Found 211.0953.

According to ***General Procedure J***, the reaction gave two pairs of diastereoisomers (*syn*-**10ac**/*anti*-**10ac** = 4.5/1, 39.5 mg, 81% combined yield), which were separable by chromatography.

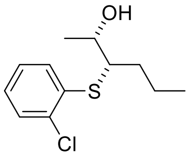

***syn*-3-((2-chlorophenyl)thio)hexan-2-ol (*syn*-10ac)**: Colourless oil. ^{1}H NMR (400 MHz, $CDCl_3$) δ 7.47 (d, *J* = 7.7 Hz, 1H), 7.39 (d, *J* = 7.7 Hz, 1H), 7.24-7.11 (m, 2H), 3.91-3.76 (m, 1H), 3.20-3.10 (m, 1H), 2.34 (d, *J* = 4.4 Hz, 1H), 1.80-1.61 (m, 2H), 1.58-1.45 (m, 2H), 1.27 (d, *J* = 6.3 Hz, 3H), 0.93 (t, *J* = 6.8 Hz, 3H); ^{13}C NMR (101 MHz, $CDCl_3$) δ 135.80, 134.97, 132.37, 130.11, 127.85, 127.29, 69.60, 57.22, 33.29, 20.57, 20.12, 14.16; HRMS (ESI) m/z: [M - OH]$^+$ Calcd for $C_{12}H_{16}{}^{35}ClS^+$ 227.0656; Found 227.0659.

According to ***General Procedure J***, the reaction gave two pairs of diastereoisomers (*syn*-**10ad**/*anti*-**10ad** = 9.7/1, 39.1 mg, 80% combined yield), which were inseparable by chromatography.

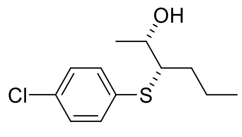

syn-**3-((4-chlorophenyl)thio)hexan-2-ol** (*syn*-**10ad**): Colourless oil. ^{1}H NMR (400 MHz, $CDCl_3$) δ 7.37 (d, *J* = 8.4 Hz, 2H), 7.25 (d, *J* = 8.3 Hz, 2H), 3.81-3.60 (m, 1H), 2.93-2.85 (m, 1H), 2.53 (d, *J* = 3.6 Hz, 1H), 1.71-1.60 (m, 2H), 1.53-1.37 (m, 2H), 1.25 (d, *J* = 6.2 Hz, 3H), 0.91 (t, *J* = 6.8 Hz, 3H); ^{13}C NMR (101 MHz, $CDCl_3$) δ 133.71, 133.41, 129.23, 69.25, 59.26, 33.33, 20.60, 20.26, 14.04; HRMS (ESI) m/z: $[M - OH]^+$ Calcd for $C_{12}H_{16}{}^{35}ClS^+$ 227.0656; Found 227.0651.

According to ***General Procedure J***, the reaction gave two pairs of diastereoisomers (*syn*-**10ae**/*anti*-**10ae** = 5.0/1, 49.7 mg, 86% combined yield), which were separable by chromatography.

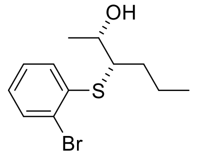

syn-**3-((2-bromophenyl)thio)hexan-2-ol** (*syn*-**10ae**): Colourless oil. ^{1}H NMR (400 MHz, $CDCl_3$) δ 7.57 (d, *J* = 8.0 Hz, 1H), 7.46 (d, *J* = 7.9 Hz, 1H), 7.29-7.22 (m, 1H), 7.10-7.03 (m, 1H), 3.92-3.81 (m, 1H), 3.21-3.13 (m, 1H), 2.26 (d, *J* = 4.6 Hz, 1H), 1.82-1.61 (m, 2H), 1.58-1.46 (m, 2H), 1.28 (d, *J* = 6.2 Hz, 3H), 0.94 (t, *J* = 6.8 Hz, 3H); ^{13}C NMR (101 MHz, $CDCl_3$) δ 137.24, 133.42, 131.77, 127.95, 127.82, 126.06, 69.63, 57.54, 33.28, 20.61, 20.18, 14.19; HRMS (ESI) m/z: $[M - OH]^+$ Calcd for $C_{12}H_{16}{}^{79}BrS^+$ 271.0151; Found 271.0154.

Procedure for the Synthesis of 6aa

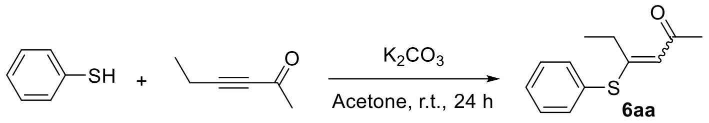

To a solution of thiophenol (2 mmol) and 3-hexyn-2-one (2.4 mmol) in acetone (20 mL) was added K_2CO_3 (4 mmol), and the resulting mixture was stirred at room temperature for 24 h. After filtration, an aliquot of the filtrate was taken for ^{1}H NMR analysis to determine the ratio of *Z* and *E* isomers of the products. Then the filtrate was concentrated, and the residue obtained was purified by flash chromatography (Hexane/EtOAc: 32/1→Hexane/EtOAc: 16/1) on silica gel to give the *Z* and *E* products. The reaction gave two stereoisomers (*Z*-**6aa**/*E*-**6aa** = 3.2/1, 370.3 mg, 90% combined yield), which were separable by chromatography.

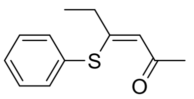

(*Z*)-4-(phenylthio)hex-3-en-2-one (*Z*-**6aa**): White solid. ^{1}H NMR (400 MHz, DMSO-d_6) δ 7.58-7.52 (m, 2H), 7.50-7.40 (m, 3H), 6.44 (s, 1H), 2.17 (s, 3H), 2.11-2.03 (m, 2H), 0.88 (t, *J* = 7.4 Hz, 3H); ^{13}C NMR (101 MHz, DMSO-d_6) δ 195.37, 161.29, 135.47, 130.35, 129.53, 129.31, 118.80, 30.36, 29.20, 13.71; LRMS (ESI) m/z: 207 $[M+H]^+$. The configuration of the carbon-carbon double bond of this compound was confirmed by NOESY analysis.

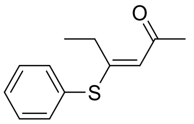

(*E*)-4-(phenylthio)hex-3-en-2-one (*E*-6aa): Colourless oil. ^{1}H NMR (400 MHz, DMSO-d_6) δ 7.57-7.50 (m, 5H), 5.61 (s, 1H), 2.71 (q, J = 7.4 Hz, 2H), 1.95 (s, 3H), 1.13 (t, J = 7.4 Hz, 3H); ^{13}C NMR (101 MHz, DMSO-d_6) δ 193.97, 164.28, 135.13, 130.16, 130.09, 128.70, 117.88, 31.48, 26.34, 13.90; LRMS (ESI) m/z: 207 $[M+H]^+$. The configuration of the carbon-carbon double bond of this compound was confirmed by NOESY analysis.

Procedure for the Synthesis of 6ab

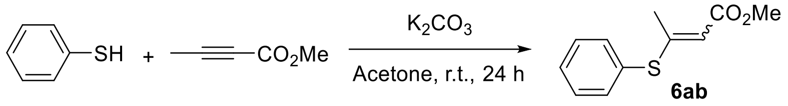

To a solution of thiophenol (2 mmol) and methyl 2-butynoate (4 mmol) in acetone (20 mL) was added K_2CO_3 (4 mmol), and the resulting mixture was stirred at room temperature for 24 h. After filtration, an aliquot of the filtrate was taken for ^{1}H NMR analysis to determine the ratio of *Z* and *E* isomers of the products. Then the filtrate was concentrated, and the residue obtained was purified by flash chromatography (Hexane/EtOAc: 16/1→Hexane/EtOAc: 8/1) on silica gel to give the *Z* and *E* products. The reaction gave two stereoisomers (*Z*-**6ab**/*E*-**6ab** = 5.5/1, 345.7 mg, 83% combined yield), which were separable by chromatography.

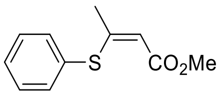

methyl (*Z*)-3-(phenylthio)but-2-enoate (*Z*-6ab): Colourless oil. ^{1}H NMR (400 MHz, $CDCl_3$) δ 7.60-7.50 (m, 2H), 7.45-7.33 (m, 3H), 5.85 (s, 1H), 3.74 (s, 3H), 1.81 (s, 3H); ^{13}C NMR (101 MHz, $CDCl_3$) δ 166.77, 158.80, 136.21, 130.89, 129.60, 129.17, 111.58, 51.27, 25.24; LRMS (ESI) m/z: 209 $[M+H]^+$. The configuration of the carbon-carbon double bond of this compound was confirmed by NOESY analysis.

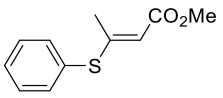

methyl (*E*)-3-(phenylthio)but-2-enoate (*E*-6ab): Colourless oil. ^{1}H NMR (400 MHz, $CDCl_3$) δ 7.53-7.46 (m, 2H), 7.46-7.39 (m, 3H), 5.23 (s, 1H), 3.61 (s, 3H), 2.43 (s, 3H); ^{13}C NMR (101 MHz, $CDCl_3$) δ 165.89, 160.45, 135.65, 130.05, 129.90, 129.68, 110.45, 50.96, 20.19; LRMS (ESI) m/z: 209 $[M+H]^+$. The configuration of the carbon-carbon double bond of this compound was confirmed by NOESY analysis.

Procedure for the Synthesis of Racemic 8aa

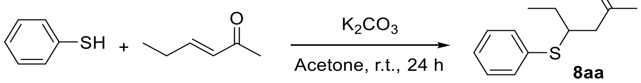

To a solution of thiophenol (2 mmol) and (*E*)-hex-3-en-2-one (2.4 mmol) in acetone (20 mL) was added K_2CO_3 (4 mmol), and the resulting mixture was stirred at room temperature for 24 h. After filtration, the filtrate was concentrated, and the residue obtained was purified by flash chromatography (Hexane/EtOAc: 16/1→Hexane/EtOAc: 8/1) on silica gel to give the desired product **8aa**.

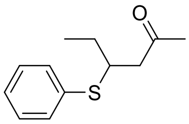

4-(phenylthio)hexan-2-one (8aa): Colourless oil (295.1 mg, 71% yield). ^{1}H NMR (400 MHz, $CDCl_3$) δ 7.45-7.37 (m, 2H), 7.34-7.19 (m, 3H), 3.61-3.49 (m, 1H), 2.77-2.58 (m, 2H), 2.13 (s, 3H), 1.71-1.50 (m, 2H), 1.02 (t, J = 7.3 Hz, 3H); ^{13}C NMR (101 MHz, $CDCl_3$) δ 206.89, 134.63, 132.45, 129.05, 127.24, 48.63, 45.38, 30.78, 27.75, 11.42; HRMS (ESI) m/z: $[M + H]^+$ Calcd for $C_{12}H_{17}OS^+$ 209.0995; Found 209.0993.

Procedure for the Synthesis of Racemic 8ab

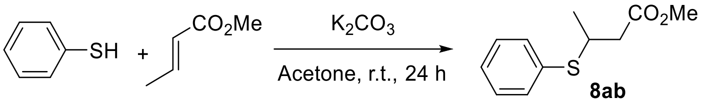

To a solution of thiophenol (2 mmol) and methyl (*E*)-but-2-enoate (4 mmol) in acetone (20 mL) was added K_2CO_3 (4 mmol), and the resulting mixture was stirred at room temperature for 24 h. After filtration, the filtrate was concentrated, and the residue obtained was purified by flash chromatography (Hexane/EtOAc: 16/1→Hexane/EtOAc: 8/1) on silica gel to give the desired product **8ab**.

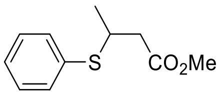

methyl 3-(phenylthio)butanoate (8ab): Colourless oil (34.0 mg, 8% yield). ^{1}H NMR (400 MHz, $CDCl_3$) δ 7.44 (d, J = 7.7 Hz, 2H), 7.36-7.21 (m, 3H), 3.66 (s, 3H), 3.65-3.57 (m, 1H), 2.65 (dd, J = 15.6, 5.9 Hz, 1H), 2.44 (dd, J = 15.6, 8.5 Hz, 1H), 1.33 (d, J = 6.8 Hz, 3H); ^{13}C NMR (101 MHz, $CDCl_3$) δ 171.94, 133.85, 132.98, 129.06, 127.57, 51.83, 41.74, 39.56, 20.98; HRMS (ESI) m/z: $[M + H]^+$ Calcd for $C_{11}H_{15}O_2S^+$ 211.0787; Found 211.0790.

Procedure for the Synthesis of Racemic 11aa and (*S*)-11aa

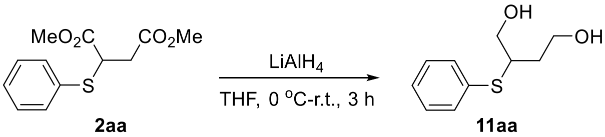

To a solution of **2aa** (1 mmol) in anhydrous THF (15 mL) at 0 °C was added dropwise $LiAlH_4$ (2 mmol, 1 mol/L in THF), and the resulting mixture was moved to room temperature and allowed to stir at the same temperature for 3 h. Then dichloromethane (15 mL) was added to dilute the reaction mixture, and water (1 mL) was added dropwise to quench the reaction. The solid which precipitated out was removed by filtration, and the filtrate was concentrated. The residue obtained was purified by flash chromatography (Hexane/EtOAc: 4/1→Hexane/EtOAc: 2/1) on silica gel to give the desired product **11aa**.

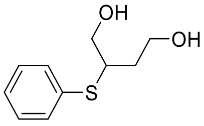

2-(phenylthio)butane-1,4-diol (11aa): Colourless oil (166.8 mg, 84% yield). ^{1}H NMR (400 MHz, DMSO-d_6) δ 7.38 (d, J = 7.7 Hz, 2H), 7.35-7.27 (m, 2H), 7.25-7.18 (m, 1H), 4.94 (t, J = 5.6 Hz, 1H), 4.58 (t, J = 5.1 Hz, 1H), 3.64-3.49 (m, 3H), 3.44-3.36 (m, 1H), 3.34-3.26 (m, 1H), 2.02-1.88 (m, 1H), 1.60-1.44 (m, 1H); ^{13}C NMR (101 MHz, DMSO-d_6) δ 135.43, 130.24, 128.97, 126.23, 63.77, 58.26, 46.55, 34.33; HRMS (ESI) m/z: $[M - OH]^+$ Calcd for $C_{10}H_{13}OS^+$ 181.0682; Found 181.0680.

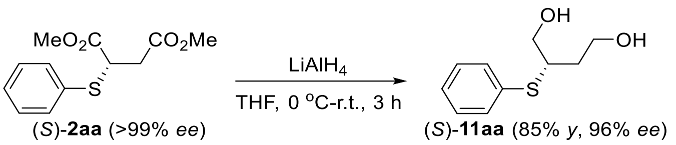

Employing (*S*)-**2aa** (1 mmol, >99% ee) as the substrate, (*S*)-**11aa** (168.1 mg, 85% yield, 96% ee) was prepared similarly following the procedure for the synthesis of racemic **11aa**. $[\alpha]_D^{25}$ = -37.0 (*c* 1.0, MeOH) for (*S*)-**11aa** with 96% ee. [lit[12]: $[\alpha]_D^{23}$ = -32.6 (*c* 3.5, MeOH) for (*S*)-**11aa** with 81% ee.]

Reaction Condition Optimization for ENE Biocatalysed Enantioselective Reduction of 1aa

Supplementary Table 3. Reaction condition optimization for ENE biocatalysed enantioselective reduction of **1aa**.[a]

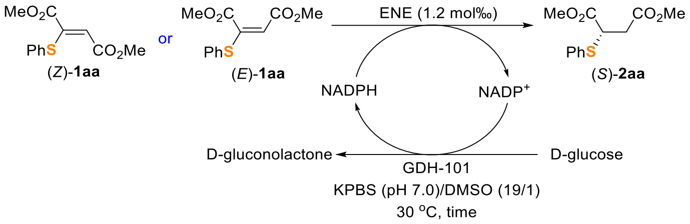

Entry	Substrate	ENE	Time (h)	Conv. of 2aa (%)[b]	ee of 2aa (%)[b]
1	(*Z*)-**1aa**	ENE-101	0.5	78	>99 (*S*)
2	(*Z*)-**1aa**	ENE-101	1	96	>99 (*S*)
3	(*Z*)-**1aa**	ENE-101	1.5	98	>99 (*S*)
4	(*Z*)-**1aa**	ENE-101	2	99	>99 (*S*)
5	(*Z*)-**1aa**	ENE-101	4	>99	>99 (*S*)
6	(*Z*)-**1aa**	ENE-101	6	>99	>99 (*S*)
7	(*Z*)-**1aa**	ENE-101	8	>99	>99 (*S*)
8	(*Z*)-**1aa**	ENE-101	10	>99	>99 (*S*)
9	(*Z*)-**1aa**	ENE-101	12	>99	>99 (*S*)
10	(*Z*)-**1aa**	ENE-101	24	>99	>99 (*S*)
11	(*E*)-**1aa**	ENE-101	2	34	92 (*S*)
12	(*E*)-**1aa**	ENE-101	4	55	90 (*S*)
13	(*E*)-**1aa**	ENE-101	6	68	90 (*S*)
14	(*E*)-**1aa**	ENE-101	8	78	90 (*S*)
15	(*E*)-**1aa**	ENE-101	10	85	90 (*S*)
16	(*E*)-**1aa**	ENE-101	12	87	90 (*S*)
17	(*E*)-**1aa**	ENE-101	24	>99	88 (*S*)
18	(*Z*)-**1aa**	NCR	24	83	98 (*S*)
19	(*Z*)-**1aa**	pQR1440	24	87	97 (*S*)
20	(*Z*)-**1aa**	pQR1907	24	85	98 (*S*)
21	(*E*)-**1aa**	NCR	24	13	98 (*S*)
22	(*E*)-**1aa**	pQR1440	24	10	97 (*S*)
23	(*E*)-**1aa**	pQR1907	24	34	97 (*S*)

[a]Reaction conditions: (*Z*)-**1aa** or (*E*)-**1aa** (0.02 mmol), ENE-101 (5.0 mg, 65.8 U/mg, 1.2 mol‰) or NCR (0.5 mg) or pQR1440 (0.5 mg) or pQR1907 (0.5 mg), GDH-101 (2.5 mg, 32.0 U/mg), D-glucose (0.1 mmol), $NADP^+$ (1 μmol), 950 μL KPBS (250 mM, pH 7.0), DMSO (50 μL), 30 °C, 0.5-24 h. [b]Determined by chiral HPLC analysis.

General Procedure for ENE-101 Biocatalysed Enantioselective Reduction of (*Z*)-1 and/or (*E*)-1 into (*S*)-2

General Procedure K: To a solution of ENE-101 (25 mg, 65.8 U/mg, 1.2 mol‰), GDH-101 (12.5 mg, 32.0 U/mg) and D-glucose (0.5 mmol) in 1.7 mL KPBS (250 mM, pH 7.0) in a 10 mL vial were added a solution of $NADP^+$ (5 μmol) in 200 μL KPBS (250 mM, pH 7.0) and a solution of (*Z*)-**1** and/or (*E*)-**1** (0.1 mmol) in DMSO (100 μL), then the vial was incubated at 30 °C with shaking at 200 rpm for 24 h. After that, the reaction mixture was extracted with EtOAc (2 mL × 5) and centrifuged at 25 °C and 8000 rpm for 5 min, and the combined organic phase was dried with $MgSO_4$. An aliquot of the organic phase was taken for HPLC analysis to determine the conversions and ee values. Then the organic solvent was removed *in vacuo*, and the residue obtained was purified by column chromatography on silica gel to give the desired products **2**.

Supplementary Table 4. ENE-101 biocatalysed enantioselective reduction of (*Z*)-**1** and/or (*E*)-**1** into (*S*)-**2**.[a]

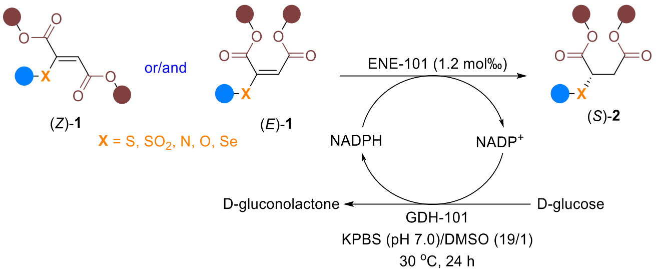

Substrate	Product	Conv. (%)[b]	Product Mass (mg), Yield (%)[c]	ee (%)[b]
(*Z*)-**1aa**	**2aa**	>99	24.1 mg, 95%	>99 (*S*)
(*E*)-**1aa**	**2aa**	99	23.6 mg, 93%	88 (*S*)
(*Z*)-**1aa**/(*E*)-**1aa** = 4.1/1	**2aa**	>99	23.2 mg, 91%	96 (*S*)
(*Z*)-**1ab**	**2ab**	>99	25.6 mg, 94%	>99 (*S*)
(*E*)-**1ab**	**2ab**	>99	25.1 mg, 92%	99 (*S*)
(*Z*)-**1ab**/(*E*)-**1ab** = 2.5/1	**2ab**	>99	25.8 mg, 95%	>99 (*S*)
(*Z*)-**1ac**	**2ac**	>99	27.6 mg, 96%	99 (*S*)
(*E*)-**1ac**	**2ac**	99	26.6 mg, 92%	98 (*S*)
(*Z*)-**1ac**/(*E*)-**1ac** = 3.5/1	**2ac**	99	26.2 mg, 91%	99 (*S*)
(*Z*)-**1ad**	**2ad**	91	22.0 mg, 76%	99 (*S*)
(*E*)-**1ad**	**2ad**	95	23.3 mg, 81%	73 (*S*)
(*Z*)-**1ad**/(*E*)-**1ad** = 1.2/1	**2ad**	92	21.2 mg, 73%	85 (*S*)
(*Z*)-**1ae**	**2ae**	>99	28.3 mg, 85%	94 (*S*)
(*E*)-**1ae**	**2ae**	>99	28.9 mg, 87%	97 (*S*)
(*Z*)-**1ae**/(*E*)-**1ae** = 2.7/1	**2ae**	>99	29.4 mg, 88%	99 (*S*)
(*Z*)-**1af**	**2af**	75	16.8 mg, 50%	99 (*S*)
(*E*)-**1af**	**2af**	89	23.6 mg, 71%	72 (*S*)
(*Z*)-**1af**/(*E*)-**1af** = 1.3/1	**2af**	85	18.1 mg, 54%	86 (*S*)
(*Z*)-**1ag**	**2ag**	98	22.5 mg, 84%	99 (*S*)
(*E*)-**1ag**	**2ag**	81	16.7 mg, 62%	91 (*S*)
(*Z*)-**1ag**/(*E*)-**1ag** = 0.9/1	**2ag**	91	19.9 mg, 74%	94 (*S*)
(*Z*)-**1ah**	**2ah**	>99	26.7 mg, 94%	99 (*S*)
(*E*)-**1ah**	**2ah**	99	27.2 mg, 96%	90 (*S*)

(*Z*)-**1ah**/(*E*)-**1ah** = 2.8/1	**2ah**	>99	26.2 mg, 92%	95 (*S*)
(*Z*)-**1ai**	**2ai**	>99	26.3 mg, 93%	>99 (*S*)
(*E*)-**1ai**	**2ai**	48	/	79 (*S*)
(*Z*)-**1ai**/(*E*)-**1ai** = 4.4/1	**2ai**	93	22.8 mg, 80%	96 (*S*)
(*Z*)-**1aj**	**2aj**	>99	26.6 mg, 94%	99 (*S*)
(*E*)-**1aj**	**2aj**	27	/	90 (*S*)
(*Z*)-**1aj**/(*E*)-**1aj** = 4.4/1	**2aj**	97	24.3 mg, 86%	97 (*S*)
(*Z*)-**1ak**	**2ak**	87	17.0 mg, 56%	>99 (*S*)
(*E*)-**1ak**	**2ak**	34	/	99 (*S*)
(*Z*)-**1ak**/(*E*)-**1ak** = 0.9/1	**2ak**	65	11.4 mg, 37%	>99 (*S*)
(*Z*)-**1al**	**2al**	91	20.5 mg, 67%	99 (*S*)
(*E*)-**1al**	**2al**	22	/	92 (*S*)
(*Z*)-**1al**/(*E*)-**1al** = 1.1/1	**2al**	74	12.5 mg, 41%	97 (*S*)
(*Z*)-**1am**	**2am**	>99	23.4 mg, 91%	99 (*S*)
(*E*)-**1am**	**2am**	90	20.7 mg, 80%	16 (*S*)
(*Z*)-**1am**/(*E*)-**1am** = 0.4/1	**2am**	93	21.4 mg, 83%	37 (*S*)
(*Z*)-**1an**	**2an**	>99	24.6 mg, 92%	97 (*S*)
(*E*)-**1an**	**2an**	27	/	50 (*S*)
(*Z*)-**1an**/(*E*)-**1an** = 0.5/1	**2an**	55	7.6 mg, 28%	19 (*S*)
(*Z*)-**1ao**	**2ao**	>99	26.2 mg, 93%	96 (*S*)
(*E*)-**1ao**	**2ao**	29	/	61 (*S*)
(*Z*)-**1ao**/(*E*)-**1ao** = 0.5/1	**2ao**	59	7.7 mg, 27%	24 (*S*)
(*Z*)-**1ap**	**2ap**	>99	28.4 mg, 95%	98 (*S*)
(*E*)-**1ap**	**2ap**	45	/	54 (*S*)
(*Z*)-**1ap**/(*E*)-**1ap** = 0.5/1	**2ap**	71	11.8 mg, 40%	12 (*S*)
(*Z*)-**1aq**	**2aq**	>99	25.3 mg, 90%	94 (*S*)
(*E*)-**1aq**	**2aq**	43	/	64 (*R*)
(*Z*)-**1aq**/(*E*)-**1aq** = 0.6/1	**2aq**	64	10.2 mg, 36%	14 (*S*)
(*Z*)-**1ar**	**2ar**	>99	23.4 mg, 94%	>99 (*S*)
(*E*)-**1ar**	**2ar**	20	/	66 (*R*)
(*Z*)-**1ar**/(*E*)-**1ar** = 0.7/1	**2ar**	42	/	23 (*S*)
(*Z*)-**1as**	**2as**	99	24.1 mg, 93%	>99 (*S*)
(*E*)-**1as**	**2as**	26	/	90 (*S*)
(*Z*)-**1as**/(*E*)-**1as** = 1.1/1	**2as**	44	/	97 (*S*)
(*E*)-**1at**	**2at**	79	13.5 mg, 51%	62 (*S*)
(*Z*)-**1au**	**2au**	99	25.4 mg, 90%	99 (*S*)
(*E*)-**1au**	**2au**	27	/	2 (*S*)
(*Z*)-**1au**/(*E*)-**1au** = 4/1	**2au**	80	17.7 mg, 63%	95 (*S*)
(*Z*)-**1av**	**2av**	27	/	>99 (*S*)
(*E*)-**1av**	**2av**	0	/	/
(*Z*)-**1aw**	**2aw**	2	/	>99 (*S*)
(*E*)-**1ax**	**2ax**	98	19.7 mg, 89%	69 (*S*)
(*Z*)-**1ay**	**2ay**	>99	23.4 mg, 82%	90 (*S*)
(*Z*)-**1az**	**2az**	>99	24.1 mg, 80%	89 (*S*)
(*E*)-**1ba**	**2ba**	24	/	>99 (*S*)

(*Z*)-**1bb**/(*E*)-**1bb** = 0.8/1	**2bb**	43	/	>99 (*S*)
(*Z*)-**1bc**/(*E*)-**1bc** = 1/1	**2bc**	27	/	>99 (*S*)
(*Z*)-**1bd**	**2bd**	>99	22.7 mg, 75%	99 (*S*)
(*E*)-**1bd**	**2bd**	91	23.4 mg, 78%	60 (*S*)
(*Z*)-**1bd** /(*E*)-**1bd** = 5.9/1	**2bd**	98	21.2 mg, 70%	89 (*S*)

[a]Reaction conditions: (*Z*)-**1** and/or (*E*)-**1** (0.1 mmol), ENE-101 (25 mg, 65.8 U/mg, 1.2 mol‰), GDH-101 (12.5 mg, 32.0 U/mg), D-glucose (0.5 mmol), $NADP^+$ (5 μmol), 1.9 mL KPBS (250 mM, pH 7.0), DMSO (100 μL), 30 °C, 24 h. [b]Determined by chiral HPLC analysis. [c]Isolated yield.

Reaction Condition Optimization for Two-step One-pot Chemoenzymatic Cascade for the Enantioselective Synthesis of (*S*)-2aa

Supplementary Table 5. Reaction condition optimization for two-step one-pot chemoenzymatic cascade for the enantioselective synthesis of (*S*)-**2aa**.[a]

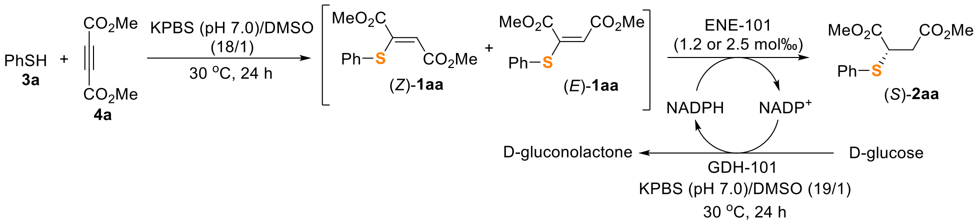

Entry	Conv. of 2aa (%)[b]	ee of 2aa (%)[b]	Yield of 2aa (%)[c]
1	75	92 (*S*)	55
2[d]	77	91 (*S*)	56
3[e]	59	92 (*S*)	41
4[f]	74	90 (*S*)	54
5[d,f]	76	89 (*S*)	53
6[g]	87	92 (*S*)	65
7[g,h]	88	91 (*S*)	65

[a]Reaction conditions: **3a** (0.1 mmol), **4a** (0.1 mmol), 1.8 mL KPBS (250 mM, pH 7.0), DMSO (100 μL), 30 °C, 24 h; then ENE-101 (25 mg, 65.8 U/mg, 1.2 mol‰), GDH-101 (12.5 mg, 32.0 U/mg), D-glucose (0.5 mmol), $NADP^+$ (5 μmol) in 100 μL KPBS (250 mM, pH 7.0), 30 °C, 24 h. [b]Determined by chiral HPLC analysis. [c]Isolated yield. [d]The second step was carried out for 48 h. [e]**4a** (0.12 mmol) was used. [f]The reaction was performed at 37 °C for both steps. [g]**3a** (0.1 mmol), **4a** (0.1 mmol), 3.6 mL KPBS (250 mM, pH 7.0), DMSO (200 μL), 30 °C, 24 h; then ENE-101 (50 mg, 65.8 U/mg, 2.5 mol‰), GDH-101 (25 mg, 32.0 U/mg), D-glucose (1 mmol), $NADP^+$ (10 μmol) in 200 μL KPBS (250 mM, pH 7.0), 30 °C, 24 h. [h]NAD^+ was used instead of $NADP^+$ as the cofactor.

General Procedure for Two-step One-pot Chemoenzymatic Cascade for the Enantioselective Synthesis of (*S*)-2

General Procedure L: 3.6 mL KPBS (250 mM, pH 7.0) was added to a solution of **3** (0.1 mmol) and **4a** (0.1 mmol) in DMSO (200 μL) in a 10 mL vial, and the resulting mixture was stirred at 30 °C for 24 h. After that, ENE-101 (50 mg, 65.8 U/mg, 2.5 mol‰), GDH-101 (25 mg, 32.0 U/mg), D-glucose (1 mmol), and a solution of $NADP^+$ (10 μmol) in 200 μL KPBS (250 mM, pH 7.0) were added sequentially. After addition, the vial was incubated at 30 °C with shaking at 200 rpm for 24 h. Then the reaction mixture was extracted with EtOAc (2 mL × 5) and centrifuged at 25 °C and 8000 rpm for 5 min, and the combined organic phase was dried with $MgSO_4$. An aliquot of the

organic phase was taken for HPLC analysis to determine the conversions and ee values. Then the organic solvent was removed *in vacuo*, and the residue obtained was purified by column chromatography on silica gel to give the desired products **2**.

Supplementary Table 6. Two-step one-pot chemoenzymatic cascade for the enantioselective synthesis of (*S*)-**2**.[a]

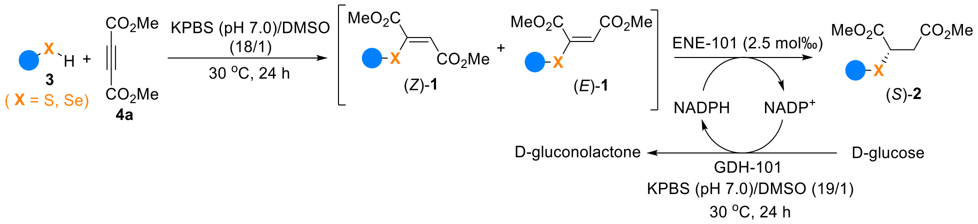

Substrate 3	Product	Conv. (%)[b]	Product Mass (mg), Yield (%)[c]	ee (%)[b]
Thiophenol	**2aa**	87	16.5 mg, 65%	92 (*S*)
2-Fluorothiophenol	**2ab**	76	14.4 mg, 53%	95 (*S*)
2-Chlorothiophenol	**2ac**	60	11.6 mg, 40%	94 (*S*)
4-Chlorothiophenol	**2ad**	66	10.2 mg, 35%	82 (*S*)
2-Bromothiophenol	**2ae**	62	13.3 mg, 40%	96 (*S*)
4-Methylthiophenol	**2ag**	85	16.6 mg, 62%	95 (*S*)
3-Methoxythiophenol	**2ah**	95	23.1 mg, 81%	87 (*S*)
4-Methoxythiophenol	**2ai**	97	23.3 mg, 82%	91 (*S*)
3,5-Dimethylthiophenol	**2aj**	99	24.8 mg, 88%	94 (*S*)
1-Naphthalenethiol	**2ak**	90	17.6 mg, 58%	99 (*S*)
2-Naphthalenethiol	**2al**	70	12.4 mg, 41%	95 (*S*)
Benzyl mercaptan	**2an**	20	/	85 (*S*)
Phenylselenol	**2bd**	46	/	87 (*S*)

[a]Reaction conditions: **3** (0.1 mmol), **4a** (0.1 mmol), 3.6 mL KPBS (250 mM, pH 7.0), DMSO (200 μL), 30 °C, 24 h; then ENE-101 (50 mg, 65.8 U/mg, 2.5 mol‰), GDH-101 (25 mg, 32.0 U/mg), D-glucose (1 mmol), $NADP^+$ (10 μmol) in 200 μL KPBS (250 mM, pH 7.0), 30 °C, 24 h. [b]Determined by chiral HPLC analysis. [c]Isolated yield.

Reaction Condition Optimization for One-Step One-pot Chemoenzymatic Cascade for the Enantioselective Synthesis of (*S*)-2aa

Supplementary Table 7. Reaction condition optimization for one-step one-pot chemoenzymatic cascade for the enantioselective synthesis of (*S*)-**2aa**.[a]

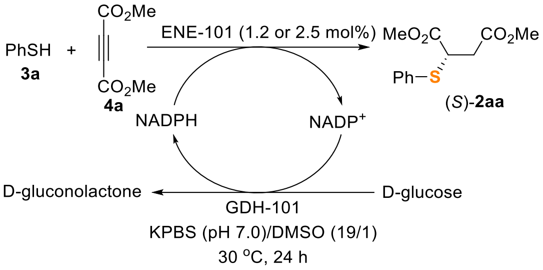

Entry	Conv. of 2aa (%)[b]	ee of 2aa (%)[b]	Yield of 2aa (%)[c]
1	56	92 (*S*)	41
2[d]	59	90 (*S*)	42
3[e]	44	92 (*S*)	/
4[d,e]	47	91 (*S*)	/
5[f]	63	89 (*S*)	46
6[d,f]	65	88 (*S*)	46
7[g]	73	92 (*S*)	56
8[g,h]	71	91 (*S*)	53

[a]Reaction conditions: **3a** (0.1 mmol), **4a** (0.1 mmol), ENE-101 (25 mg, 65.8 U/mg, 1.2 mol‰), GDH-101 (12.5 mg, 32.0 U/mg), D-glucose (0.5 mmol), $NADP^+$ (5 µmol), 1.9 mL KPBS (250 mM, pH 7.0), DMSO (100 µL), 30 °C, 24 h. [b]Determined by chiral HPLC analysis. [c]Isolated yield. [d]The reaction was carried out for 48 h. [e]**4a** (0.12 mmol) was used. [f]The reaction was performed at 37 °C for both steps. [g]**3a** (0.1 mmol), **4a** (0.1 mmol), ENE-101 (50 mg, 65.8 U/mg, 2.5 mol‰), GDH-101 (25 mg, 32.0 U/mg), D-glucose (1 mmol), $NADP^+$ (10 µmol), 3.8 mL KPBS (250 mM, pH 7.0), DMSO (200 µL), 30 °C, 24 h. [h] NAD^+ was used instead of $NADP^+$ as the cofactor.

General Procedure for One-Step One-pot Chemoenzymatic Cascade for the Enantioselective Synthesis of (*S*)-2

General Procedure M: To a solution of **3** (0.1 mmol) and **4a** (0.1 mmol) in DMSO (200 µL) in a 10 mL vial were added 3.6 mL KPBS (250 mM, pH 7.0), ENE-101 (50 mg, 65.8 U/mg, 2.5 mol‰), GDH-101 (25 mg, 32.0 U/mg), D-glucose (1 mmol), and a solution of $NADP^+$ (10 µmol) in 200 µL KPBS (250 mM, pH 7.0), and the vial was incubated at 30 °C with shaking at 200 rpm for 24 h. Then the reaction mixture was extracted with EtOAc (2 mL × 5) and centrifuged at 25 °C and 8000 rpm for 5 min, and the combined organic phase was dried with $MgSO_4$. An aliquot of the organic phase was taken for HPLC analysis to determine the conversions and ee values. Then the organic solvent was removed *in vacuo*, and the residue obtained was purified by column chromatography on silica gel to give the desired products **2**.

Supplementary Table 8. One-step one-pot chemoenzymatic cascade for the enantioselective synthesis of (*S*)-**2**.[a]

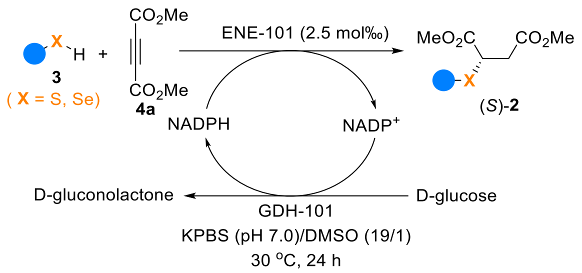

Substrate	Product	Conv. (%)[b]	Product Mass (mg), Yield (%)[c]	ee (%)[b]
Thiophenol	**2aa**	73	14.2 mg, 56%	92 (*S*)
2-Fluorothiophenol	**2ab**	92	21.7 mg, 80%	95 (*S*)
2-Chlorothiophenol	**2ac**	93	23.3 mg, 81%	95 (*S*)
4-Chlorothiophenol	**2ad**	74	11.8 mg, 41%	86 (*S*)
4-Methylthiophenol	**2ag**	96	21.6 mg, 81%	95 (*S*)
3-Methoxythiophenol	**2ah**	96	23.2 mg, 82%	90 (*S*)
4-Methoxythiophenol	**2ai**	97	23.5 mg, 83%	91 (*S*)
3,5-Dimethylthiophenol	**2aj**	94	24.0 mg, 85%	94 (*S*)
1-Naphthalenethiol	**2ak**	82	15.4 mg, 51%	99 (*S*)
2-Naphthalenethiol	**2al**	94	18.8 mg, 62%	95 (*S*)
Benzyl mercaptan	**2an**	18	/	88 (*S*)
Phenylselenol	**2bd**	38	/	95 (*S*)

[a]Reaction conditions: **3** (0.1 mmol), **4a** (0.1 mmol), ENE-101 (50 mg, 65.8 U/mg, 2.5 mol‰), GDH-101 (25 mg, 32.0 U/mg), D-glucose (1 mmol), $NADP^+$ (10 μmol), 3.8 mL KPBS (250 mM, pH 7.0), DMSO (200 μL), 30 °C, 24 h. [b]Determined by chiral HPLC analysis. [c]Isolated yield.

Reaction Condition Optimization for ENE Biocatalysed Enantioselective Reduction of 5aa

Supplementary Table 9. Reaction condition optimization for ENE biocatalysed enantioselective reduction of **5aa**.[a] (The 7 ENE reductases from Johnson Matthey were screened on compound **5aa**. In addtion, three ENE reductases availble at UCL were also screened to compare the enantioselctivity.)

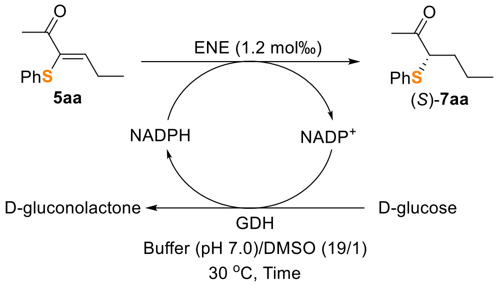

Entry	ENE	GDH	Buffer	Time (h)	Conv. of 7aa (%)[b]	ee of 7aa (%)[b]
1	ENE-101	GDH-101	KPBS (pH 7.0)	6	>99 (90[e])	72 (72[f]) (*S*)
2	ENE-101	GDH-101	KPBS (pH 7.0)	3	>99	83 (*S*)
3	ENE-101	GDH-101	KPBS (pH 7.0)	2	>99	89 (*S*)
4	ENE-101	GDH-101	KPBS (pH 7.0)	1.5	>99 (91[e])	92 (90[f]) (*S*)
5	ENE-101	GDH-101	KPBS (pH 7.0)	1	94	93 (*S*)
6	ENE-102	GDH-101	KPBS (pH 7.0)	1.5	4	35 (*S*)
7	ENE-103	GDH-101	KPBS (pH 7.0)	1.5	85	72 (*S*)
8	ENE-105	GDH-101	KPBS (pH 7.0)	1.5	96	17 (*R*)
9	ENE-107	GDH-101	KPBS (pH 7.0)	1.5	96	89 (*S*)
10	ENE-108	GDH-101	KPBS (pH 7.0)	1.5	83	77 (*S*)
11	ENE-109	GDH-101	KPBS (pH 7.0)	1.5	69	65 (*S*)
12	NCR	GDH-101	KPBS (pH 7.0)	1.5	91	84 (*S*)
13	pQR1440	GDH-101	KPBS (pH 7.0)	1.5	10	56 (*S*)
14	pQR1907	GDH-101	KPBS (pH 7.0)	1.5	87	88 (*S*)
15	ENE-101	GDH-5	KPBS (pH 7.0)	1.5	91	91 (*S*)
16	ENE-101	GDH-8	KPBS (pH 7.0)	1.5	94	90 (*S*)
17[c]	ENE-101	GDH-101	KPBS (pH 7.0)	1.5	99	91 (*S*)
18[d]	ENE-101	GDH-101	KPBS (pH 7.0)	1.5	>99	85 (*S*)
19	ENE-101	GDH-101	KPBS (pH 6.0)	1.5	91	92 (*S*)
20	ENE-101	GDH-101	KPBS (pH 6.5)	1.5	95	91 (*S*)
21	ENE-101	GDH-101	KPBS (pH 7.5)	1.5	92	89 (*S*)
22	ENE-101	GDH-101	KPBS (pH 8.0)	1.5	86	89 (*S*)
23	ENE-101	GDH-101	KPBS (pH 8.5)	1.5	83	89 (*S*)
24	ENE-101	GDH-101	Tris-HCl (pH 7.0)	1.5	98	92 (*S*)
25	ENE-101	GDH-101	Tris-HCl (pH 7.5)	1.5	99	92 (*S*)
26	ENE-101	GDH-101	Tris-HCl (pH 8.0)	1.5	>99	92 (*S*)
27	ENE-101	GDH-101	Tris-HCl (pH 8.5)	1.5	>99	90 (*S*)

[a]Reaction conditions: **5aa** (0.02 mmol), ENE-101~ENE-109 (5.0 mg, 1.2 mol‰) or NCR (0.5 mg) or pQR1440 (0.5 mg) or pQR1907 (0.5 mg), GDH (2.5 mg), D-glucose (0.1 mmol), $NADP^+$ (1 μmol), 950 μL KPBS (250 mM, pH 6.0~8.5) or Tris-HCl (50 mM, pH 7.0~8.5), DMSO (50 μL), 30 °C, 1-6 h. [b]Determined by chiral HPLC analysis. [c]NAD^+ was used instead of $NADP^+$ as the cofactor. [d]The reaction was performed at 37 °C. [e]Isolated yield of **7aa** at 0.1 mmol **5aa**. [f]The ee value of **7aa** at 0.1 mmol **5aa**. ENE-101 (65.8 U/mg), ENE-102 (5.4 U/mg), ENE-103 (3.0 U/mg), ENE-105 (9.0 U/mg), ENE-107 (101.0 U/mg), ENE-108 (6.0 U/mg), ENE-109 (6.1 U/mg), GDH-101 (32.0 U/mg), GDH-5 (51.2 U/mg), GDH-8 (1.6 U/mg).

Investigation of the Configurational Stability of (*S*)-7aa

Supplementary Table 10. Investigation of the configurational stability of (*S*)-**7aa**.

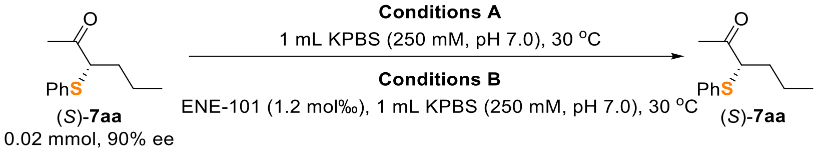

Time	0 h	2 h	4 h	6 h	21 h	30 h	49 h	76 h	78 h
ee of (*S*)-**7aa** (Conditions A)	90%	89%	89%	88%	79%	76%	67%	56%	56%
ee of (*S*)-**7aa** (Conditions B)	90%	71%	61%	57%	36%	28%	17%	9%	8%

Conditions A: 1 mL KPBS (250 mM, pH 7.0) was added to 0.02 mmol (*S*)-**7aa** (90% ee), and the resulting mixture was incubated at 30 °C with shaking at 200 rpm. The ee value of (*S*)-**7aa** was monitored at different time points by taking 20 µL aliquots, which were extracted with EtOAc (20 µL× 3) and then used for chiral HPLC analysis. Conditions B: 1 mL KPBS (250 mM, pH 7.0) and ENE-101 (5.0 mg, 65.8 U/mg, 1.2 mol‰) were added sequentially to 0.02 mmol (*S*)-**7aa** (90% ee), and the resulting mixture was incubated at 30 °C with shaking at 200 rpm. The ee value of (*S*)-**7aa** was monitored at different time points by taking 20 µL aliquots, which were extracted with EtOAc (20 µL× 3) and then used for chiral HPLC analysis.

General Procedure for ENE-101 Biocatalysed Enantioselective Reduction of 5 or 6 into (*S*)-7 or 8

General Procedure N: To a solution of ENE-101 (25 mg, 65.8 U/mg, 1.2 mol‰), GDH-101 (12.5 mg, 32.0 U/mg) and D-glucose (0.5 mmol) in 1.7 mL KPBS (250 mM, pH 7.0) in a 10 mL vial were added a solution of $NADP^+$ (5 µmol) in 200 µL KPBS (250 mM, pH 7.0) and a solution of **5** or **6** (0.1 mmol) in DMSO (100 µL), then the vial was incubated at 30 °C with shaking at 200 rpm for 1-24 h. After that, the reaction mixture was extracted with EtOAc (2 mL × 5) and centrifuged at 25 °C and 8000 rpm for 5 min, and the combined organic phase was dried with $MgSO_4$. An aliquot of the organic phase was taken for HPLC analysis to determine the conversions and ee values. Then the organic solvent was removed *in vacuo*, and the residue obtained was purified by column chromatography on silica gel to give the desired products **7** or **8**.

Supplementary Table 11. ENE-101 biocatalysed enantioselective reduction of **5** or **6** into (*S*)-**7** or **8**.[a]

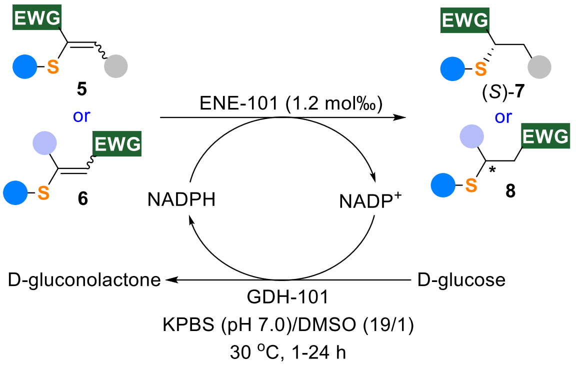

Substrate	Product	Conv. (%)[b]	Product Mass (mg), Yield (%)[c]	ee (%)[b]
(*Z*)-**5aa** (1.5 h)	**7aa**	>99	18.9 mg, 91%	90 (*S*)
(*Z*)-**5aa** (6 h)	**7aa**	>99	18.7 mg, 90%	72 (*S*)
(*Z*)-**5ab** (2 h)	**7ab**	87	17.1 mg, 76%	92 (*S*)
(*Z*)-**5ac** (2 h)	**7ac**	>99	19.9 mg, 82%	>99 (*S*)
(*Z*)-**5ad** (3 h)	**7ad**	>99	22.3 mg, 92%	90 (*S*)
(*Z*)-**5ae** (2 h)	**7ae**	>99	24.3 mg, 85%	95 (*S*)
(*Z*)-**5af** (2 h)	**7af**	88	20.6 mg, 72%	94 (*S*)
(*Z*)-**5ag** (5 h)	**7ag**	63	11.7 mg, 46%	85 (*S*)
(*Z*)-**5ag** (19 h)	**7ag**	98	22.8 mg, 89%	79 (*S*)
(*E*)-**5ag** (5 h)	**7ag**	68	12.2 mg, 48%	87 (*S*)
(*E*)-**5ag** (19 h)	**7ag**	93	20.8 mg, 81%	79 (*S*)
(*Z*)-**5ah** (1.5 h)	**7ah**	20	/	87 (*S*)
(*Z*)-**5ah** (23 h)	**7ah**	79	17.7 mg, 61%	76 (*S*)
(*E*)-**5ah** (1.5 h)	**7ah**	19	/	88 (*S*)
(*E*)-**5ah** (23 h)	**7ah**	52	8.7 mg, 30%	70 (*S*)
(*Z*)-**5ai** (30 h)	**7ai**	0	/	/
(*Z*)-**5aj** (1 h)	**7aj**	>99	16.1 mg, 83%	18 (*S*)
(*Z*)-**5aj** (2 h)	**7aj**	>99	16.5 mg, 85%	12 (*S*)
(*Z*)-**5ak**/(*E*)-**5ak** = 0.4/1 (24 h)	**7ak**	0	/	/
(*Z*)-**5al**/(*E*)-**5al** = 1/1 (24 h)	**7al**	>99	17.6 mg, 92%	98 (*S*)
(*Z*)-**5am** (24 h)	**7am**	>99	18.4 mg, 88%	>99 (*S*)
(*Z*)-**6aa** (24 h)	**8aa**	0	/	/
(*E*)-**6aa** (24 h)	**8aa**	15	/	75
(*Z*)-**6ab** (24 h)	**8ab**	0	/	/
(*E*)-**6ab** (24 h)	**8ab**	0	/	/

[a]Reaction conditions: **5** or **6** (0.1 mmol), ENE-101 (25 mg, 65.8 U/mg, 1.2 mol‰), GDH-101 (12.5 mg, 32.0 U/mg), D-glucose (0.5 mmol), $NADP^+$ (5 μmol), 1.9 mL KPBS (250 mM, pH 7.0), DMSO (100 μL), 30 °C, 1-24 h. [b]Determined by chiral HPLC analysis. [c]Isolated yield.

The time course experiment on ENE-101 biocatalysed reduction of 5aj

Supplementary Table 12. The time course experiment on ENE-101 biocatalysed reduction of **5aj**.[a]

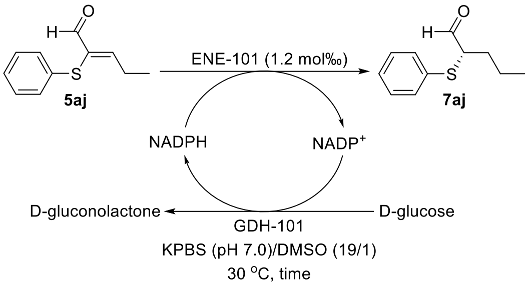

Entry	Time	Conv. of 7aj (%)[b]	ee of 7aj (%)[b]	Yield of 7aj (%)[c]
1	10 min	28	37 (*S*)	/
2	20 min	71	31 (*S*)	/
3	30 min	76	26 (*S*)	/
4	40 min	94	20 (*S*)	/
5	1 h	>99	18 (*S*)	83
6	2 h	>99	12 (*S*)	85
7	4 h	>99	0	84

[a]Reaction conditions: **5aj** (0.1 mmol), ENE-101 (25 mg, 65.8 U/mg, 1.2 mol‰), GDH-101 (12.5 mg, 32.0 U/mg), D-glucose (0.5 mmol), $NADP^+$ (5 μmol), 1.9 mL KPBS (250 mM, pH 7.0), DMSO (100 μL), 30 °C, 10 min-3 h. [b]Determined by chiral HPLC analysis. [c]Isolated yield.

Screening of ADHs for the Oxidation of 9aa into 5aa

Supplementary Table 13. Screening of ADHs for the oxidation of **9aa** into **5aa**.[a]

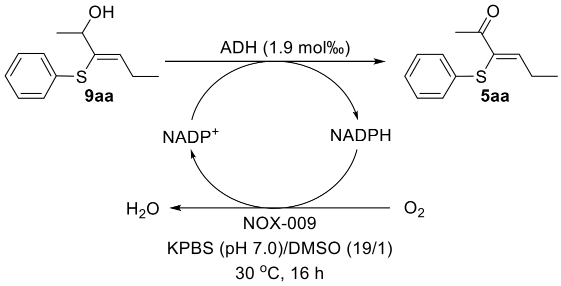

Entry	ADH	Conv. (%)[b]	Entry	ADH	Conv. (%)[b]
1	ADH-19	37	8	ADH-153	38
2	ADH-20	11	9	ADH-159	68
3	ADH-61	35	10	ADH-160	7
4	ADH-62	35	11	ADH-171	1
5	ADH-101	31	12	ADH-220	28
6	ADH-110	29	13	ADH-230	6
7	ADH-150	37	14	ADH-244	20

[a]Reaction conditions: **9aa** (0.02 mmol), ADH (5 mg, 1.9 mol‰), NOX-009 (5 mg), $NADP^+$ (1 μmol), air, 0.95 mL KPBS (250 mM, pH 7.0), DMSO (50 μL), 30 °C, 200 rpm, 16 h. [b]Determined by HPLC analysis. ADH-19 (2.8 U/mg), ADH-20 (1.0 U/mg), ADH-61 (1.9 U/mg), ADH-62 (3.2 U/mg), ADH-101 (27.4 U/mg), ADH-110 (8.5 U/mg), ADH-150 (4.1 U/mg), ADH-153 (3.3 U/mg), ADH-159 (0.2 U/mg), ADH-160 (0.5 U/mg), ADH-171 (10.9 U/mg), ADH-220 (1.2 U/mg), ADH-230 (0.2 U/mg), ADH-244 (2.1 U/mg).

General Procedure for One-Step One-Pot Hydrogen-Borrowing Cascade for the Enantioselective Synthesis of (*S*)-7 from 9

General Procedure O: To a solution of ENE-101 (5 mg, 65.8 U/mg, 1.2 mol‰) and ADH-159 (5 mg, 0.2 U/mg, 1.9 mol‰) in 0.85 mL KPBS (250 mM, pH 7.0) in a 2 mL vial were added a solution of $NADP^+$ (1 μmol) in 100 μL KPBS (250 mM, pH 7.0) and a solution of **9** (0.02 mmol) in DMSO (50 μL), then the vial was incubated at 30 °C with shaking at 200 rpm for 1.5 or 12 h. After that, the reaction mixture was extracted with EtOAc (0.5 mL × 5) and centrifuged at 25 °C and 8000 rpm for 5 min, and the combined organic phase was dried with $MgSO_4$. An aliquot of the organic phase was taken for HPLC analysis to determine the conversions and ee values.

Supplementary Table 14. One-step one-pot hydrogen-borrowing cascade for the enantioselective synthesis of (*S*)-**7** from **9**.[a]

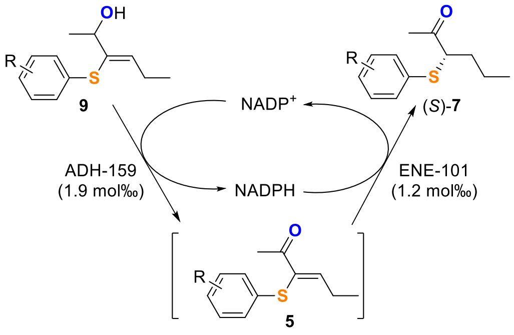

R	t (h)	Product	Conv. (%)[b]	ee (%)[b]
H	1.5	**7aa**	9	88 (*S*)
H	12	**7aa**	38	74 (*S*)
2-Br	12	**7ae**	13	75 (*S*)

[a]Reaction conditions: **9** (0.02 mmol), ENE-101 (5 mg, 65.8 U/mg, 1.2 mol‰), ADH-159 (5 mg, 0.2 U/mg, 1.9 mol‰), $NADP^+$ (1 μmol), 0.95 mL KPBS (250 mM, pH 7.0), DMSO (50 μL), 30 °C, 1.5 or 12 h. [b]Determined by chiral HPLC analysis.

Screening of ADHs for the Reduction of 7ad into 9ad

Supplementary Table 15. Screening of ADHs for the reduction of **7ad** into **9ad**.[a]

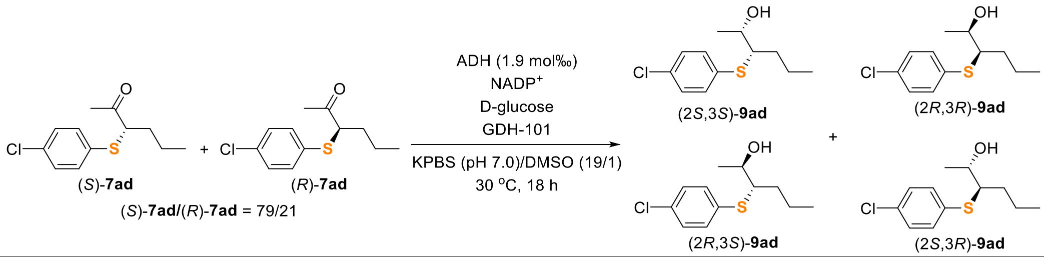

Entry	ADH	Conv. (%)[b]	*dr (syn/anti)*[b]	*ee syn (%)*[b]	*ee anti (%)*[b]
1	ADH-19	96	31/69	57 (*2R,3R*)	>99 *(2R,3S)*
2	ADH-20	98	34/66	49 *(2R, 3R)*	>99 *(2R,3S)*
3	ADH-27	98	81/19	>99 (*2S,3S*)	>99 *(2S,3R)*
4	ADH-61	17	75/25	>99 (*2R,3R*)	>99 *(2R,3S)*
5	ADH-62	33	10/90	>99 (*2R,3R*)	>99 *(2R,3S)*
6	ADH-101	9	25/75	>99 (*2S,3S*)	>99 *(2R,3S)*
7	ADH-105	38	27/73	>99 (*2S,3S*)	>99 *(2S,3R)*
8	ADH-110	5	/	/	/
9	ADH-150	91	95/5	69 (*2S,3S*)	>99 *(2S,3R)*
10	ADH-153	82	100/0	>99 (*2S,3S*)	/
11	ADH-159	>99	96/4	66 (*2S,3S*)	>99 *(2S,3R)*
12	ADH-220	5	/	/	/
13	ADH-244	77	73/27	>99 (*2S,3S*)	81 *(2S,3R)*

[a]Reaction conditions: **7ad** (58% ee (*S*), 0.01 mmol), ADH (2.5 mg, 1.9 mol‰), GDH-101 (1.25 mg, 32.0 U/mg), D-glucose (0.05 mmol), NADP+ (0.5 μmol), 950 μL KPBS (250 mM, pH 7.0), DMSO (50 μL), 30 °C, 18 h. [b]Determined by chiral HPLC analysis. ADH-19 (2.8 U/mg), ADH-20 (1.0 U/mg), ADH-27 (1.6 U/mg), ADH-61 (1.9 U/mg), ADH-62 (3.2 U/mg), ADH-101 (27.4 U/mg), ADH-105 (4.9 U/mg), ADH-110 (8.5 U/mg), ADH-150 (4.1 U/mg), ADH-153 (3.3 U/mg), ADH-159 (0.2 U/mg), ADH-220 (1.2 U/mg), ADH-244 (2.1 U/mg).

General Procedure for Two-Step One-Pot Biocatalytic-Chemical (Bio-Chem) or Biocatalytic-Biocatalytic (Bio-Bio) Cascade for the Enantioselective Synthesis of 10 from 5

General Procedure P: To a solution of ENE-101 (25 mg, 65.8 U/mg, 1.2 mol‰), GDH-101 (12.5 mg, 32.0 U/mg) and D-glucose (0.5 mmol) in 1.7 mL KPBS (250 mM, pH 7.0) in a 10 mL vial were added a solution of NADP+ (5 μmol) in 200 μL KPBS (250 mM, pH 7.0) and a solution of **5** (0.1 mmol) in DMSO (100 μL), then the vial was incubated at 30 °C with shaking at 200 rpm for 1.5, 2 or 3 h. Subsequently, the reaction was added with either a) MeOH (1 mL) and $NaBH_4$ (0.5 mmol) with stirring at room temperature for 1 h, or b) ADH-153 (50 mg, 3.3 U/mg, 3.8 mol‰) or ADH-19 (50 mg, 2.8 U/mg, 3.8 mol‰) with incubation at 30 °C and shaking at 200 rpm for 18 h. After that, the reaction mixture was extracted with EtOAc (2 mL × 5) and centrifuged at 25 °C and 8000 rpm for 5 min, and the combined organic phase was dried with $MgSO_4$. An aliquot of the organic phase was taken for HPLC analysis to determine the conversions, dr and ee values. Then the organic solvent was removed *in vacuo*, and the residue obtained was purified by column chromatography on silica gel to give the desired products **10**.

Supplementary Table 16. Two-step one-pot bio-chem or bio-bio cascade for the enantioselective synthesis of **10** from **5**.[a]

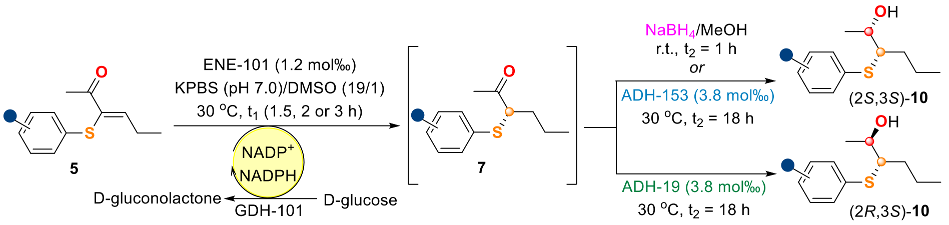

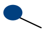	t_1 (h)	Reducing agent	Product	Conv. (%)[b]	Yield (%)[c]	*dr* (*syn*/*anti*)[b]	ee *syn* (%)[b]	ee *anti* (%)[b]
		$NaBH_4$	**10aa**	94	16.8 mg, 80%	81/19	>99 (2*S*,3*S*)	/
H	1.5	ADH-153	**10aa**	97	17.8 mg, 85%	100/0	>99 (2*S*,3*S*)	/
		ADH-19	**10aa**	96	17.0 mg, 81%	27/73	>99 (2*S*,3*S*)	/
2-F	2	ADH-153	**10ab**	98	18.7 mg, 82%	100/0	>99 (2*S*,3*S*)	/
		ADH-19	**10ab**	97	19.2 mg, 84%	42/58	86 (2*S*,3*S*)	>99 (2*R*,3*S*)
2-Cl	2	ADH-153	**10ac**	91	19.3 mg, 79%	100/0	>99 (2*S*,3*S*)	/
		ADH-19	**10ac**	97	20.2 mg, 83%	55/45	80 (2*S*,3*S*)	95 (2*R*,3*S*)
		$NaBH_4$	**10ad**	99	21.1 mg, 86%	81/19	89 (2*S*,3*S*)	87 (2*R*,3*S*)
4-Cl	3	ADH-153	**10ad**	89	18.3 mg, 75%	100/0	>99 (2*S*,3*S*)	/
		ADH-19	**10ad**	>99	22.0 mg, 90%	11/89	20 (2*S*,3*S*)	>99 (2*R*,3*S*)
		$NaBH_4$	**10ae**	90	21.3 mg, 74%	80/20	84 (2*S*,3*S*)	87 (2*R*,3*S*)
2-Br	2	ADH-153	**10ae**	92	21.9 mg, 76%	100/0	>99 (2*S*,3*S*)	/
		ADH-19	**10ae**	98	25.4 mg, 88%	58/42	85 (2*S*,3*S*)	98 (2*R*,3*S*)

[a]Reaction conditions: **5** (0.1 mmol), ENE-101 (25 mg, 65.8 U/mg, 1.2 mol‰), GDH-101 (12.5 mg, 32.0 U/mg), D-glucose (0.5 mmol), $NADP^+$ (5 μmol), 1.9 mL KPBS (250 mM, pH 7.0), DMSO (100 μL), 30 °C, 1.5, 2 or 3 h; then MeOH (1 mL), $NaBH_4$ (0.5 mmol), room temperature, 1 h; or ADH-153 (50 mg, 3.3 U/mg, 3.8 mol‰), 30 °C, 18 h; or ADH-19 (50 mg, 2.8 U/mg, 3.8 mol‰), 30 °C, 18 h. [b]Determined by chiral HPLC analysis. [c]Isolated yield.

General Procedure for One-Step One-Pot Biocatalytic-Biocatalytic (Bio-Bio) Cascade for the Enantioselective Synthesis of 10 from 5

General Procedure Q: To a solution of ENE-101 (25 mg, 65.8 U/mg, 1.2 mol‰), ADH-153 (50 mg, 3.3 U/mg, 3.8 mol‰) or ADH-19 (50 mg, 2.8 U/mg, 3.8 mol‰), GDH-101 (12.5 mg, 32.0 U/mg) and D-glucose (0.5 mmol) in 1.7 mL KPBS (250 mM, pH 7.0) in a 10 mL vial were added a solution of $NADP^+$ (5 μmol) in 200 μL KPBS (250 mM, pH 7.0) and a solution of **5** (0.1 mmol) in DMSO (100 μL), then the vial was incubated at 30 °C with shaking at 200 rpm for 18 h. After that, the reaction mixture was extracted with EtOAc (2 mL × 5) and centrifuged at 25 °C and 8000 rpm for 5 min, and the combined organic phase was dried with $MgSO_4$. An aliquot of the organic phase was taken for HPLC analysis to determine the conversions, dr and ee values. Then the organic solvent was removed *in vacuo*, and the residue obtained was purified by column chromatography on silica gel to give the desired products **10**.

Supplementary Table 17. One-step one-pot bio-bio cascade for the enantioselective synthesis of **10** from **5**.[a]

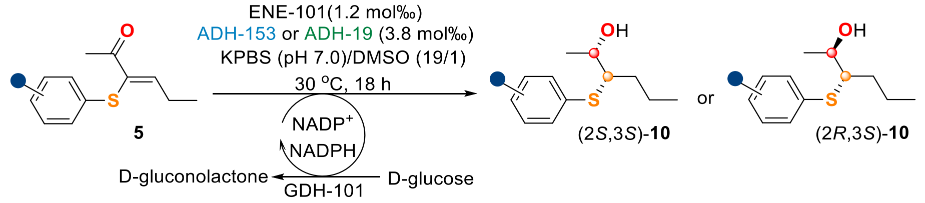

	ADH	Product	Conv. (%)[b]	Yield (%)[c]	*dr* (*syn*/*anti*)[b]	ee *syn* (%)[b]	ee *anti* (%)[b]
H	ADH-153	**10aa**	58	7.9 mg, 38%	100/0	>99 (2*S*,3*S*)	/
2-F	ADH-153	**10ab**	80	13.9 mg, 61%	98:2	93 (2*S*,3*S*)	/
2-F	ADH-19	**10ab**	95	18.8 mg, 82%	52/48	14 (2*R*,3*R*)	>99 (2*R*,3*S*)
2-Cl	ADH-153	**10ac**	97	20.4 mg, 83%	100/0	>99 (2*S*,3*S*)	/
2-Cl	ADH-19	**10ac**	94	18.3 mg, 75%	57/43	44 (2*S*,3*S*)	>99 (2*R*,3*S*)
4-Cl	ADH-153	**10ad**	88	17.7 mg, 72%	100/0	>99 (2*S*,3*S*)	/
4-Cl	ADH-19	**10ad**	77	13.7 mg, 56%	25/75	49 (2*R*,3*R*)	>99 (2*R*,3*S*)
2-Br	ADH-153	**10ae**	95	22.8 mg, 79%	100/0	>99 (2*S*,3*S*)	/

[a]Reaction conditions: **5** (0.1 mmol), ENE-101 (25 mg, 65.8 U/mg, 1.2 mol‰), ADH-153 (50 mg, 3.3 U/mg, 3.8 mol‰) or ADH-19 (50 mg, 2.8 U/mg, 3.8 mol‰), GDH-101 (12.5 mg, 32.0 U/mg), D-glucose (0.5 mmol), $NADP^+$ (5 μmol), 1.9 mL KPBS (250 mM, pH 7.0), DMSO (100 μL), 30 °C, 18 h. [b]Determined by chiral HPLC analysis. [c]Isolated yield.

General Procedure for Three-Step One-Pot Biocatalytic-Biocatalytic-Biocatalytic (Bio-Bio-Bio) Cascade for the Enantioselective Synthesis of 10 from 9

General Procedure R: To a solution of ADH-159 (25 mg, 0.2 U/mg, 1.9 mol‰) or ADH-153 (25 mg, 3.3 U/mg, 1.9 mol‰) or ADH-19 (25 mg, 2.8 U/mg, 1.9 mol‰) and NOX-009 (25 mg) in 3.6 mL KPBS (250 mM, pH 7.0) in a 10 mL vial were added a solution of $NADP^+$ (5 μmol) in 200 μL KPBS (250 mM, pH 7.0) and a solution of **9** (0.1 mmol) in DMSO (200 μL), and the vial was incubated at 30 °C with shaking at 200 rpm for 24 h. Then ENE-101 (25 mg, 65.8 U/mg, 1.2 mol‰), GDH-101 (12.5 mg, 32.0 U/mg) and D-glucose (0.5 mmol) were added sequentially, and the vial was incubated at 30 °C with shaking at 200 rpm for 8 h. Then the reaction mixture was extracted with EtOAc (2 mL × 5) and centrifuged at 25 °C and 8000 rpm for 5 min, and the combined organic phase was dried with $MgSO_4$. An aliquot of the organic phase was taken for HPLC analysis to determine the conversions and ee values. Then the organic solvent was removed *in vacuo*, and the residue obtained was purified by column chromatography on silica gel to give the desired products **10**.

Supplementary Table 18. Three-step one-pot bio-bio-bio cascade for the enantioselective synthesis of **10** from **9**.[a]

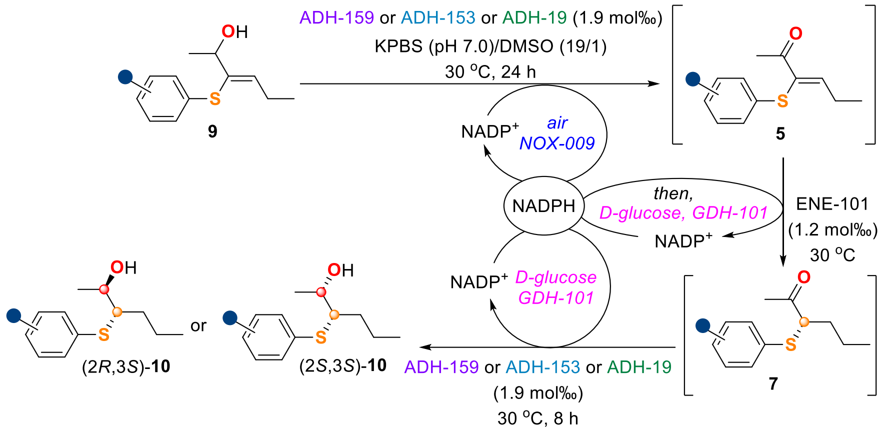

(substituent)	ADH	Product	Conv. (%)[b]	Yield (%)[c]	*dr* (*syn*/*anti*)[b]	ee *syn* (%)[b]	ee *anti* (%)[b]
H	ADH-159	**10aa**	71	9.7 mg, 46%	98/2	68 (2*S*,3*S*)	/
	ADH-153	**10aa**	37	/	100/0	88 (2*S*,3*S*)	/
2-F	ADH-159	**10ab**	68	9.4 mg, 41%	97/3	82 (2*S*,3*S*)	/
	ADH-153	**10ab**	27	/	99/1	97 (2*S*,3*S*)	/
2-Cl	ADH-159	**10ac**	38	/	99/1	85 (2*S*,3*S*)	/
	ADH-19	**10ac**	96	17.6 mg, 72%	70/30	22 (2*R*,3*R*)	>99 (2*R*,3*S*)
4-Cl	ADH-159	**10ad**	70	11.7 mg, 48%	95/5	76 (2*S*,3*S*)	/
	ADH-153	**10ad**	29	/	99/1	99 (2*S*,3*S*)	/
	ADH-19	**10ad**	59	10.1 mg, 41%	39/61	72 (2*R*,3*R*)	89 (2*R*,3*S*)
2-Br	ADH-159	**10ae**	25	/	92/8	>99 (2*S*,3*S*)	/

[a]Reaction conditions: **9** (0.1 mmol), ADH-159 (25 mg, 0.2 U/mg, 1.9 mol‰) or ADH-153 (25 mg, 3.3 U/mg, 1.9 mol‰) or ADH-19 (25 mg, 2.8 U/mg, 1.9 mol‰), NOX-009 (25 mg), $NADP^+$ (5 μmol), 3.8 mL KPBS (250 mM, pH 7.0), DMSO (200 μL), air, 30 °C, 24 h; then ENE-101 (25 mg, 65.8 U/mg, 1.2 mol‰), GDH-101 (12.5 mg, 32.0 U/mg), D-glucose (0.5 mmol), 30 °C, 8 h. [b]Determined by chiral HPLC analysis. [c]Isolated yield.

Deuterium-Labelling Experiments

Deuterium-Labelling Experiments A

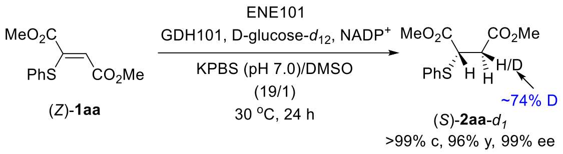

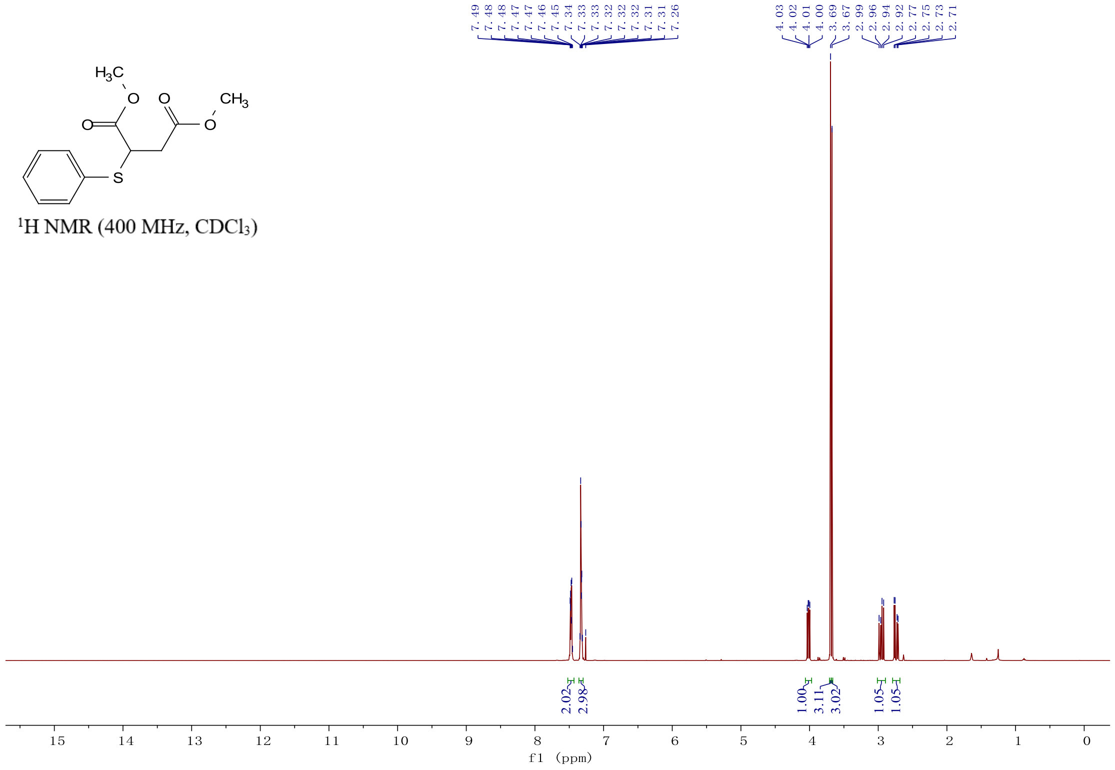

Obtained with D-glucose-d_{12} in KPBS (250 mM, pH 7.0)

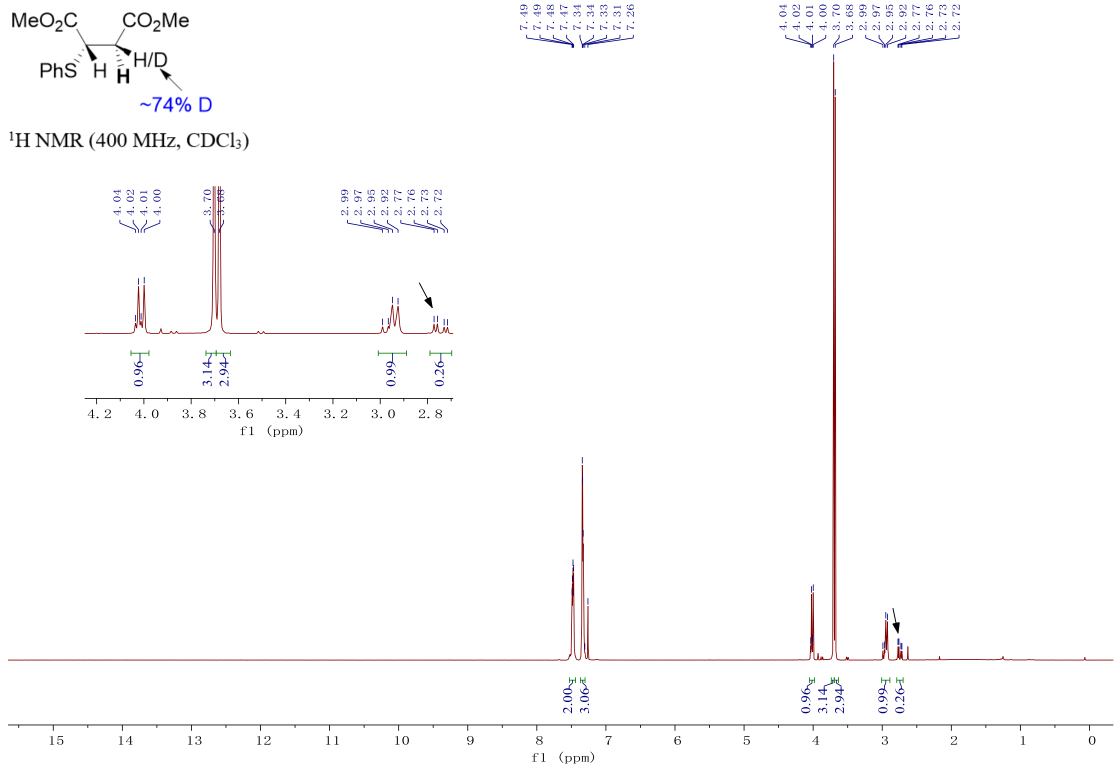

Deuterium-Labelling Experiments B

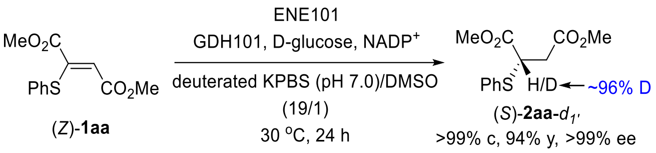

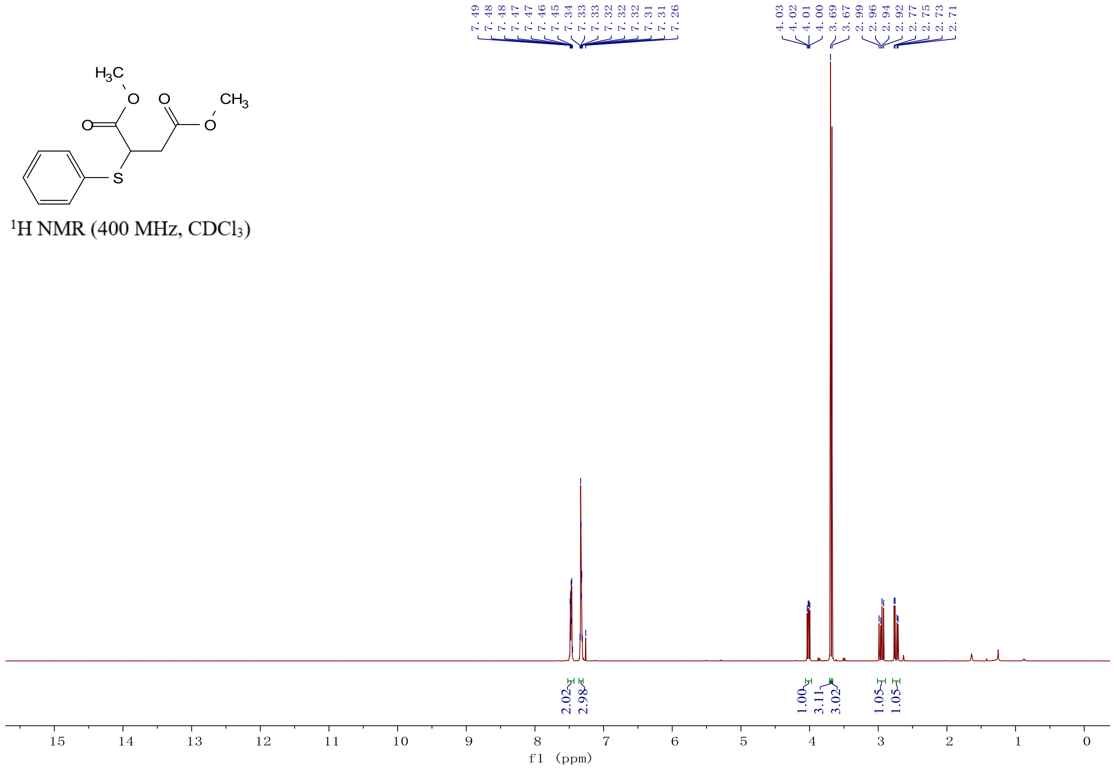

Obtained in deuterated KPBS (250 mM, pH 7.0)

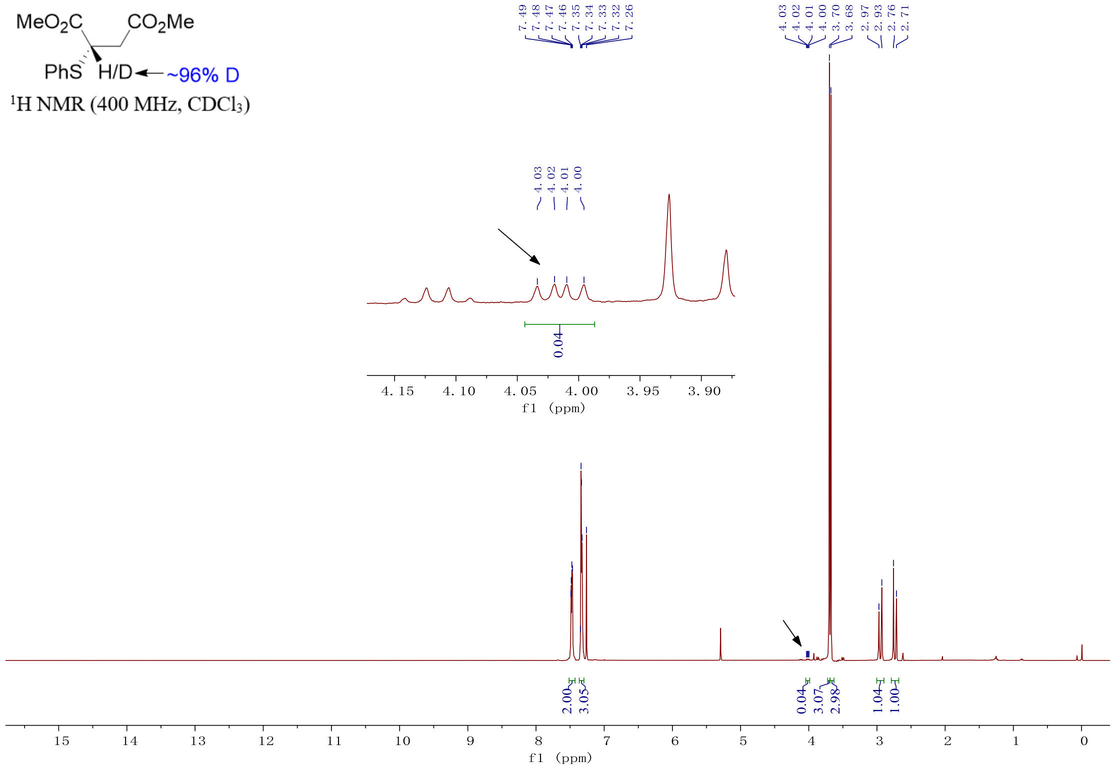

Deuterium-Labelling Experiments C

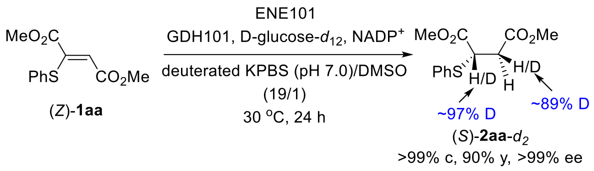

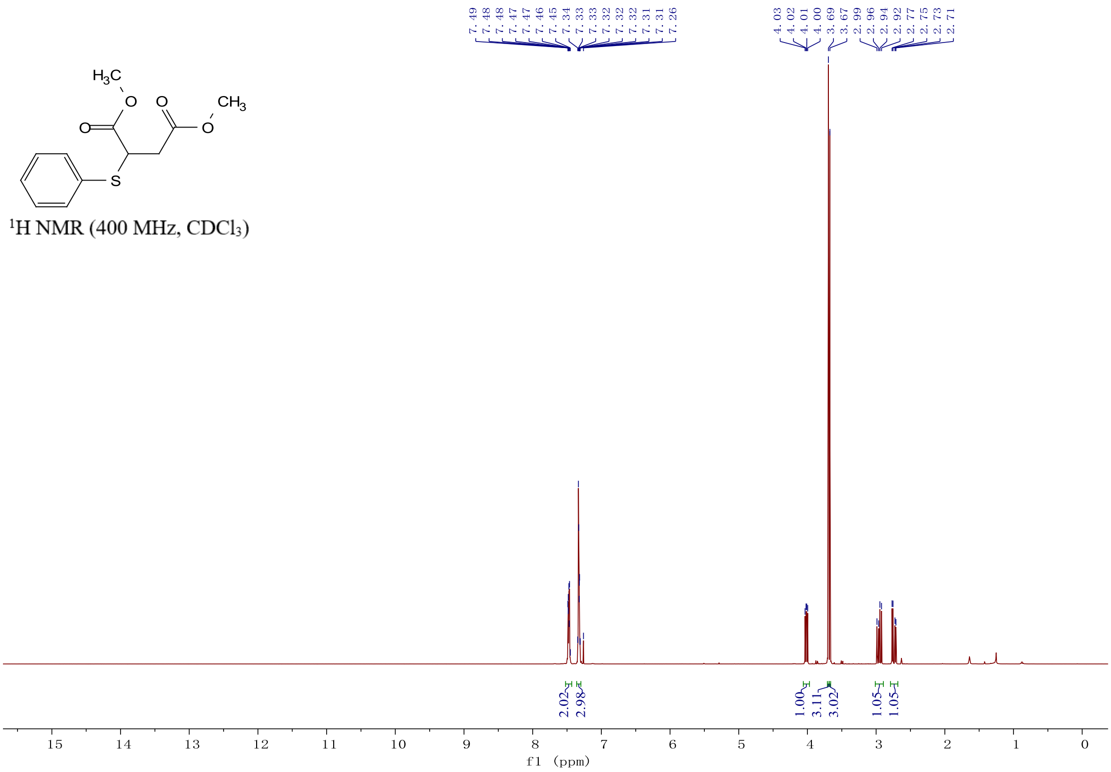

Obtained with D-glucose-d_{12} in deuterated KPBS (250 mM, pH 7.0)

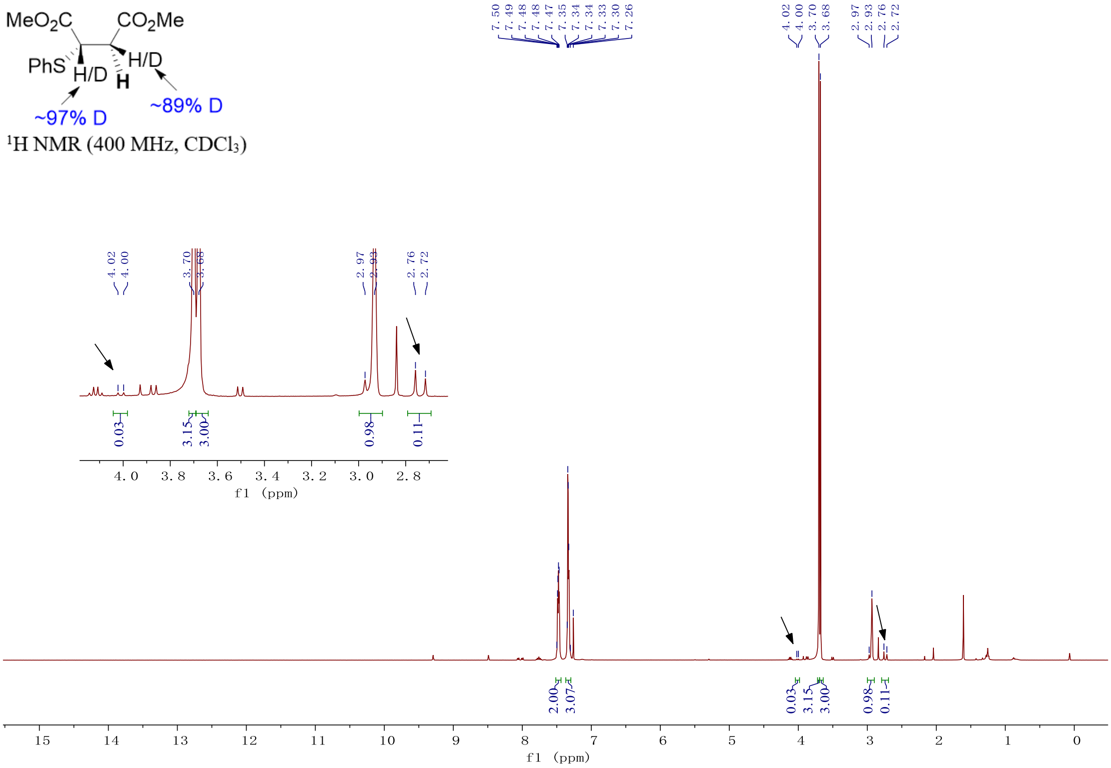

Optical Rotations of Chiral Sulfide Products

The optical rotations of chiral sulfide products **2** and **7** were measured at the concentration of 10 mg/mL in $CHCl_3$ at 25 °C with a Bellingham and Stanley ADP440+ Polarimeter with a cell length of 0.5 dm, and the $[\alpha]_D^{25}$ values of chiral sulfide products **2** and **7** were reported in Supplementary Table 19.

Supplementary Table 19. $[\alpha]_D^{25}$ values of chiral sulfide products **2** and **7**.[a]

Compound	ee (%)	$[\alpha]_D^{25}$	Compound	ee (%)	$[\alpha]_D^{25}$
**2aa**	>99 (*S*)	-127.2	**2av**	>99 (*S*)	ND
**2ab**	>99 (*S*)	-124.8	**2aw**	>99 (*S*)	ND
**2ac**	99 (*S*)	-197.0	**2ax**	69 (*S*)	+42.0
**2ad**	99 (*S*)	-121.6	**2ay**	90 (*S*)	ND
**2ae**	99 (*S*)	-147.6	**2az**	89 (*S*)	ND
**2af**	99 (*S*)	-107.2	**2ba**	>99 (*S*)	ND
**2ag**	99 (*S*)	-120.4	**2bb**	>99 (*S*)	ND
**2ah**	99 (*S*)	-145.4	**2bc**	>99 (*S*)	ND
**2ai**	>99 (*S*)	-131.6	**2bd**	99 (*S*)	-170.2
**2aj**	99 (*S*)	-154.4	**7aa**	90 (*S*)	-120.4
**2ak**	99 (*S*)	-146.6	**7ab**	92 (*S*)	-47.4
**2al**	99 (*S*)	-145.0	**7ac**	>99 (*S*)	-67.2
**2am**	99 (*S*)	-283.6	**7ad**	90 (*S*)	-102.0
**2an**	97 (*S*)	-246.8	**7ae**	95 (*S*)	-57.2
**2ao**	96 (*S*)	-237.0	**7af**	93 (*S*)	-51.8
**2ap**	98 (*S*)	-245.0	**7ag**	79 (*S*)	-41.0
**2aq**	94 (*S*)	-121.4	**7ah**	76 (*S*)	ND
**2ar**	>99 (*S*)	-138.4	**7aj**	18 (*S*)	ND
**2as**	>99 (*S*)	-164.2	**7al**	98 (*S*)	-122.2
**2at**	62 (*S*)	-70.0	**7am**	>99 (*S*)	-122.8
**2au**	99 (*S*)	-134.4	**8aa**	75	ND

[a]All $[\alpha]_D^{25}$ values were recorded in $CHCl_3$, *c* = 10 mg/mL, 25 °C. ND = Not determined.

IV. Computational Study

The homology model of ENE-101 enzymes was built from Glycerol Trinitrate Reductase NerA from *Agrobacterium radiobacter* (PDB ID: 4JIC, 66% sequence identity) using the Homology Modeling tool of the Schrodinger routine (version 2021-1)[13]. The Protein Preparation Wizard tool within the Schrodinger suite was applied to the structure of the enzyme to assign bond orders, add hydrogens, and perform a minimization with the OPLS3e force fields. The structures of the substrates were sketched with the 3D builder routine and submitted to LigPrep (Schrodinger suite) to generate input ligand structures for next calculations. To set up docking simulations, the receptor grid generation routine of the Glide (grid-based ligand docking with energetics) software (version 9.0)[14] was used to codify the shape and properties of the enzymatic binding pocket into a grid in turn used to score the ligand poses. The cofactor structure was used to define the grid, whose inner size was set to 14x14x14 Å to allow substrate structure to find all the possible binding modes within the catalytic pocket. The hydroxyl groups of Ser, Thr, Tyr and the thiol group of Cys were allowed to rotate and adopt different orientations for the most profitable interactions with different ligands.

Extra-precision molecular docking simulations were performed with flexible ligand sampling and post-docking minimization of the resulting substrate/enzyme complexes.[15]
Fukui functions were calculated at the B3LYP-D3/LACVP*+ level of theory by using the Jaguar software (version 11.1)[16] and used to derive Fukui indices.[17,18]

V. Copies of ^{1}H NMR, ^{13}C NMR, ^{19}F NMR and NOESY Spectra

dimethyl 2-(phenylthio)fumarate (*Z*-1aa)

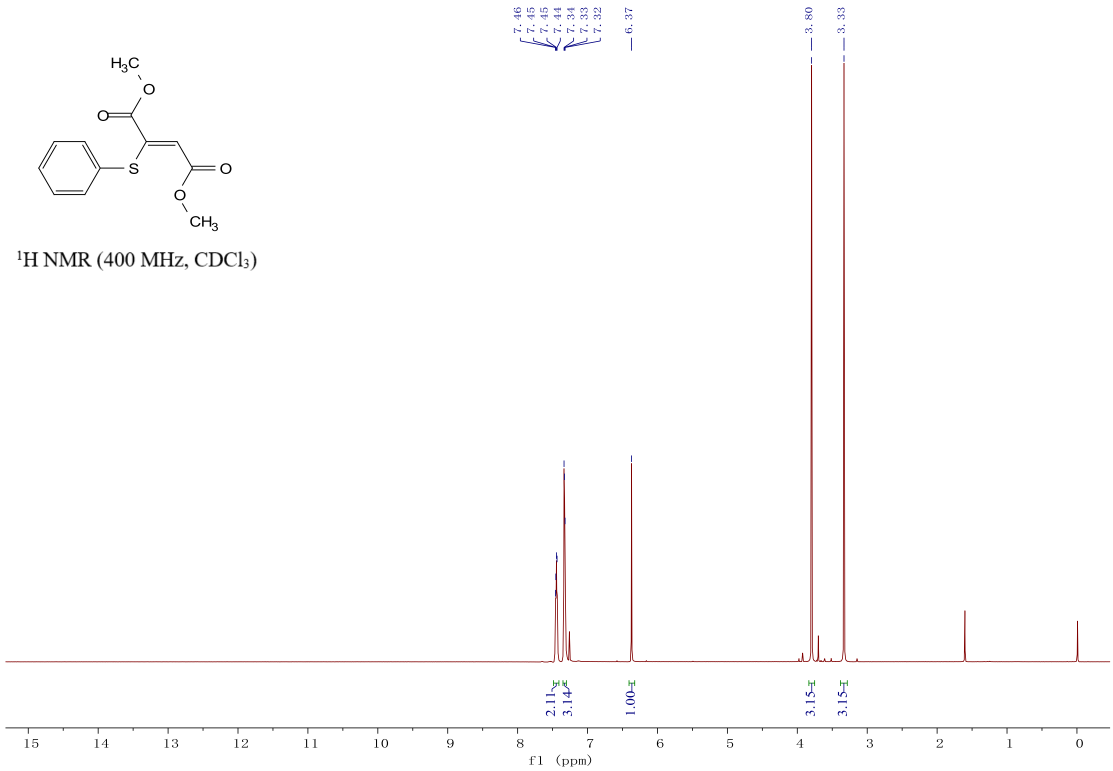

^{1}H NMR (400 MHz, $CDCl_3$)

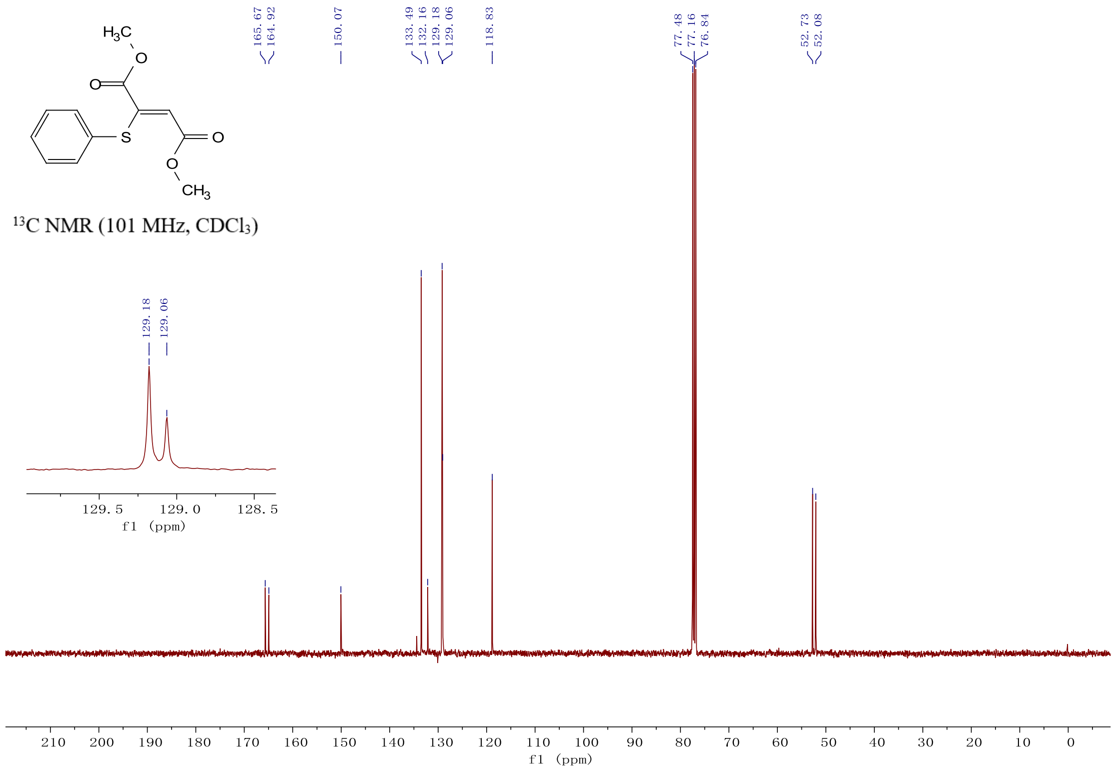

^{13}C NMR (101 MHz, $CDCl_3$)

dimethyl 2-(phenylthio)maleate (*E*-1aa)

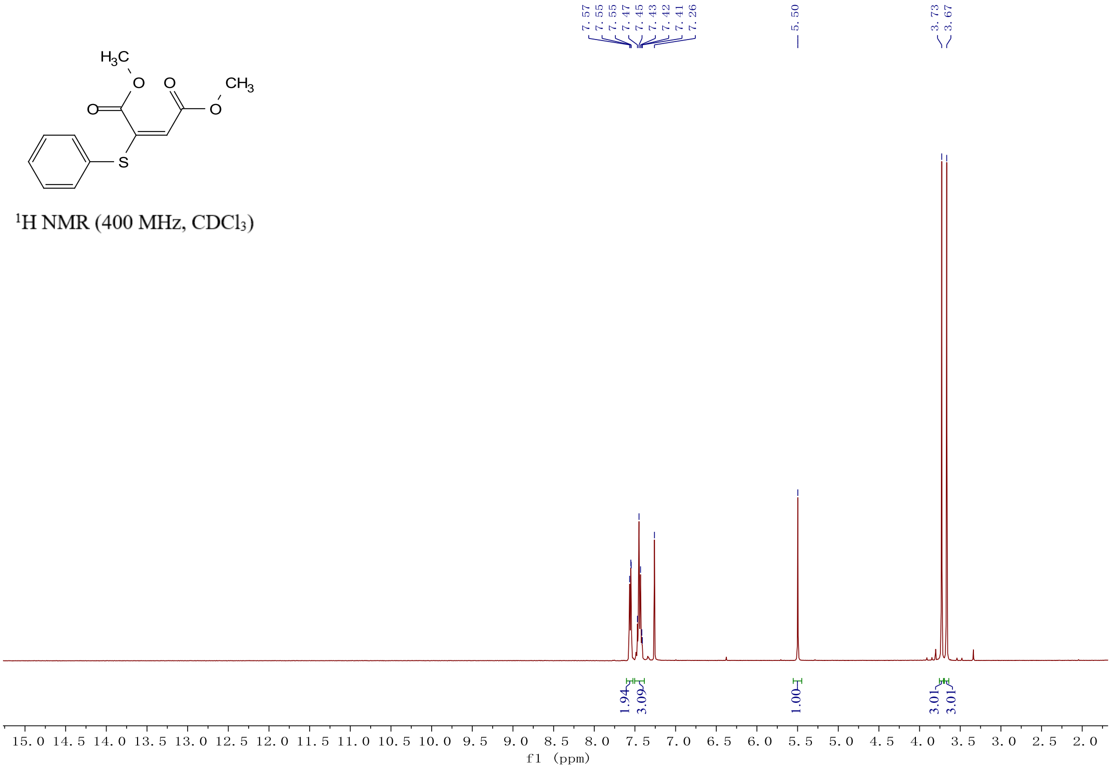

^{1}H NMR (400 MHz, $CDCl_3$)

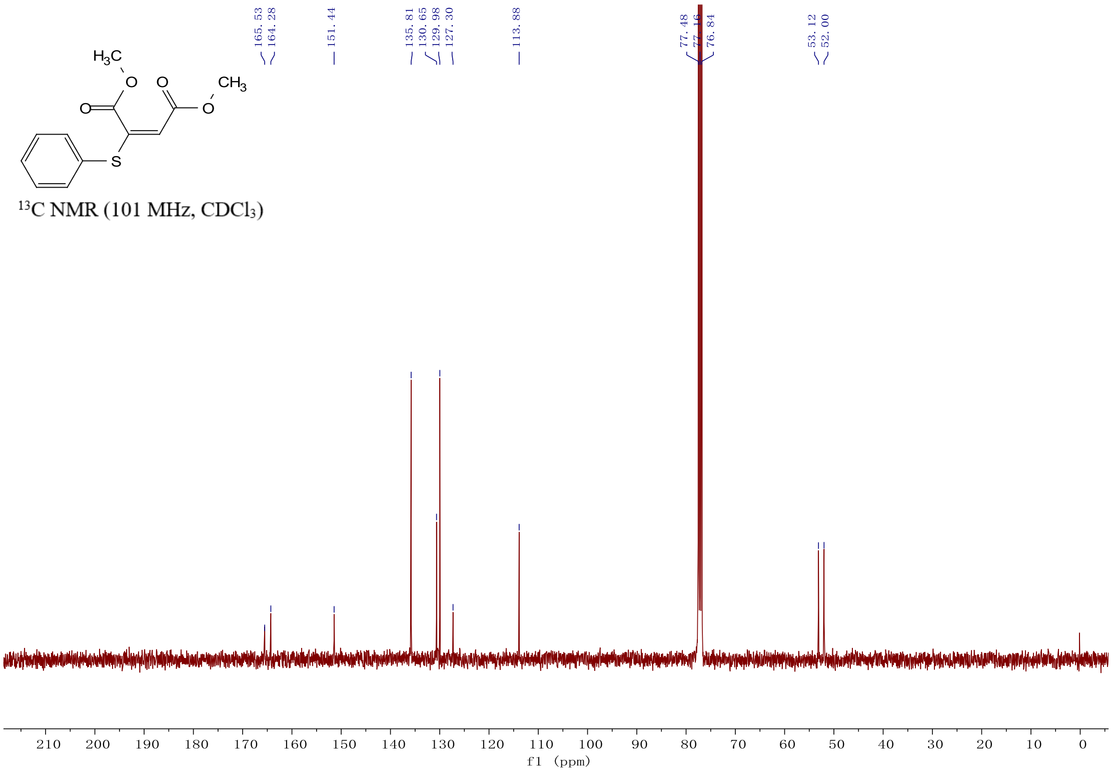

^{13}C NMR (101 MHz, $CDCl_3$)

dimethyl 2-((2-fluorophenyl)thio)fumarate (*Z*-1ab)

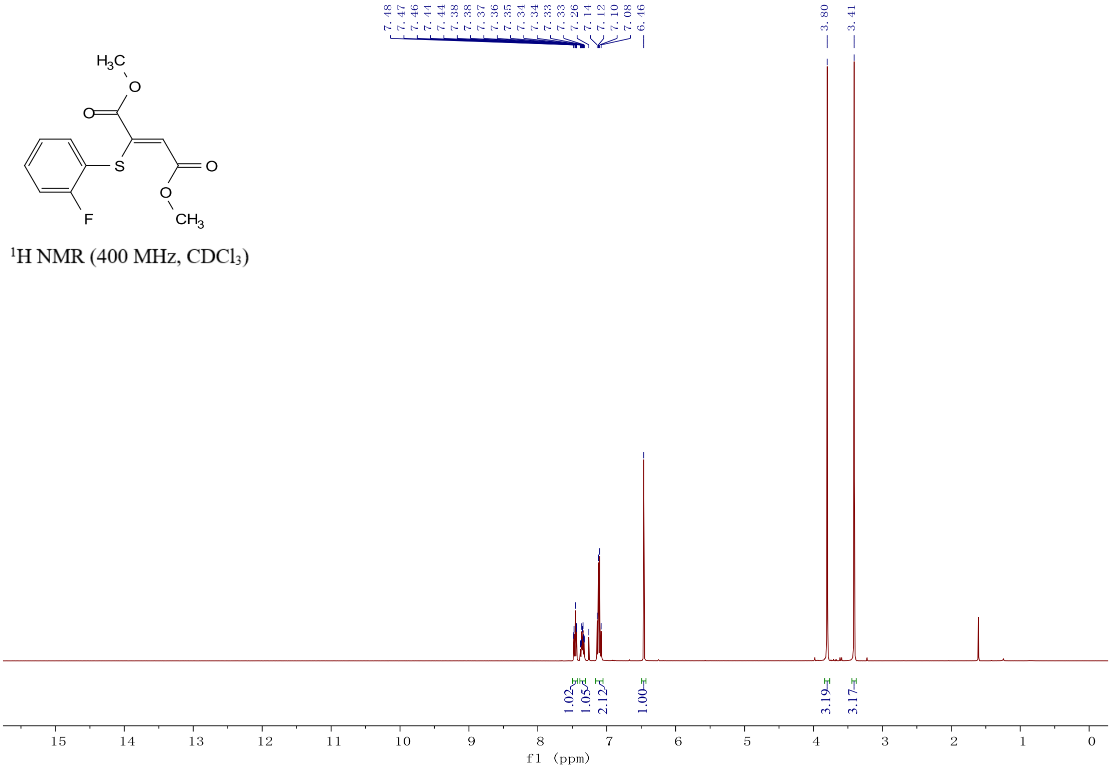

^{1}H NMR (400 MHz, $CDCl_3$)

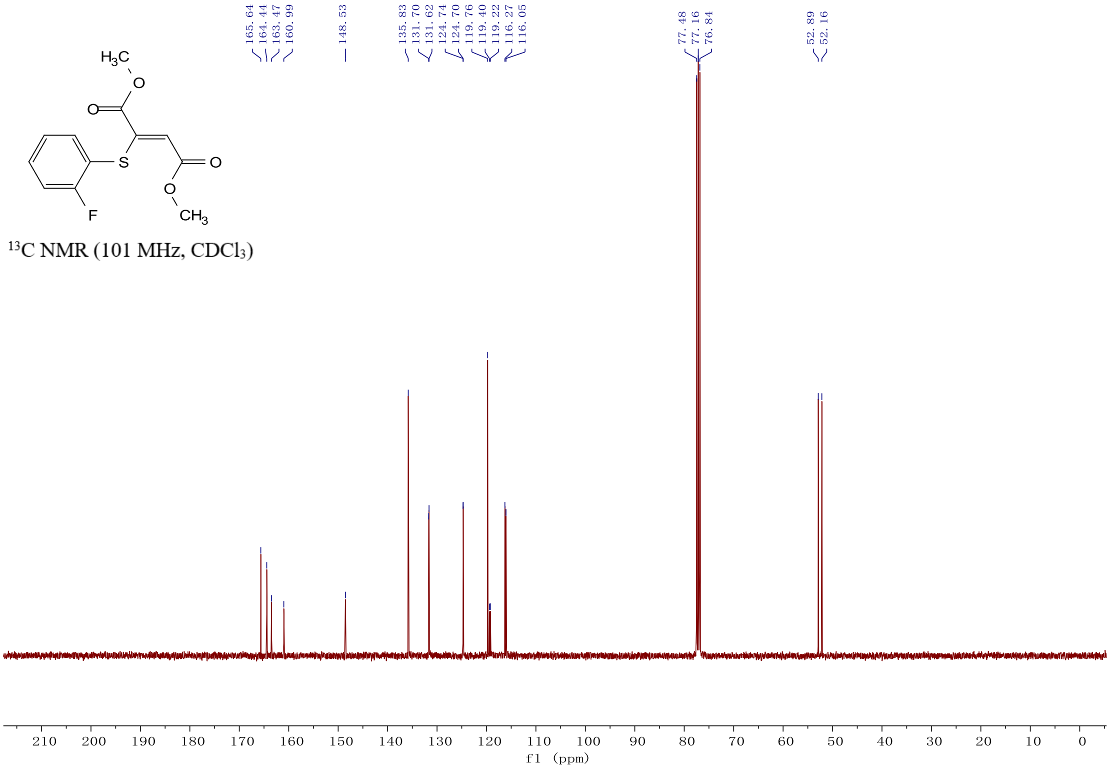

^{13}C NMR (101 MHz, $CDCl_3$)

dimethyl 2-((2-fluorophenyl)thio)fumarate (*Z*-1ab)

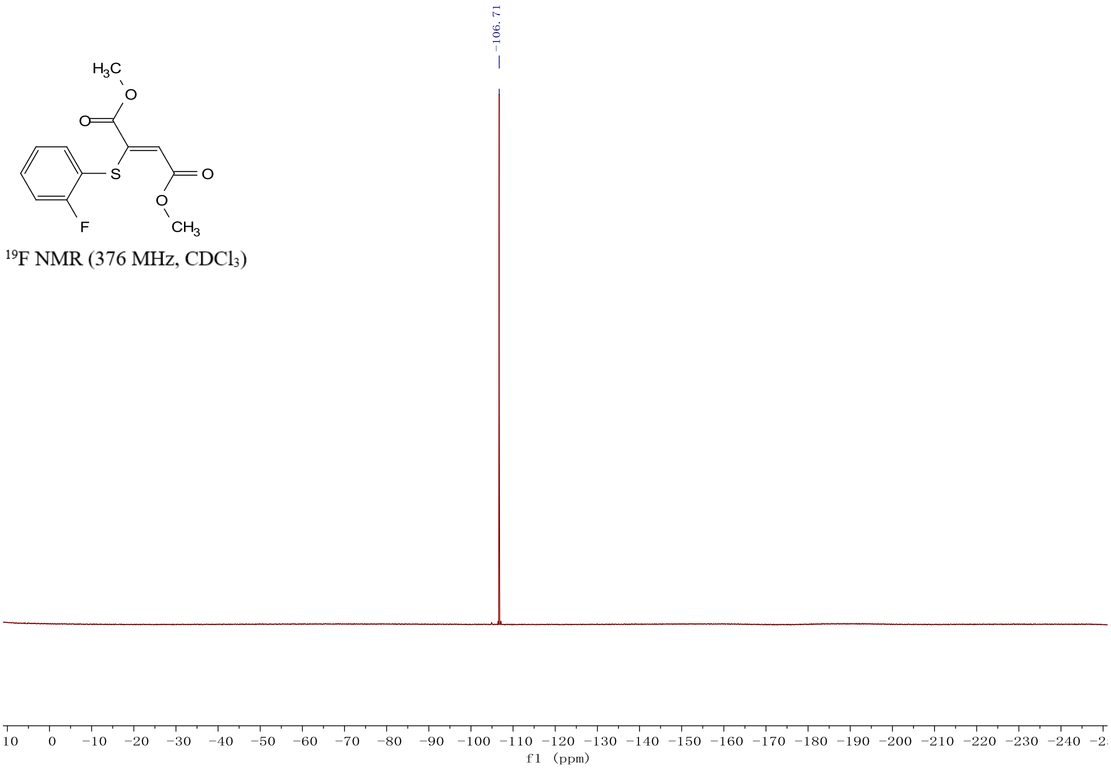

^{19}F NMR (376 MHz, $CDCl_3$)

dimethyl 2-((2-fluorophenyl)thio)maleate (*E*-1ab)

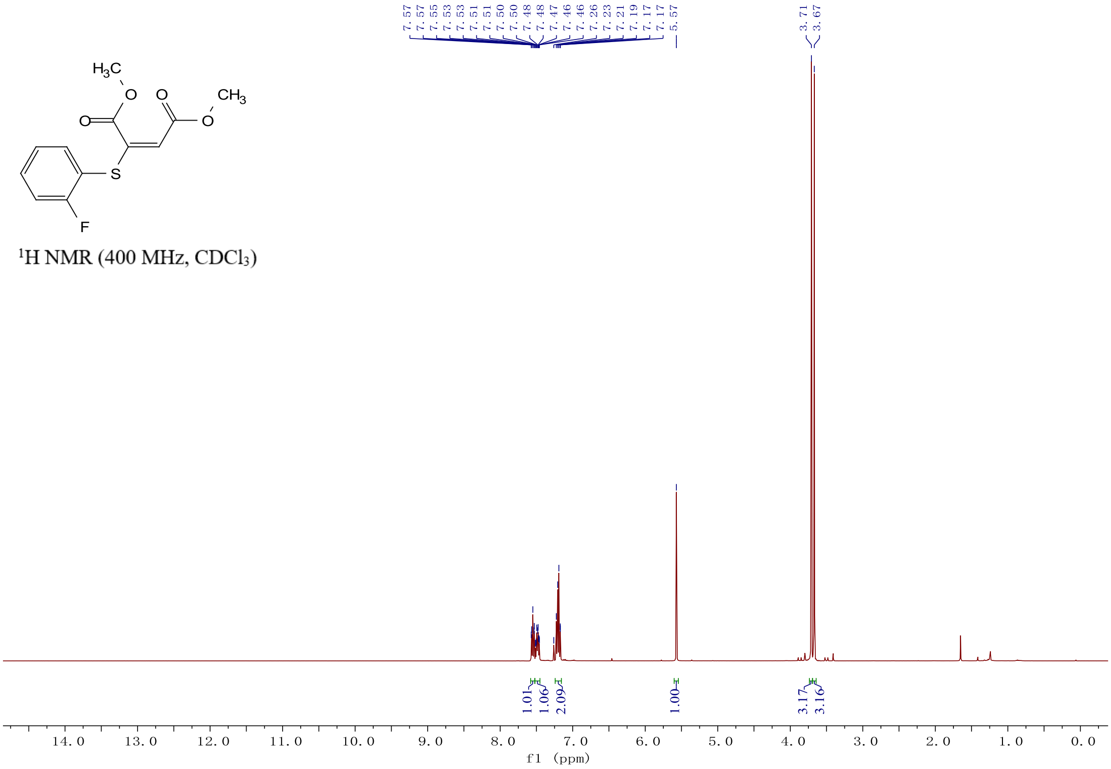

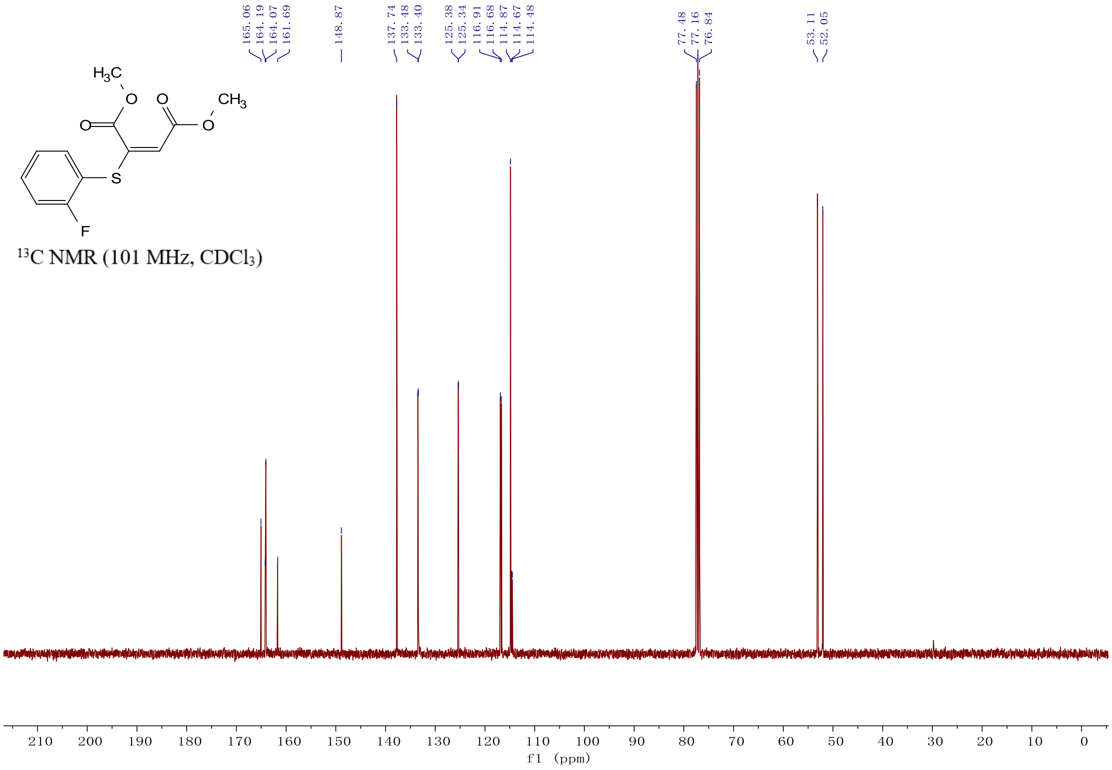

dimethyl 2-((2-fluorophenyl)thio)maleate (*E*-1ab)

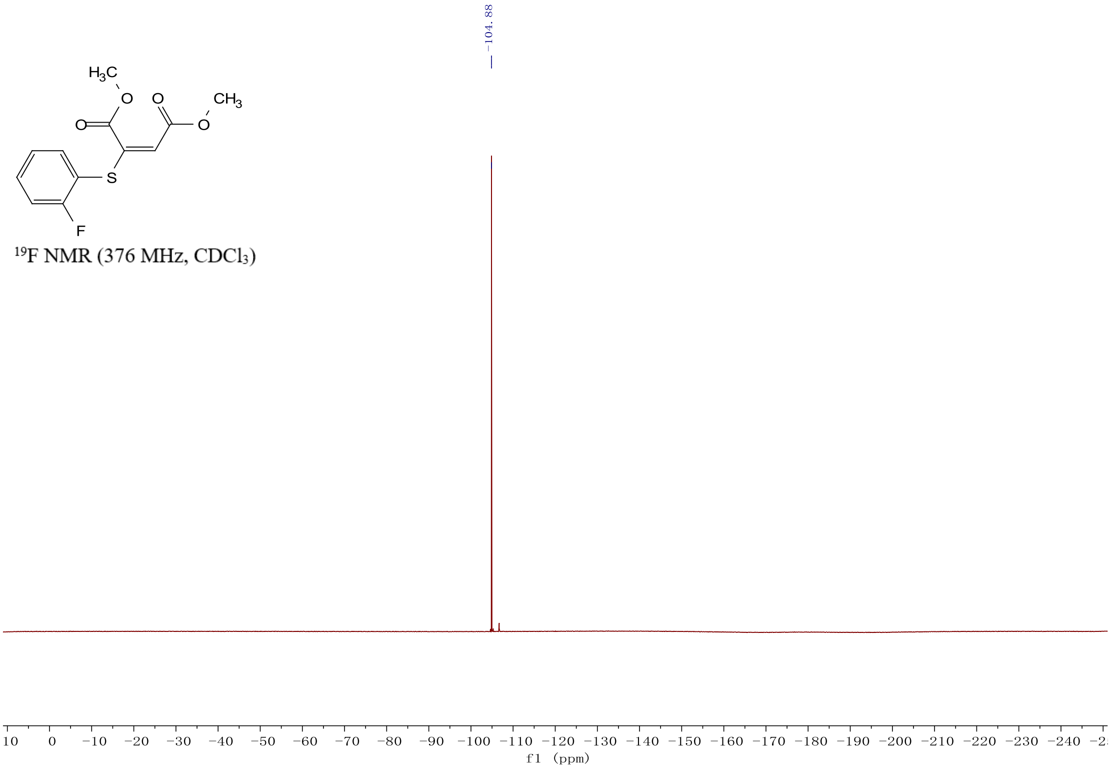

^{19}F NMR (376 MHz, $CDCl_3$)

dimethyl 2-((2-chlorophenyl)thio)fumarate (*Z*-1ac)

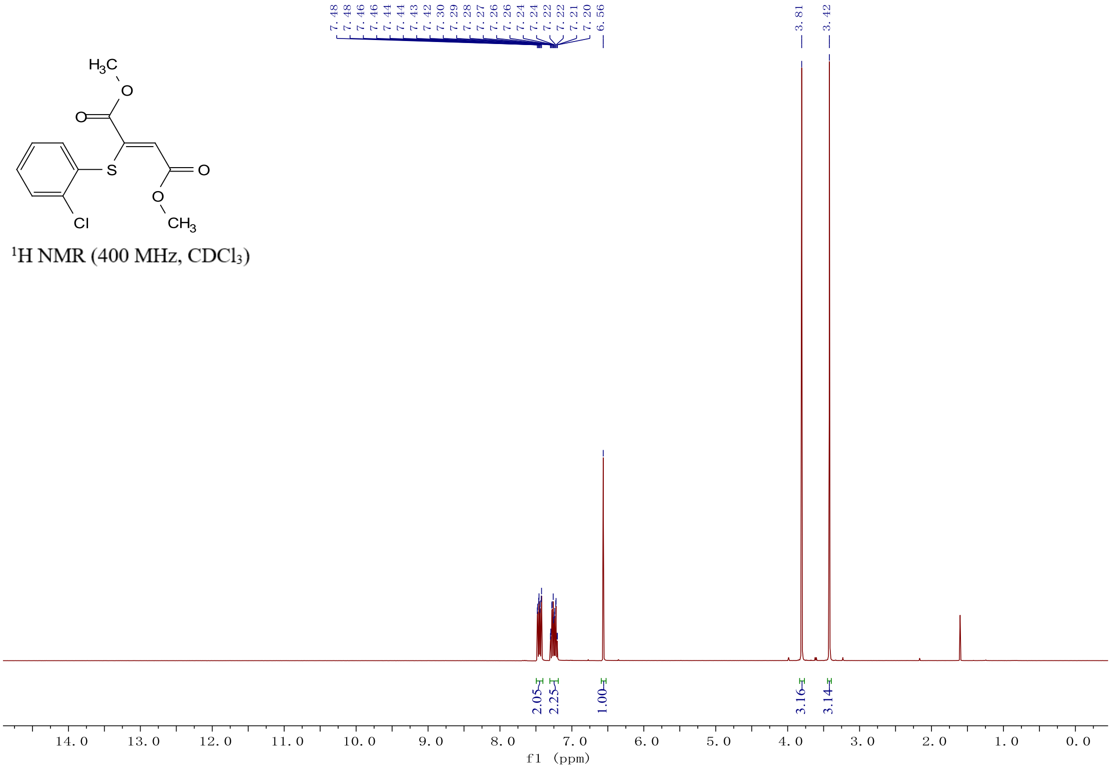

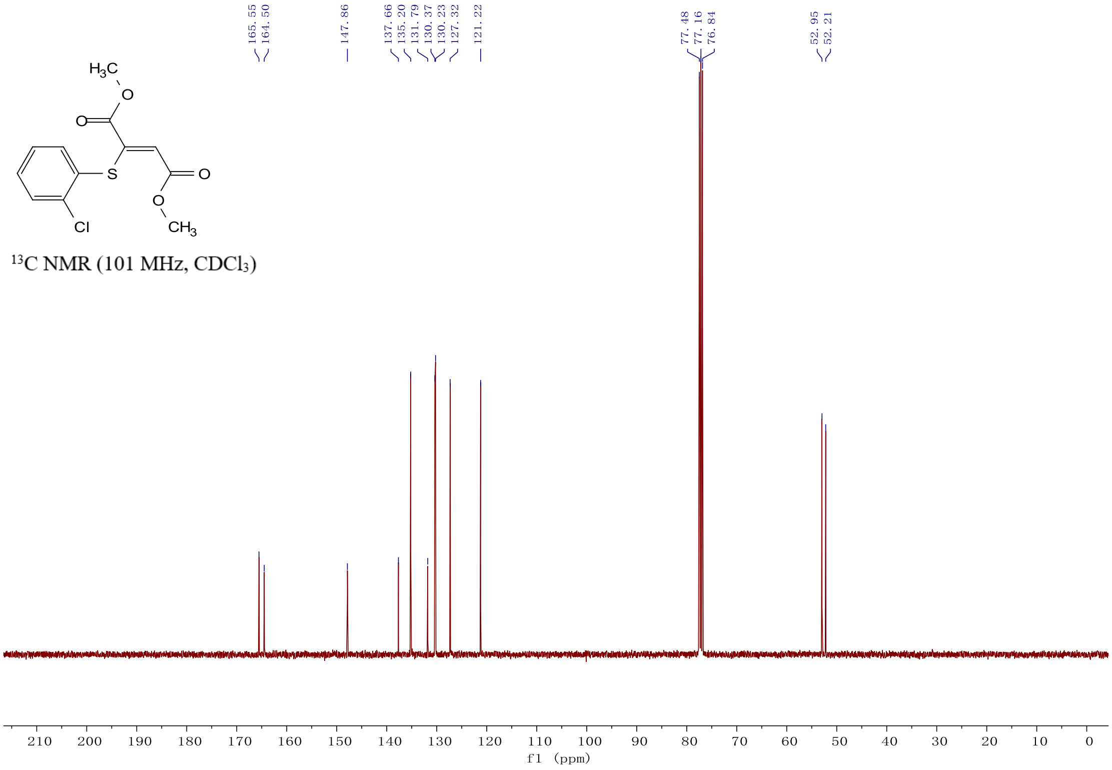

dimethyl 2-((2-chlorophenyl)thio)maleate (*E*-1ac)

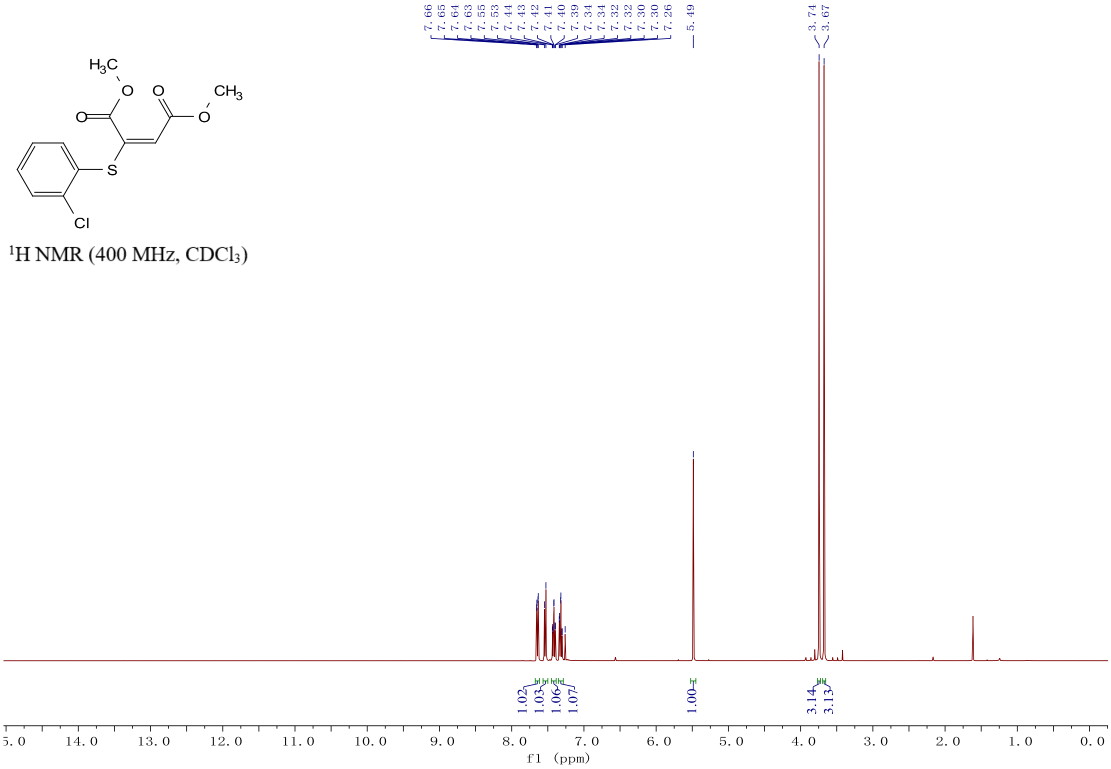

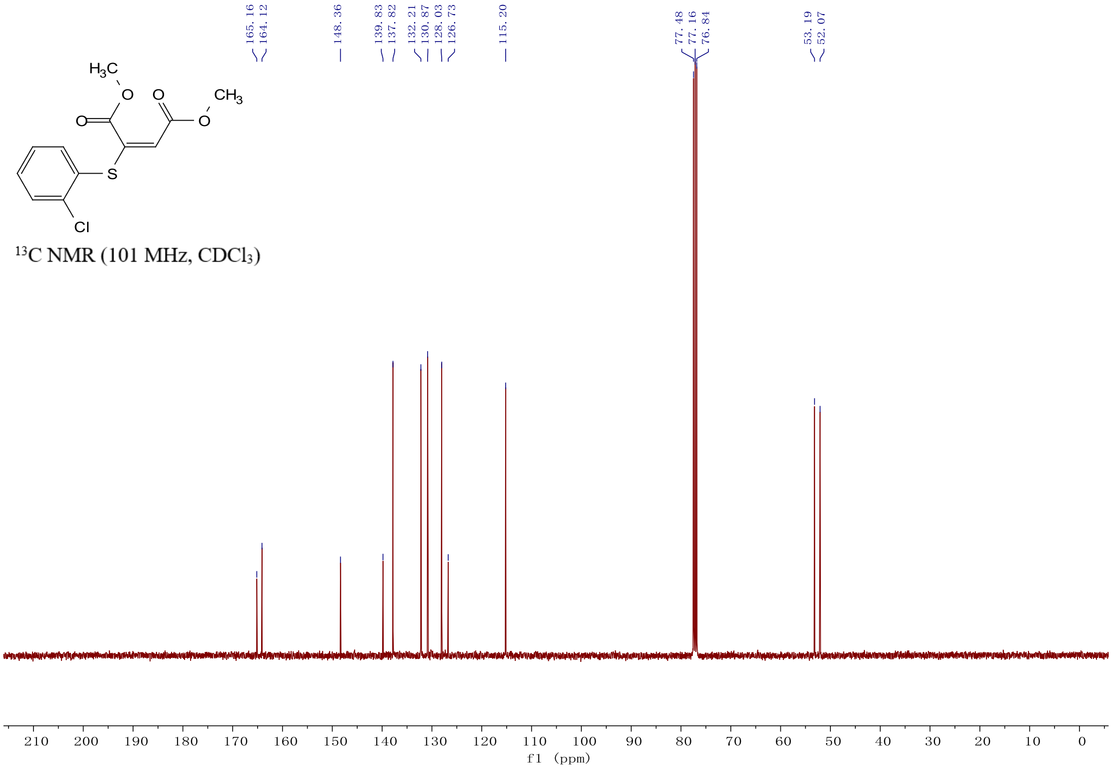

dimethyl 2-((4-chlorophenyl)thio)fumarate (*Z*-1ad)

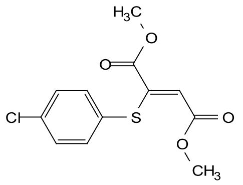

^{1}H NMR (400 MHz, $CDCl_3$)

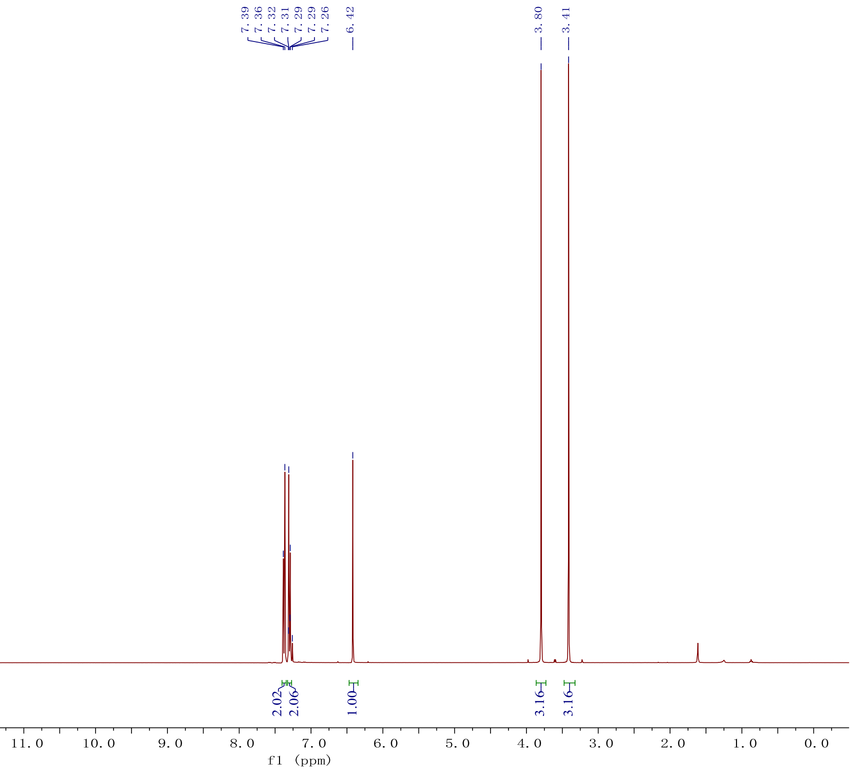

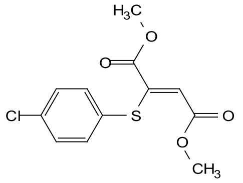

^{13}C NMR (101 MHz, $CDCl_3$)

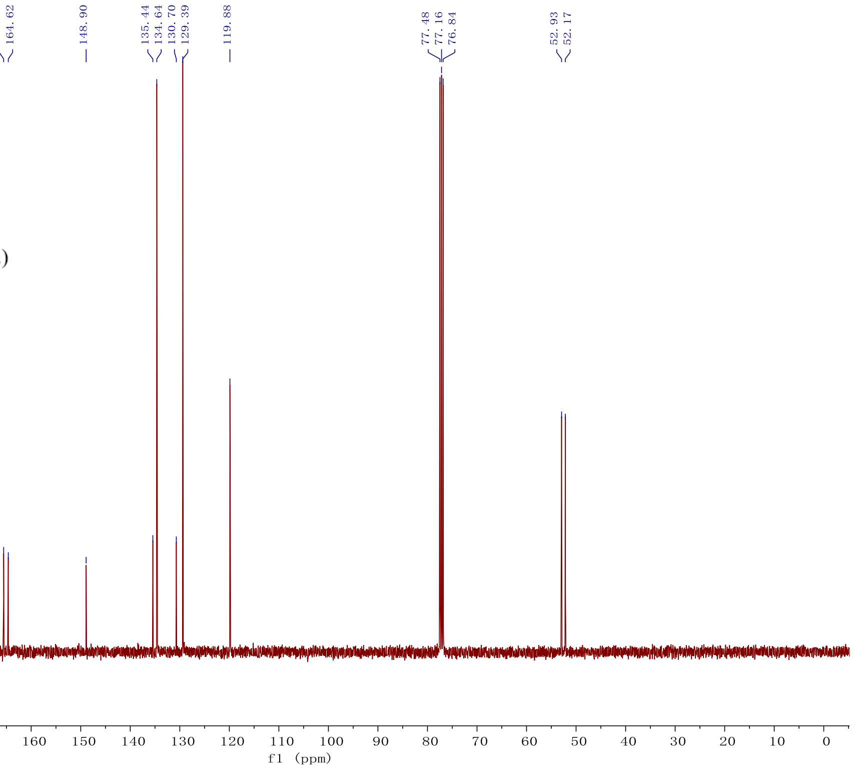

dimethyl 2-((4-chlorophenyl)thio)maleate (*E*-1ad)

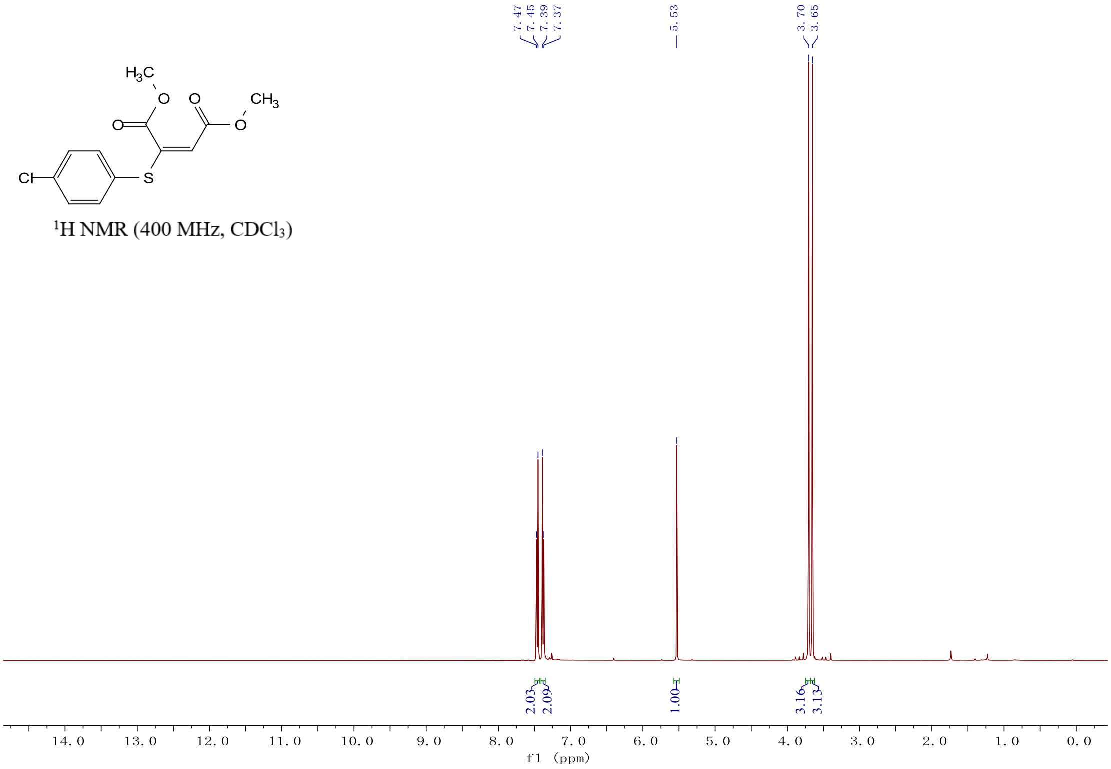

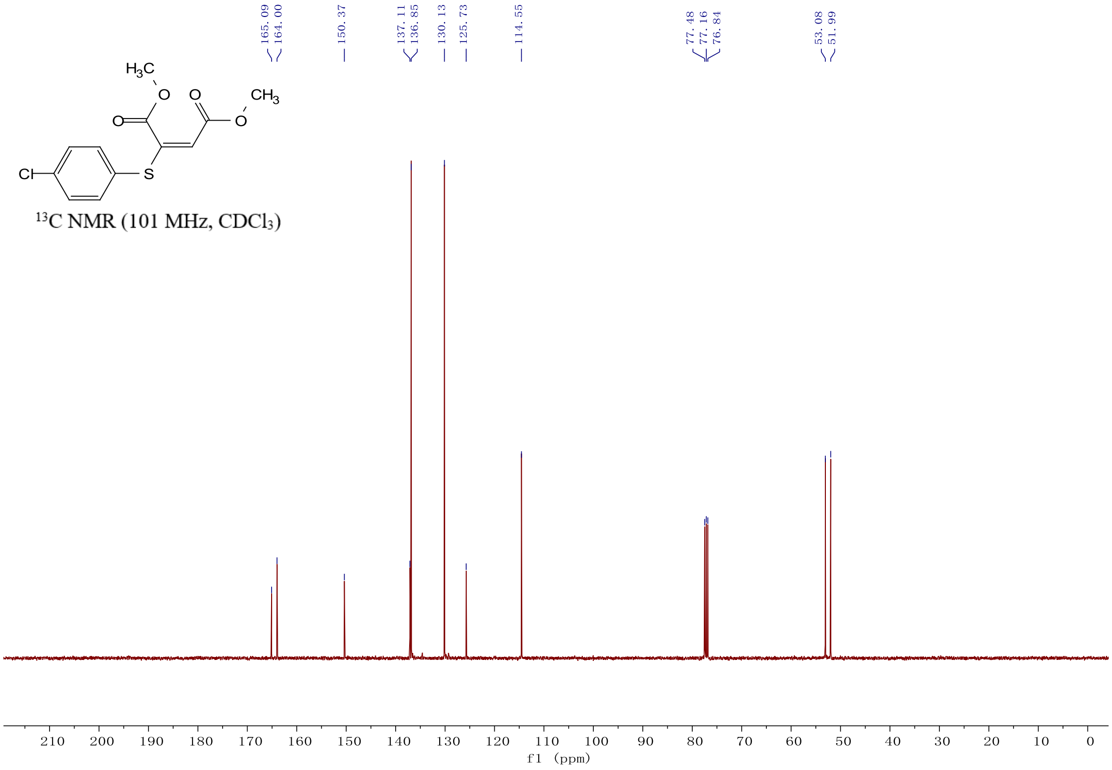

dimethyl 2-((2-bromophenyl)thio)fumarate (*Z*-1ae)

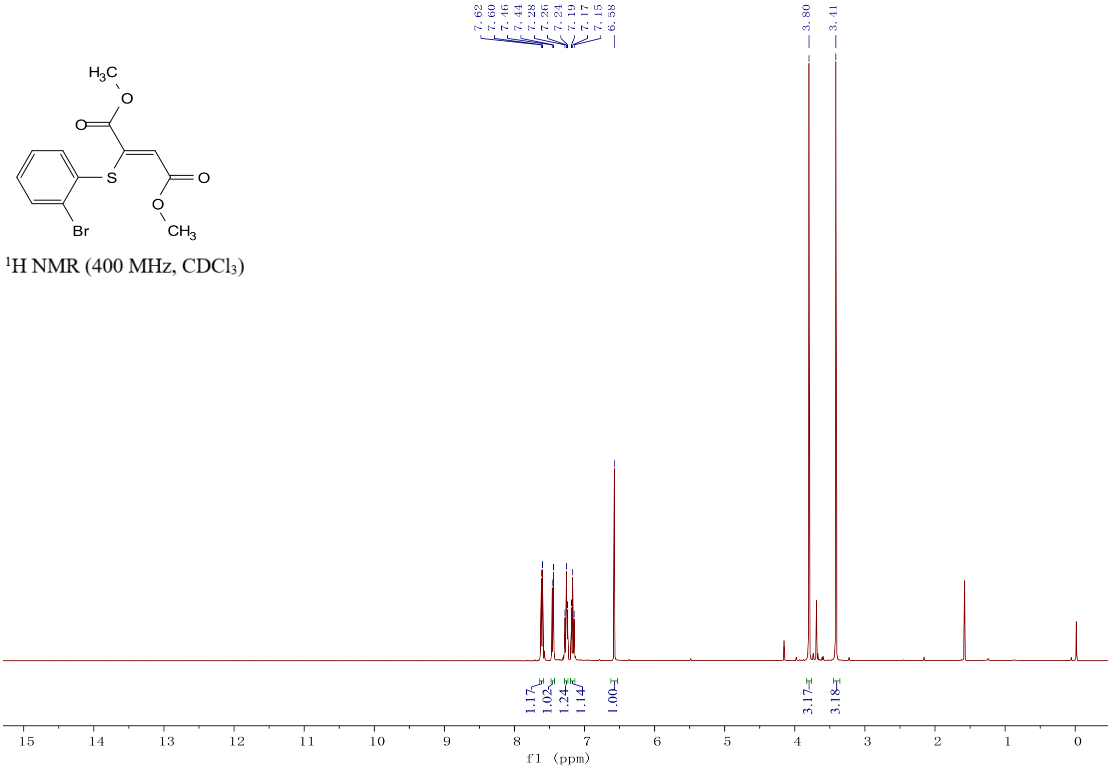

^{1}H NMR (400 MHz, $CDCl_3$)

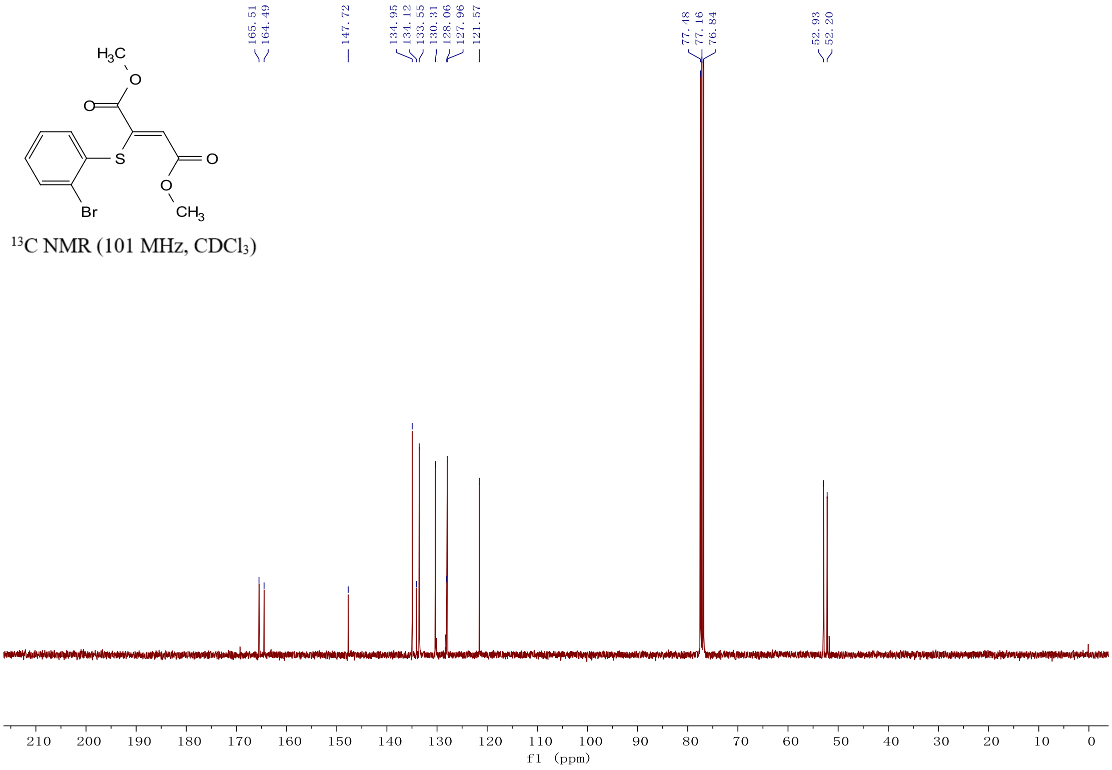

^{13}C NMR (101 MHz, $CDCl_3$)

dimethyl 2-((2-bromophenyl)thio)maleate (*E*-1ae)

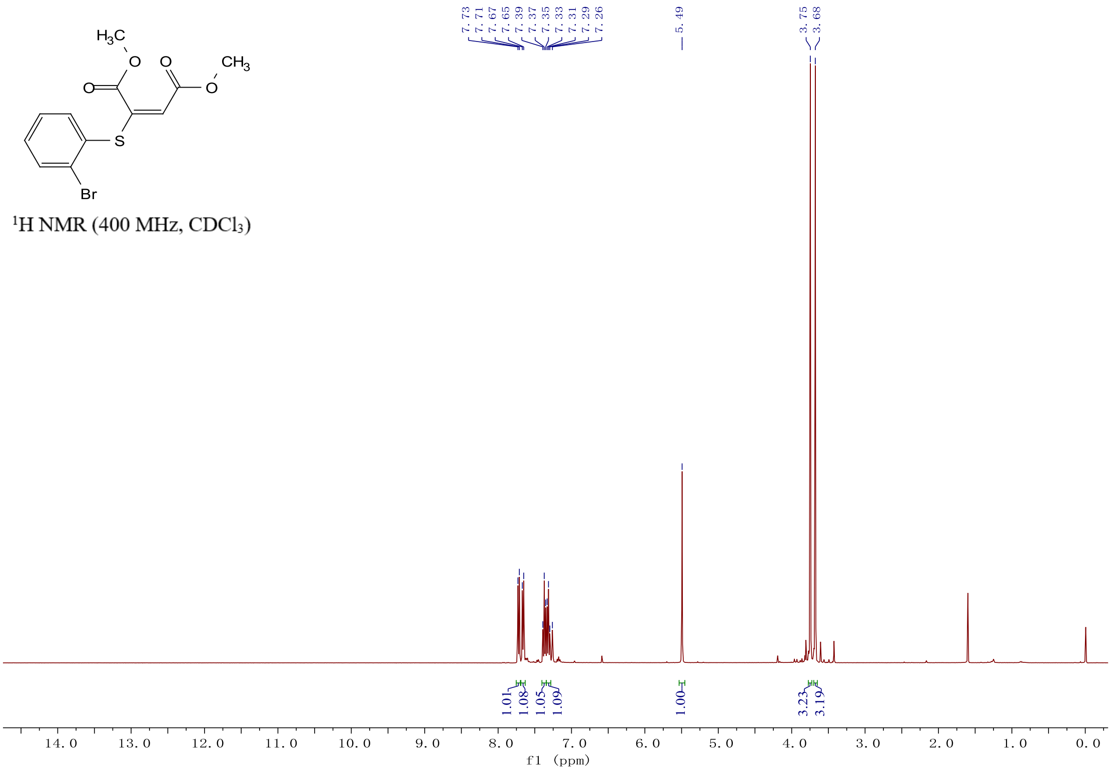

^{1}H NMR (400 MHz, $CDCl_3$)

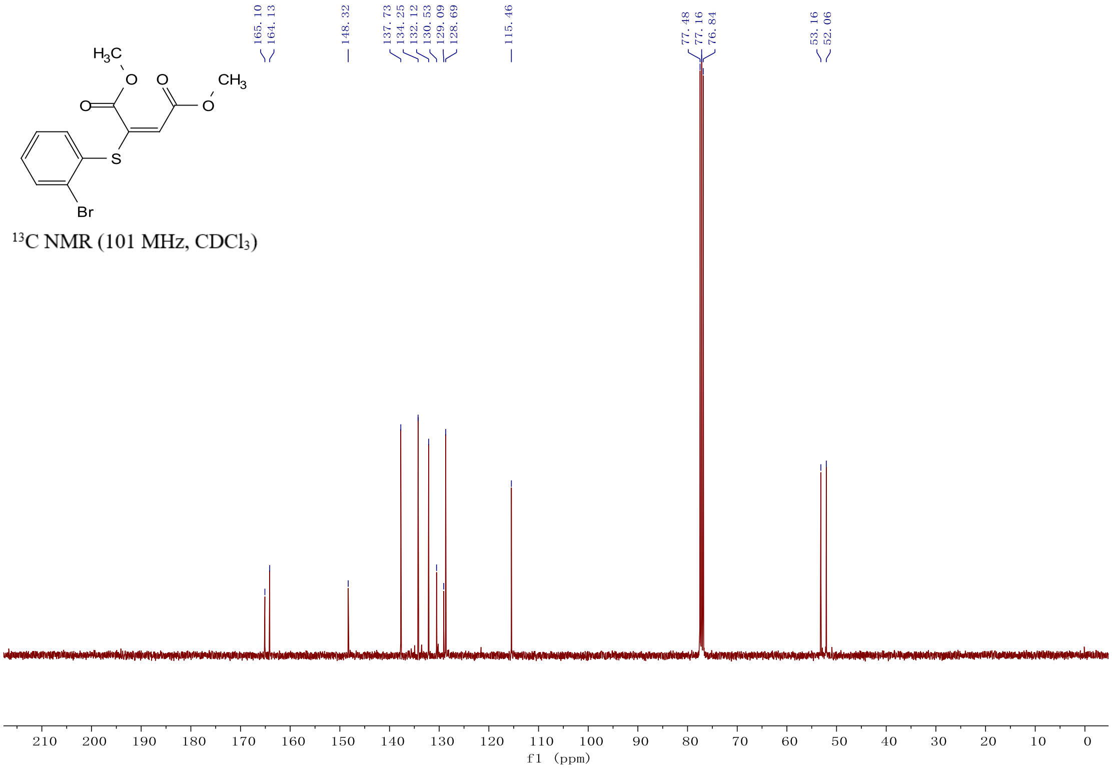

^{13}C NMR (101 MHz, $CDCl_3$)

dimethyl 2-((4-bromophenyl)thio)fumarate (*Z*-1af)

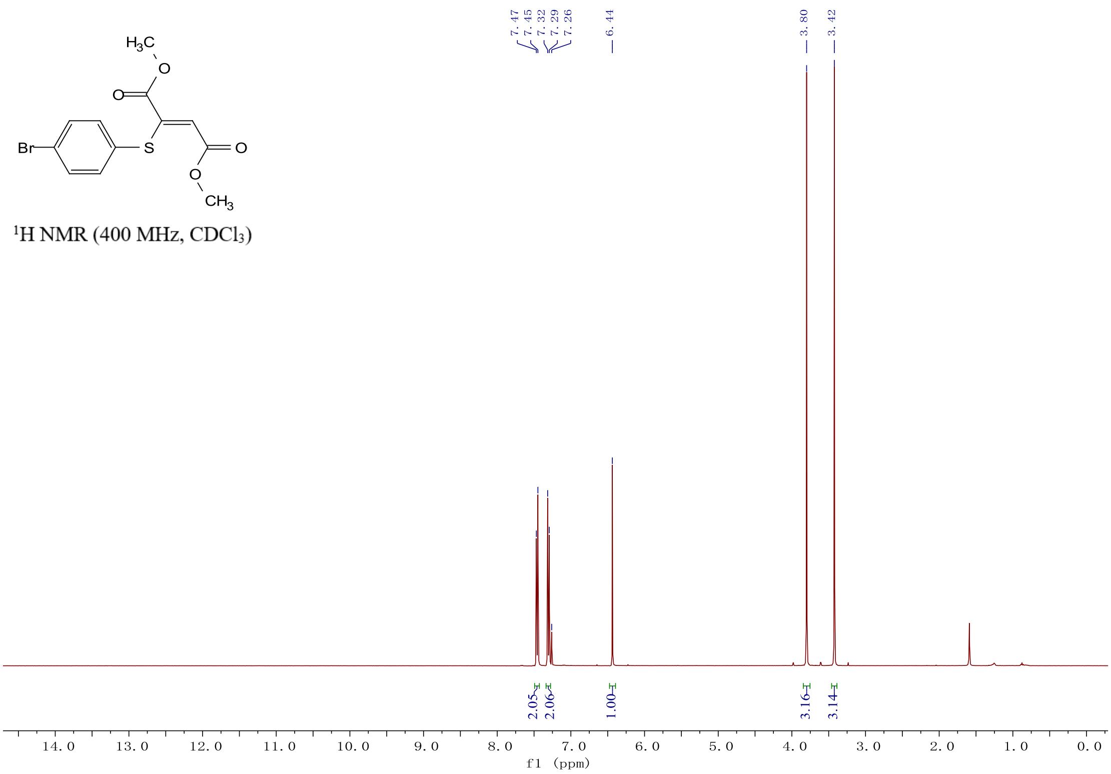

^{1}H NMR (400 MHz, $CDCl_3$)

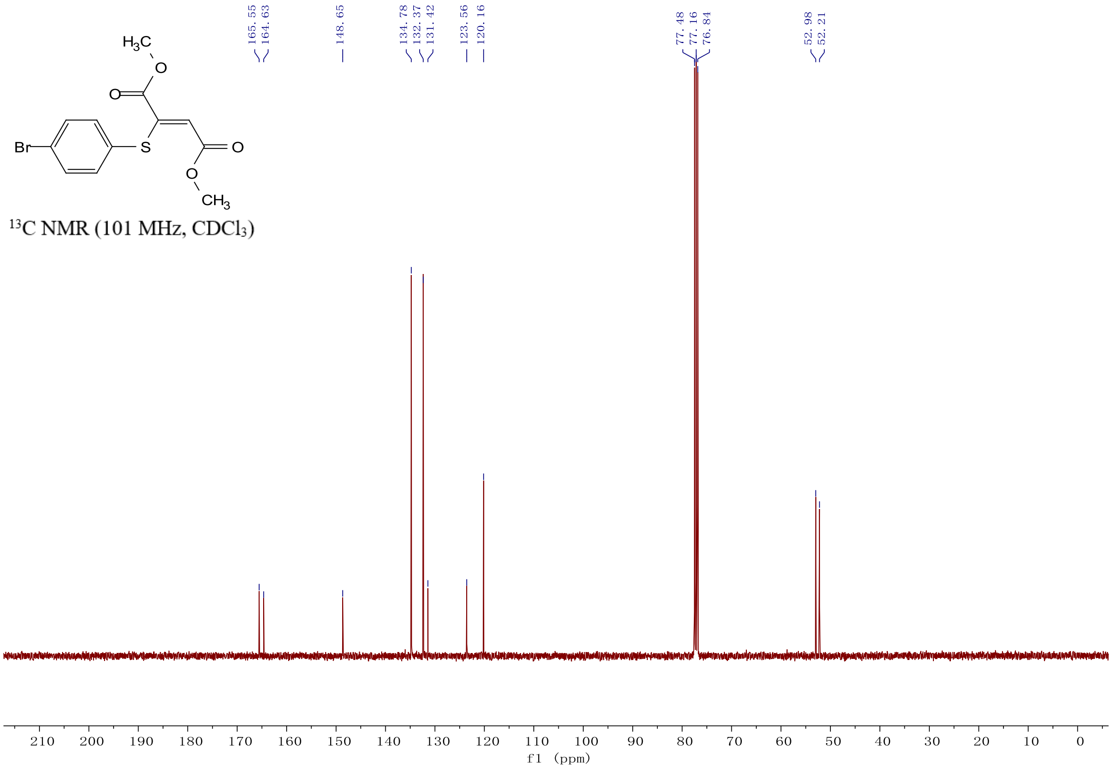

^{13}C NMR (101 MHz, $CDCl_3$)

dimethyl 2-((4-bromophenyl)thio)maleate (*E*-1af)

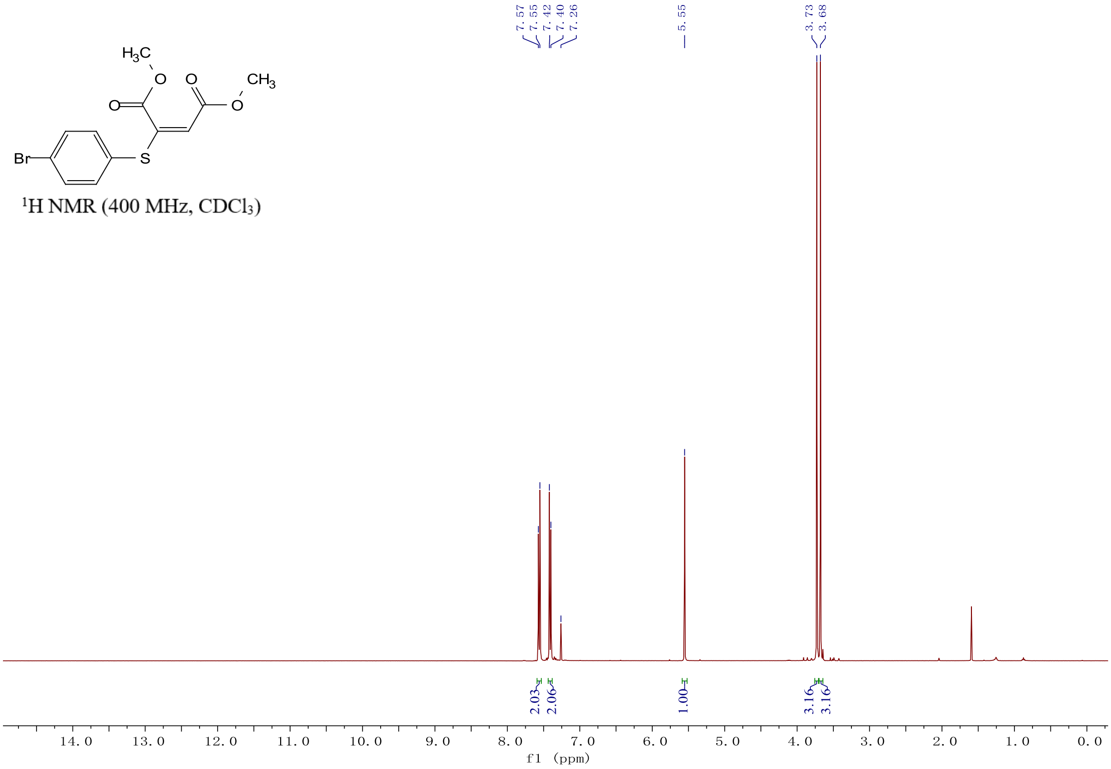

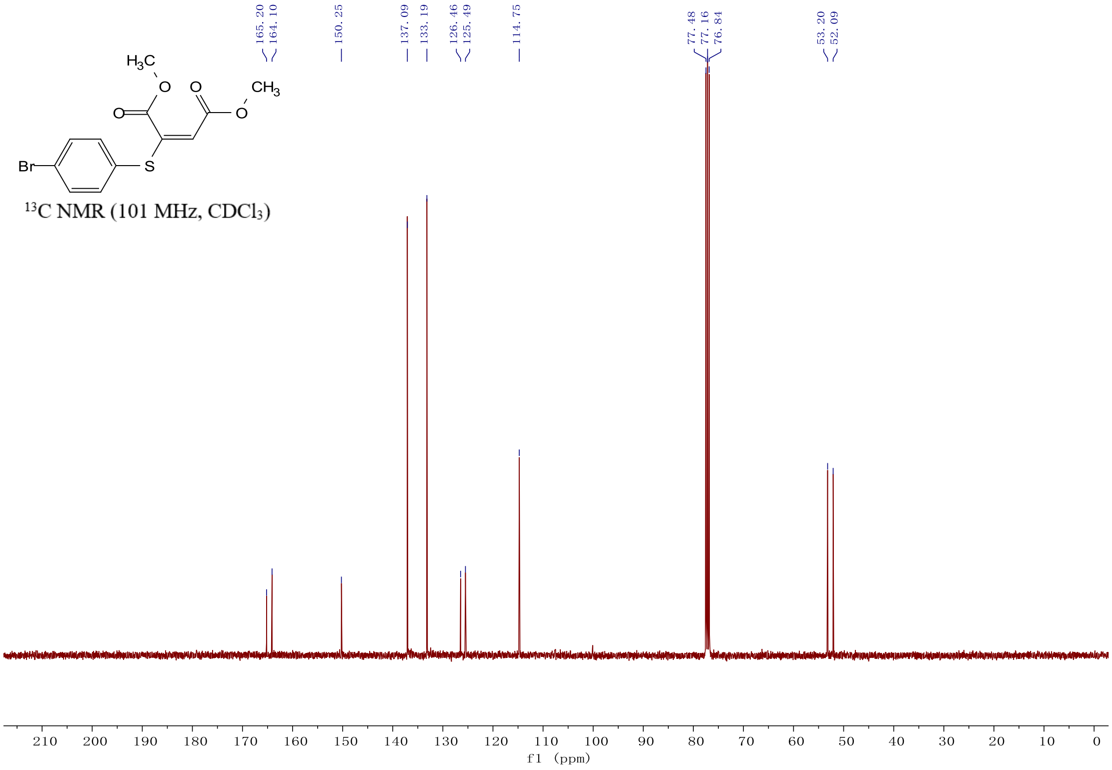

dimethyl 2-(*p*-tolylthio)fumarate (*Z*-1ag)

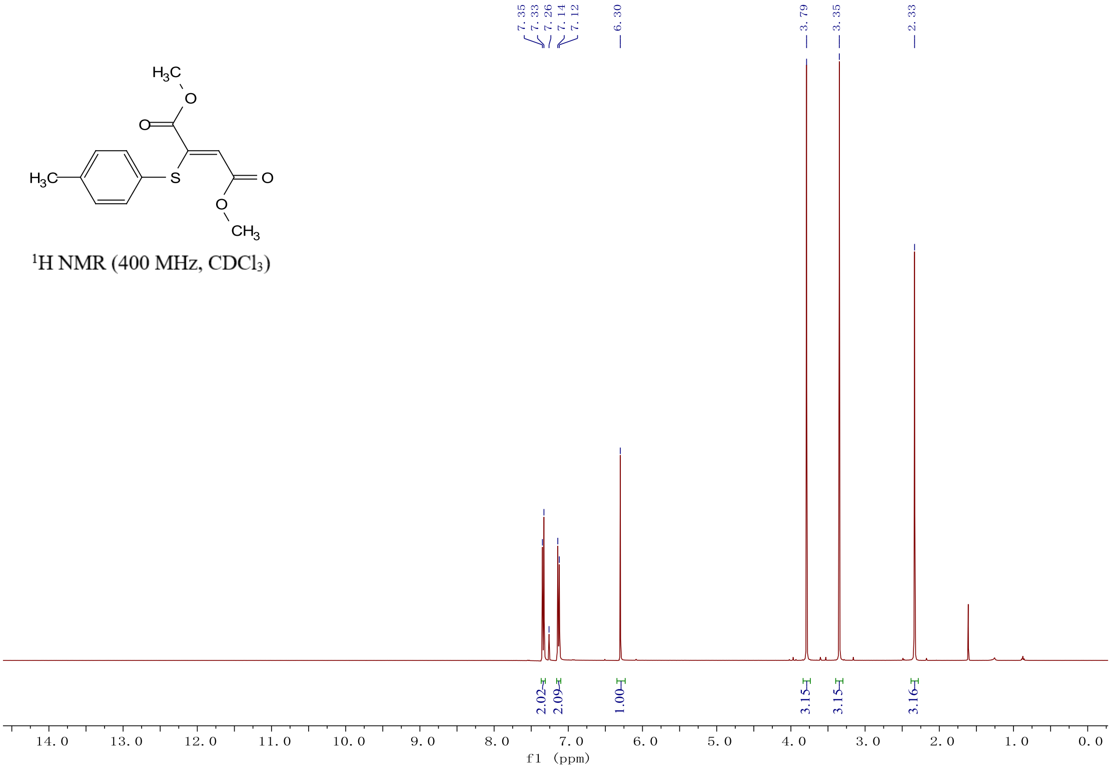

^{1}H NMR (400 MHz, $CDCl_3$)

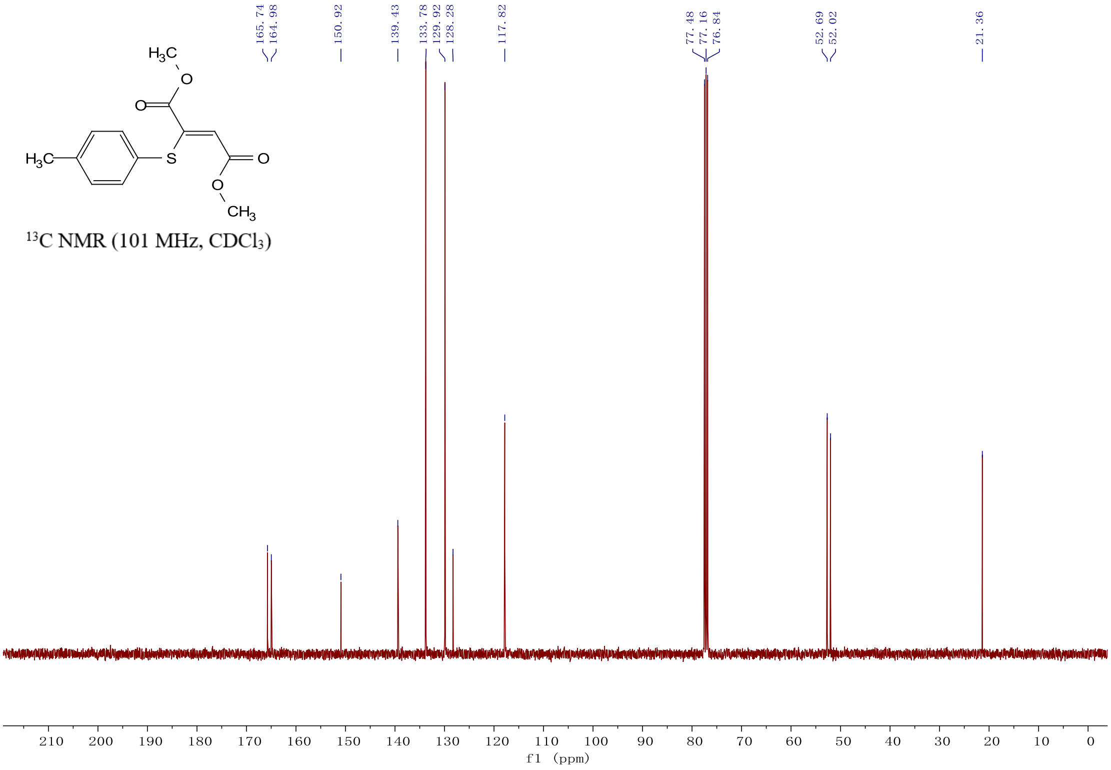

^{13}C NMR (101 MHz, $CDCl_3$)

dimethyl 2-(*p*-tolylthio)maleate (*E*-1ag)

^{1}H NMR (400 MHz, $CDCl_3$)

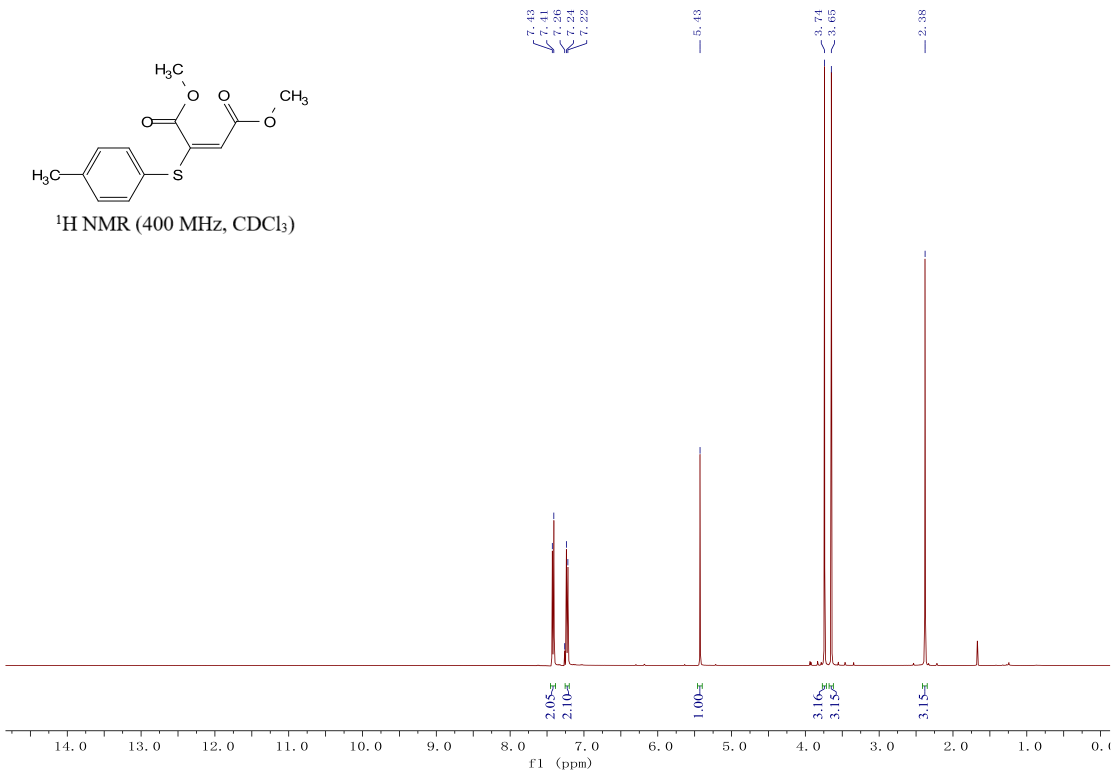

^{13}C NMR (101 MHz, $CDCl_3$)

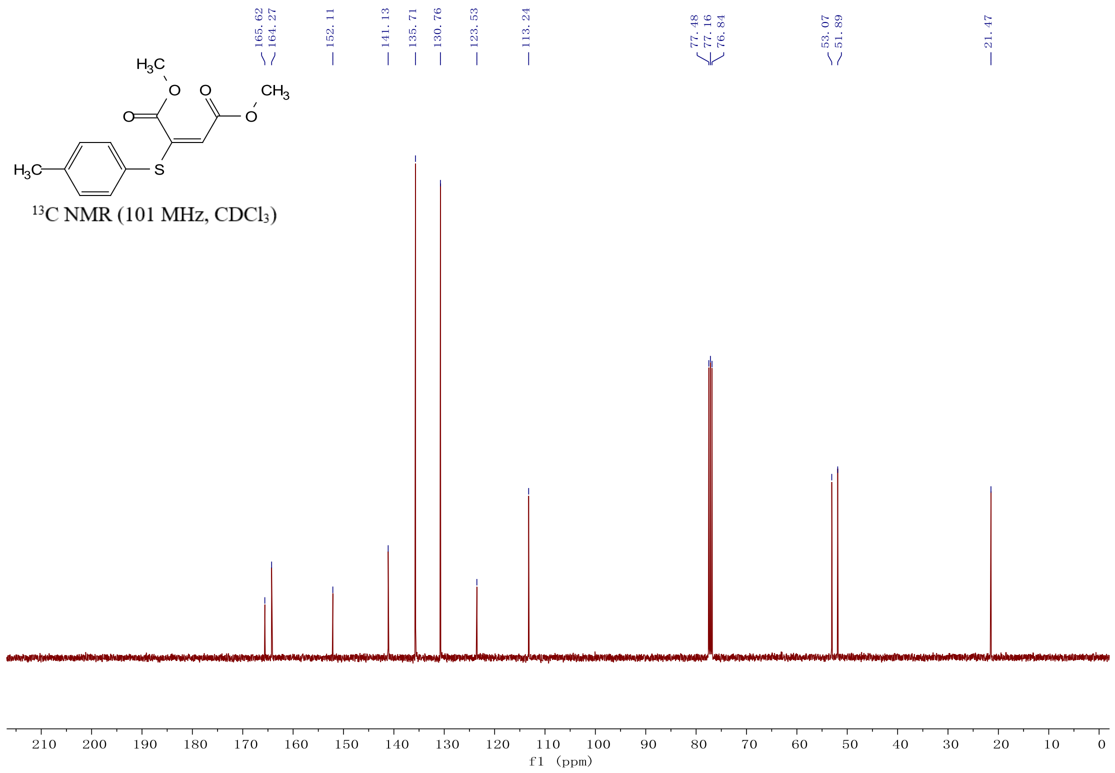

dimethyl 2-((3-methoxyphenyl)thio)fumarate (*Z*-1ah)

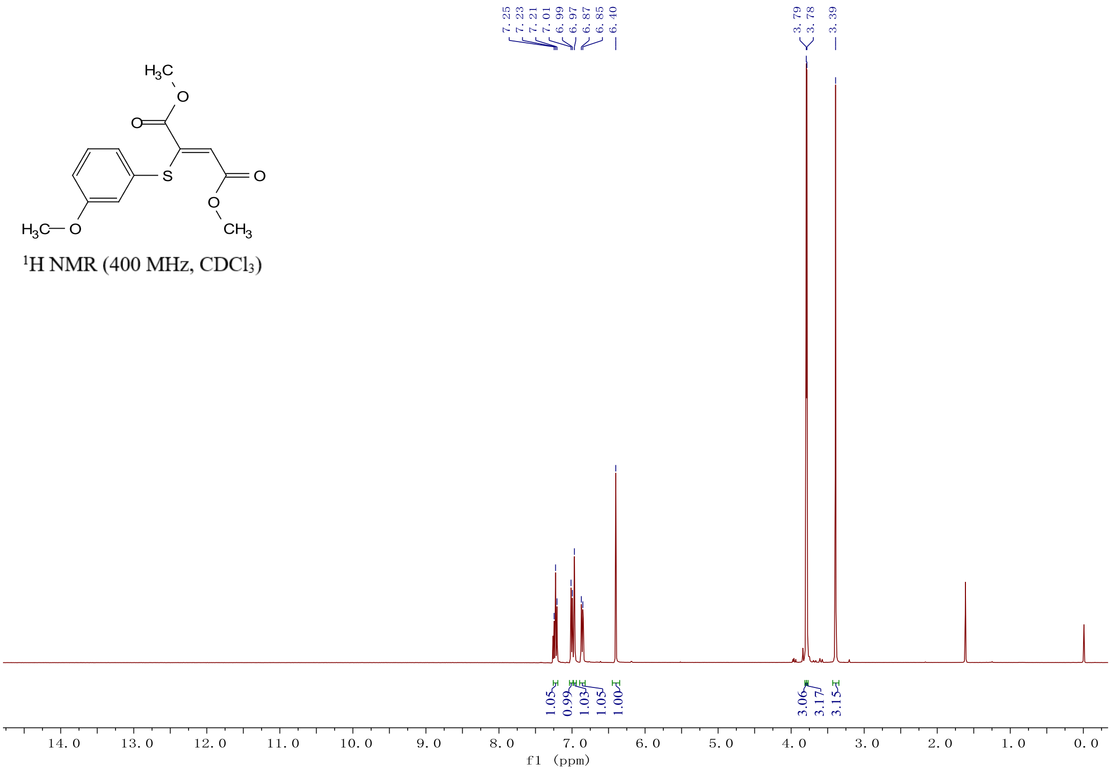

^{1}H NMR (400 MHz, $CDCl_3$)

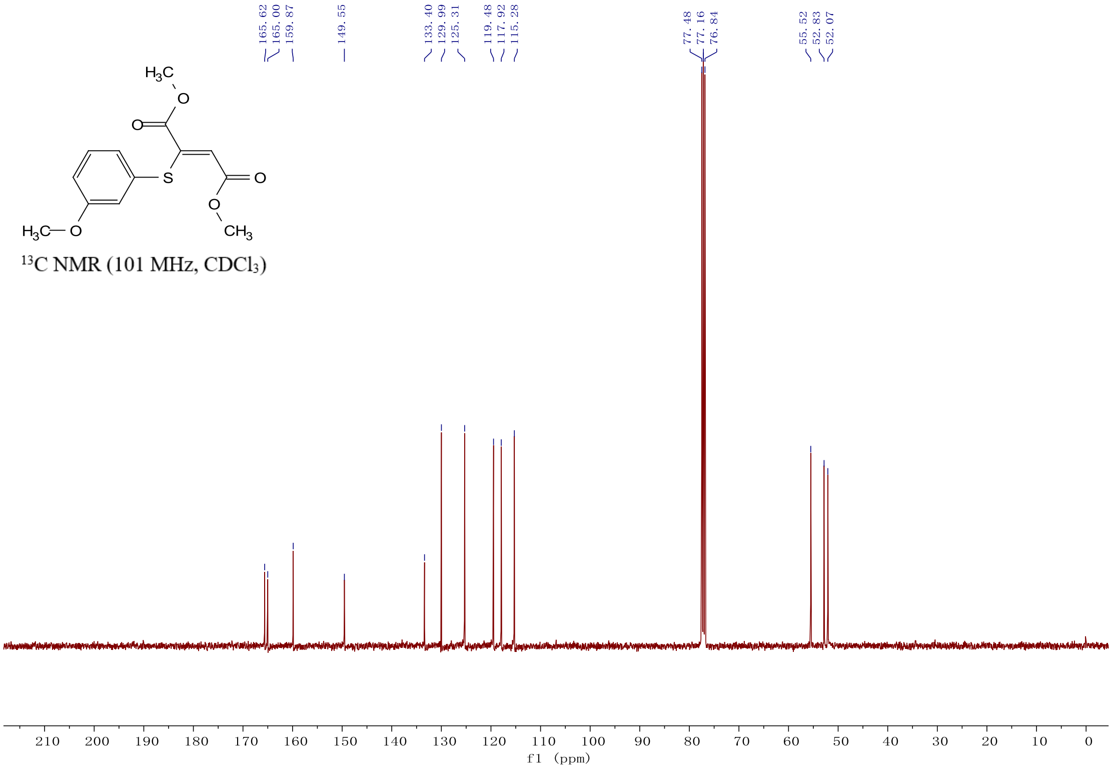

^{13}C NMR (101 MHz, $CDCl_3$)

dimethyl 2-((3-methoxyphenyl)thio)maleate (*E*-1ah)

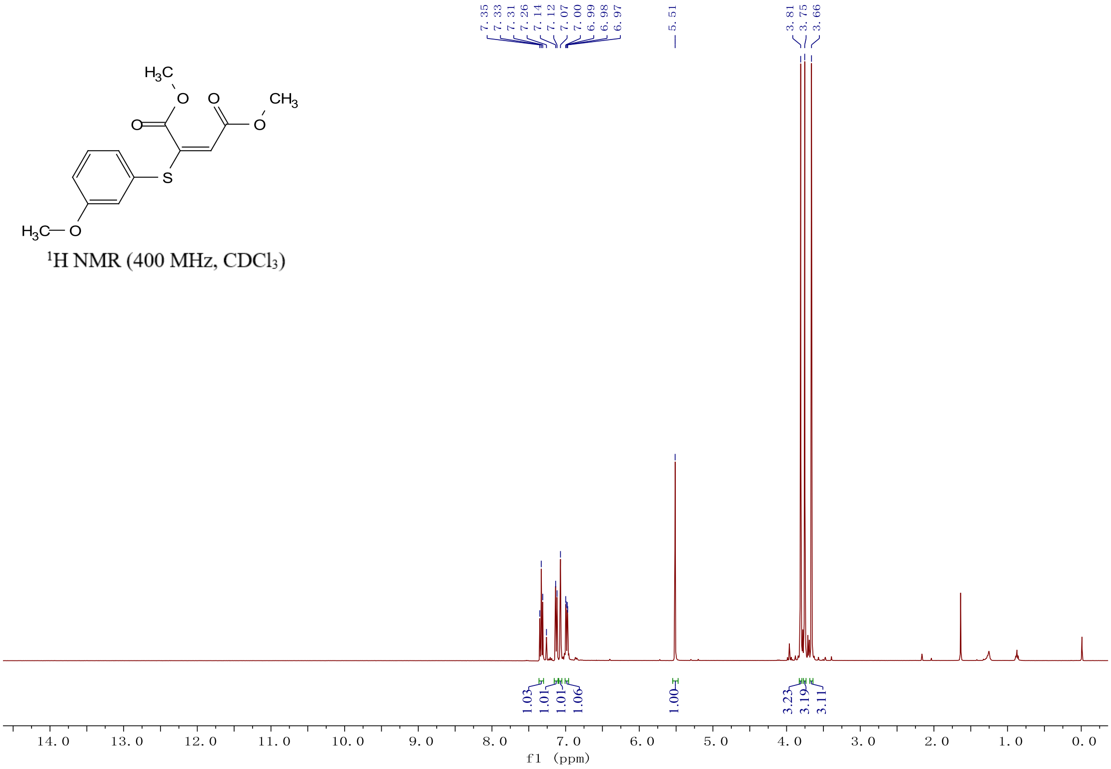

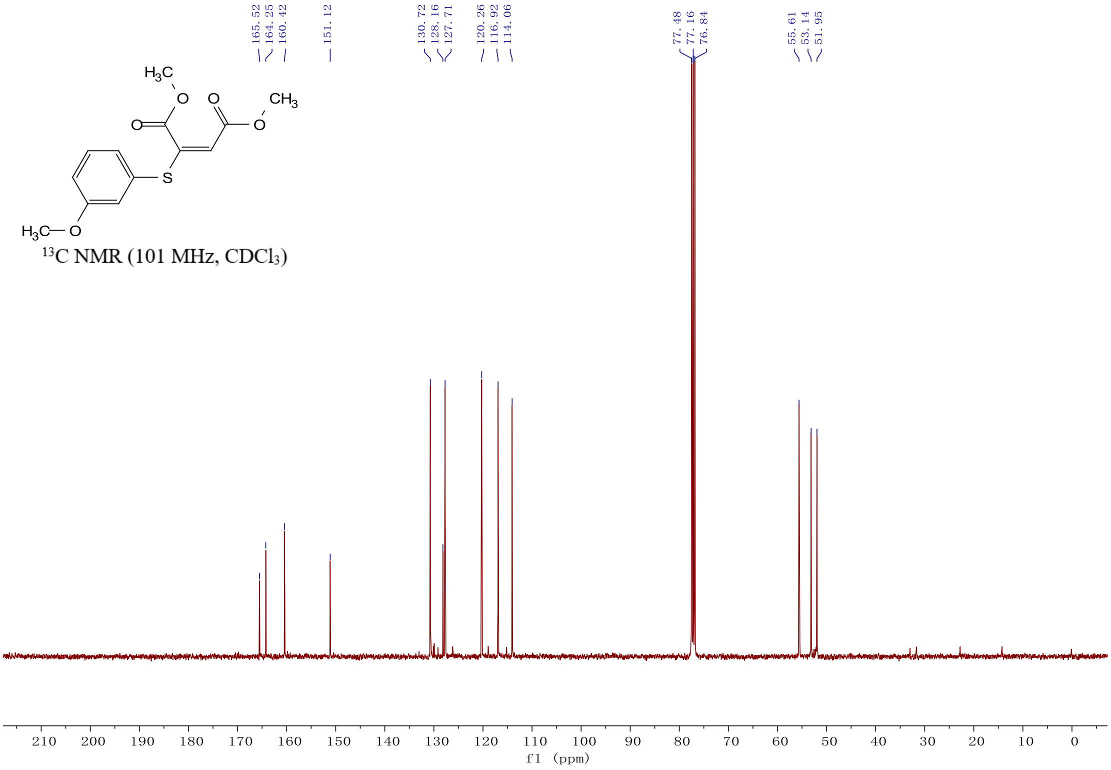

dimethyl 2-((4-methoxyphenyl)thio)fumarate (*Z*-1ai)

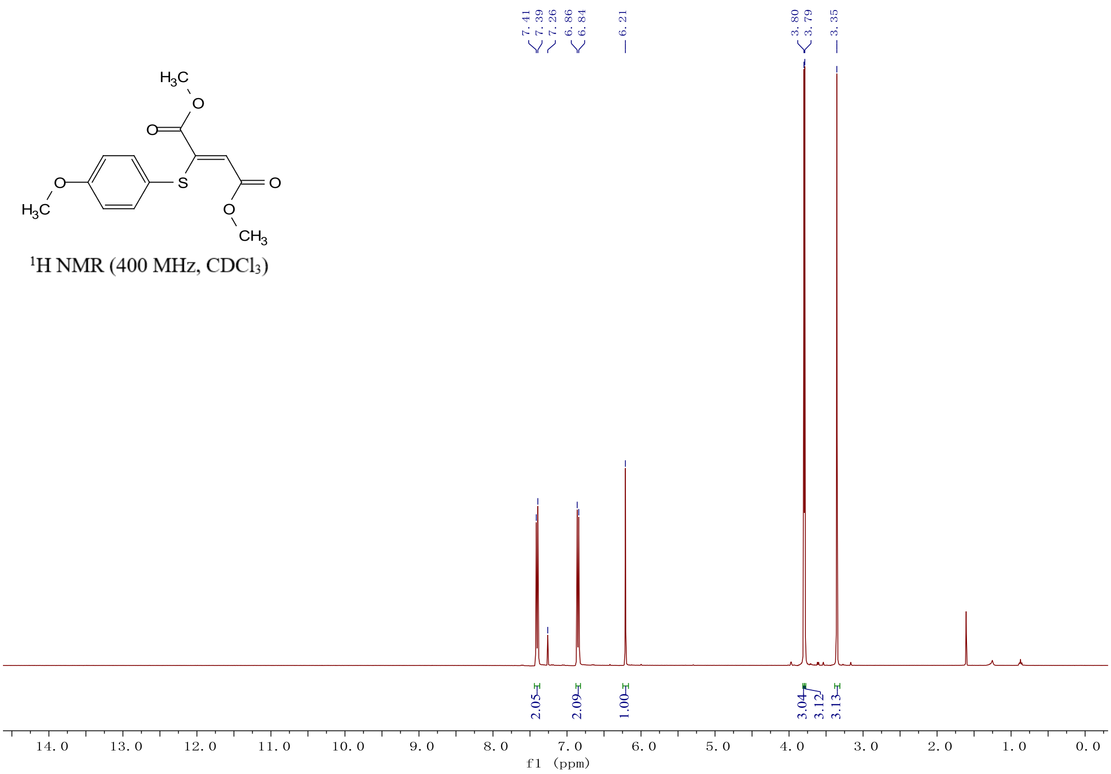

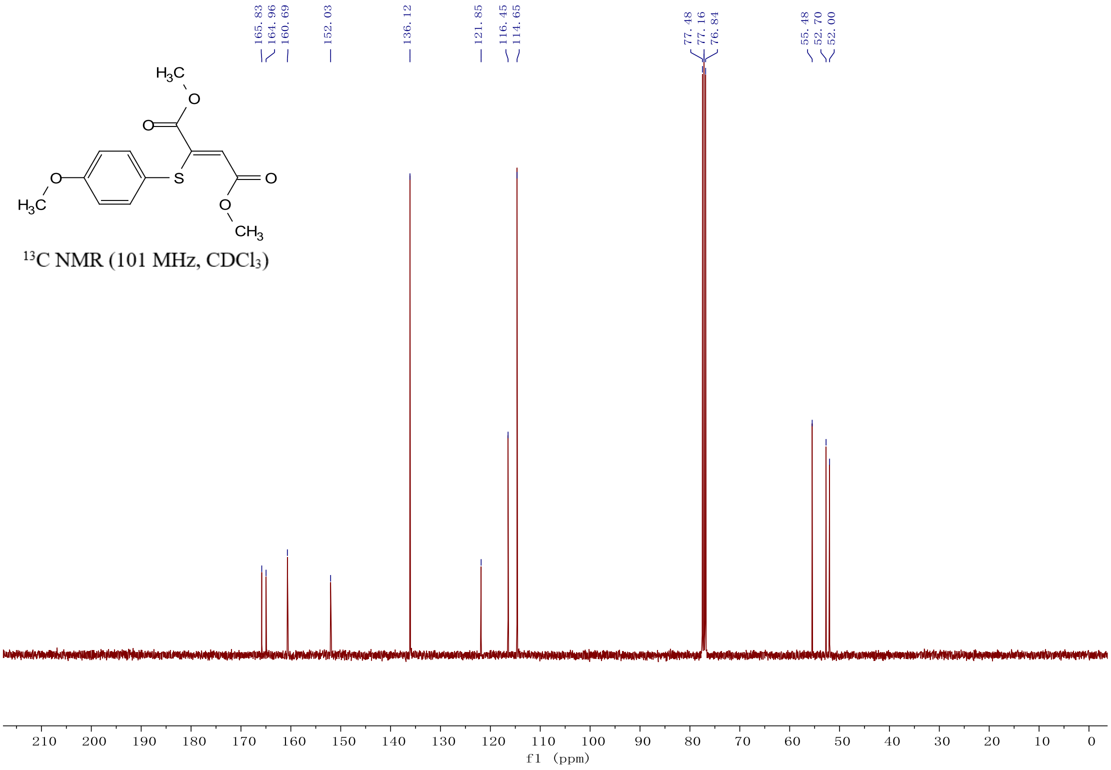

dimethyl 2-((4-methoxyphenyl)thio)maleate (*E*-1ai)

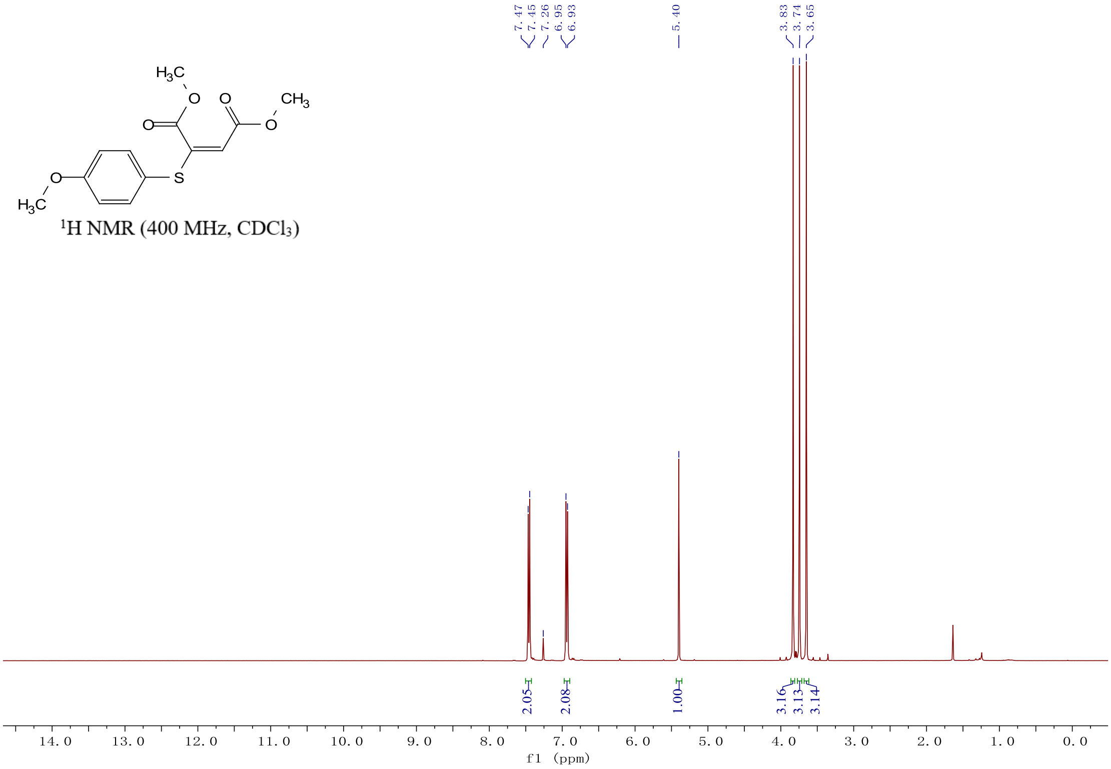

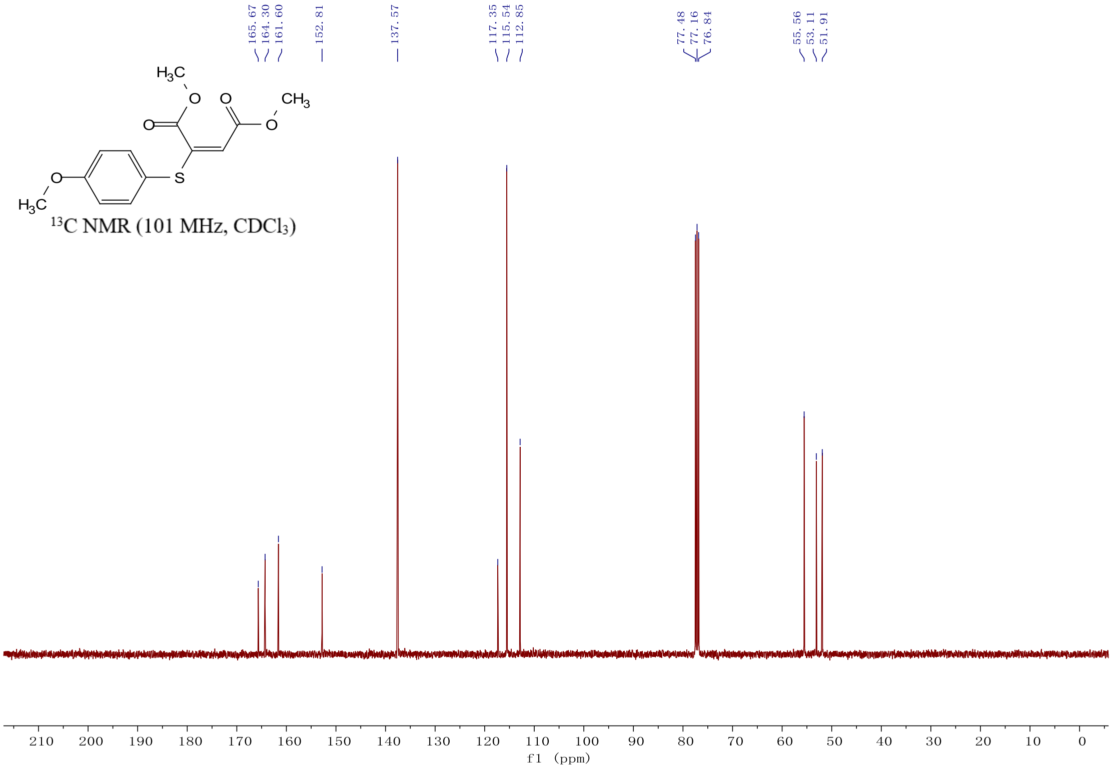

dimethyl 2-((3,5-dimethylphenyl)thio)fumarate (*Z*-1aj)

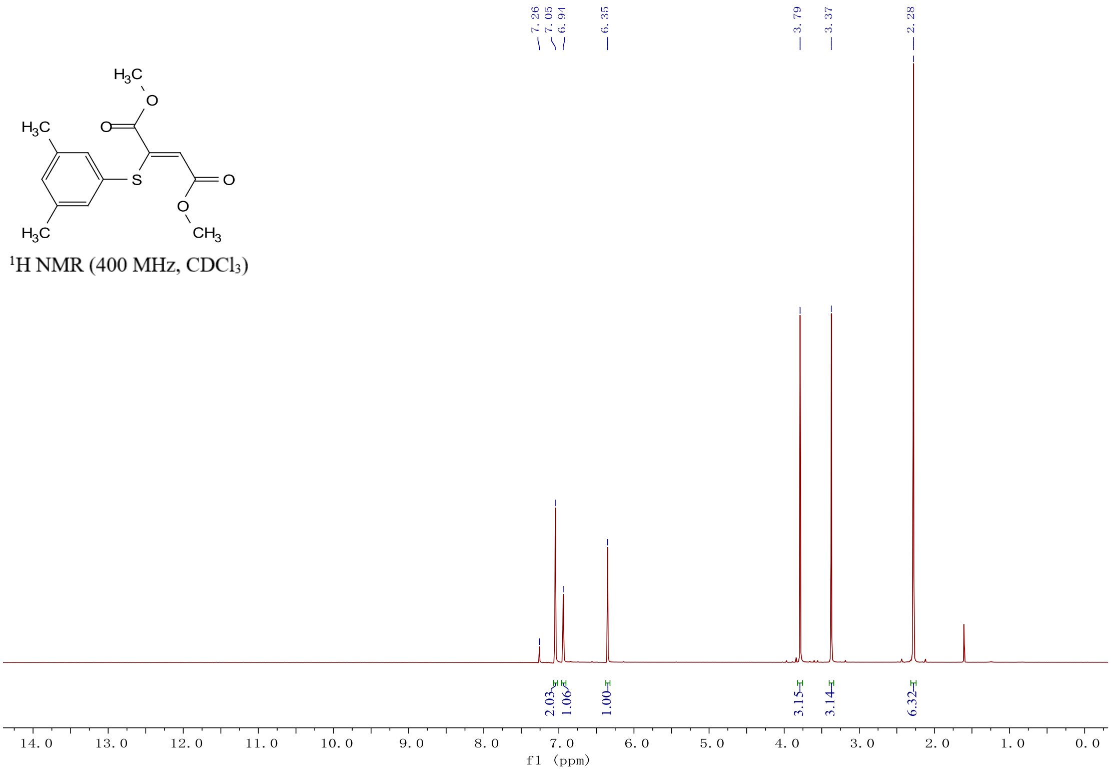

^{1}H NMR (400 MHz, $CDCl_3$)

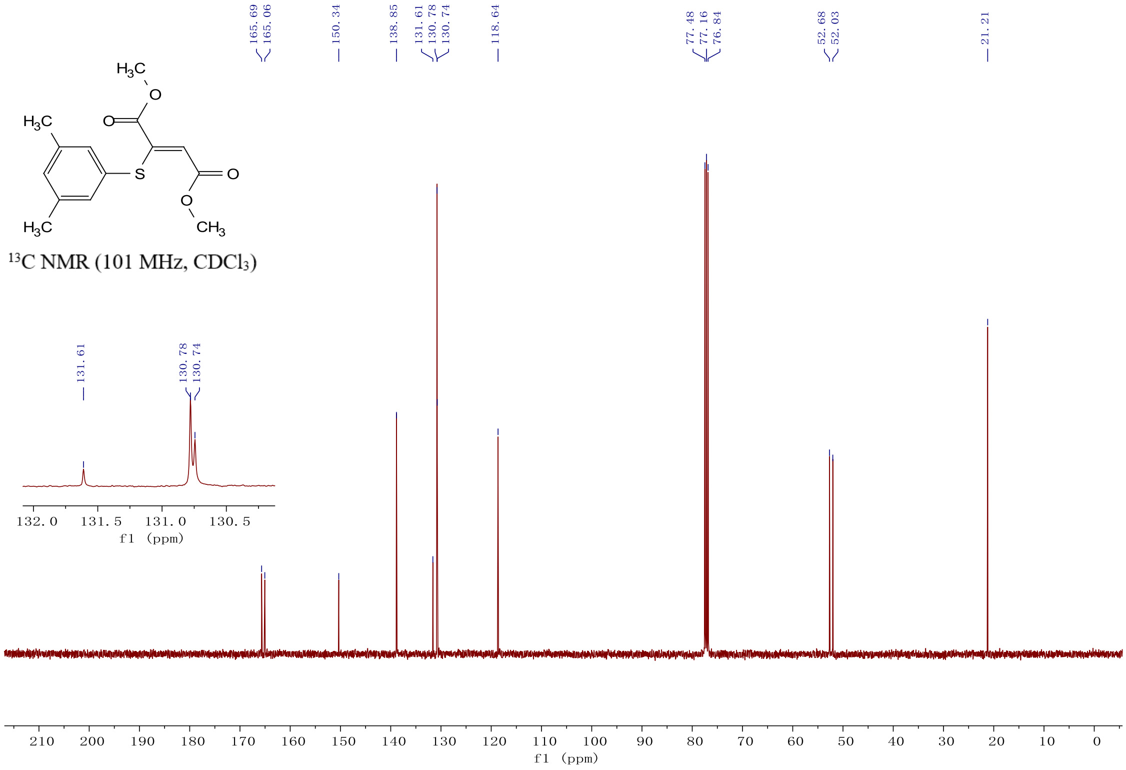

^{13}C NMR (101 MHz, $CDCl_3$)

dimethyl 2-((3,5-dimethylphenyl)thio)maleate (*E*-1aj)

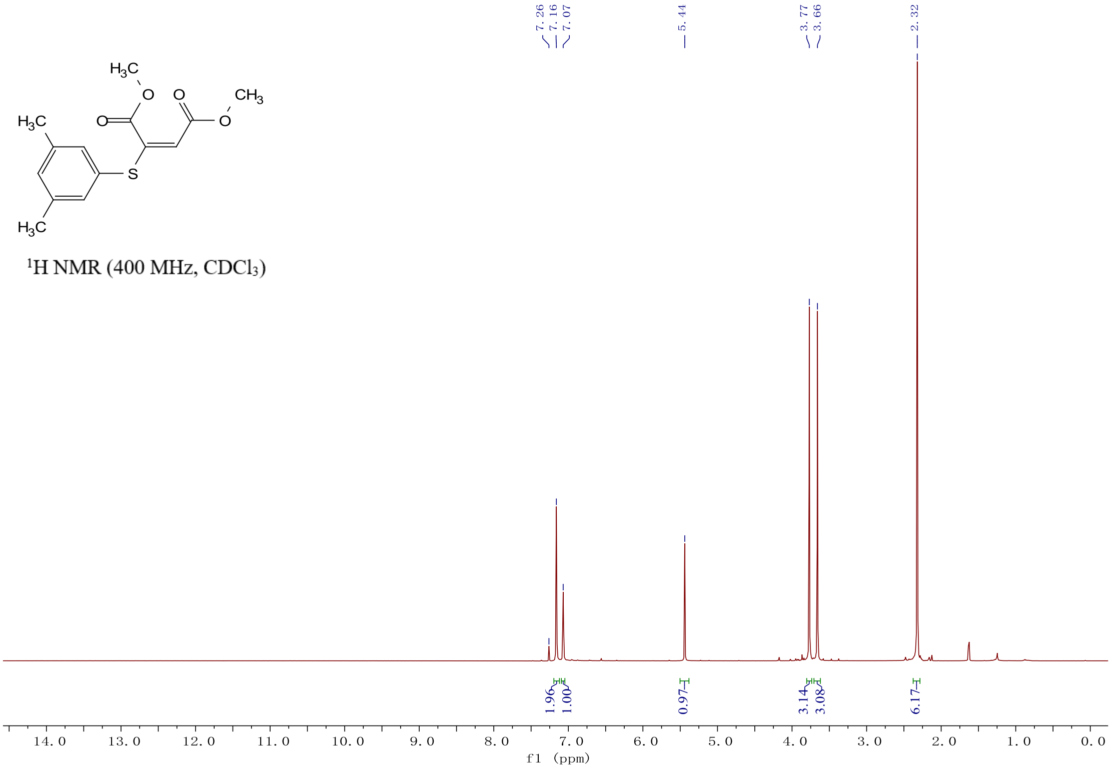

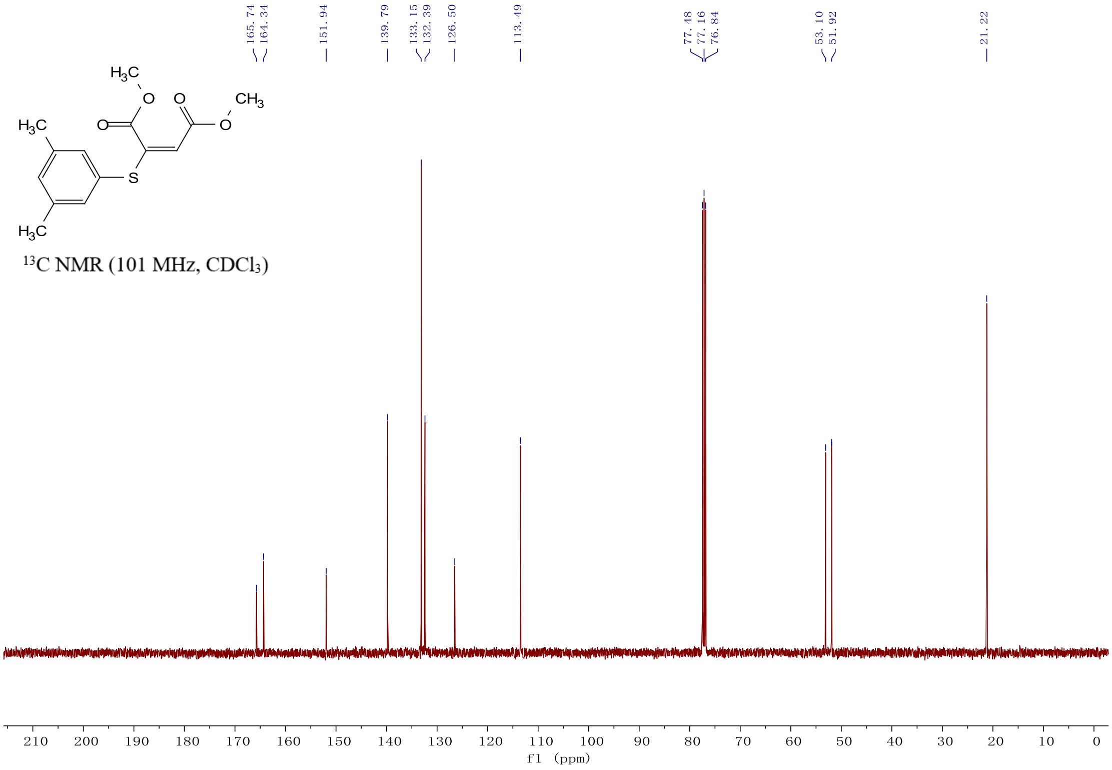

dimethyl 2-(naphthalen-1-ylthio)fumarate (*Z*-1ak)

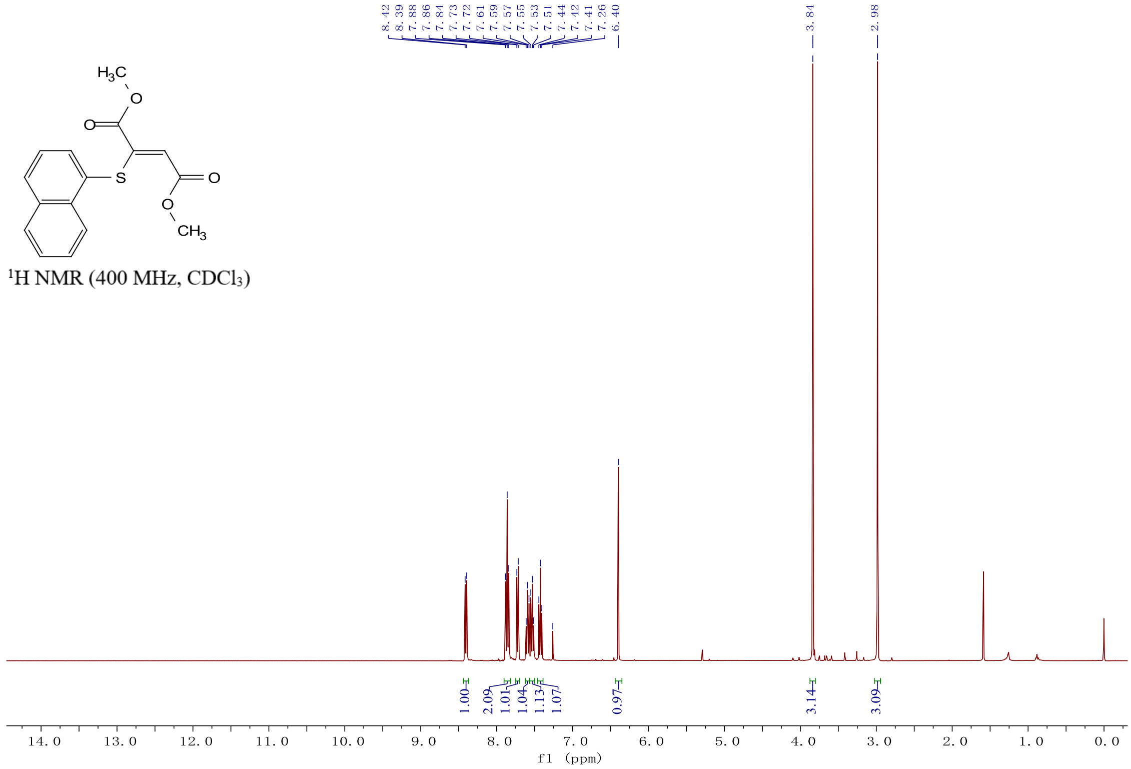

^{1}H NMR (400 MHz, $CDCl_3$)

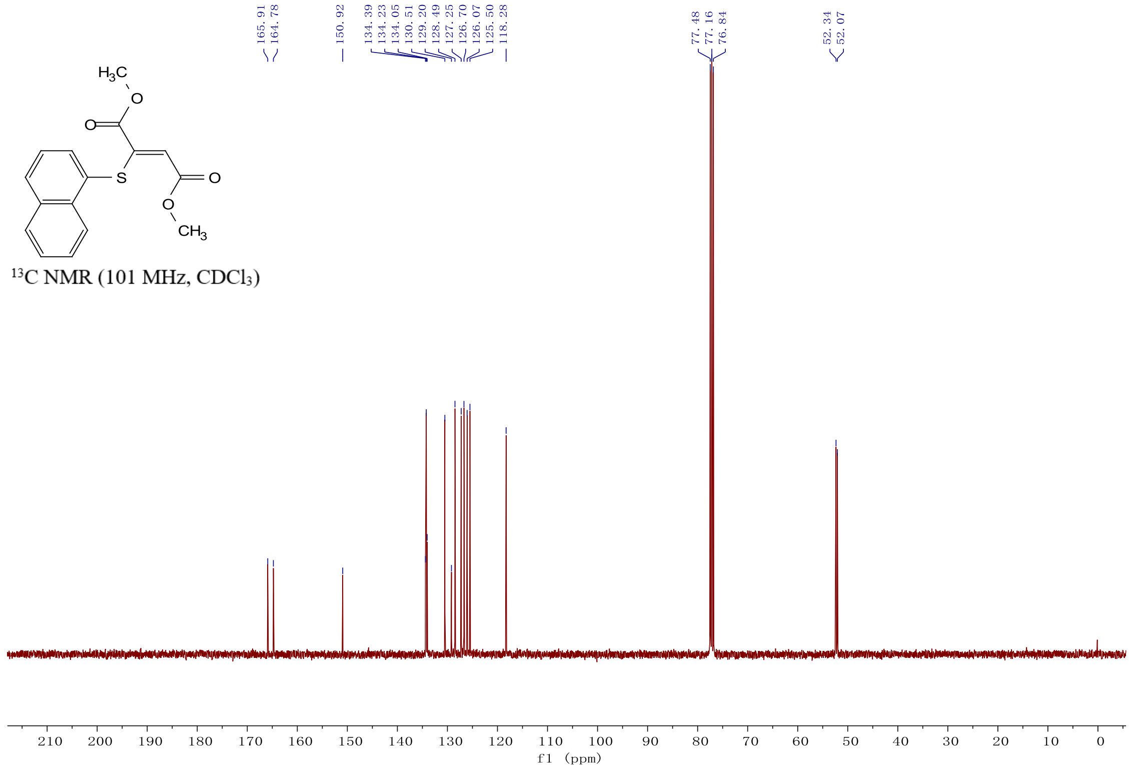

^{13}C NMR (101 MHz, $CDCl_3$)

dimethyl 2-(naphthalen-1-ylthio)maleate (*E*-1ak)

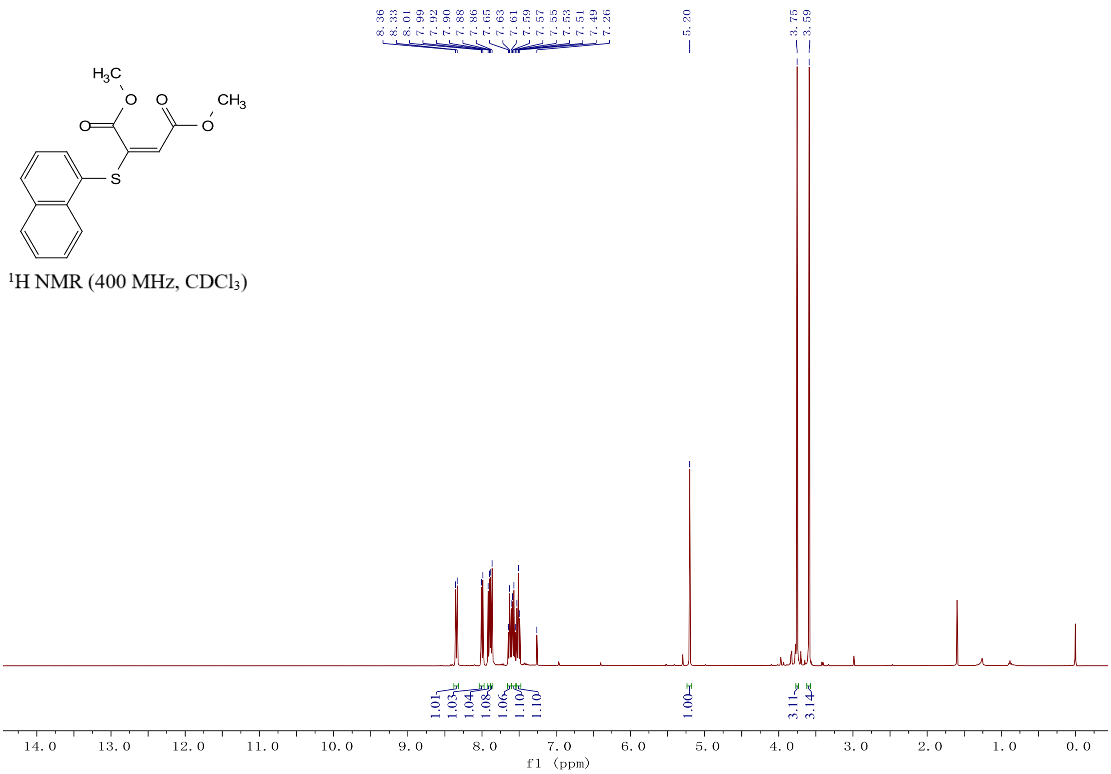

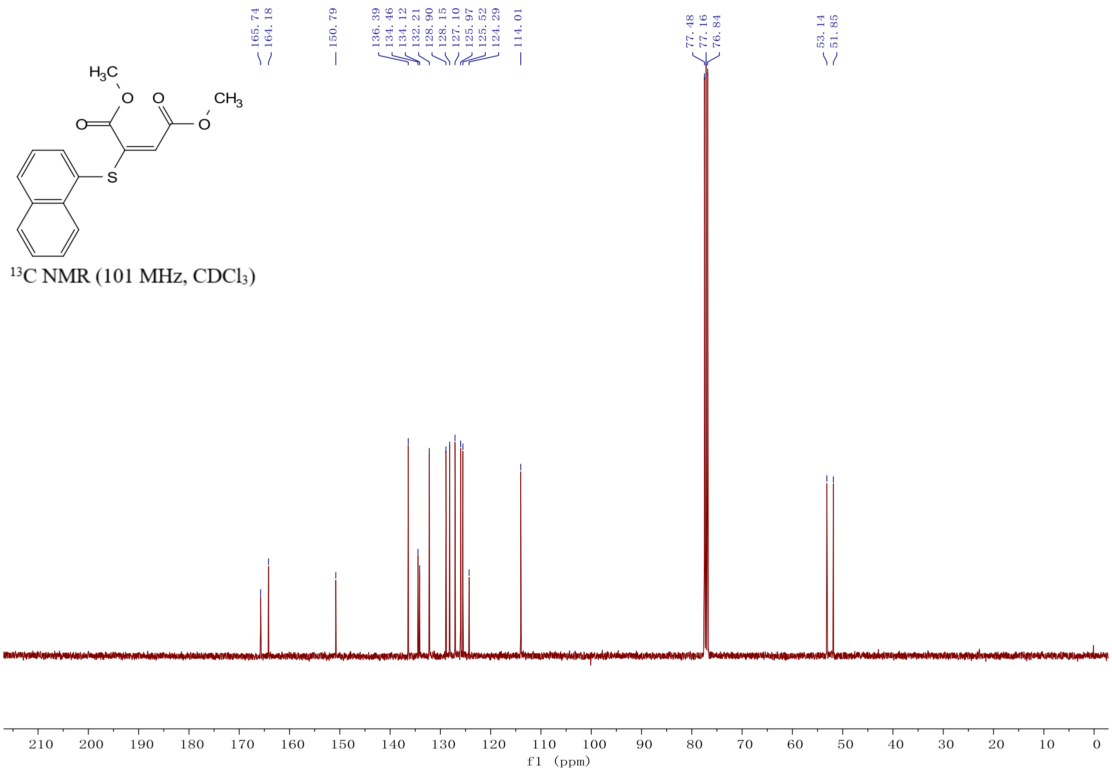

dimethyl 2-(naphthalen-2-ylthio)fumarate (*Z*-1al)

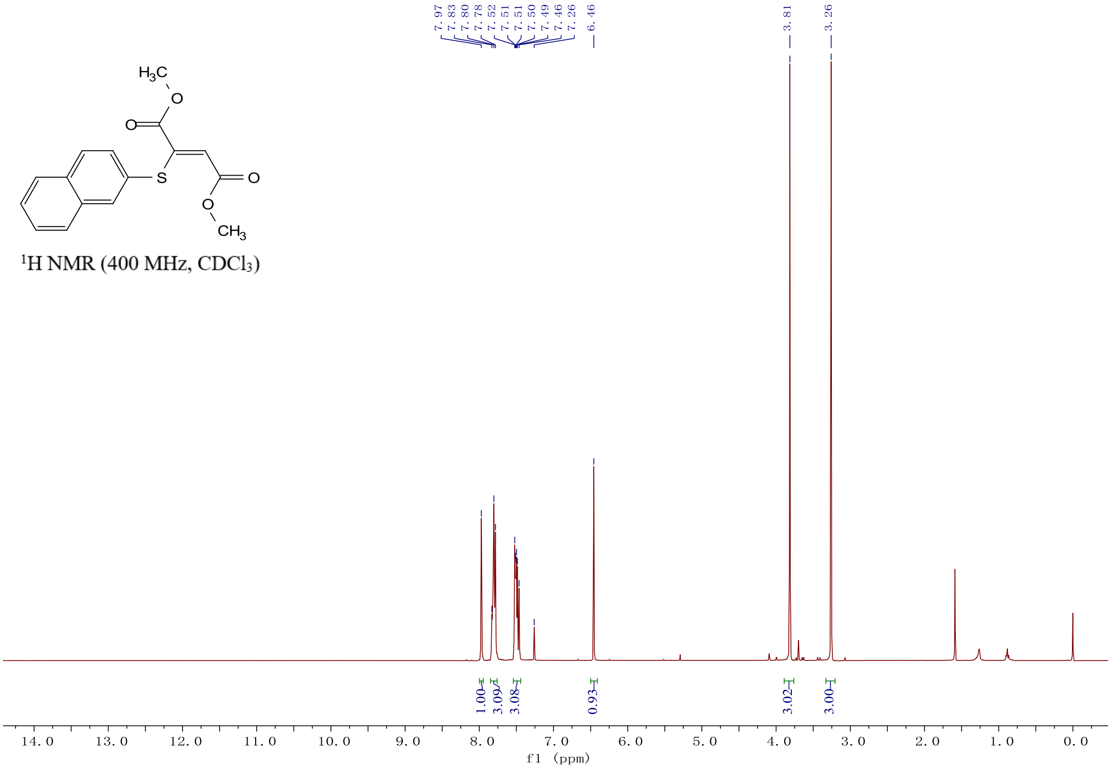

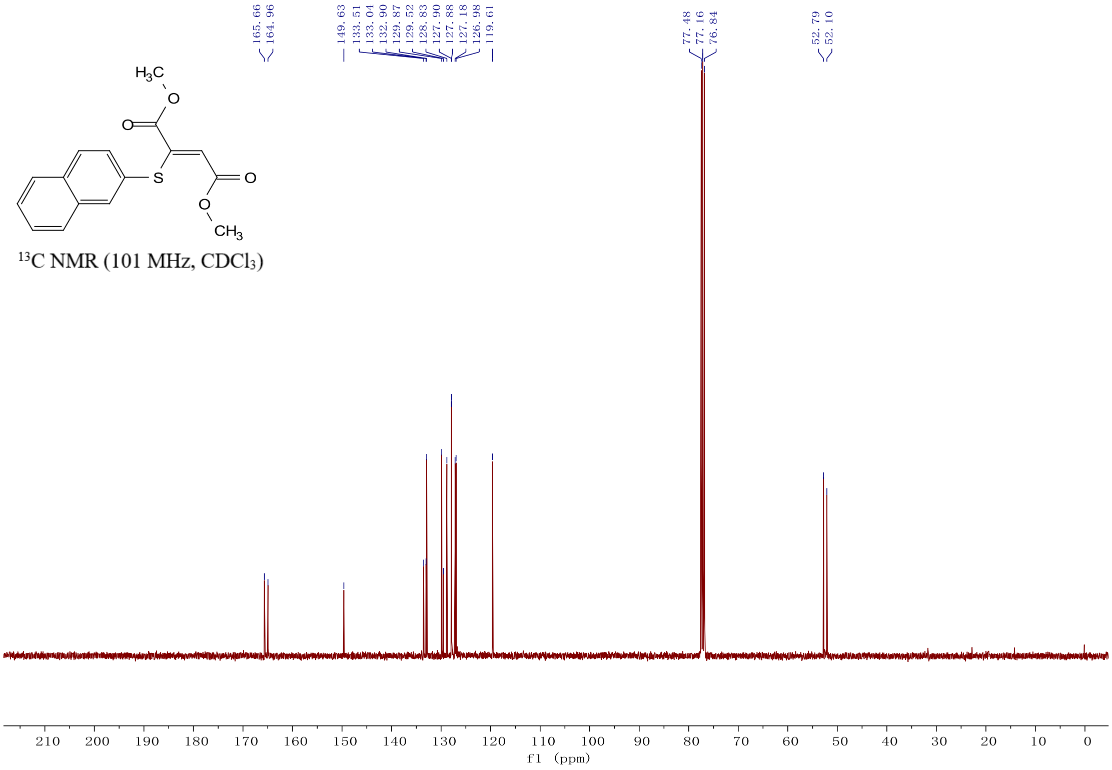

dimethyl 2-(naphthalen-2-ylthio)maleate (*E*-1al)

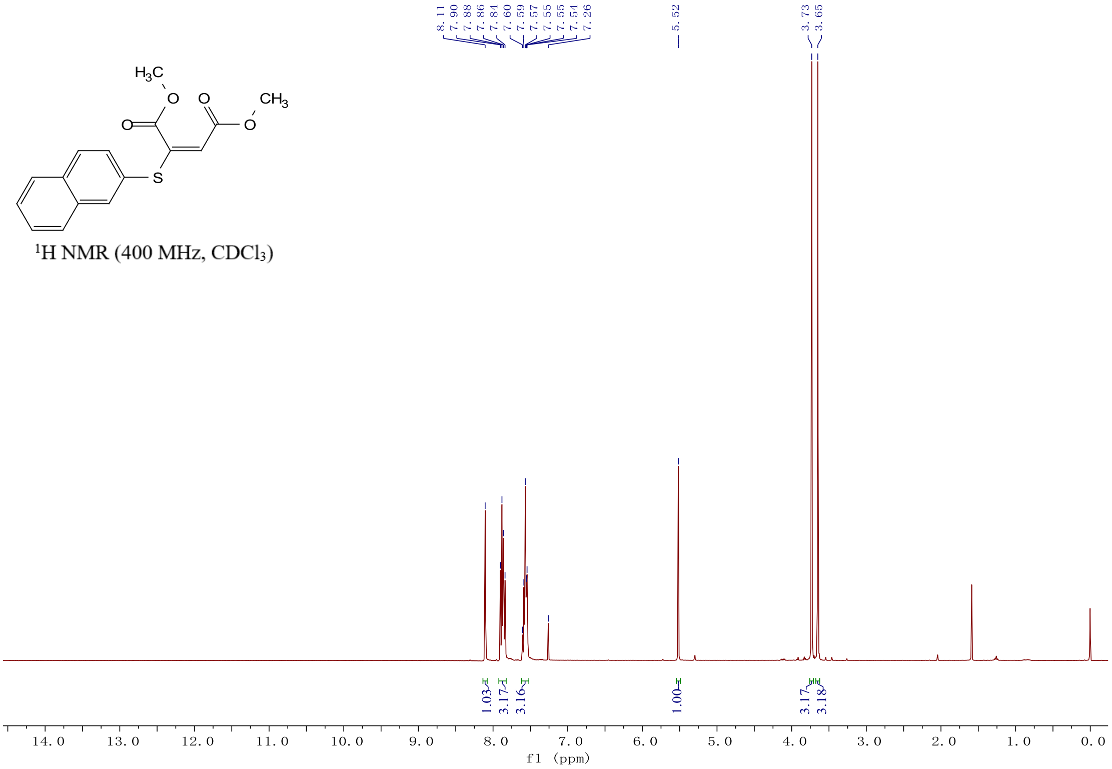

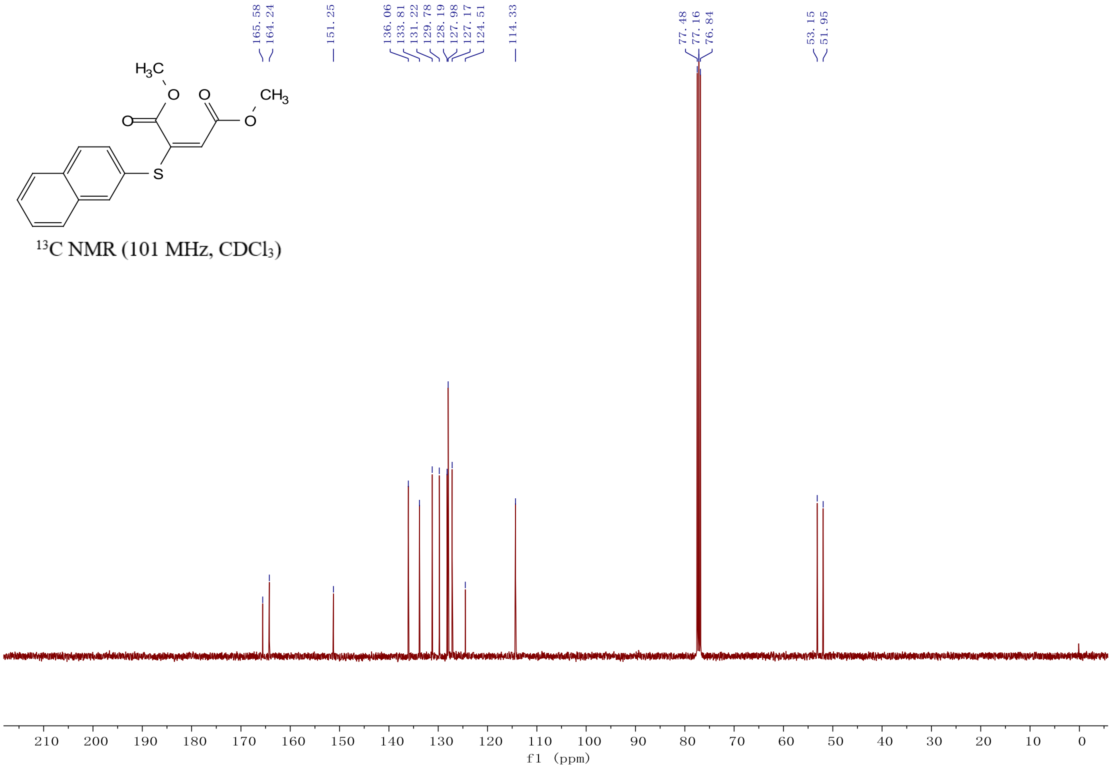

dimethyl 2-((furan-2-ylmethyl)thio)fumarate (*Z*-1am)

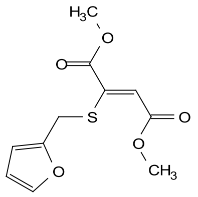

^{1}H NMR (400 MHz, $CDCl_3$)

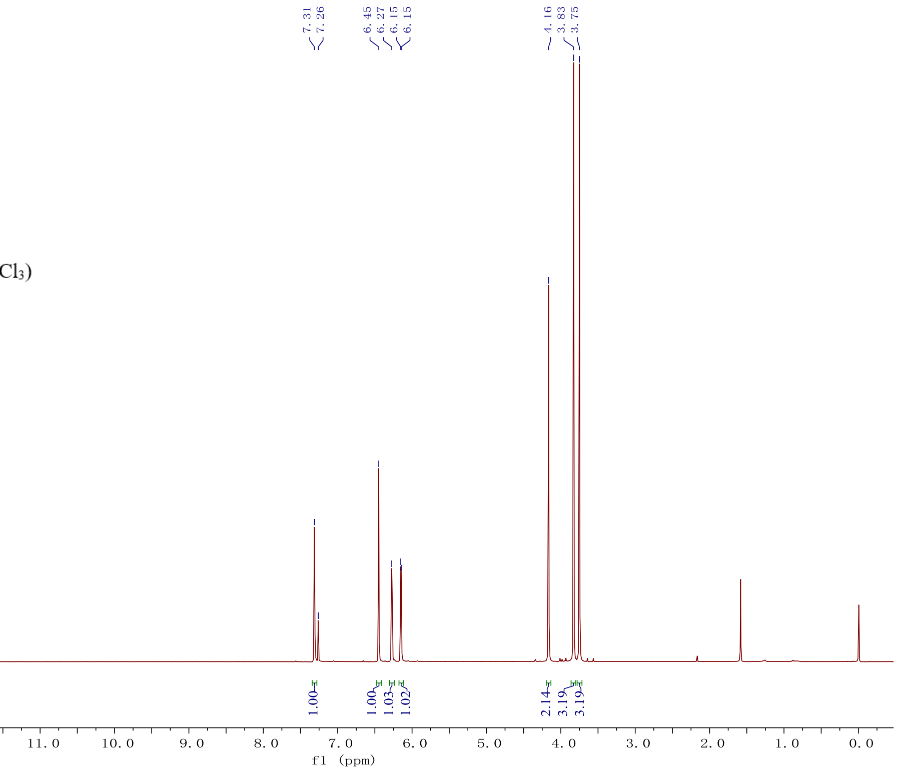

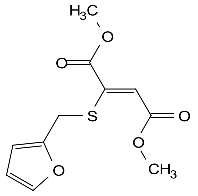

^{13}C NMR (101 MHz, $CDCl_3$)

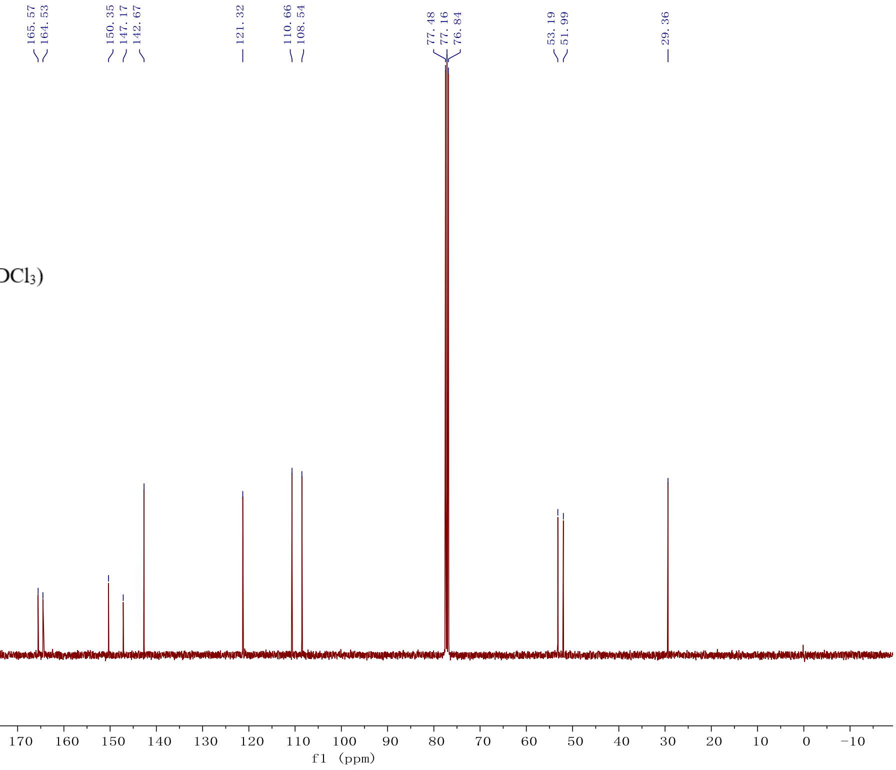

dimethyl 2-((furan-2-ylmethyl)thio)maleate (*E*-1am)

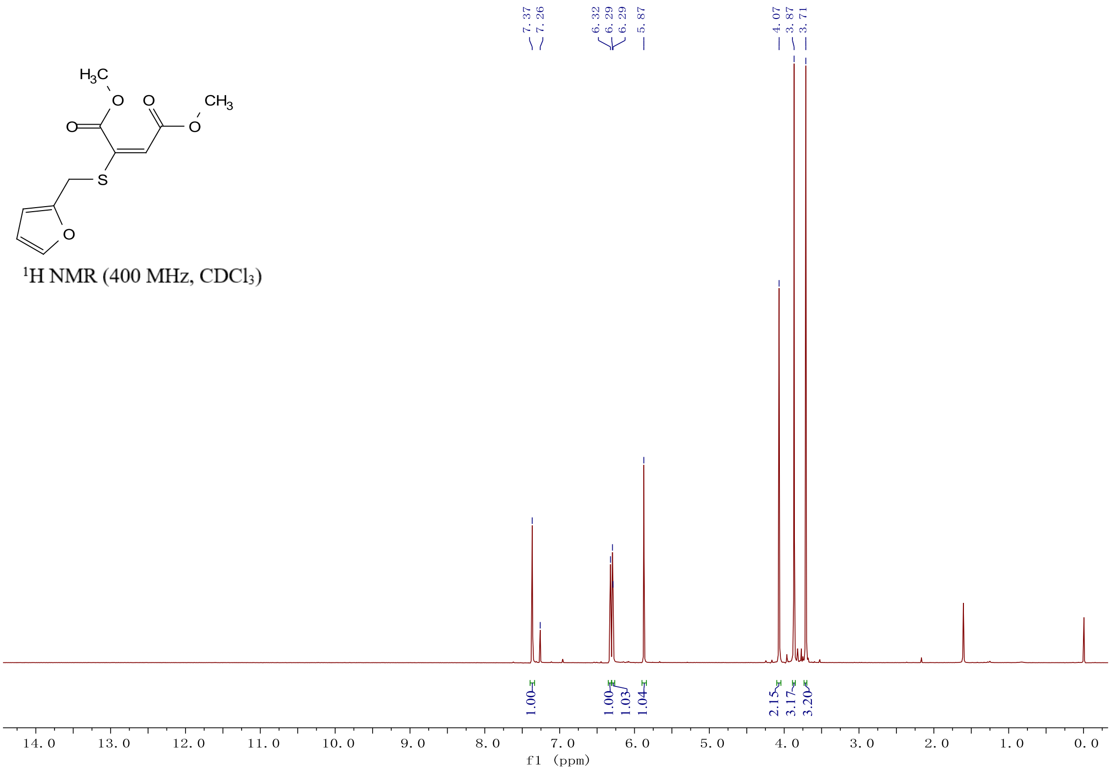

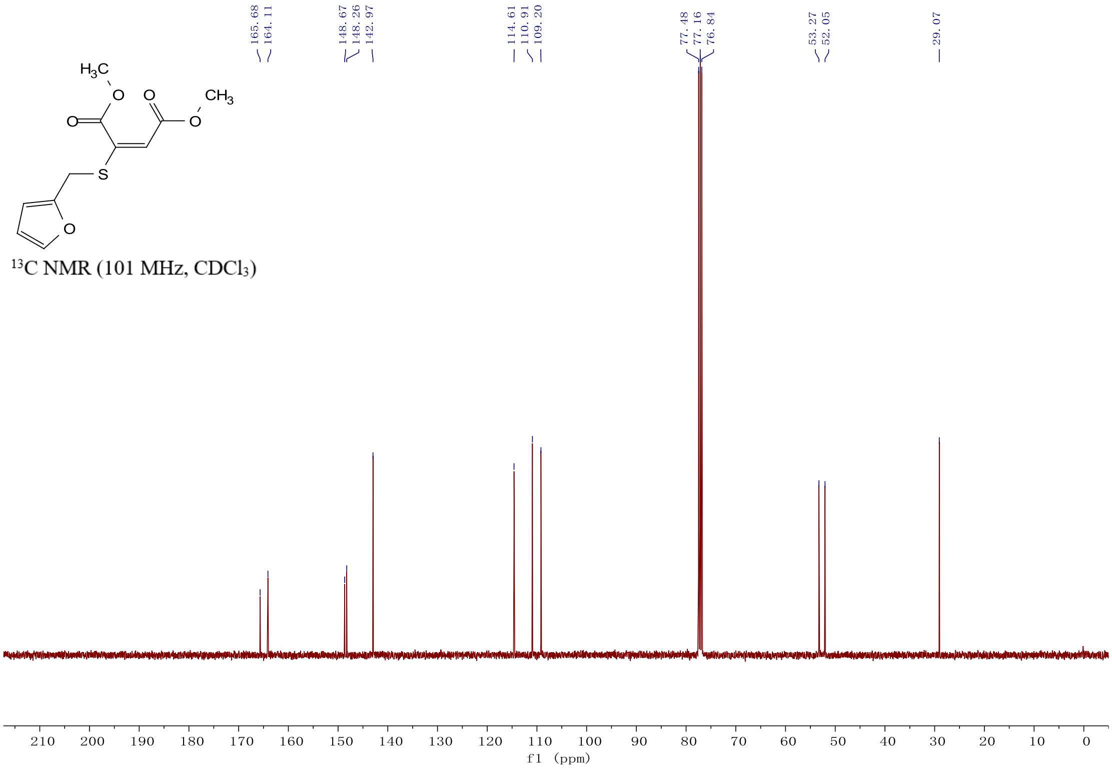

dimethyl 2-(benzylthio)fumarate (*Z*-1an)

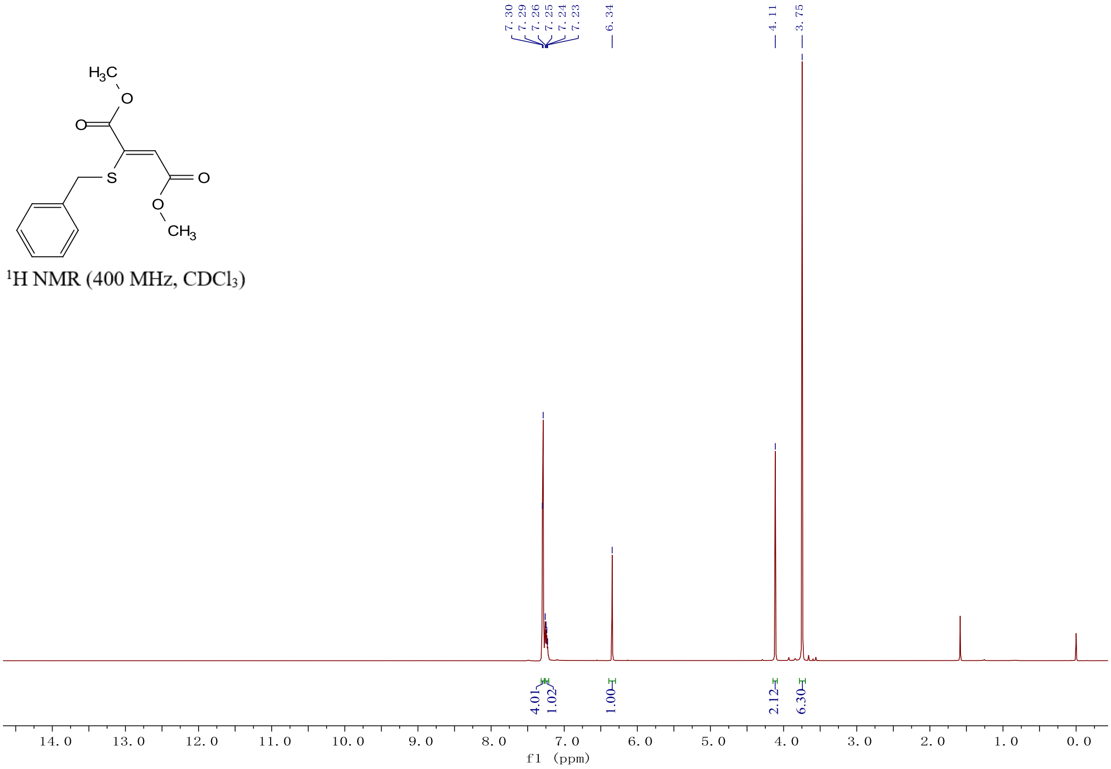

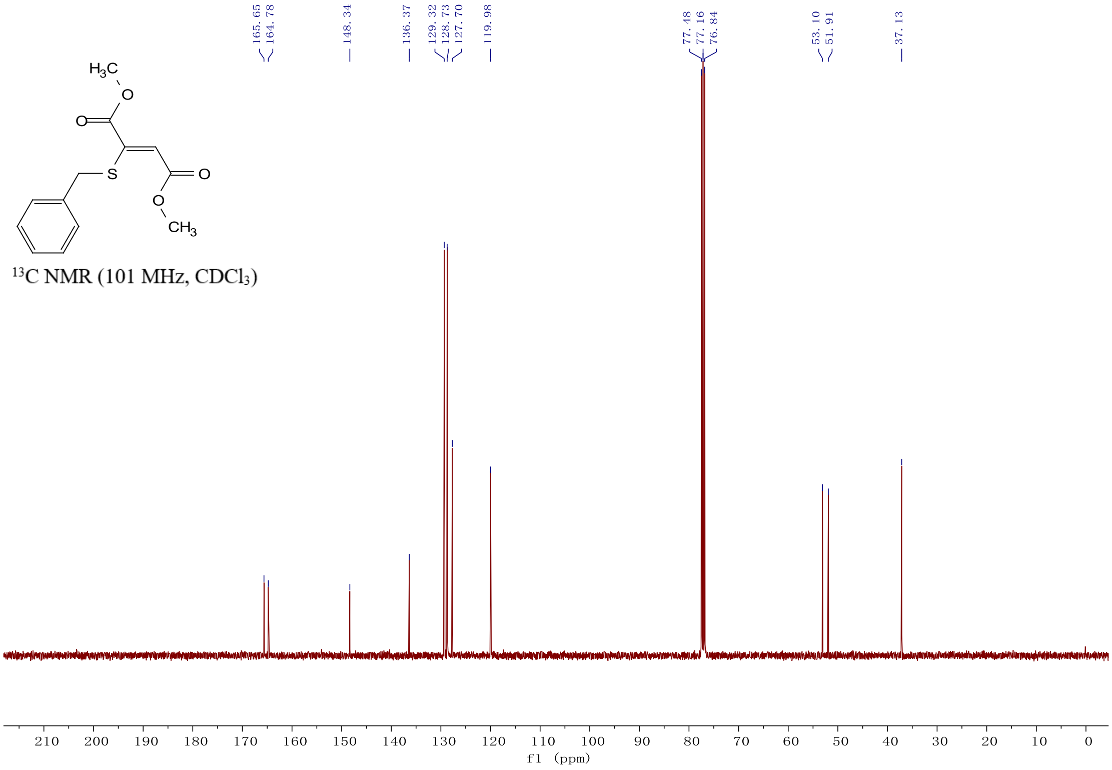

dimethyl 2-(benzylthio)maleate (*E*-1an)

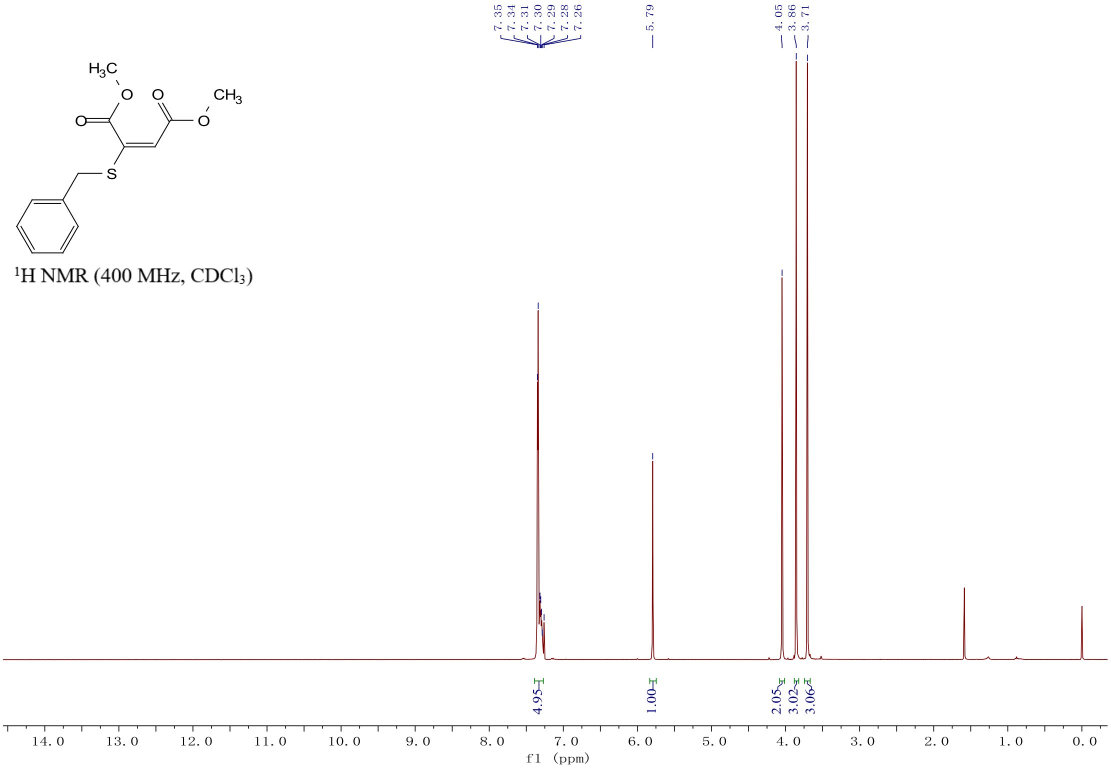

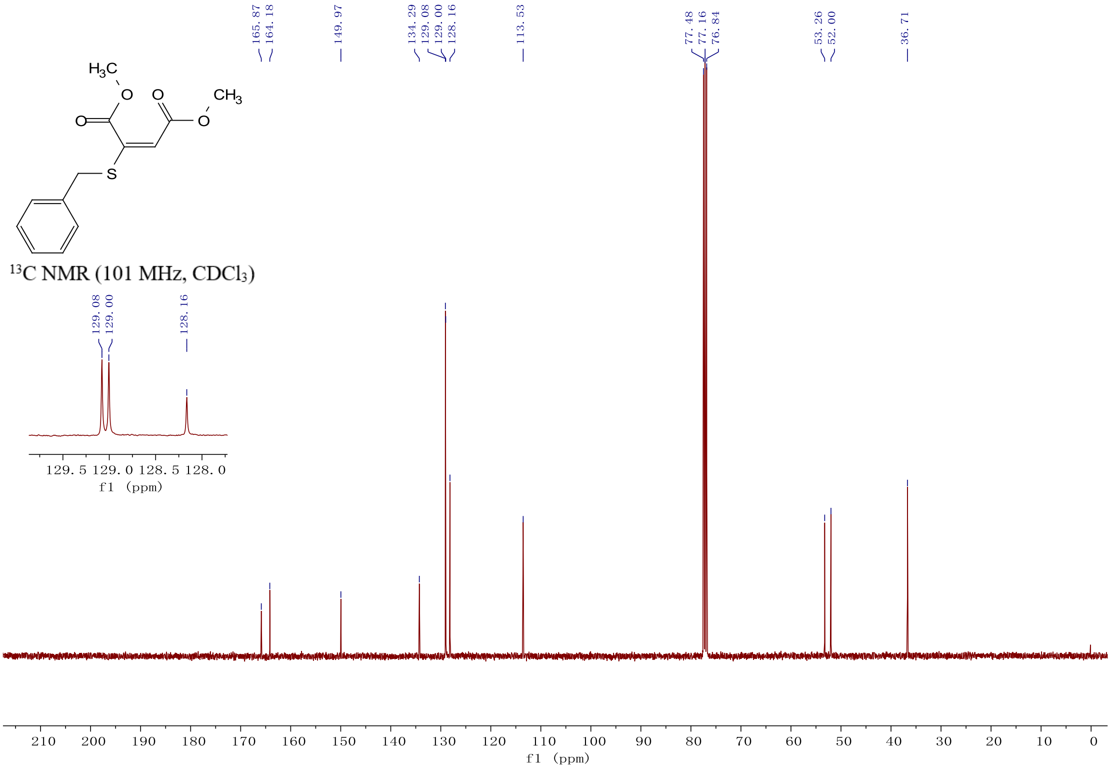

dimethyl 2-((4-methylbenzyl)thio)fumarate (*Z*-1ao)

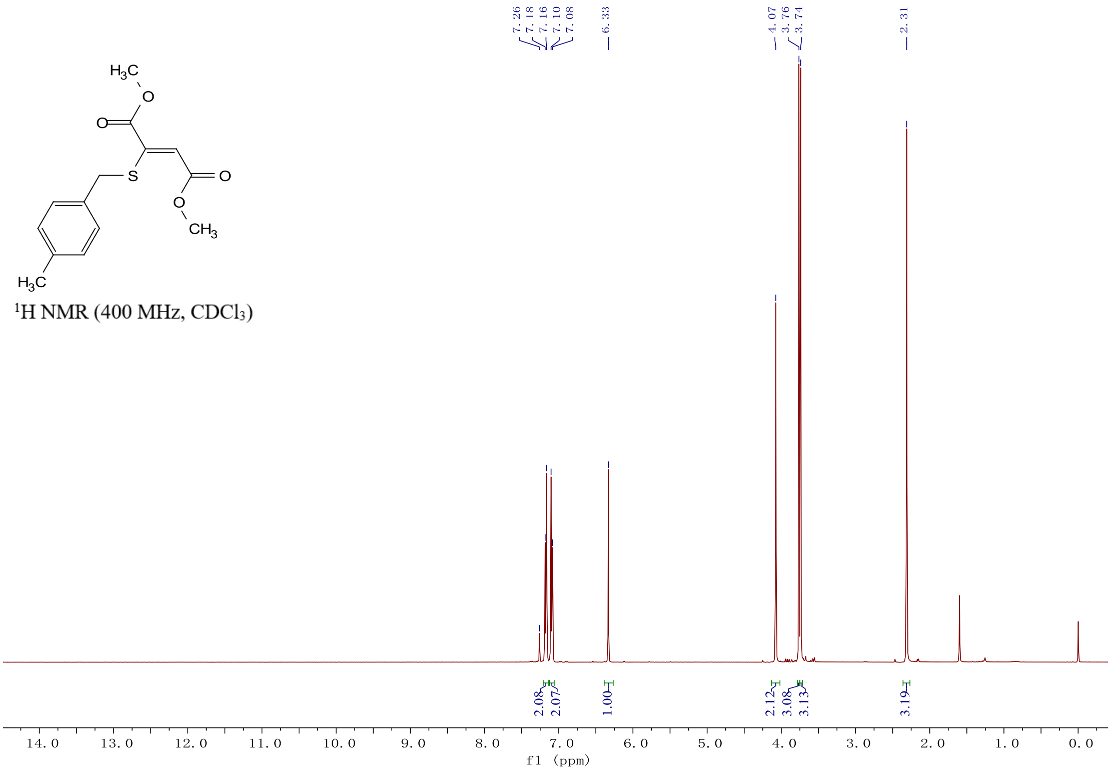

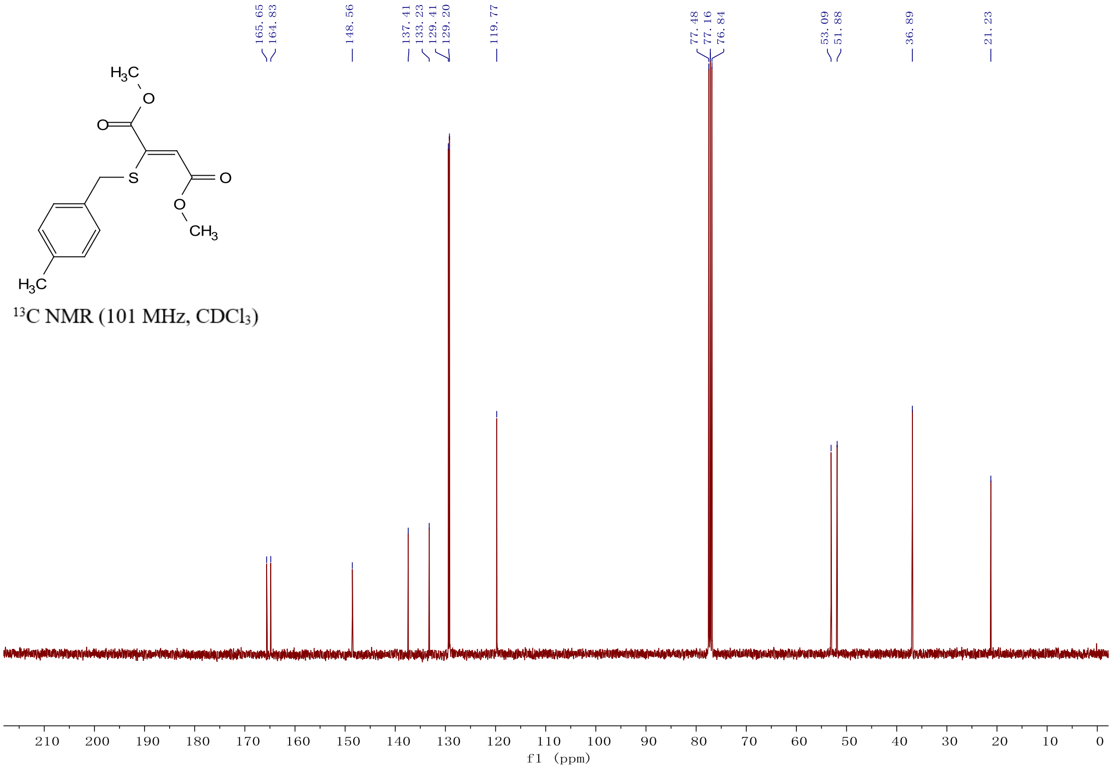

dimethyl 2-((4-methylbenzyl)thio)maleate (*E*-1ao)

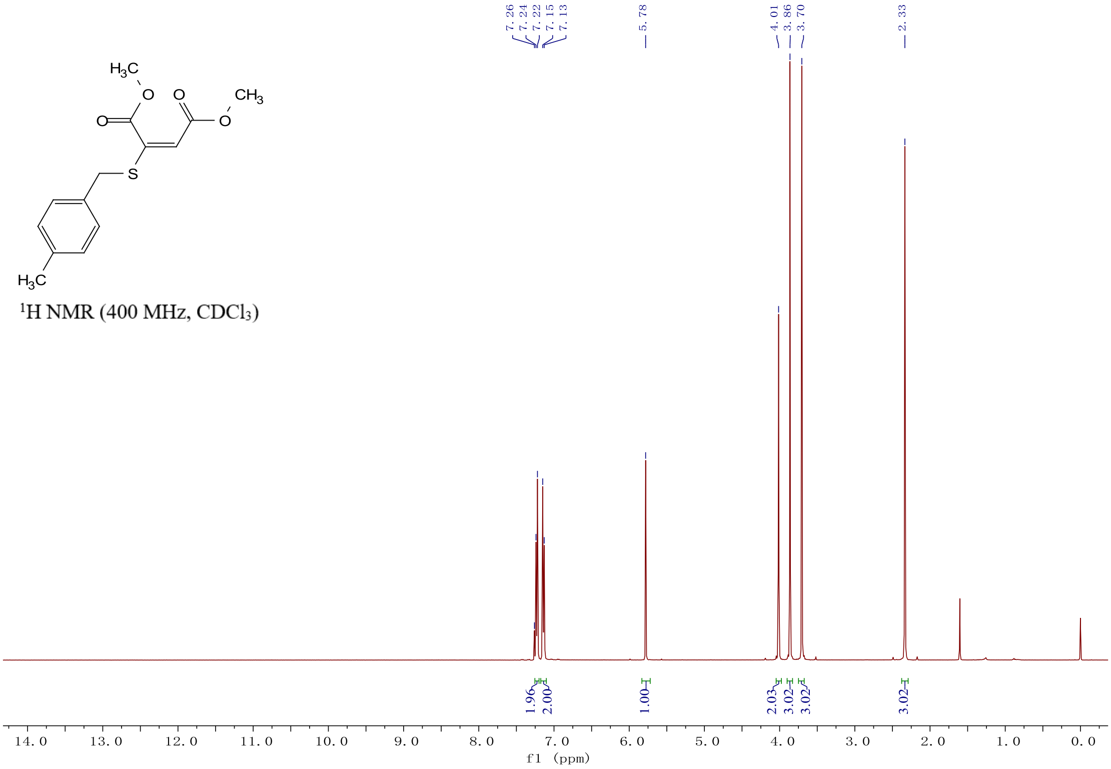

^{1}H NMR (400 MHz, CDCl$_3$)

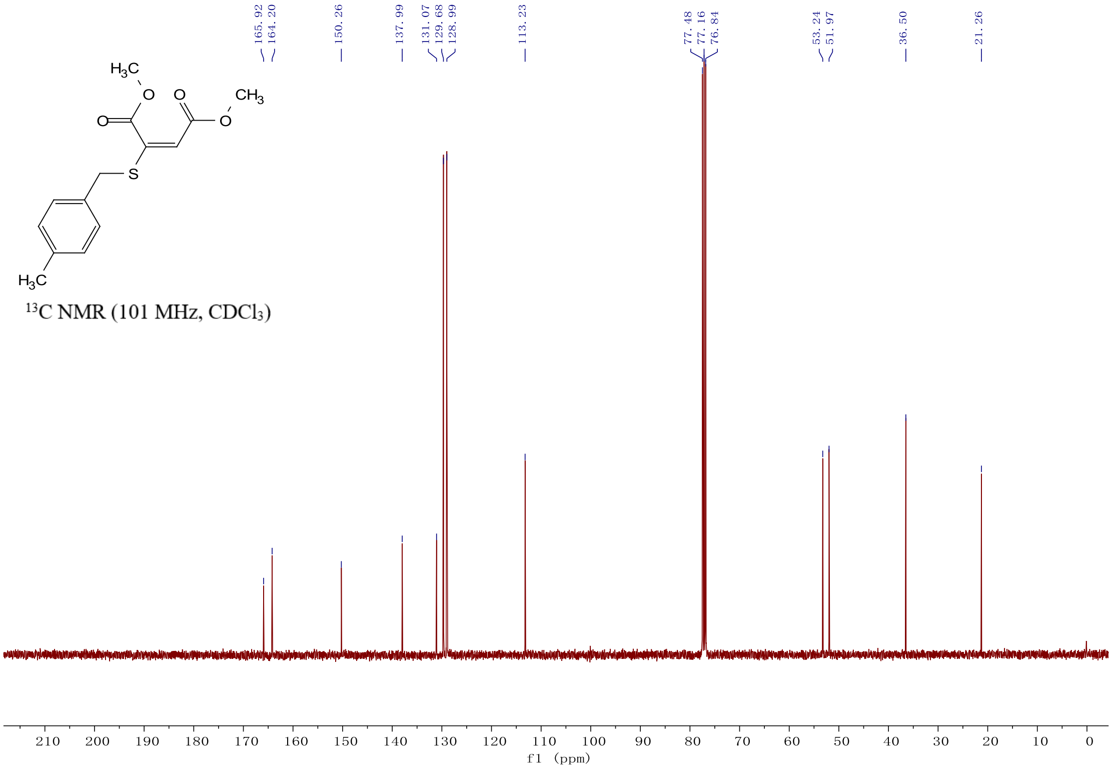

^{13}C NMR (101 MHz, CDCl$_3$)

dimethyl 2-((4-methoxybenzyl)thio)fumarate (*Z*-1ap)

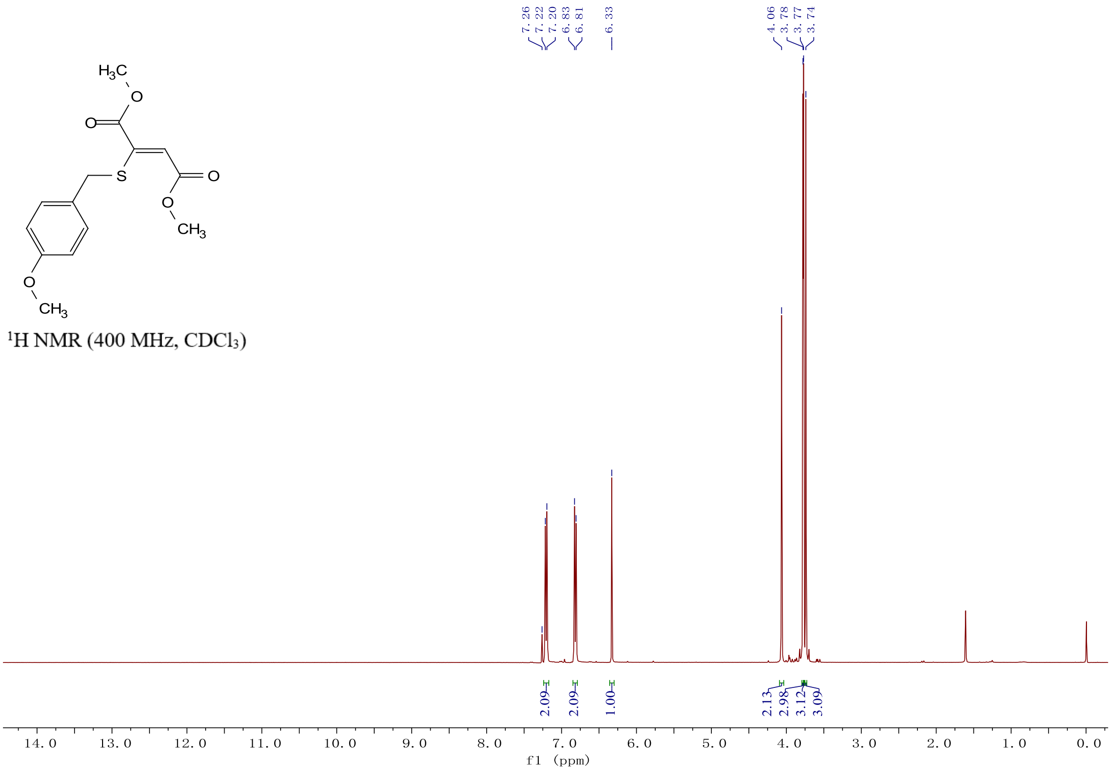

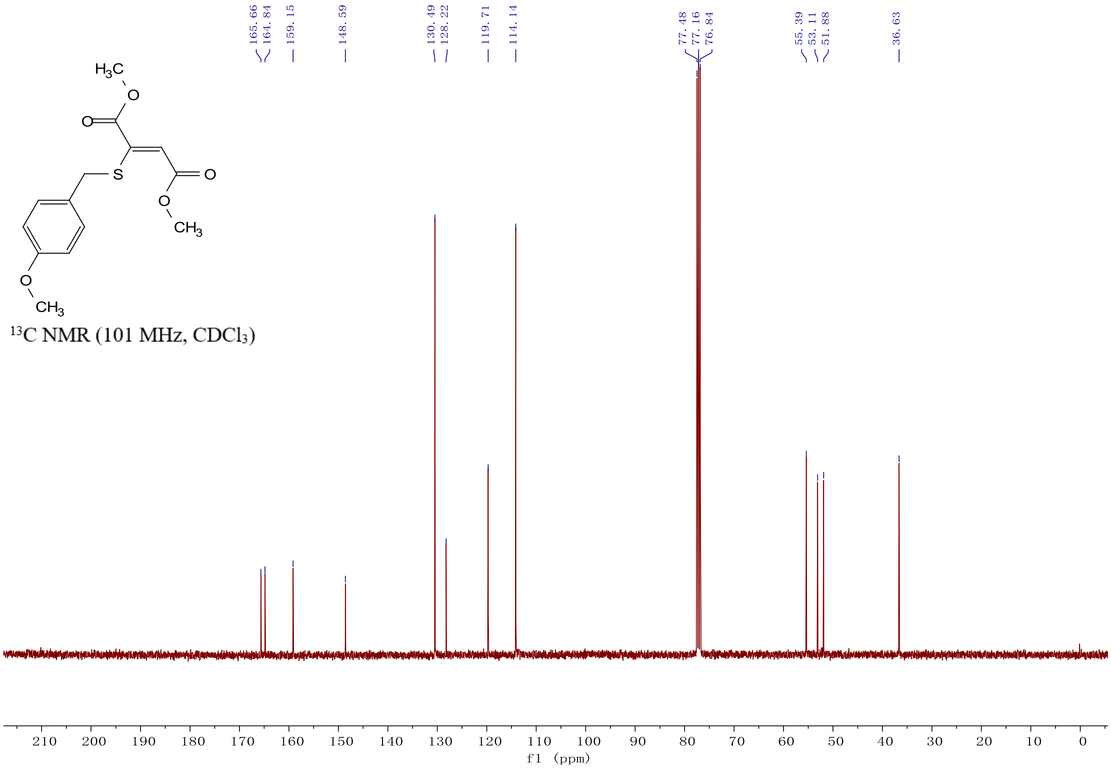

dimethyl 2-((4-methoxybenzyl)thio)maleate (*E*-1ap)

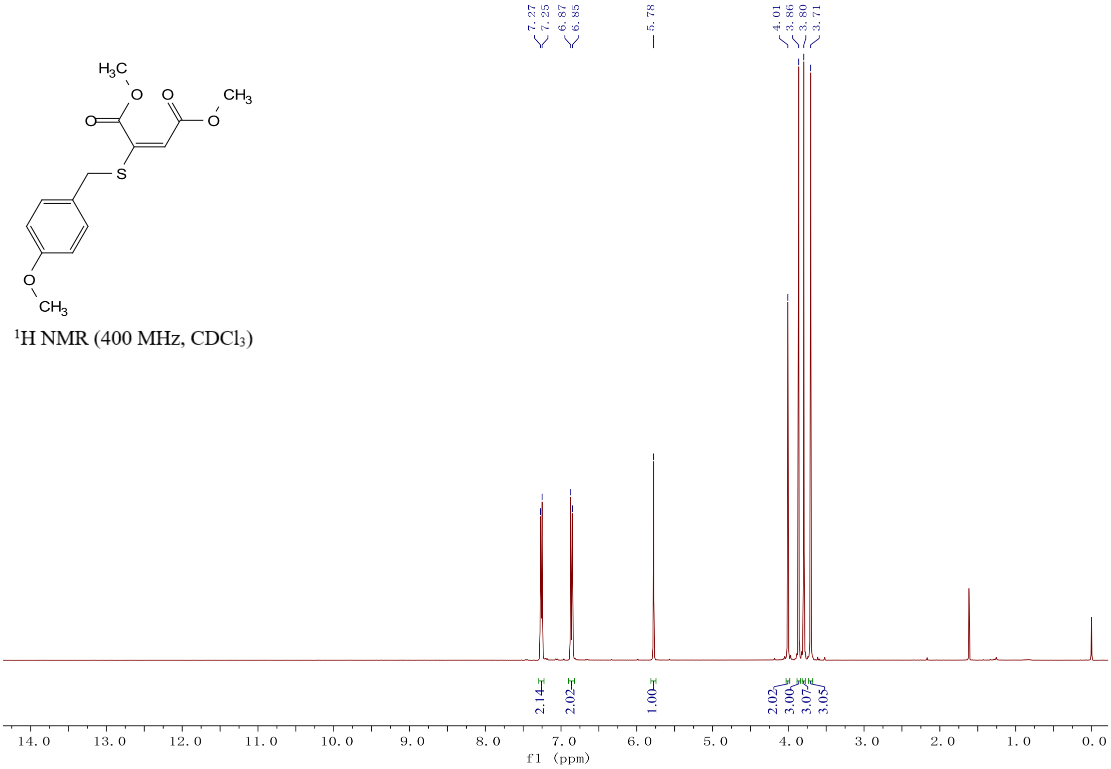

^{1}H NMR (400 MHz, $CDCl_3$)

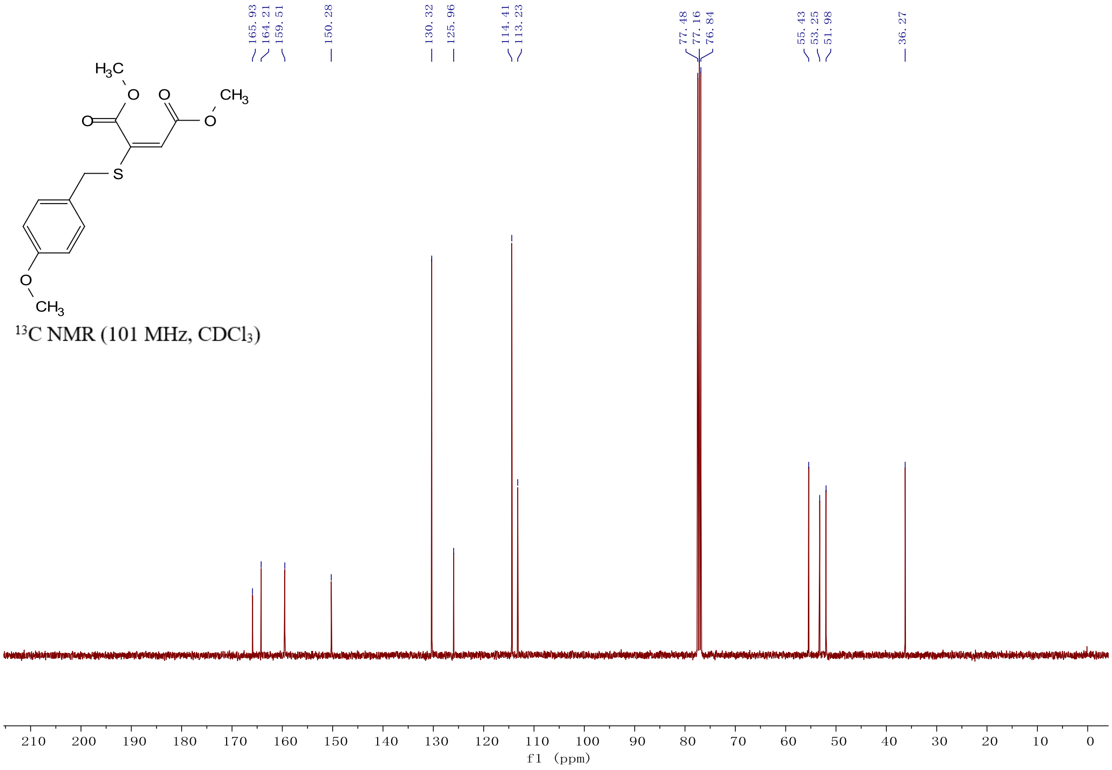

^{13}C NMR (101 MHz, $CDCl_3$)

dimethyl 2-(phenethylthio)fumarate (*Z*-1aq)

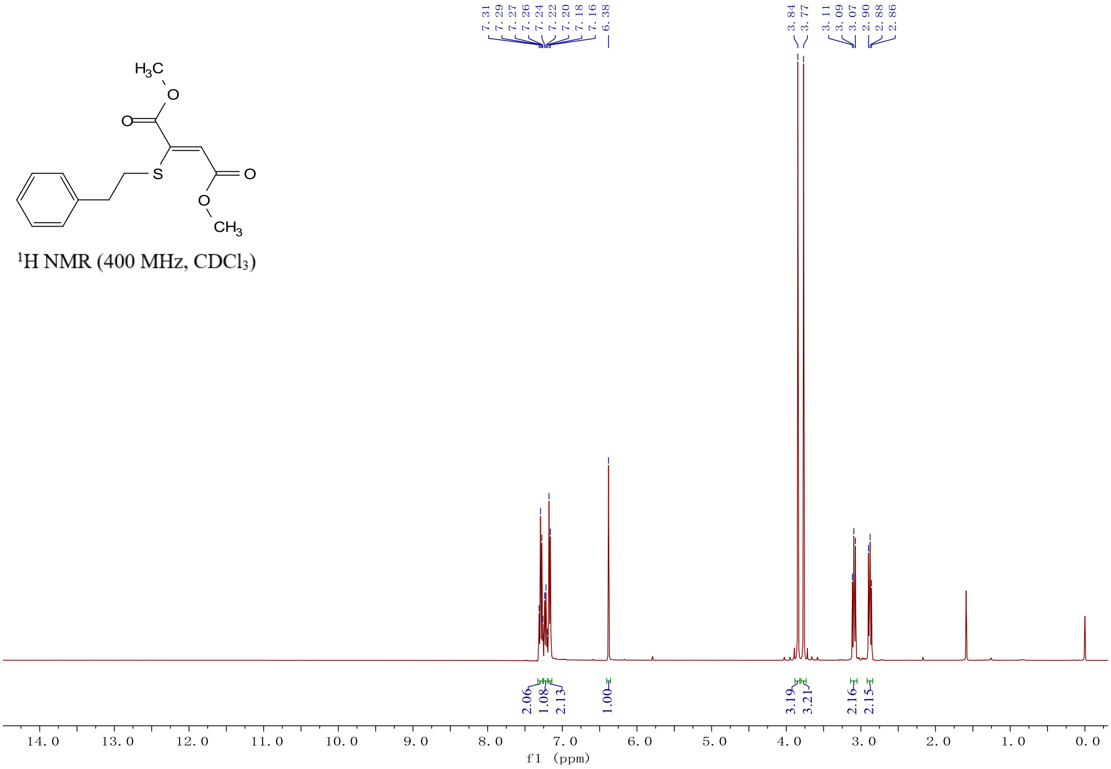

^{1}H NMR (400 MHz, $CDCl_3$)

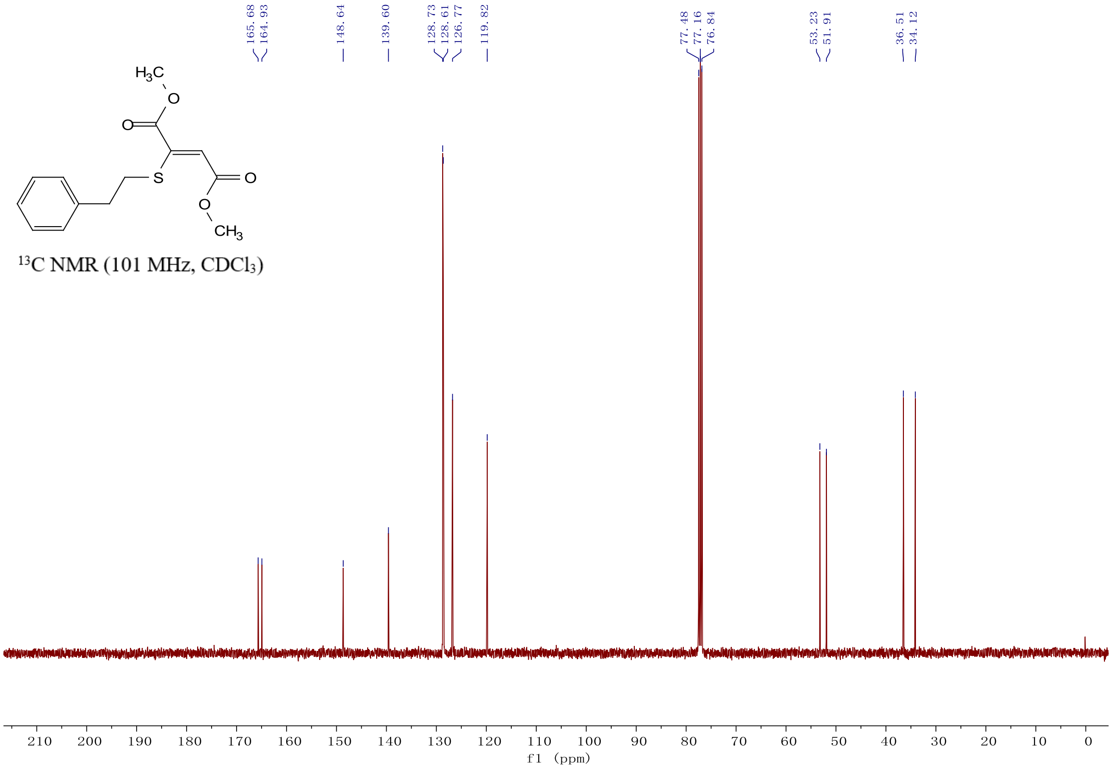

^{13}C NMR (101 MHz, $CDCl_3$)

dimethyl 2-(phenethylthio)maleate (*E*-1aq)

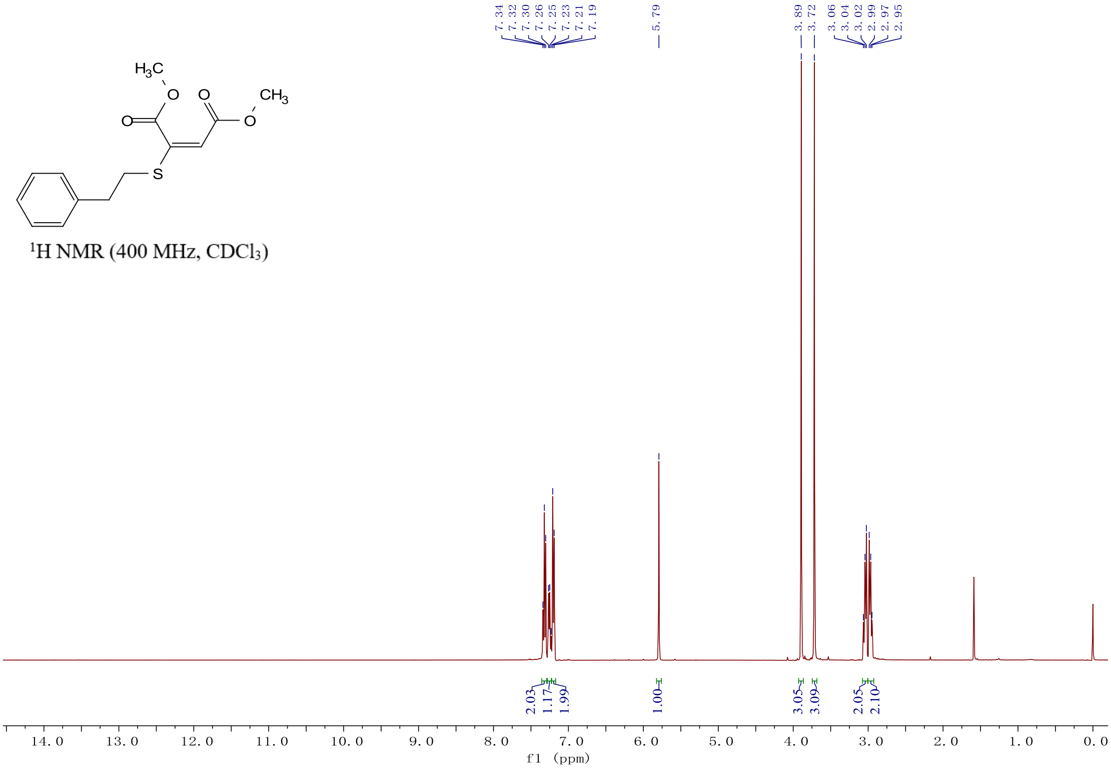

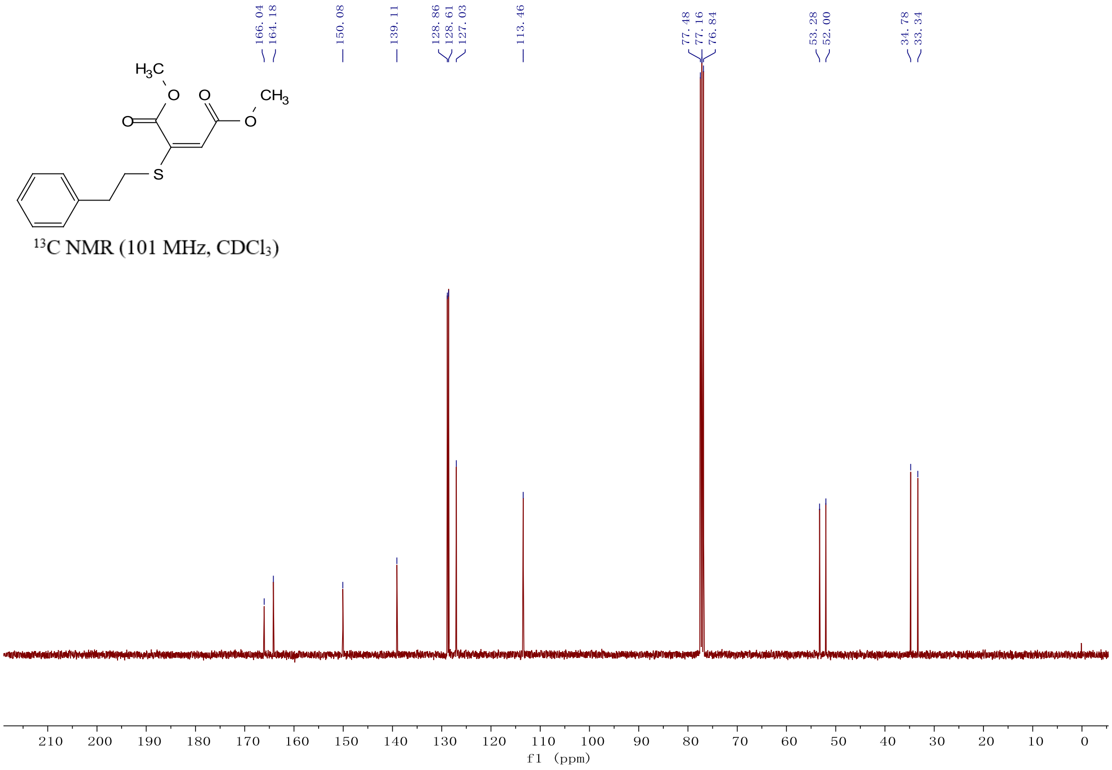

dimethyl 2-(pentylthio)fumarate (*Z*-1ar)

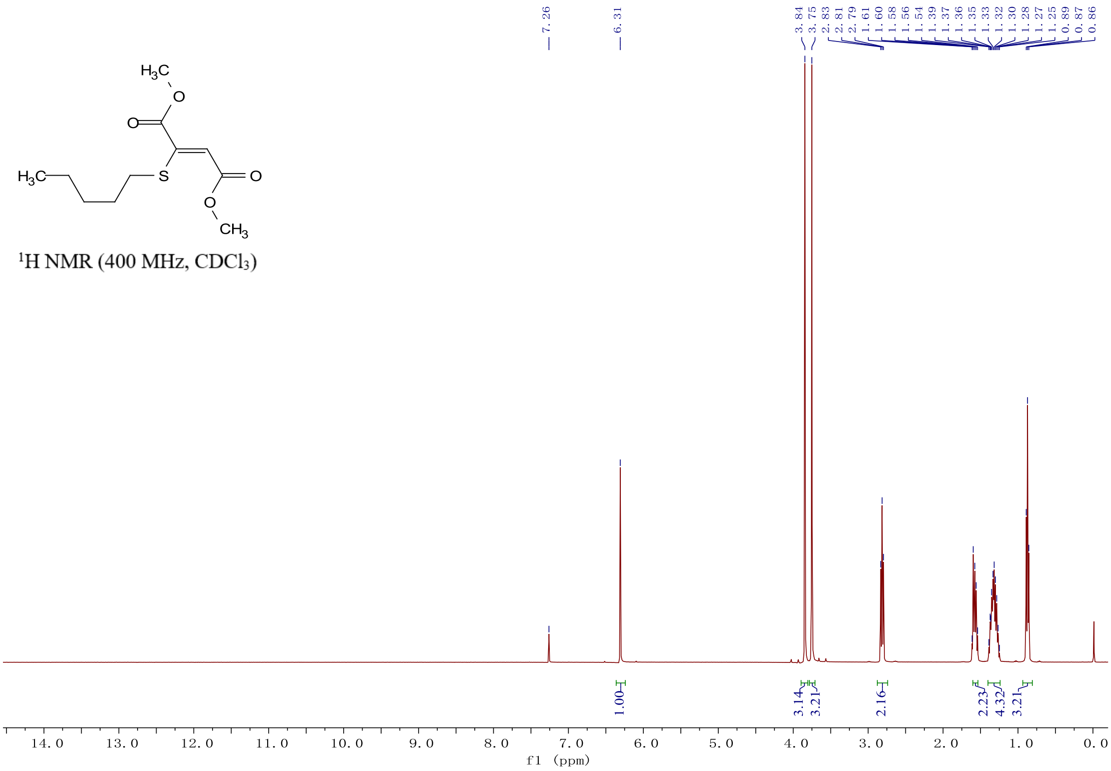

^{1}H NMR (400 MHz, $CDCl_3$)

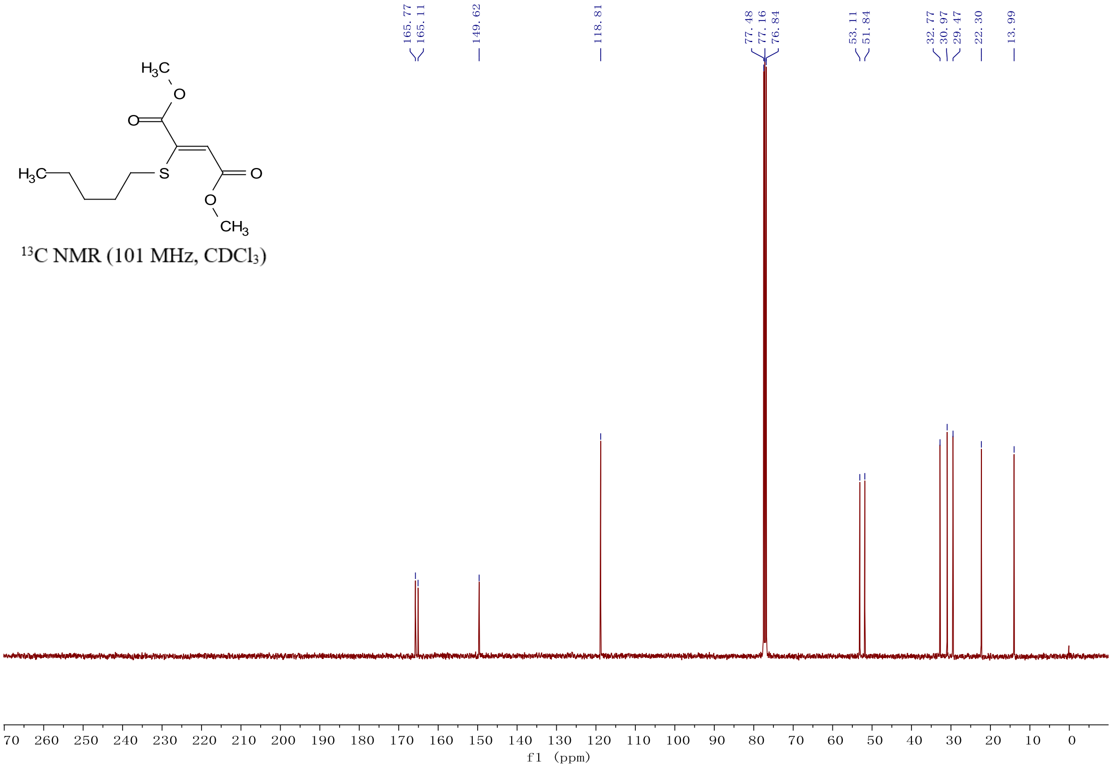

^{13}C NMR (101 MHz, $CDCl_3$)

dimethyl 2-(pentylthio)maleate (*E*-1ar)

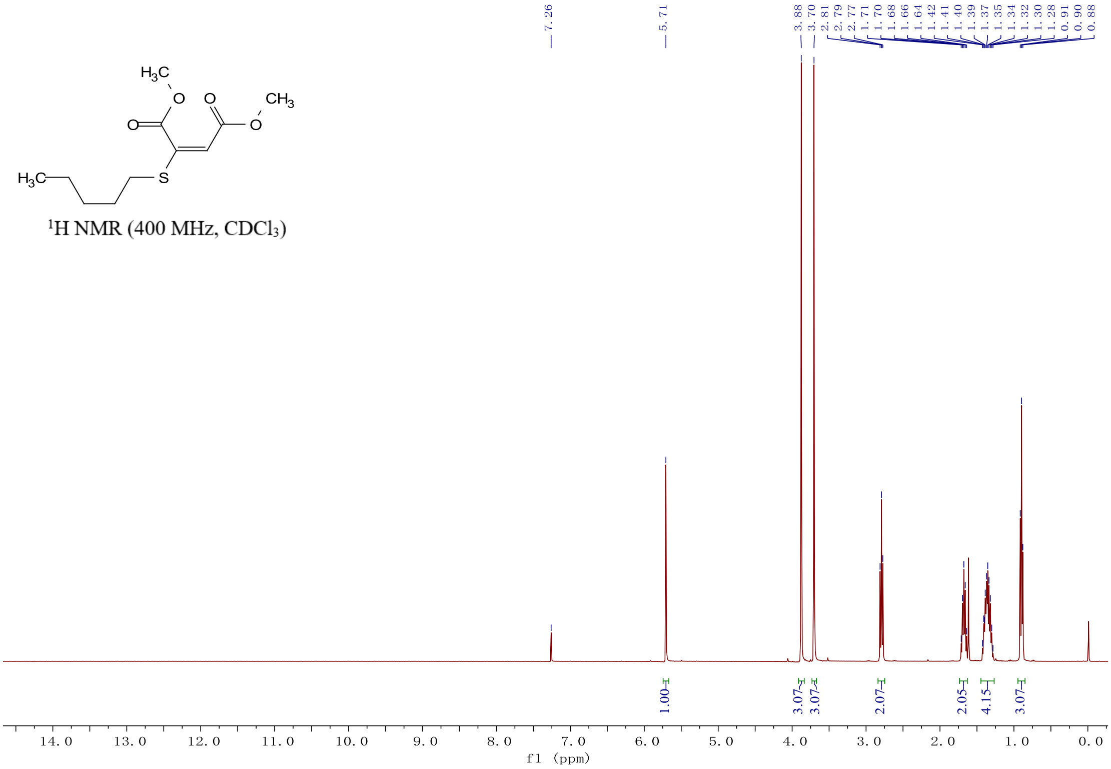

^{1}H NMR (400 MHz, $CDCl_3$)

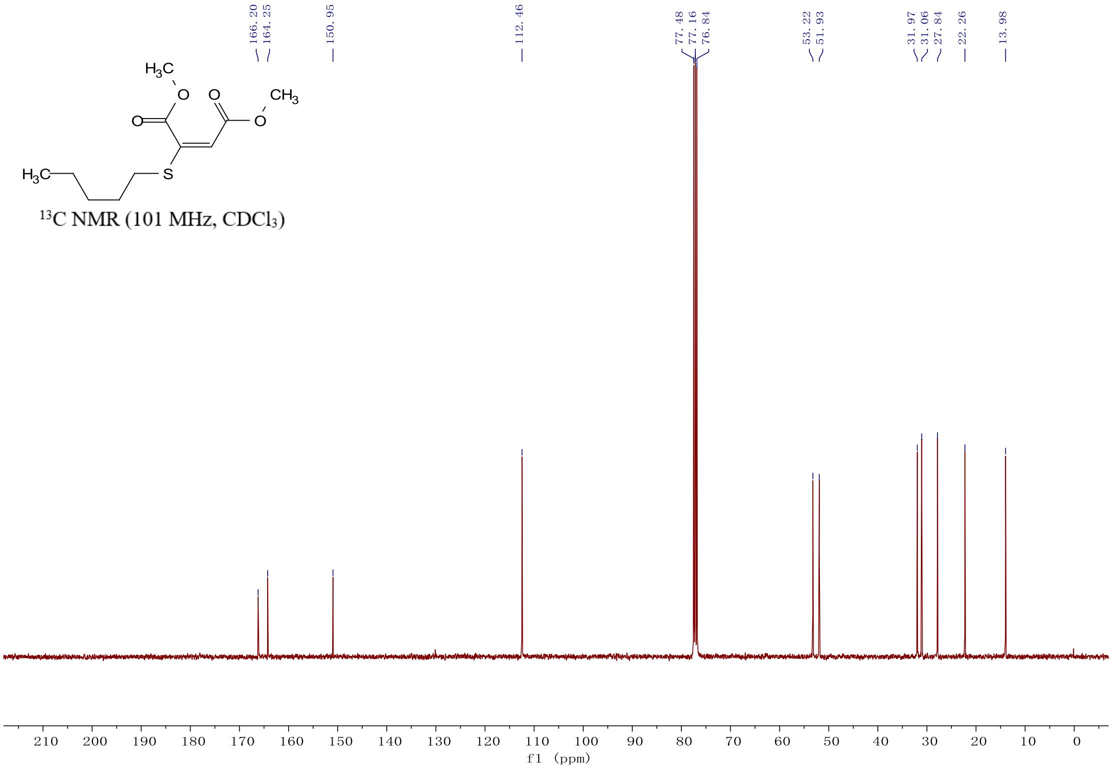

^{13}C NMR (101 MHz, $CDCl_3$)

dimethyl 2-(cyclohexylthio)fumarate (*Z*-1as)

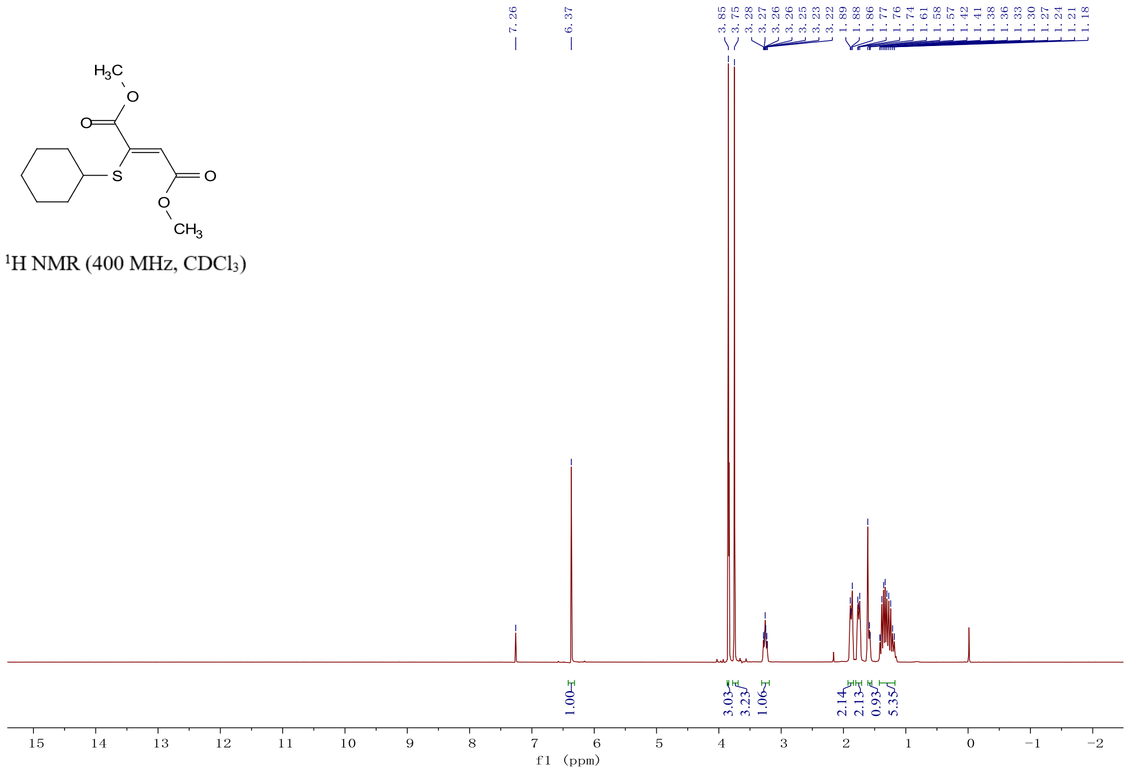

^{1}H NMR (400 MHz, $CDCl_3$)

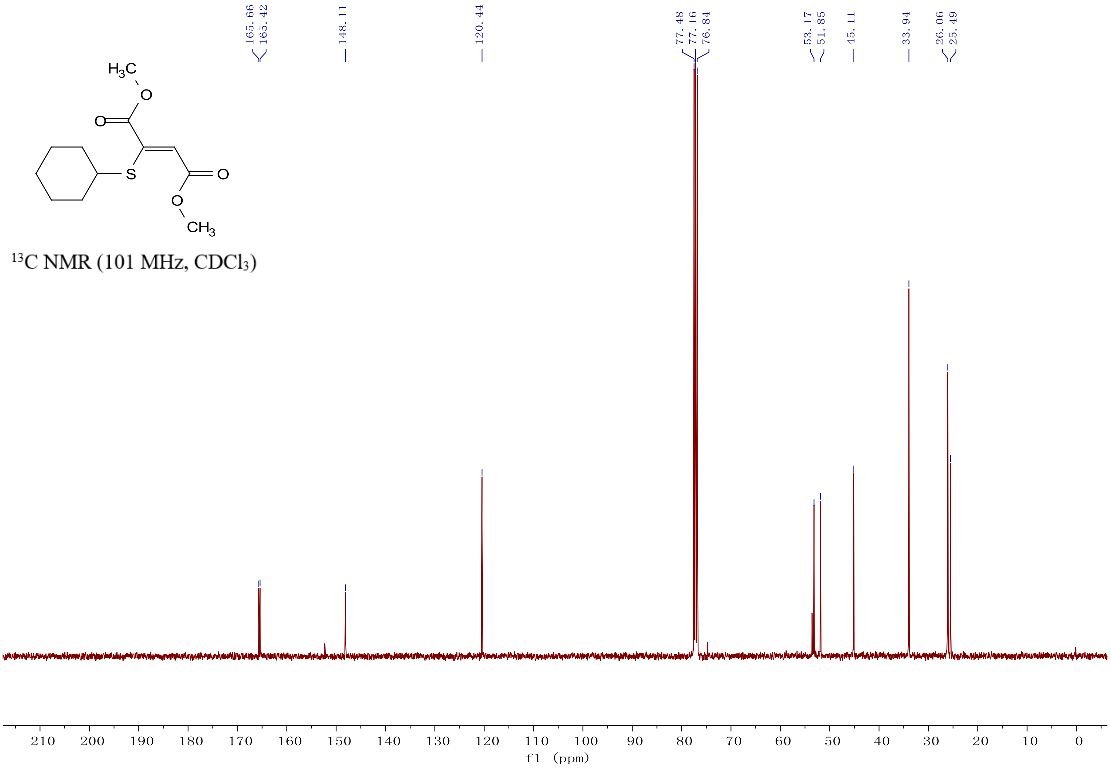

^{13}C NMR (101 MHz, $CDCl_3$)

dimethyl 2-(cyclohexylthio)maleate (*E*-1as)

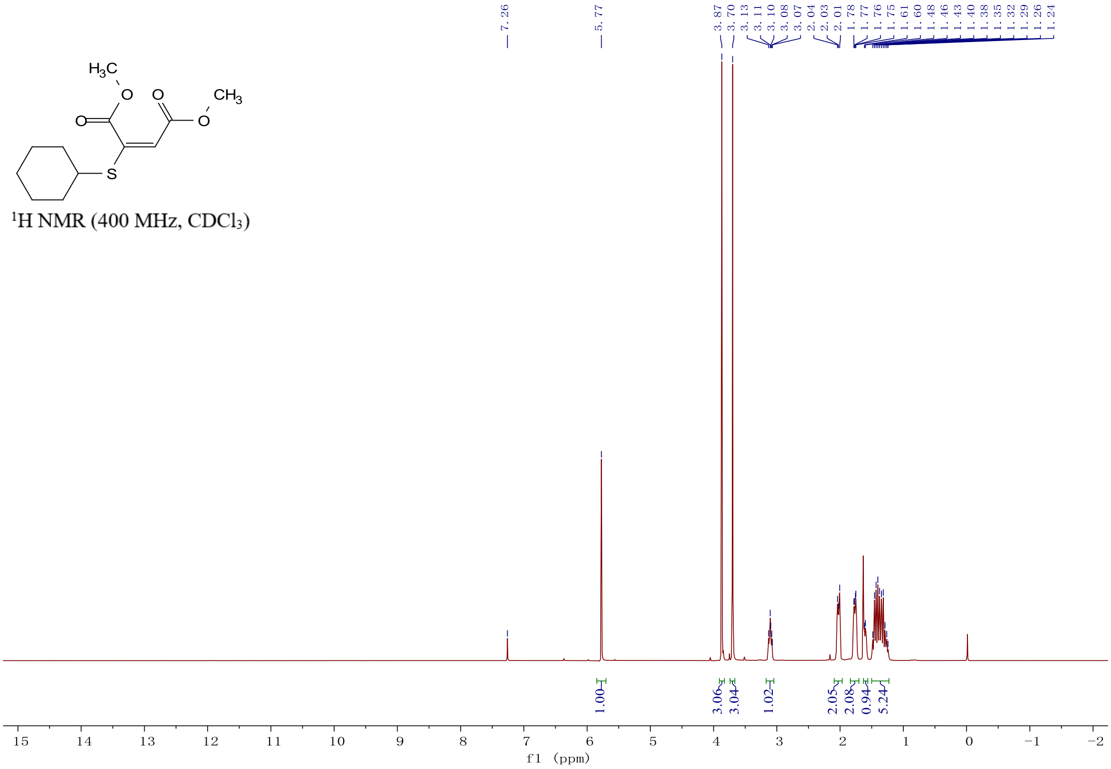

^{1}H NMR (400 MHz, $CDCl_3$)

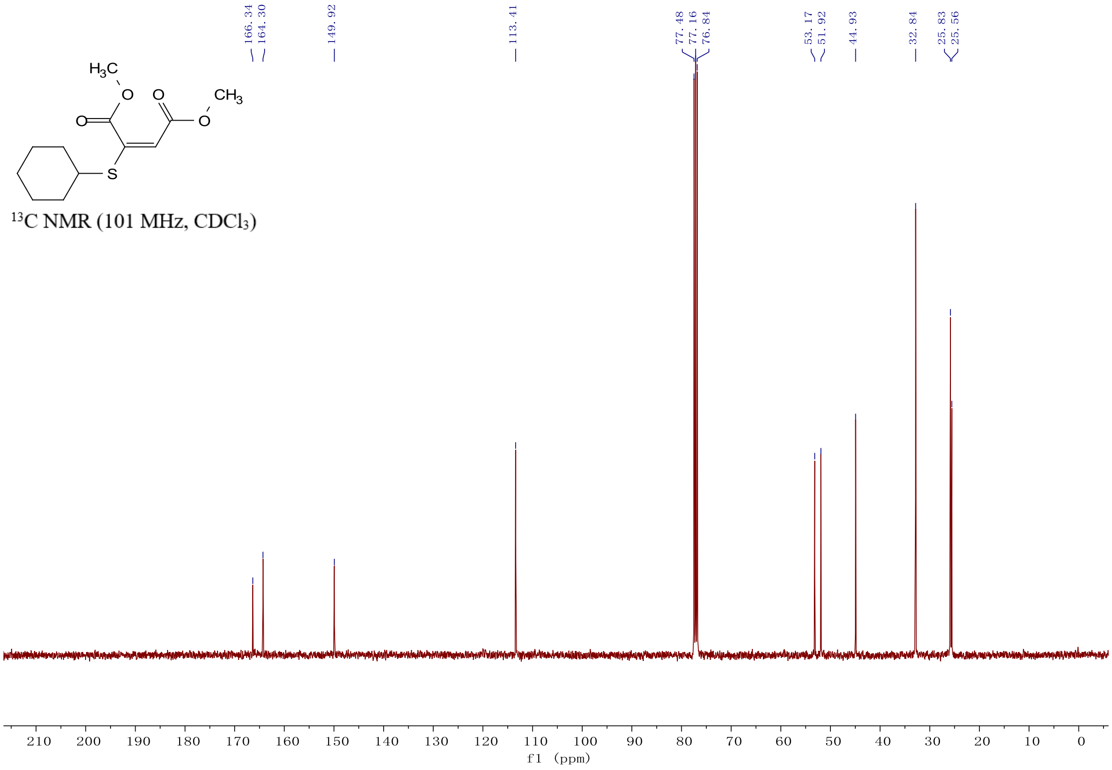

^{13}C NMR (101 MHz, $CDCl_3$)

dimethyl 2-((2-ethoxy-2-oxoethyl)thio)maleate (*E*-1at)

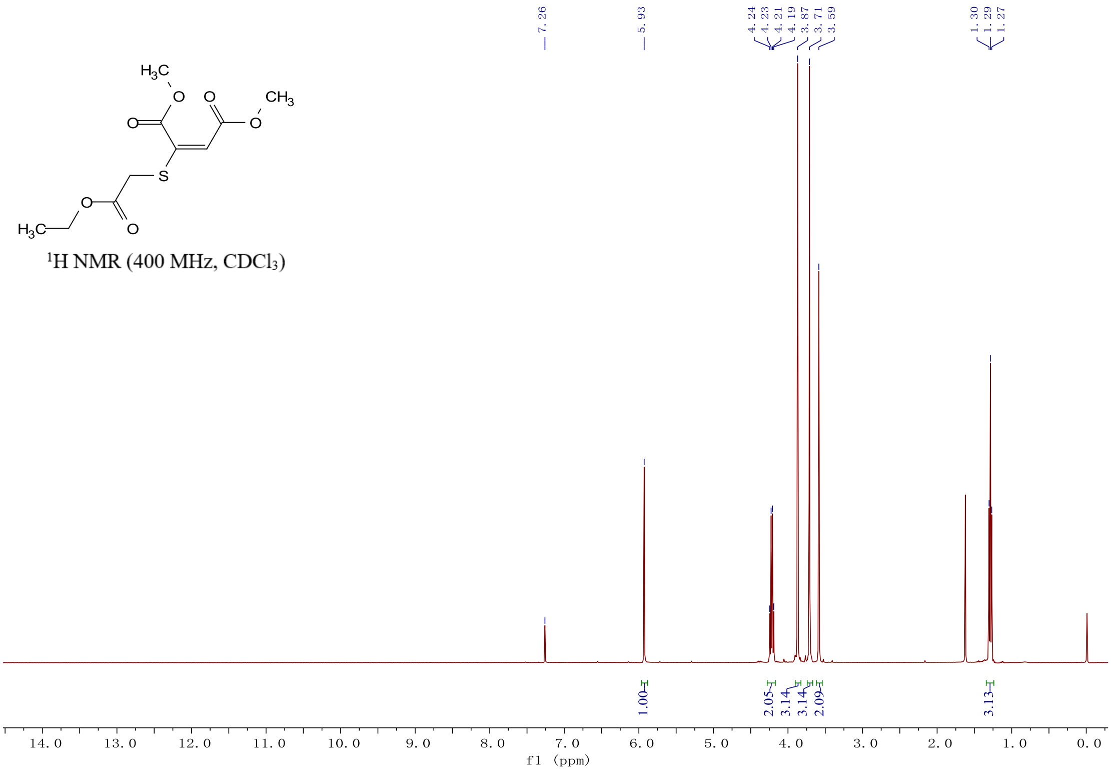

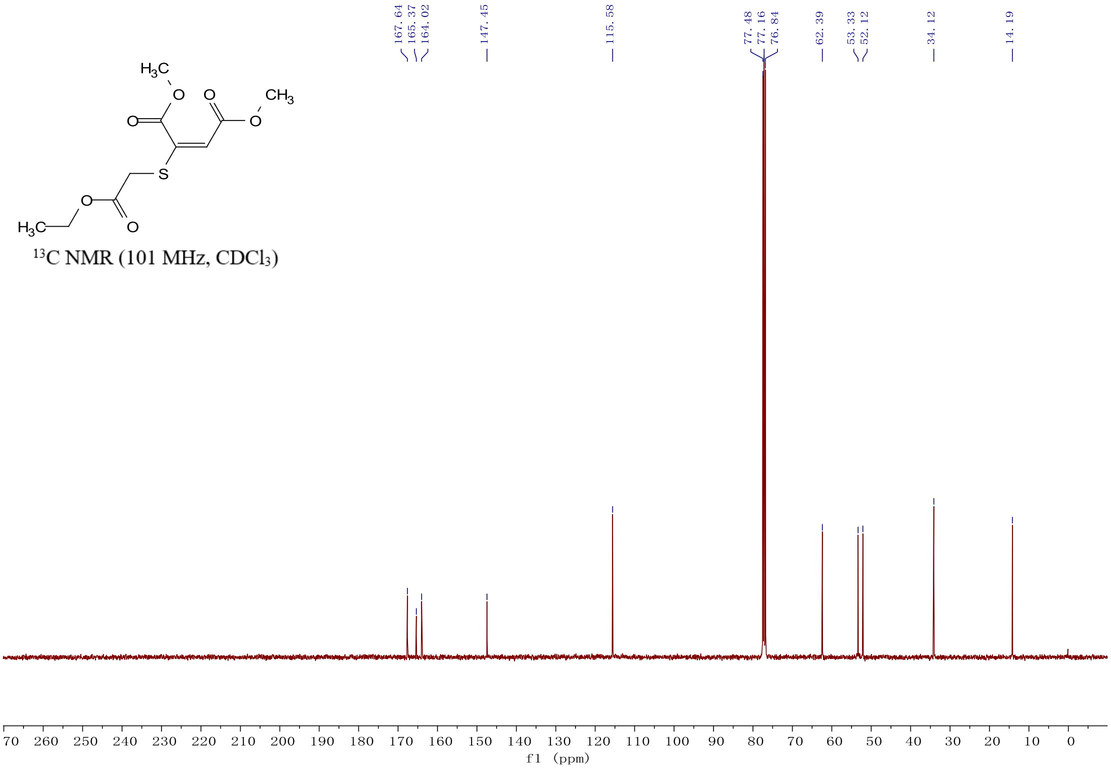

diethyl 2-(phenylthio)fumarate (*Z*-1au)

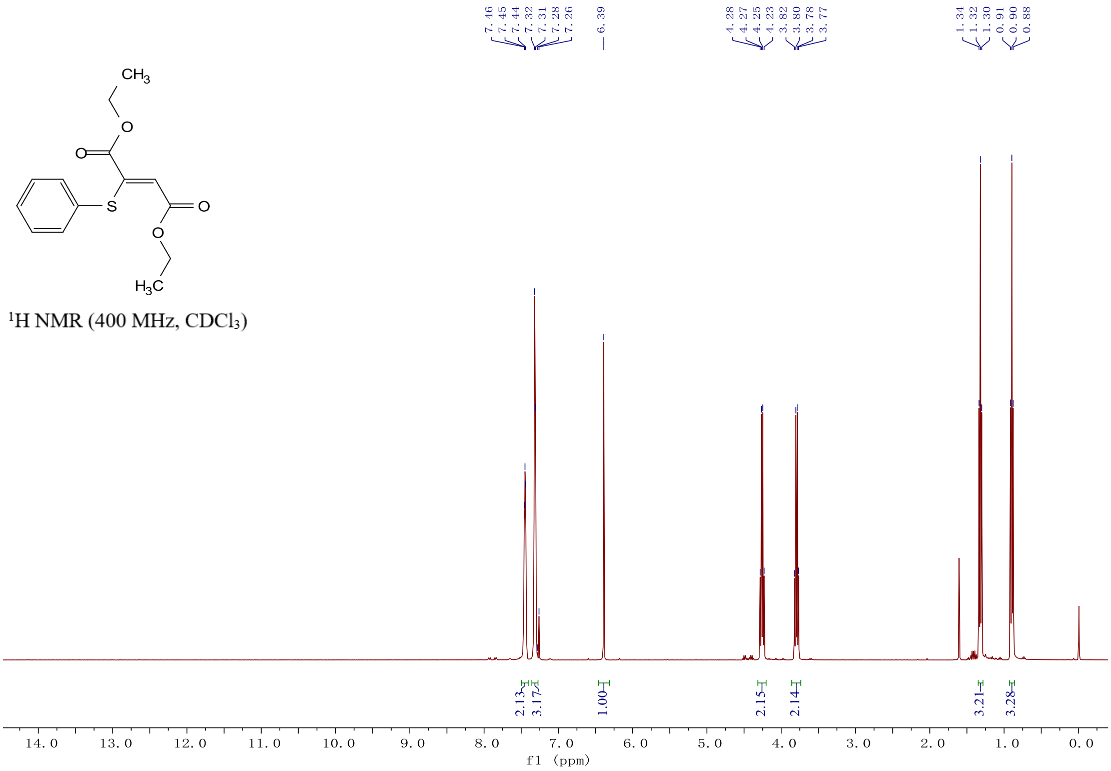

^{1}H NMR (400 MHz, $CDCl_3$)

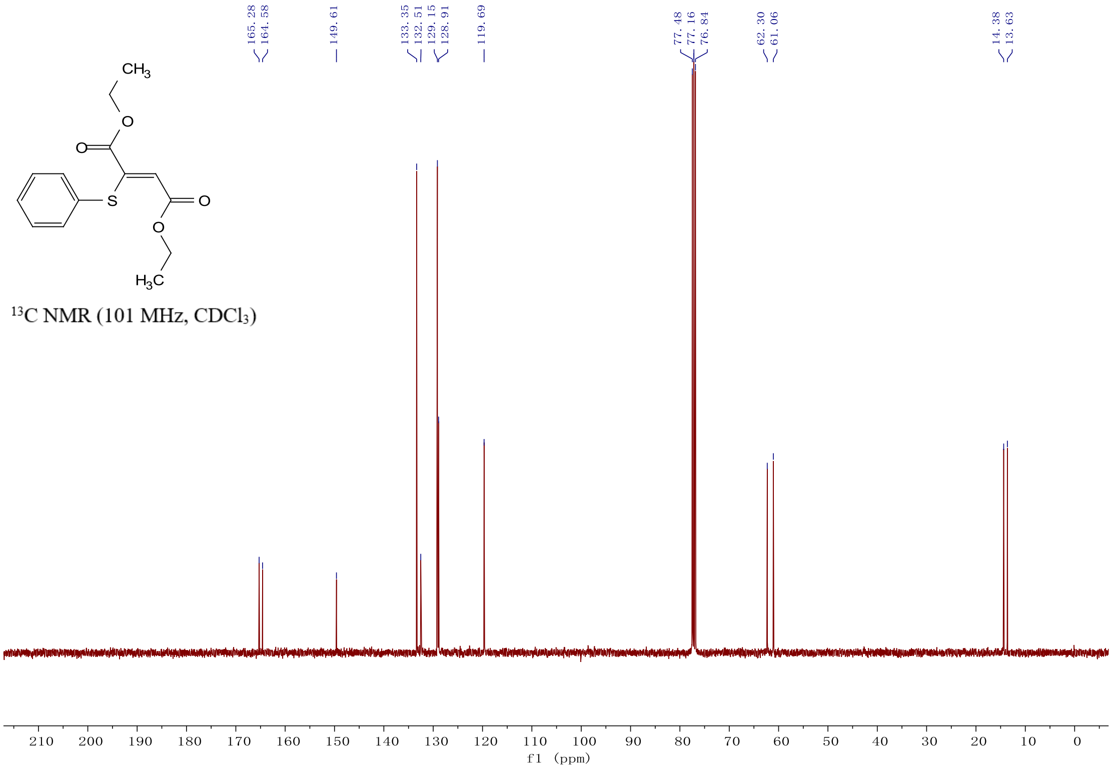

^{13}C NMR (101 MHz, $CDCl_3$)

diethyl 2-(phenylthio)maleate (*E*-1au)

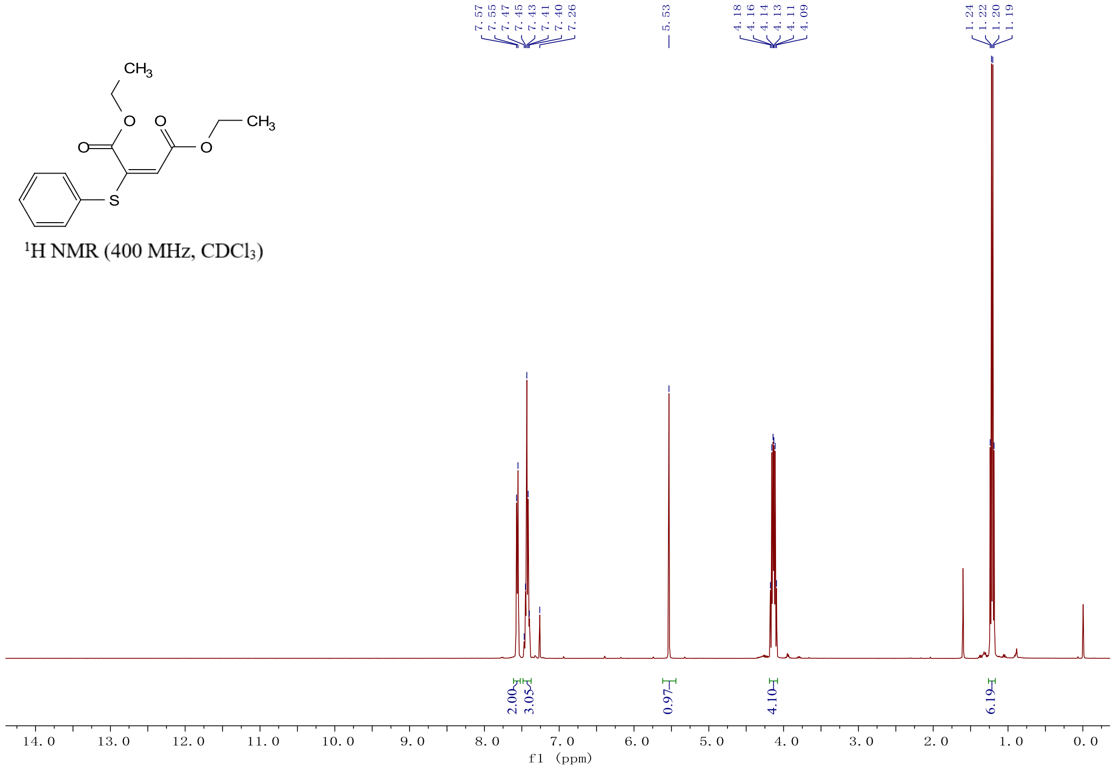

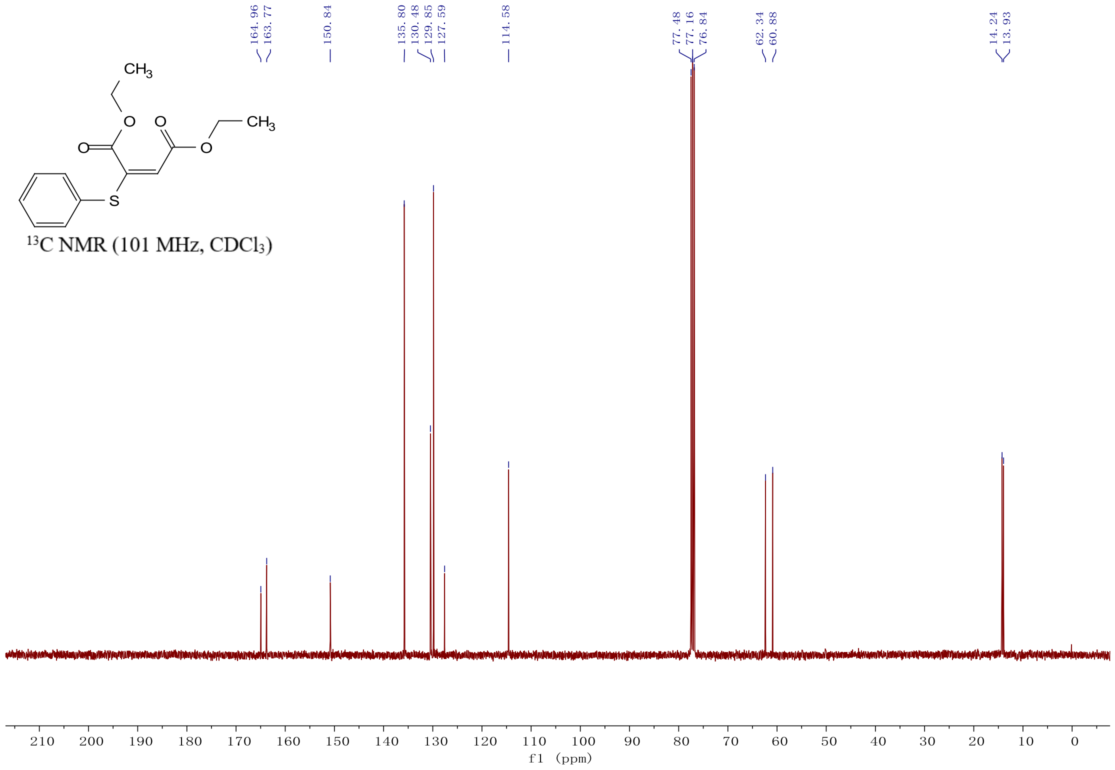

diisopropyl 2-(phenylthio)fumarate (*Z*-1av)

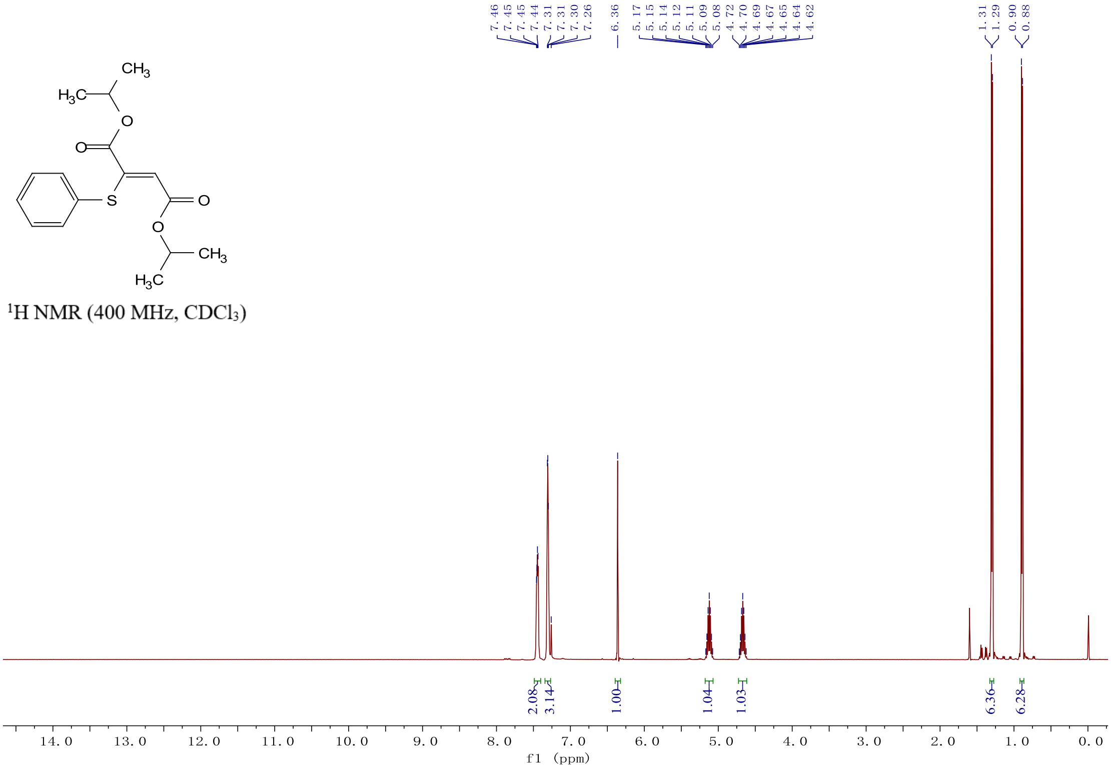

^{1}H NMR (400 MHz, $CDCl_3$)

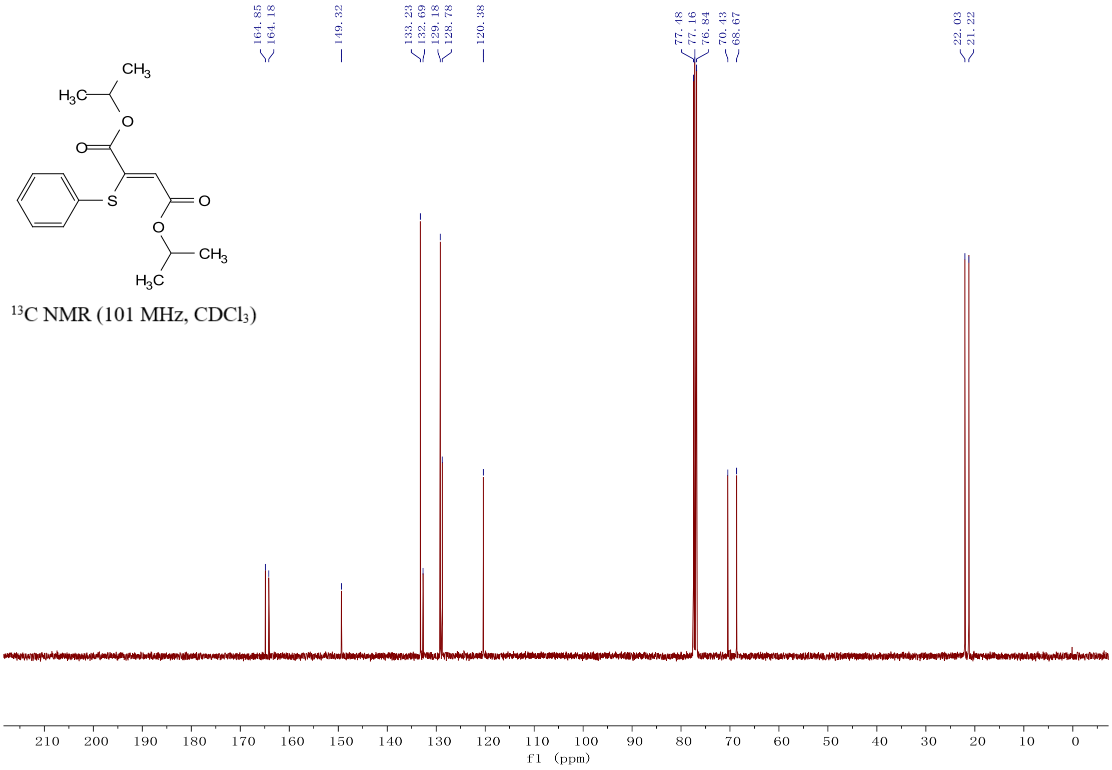

^{13}C NMR (101 MHz, $CDCl_3$)

diisopropyl 2-(phenylthio)maleate (*E*-1av)

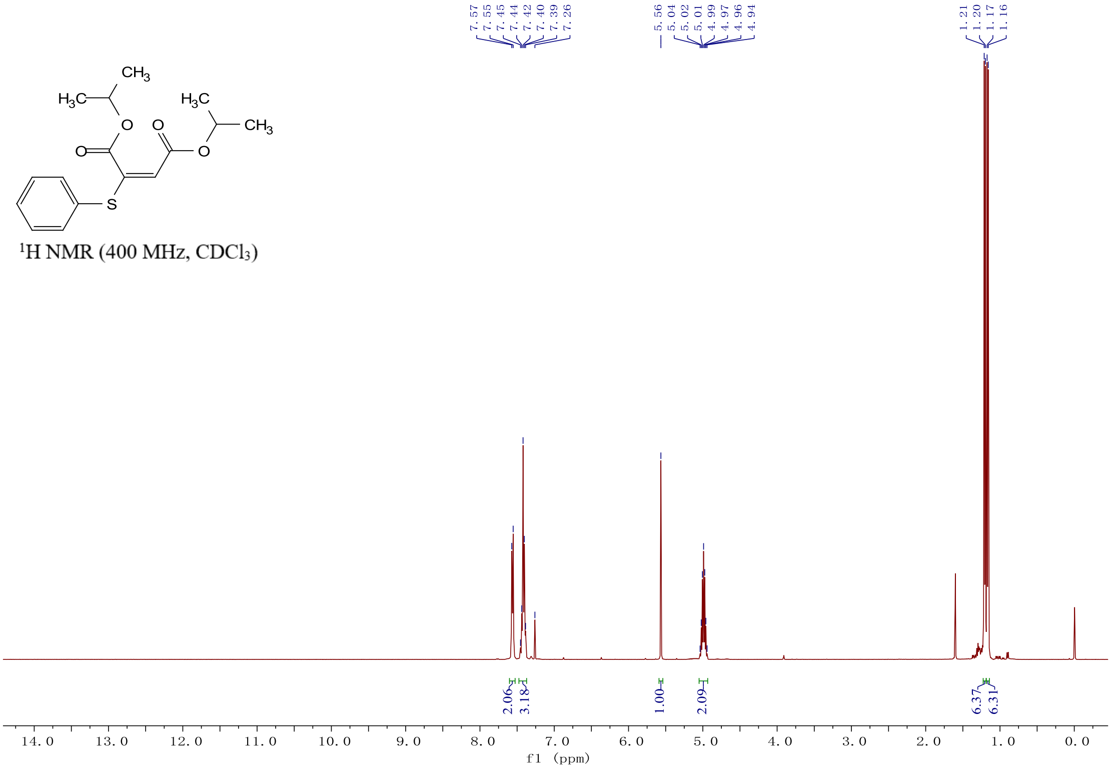

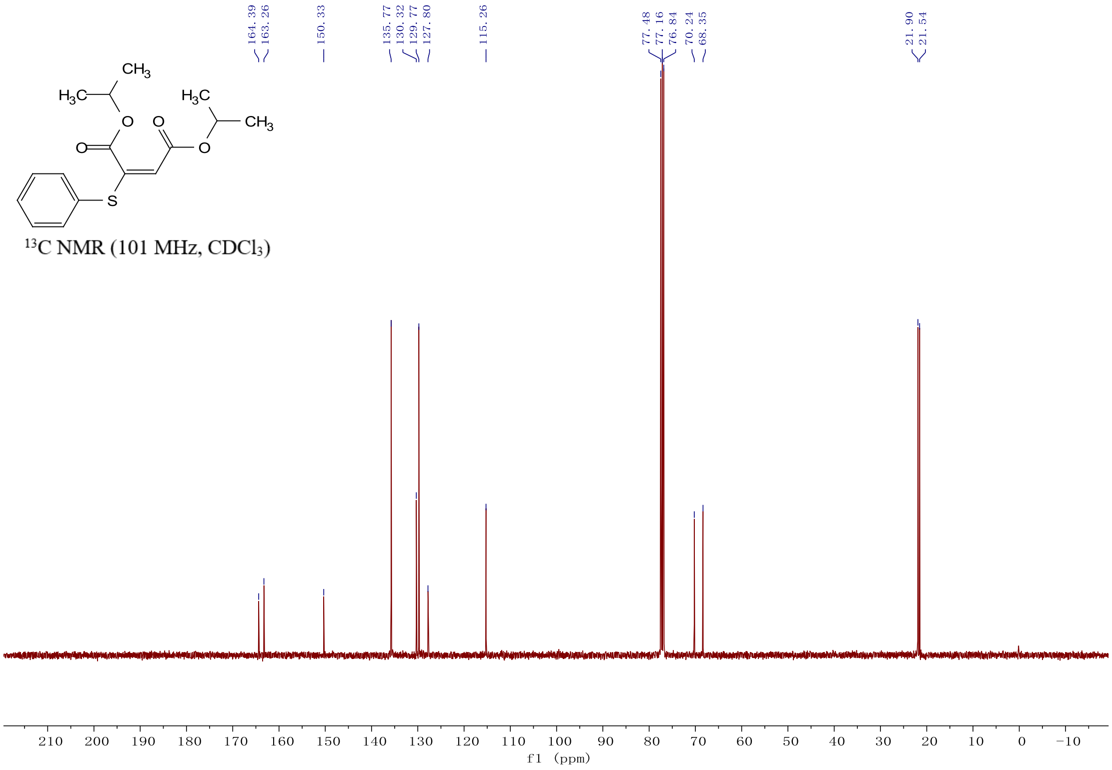

di-*tert*-butyl 2-(phenylthio)fumarate (*Z*-1aw)

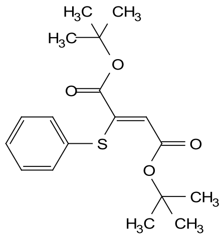

^{1}H NMR (400 MHz, $CDCl_3$)

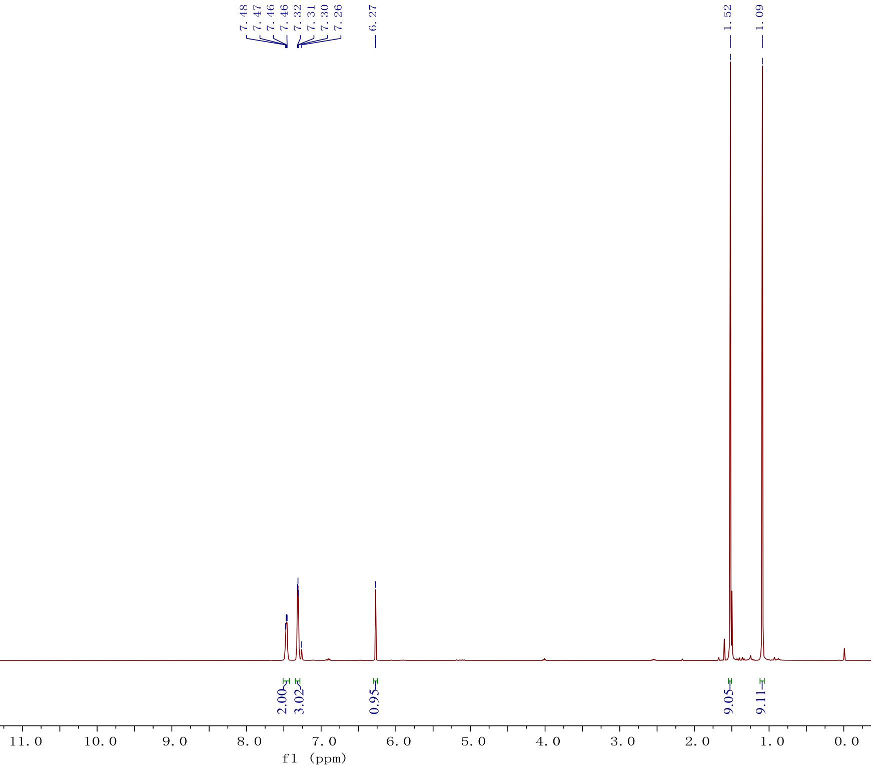

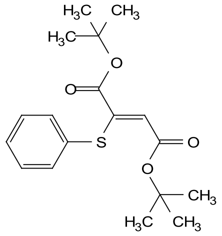

^{13}C NMR (101 MHz, $CDCl_3$)

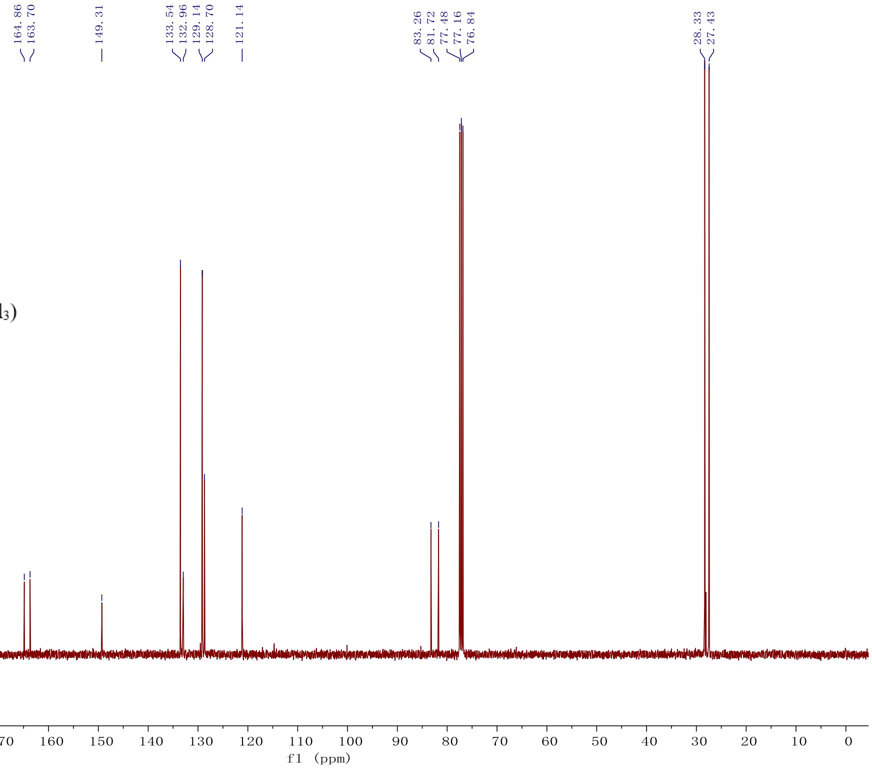

1-methyl-3-(phenylthio)-1*H*-pyrrole-2,5-dione (*E*-1ax)

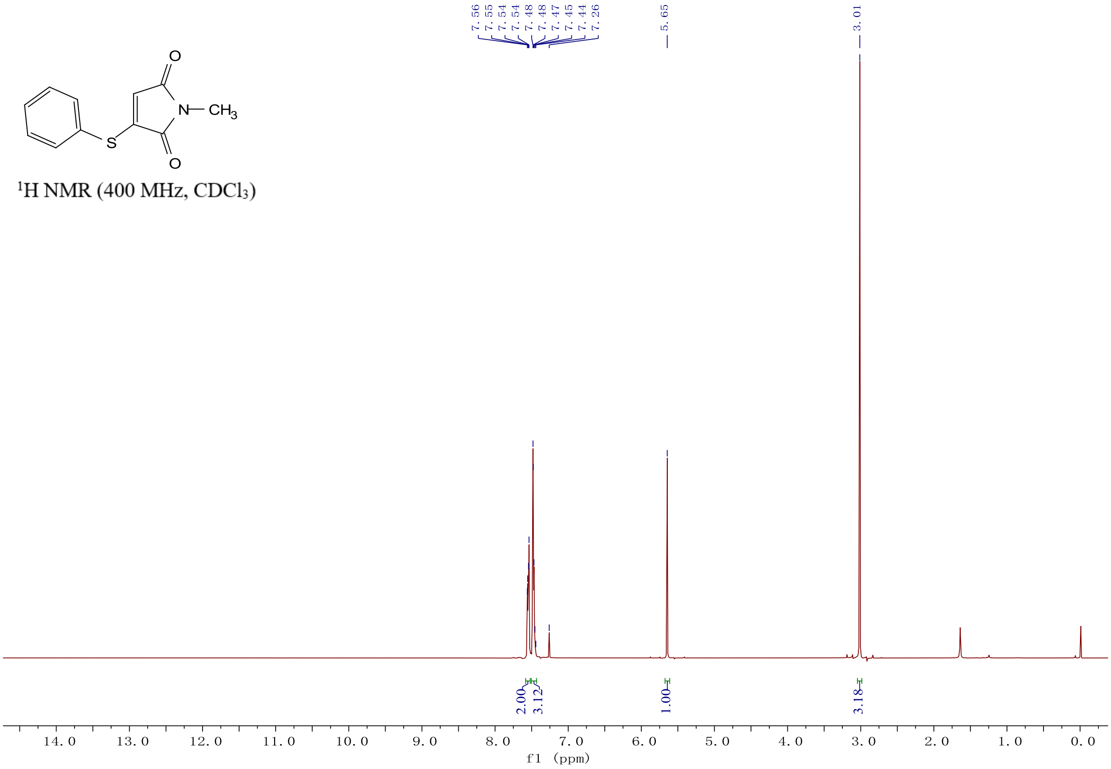

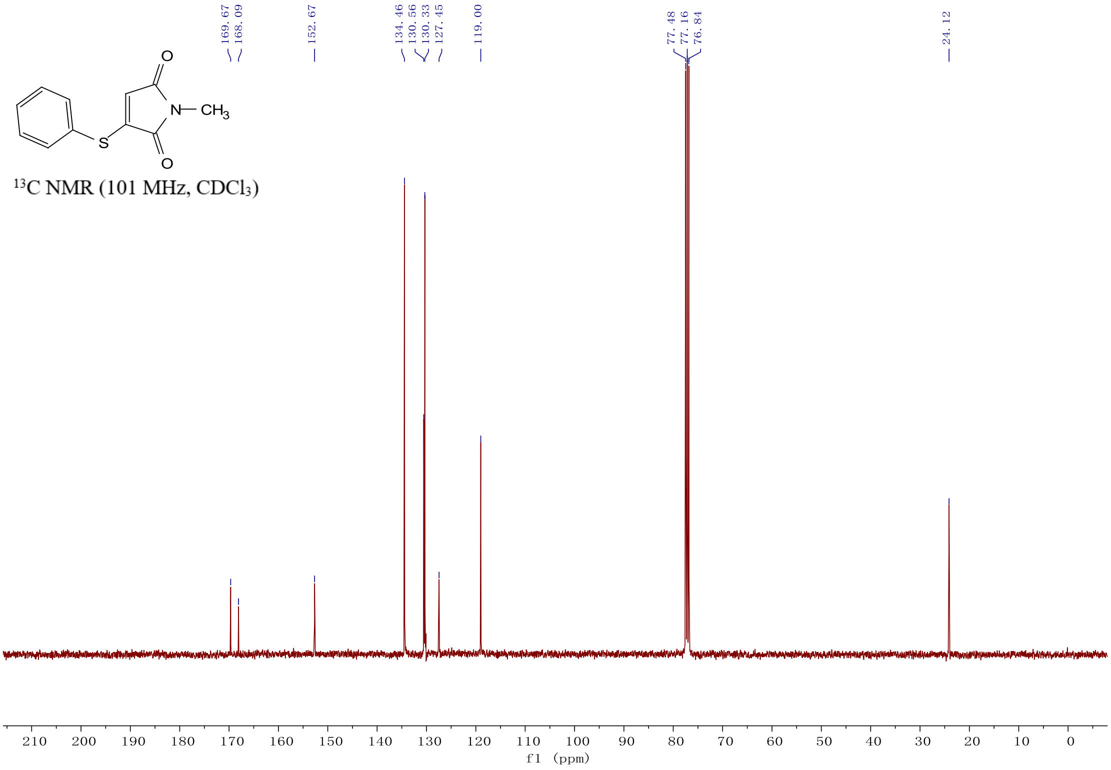

dimethyl 2-(phenylsulfonyl)fumarate (*Z*-1ay)

^{1}H NMR (400 MHz, $CDCl_3$)

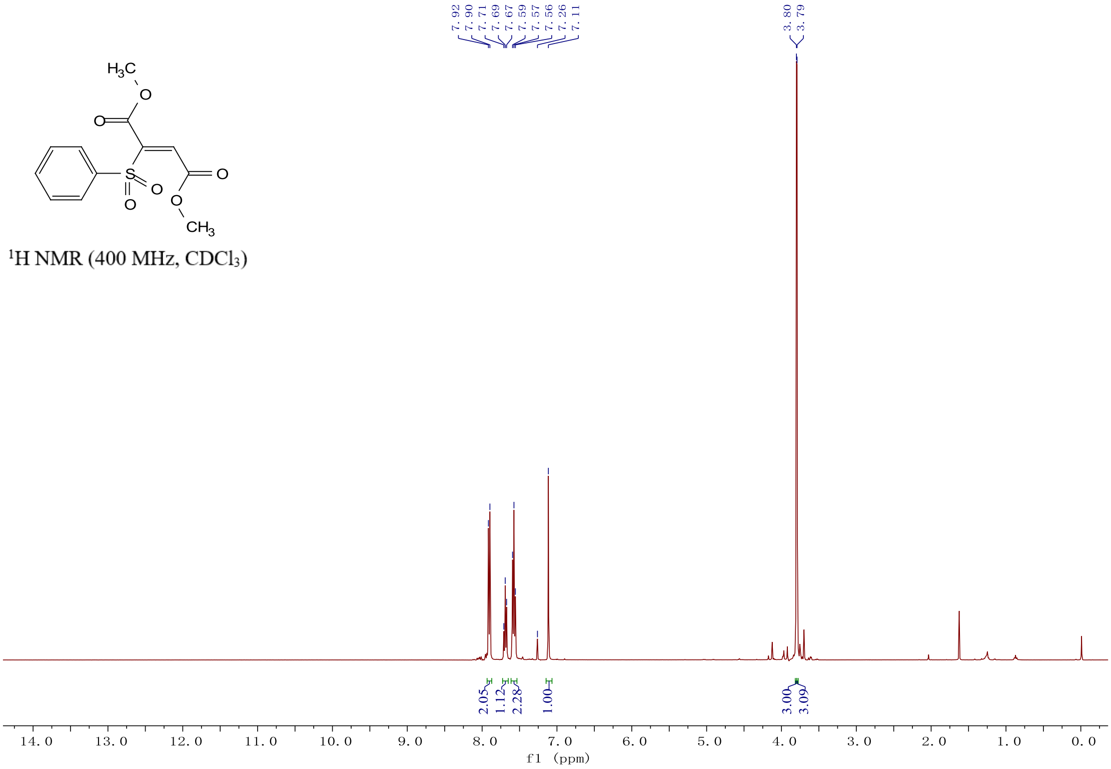

^{13}C NMR (101 MHz, $CDCl_3$)

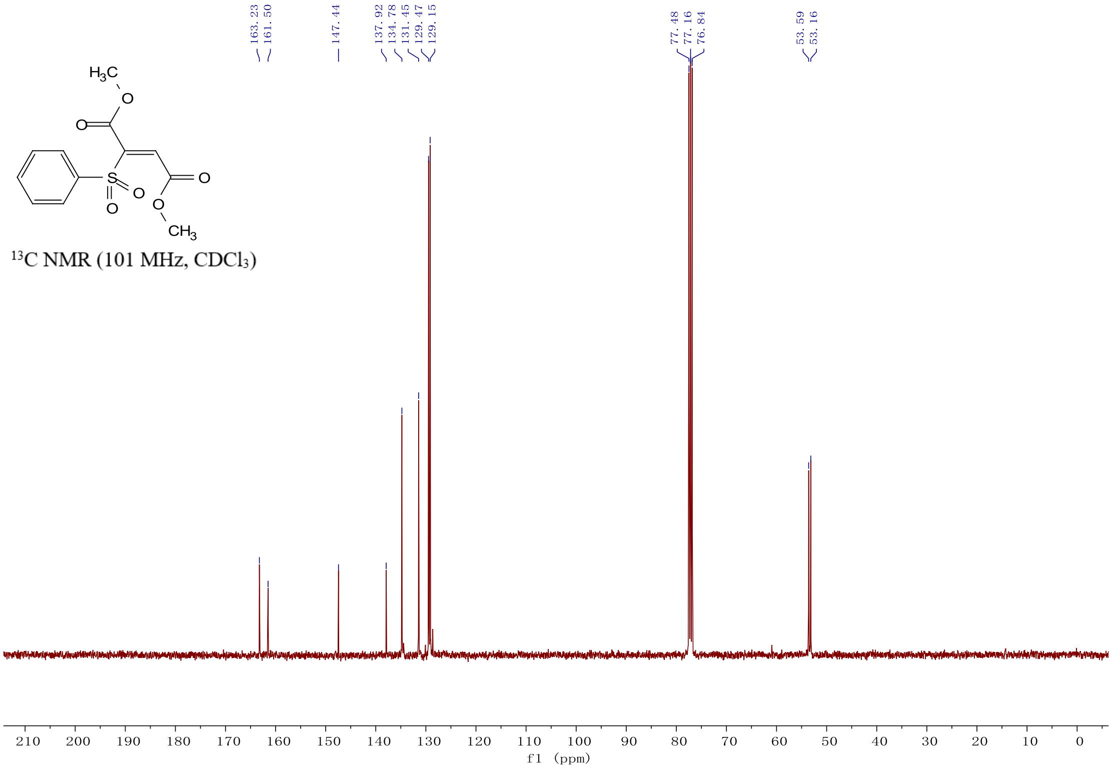

dimethyl 2-tosylfumarate (*Z*-1az)

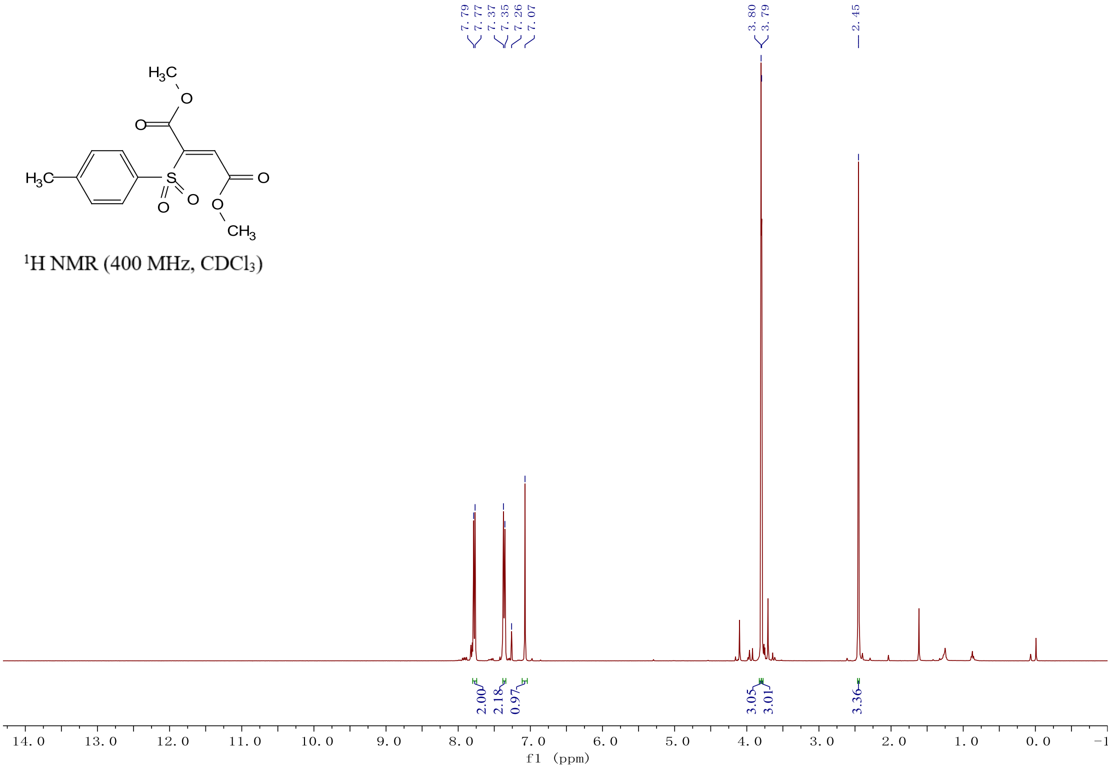

^{1}H NMR (400 MHz, $CDCl_3$)

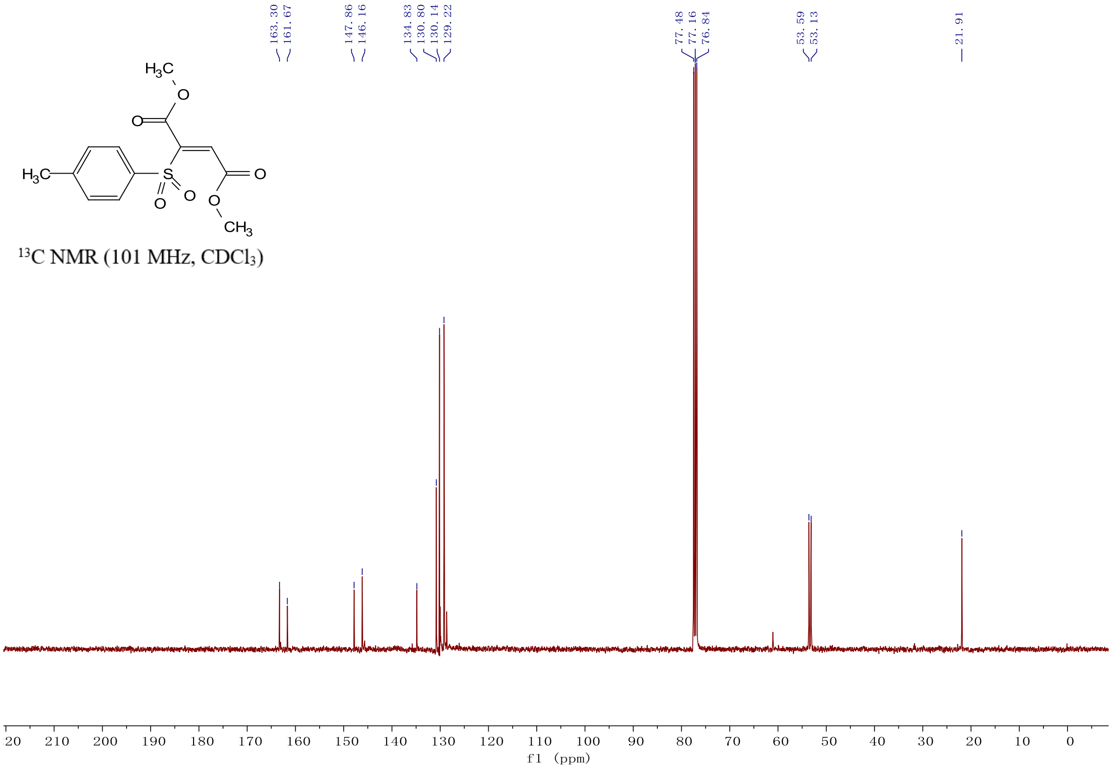

^{13}C NMR (101 MHz, $CDCl_3$)

dimethyl 2-(phenylamino)maleate (*E*-1ba)

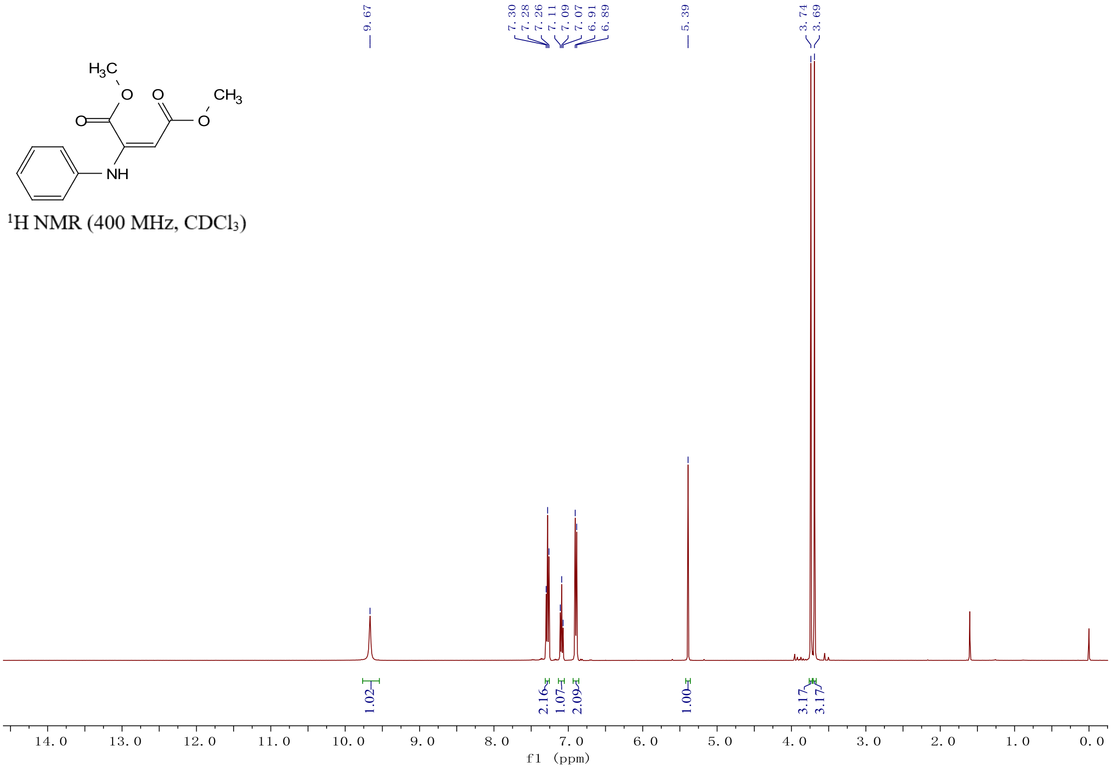

^{1}H NMR (400 MHz, $CDCl_3$)

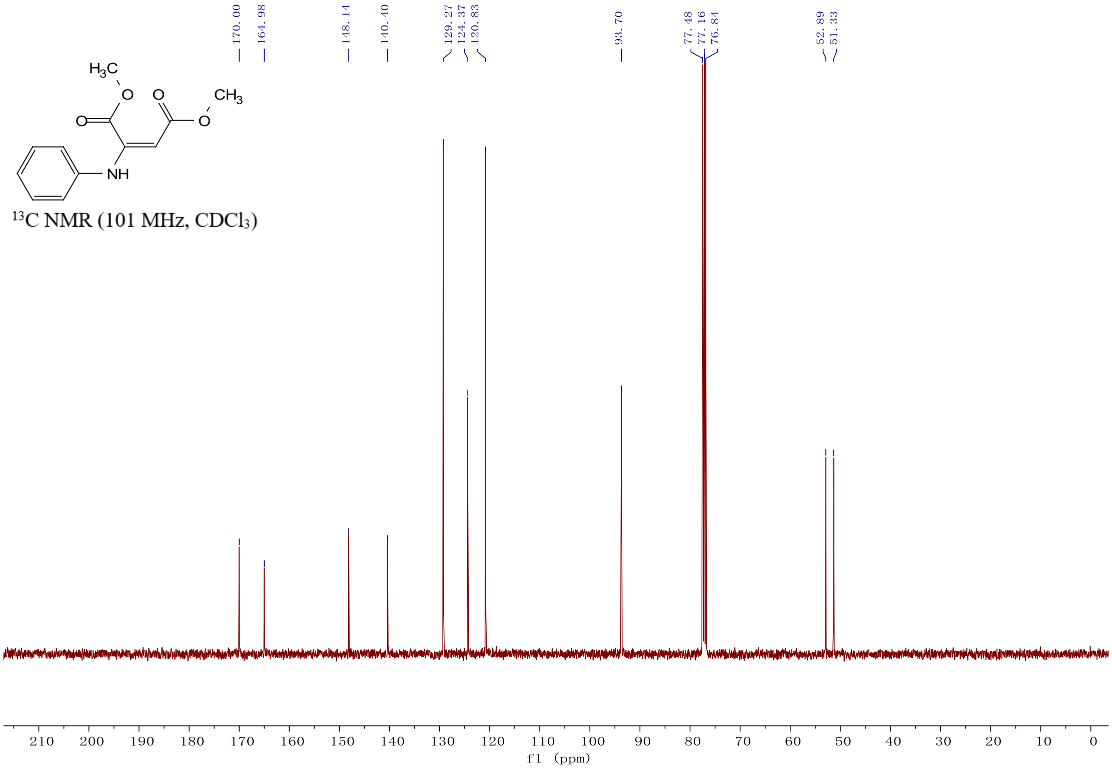

^{13}C NMR (101 MHz, $CDCl_3$)

dimethyl 2-phenoxyfumarate/dimethyl 2-phenoxymaleate (*Z*-**1bb**/*E*-**1bb** = 0.8/1)

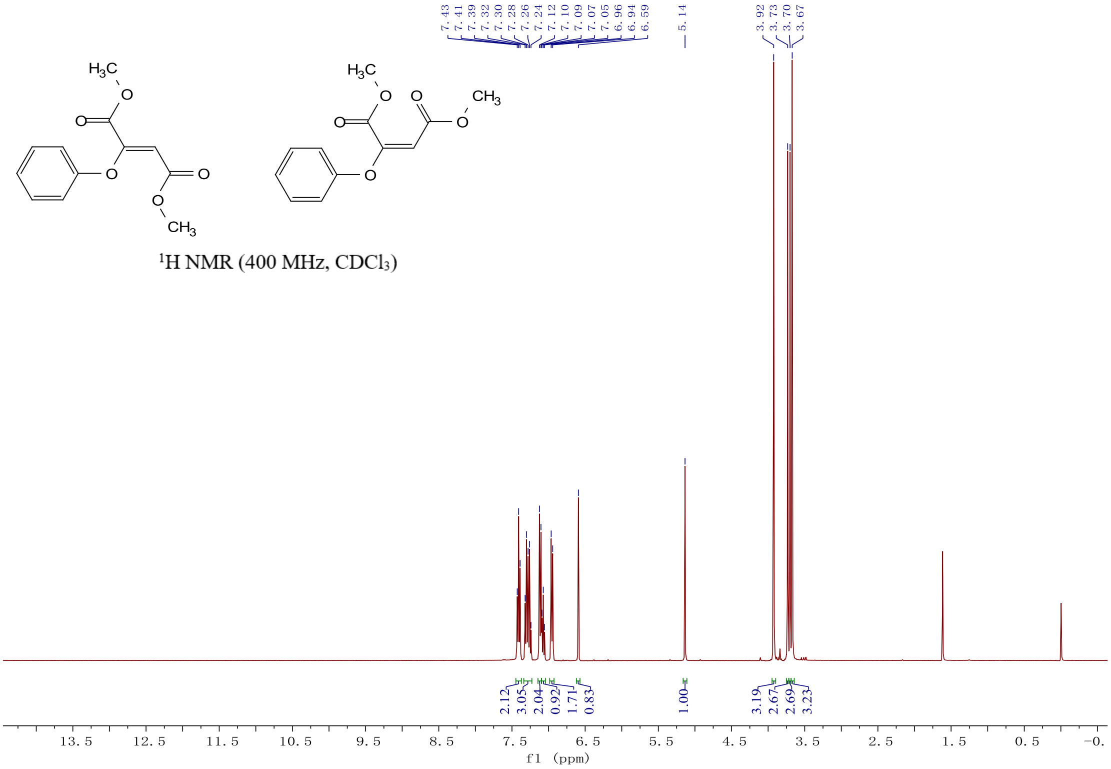

^{1}H NMR (400 MHz, $CDCl_3$)

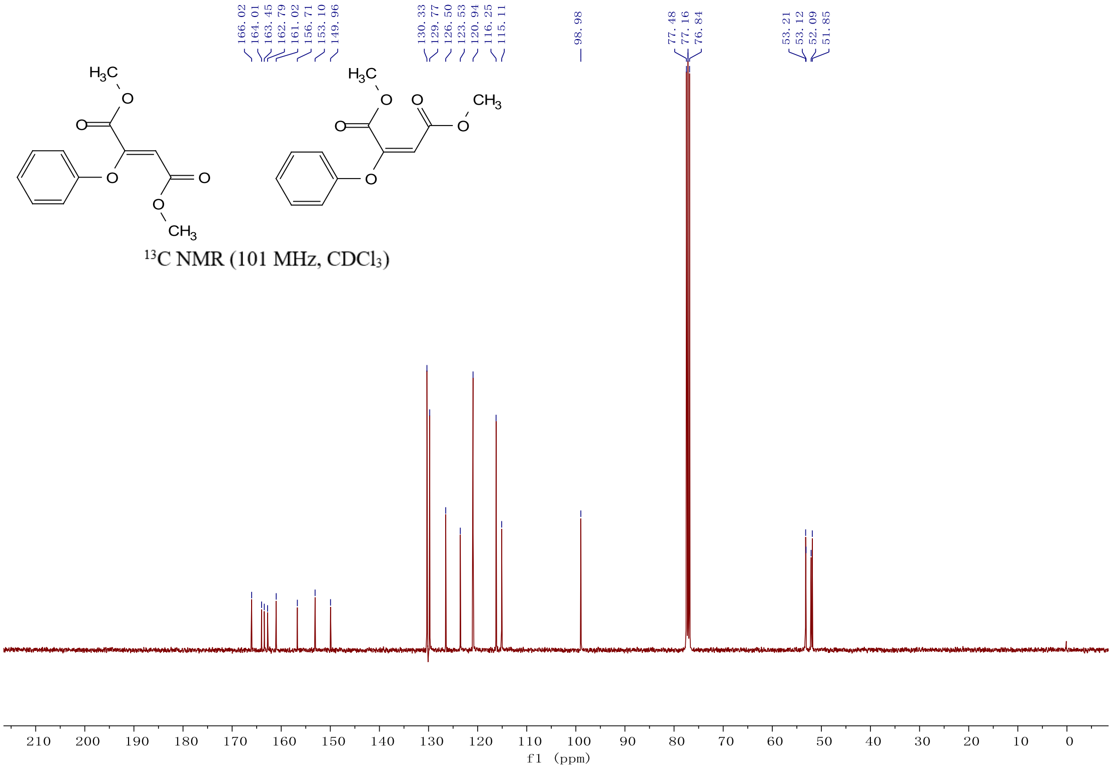

^{13}C NMR (101 MHz, $CDCl_3$)

dimethyl 2-(4-methoxyphenoxy)fumarate/dimethyl 2-(4-methoxyphenoxy)maleate (*Z*-**1bc**/*E*-**1bc** = 1/1)

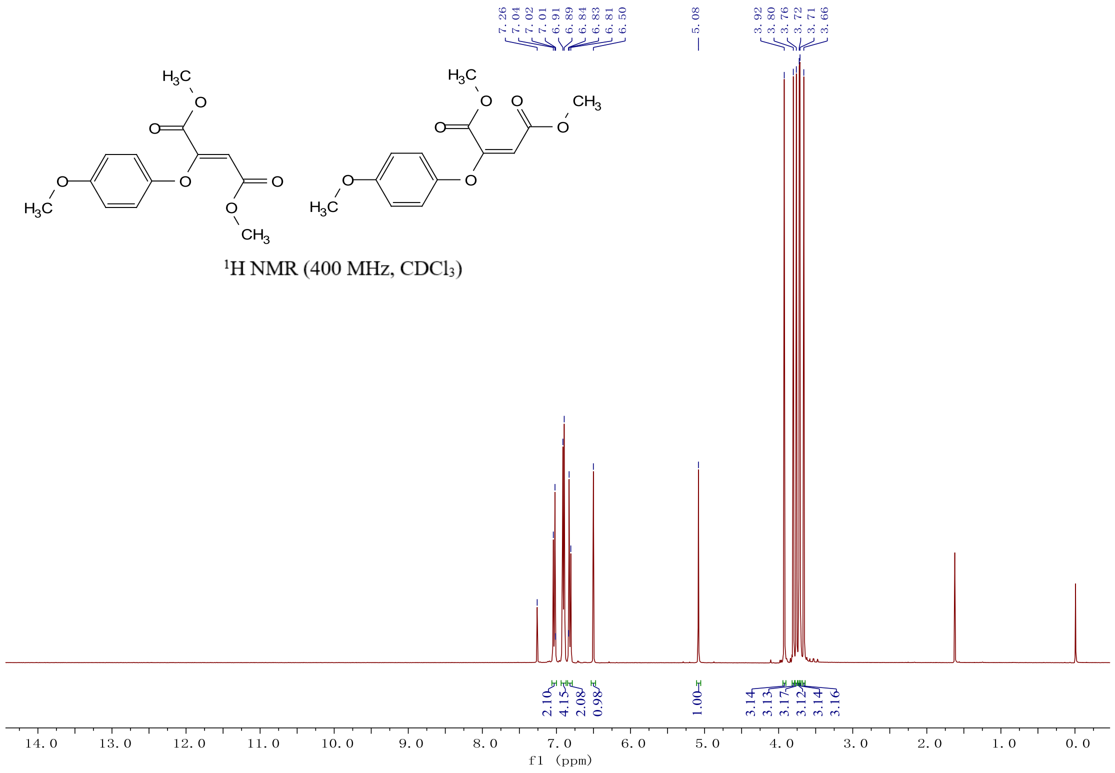

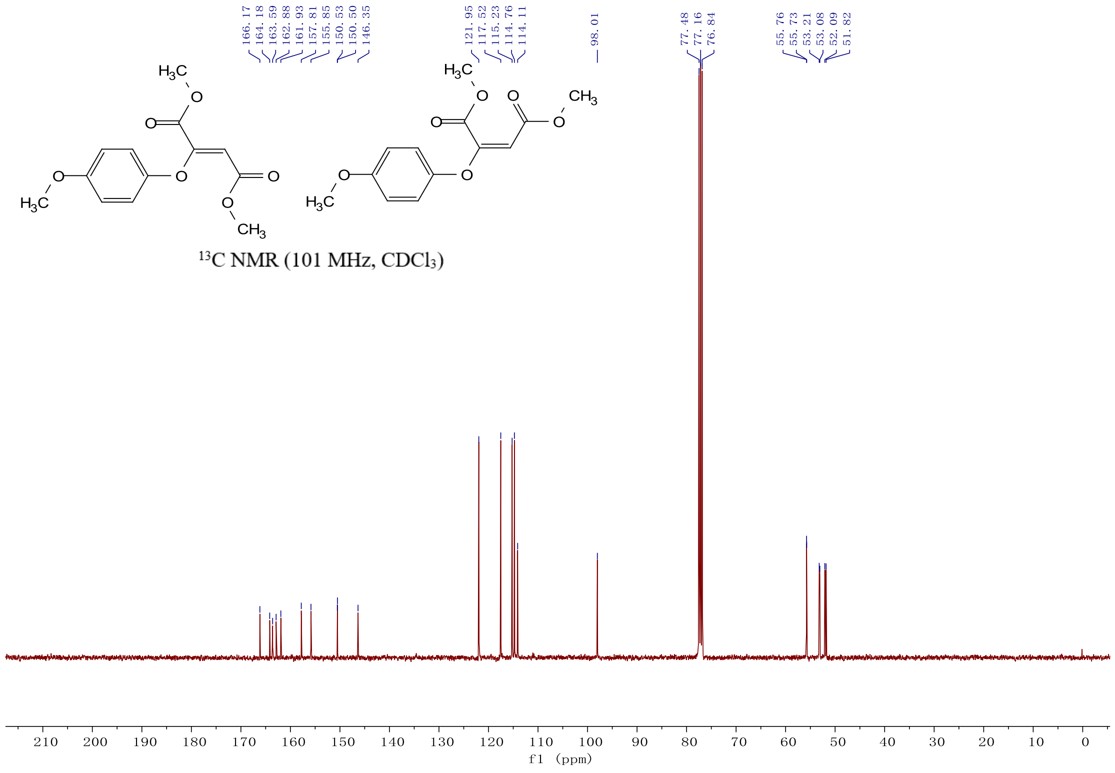

dimethyl 2-(phenylselanyl)fumarate (*Z*-1bd)

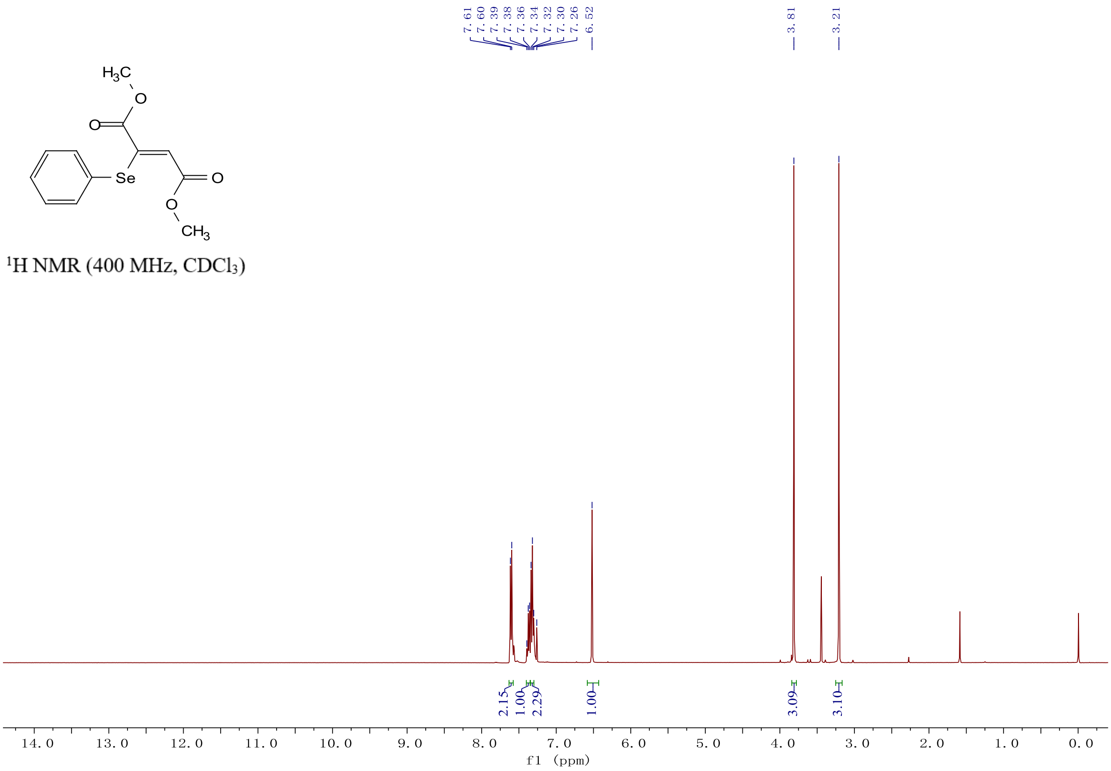

^{1}H NMR (400 MHz, $CDCl_3$)

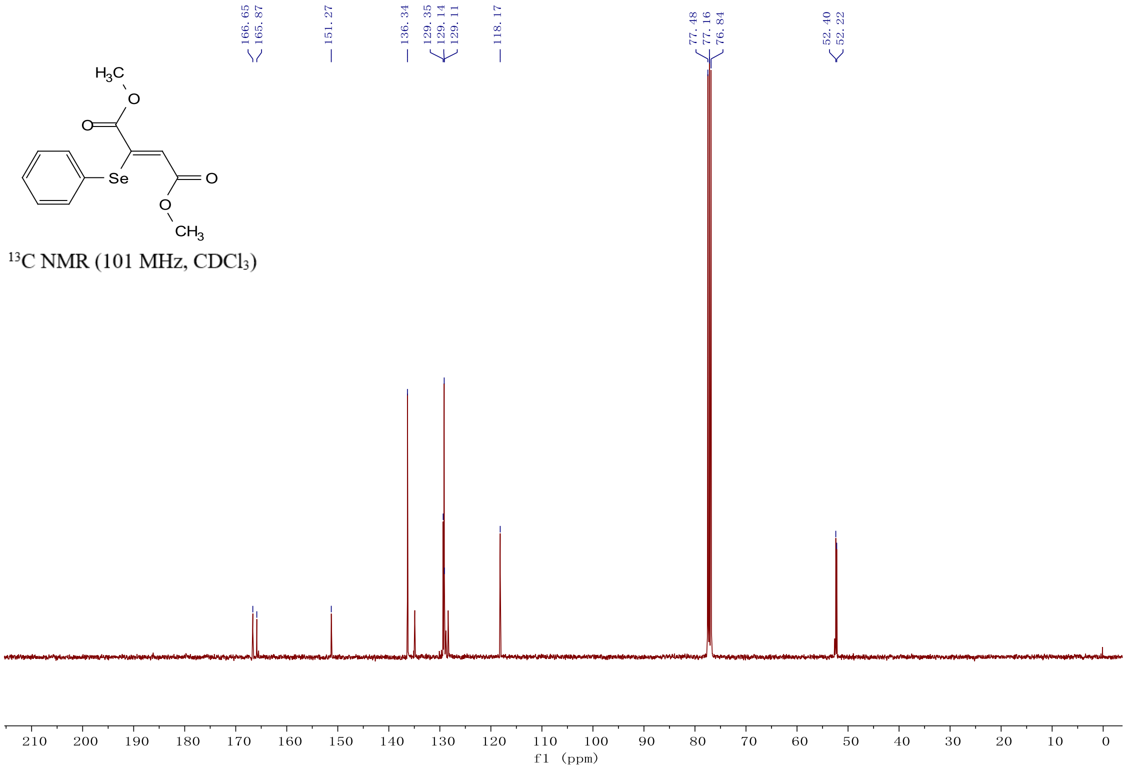

^{13}C NMR (101 MHz, $CDCl_3$)

dimethyl 2-(phenylselanyl)maleate (*E*-1bd)

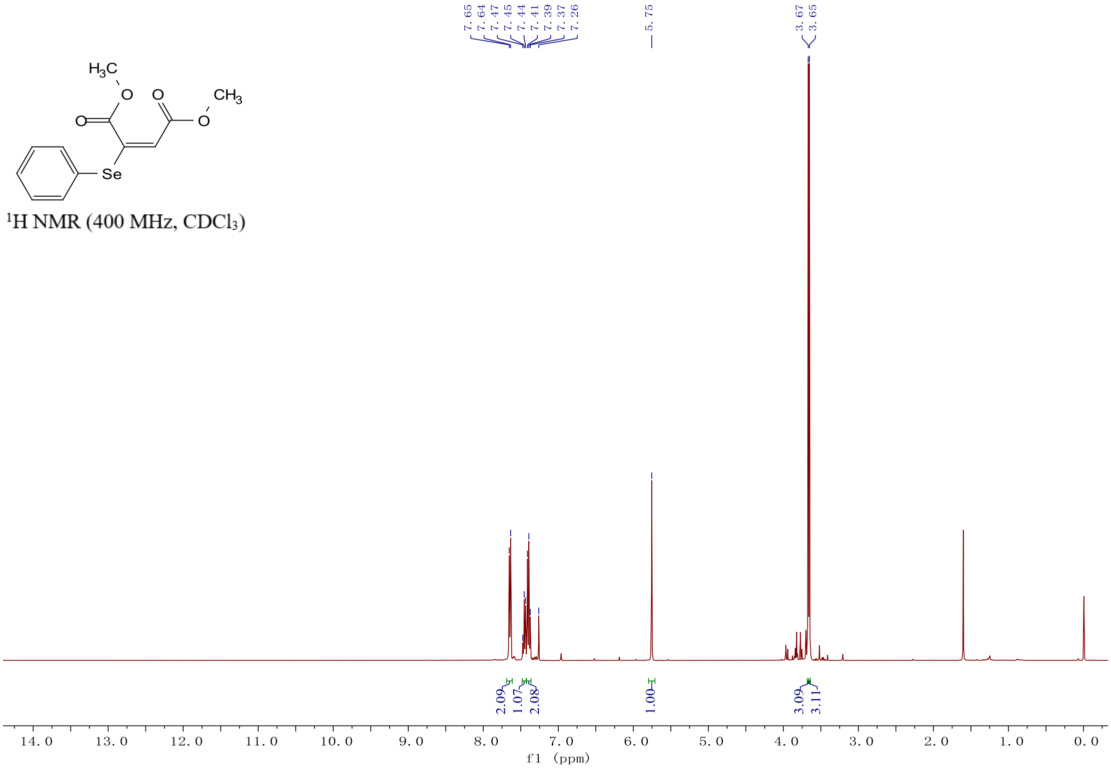

^{1}H NMR (400 MHz, $CDCl_3$)

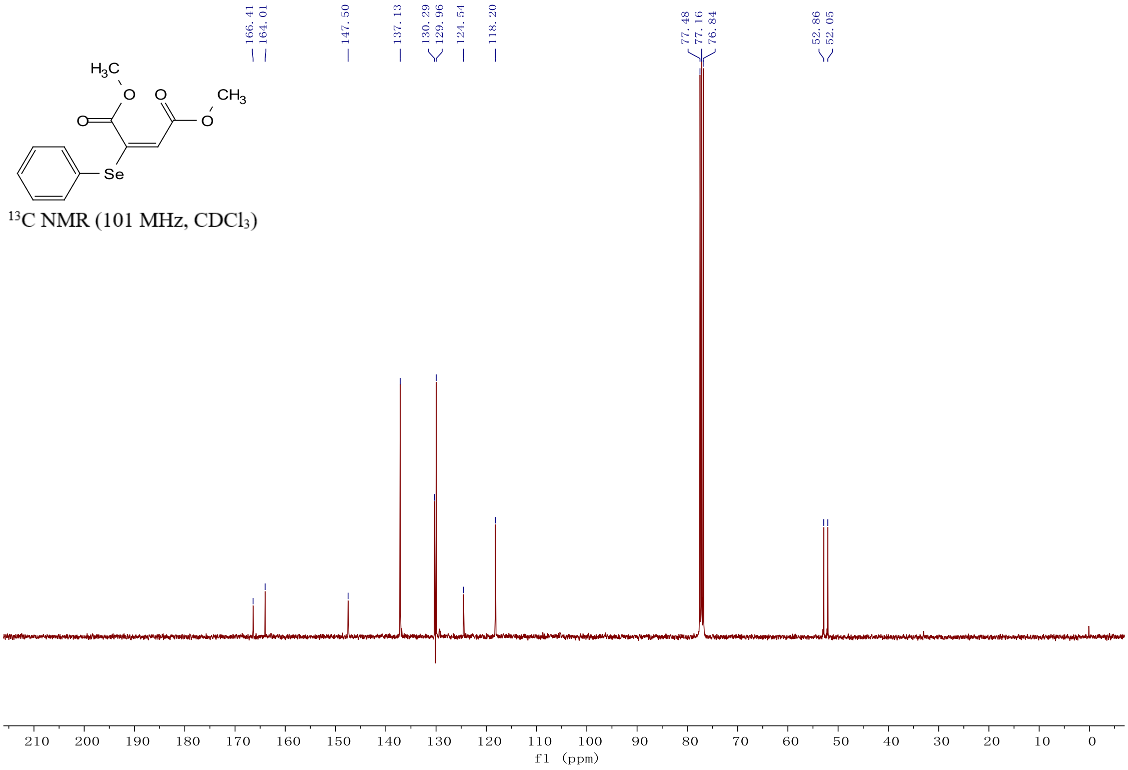

^{13}C NMR (101 MHz, $CDCl_3$)

dimethyl 2-(phenylthio)succinate (2aa)

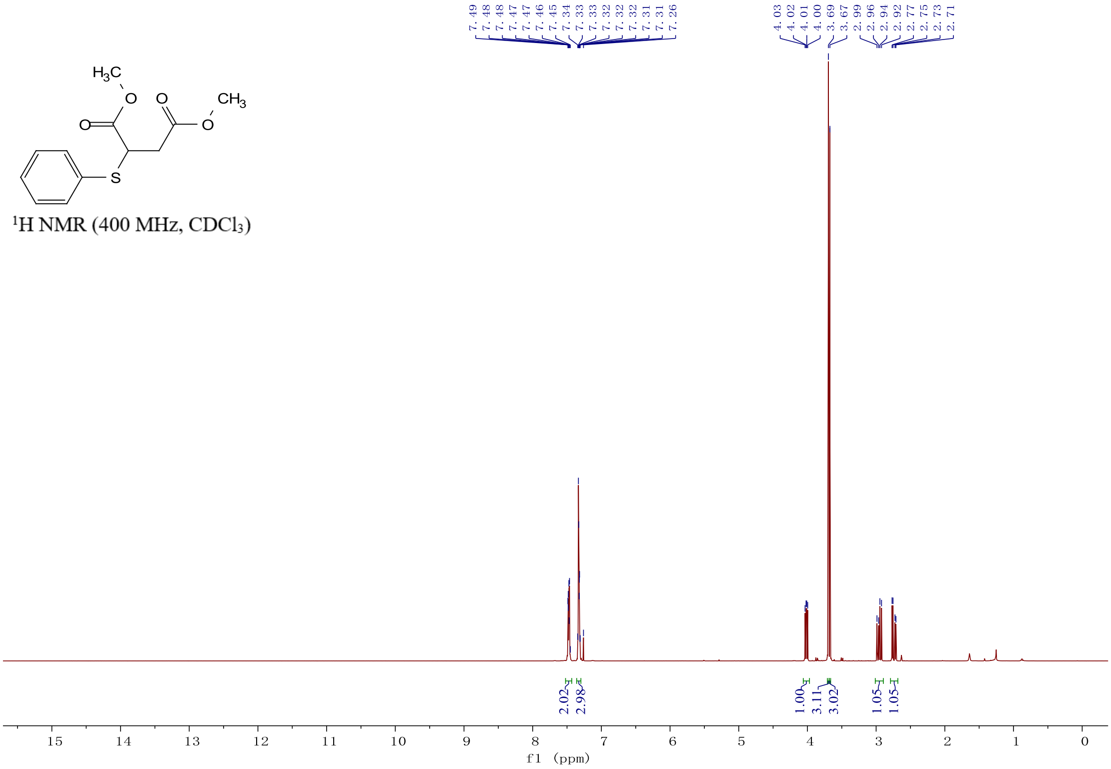

^{1}H NMR (400 MHz, $CDCl_3$)

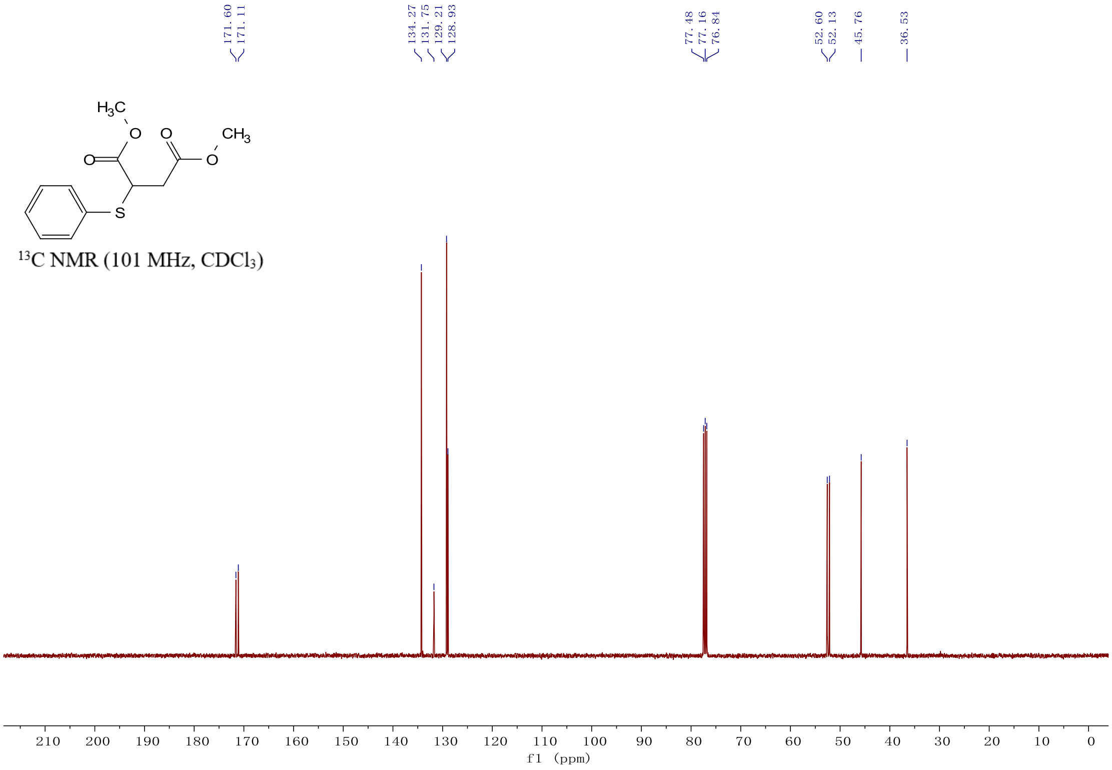

^{13}C NMR (101 MHz, $CDCl_3$)

dimethyl 2-((2-fluorophenyl)thio)succinate (2ab)

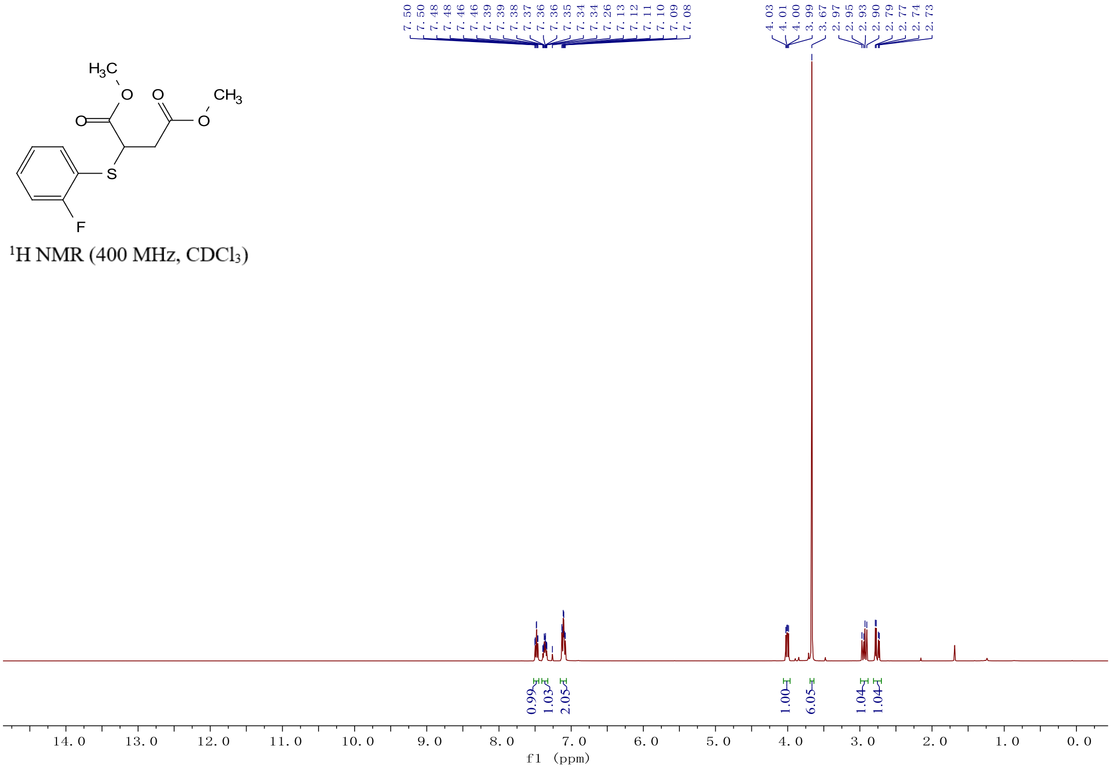

^{1}H NMR (400 MHz, $CDCl_3$)

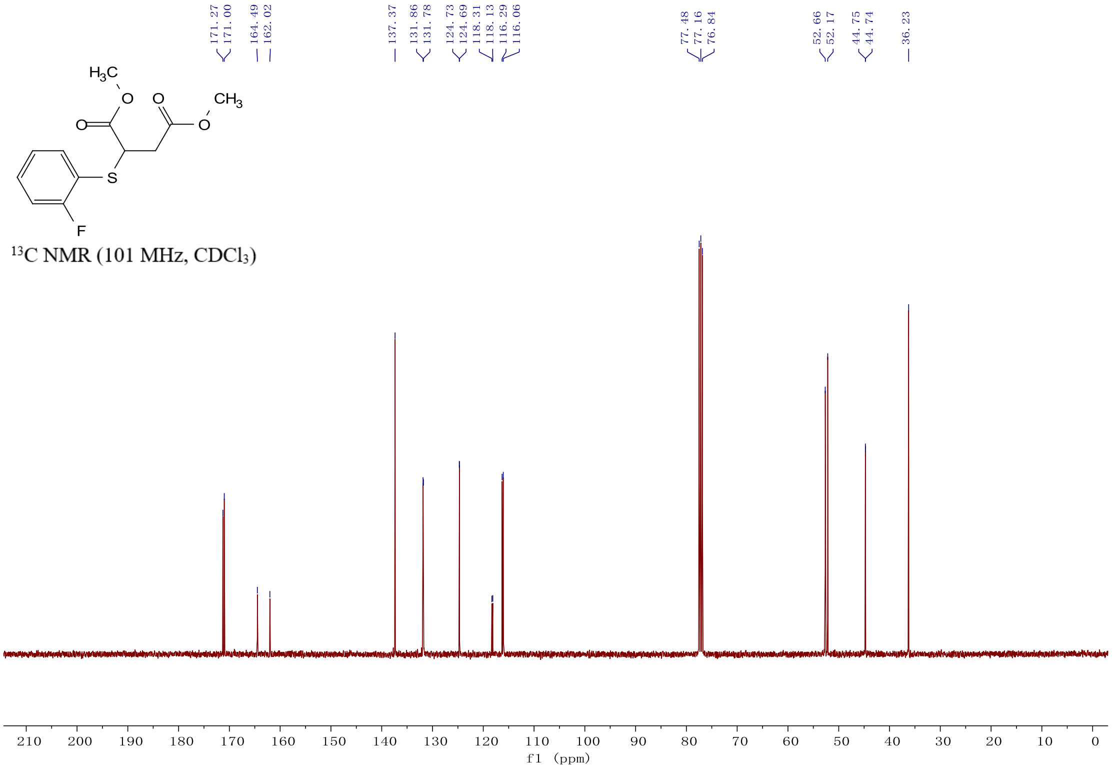

^{13}C NMR (101 MHz, $CDCl_3$)

dimethyl 2-((2-fluorophenyl)thio)succinate (2ab)

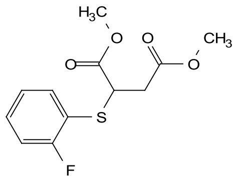

^{19}F NMR (376 MHz, $CDCl_3$)

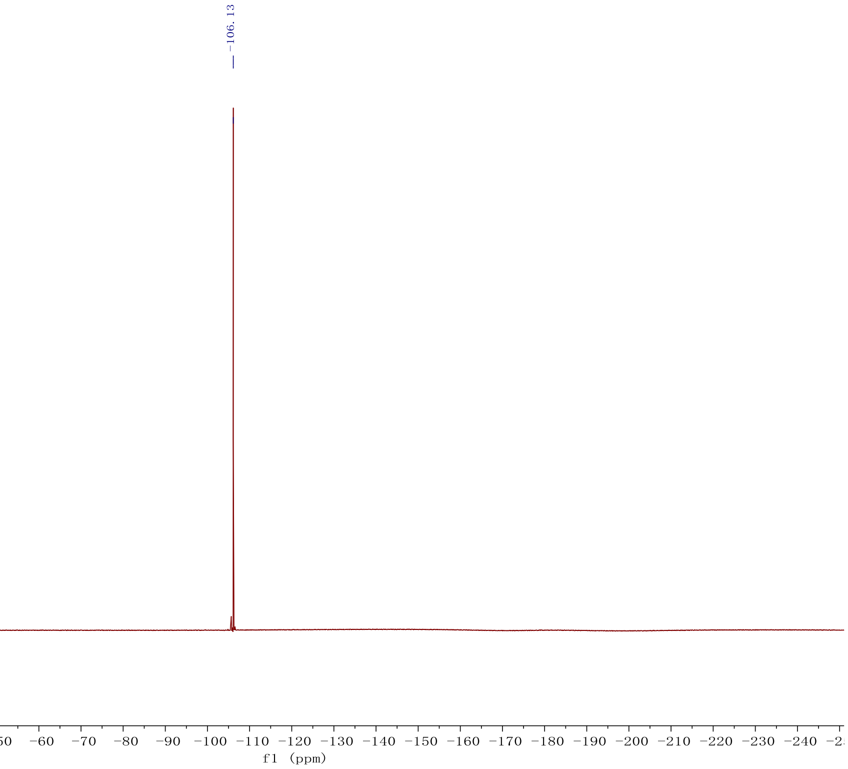

dimethyl 2-((2-chlorophenyl)thio)succinate (2ac)

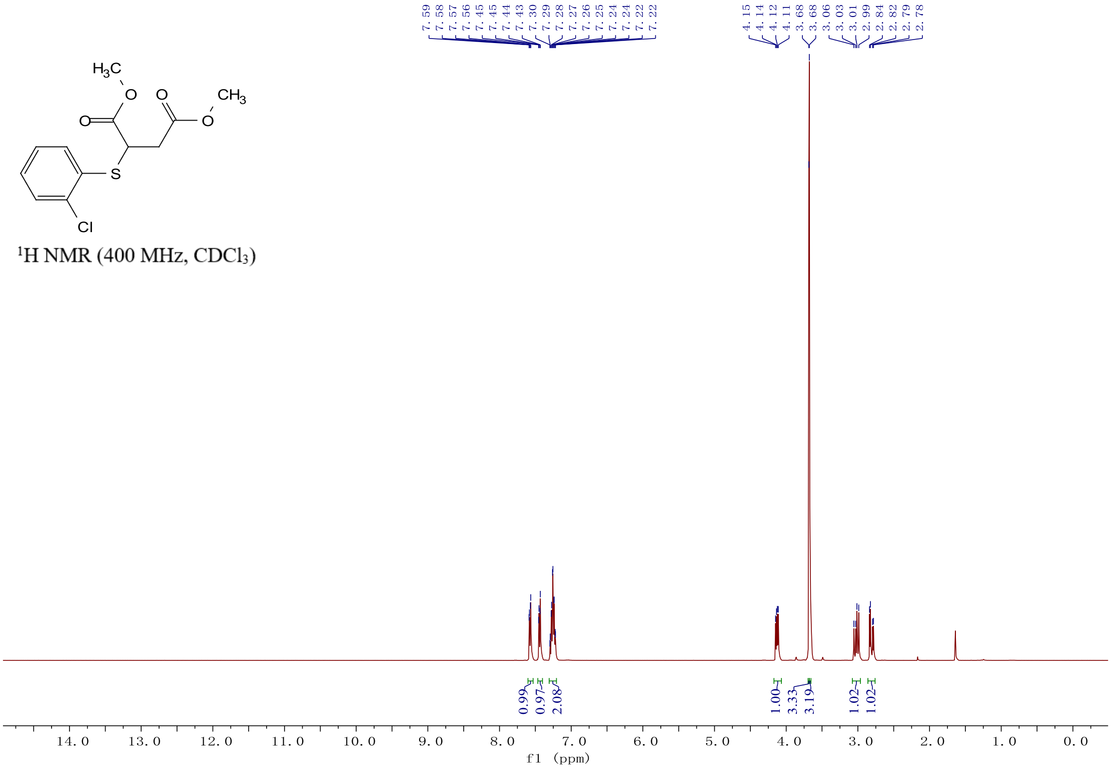

^{1}H NMR (400 MHz, $CDCl_3$)

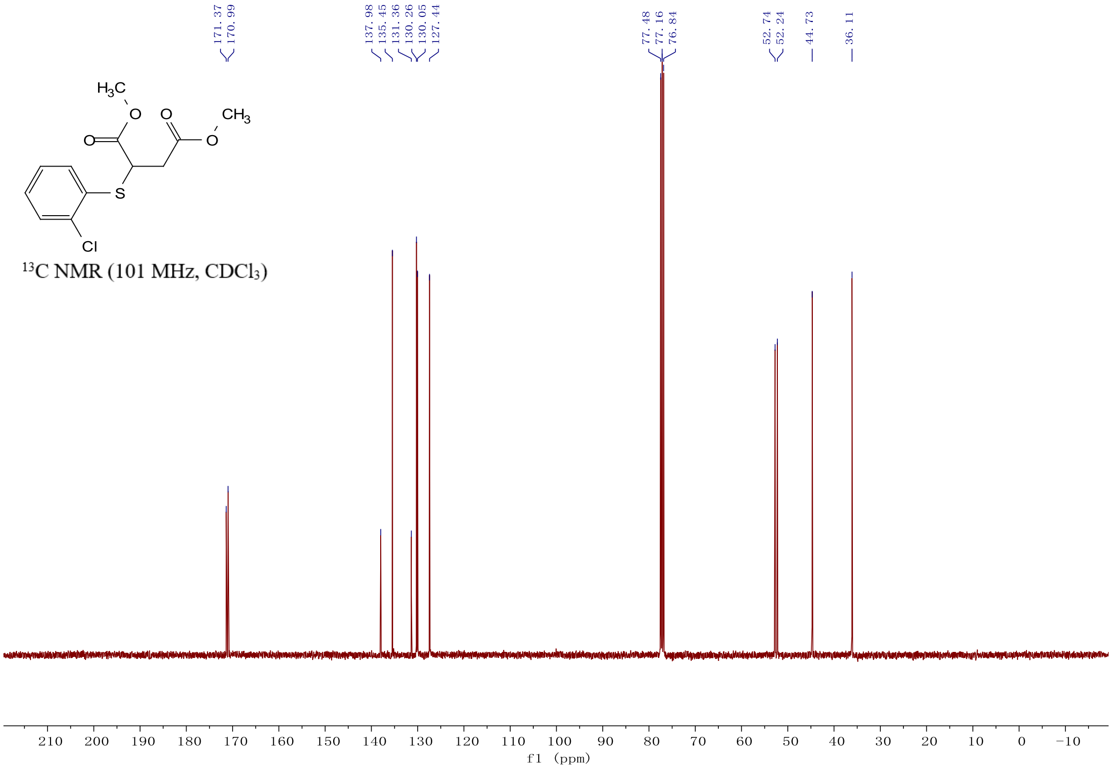

^{13}C NMR (101 MHz, $CDCl_3$)

dimethyl 2-((4-chlorophenyl)thio)succinate (2ad)

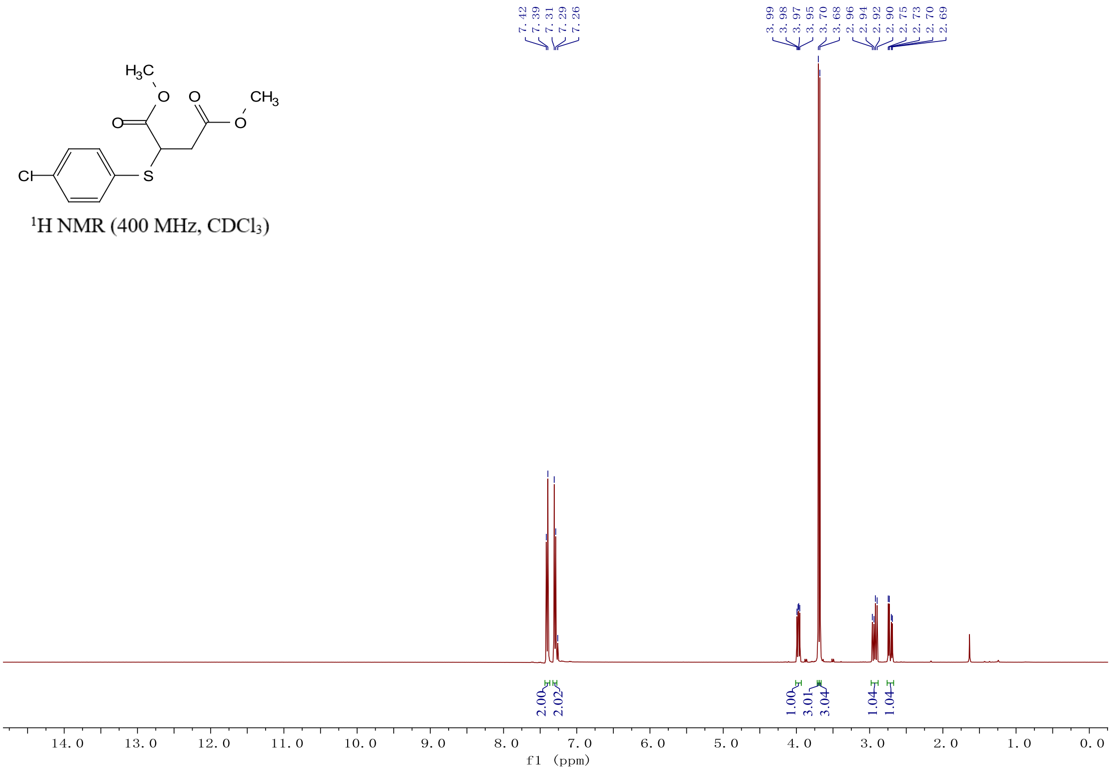

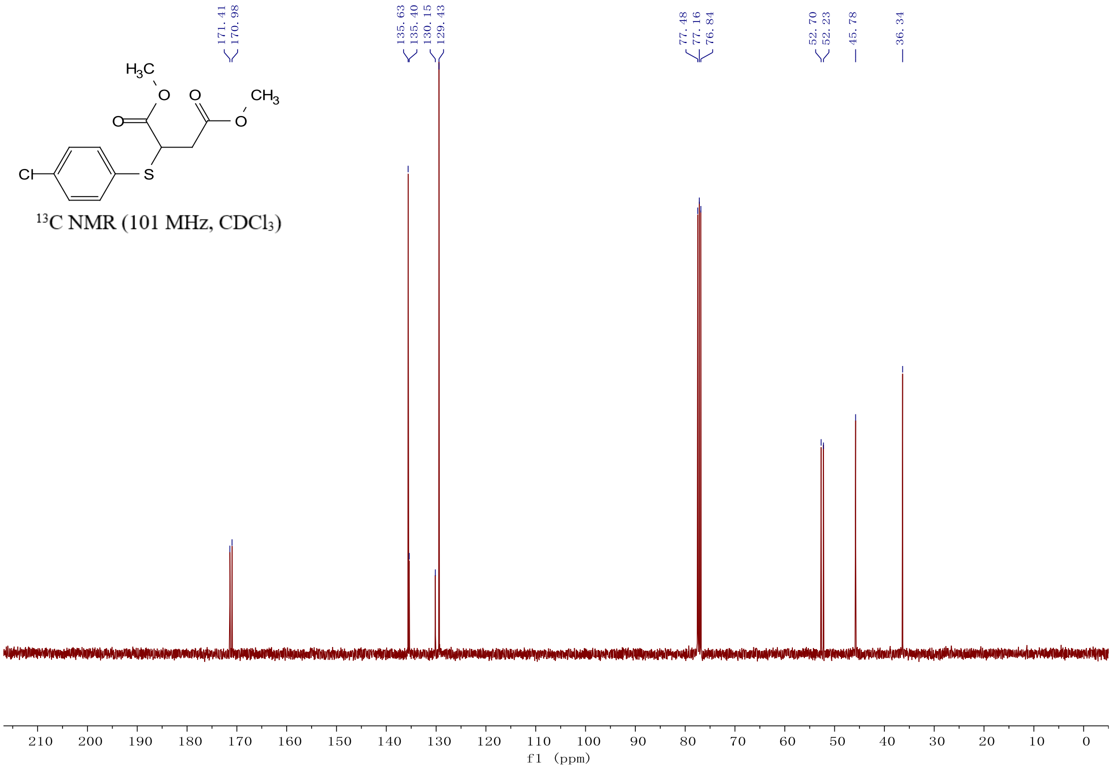

dimethyl 2-((2-bromophenyl)thio)succinate (2ae)

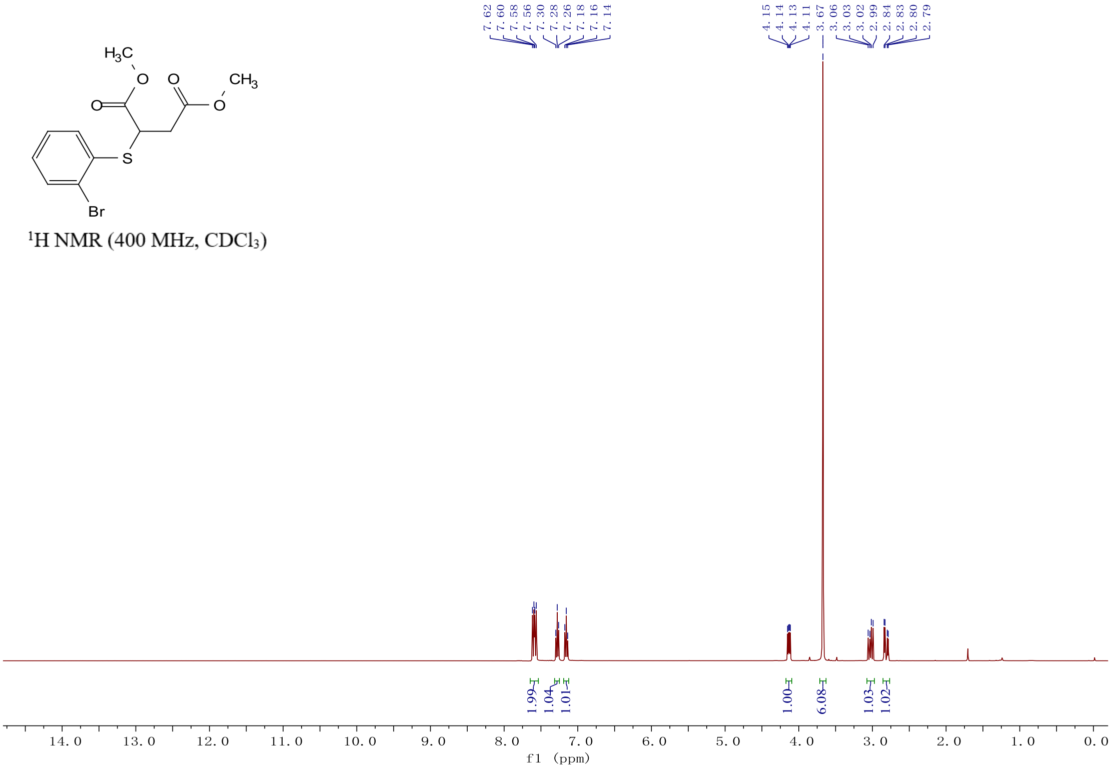

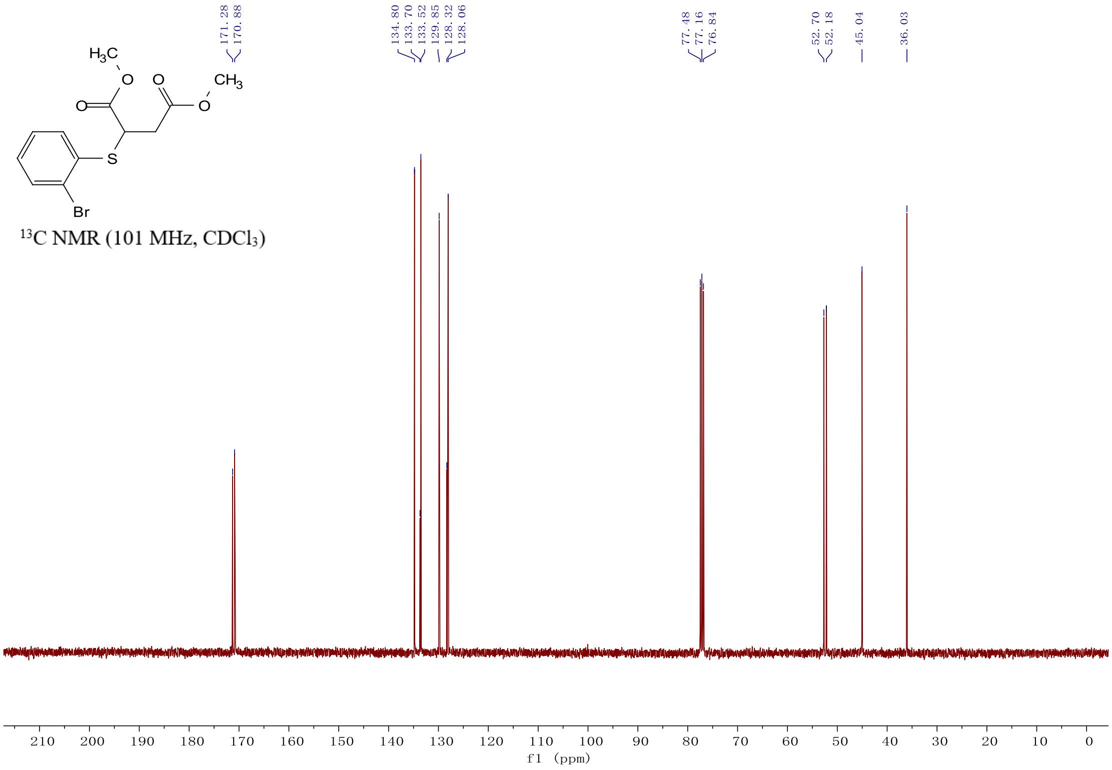

dimethyl 2-((4-bromophenyl)thio)succinate (2af)

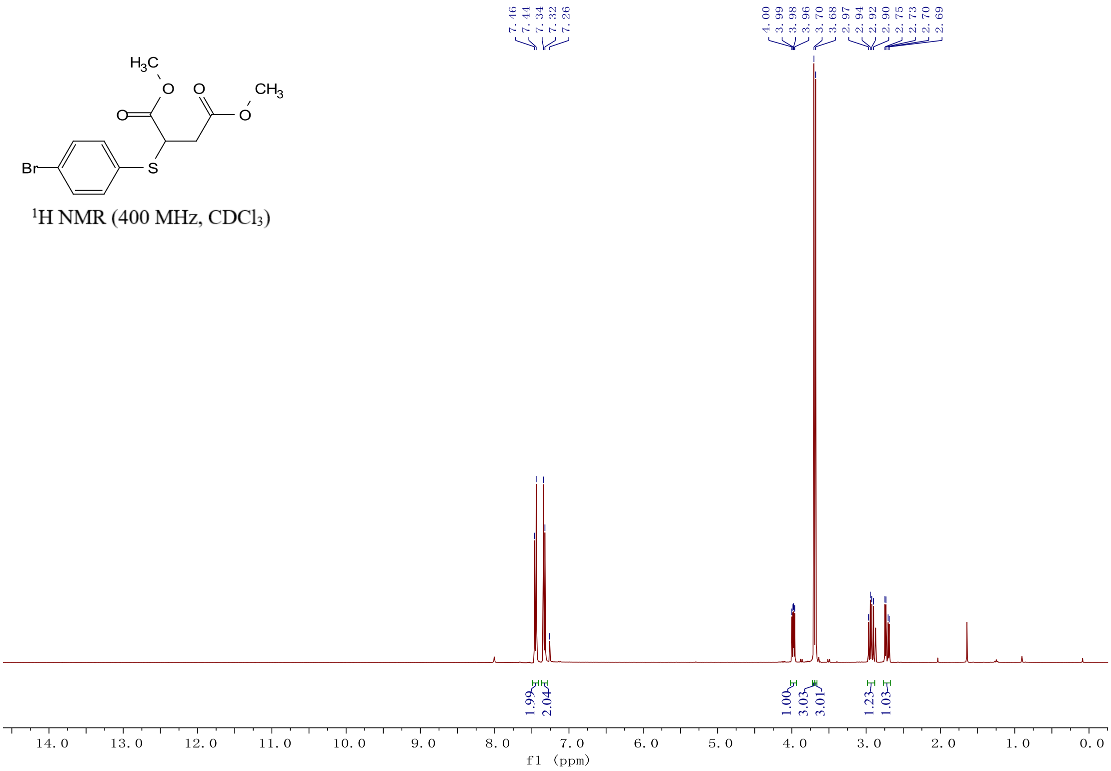

^{1}H NMR (400 MHz, CDCl$_3$)

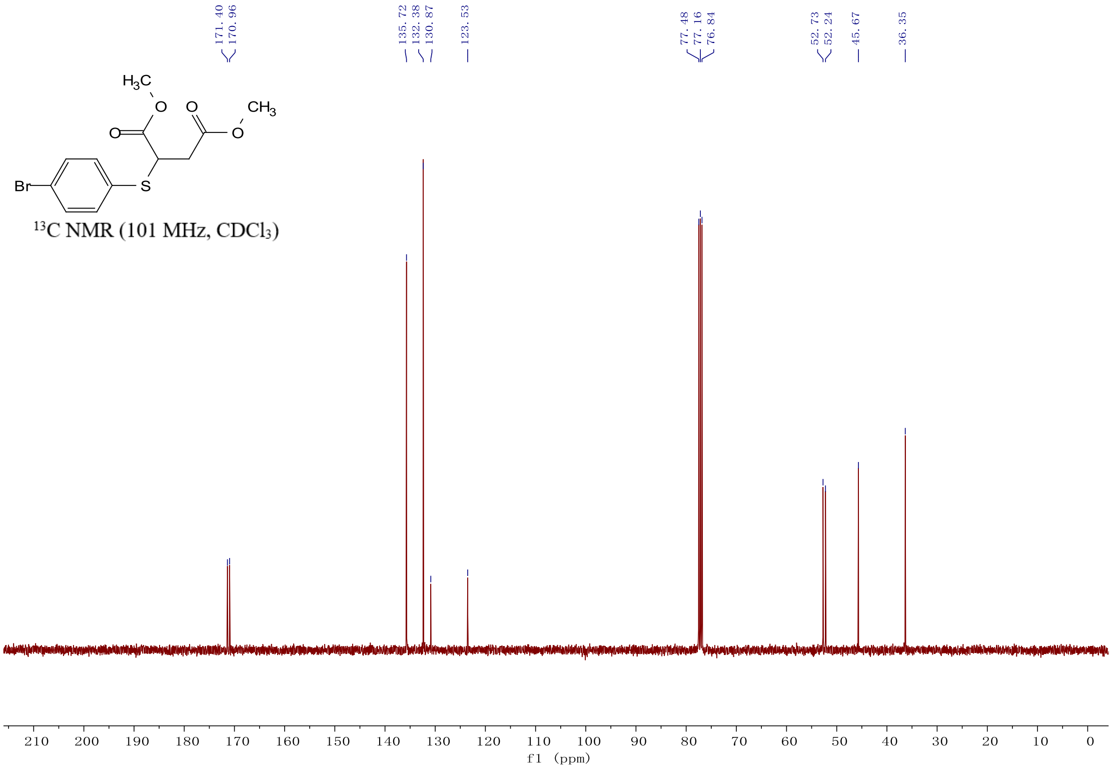

^{13}C NMR (101 MHz, CDCl$_3$)

dimethyl 2-(*p*-tolylthio)succinate (2ag)

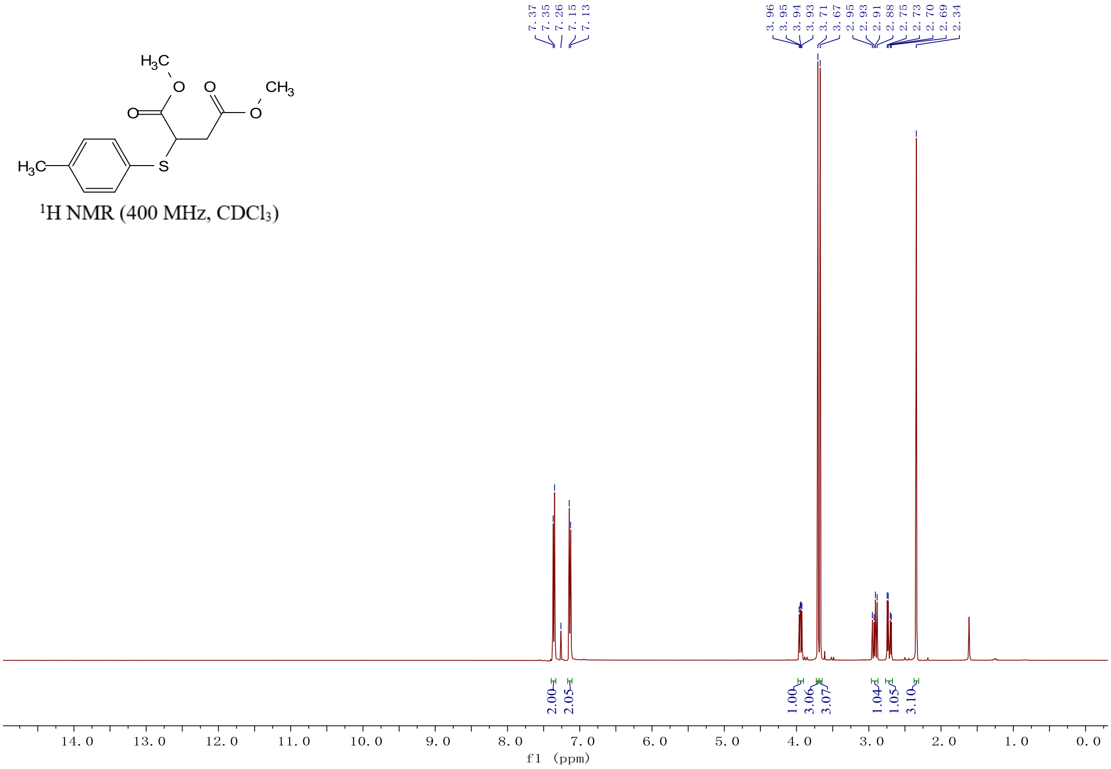

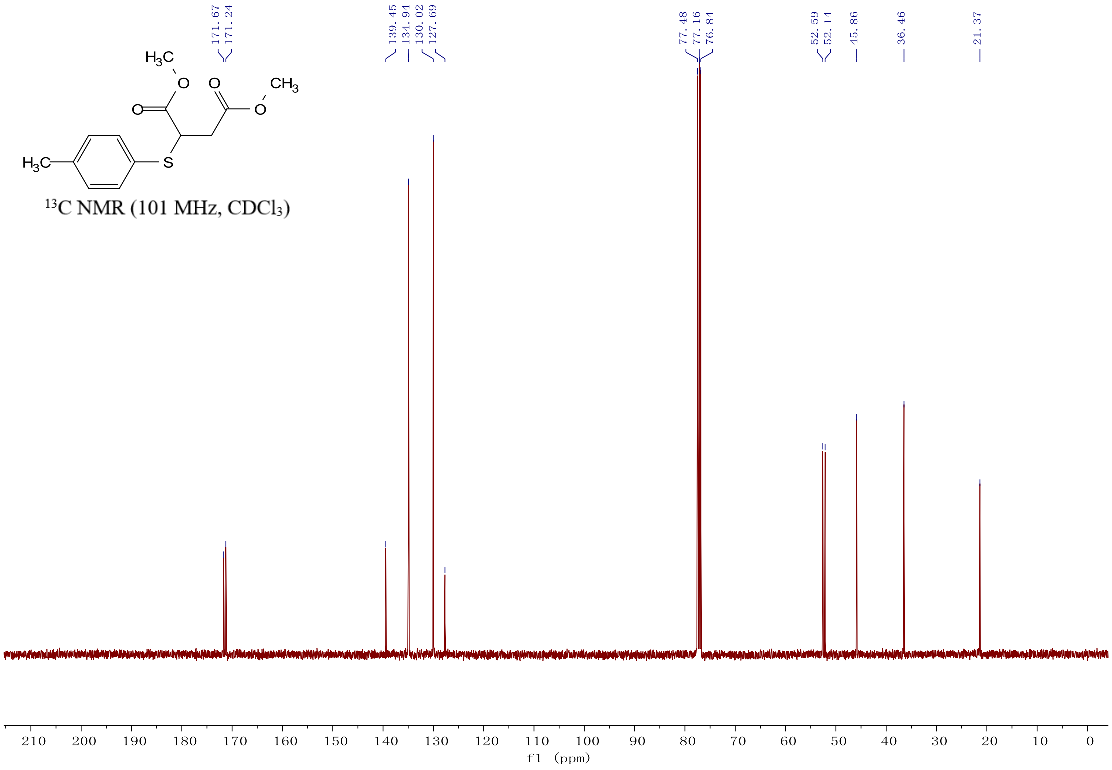

dimethyl 2-((3-methoxyphenyl)thio)succinate (2ah)

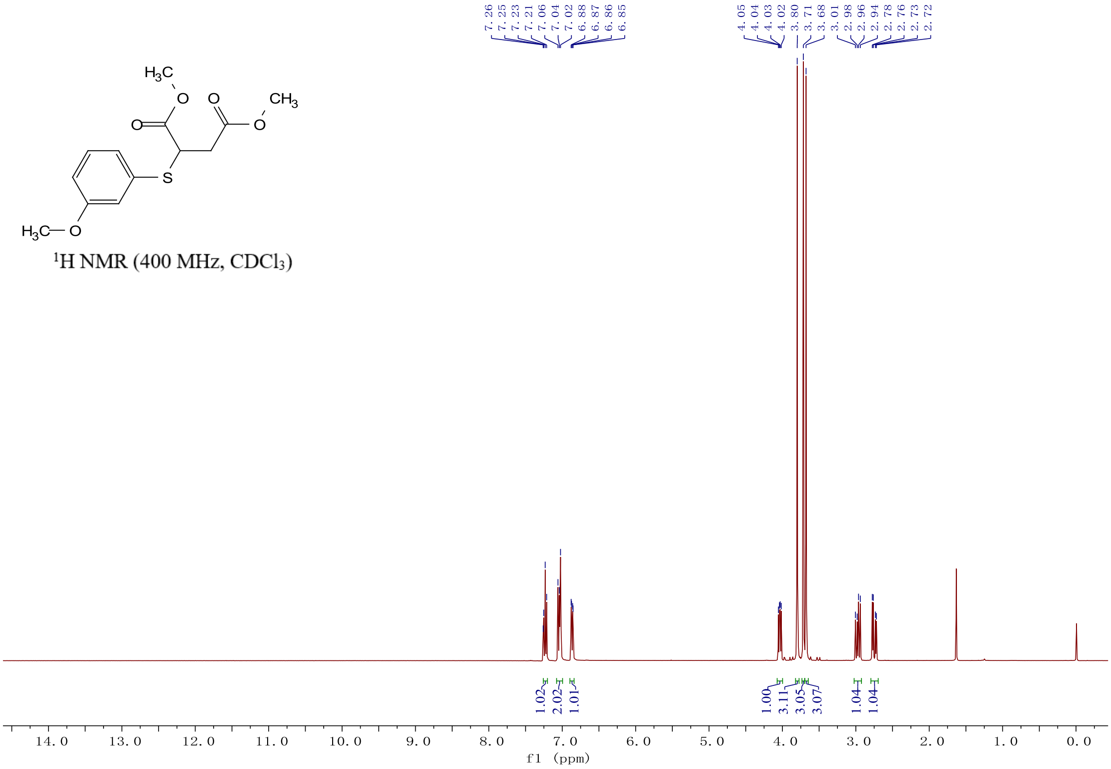

^{1}H NMR (400 MHz, $CDCl_3$)

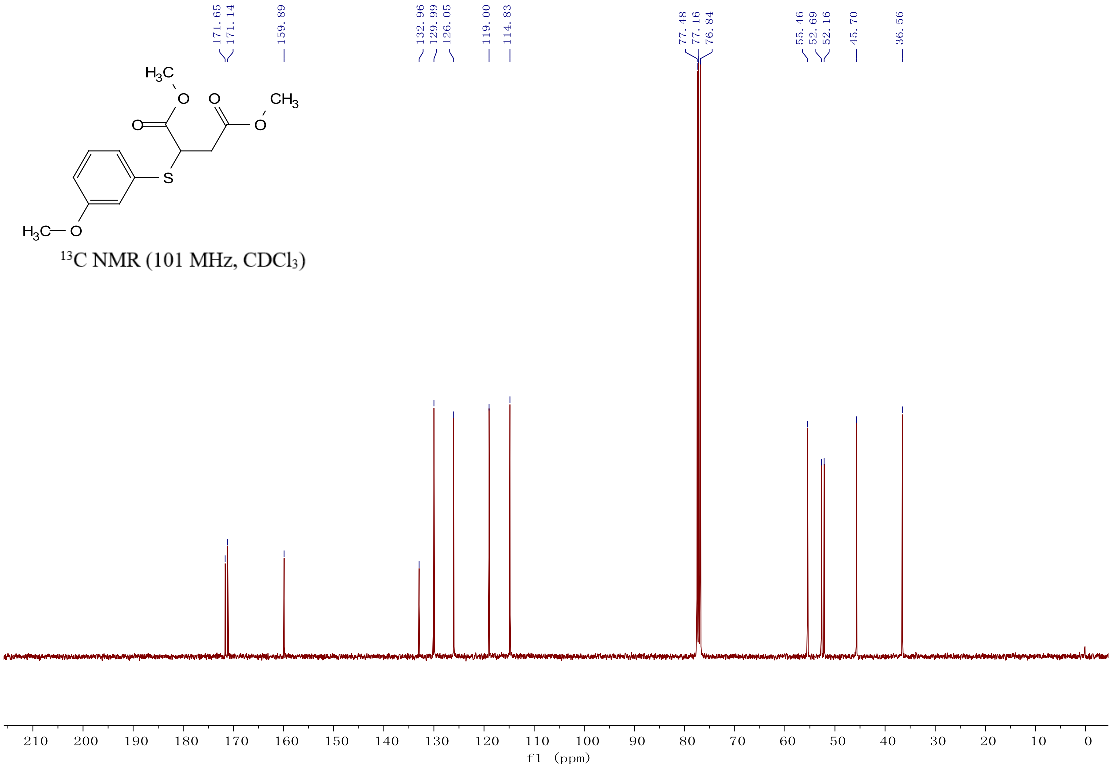

^{13}C NMR (101 MHz, $CDCl_3$)

dimethyl 2-((4-methoxyphenyl)thio)succinate (2ai)

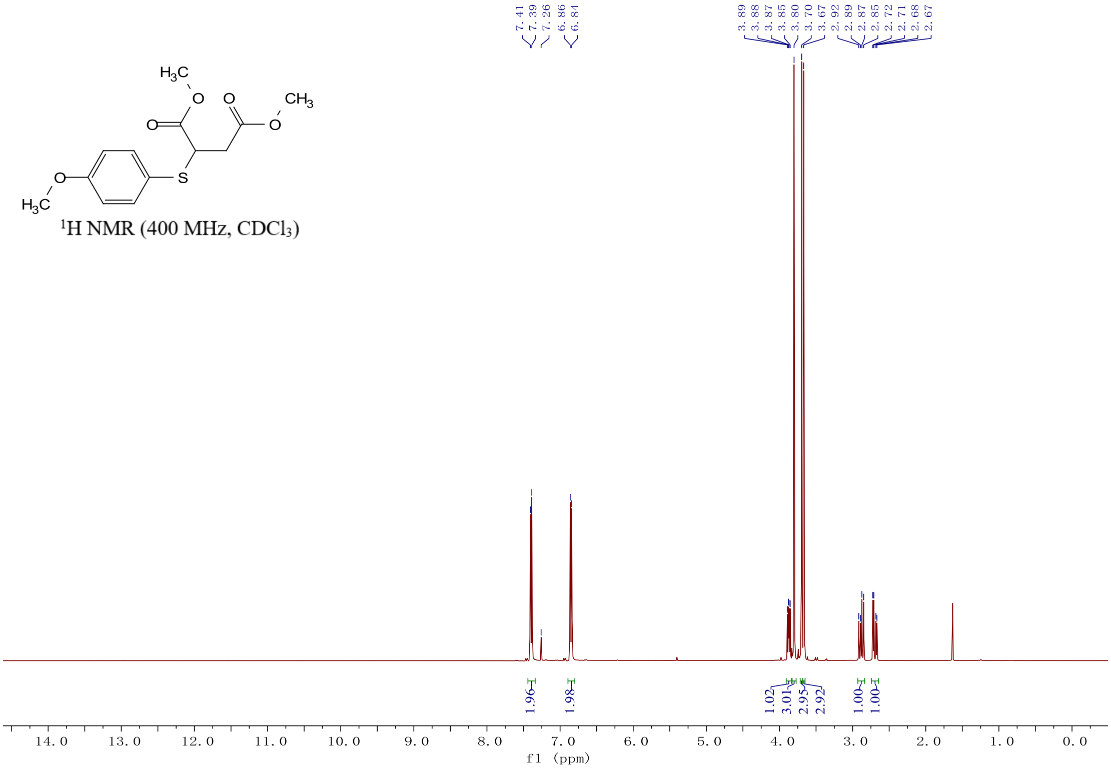

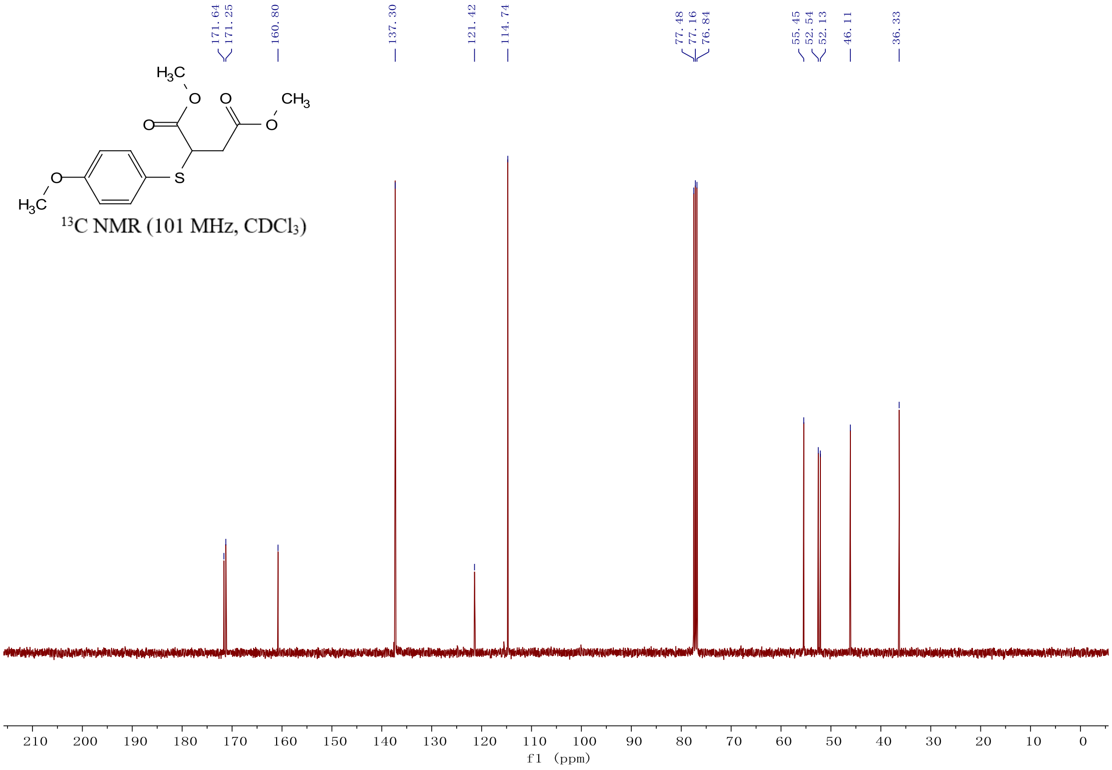

dimethyl 2-((3,5-dimethylphenyl)thio)succinate (2aj)

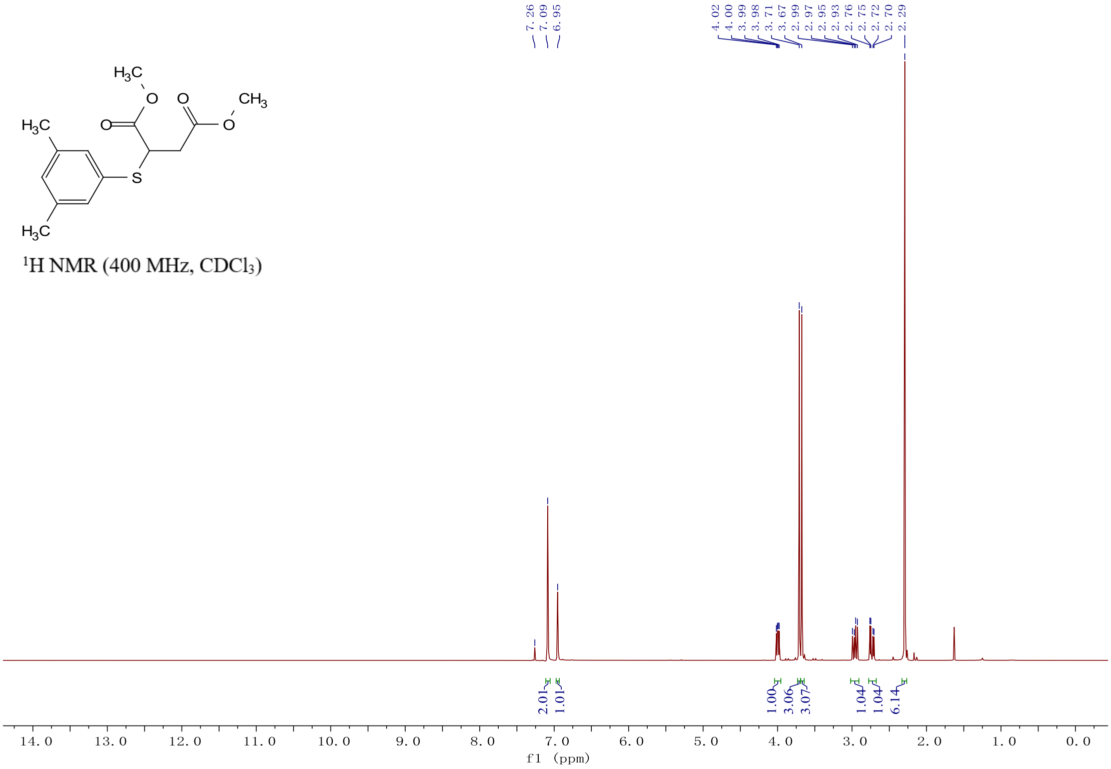

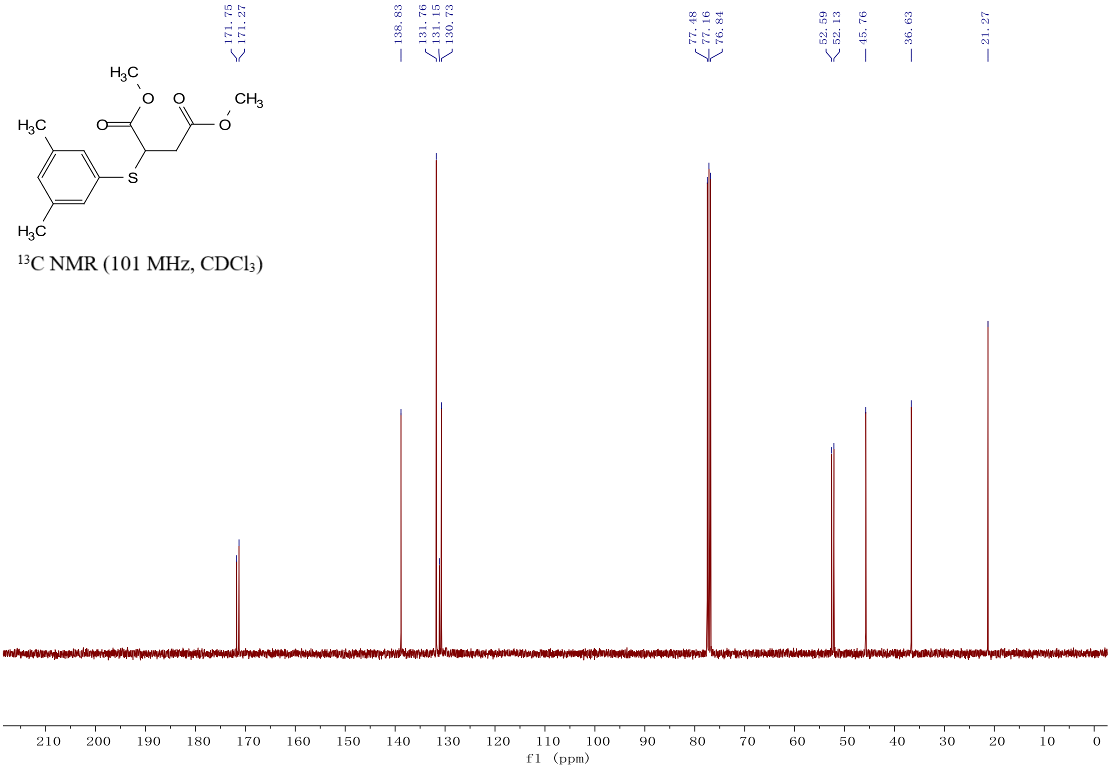

dimethyl 2-(naphthalen-1-ylthio)succinate (2ak)

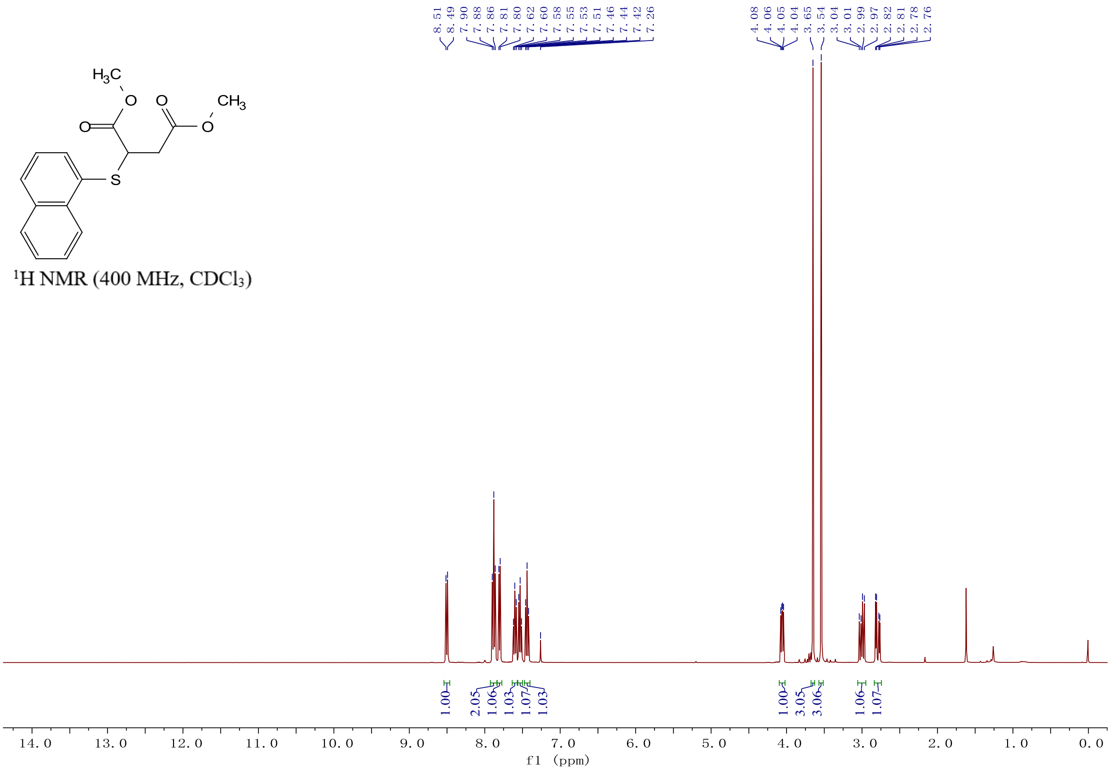

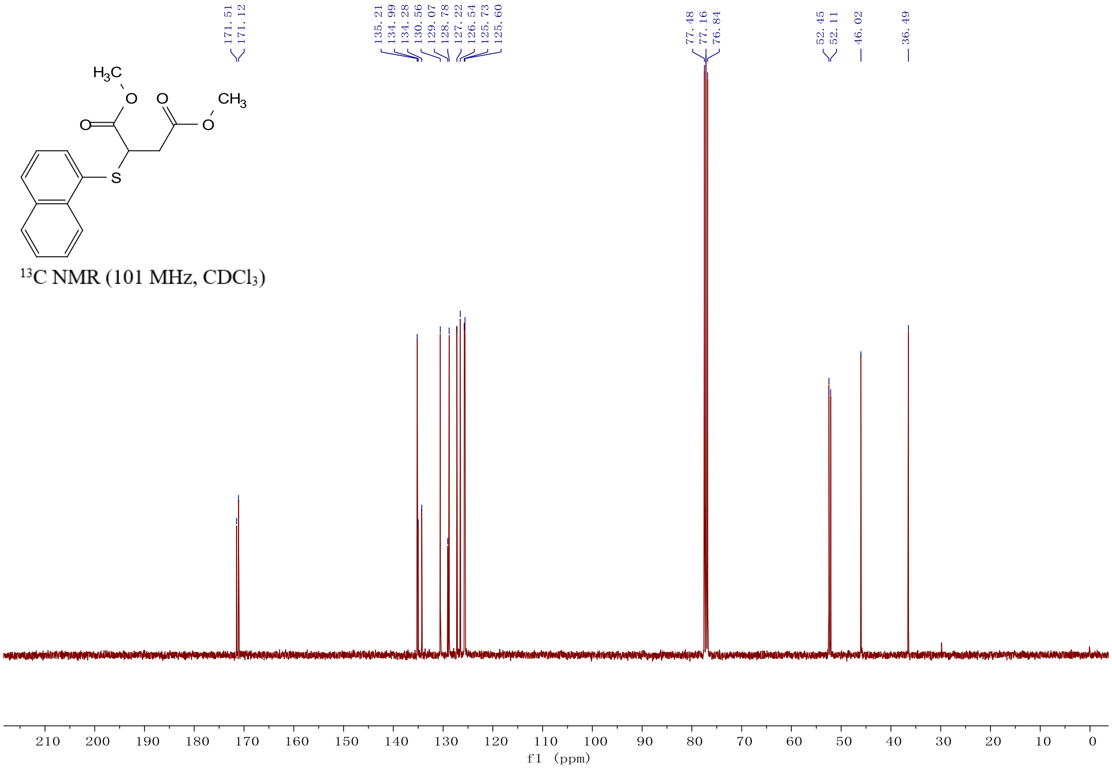

dimethyl 2-(naphthalen-2-ylthio)succinate (2al)

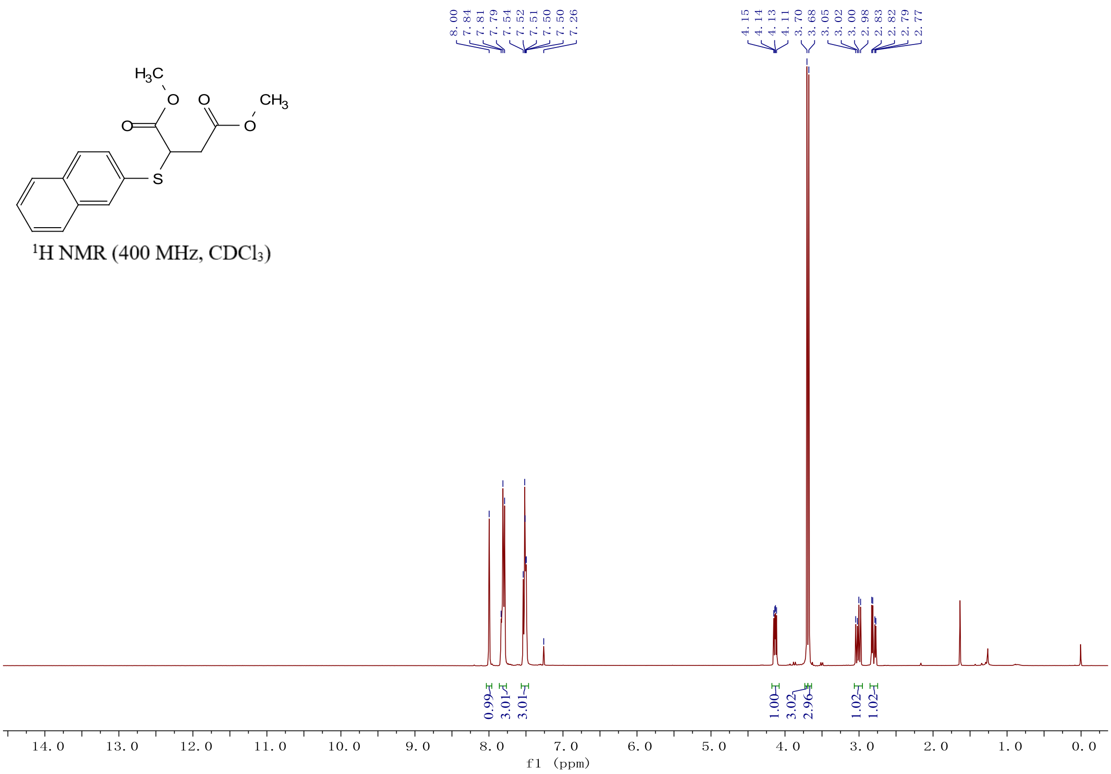

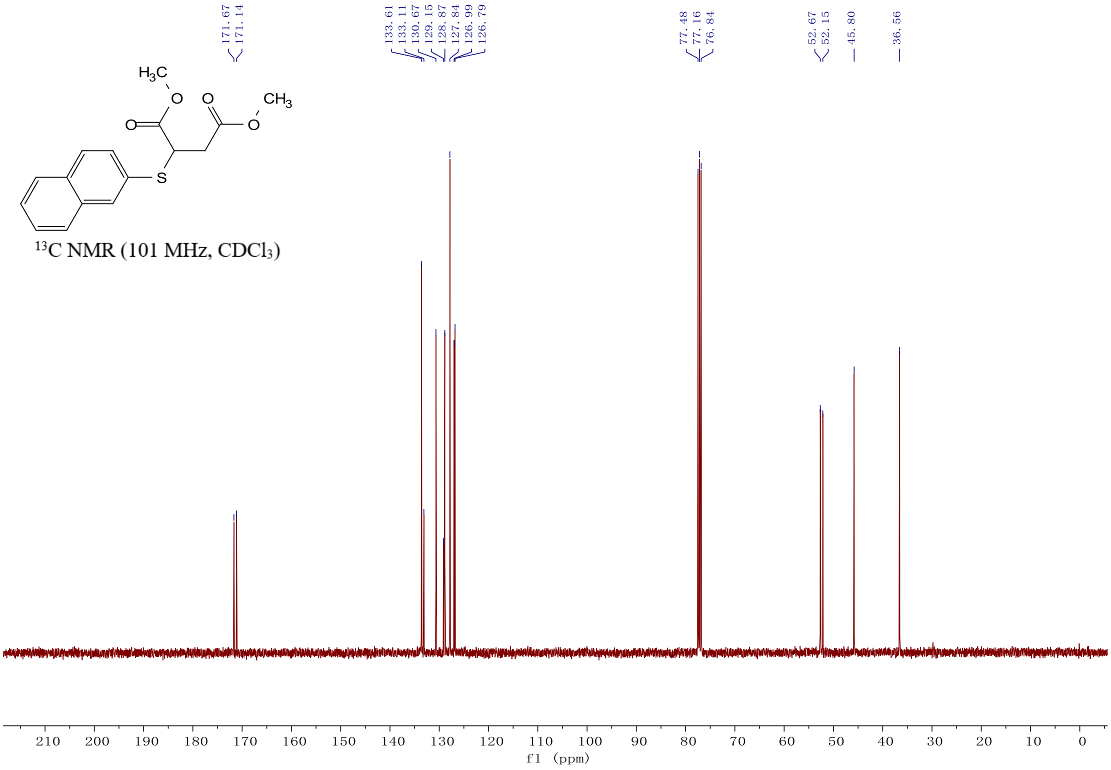

dimethyl 2-((furan-2-ylmethyl)thio)succinate (2am)

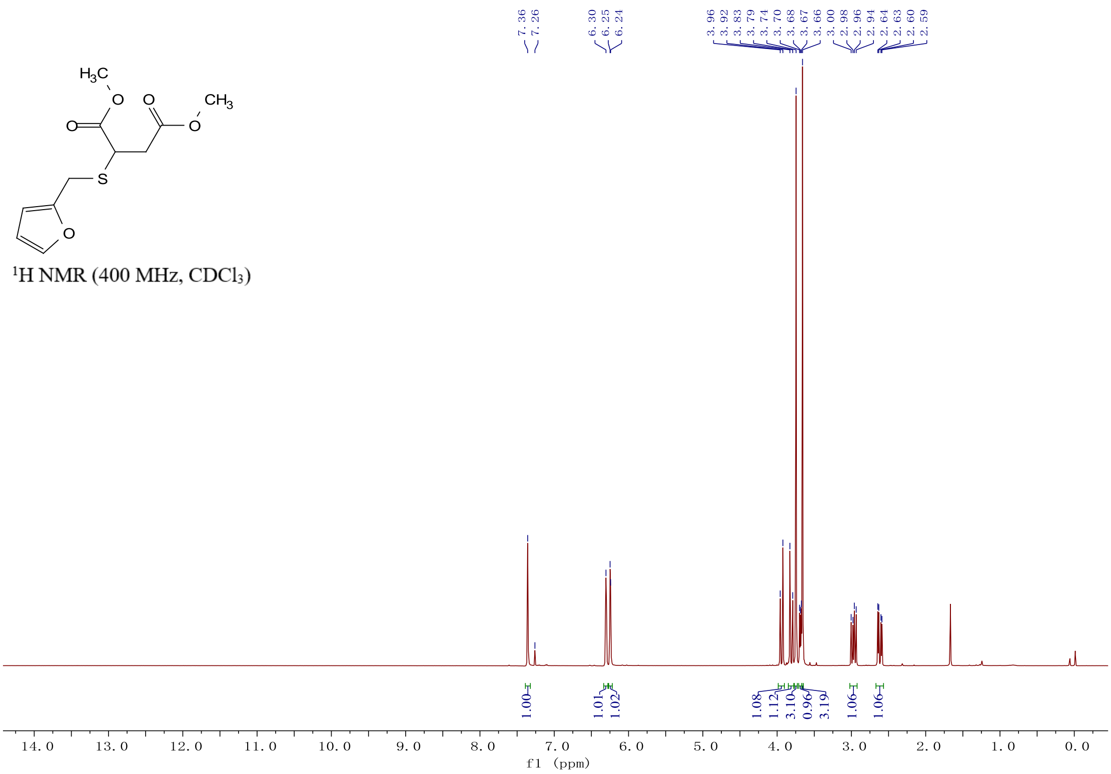

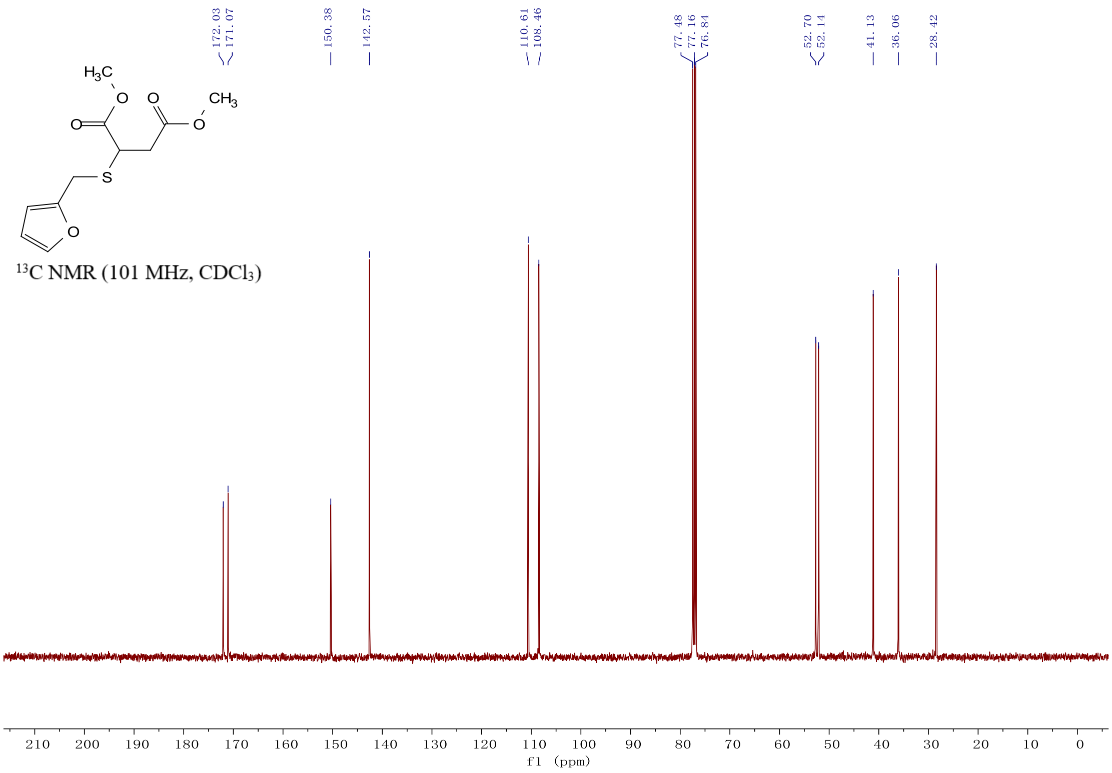

dimethyl 2-(benzylthio)succinate (2an)

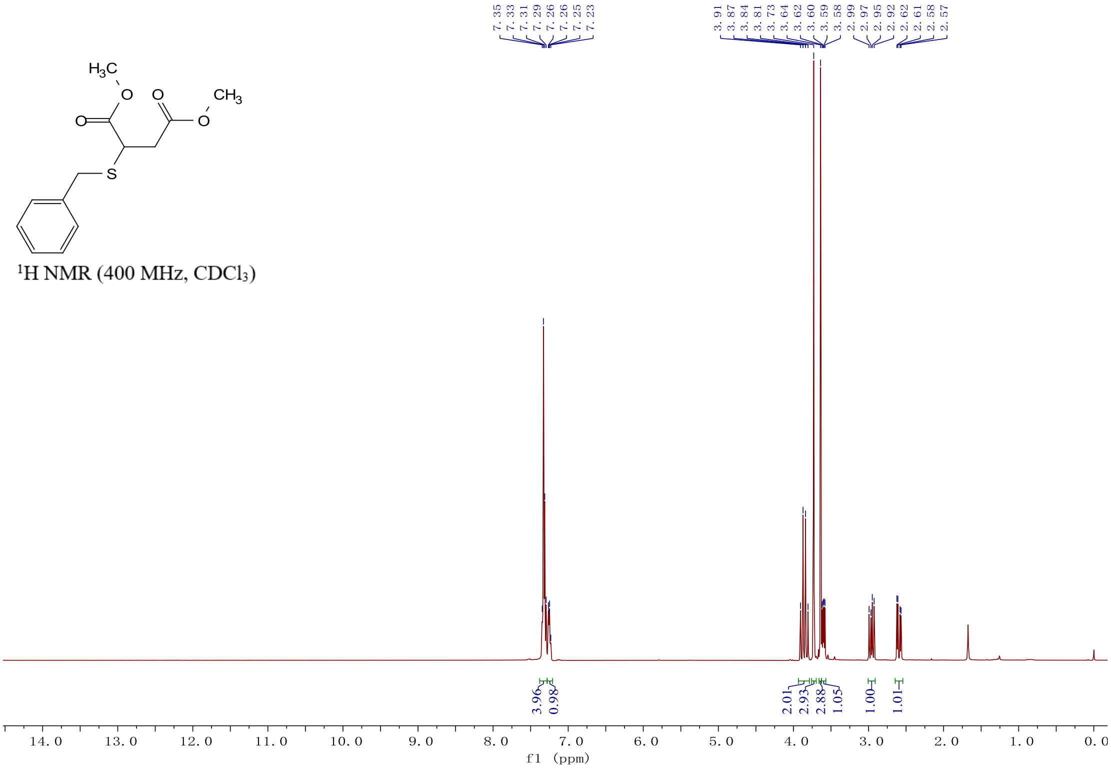

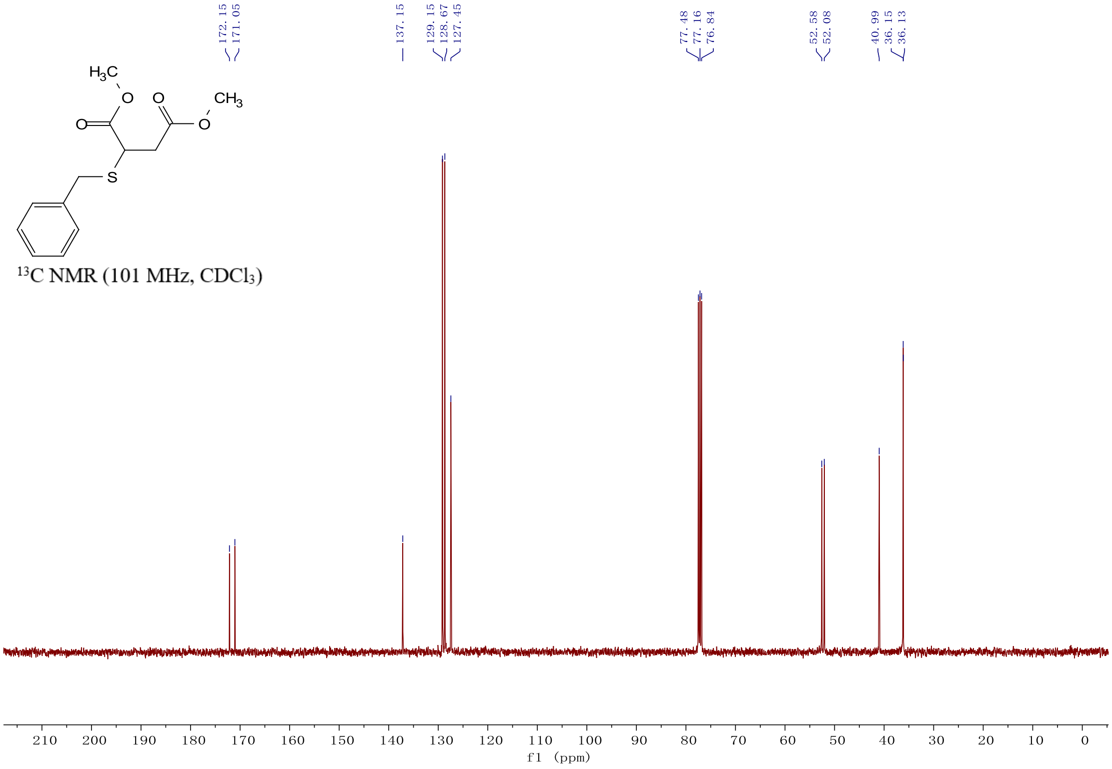

dimethyl 2-((4-methylbenzyl)thio)succinate (2ao)

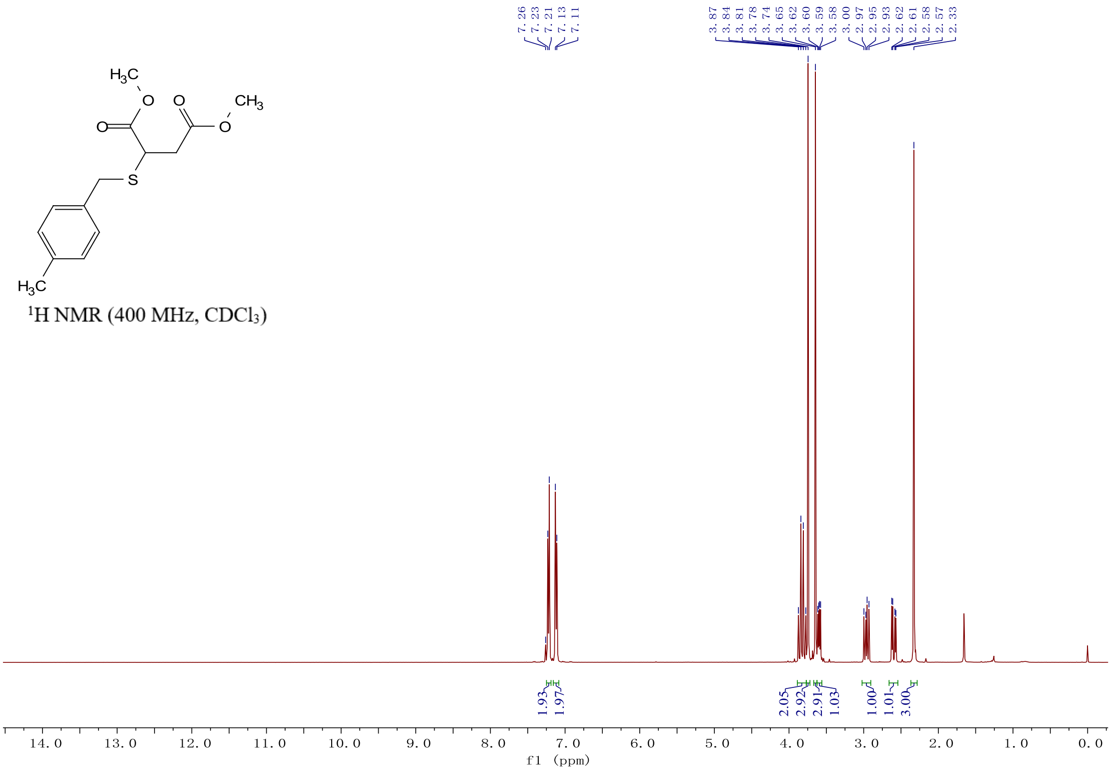

^{1}H NMR (400 MHz, $CDCl_3$)

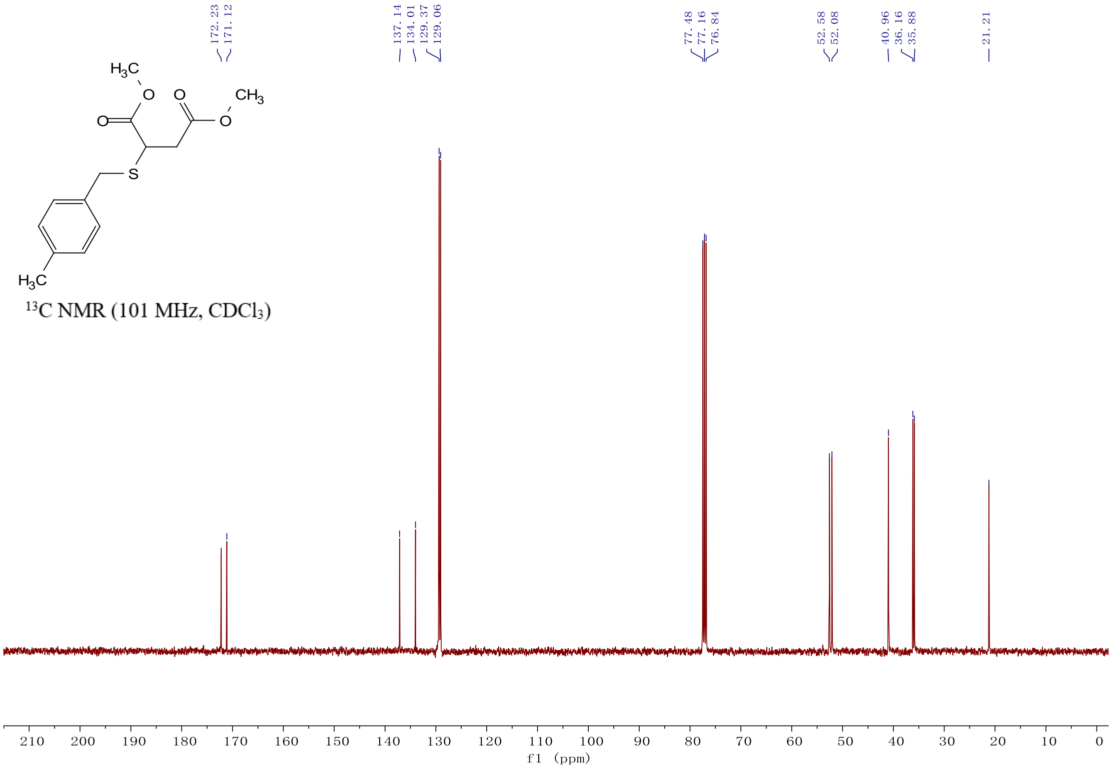

^{13}C NMR (101 MHz, $CDCl_3$)

dimethyl 2-((4-methoxybenzyl)thio)succinate (2ap)

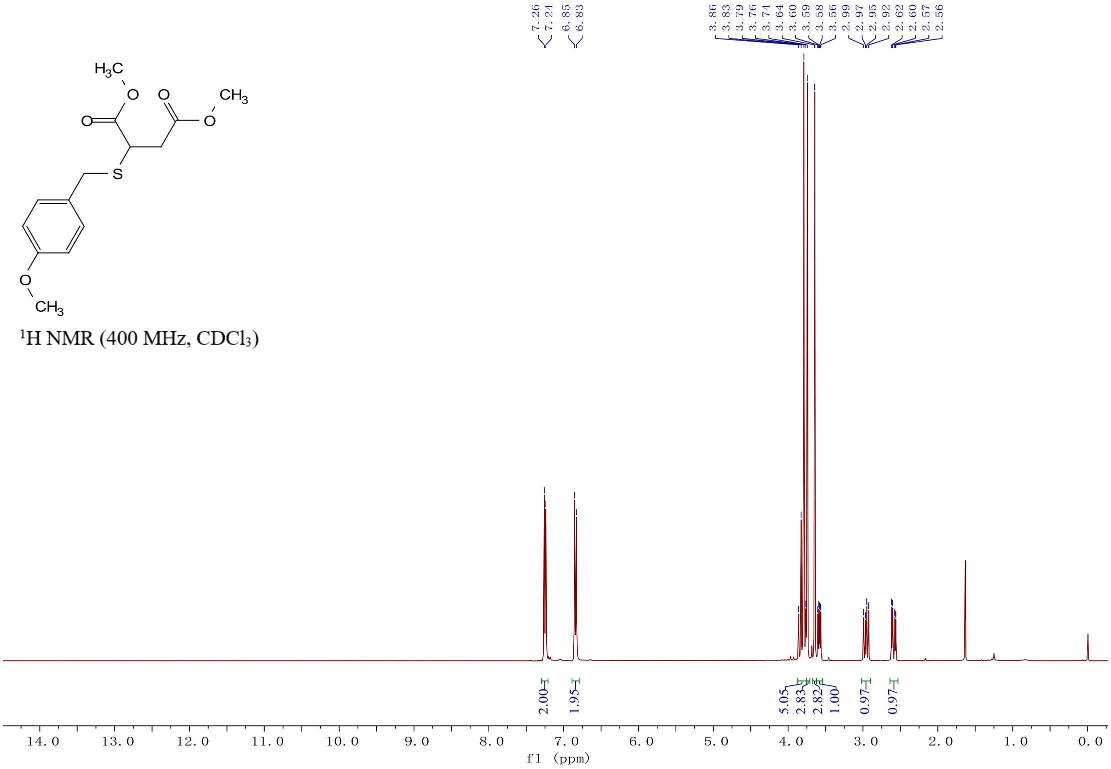

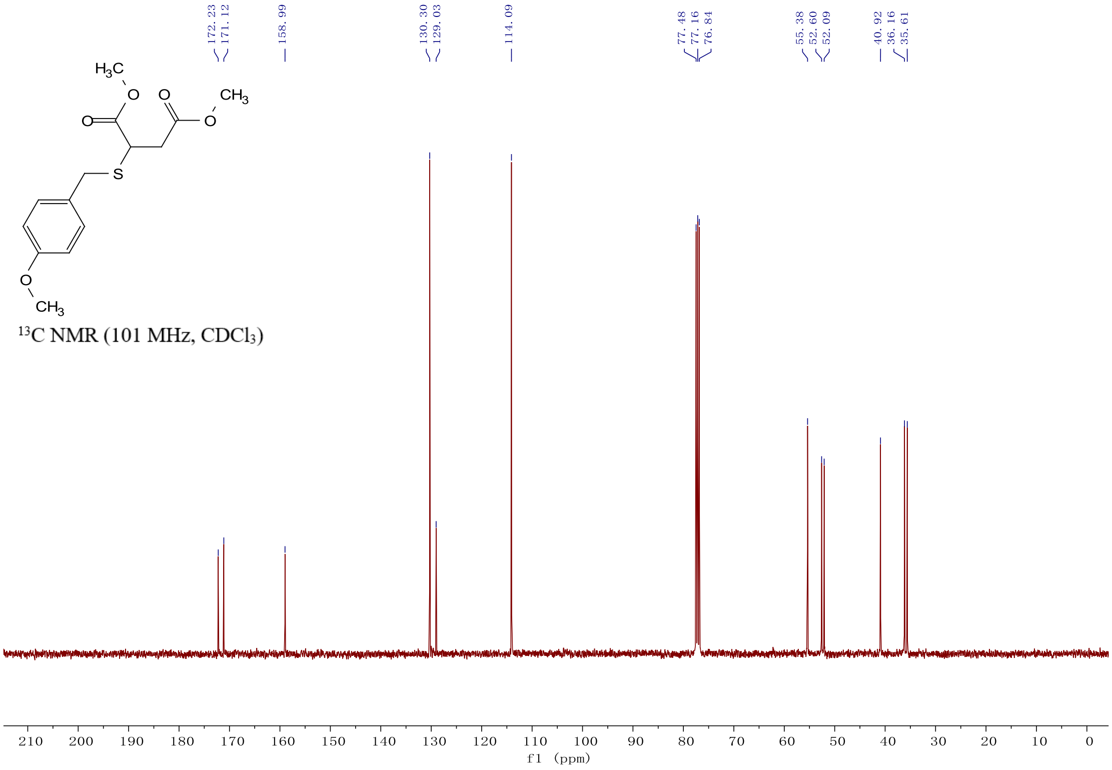

dimethyl 2-(phenethylthio)succinate (2aq)

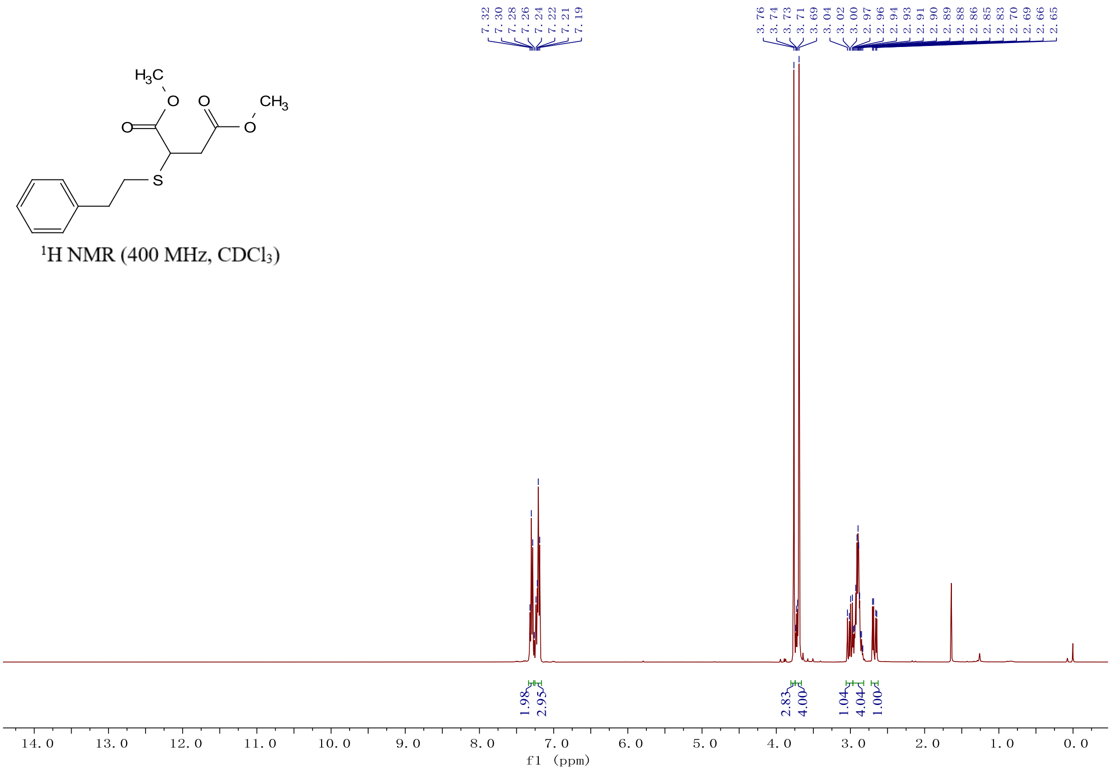

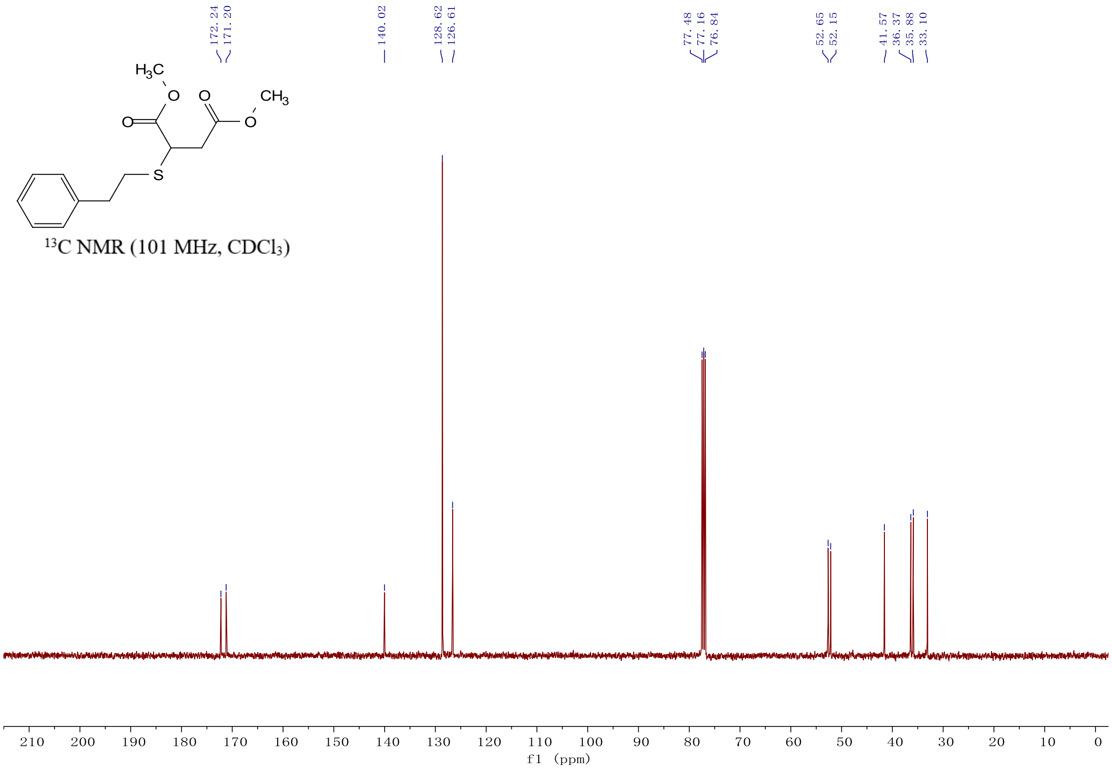

dimethyl 2-(pentylthio)succinate (2ar)

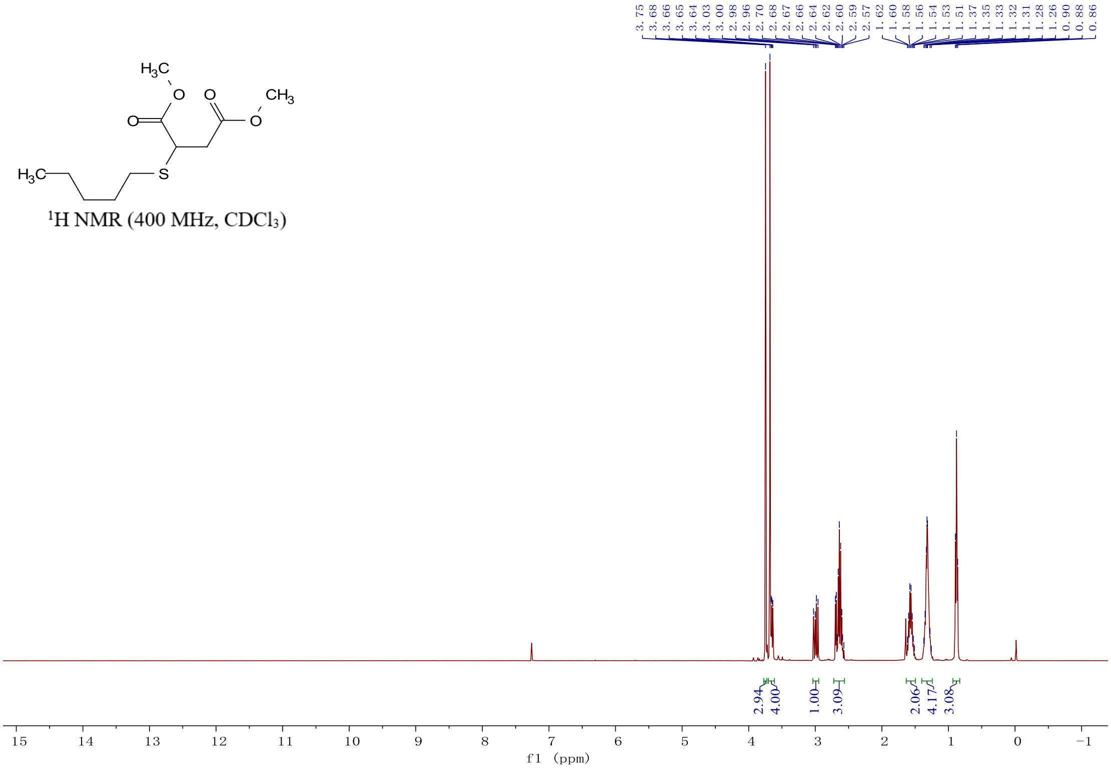

^{1}H NMR (400 MHz, $CDCl_3$)

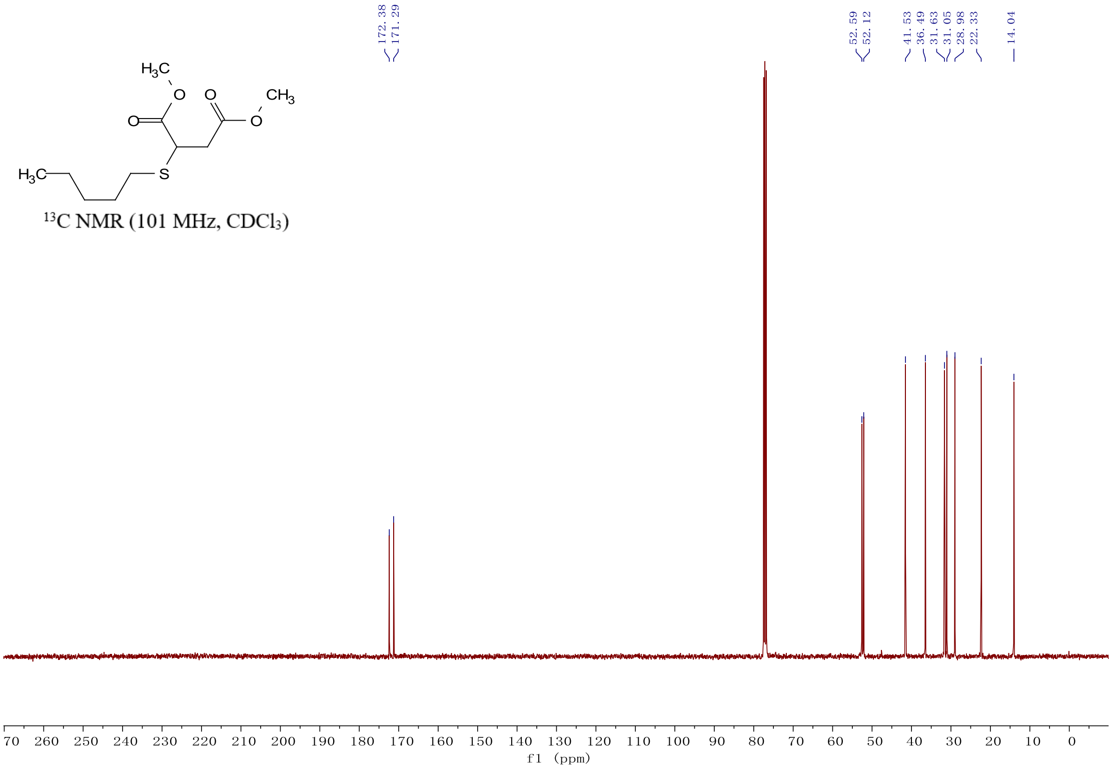

^{13}C NMR (101 MHz, $CDCl_3$)

dimethyl 2-(cyclohexylthio)succinate (2as)

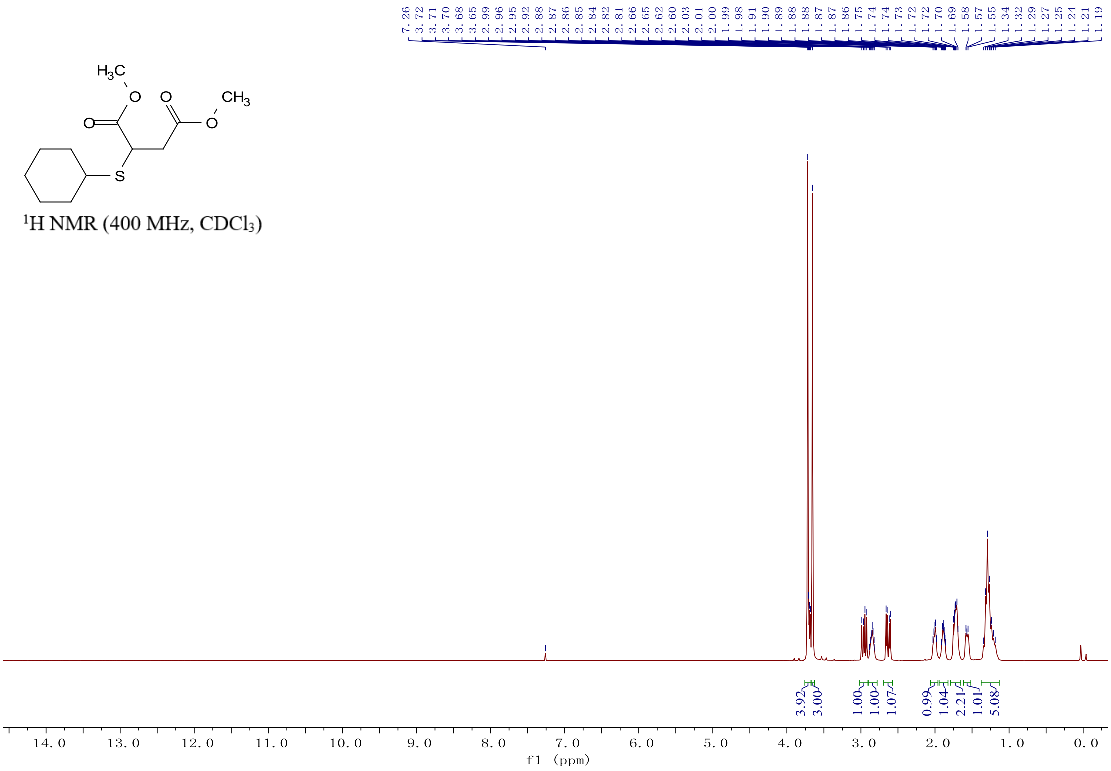

^{1}H NMR (400 MHz, $CDCl_3$)

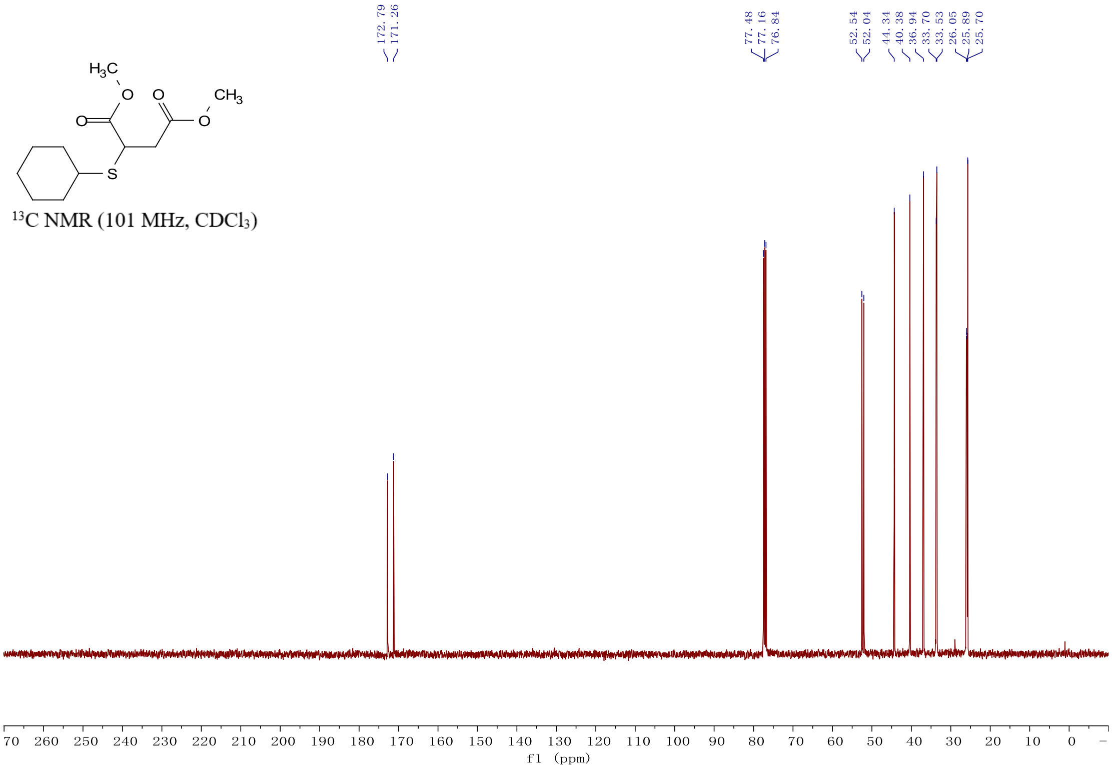

^{13}C NMR (101 MHz, $CDCl_3$)

dimethyl 2-((2-ethoxy-2-oxoethyl)thio)succinate (2at)

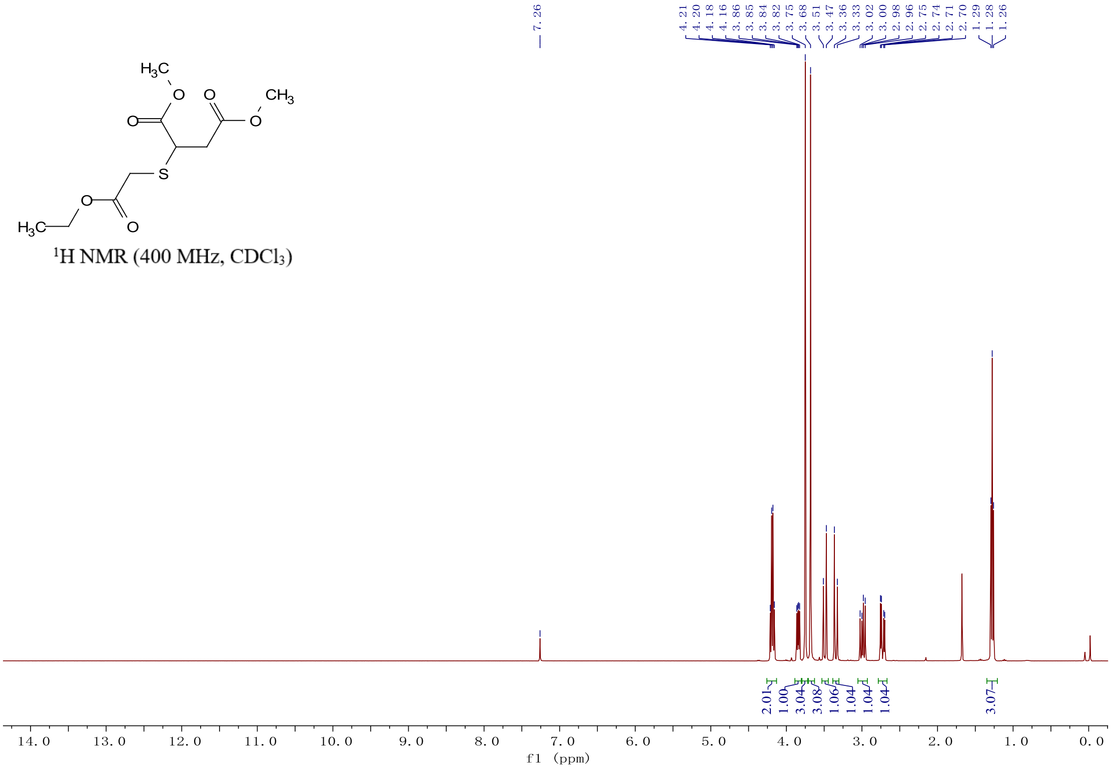

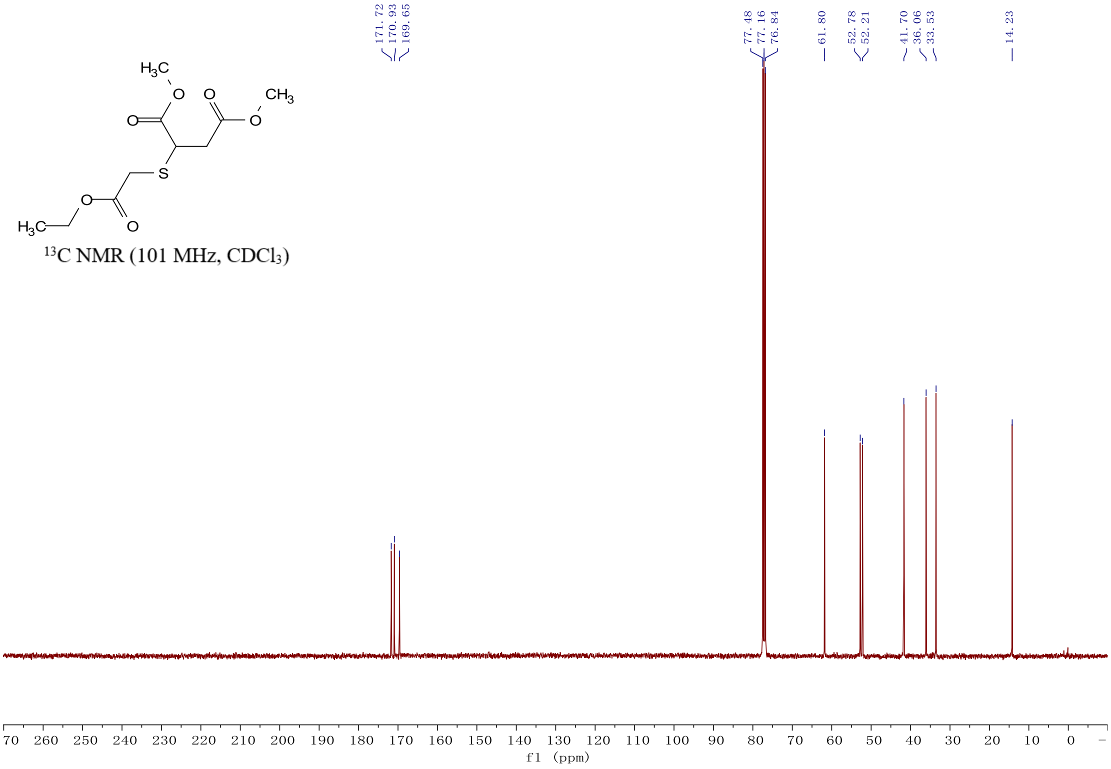

diethyl 2-(phenylthio)succinate (2au)

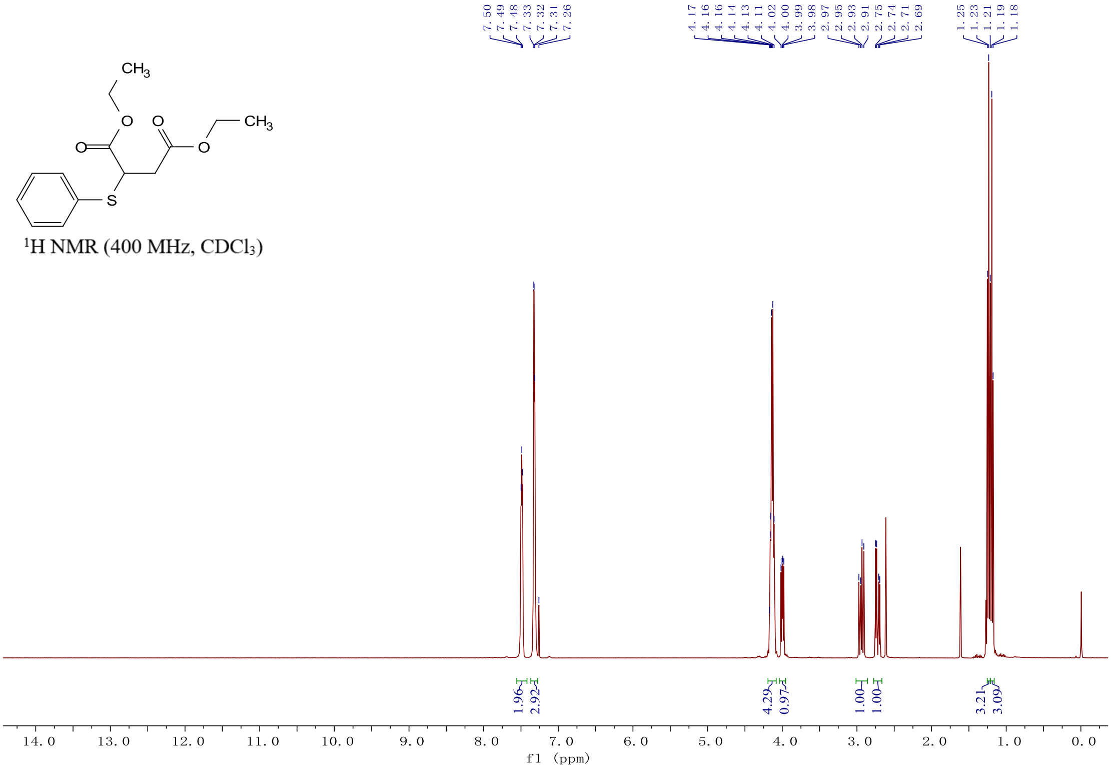

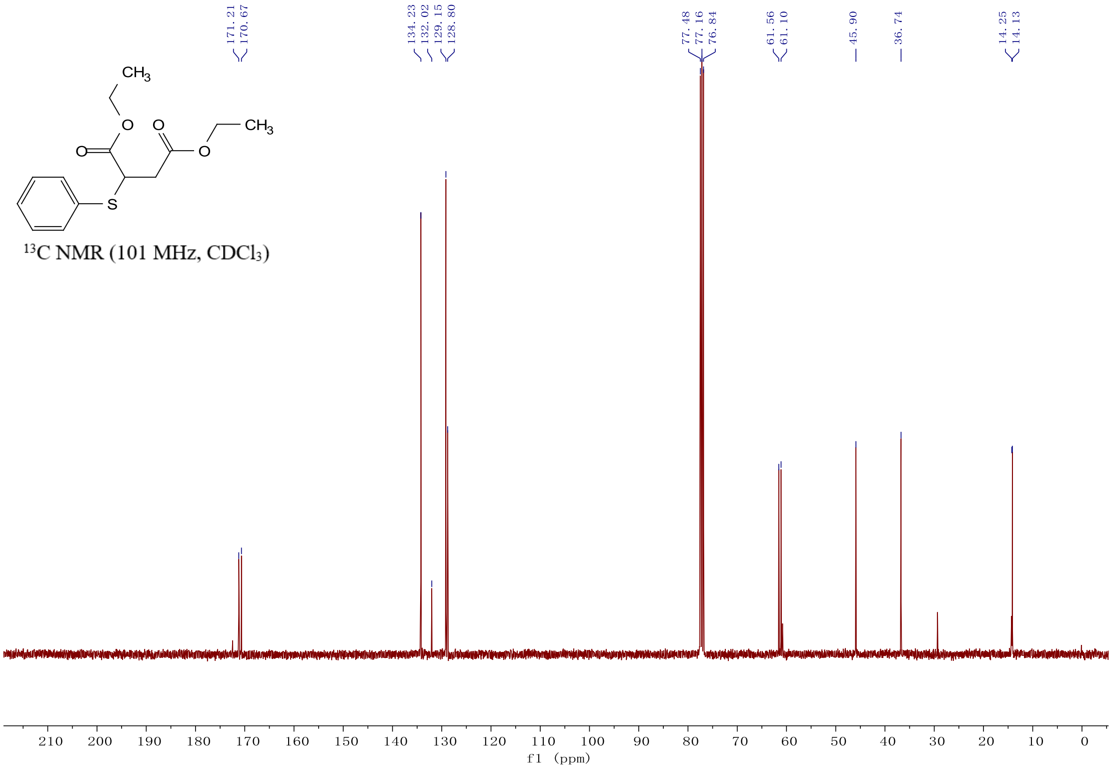

diisopropyl 2-(phenylthio)succinate (2av)

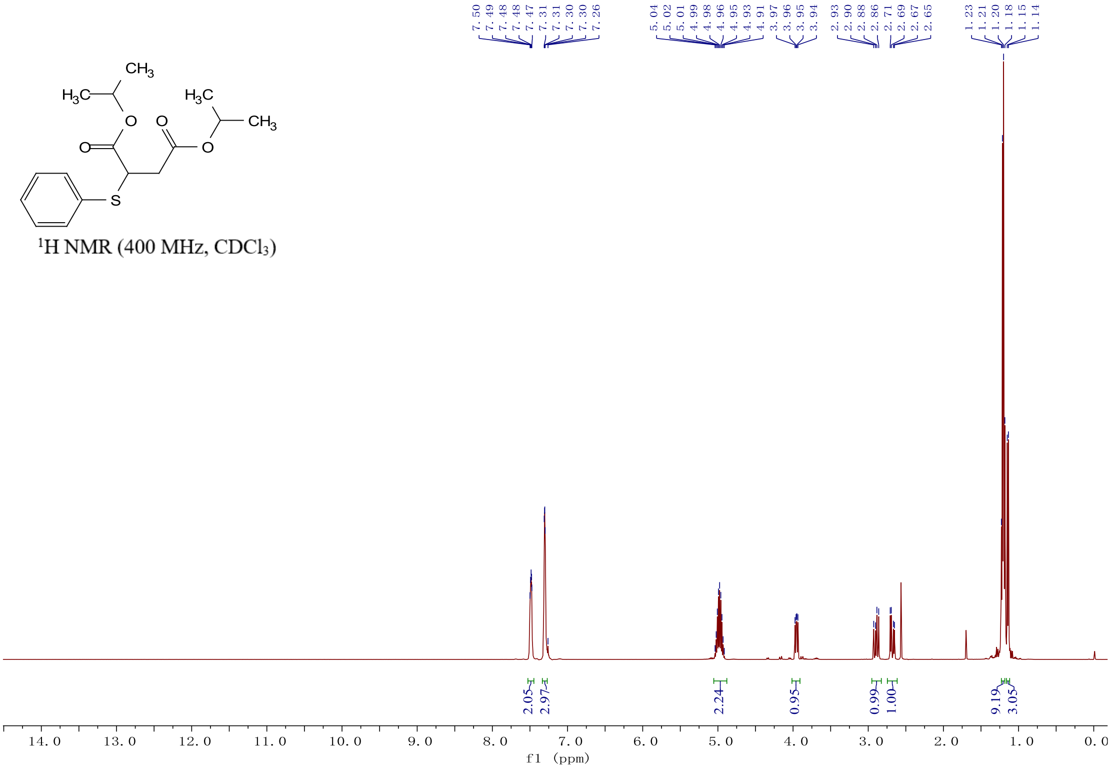

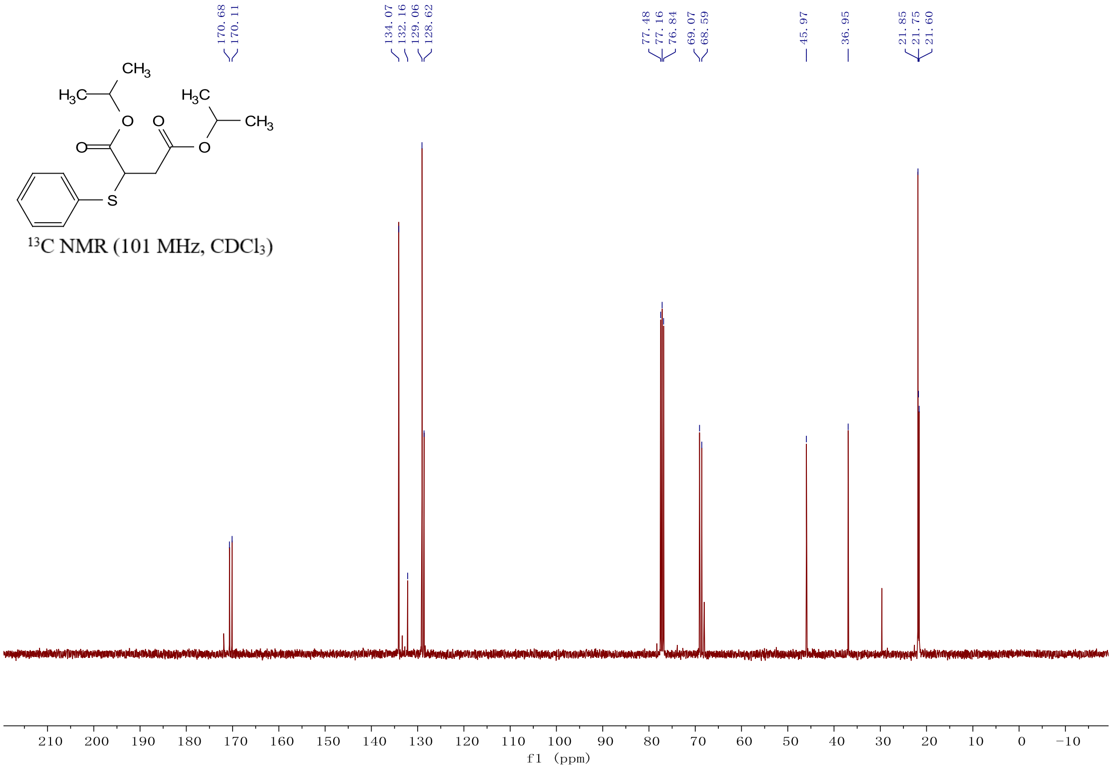

di-*tert*-butyl 2-(phenylthio)succinate (2aw)

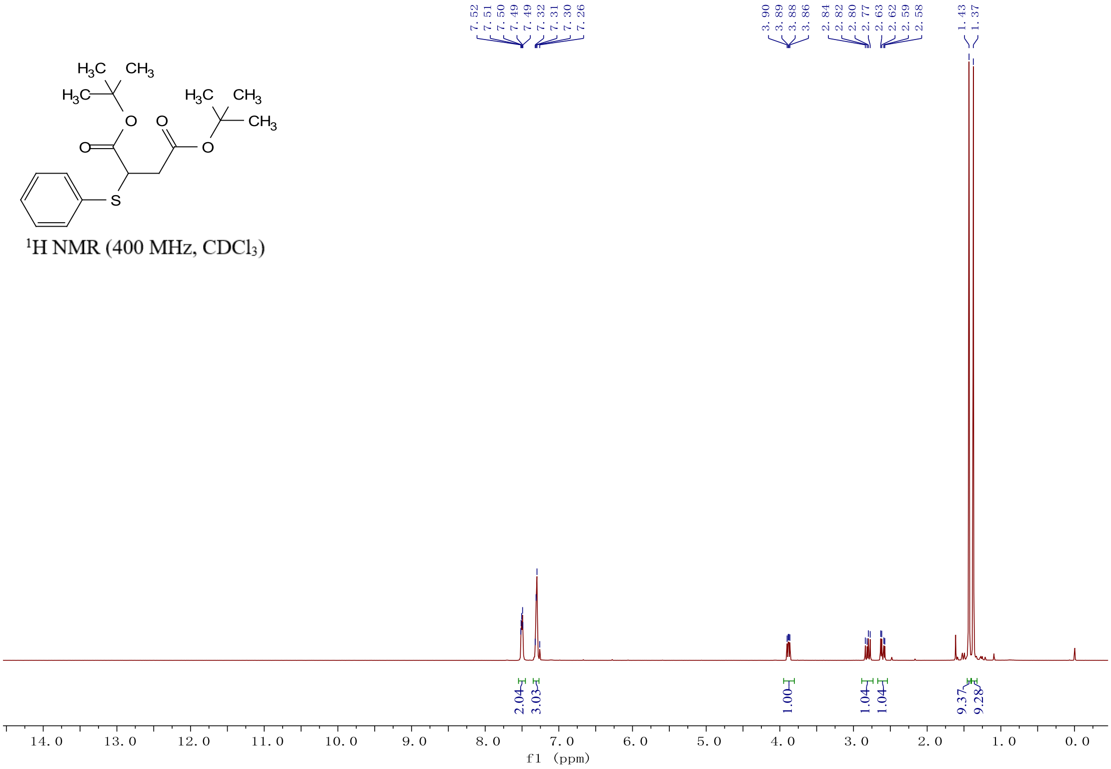

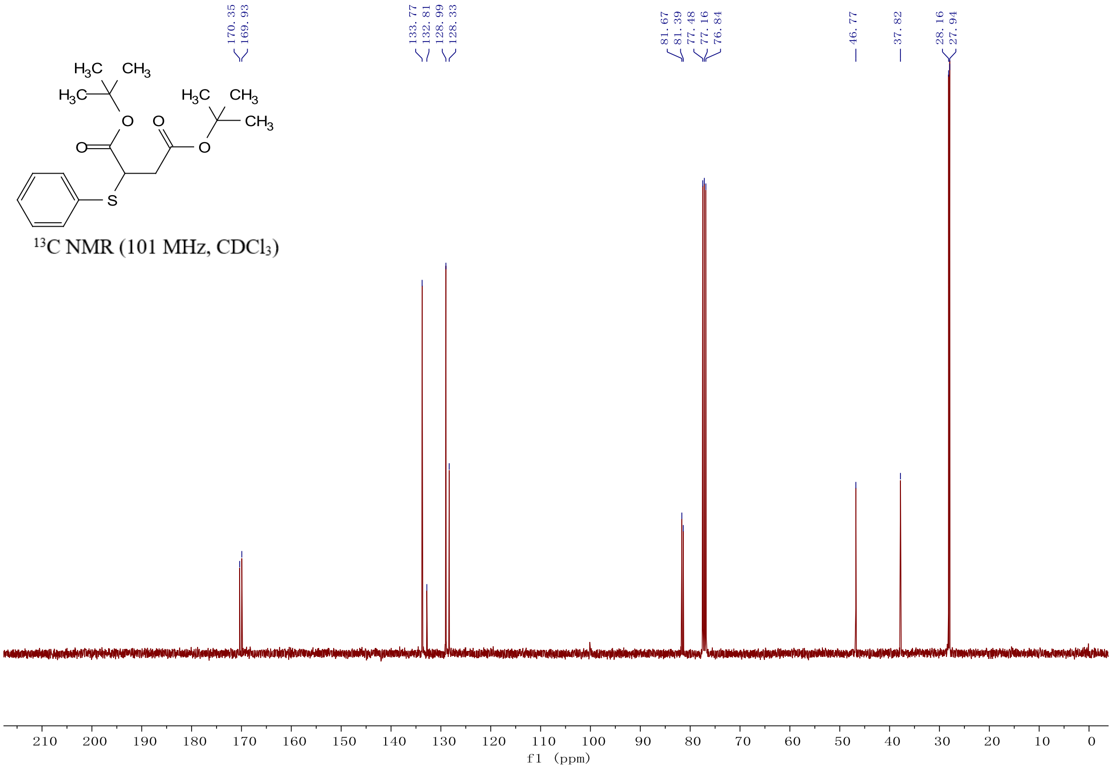

1-methyl-3-(phenylthio)pyrrolidine-2,5-dione (2ax)

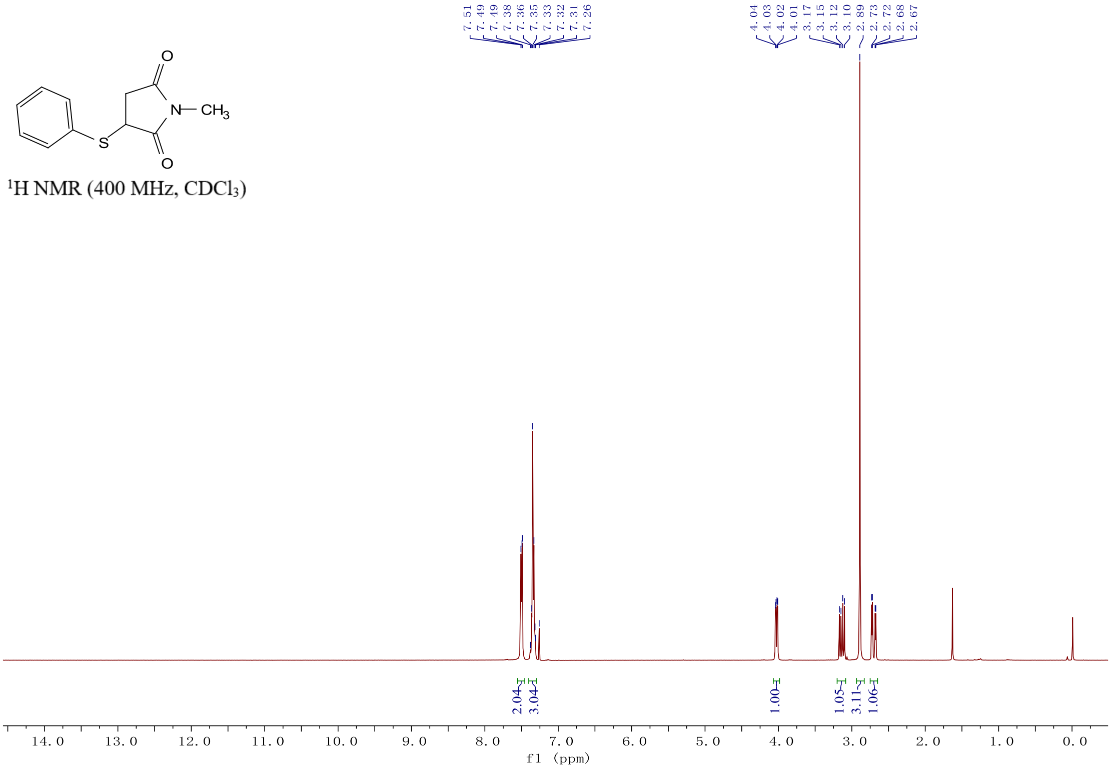

^{1}H NMR (400 MHz, $CDCl_3$)

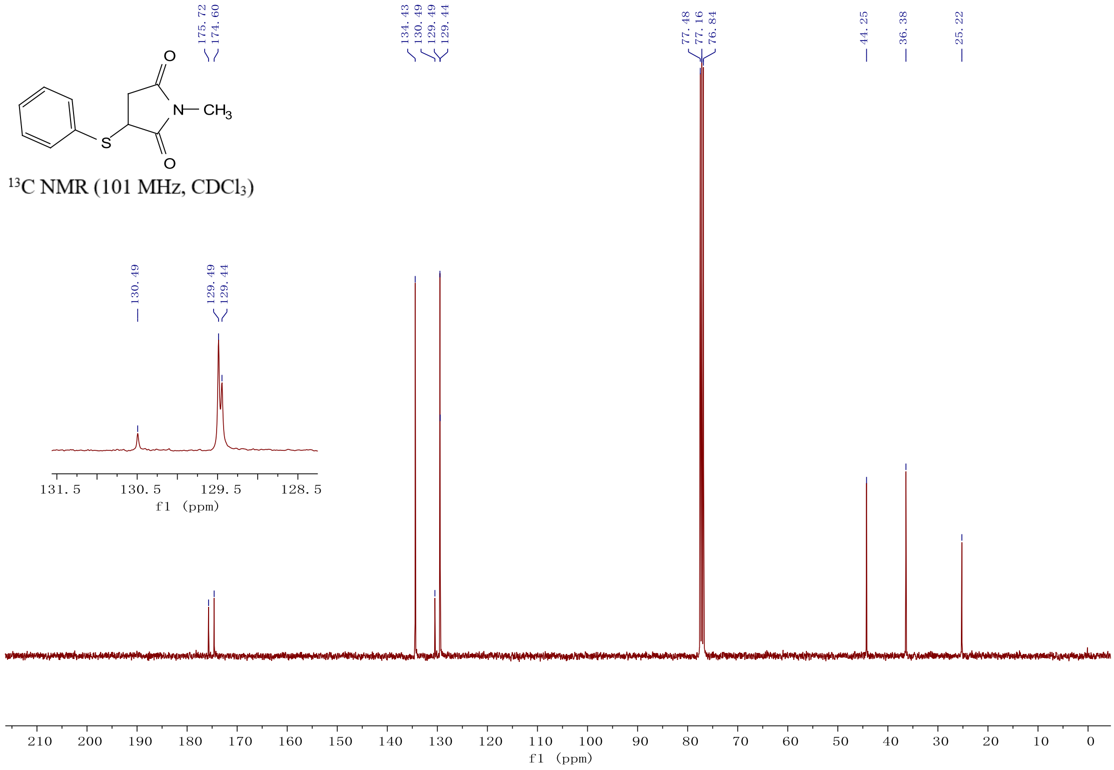

^{13}C NMR (101 MHz, $CDCl_3$)

dimethyl 2-(phenylsulfonyl)succinate (2ay)

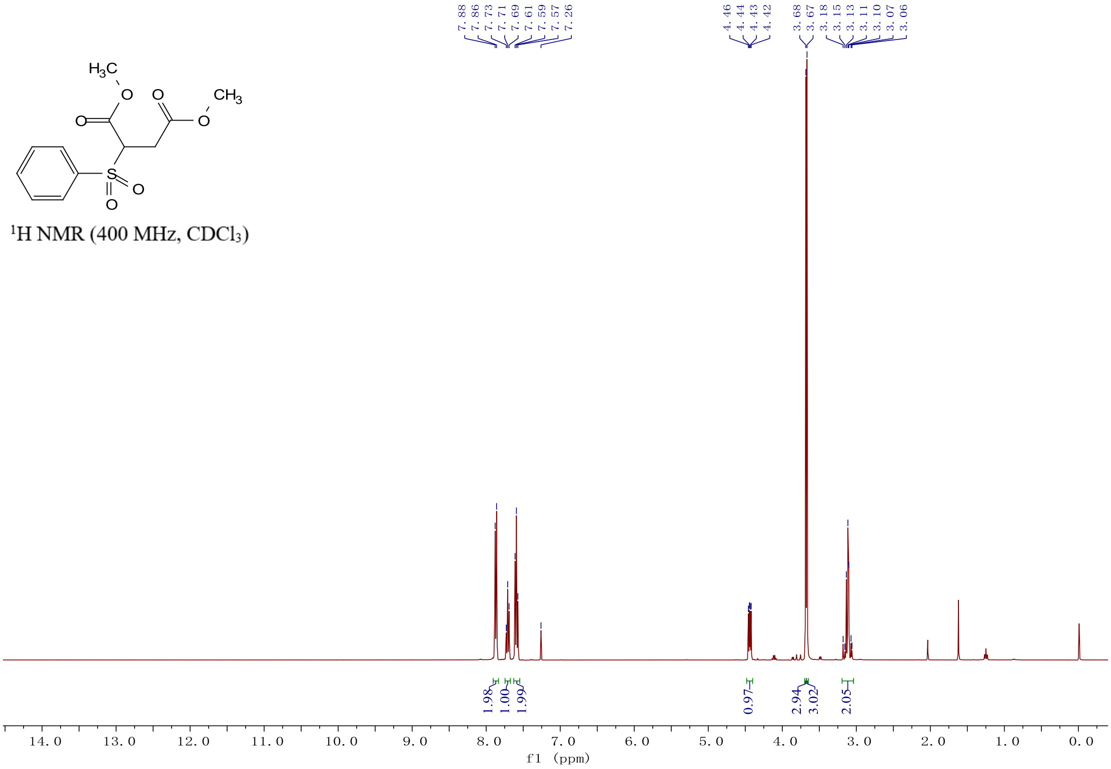

^{1}H NMR (400 MHz, $CDCl_3$)

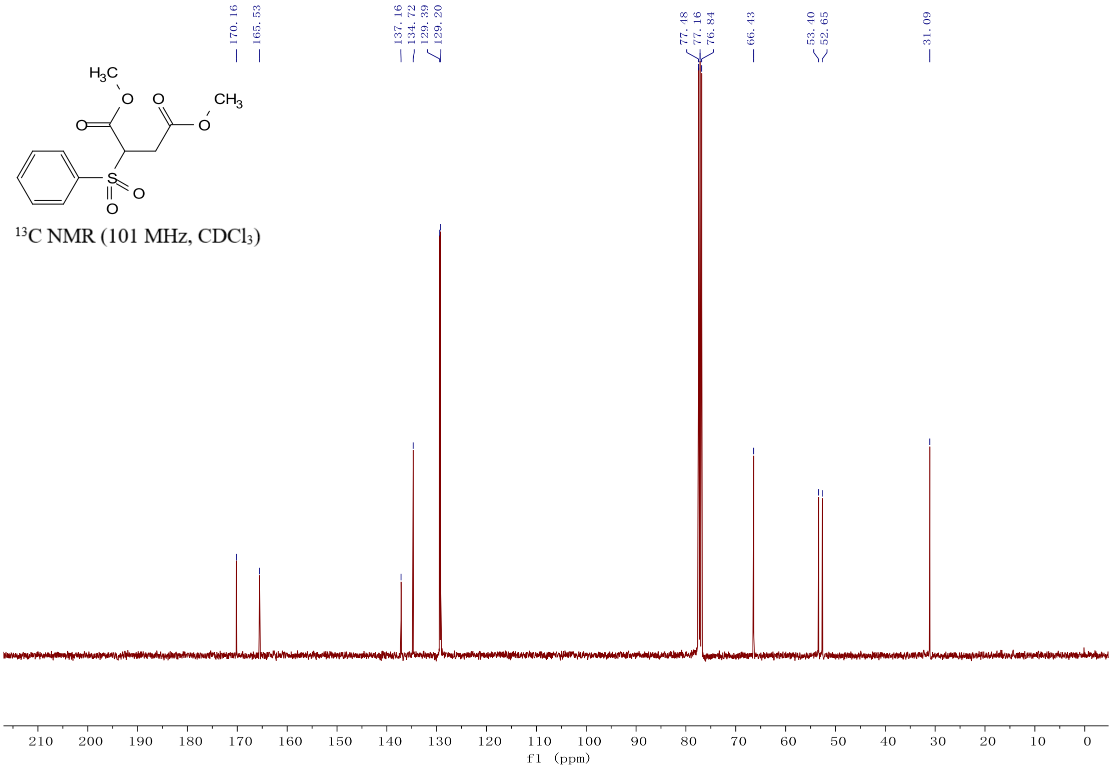

^{13}C NMR (101 MHz, $CDCl_3$)

dimethyl 2-tosylsuccinate (2az)

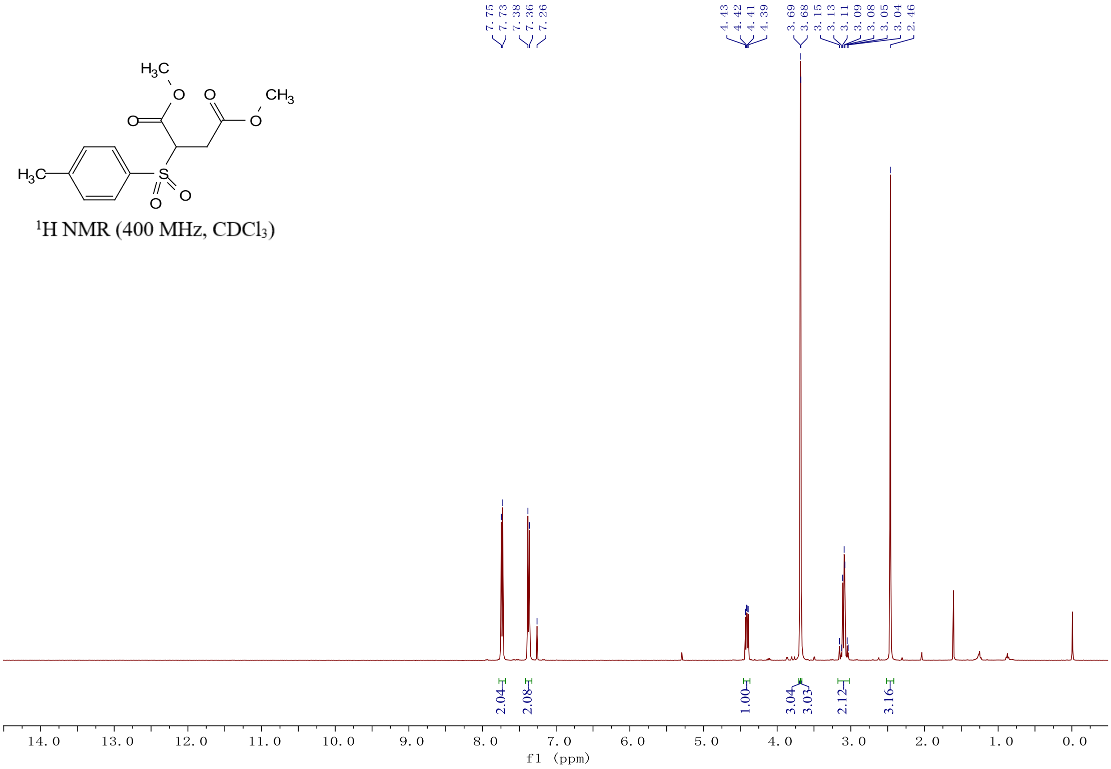

^{1}H NMR (400 MHz, CDCl$_3$)

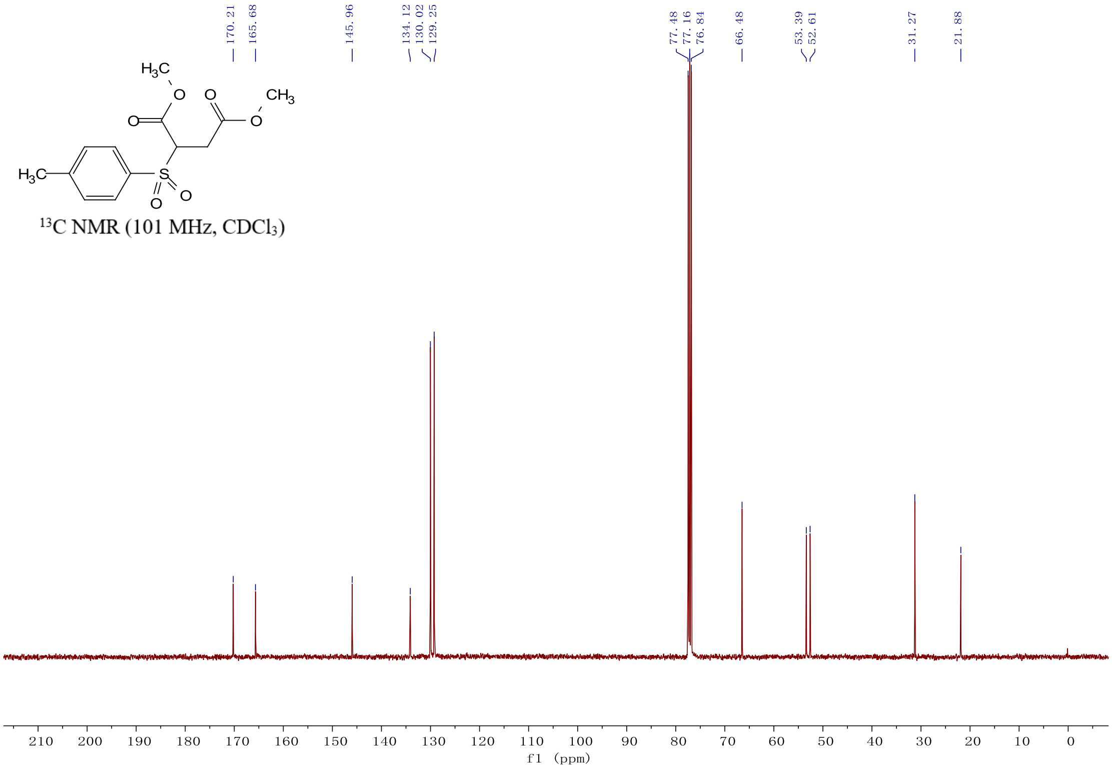

^{13}C NMR (101 MHz, CDCl$_3$)

dimethyl phenylaspartate (2ba)

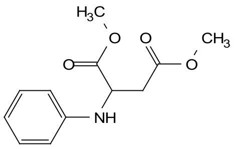

^{1}H NMR (400 MHz, DMSO-d_6)

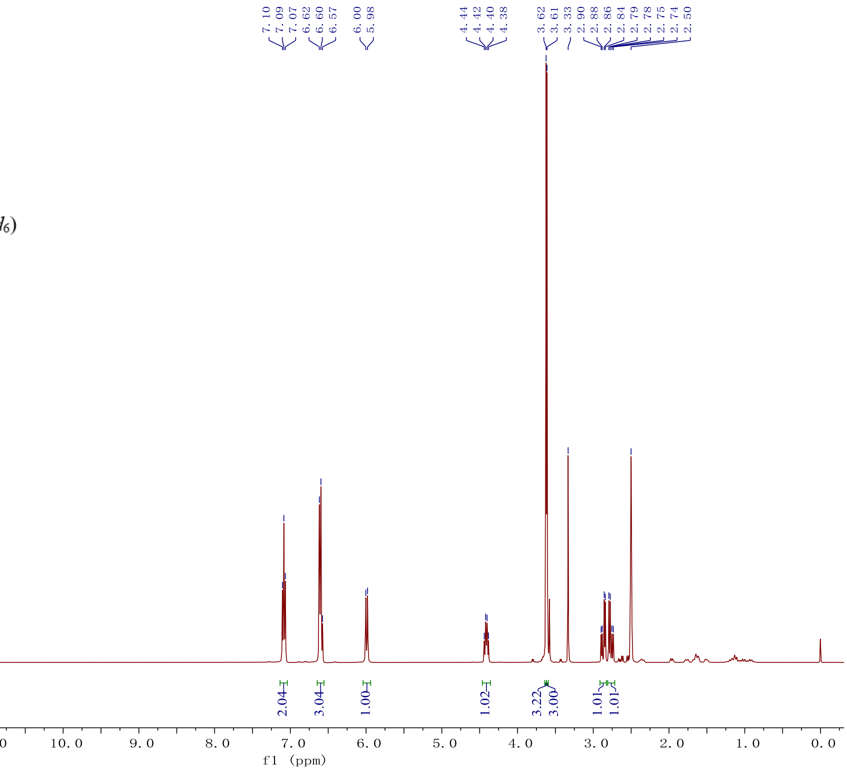

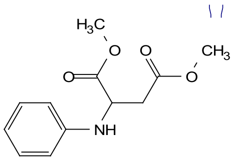

^{13}C NMR (101 MHz, DMSO-d_6)

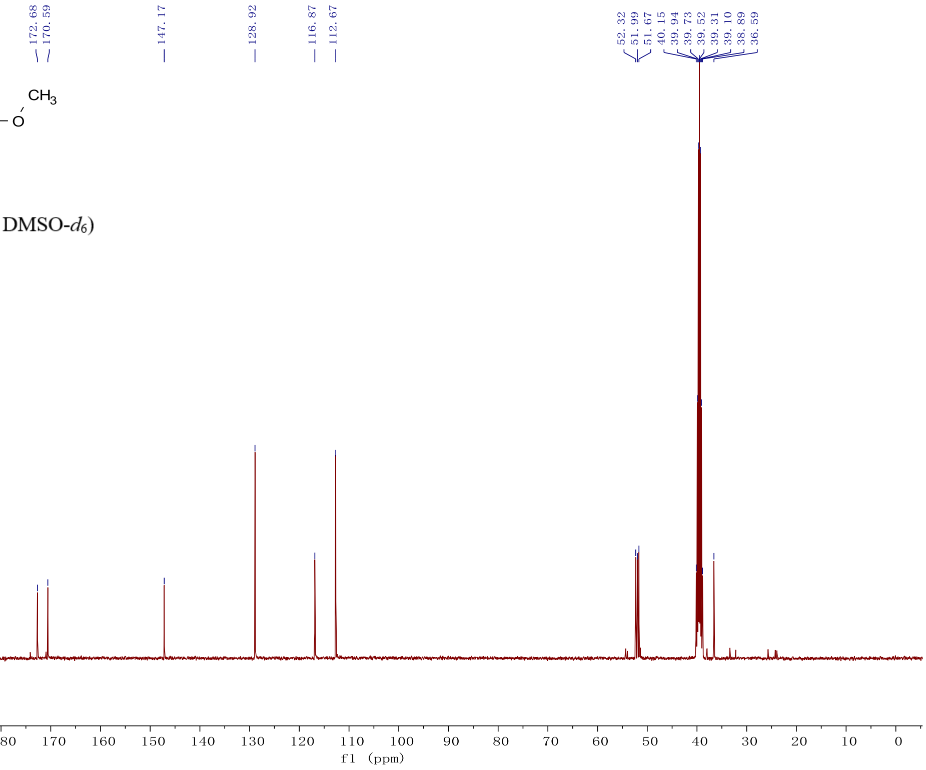

dimethyl 2-phenoxysuccinate (2bb)

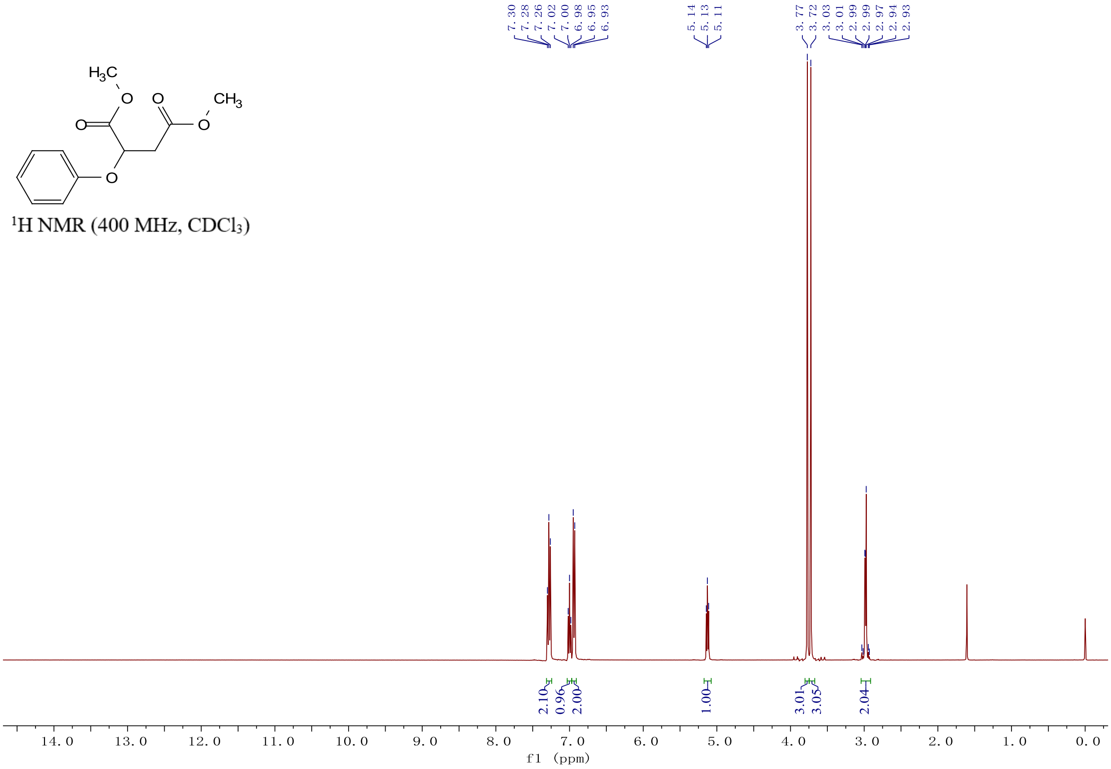

^{1}H NMR (400 MHz, $CDCl_3$)

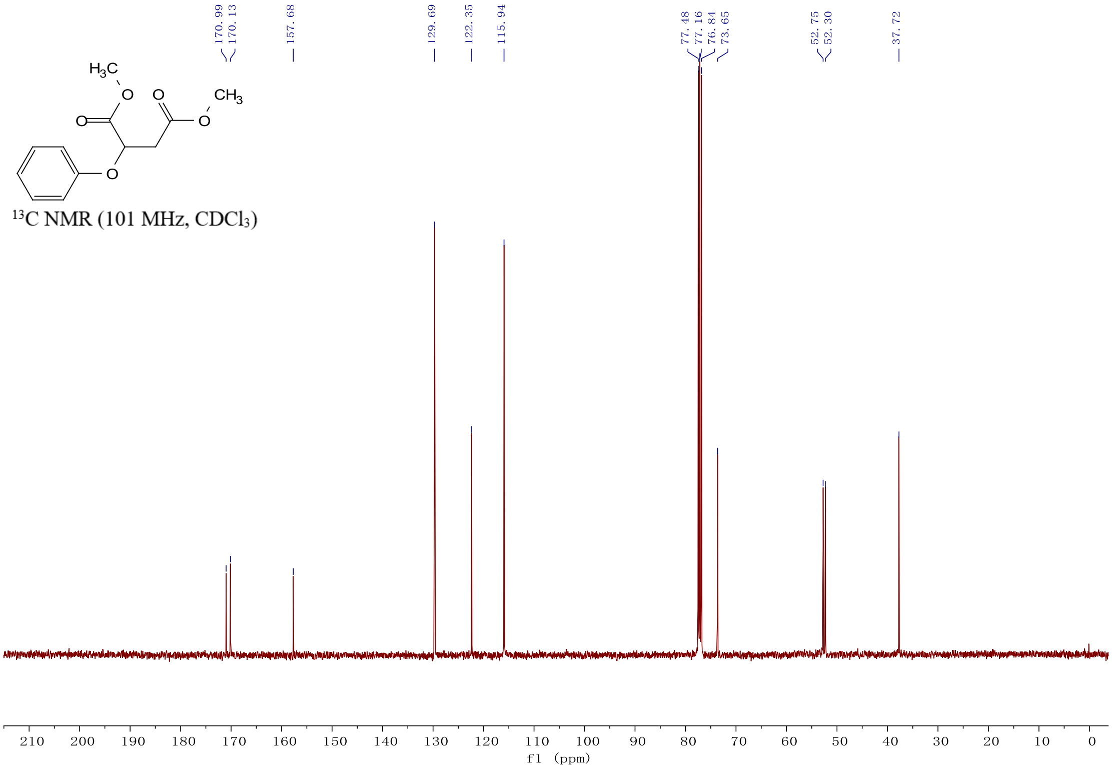

^{13}C NMR (101 MHz, $CDCl_3$)

dimethyl 2-(4-methoxyphenoxy)succinate (2bc)

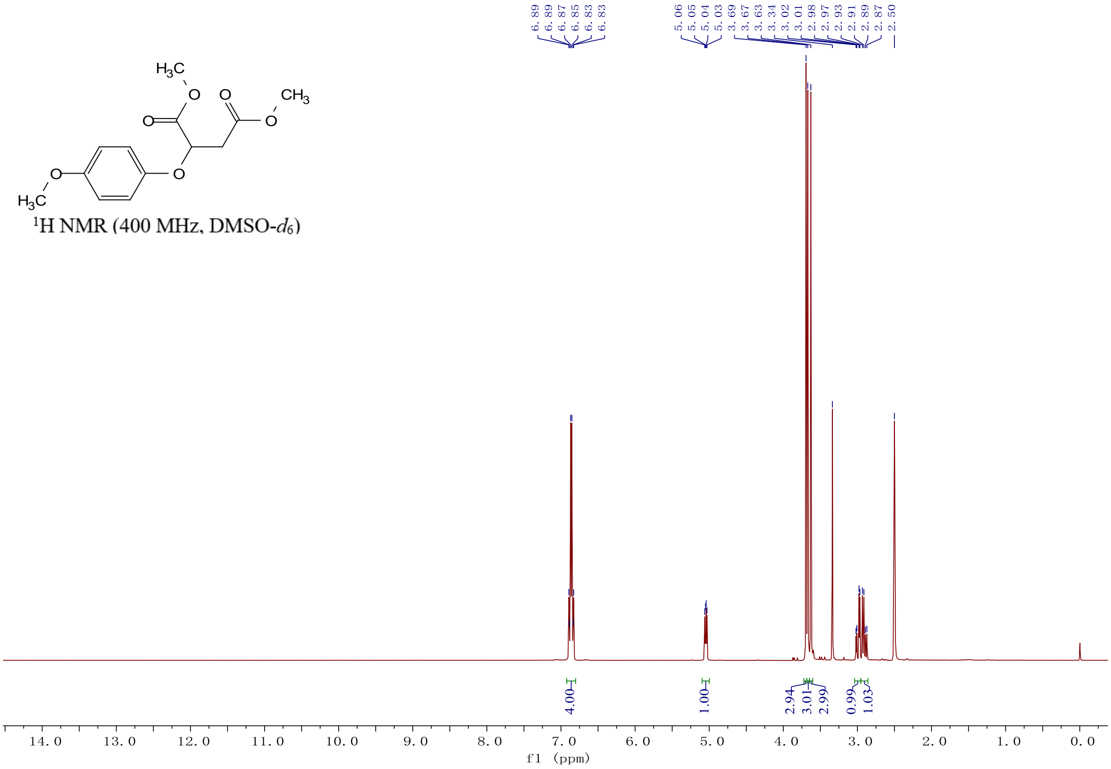

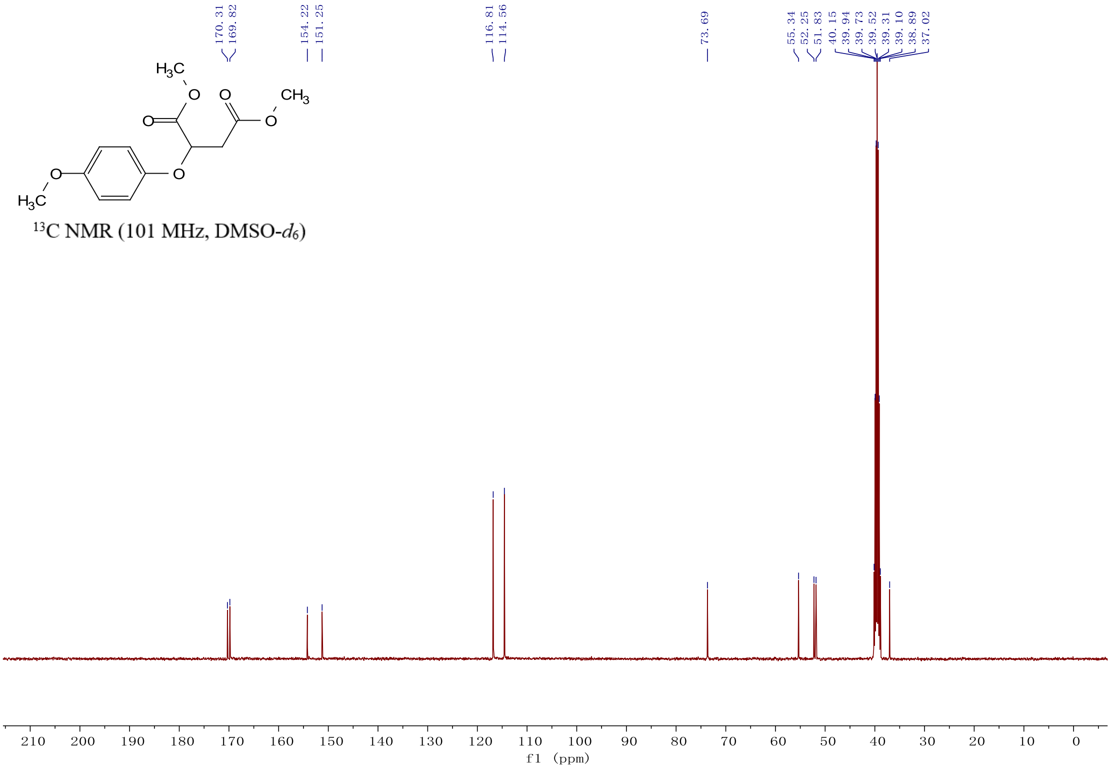

dimethyl 2-(phenylselanyl)succinate (2bd)

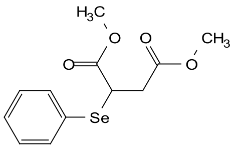

^{1}H NMR (400 MHz, $CDCl_3$)

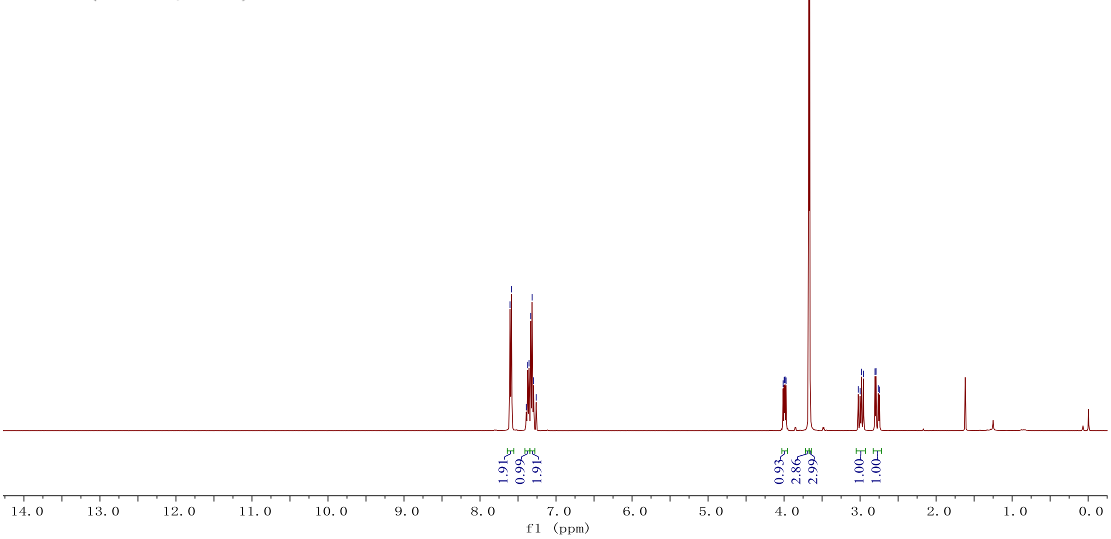

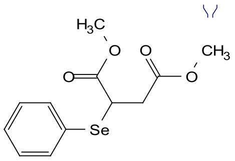

^{13}C NMR (101 MHz, $CDCl_3$)

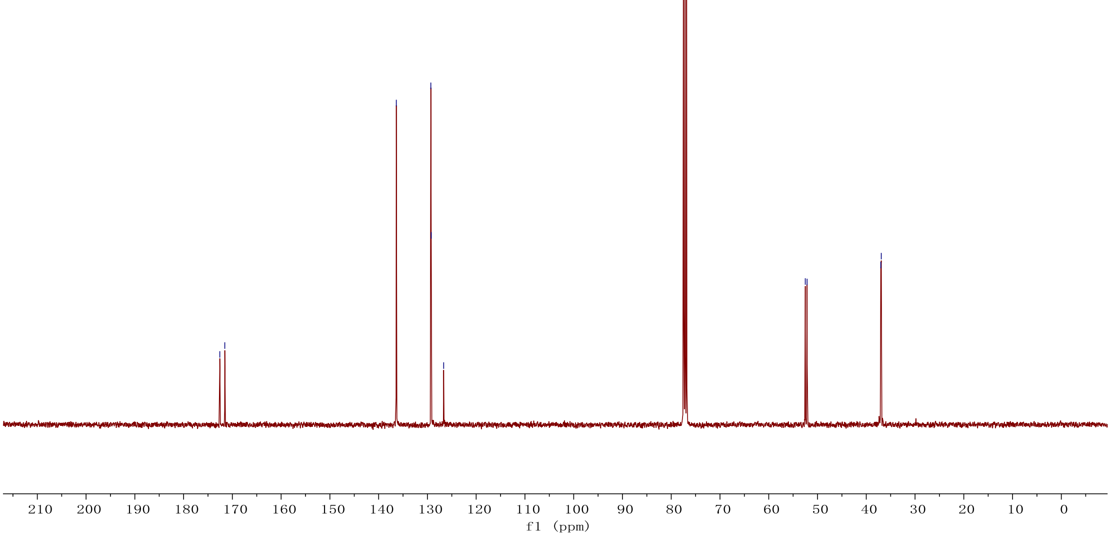

(*Z*)-3-(phenylthio)hex-3-en-2-ol (*Z*-9aa)

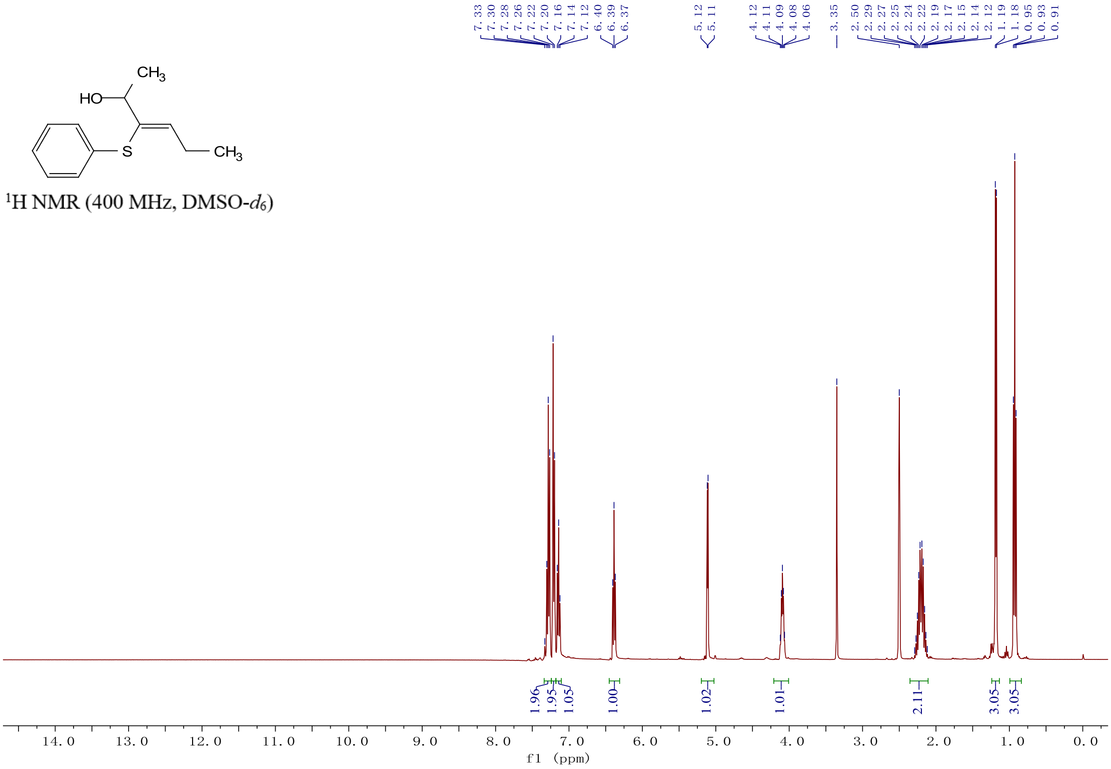

^{1}H NMR (400 MHz, DMSO-*d*$_6$)

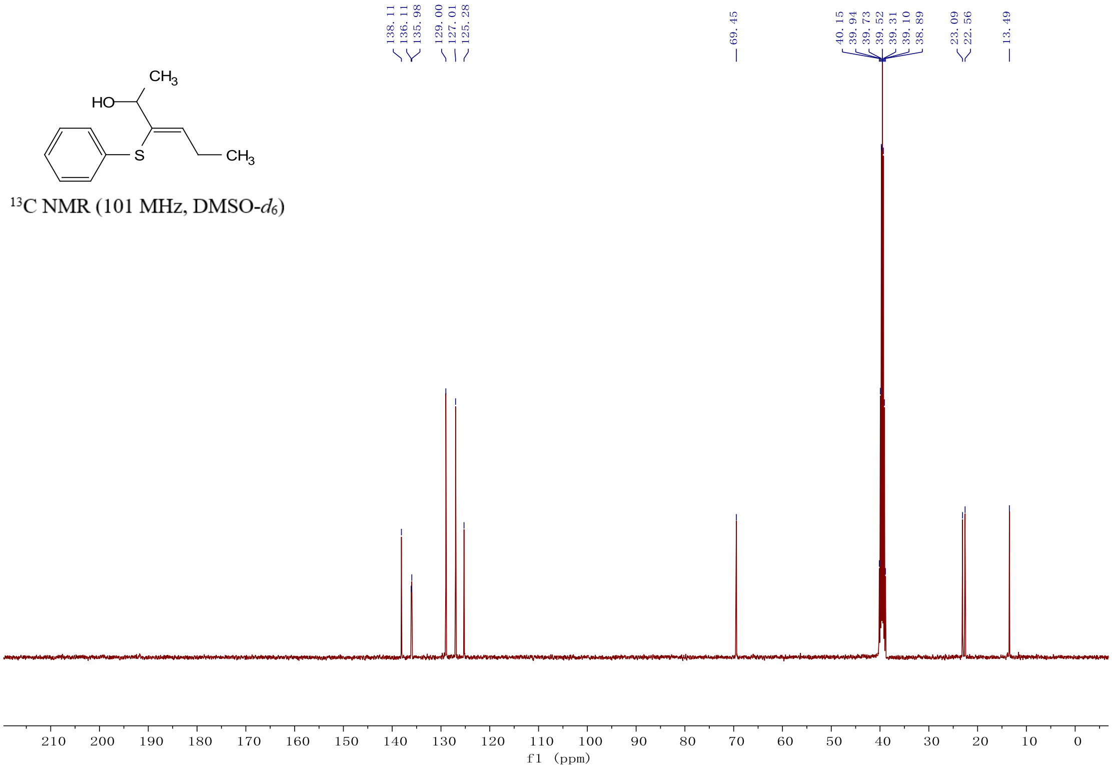

^{13}C NMR (101 MHz, DMSO-*d*$_6$)

(*Z*)-3-(phenylthio)hex-3-en-2-ol (*Z*-9aa)

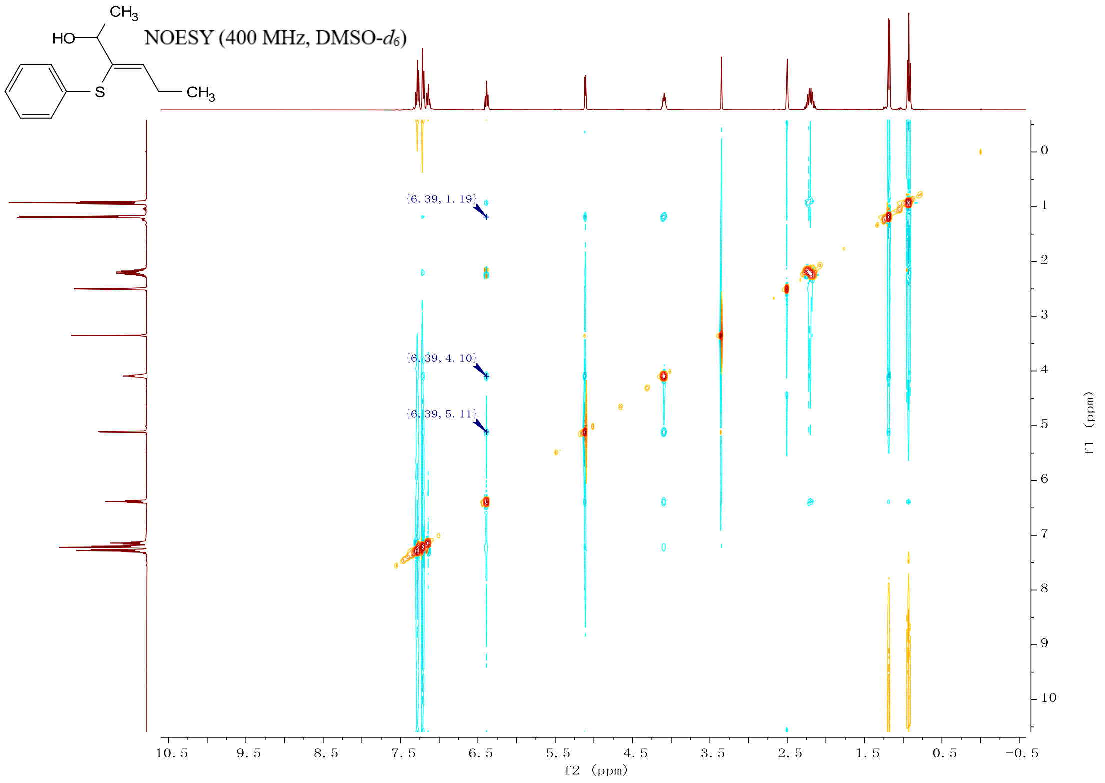

(*Z*)-3-((2-fluorophenyl)thio)hex-3-en-2-ol (*Z*-9ab)

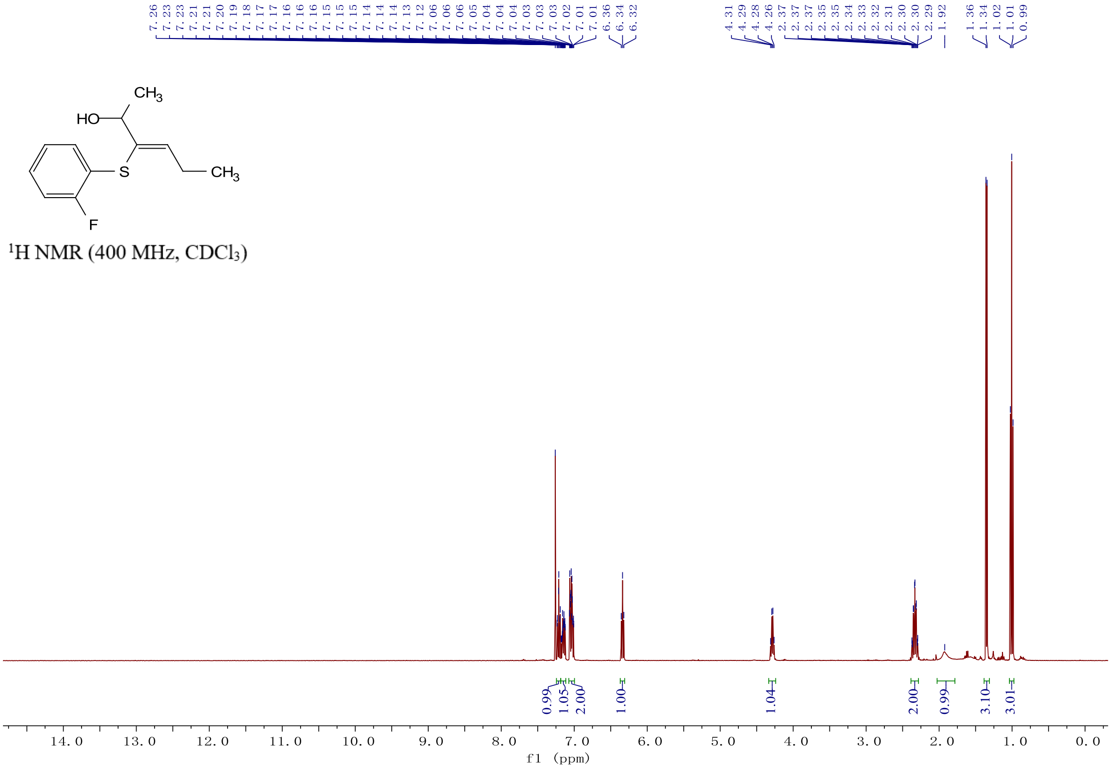

^{1}H NMR (400 MHz, $CDCl_3$)

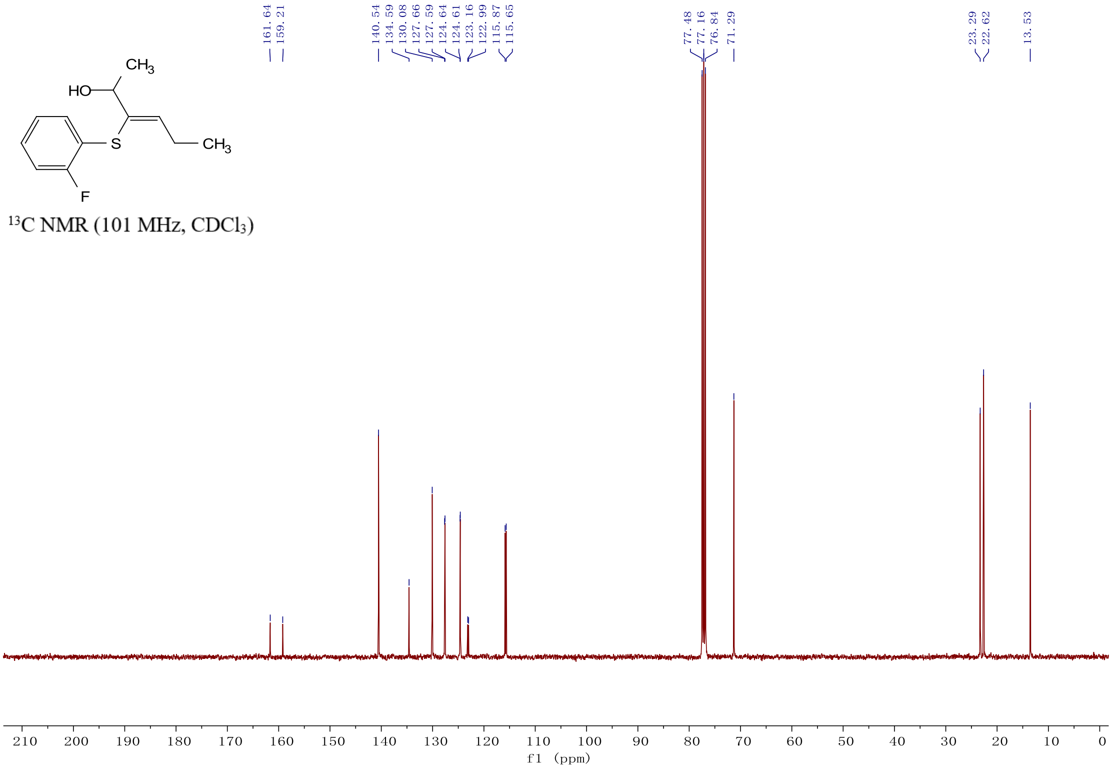

^{13}C NMR (101 MHz, $CDCl_3$)

(*Z*)-3-((2-fluorophenyl)thio)hex-3-en-2-ol (*Z*-9ab)

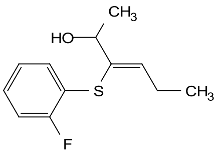

^{19}F NMR (376 MHz, CDCl$_3$)

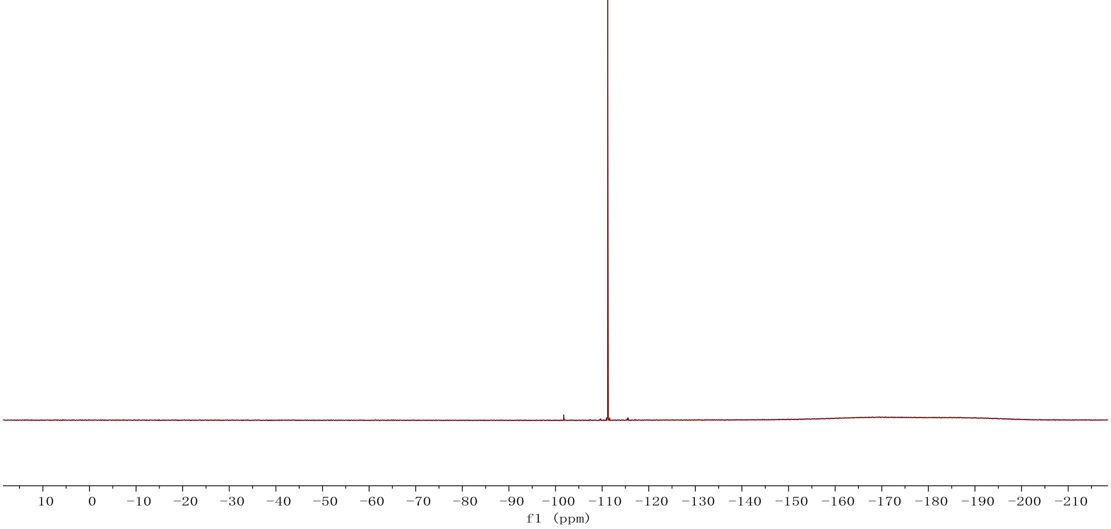

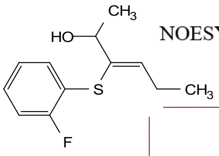

NOESY (400 MHz, CDCl$_3$)

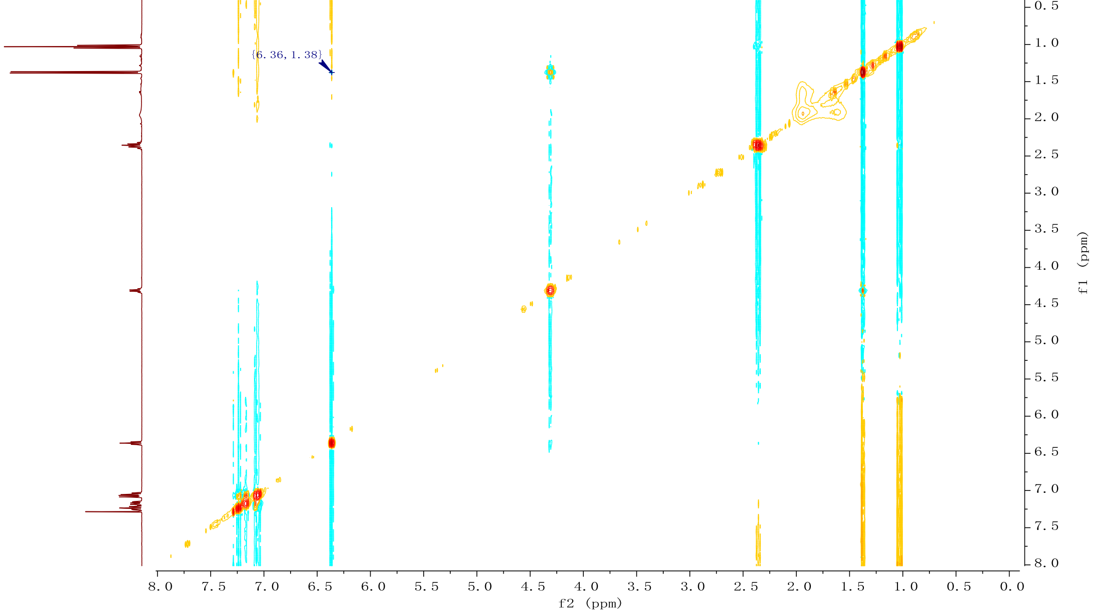

(Z)-3-((2-chlorophenyl)thio)hex-3-en-2-ol (*Z*-9ac)

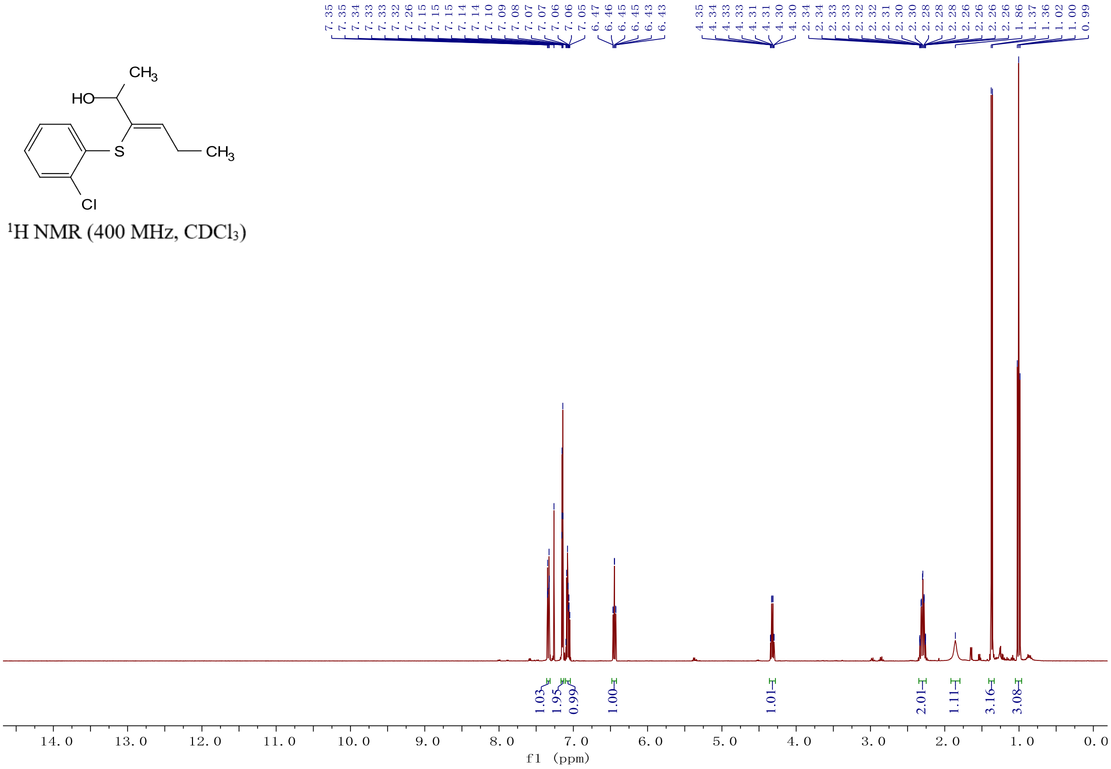

^{1}H NMR (400 MHz, CDCl$_3$)

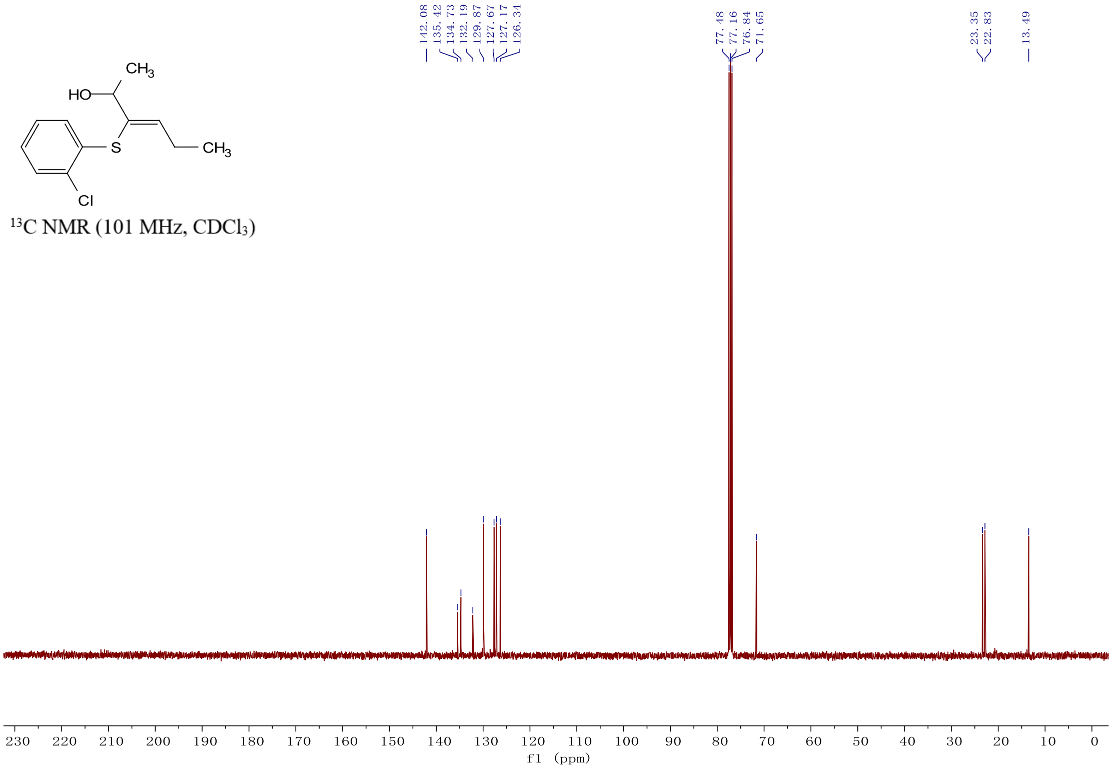

^{13}C NMR (101 MHz, CDCl$_3$)

(*Z*)-3-((2-chlorophenyl)thio)hex-3-en-2-ol (*Z*-9ac)

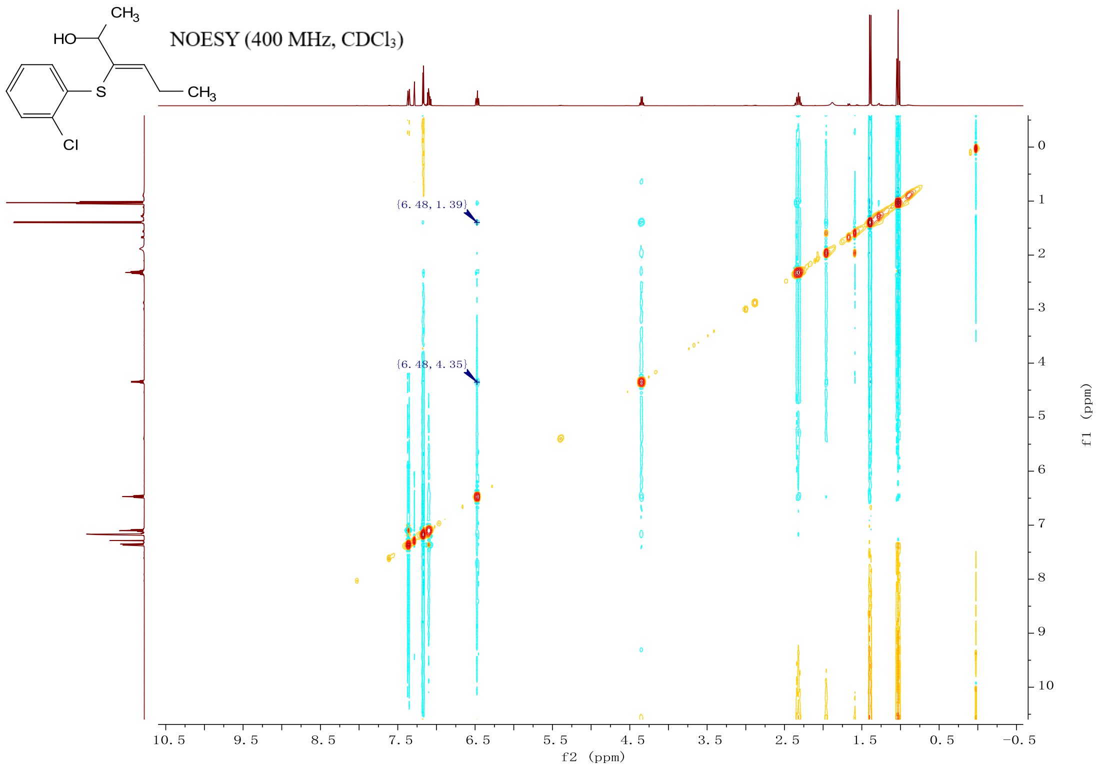

(*Z*)-3-((4-chlorophenyl)thio)hex-3-en-2-ol (*Z*-9ad)

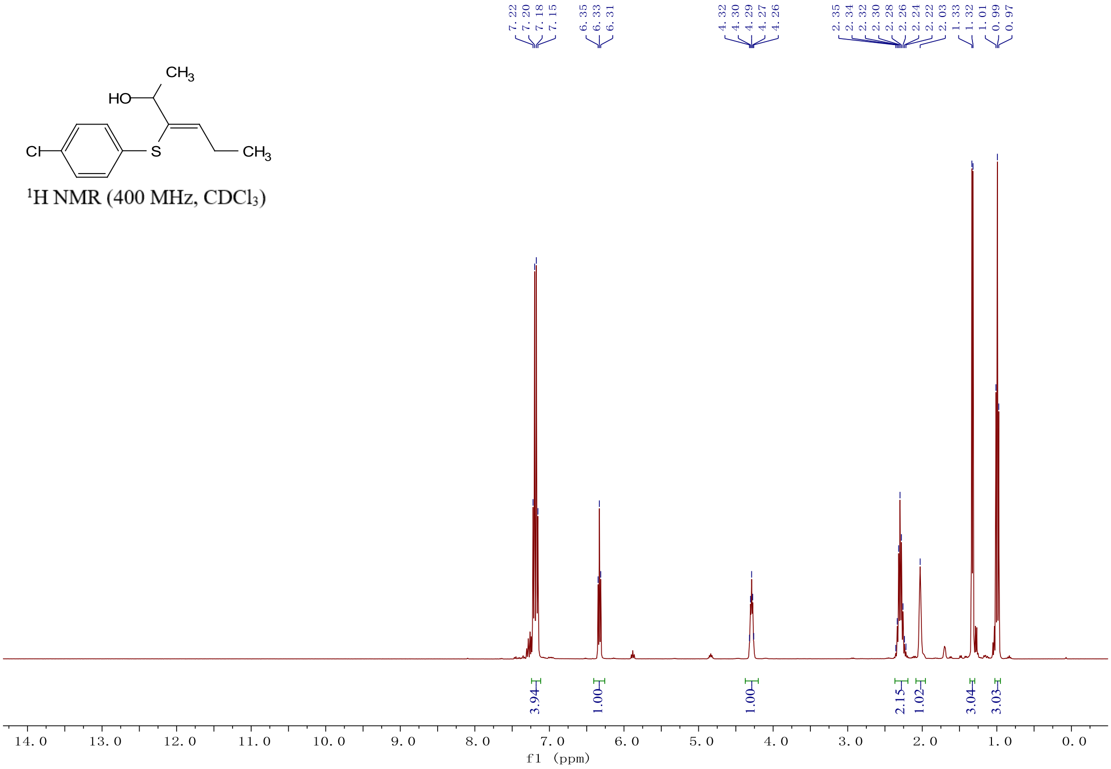

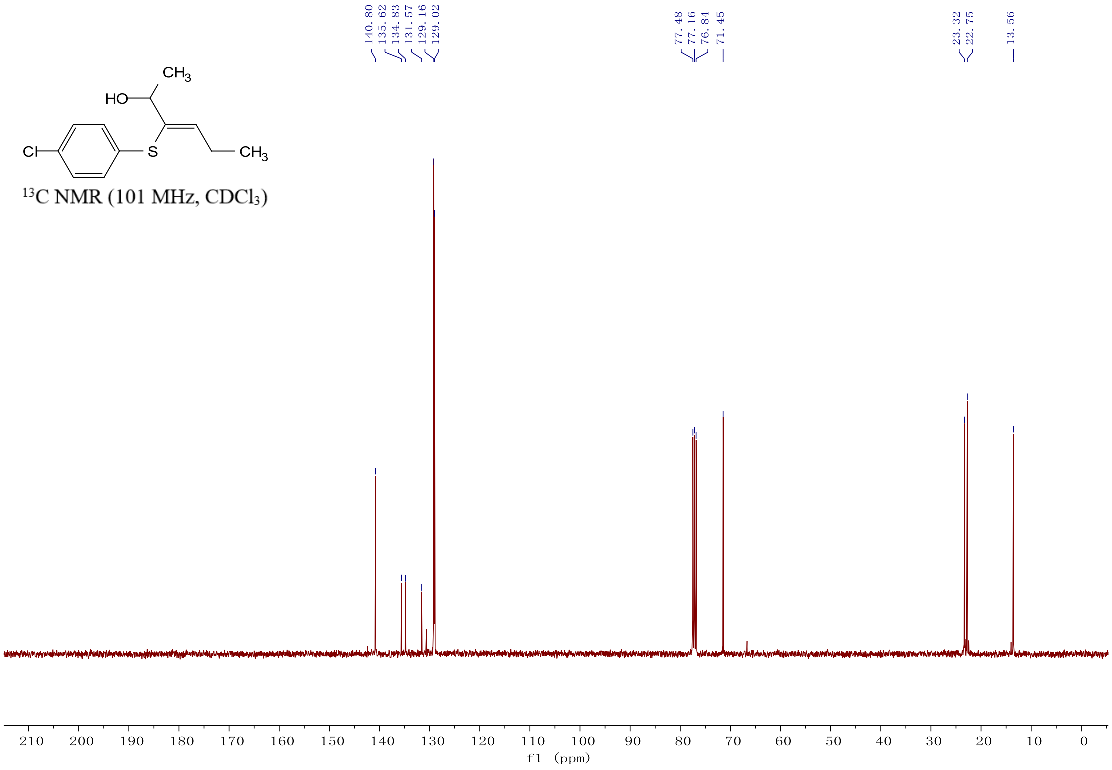

(*Z*)-3-((4-chlorophenyl)thio)hex-3-en-2-ol (*Z*-9ad)

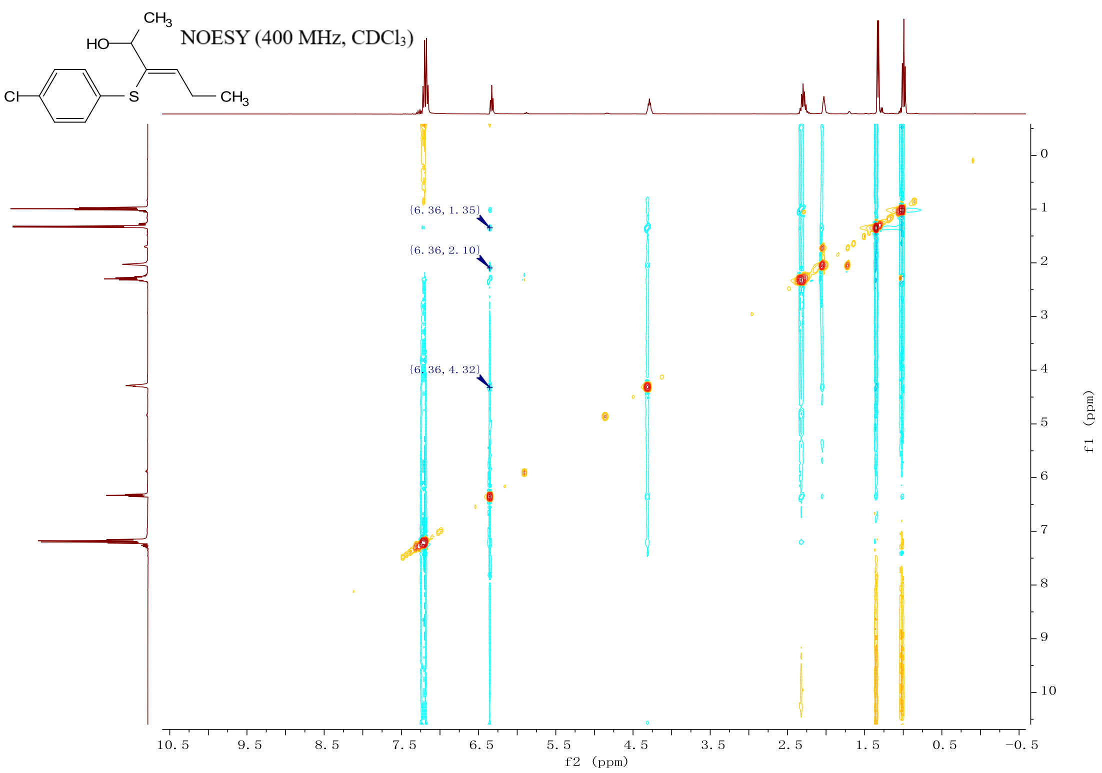

(*Z*)-3-((2-bromophenyl)thio)hex-3-en-2-ol (*Z*-9ae)

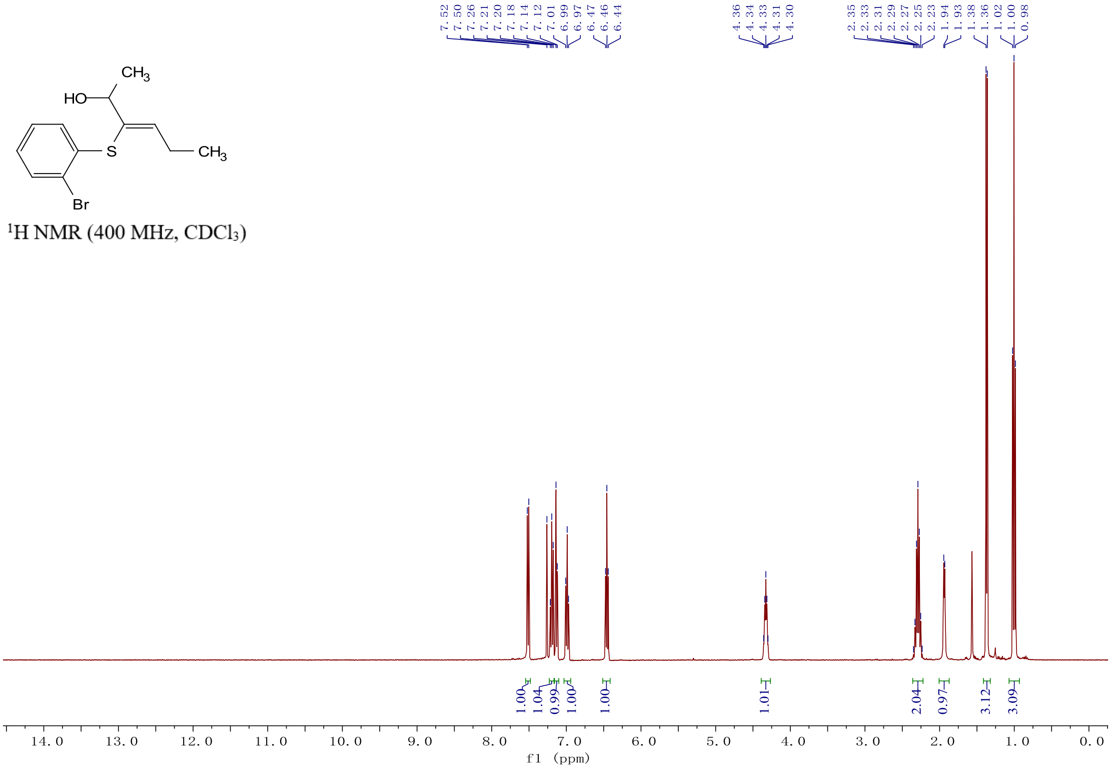

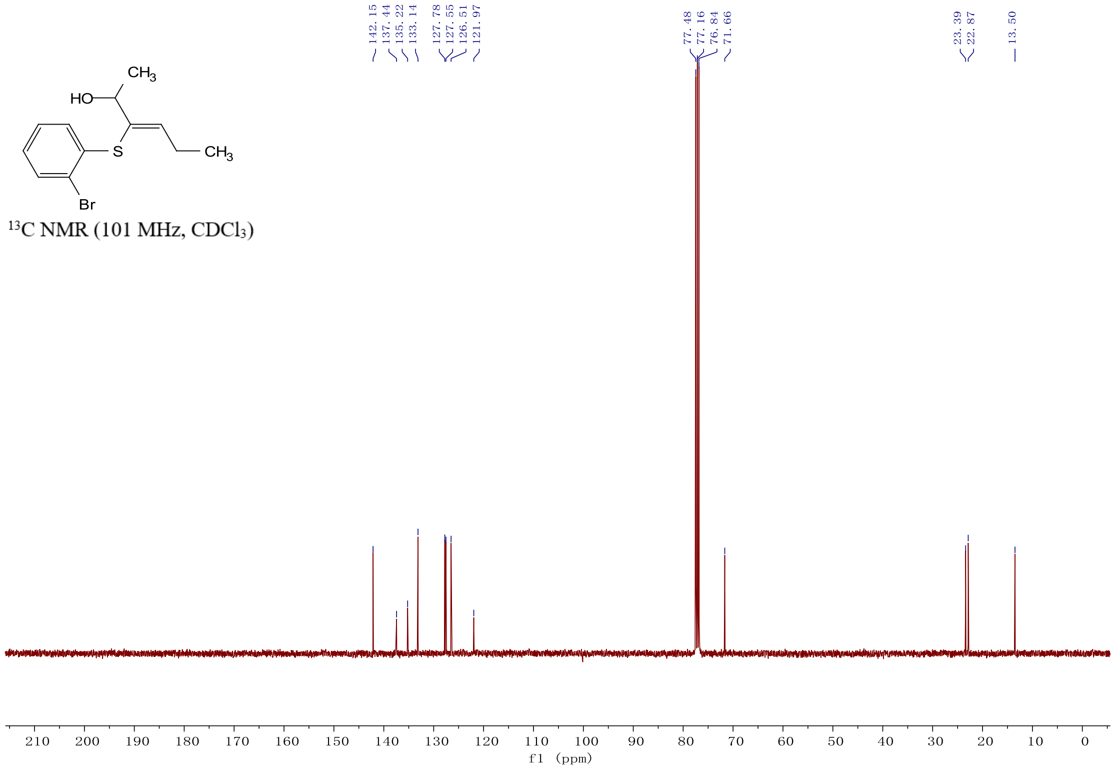

(*Z*)-3-((2-bromophenyl)thio)hex-3-en-2-ol (*Z*-9ae)

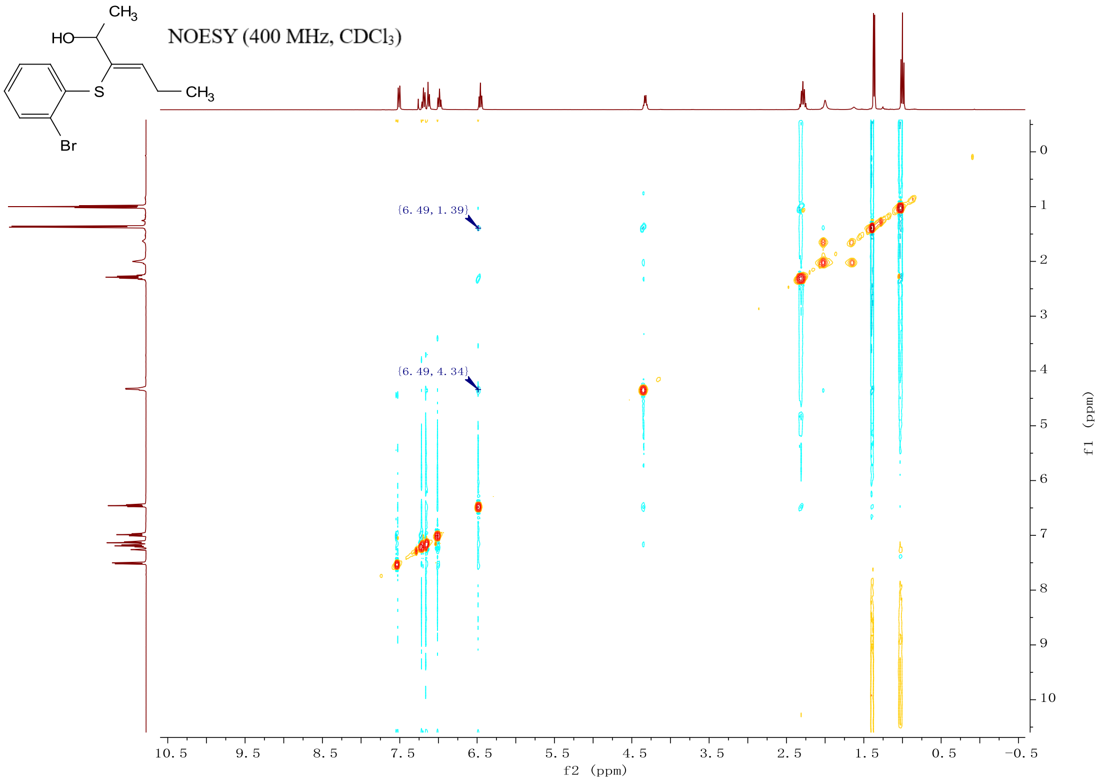

(*Z*)-3-((4-bromophenyl)thio)hex-3-en-2-ol (*Z*-9af)

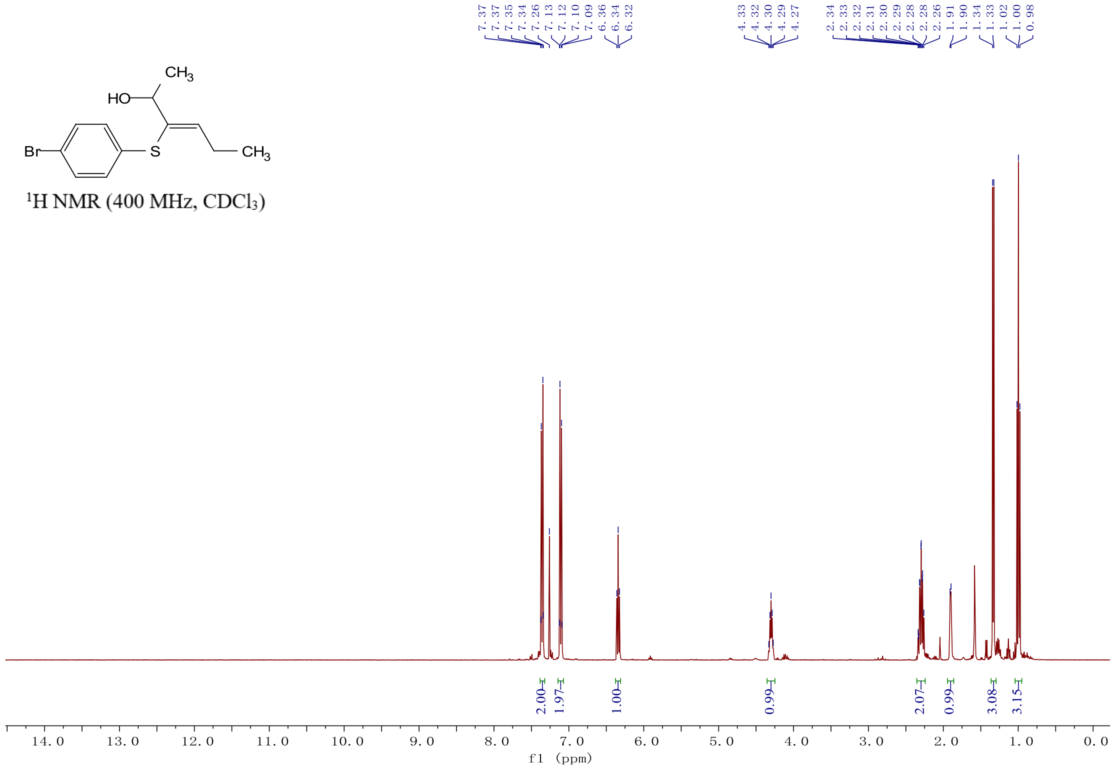

^{1}H NMR (400 MHz, $CDCl_3$)

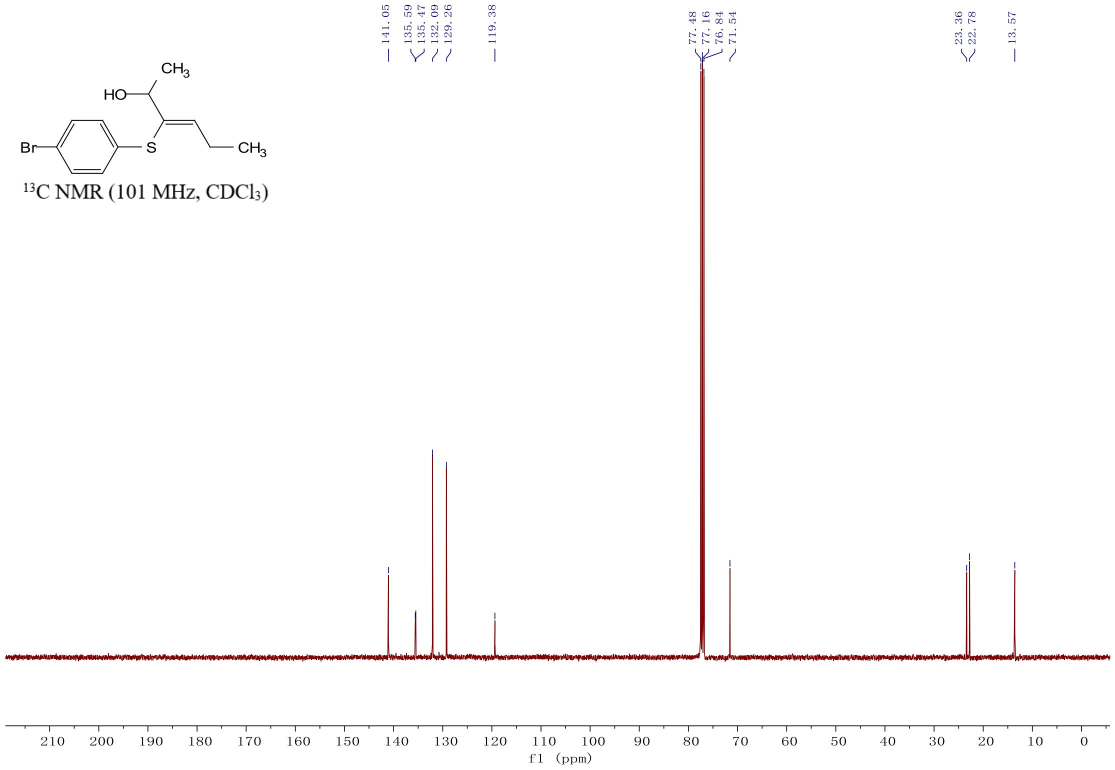

^{13}C NMR (101 MHz, $CDCl_3$)

(*Z*)-3-((4-bromophenyl)thio)hex-3-en-2-ol (*Z*-9af)

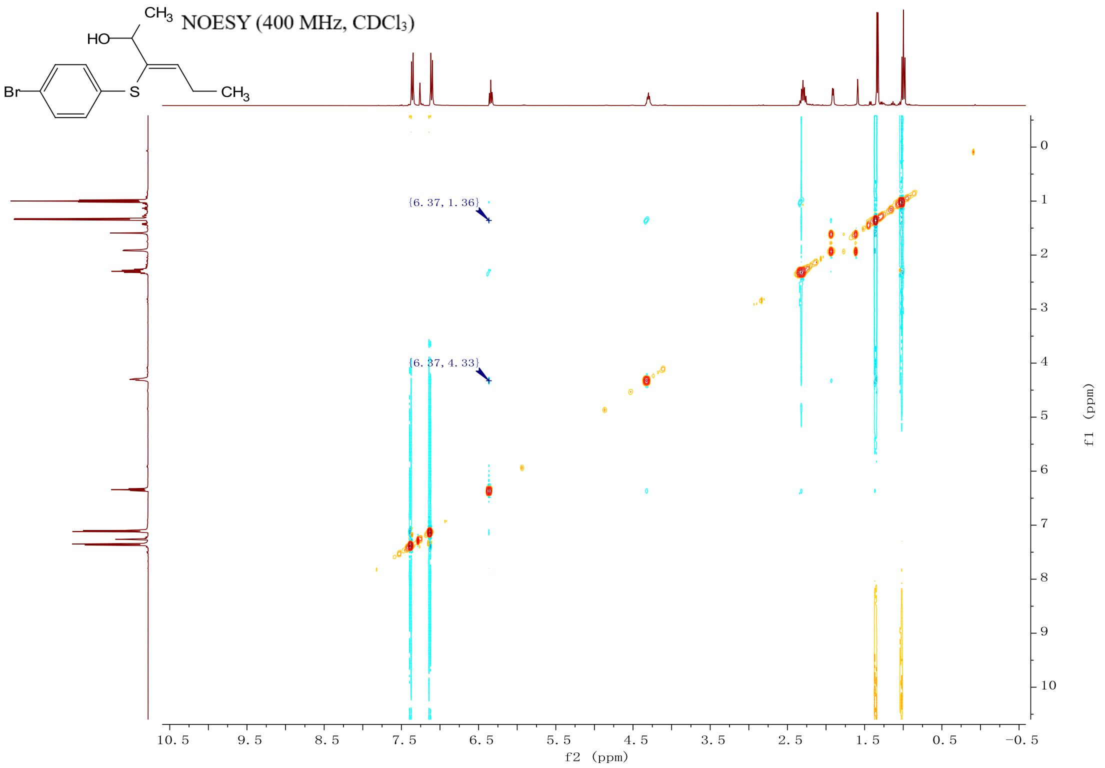

(*Z*)-4-phenyl-3-(phenylthio)but-3-en-2-ol (*Z*-9ag)

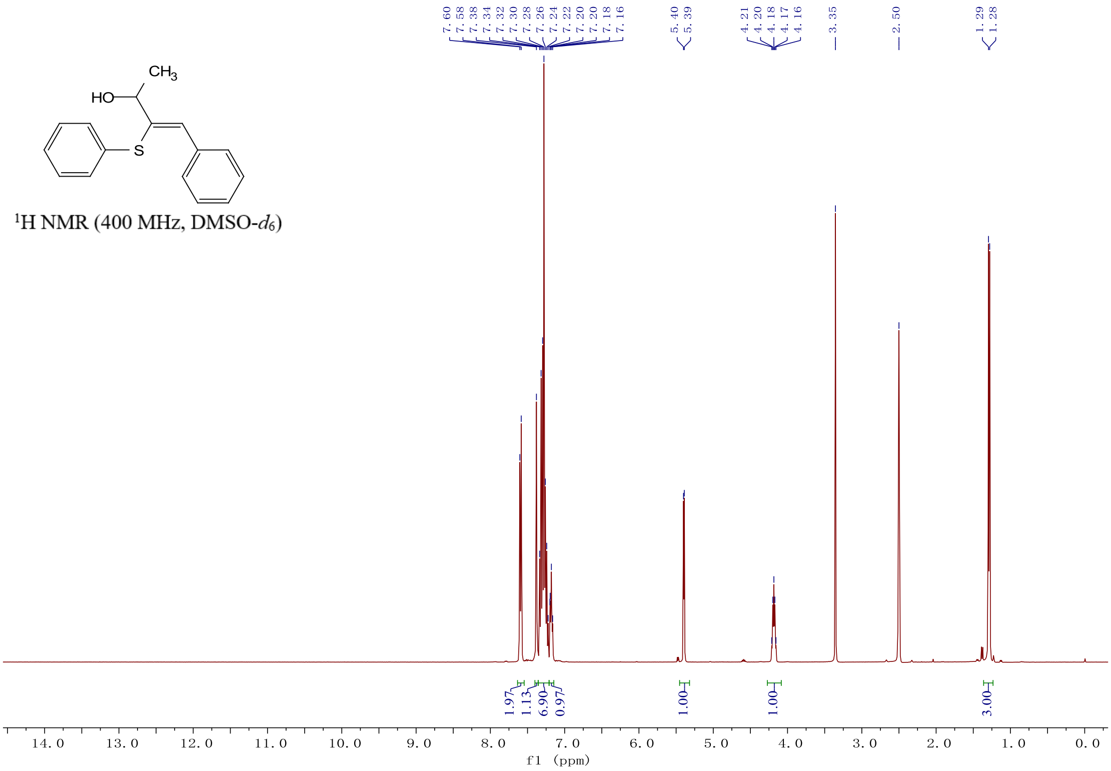

^{1}H NMR (400 MHz, DMSO-d_6)

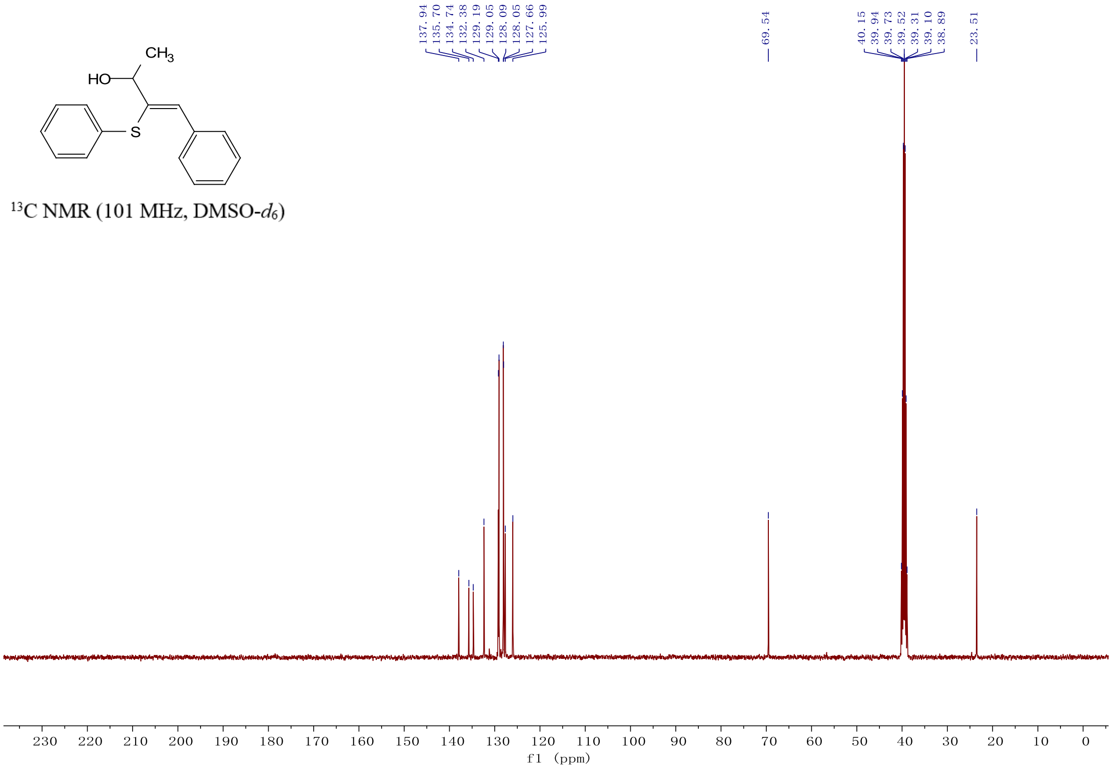

^{13}C NMR (101 MHz, DMSO-d_6)

(*Z*)-4-phenyl-3-(phenylthio)but-3-en-2-ol (*Z*-9ag)

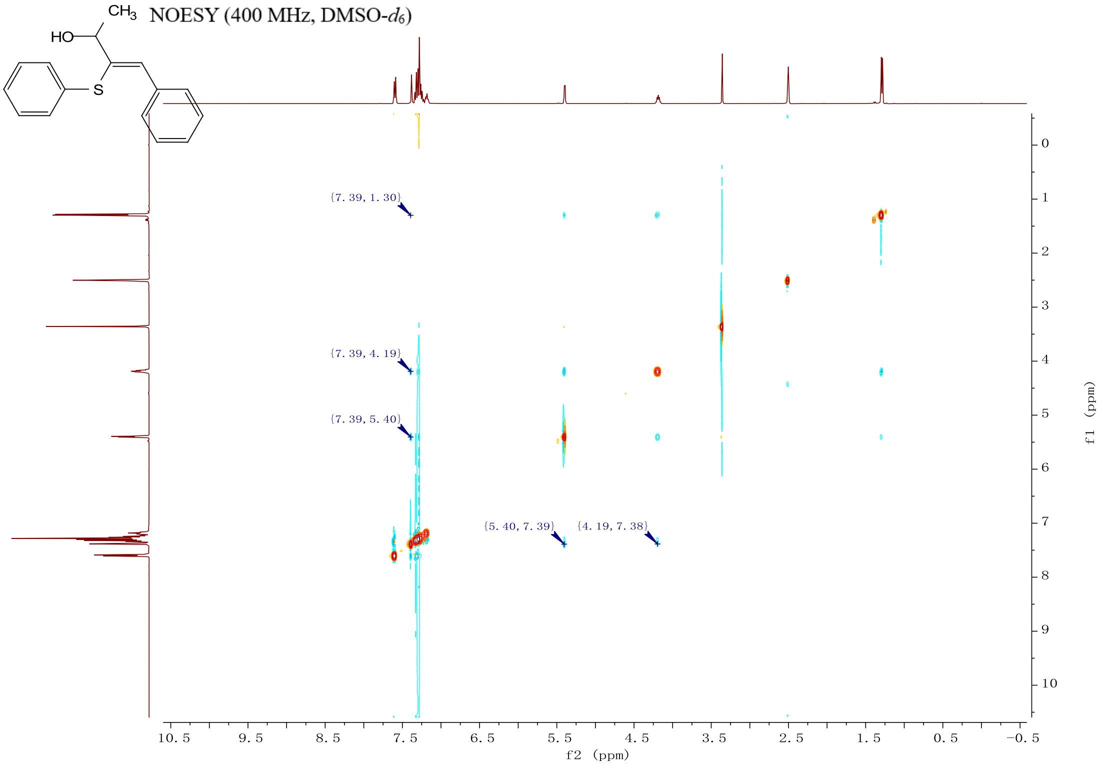

NOESY (400 MHz, DMSO-d_6)

(*E*)-4-phenyl-3-(phenylthio)but-3-en-2-ol (*E*-9ag)

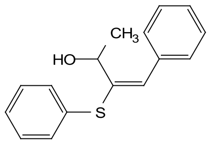

^{1}H NMR (400 MHz, DMSO-d_6)

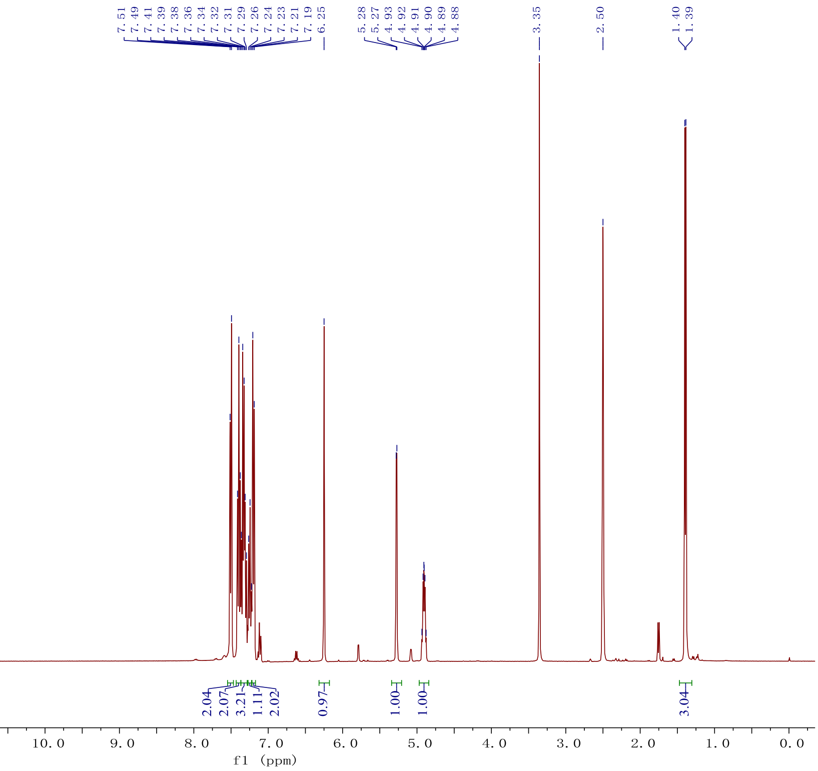

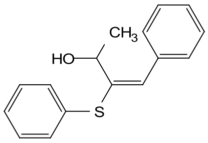

^{13}C NMR (101 MHz, DMSO-d_6)

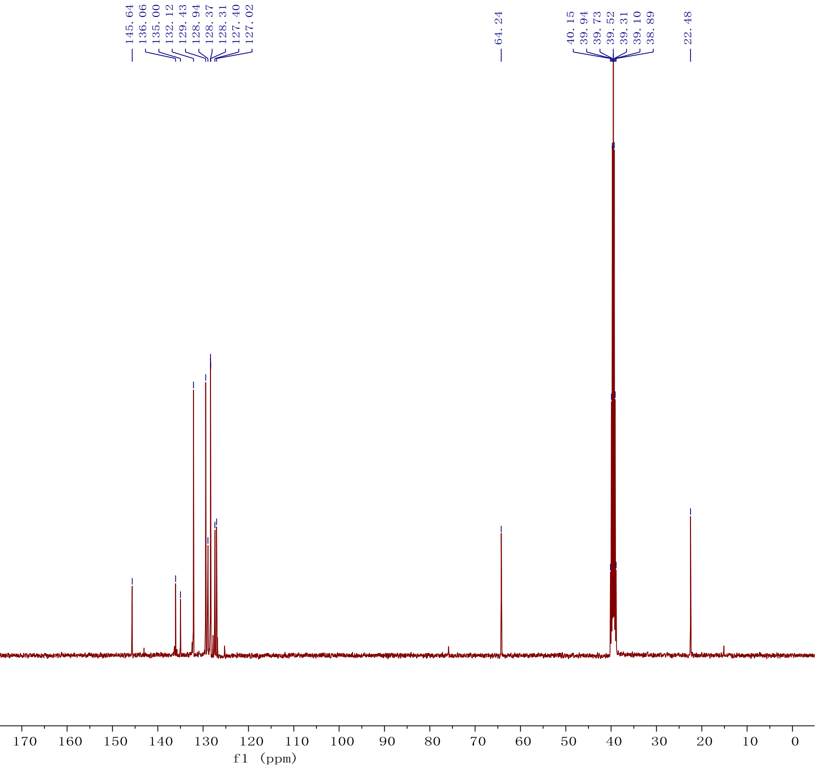

(*E*)-4-phenyl-3-(phenylthio)but-3-en-2-ol (*E*-9ag)

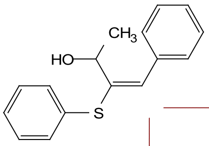

NOESY (400 MHz, DMSO-d_6)

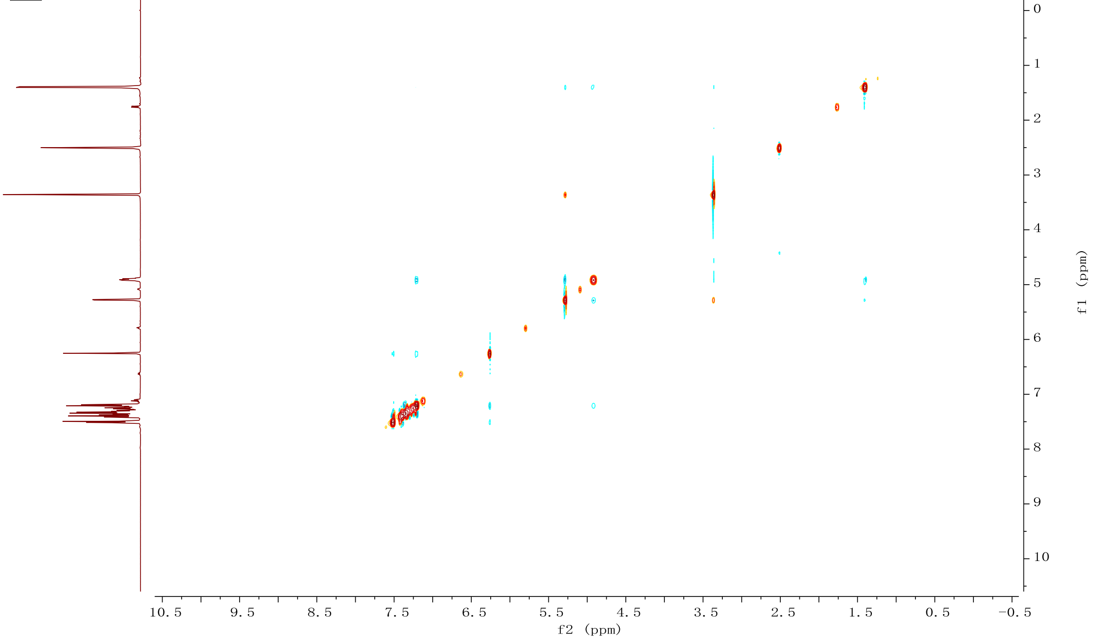

(*Z*)-3-((4-chlorophenyl)thio)-4-phenylbut-3-en-2-ol (*Z*-9ah)

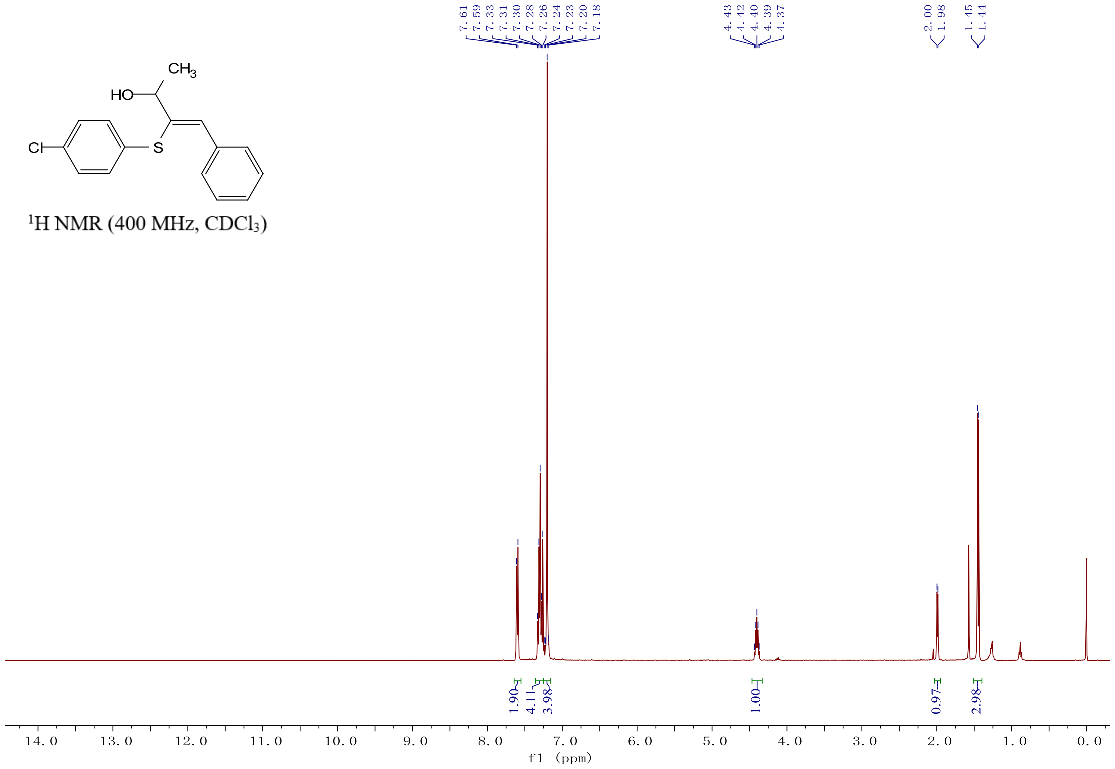

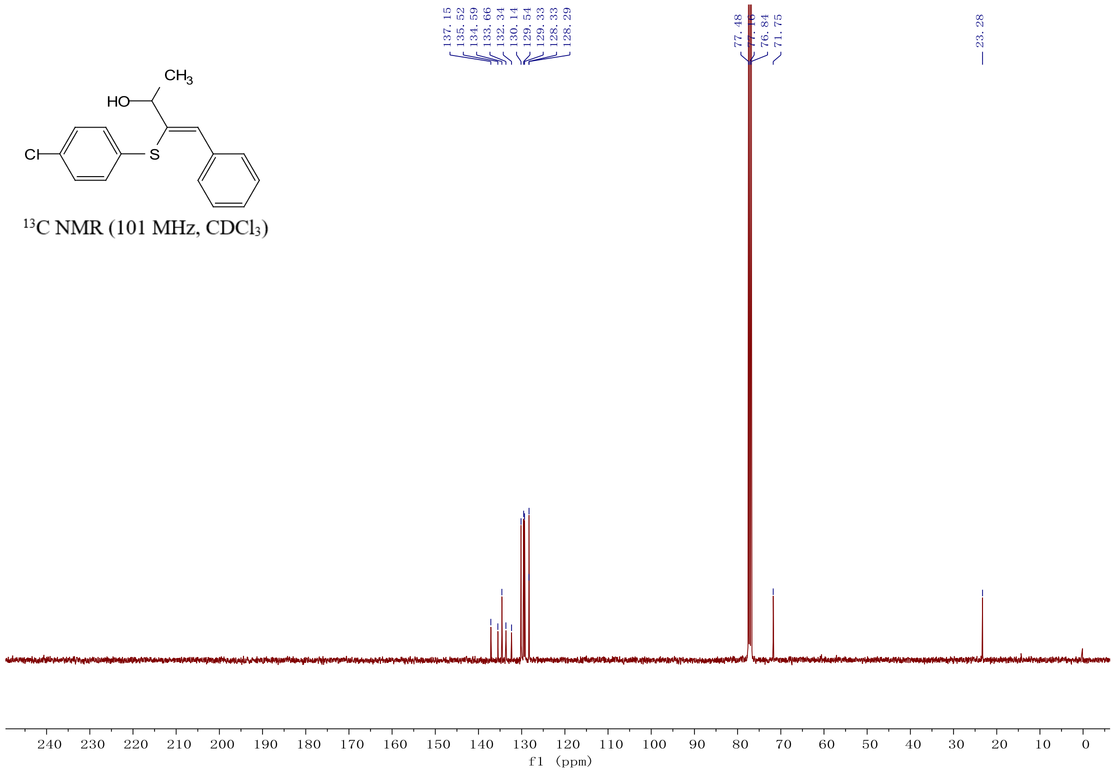

(*Z*)-3-((4-chlorophenyl)thio)-4-phenylbut-3-en-2-ol (*Z*-9ah)

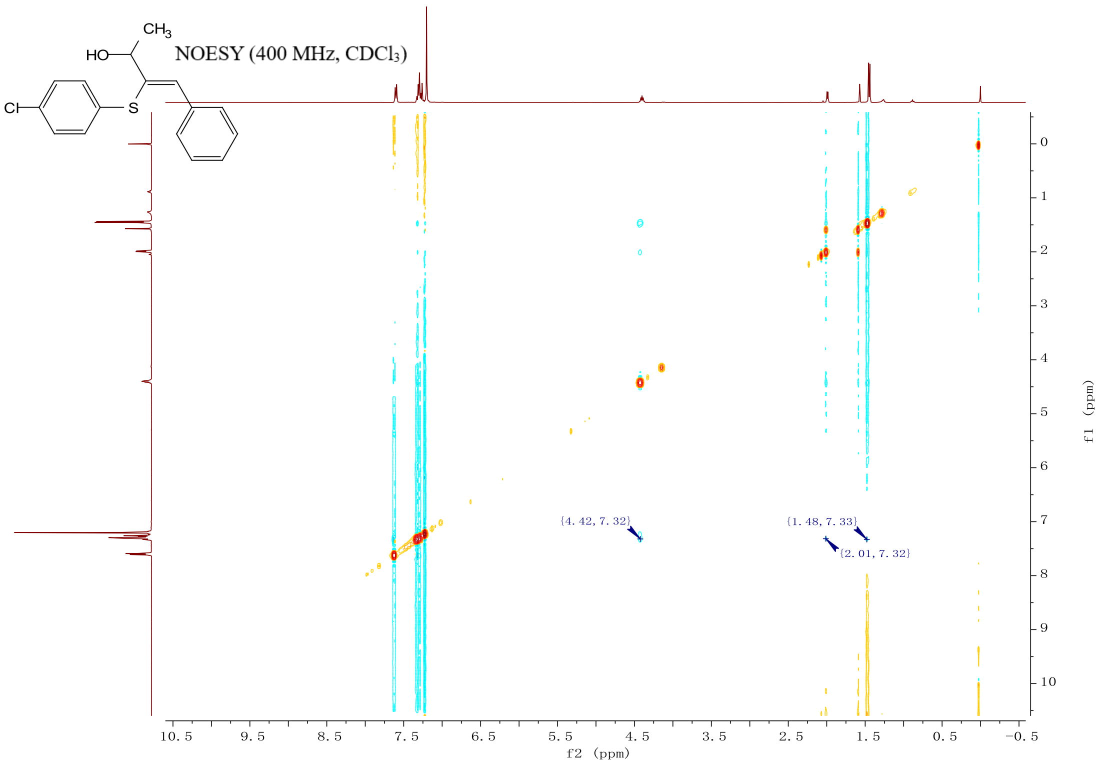

(*E*)-3-((4-chlorophenyl)thio)-4-phenylbut-3-en-2-ol (*E*-9ah)

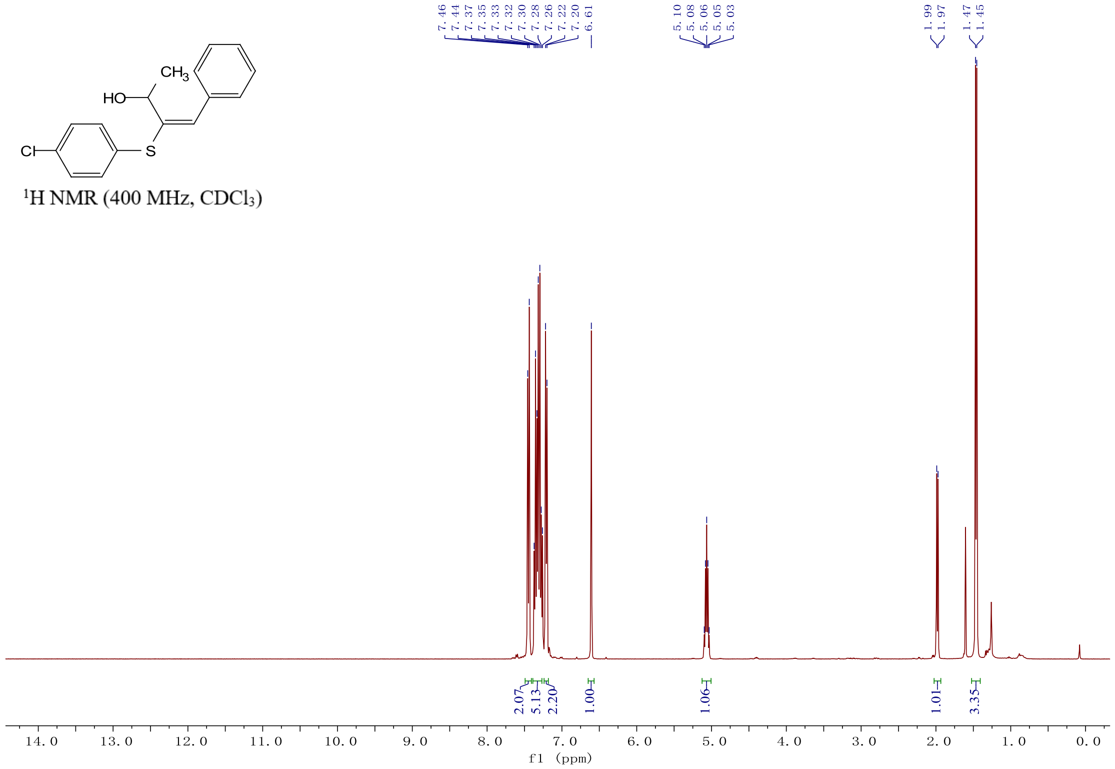

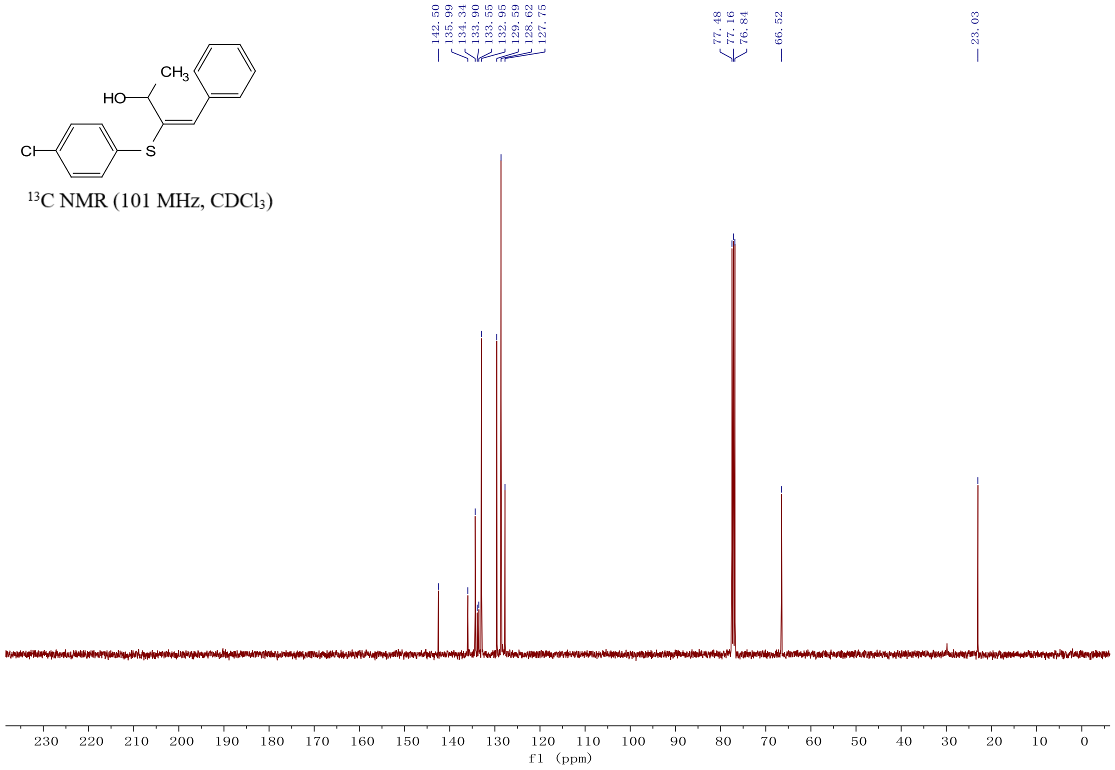

(*E*)-3-((4-chlorophenyl)thio)-4-phenylbut-3-en-2-ol (*E*-9ah)

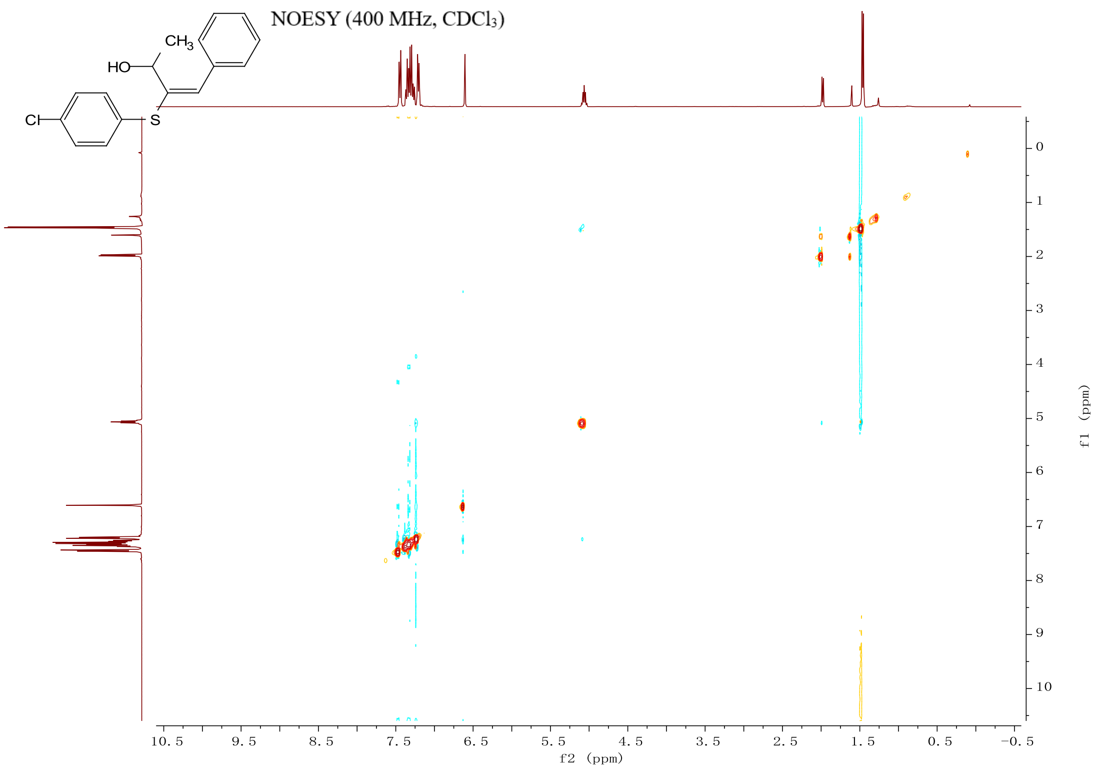

(*Z*)-1-phenyl-2-(phenylthio)but-2-en-1-ol (*Z*-9ai)

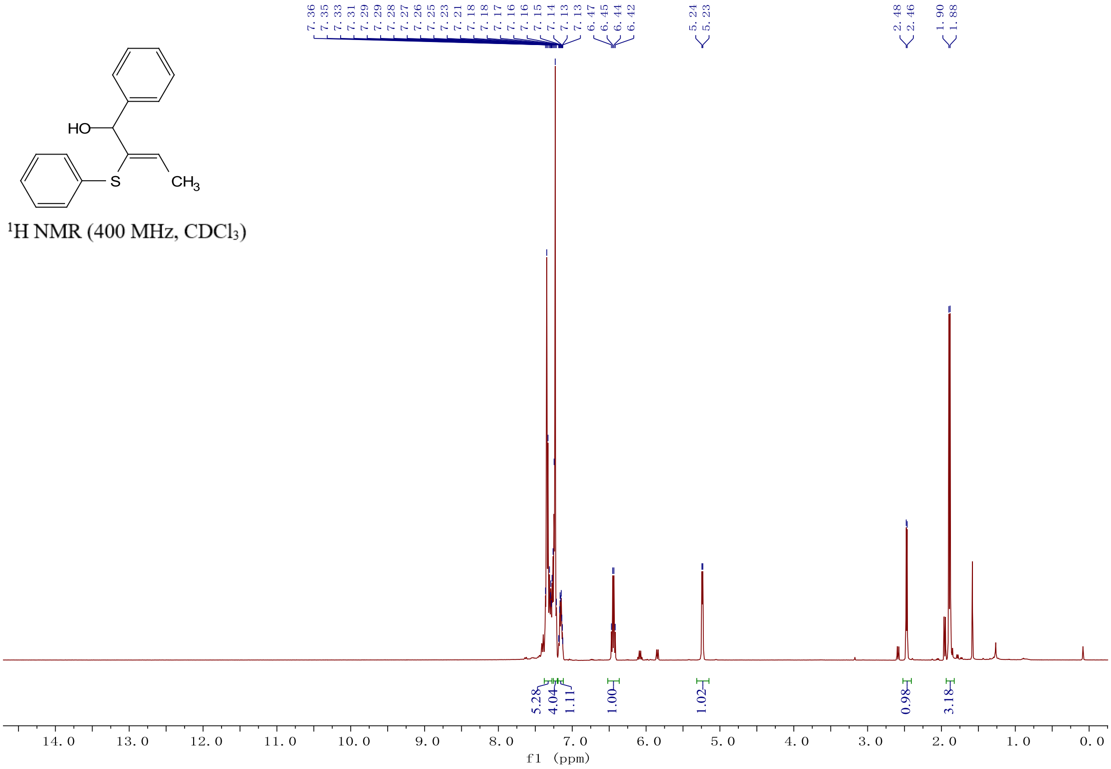

^{1}H NMR (400 MHz, $CDCl_3$)

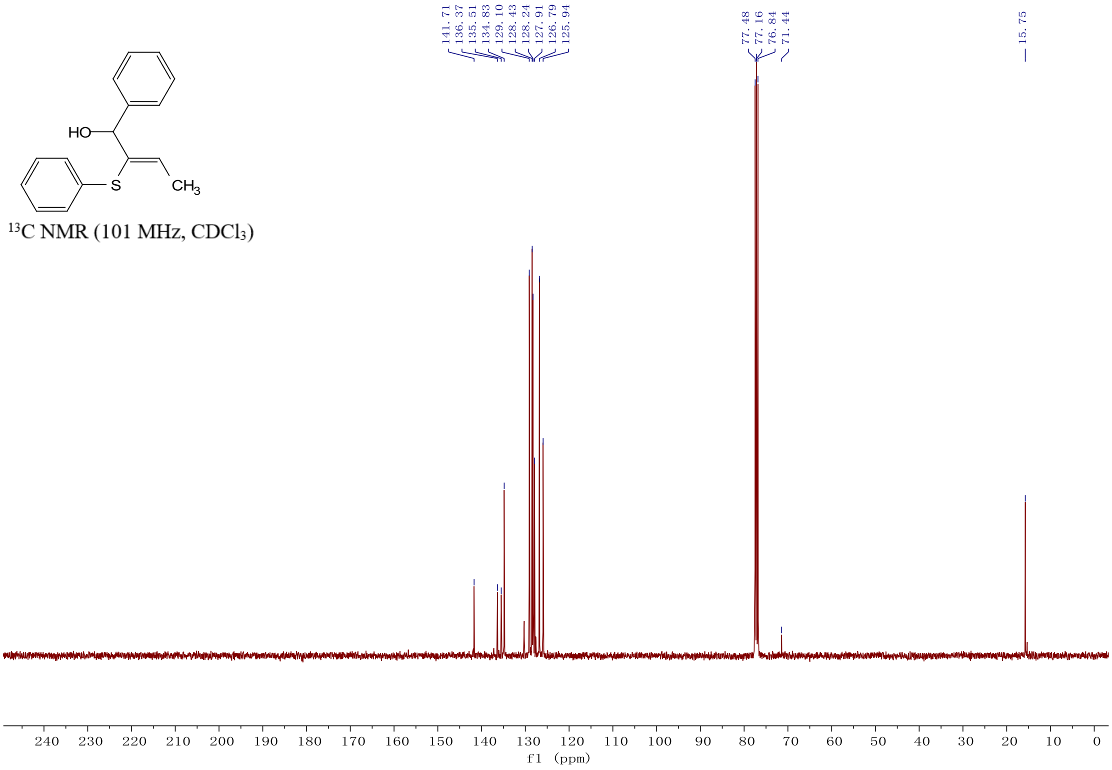

^{13}C NMR (101 MHz, $CDCl_3$)

(*Z*)-1-phenyl-2-(phenylthio)but-2-en-1-ol (*Z*-9ai)

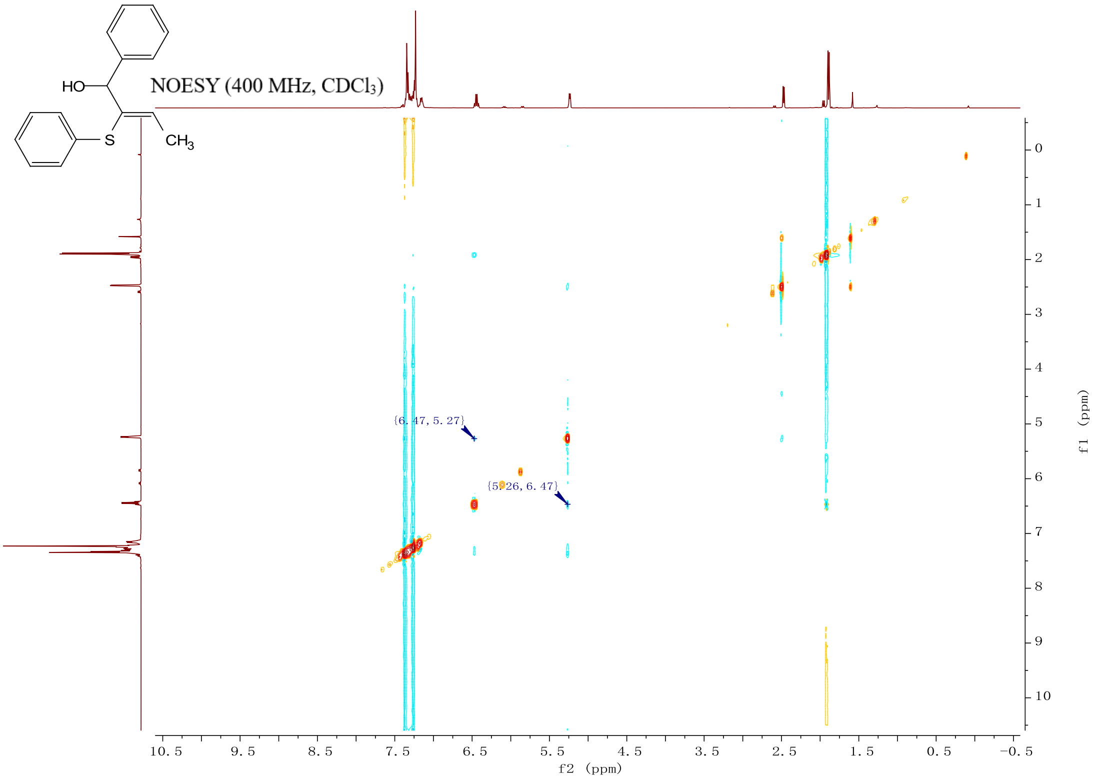

(*Z*)-2-(phenylthio)pent-2-en-1-ol (*Z*-9aj)

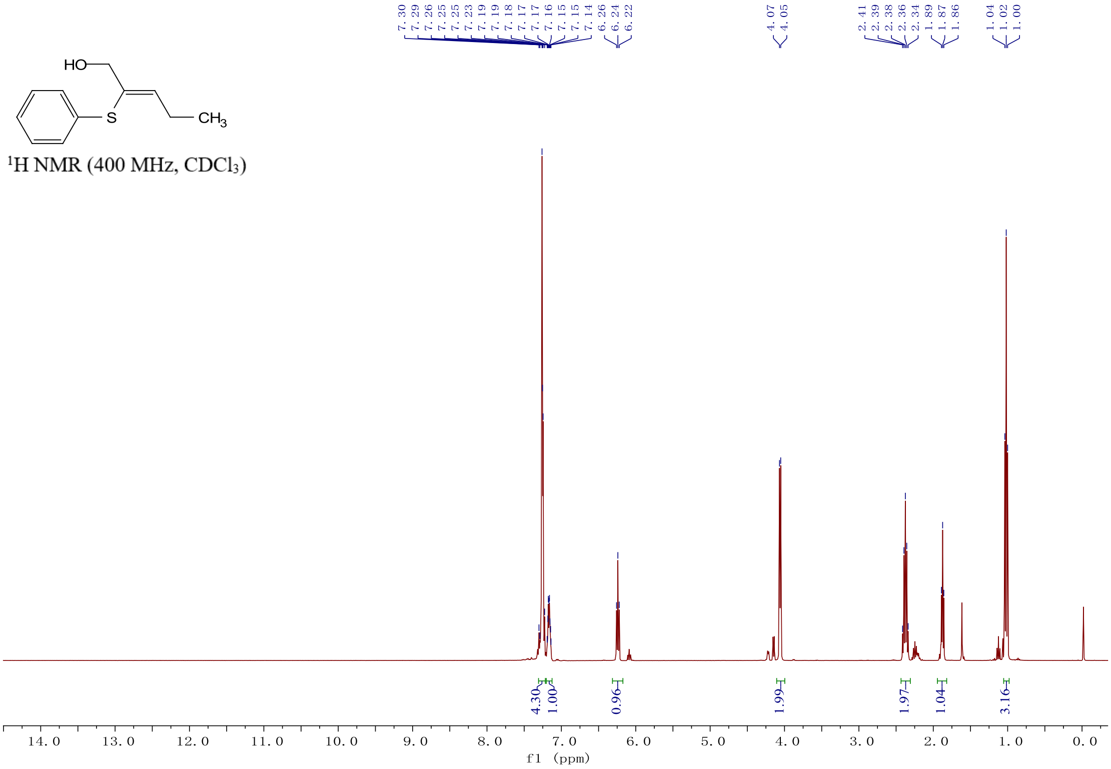

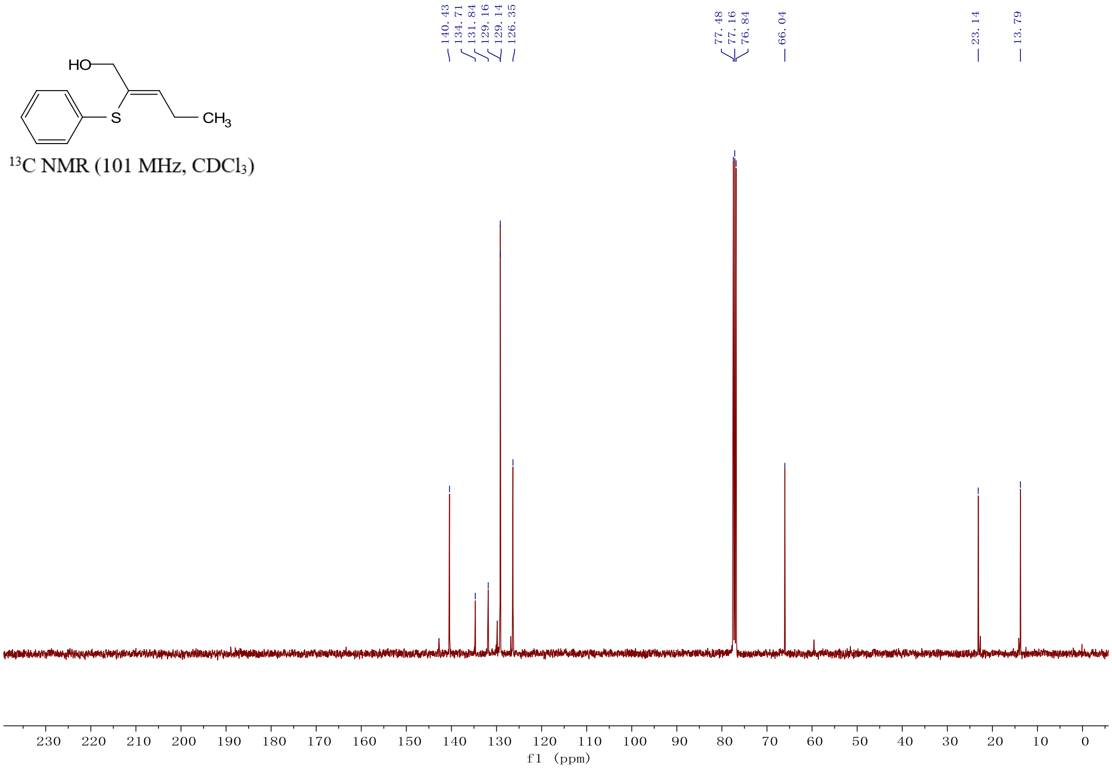

(*Z*)-3-(phenylthio)hex-3-en-2-one (*Z*-5aa)

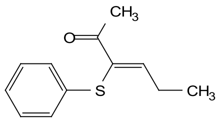

^{1}H NMR (500 MHz, $CDCl_3$)

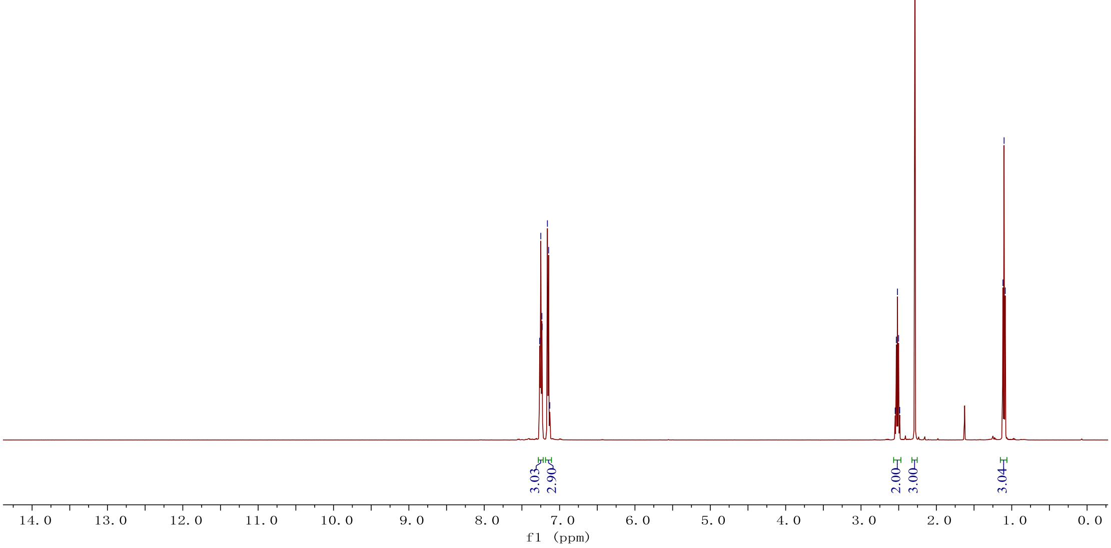

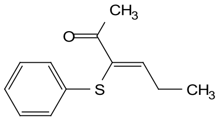

^{13}C NMR (126 MHz, $CDCl_3$)

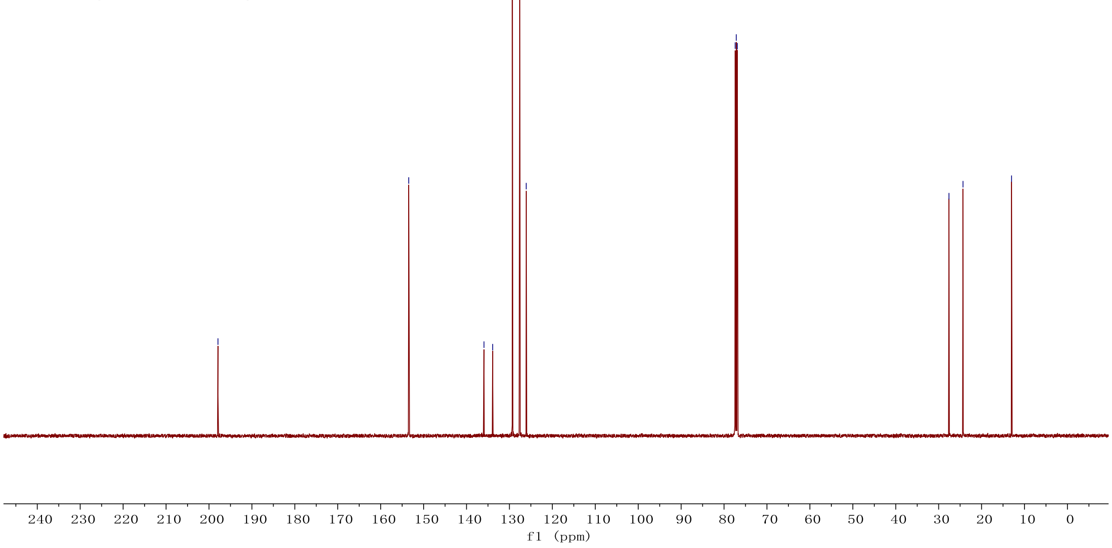

(*Z*)-3-(phenylthio)hex-3-en-2-one (*Z*-5aa)

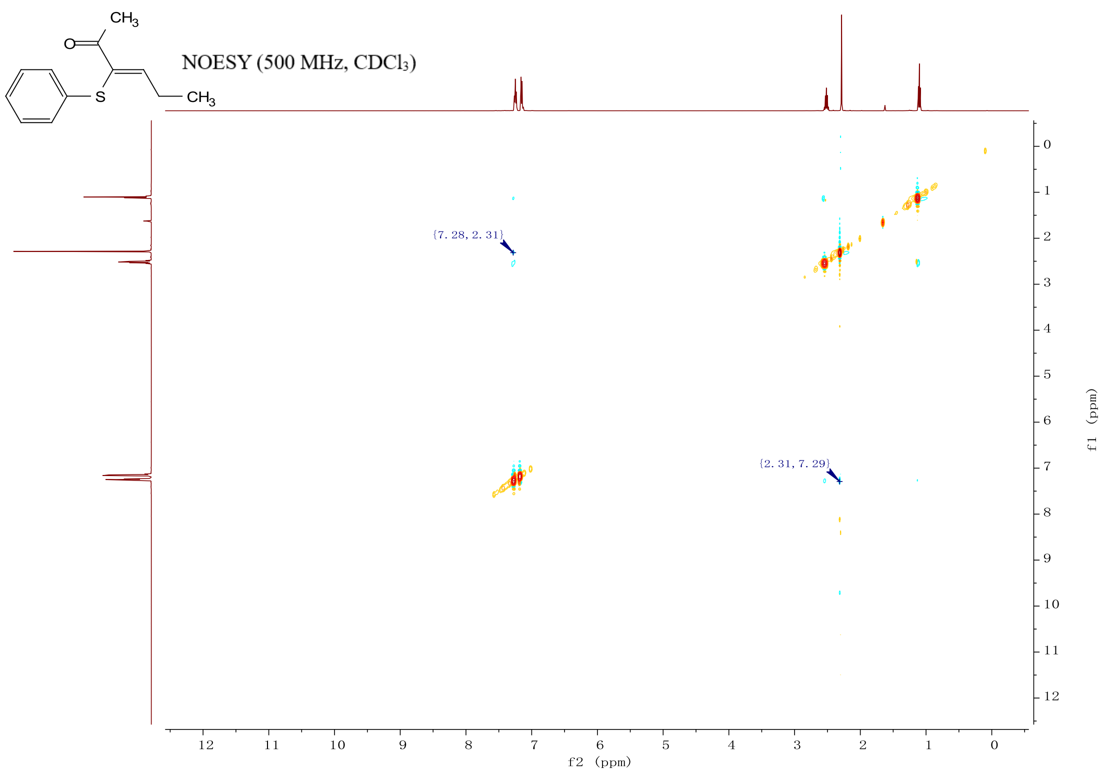

(*Z*)-3-((2-fluorophenyl)thio)hex-3-en-2-one (*Z*-5ab)

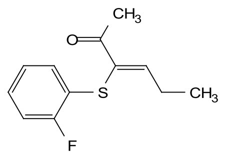

^{1}H NMR (400 MHz, CDCl3)

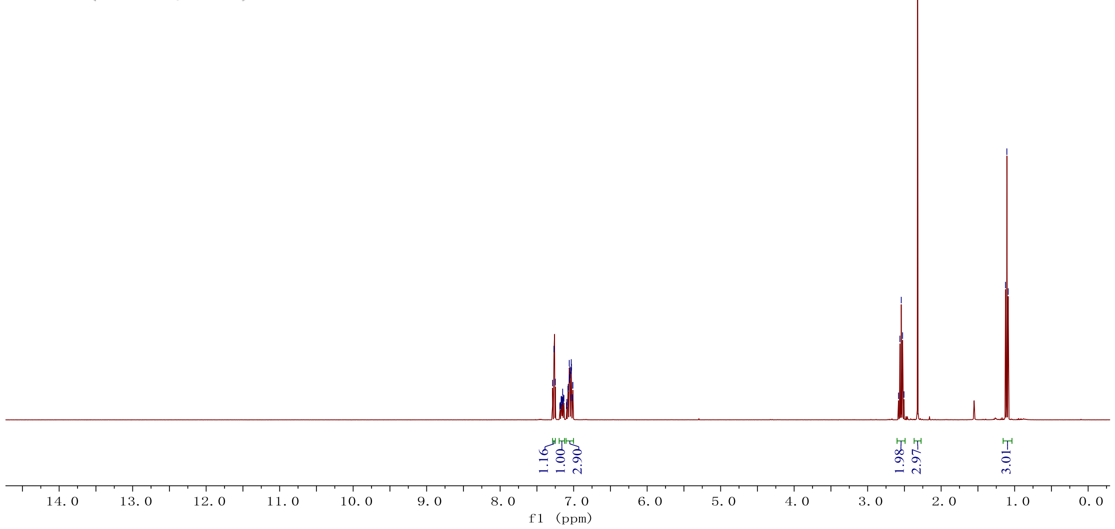

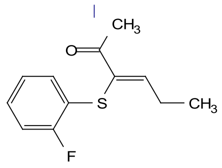

^{13}C NMR (101 MHz, CDCl3)

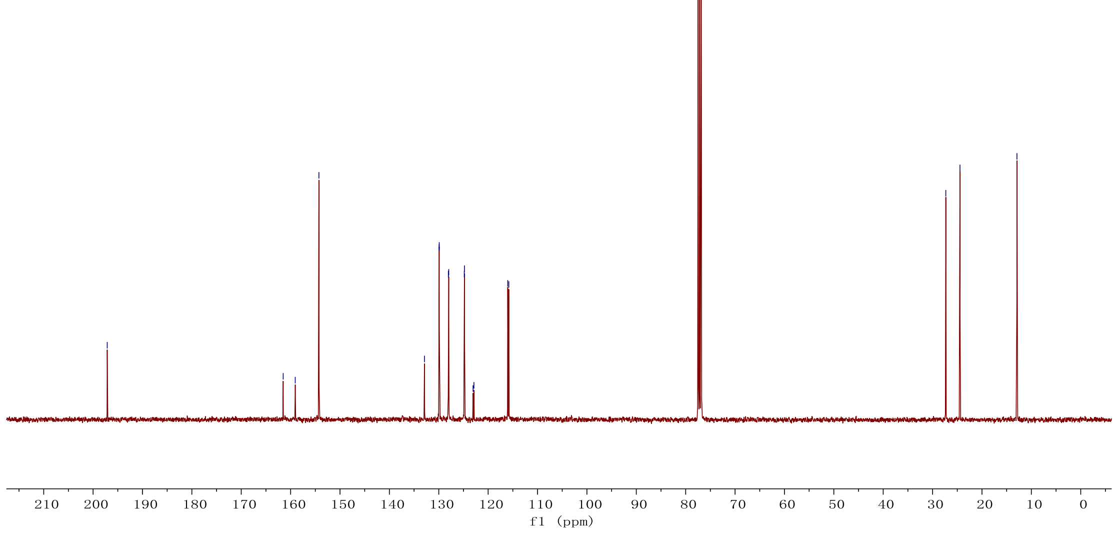

(*Z*)-3-((2-fluorophenyl)thio)hex-3-en-2-one (*Z*-5ab)

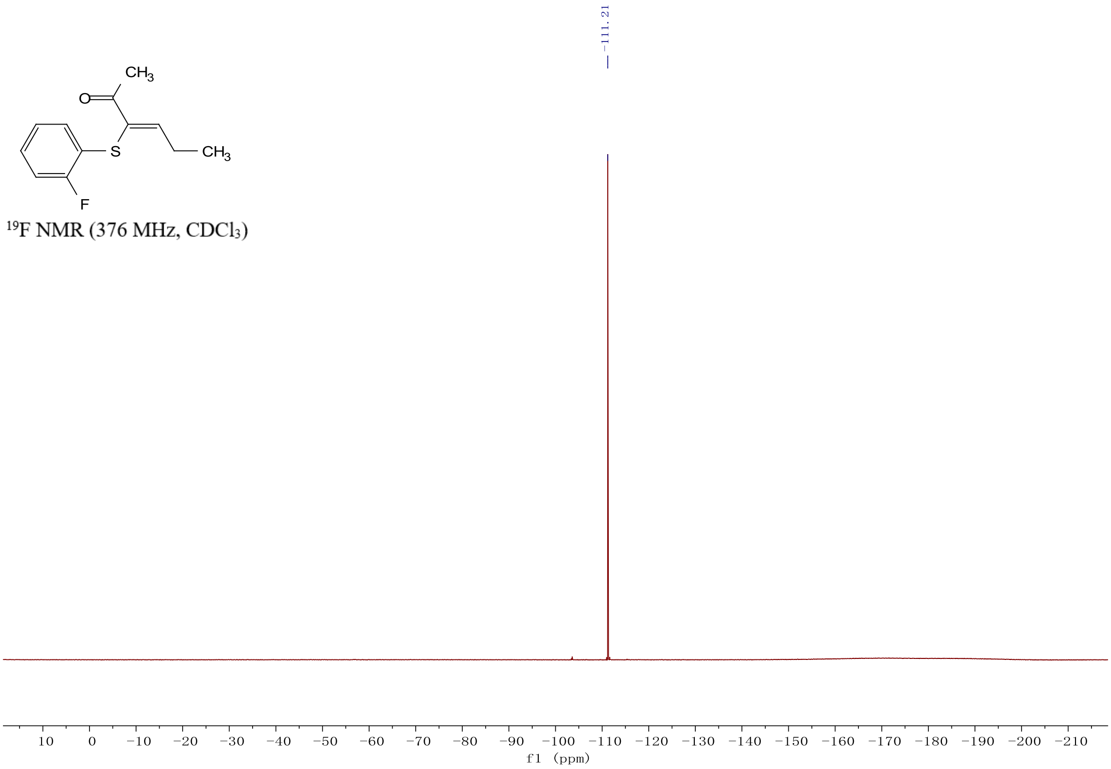

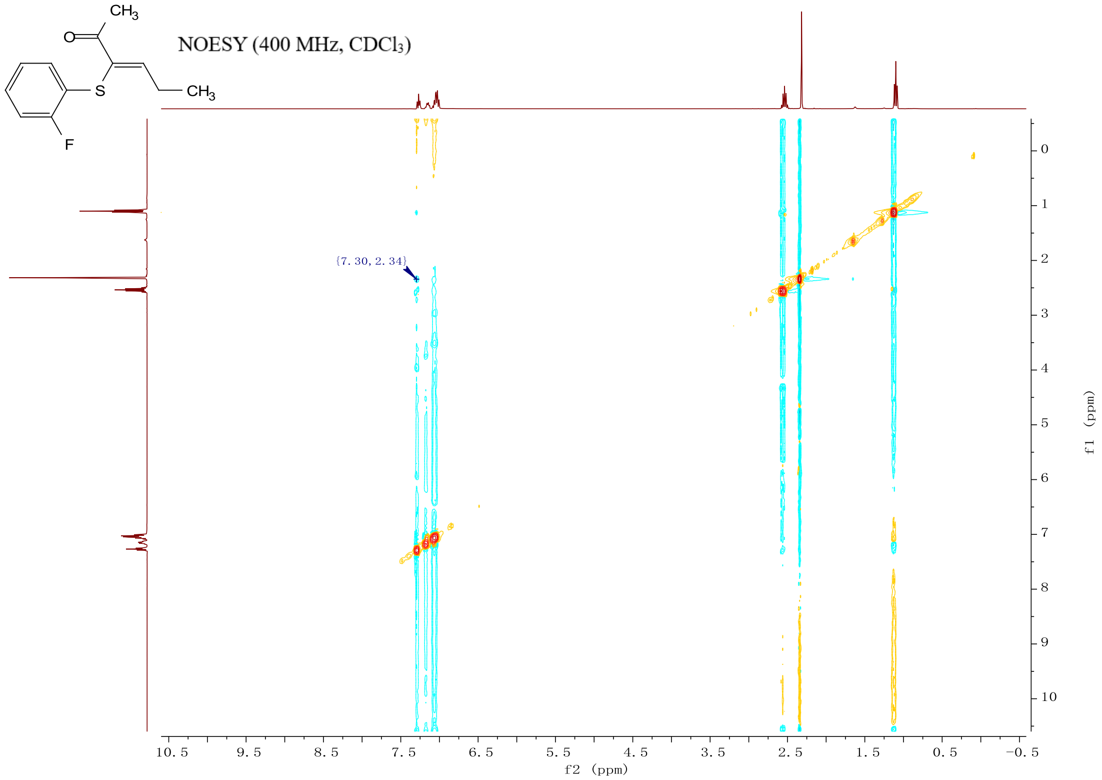

(*Z*)-3-((2-chlorophenyl)thio)hex-3-en-2-one (*Z*-5ac)

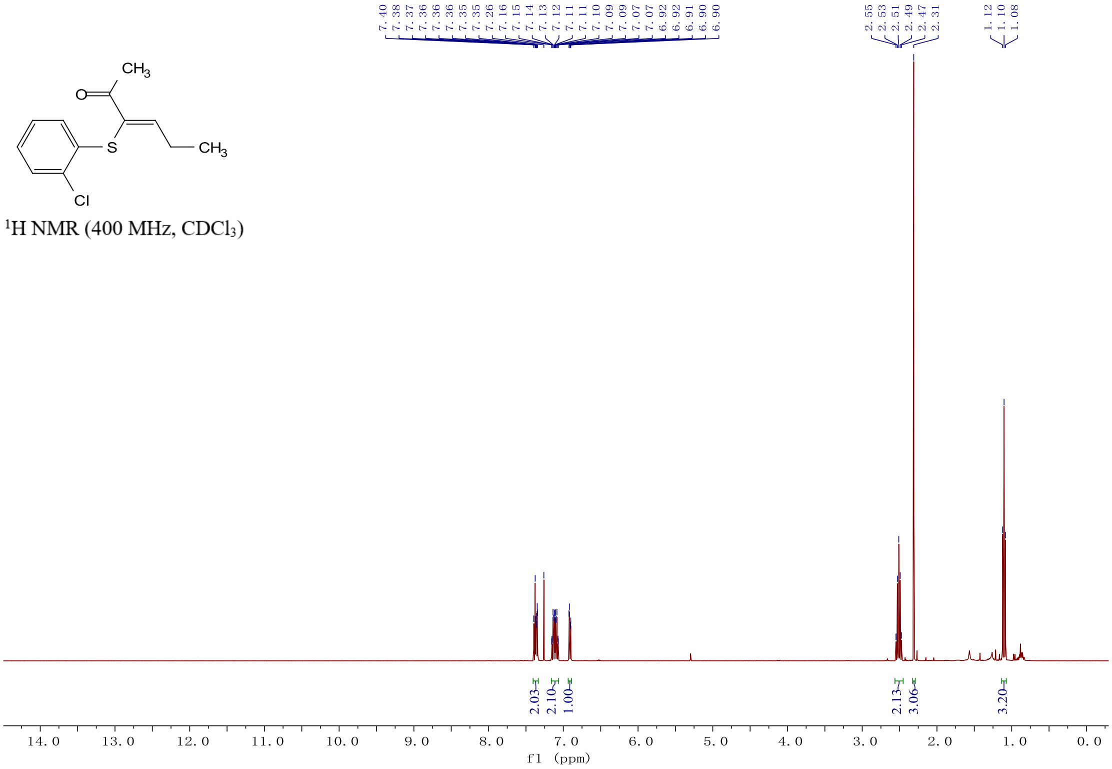

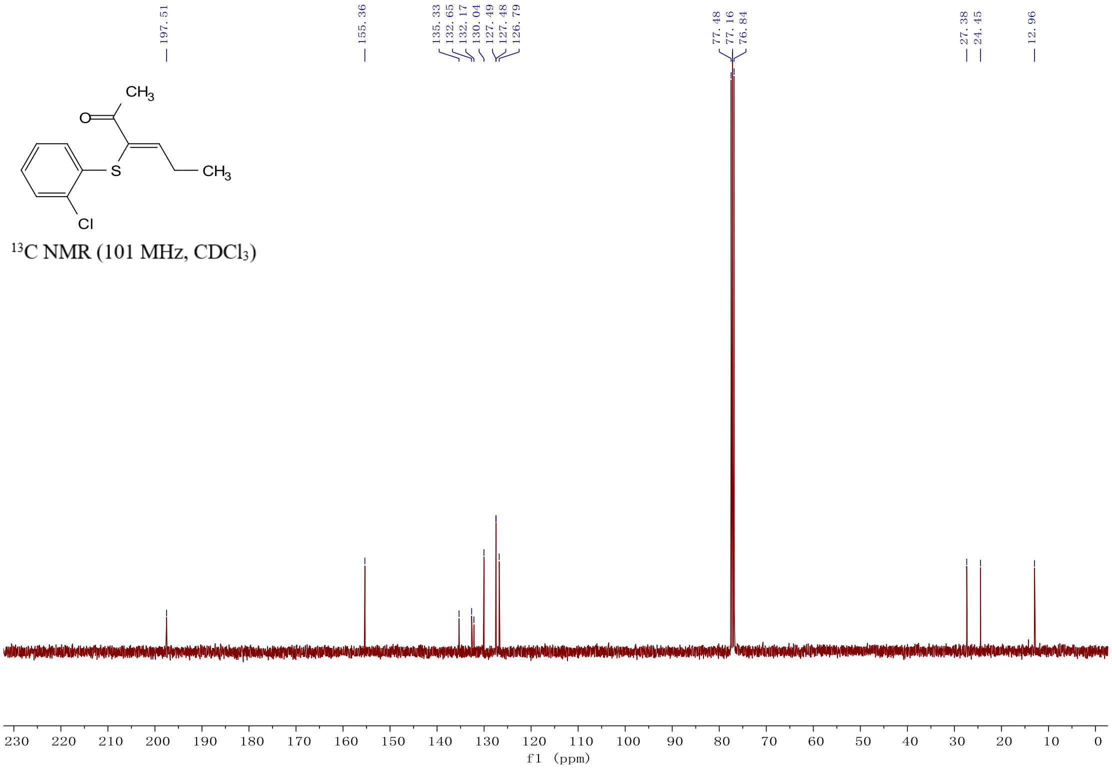

(*Z*)-3-((4-chlorophenyl)thio)hex-3-en-2-one (*Z*-5ad)

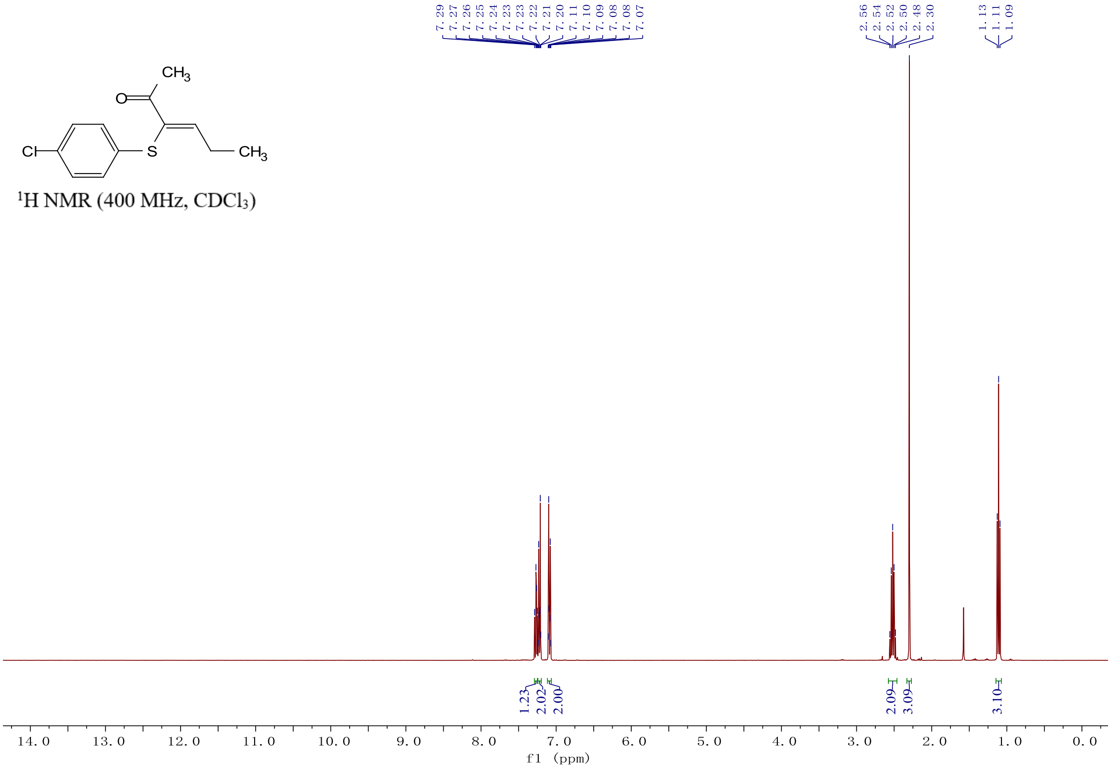

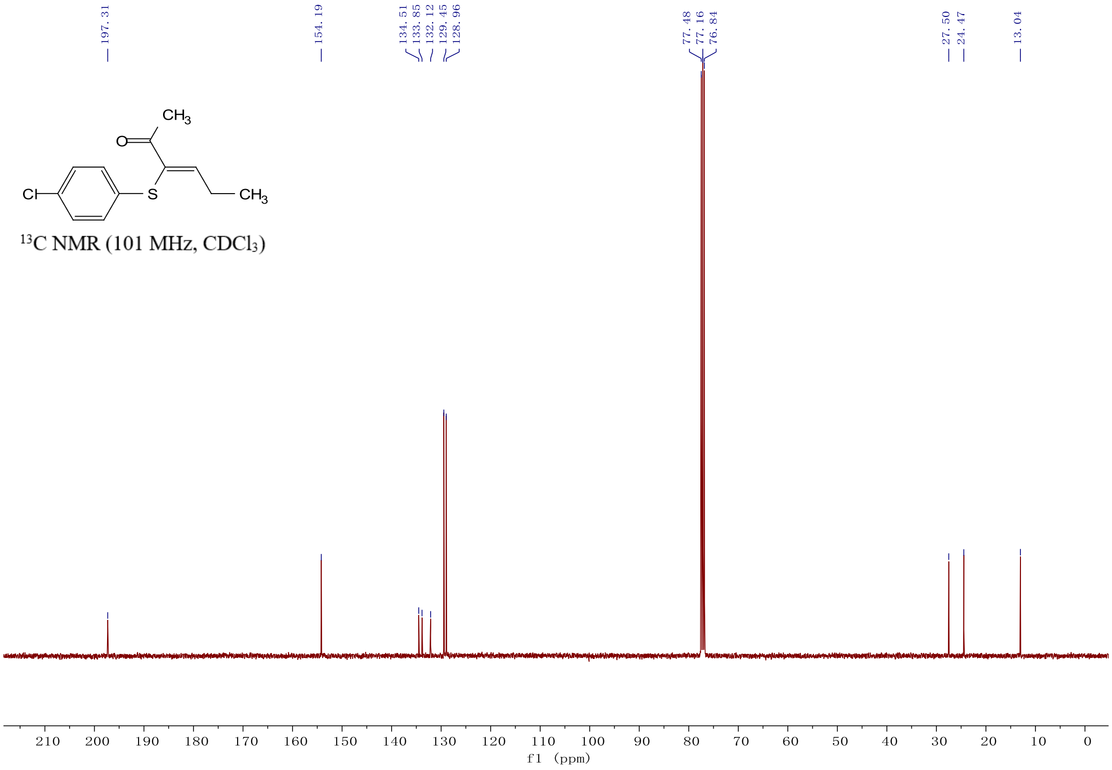

(*Z*)-3-((2-bromophenyl)thio)hex-3-en-2-one (*Z*-5ae)

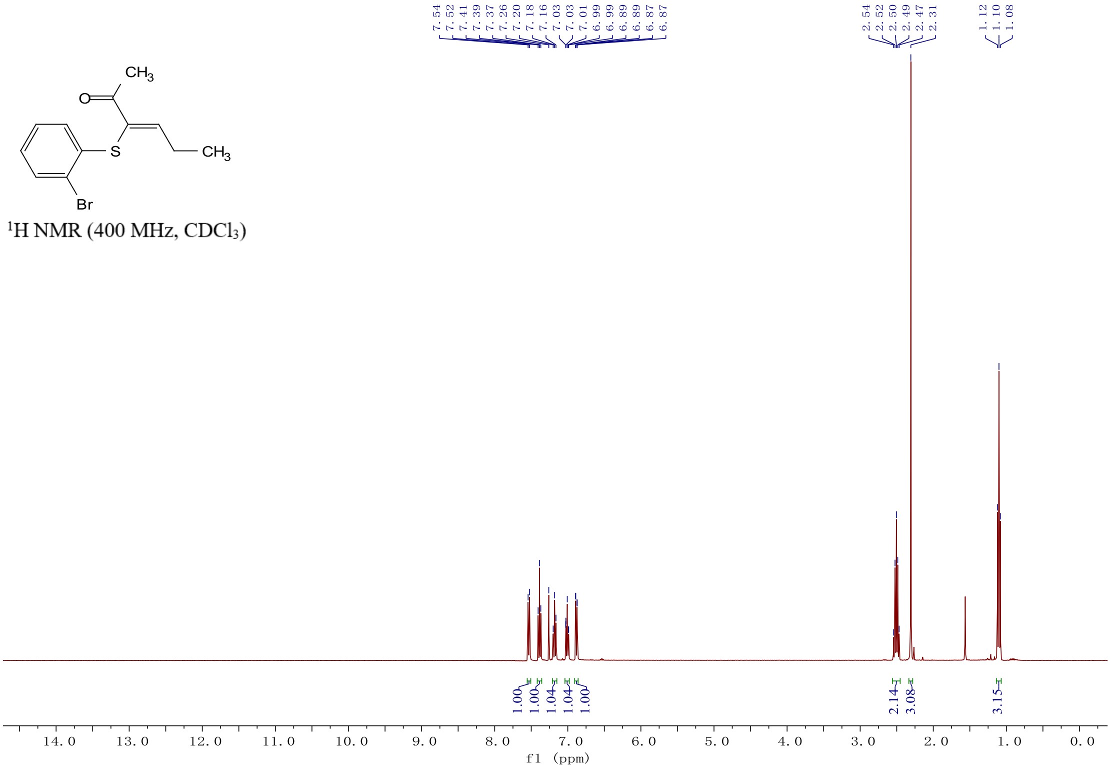

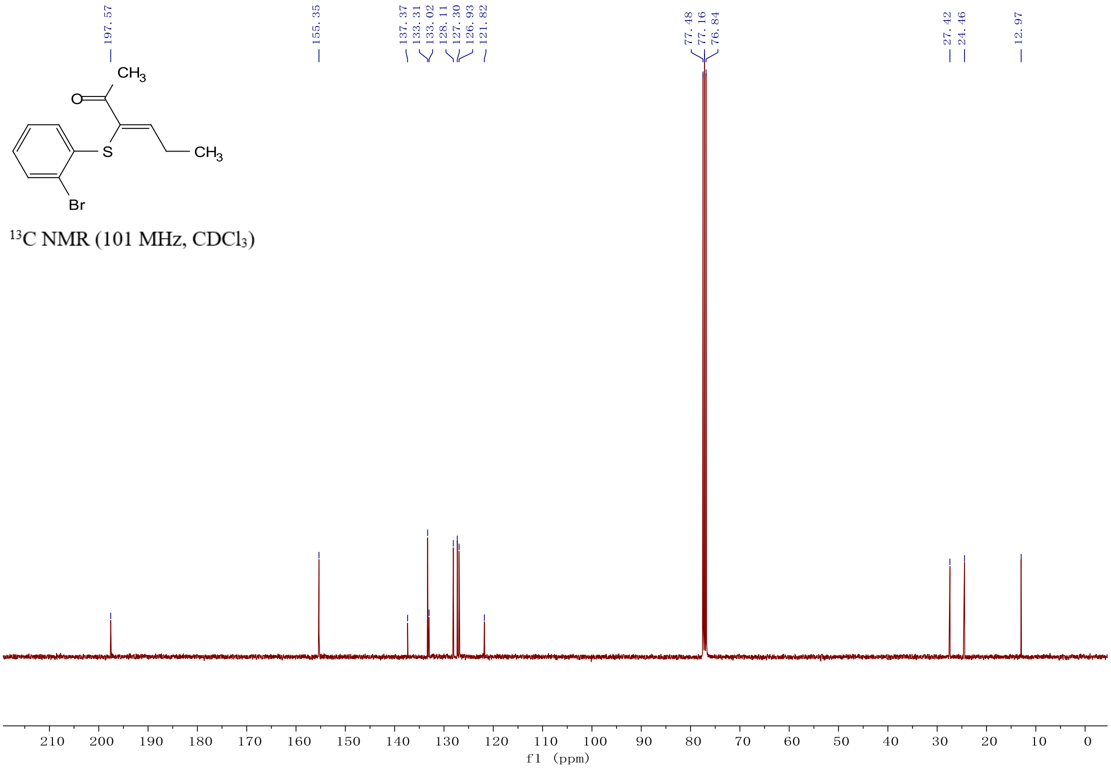

(*Z*)-3-((4-bromophenyl)thio)hex-3-en-2-one (*Z*-5af)

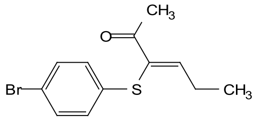

^{1}H NMR (400 MHz, $CDCl_3$)

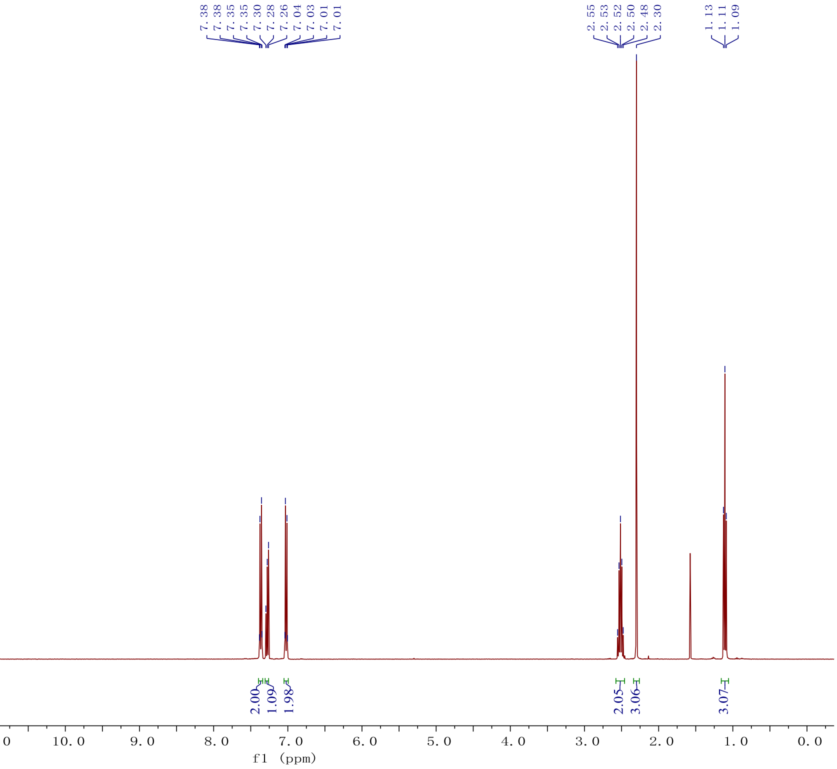

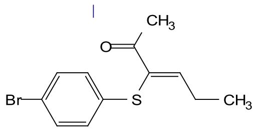

^{13}C NMR (101 MHz, $CDCl_3$)

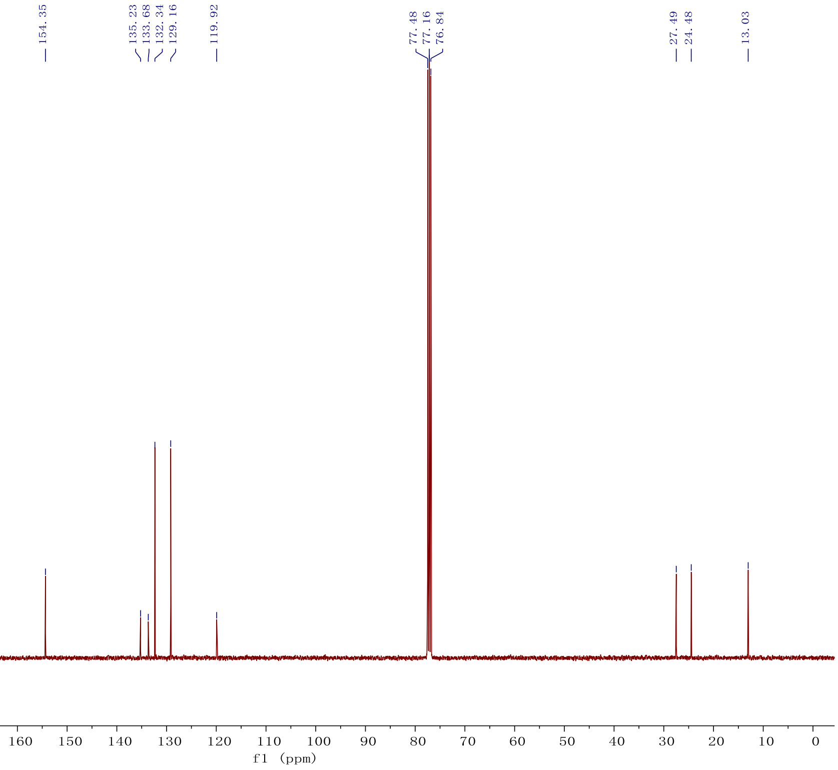

(*Z*)-4-phenyl-3-(phenylthio)but-3-en-2-one (*Z*-5ag)

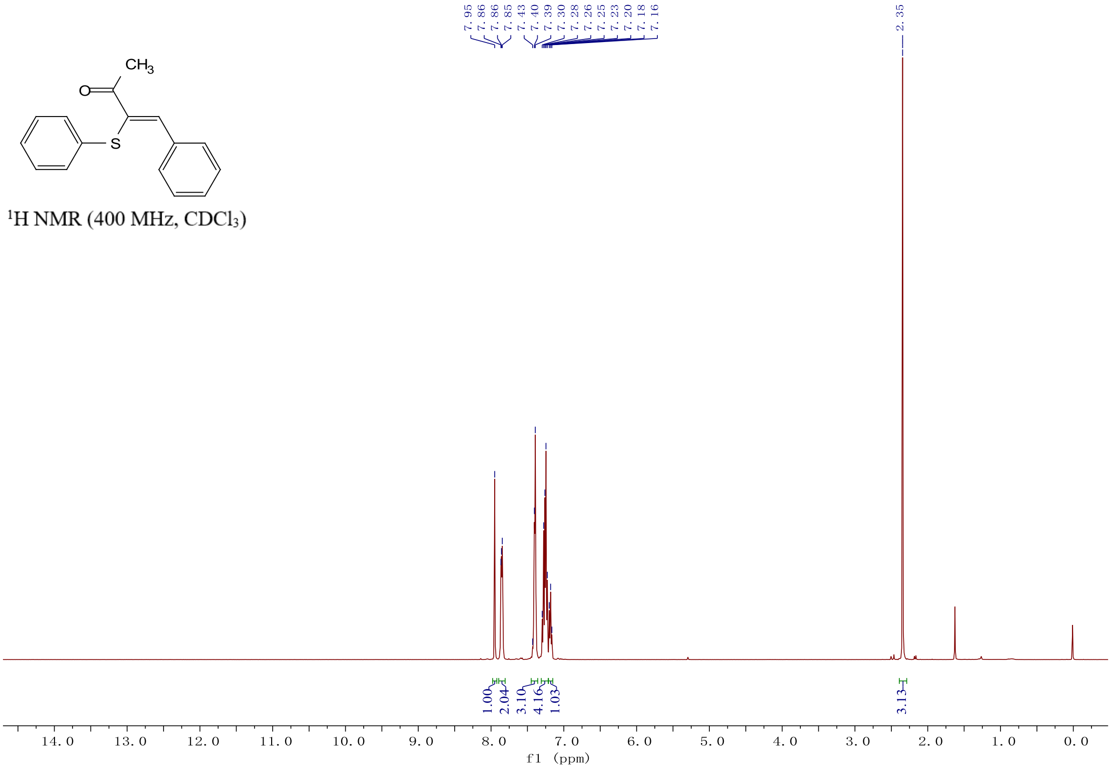

^{1}H NMR (400 MHz, $CDCl_3$)

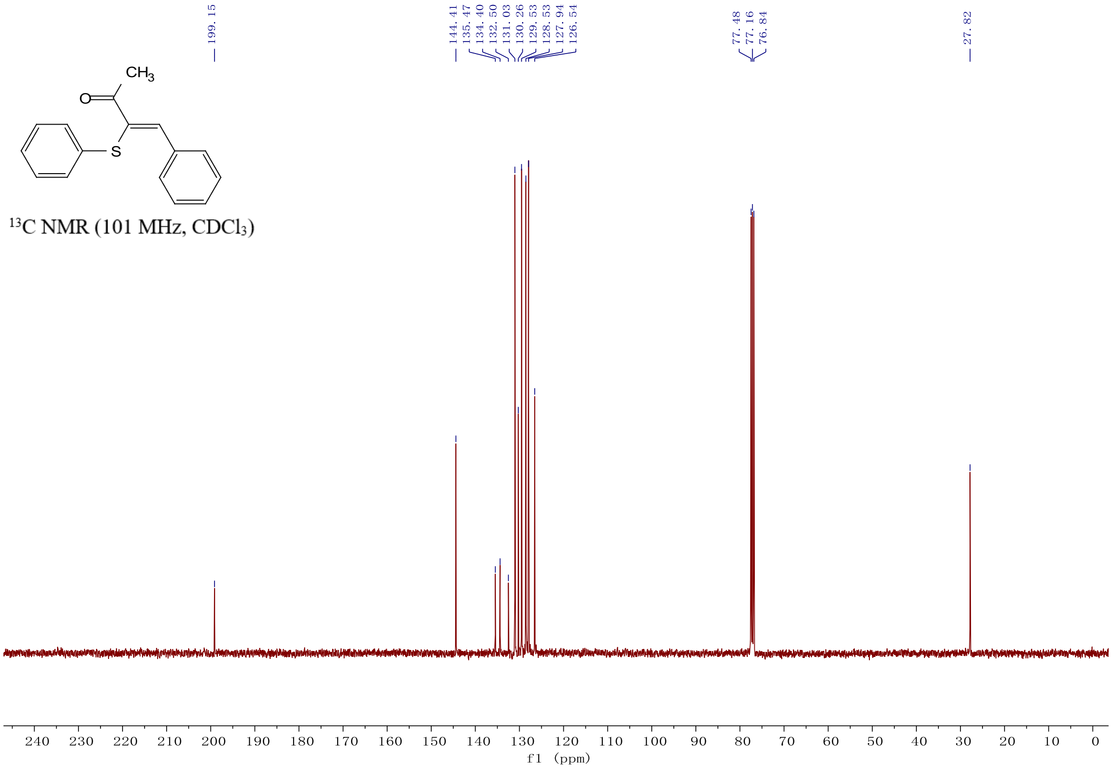

^{13}C NMR (101 MHz, $CDCl_3$)

(*Z*)-4-phenyl-3-(phenylthio)but-3-en-2-one (*Z*-5ag)

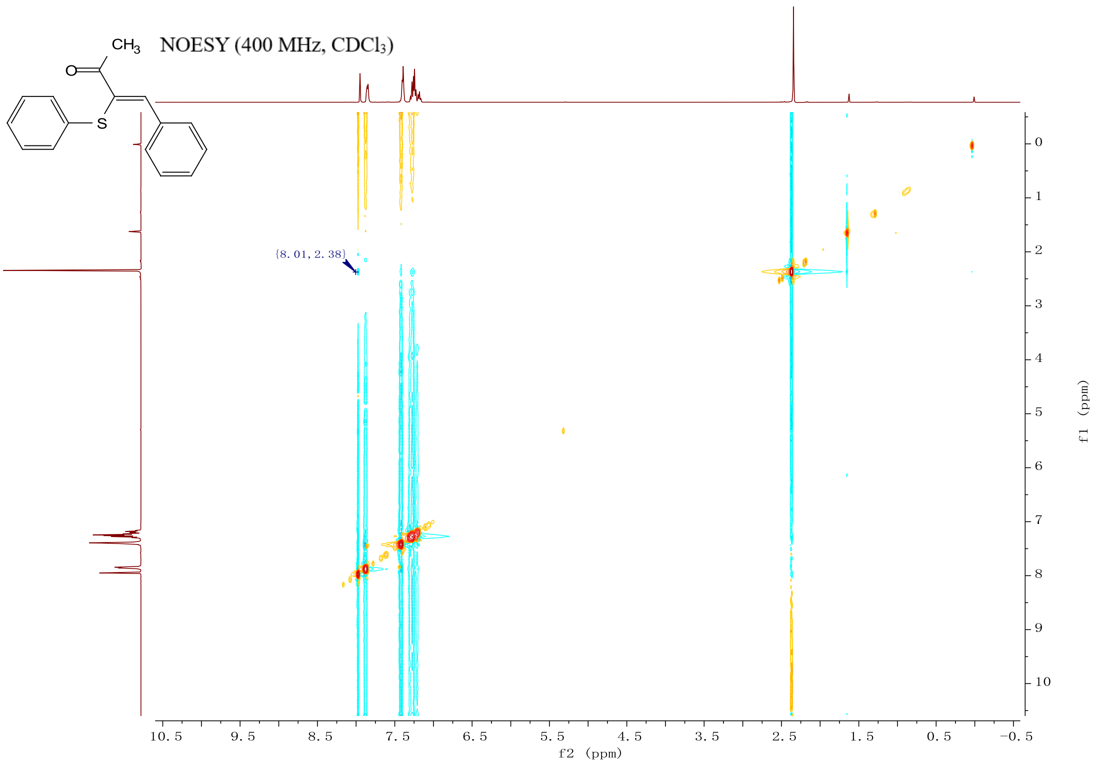

(*E*)-4-phenyl-3-(phenylthio)but-3-en-2-one (*E*-5ag)

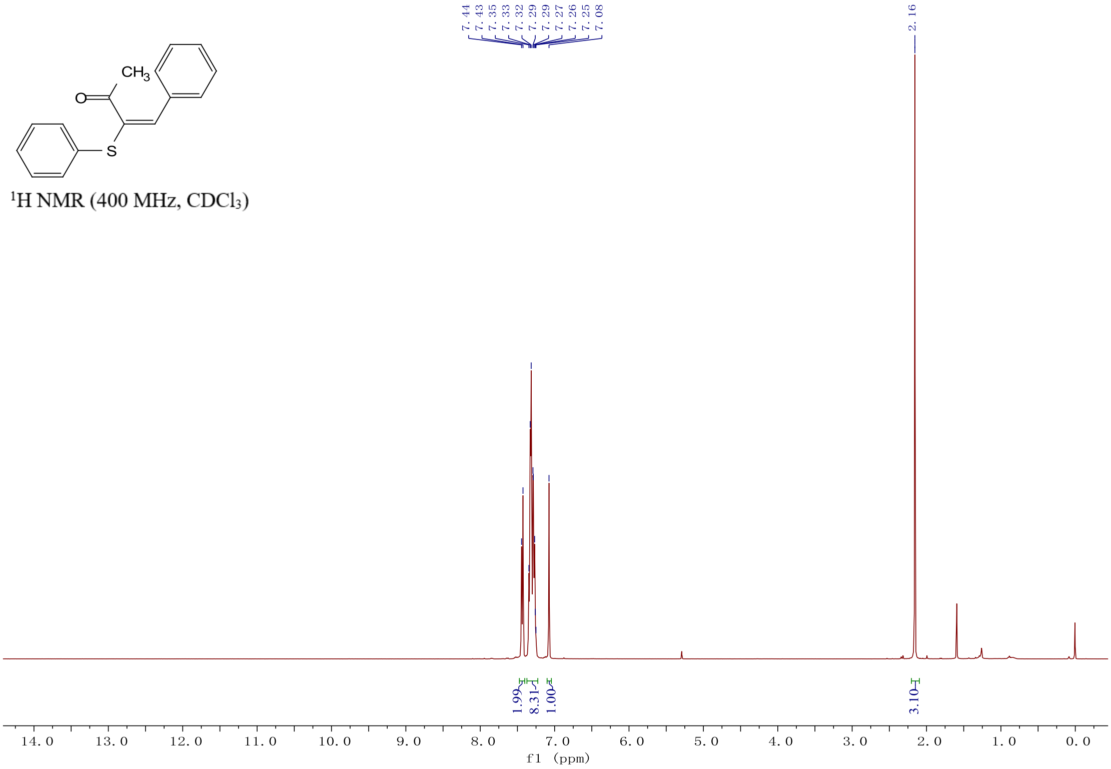

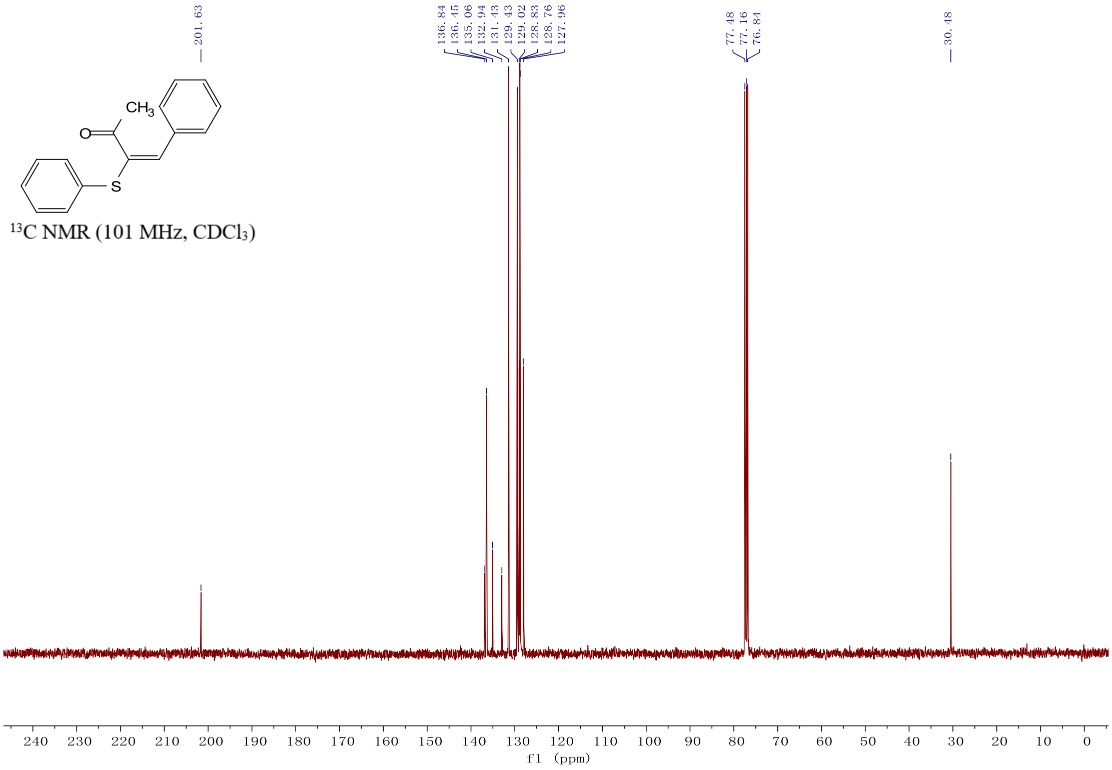

(*E*)-4-phenyl-3-(phenylthio)but-3-en-2-one (*E*-5ag)

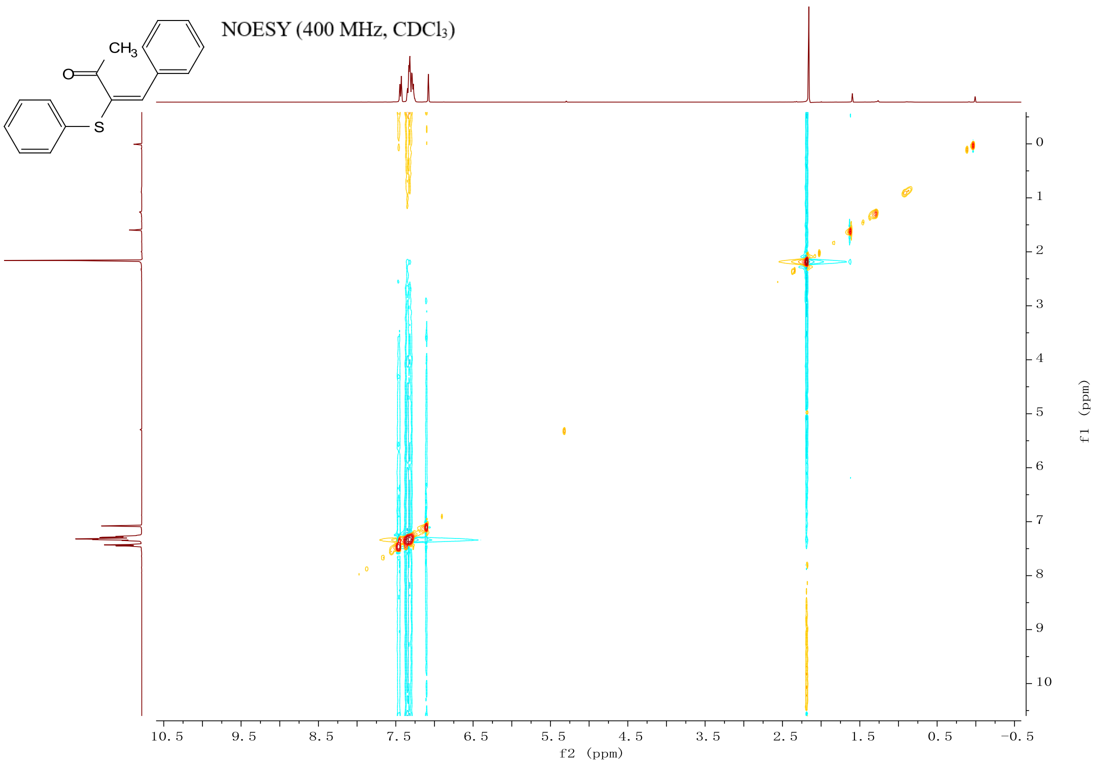

(*Z*)-3-((4-chlorophenyl)thio)-4-phenylbut-3-en-2-one (*Z*-5ah)

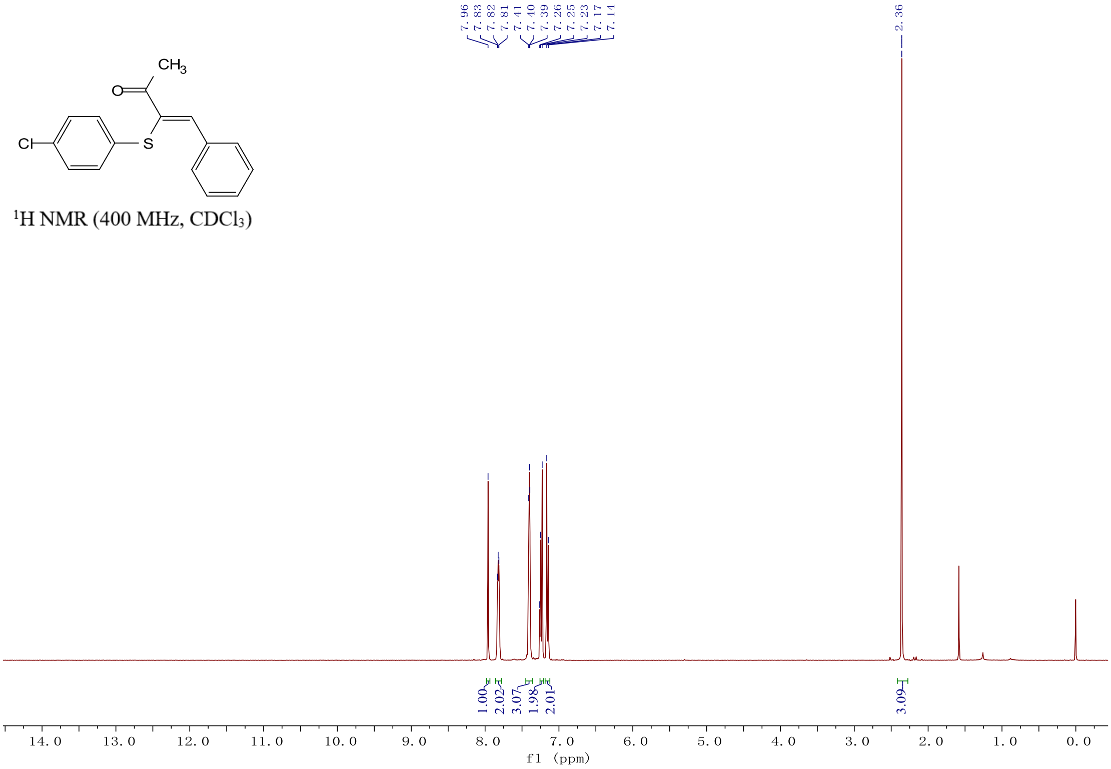

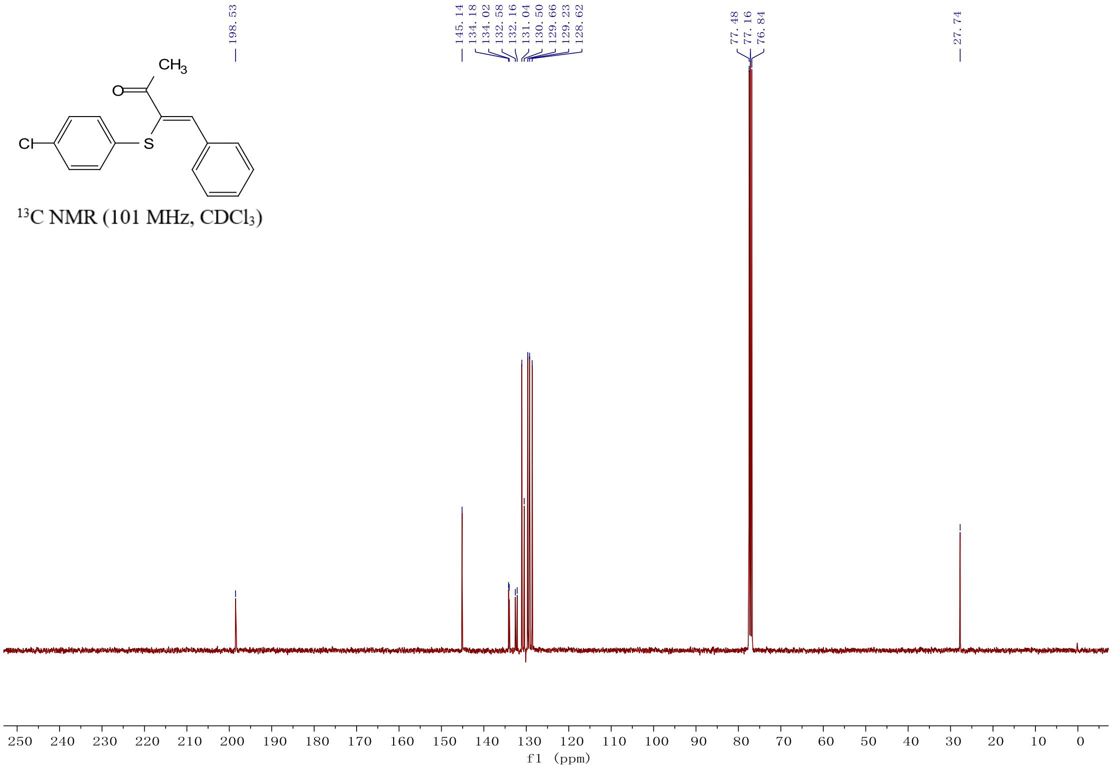

(*Z*)-3-((4-chlorophenyl)thio)-4-phenylbut-3-en-2-one (*Z*-5ah)

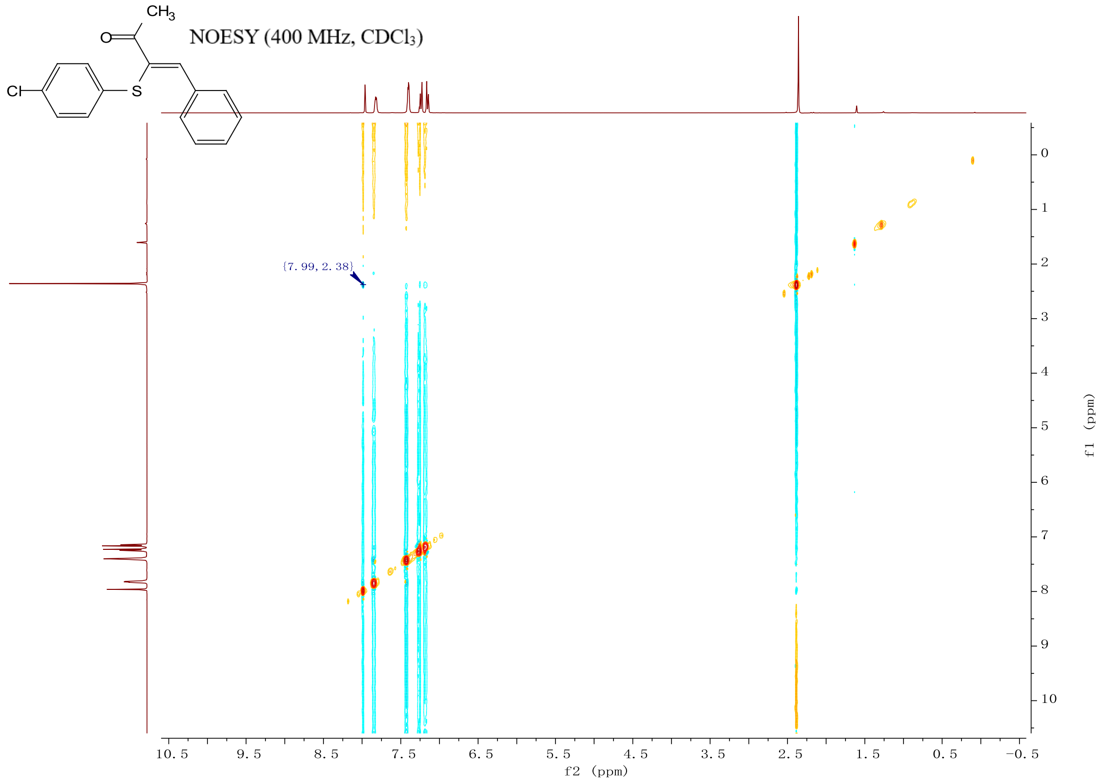

(*E*)-3-((4-chlorophenyl)thio)-4-phenylbut-3-en-2-one (*E*-5ah)

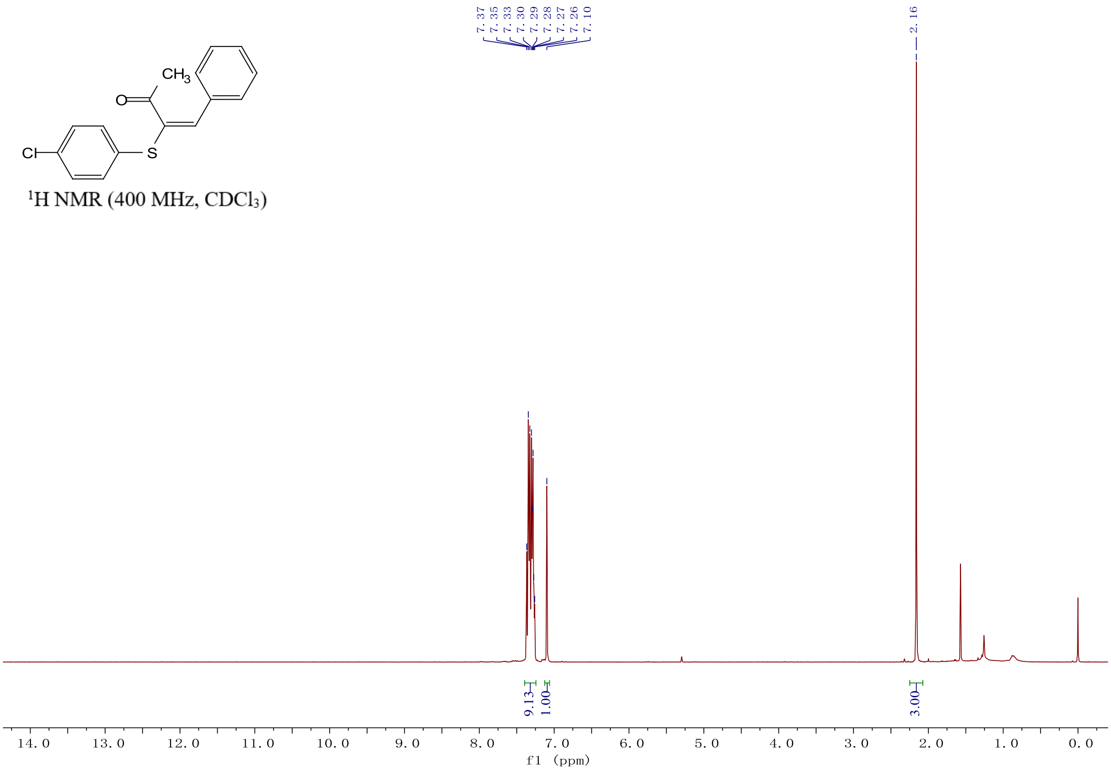

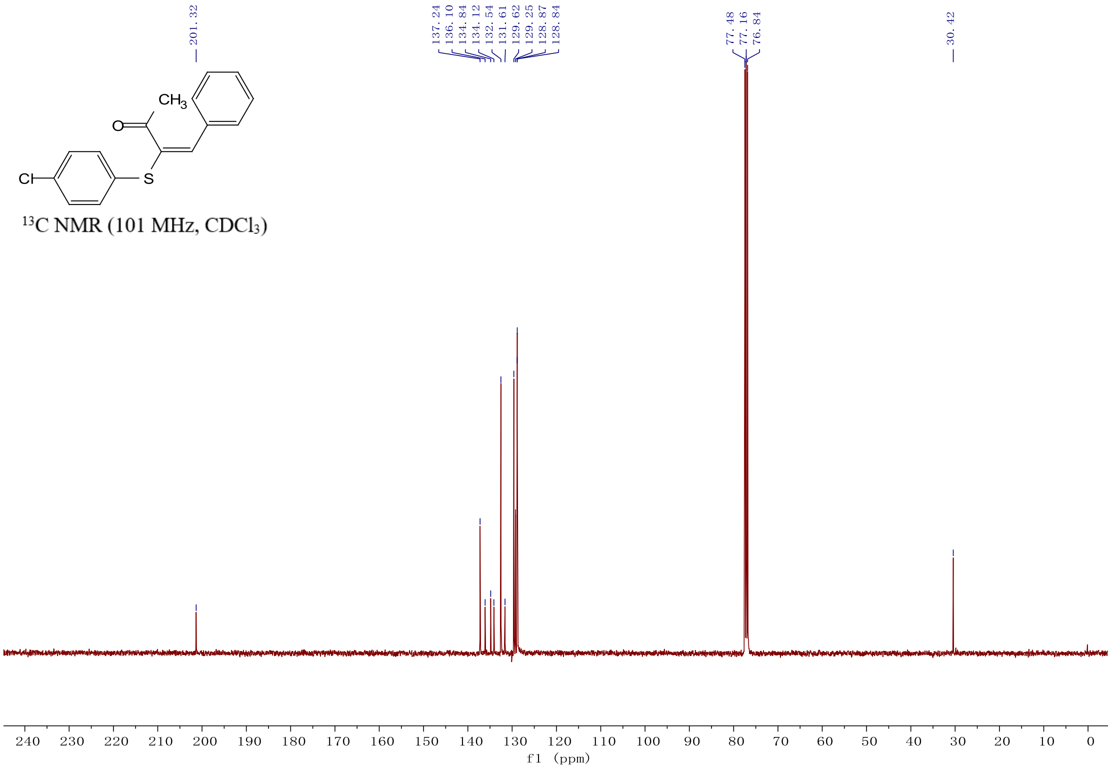

(*E*)-3-((4-chlorophenyl)thio)-4-phenylbut-3-en-2-one (*E*-5ah)

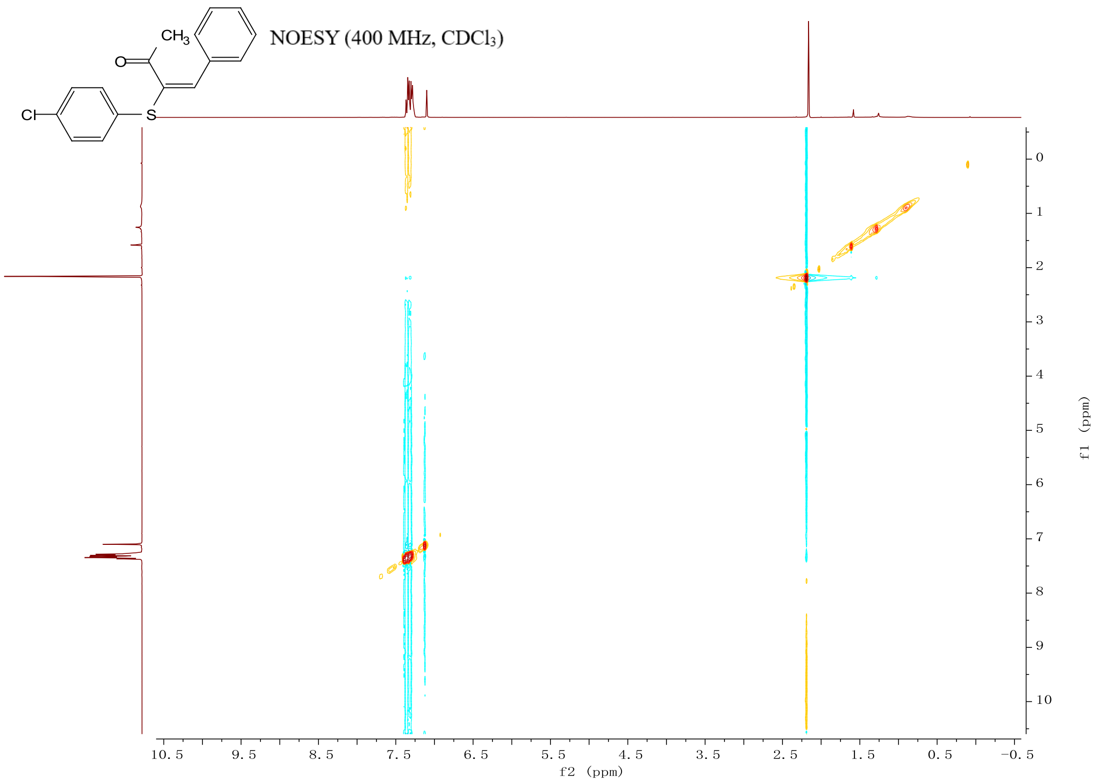

(*Z*)-1-phenyl-2-(phenylthio)but-2-en-1-one (*Z*-5ai)

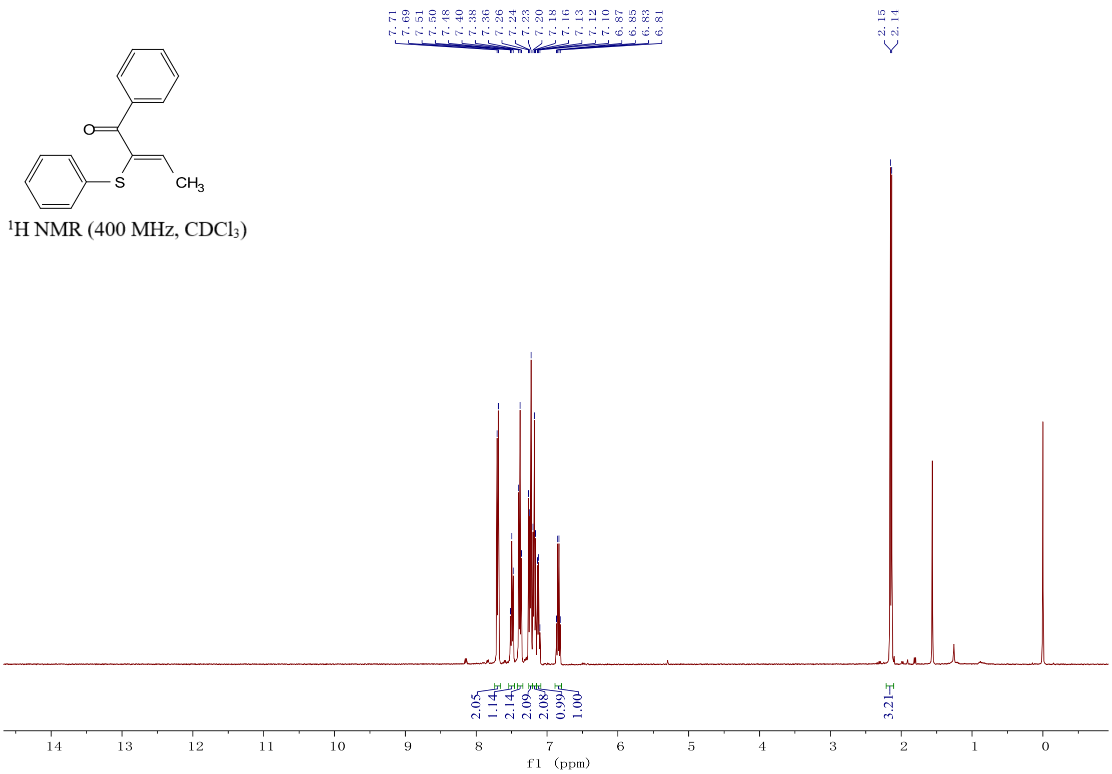

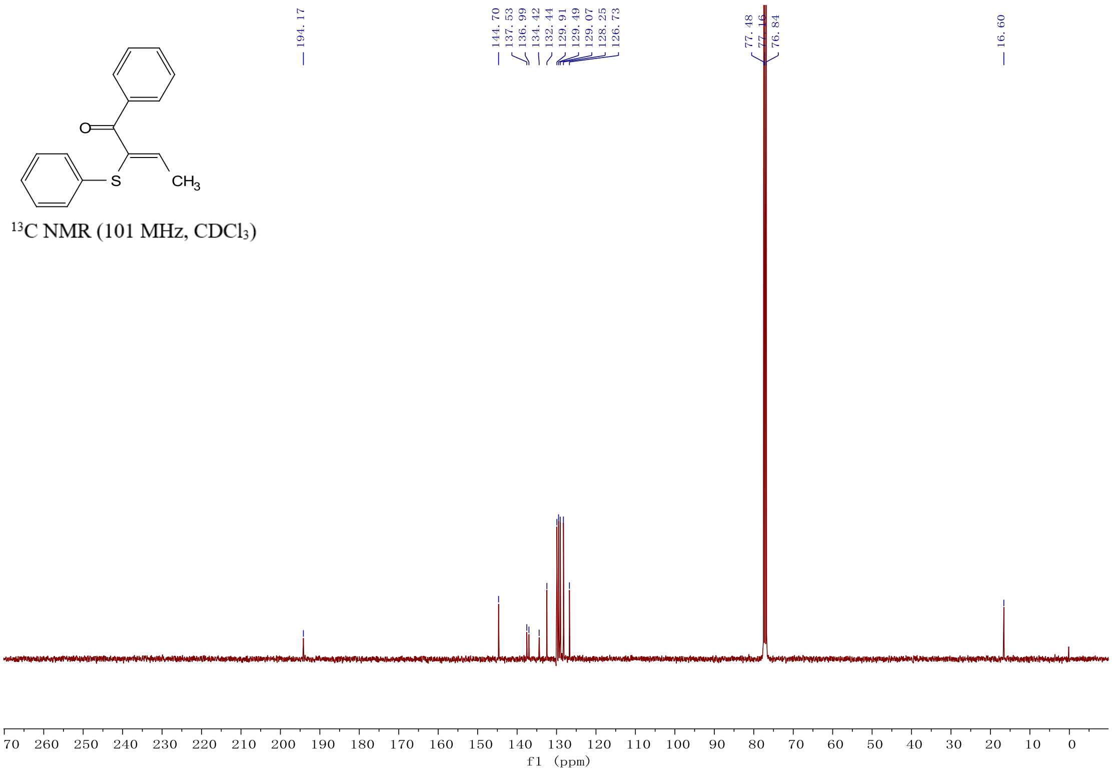

(*Z*)-2-(phenylthio)pent-2-enal (*Z*-5aj)

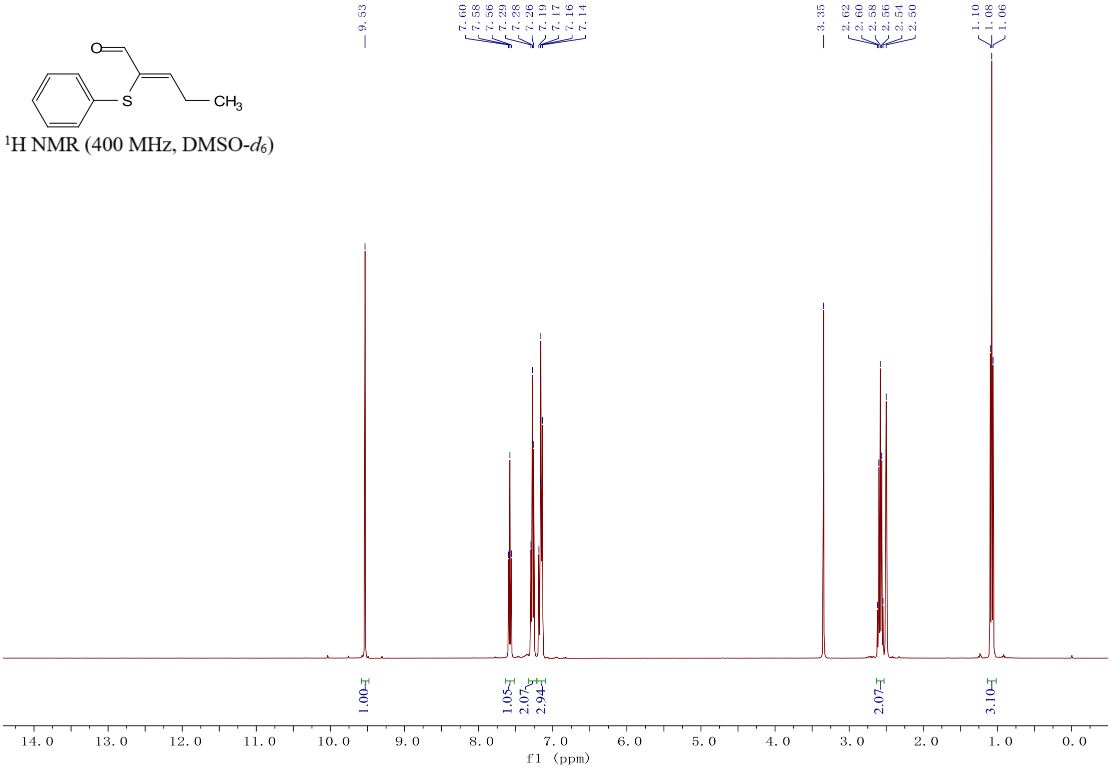

^{1}H NMR (400 MHz, DMSO-d_6)

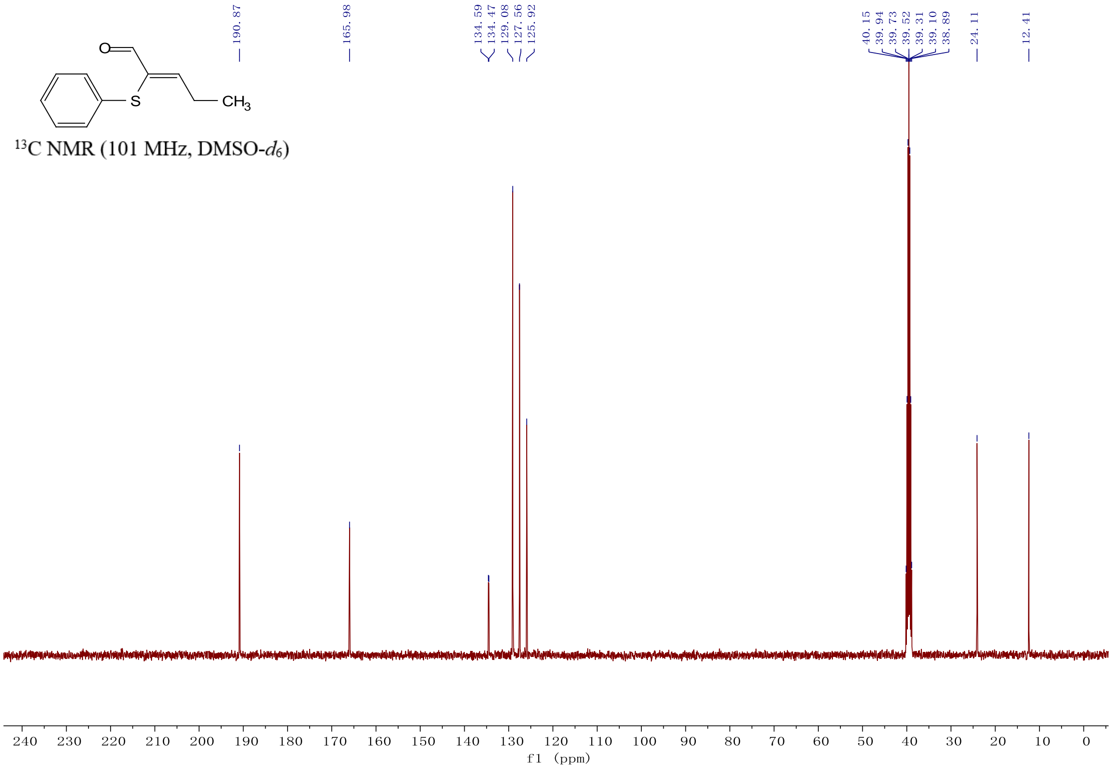

^{13}C NMR (101 MHz, DMSO-d_6)

(*Z*)-2-(phenylthio)pent-2-enal (*Z*-5aj)

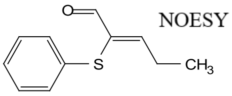

NOESY (400 MHz, DMSO-d_6)

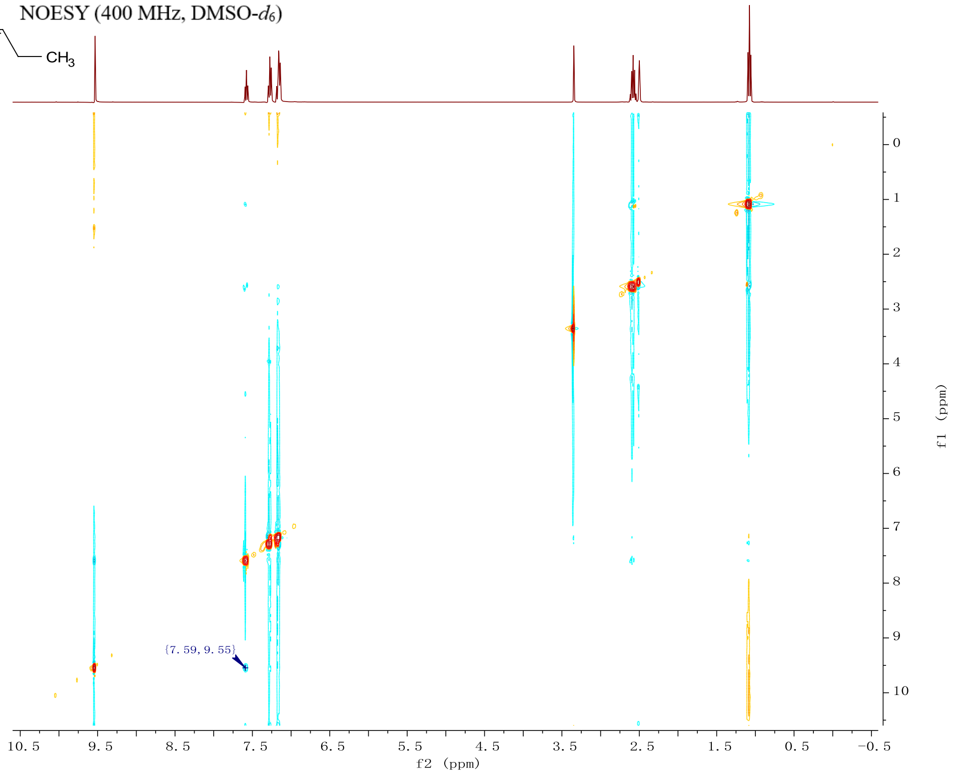

(1*Z*,2*Z*)-2-(phenylthio)pent-2-enal oxime/(1*E*,2*Z*)-2-(phenylthio)pent-2-enal oxime (*1Z,2Z*-**5ak**/*1E,2Z*-**5ak** = 0.4/1)

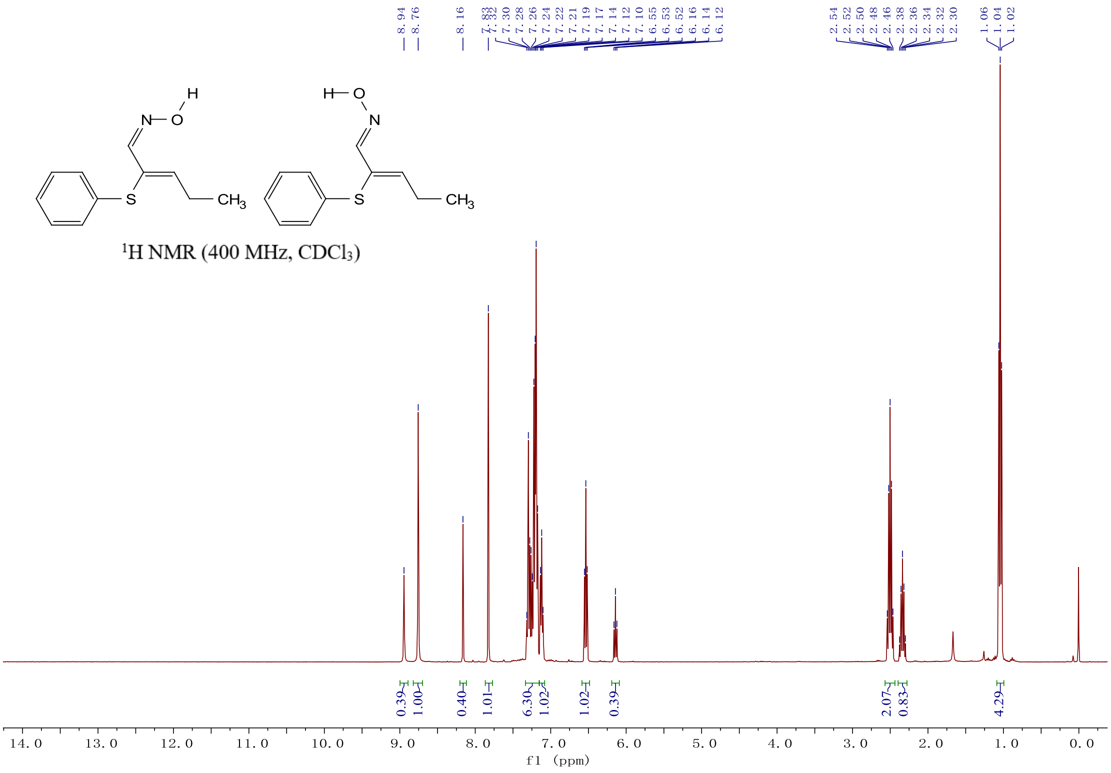

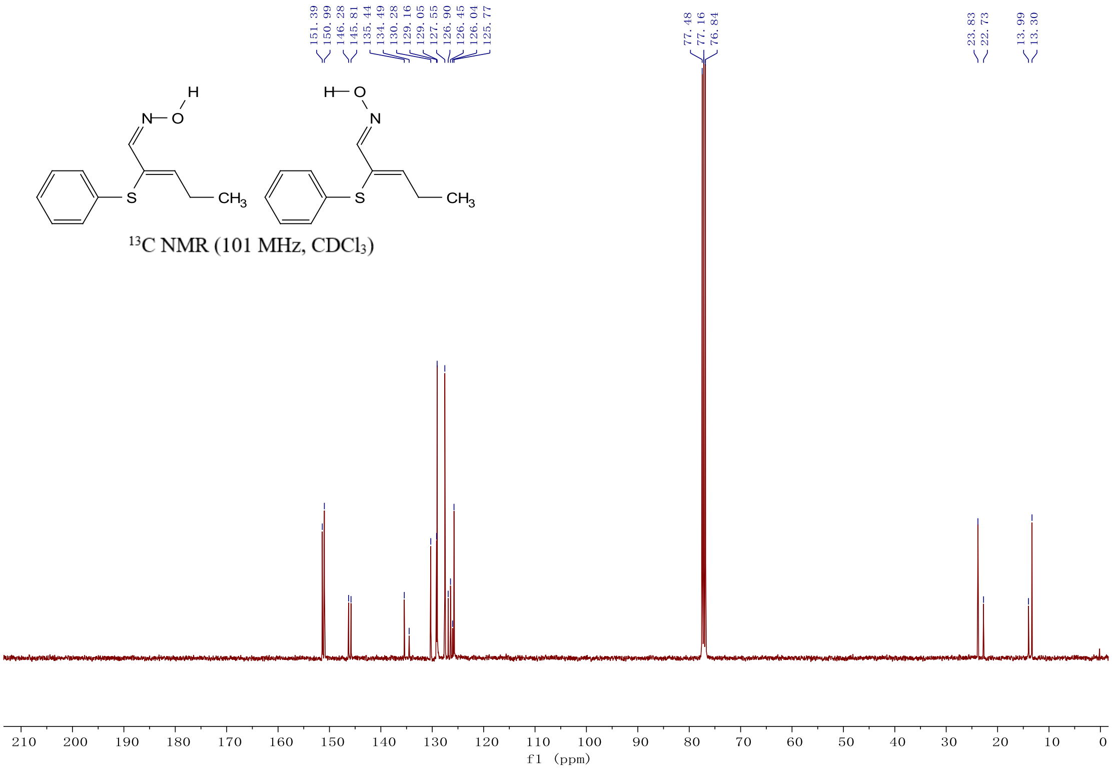

(1*E*,2*Z*)-2-(phenylthio)pent-2-enal oxime (*1Z,2Z*-5ak)

NOESY (400 MHz, $CDCl_3$)

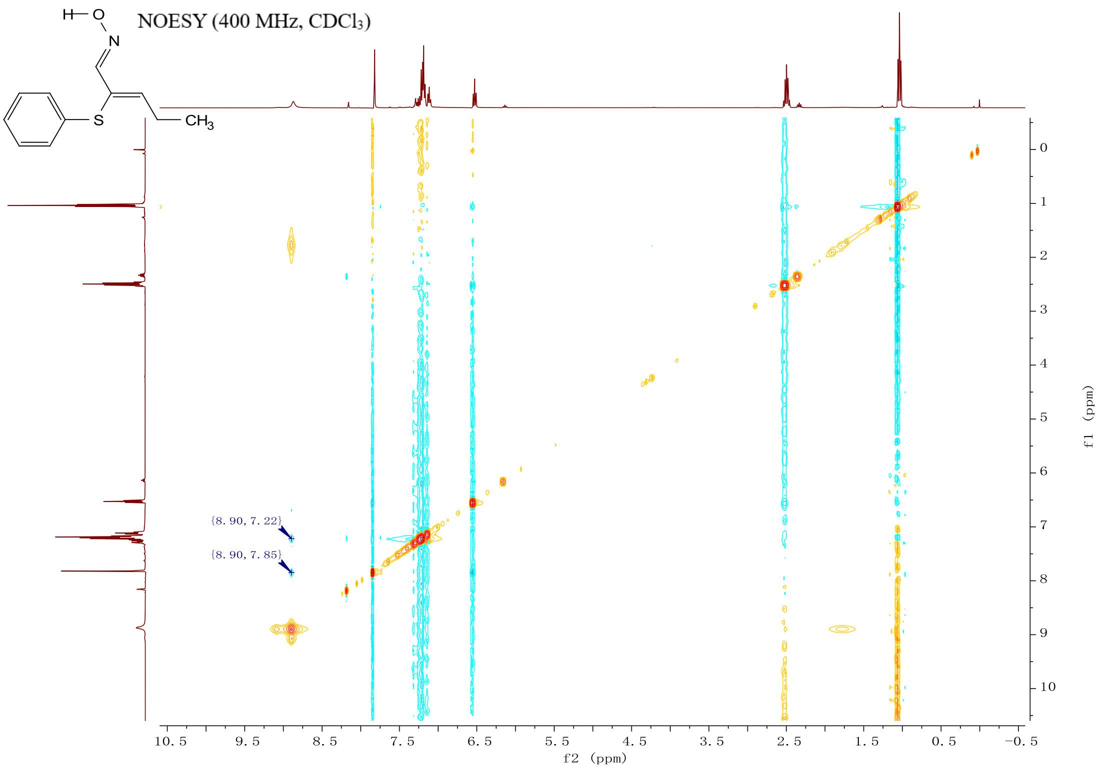

(*Z*)-2-(phenylthio)pent-2-enenitrile (*Z*-5al)

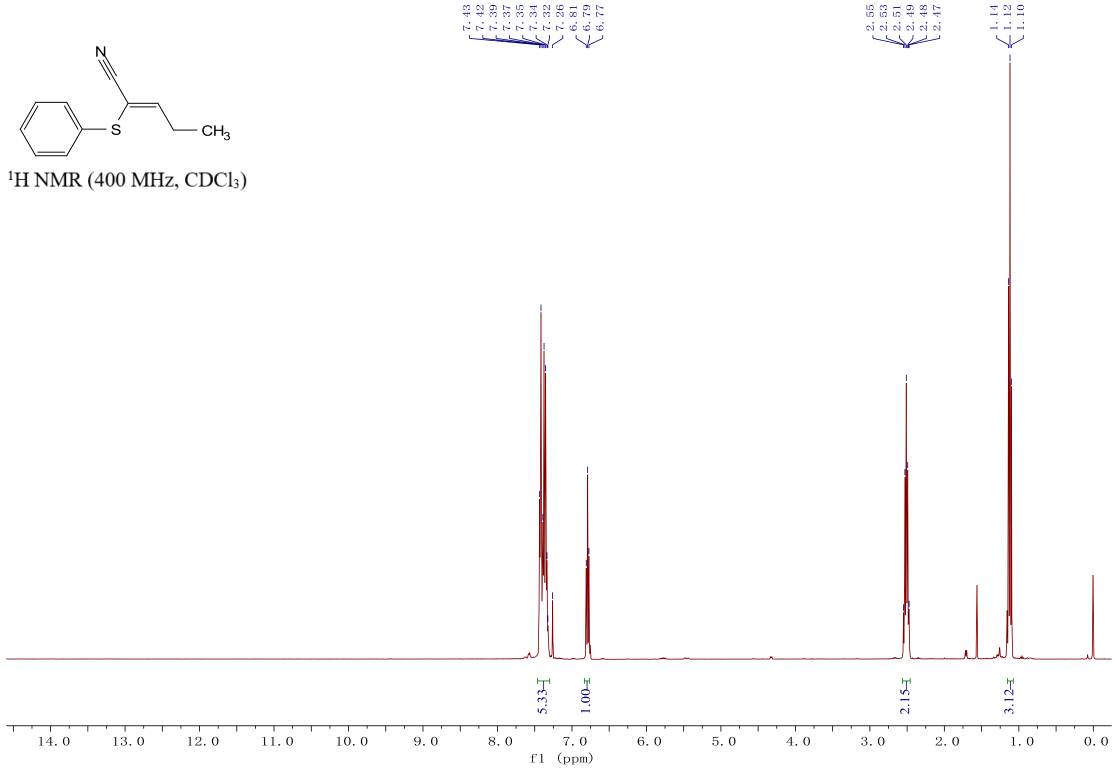

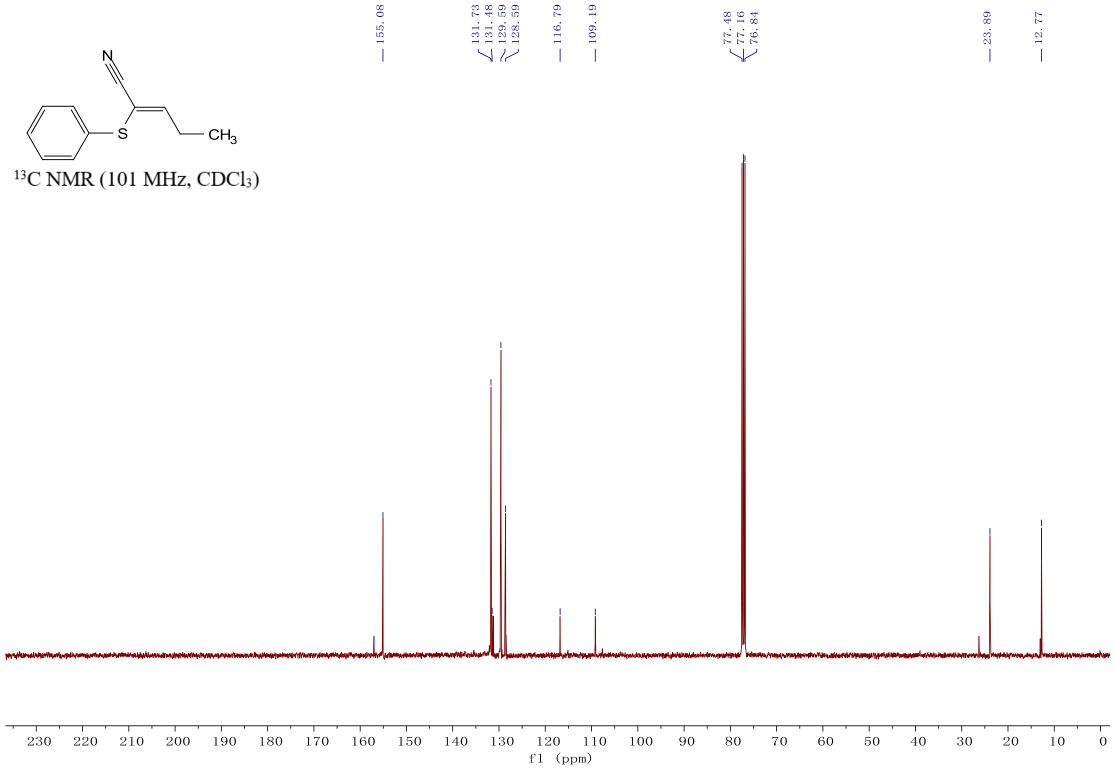

(*E*)-2-(phenylthio)pent-2-enenitrile (*E*-5al)

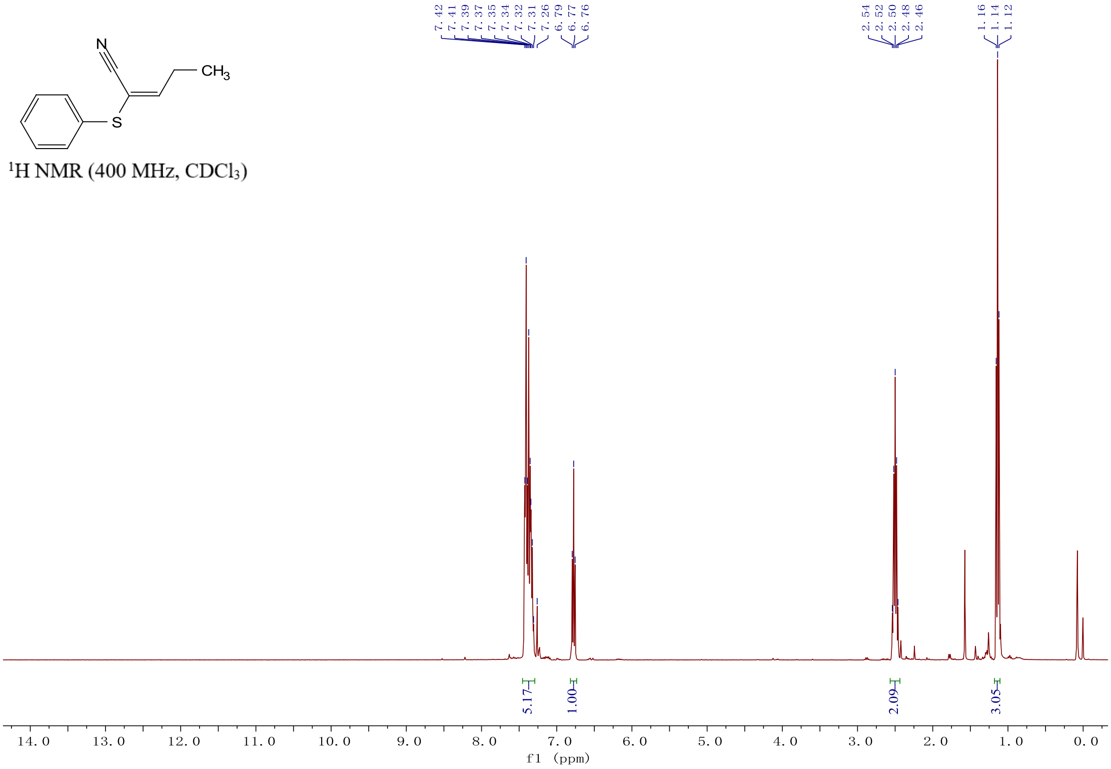

^{1}H NMR (400 MHz, $CDCl_3$)

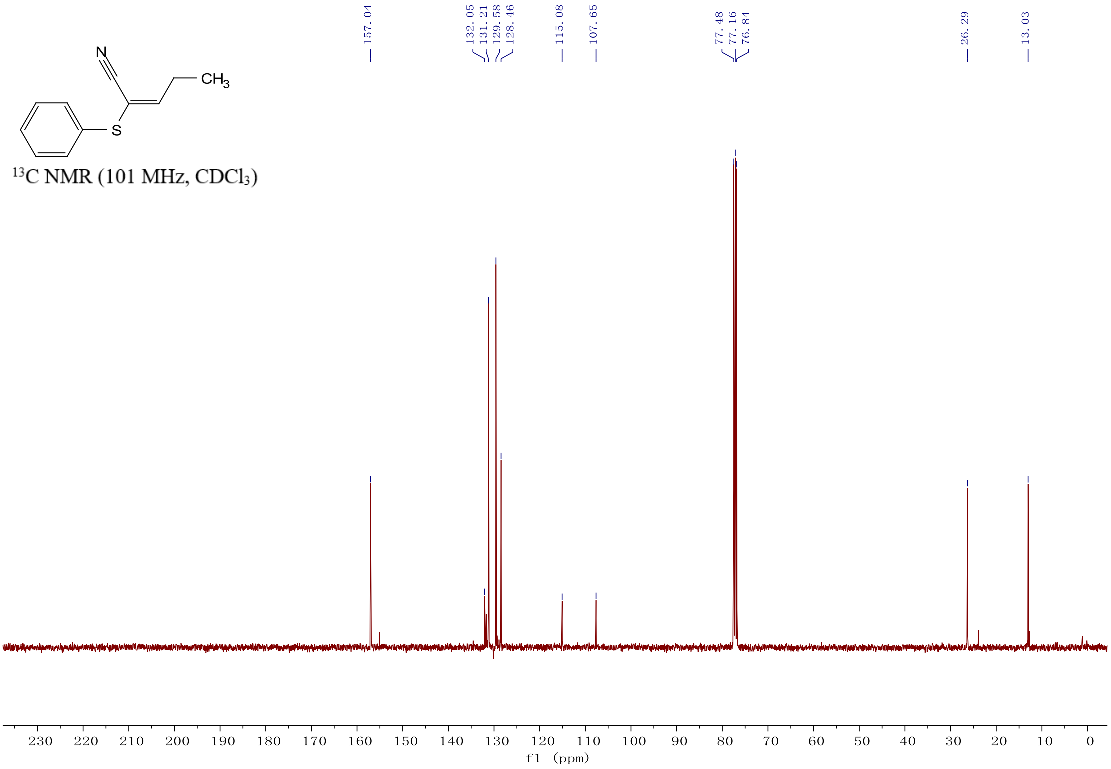

^{13}C NMR (101 MHz, $CDCl_3$)

methyl (*Z*)-2-(phenylthio)but-2-enoate (*Z*-5am)

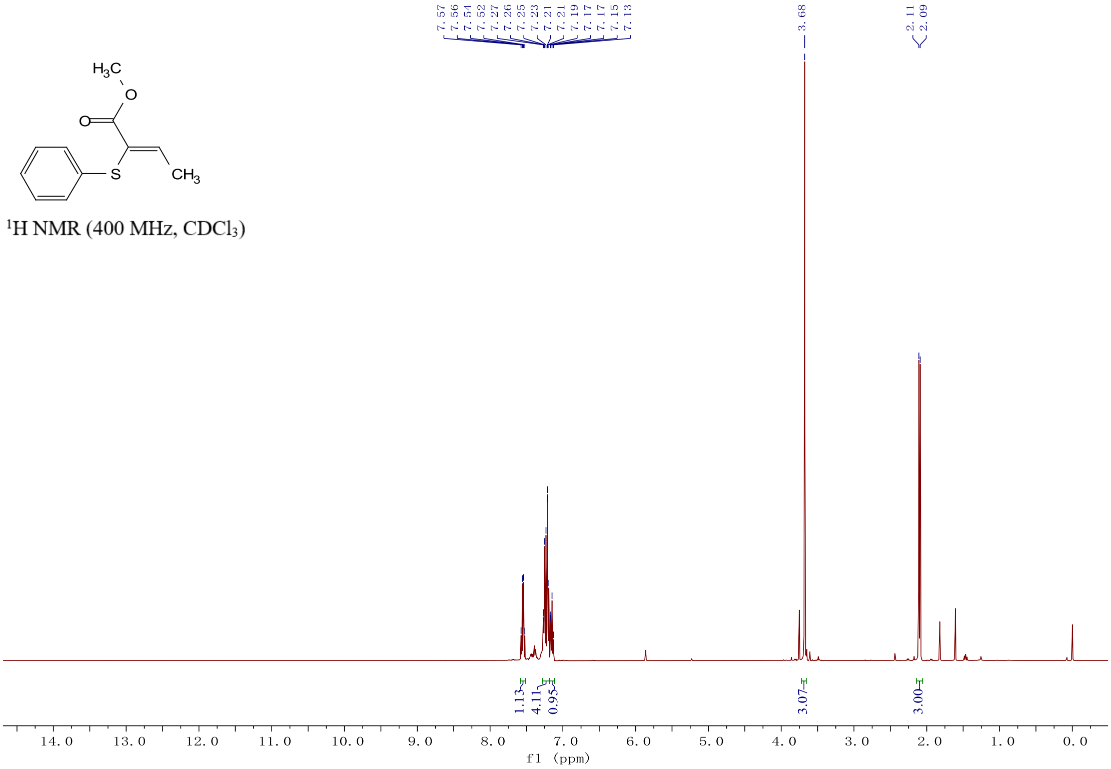

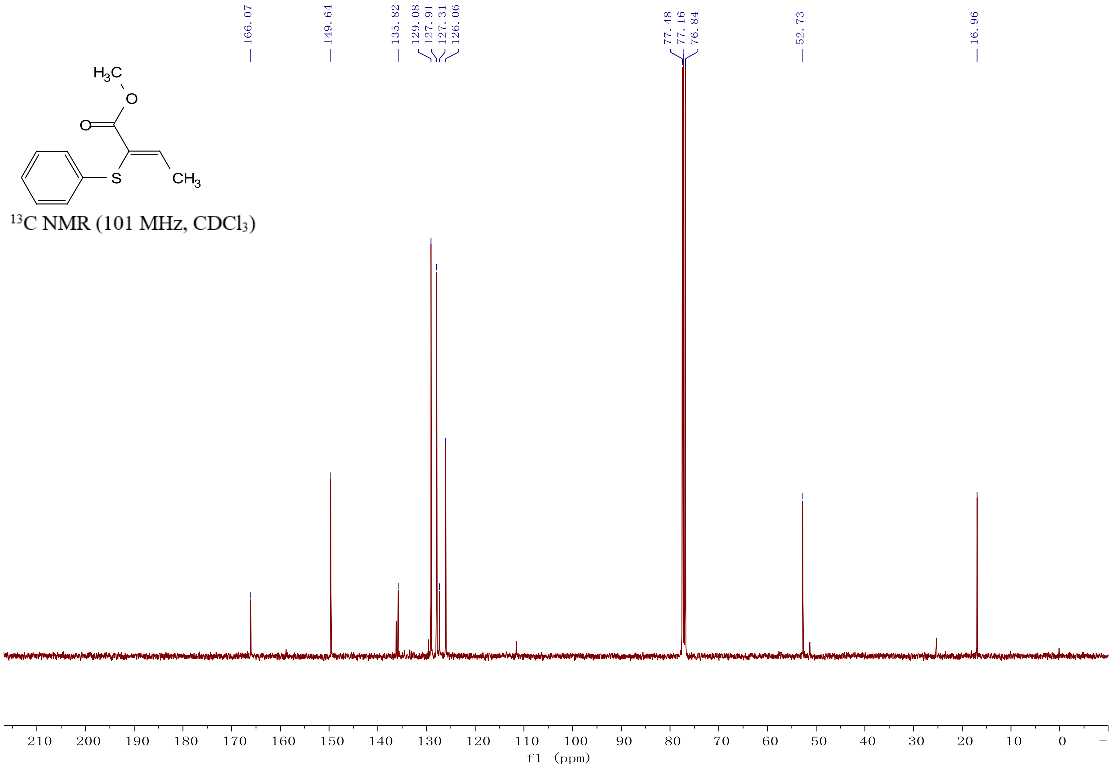

3-(phenylthio)hexan-2-one (7aa)

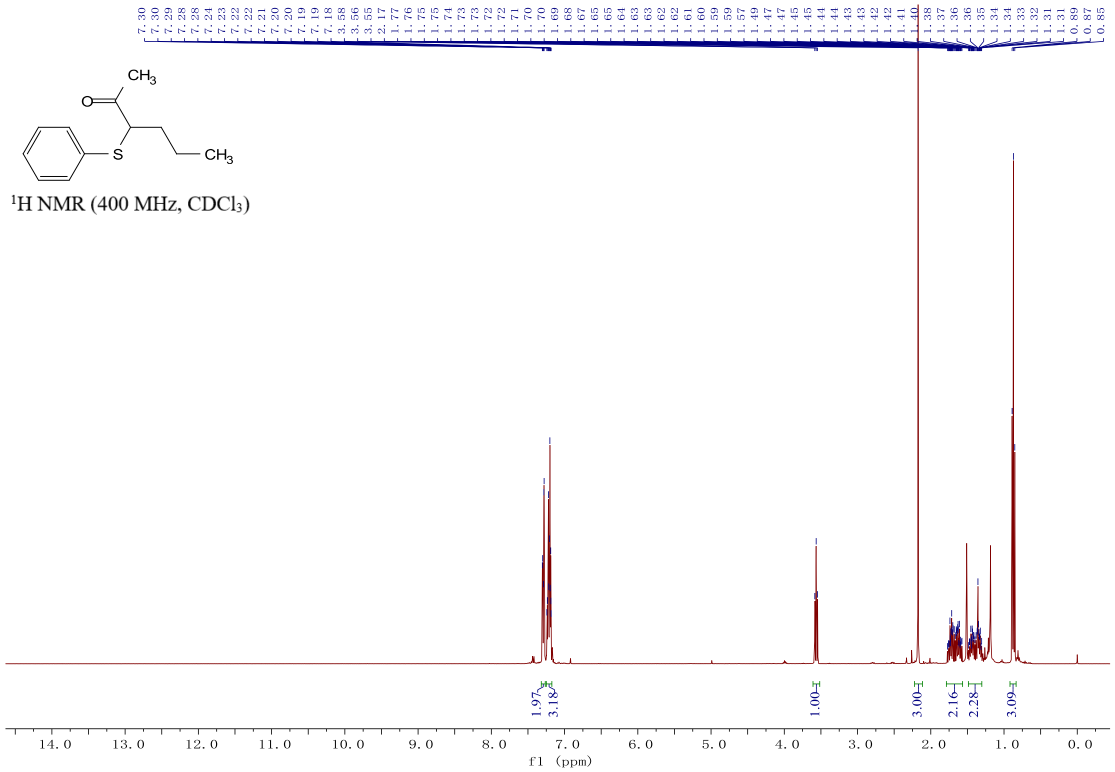

^{1}H NMR (400 MHz, $CDCl_3$)

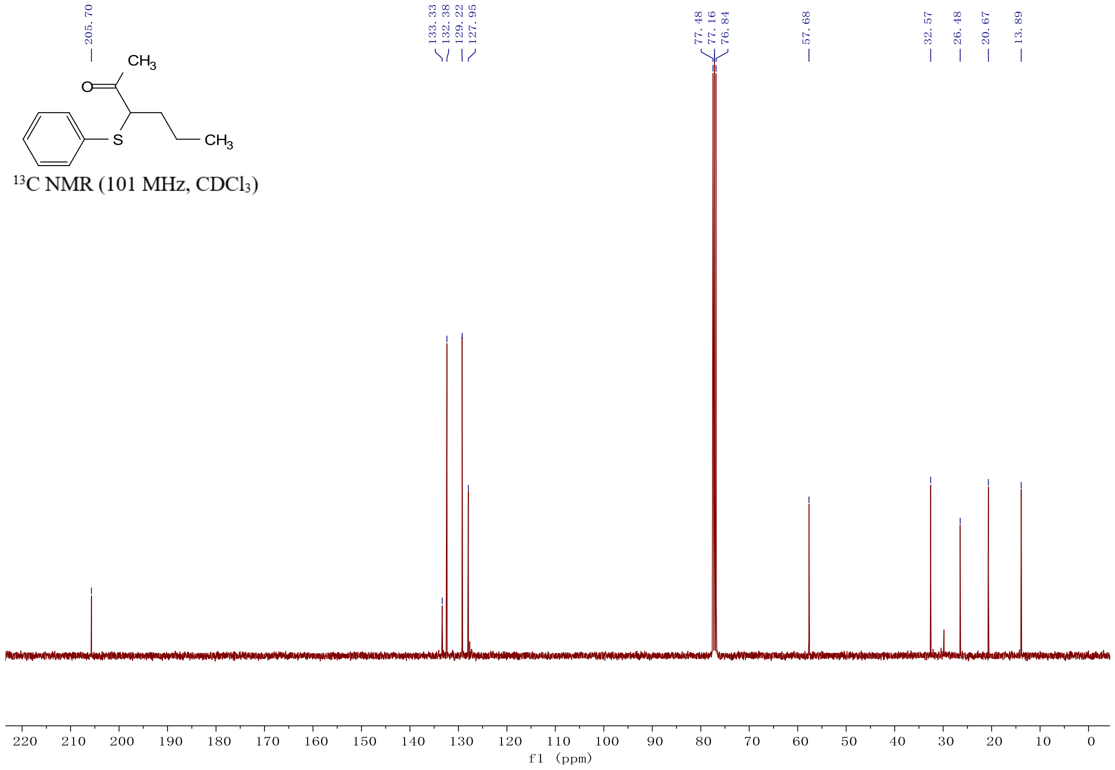

^{13}C NMR (101 MHz, $CDCl_3$)

3-((2-fluorophenyl)thio)hexan-2-one (7ab)

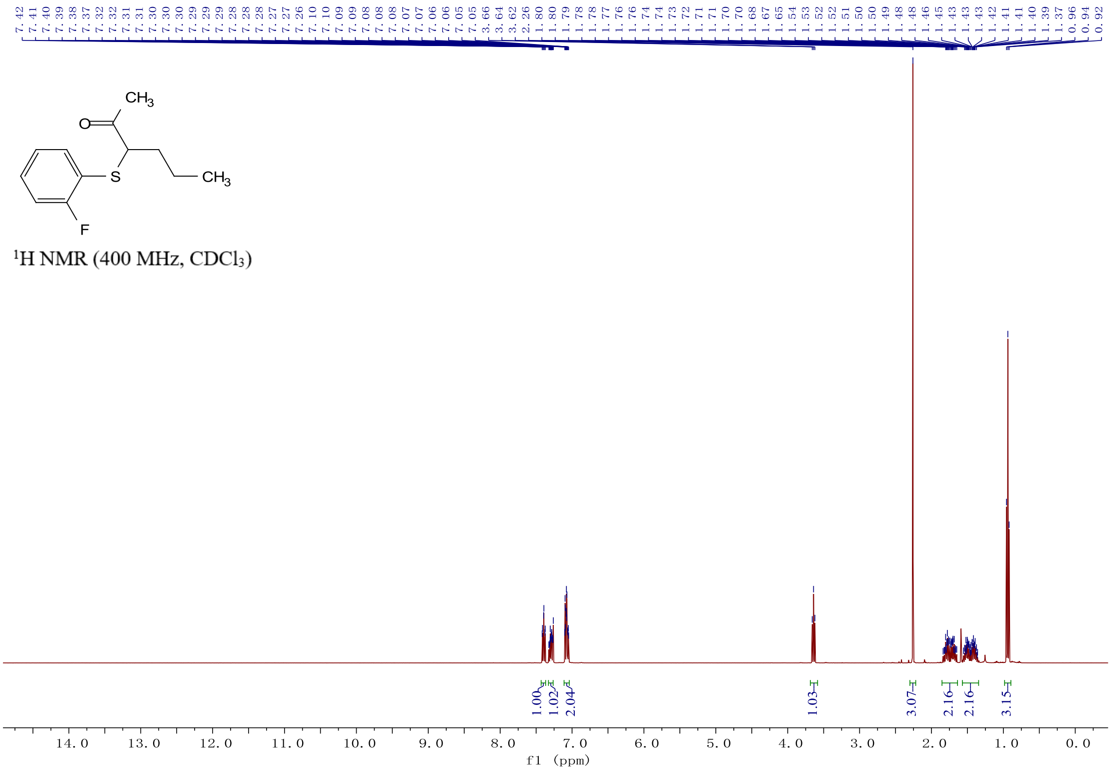

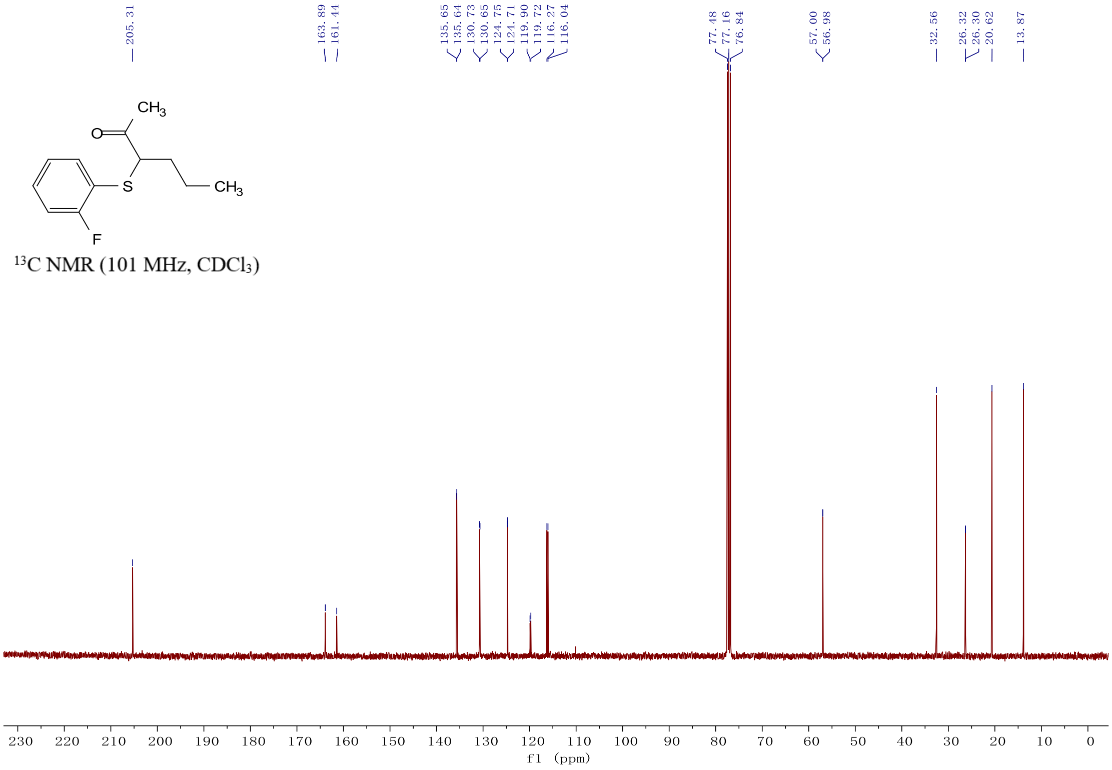

3-((2-fluorophenyl)thio)hexan-2-one (7ab)

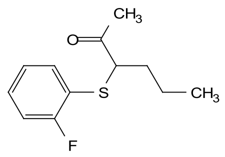

^{19}F NMR (376 MHz, $CDCl_3$)

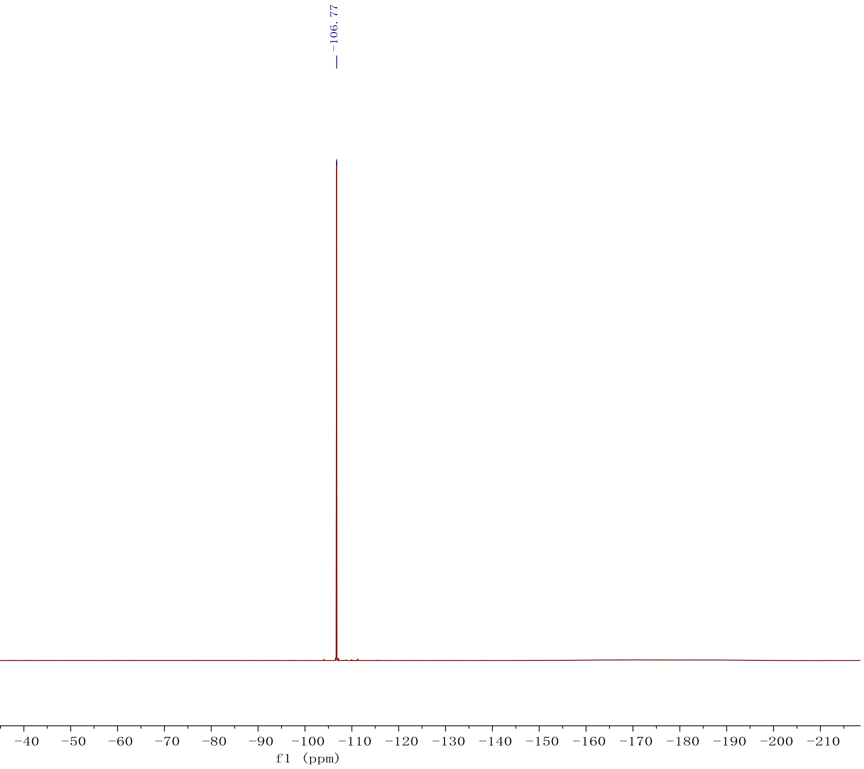

3-((2-chlorophenyl)thio)hexan-2-one (7ac)

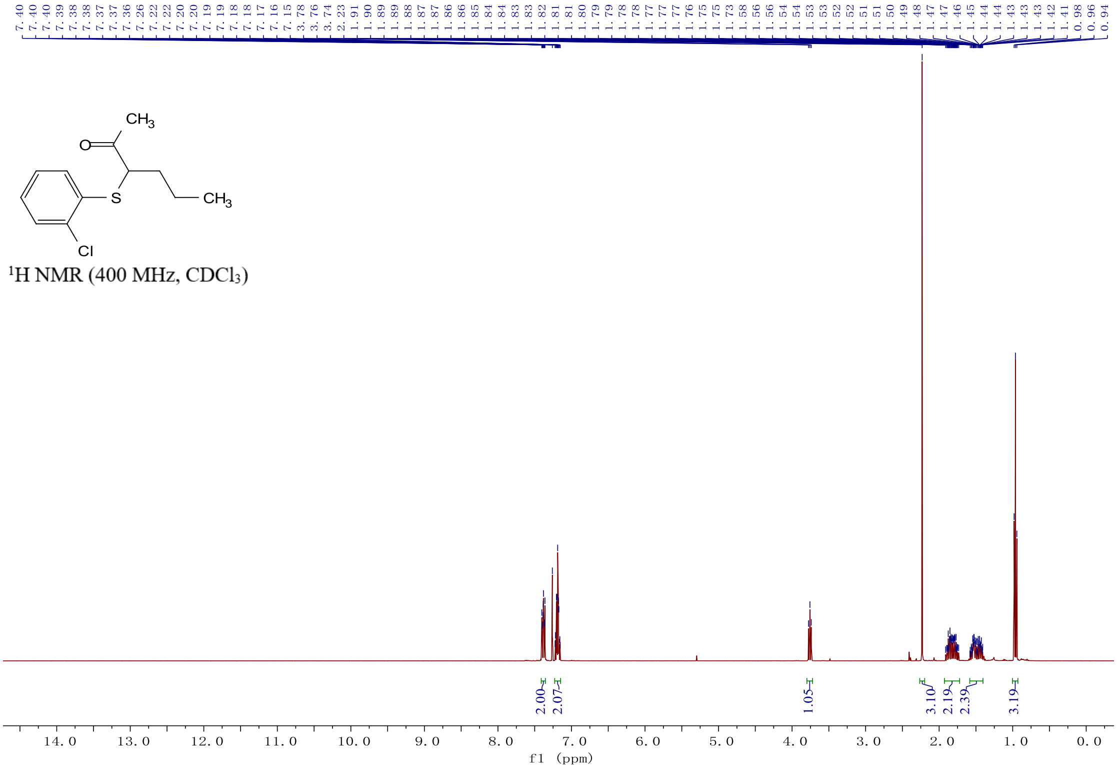

^{1}H NMR (400 MHz, $CDCl_3$)

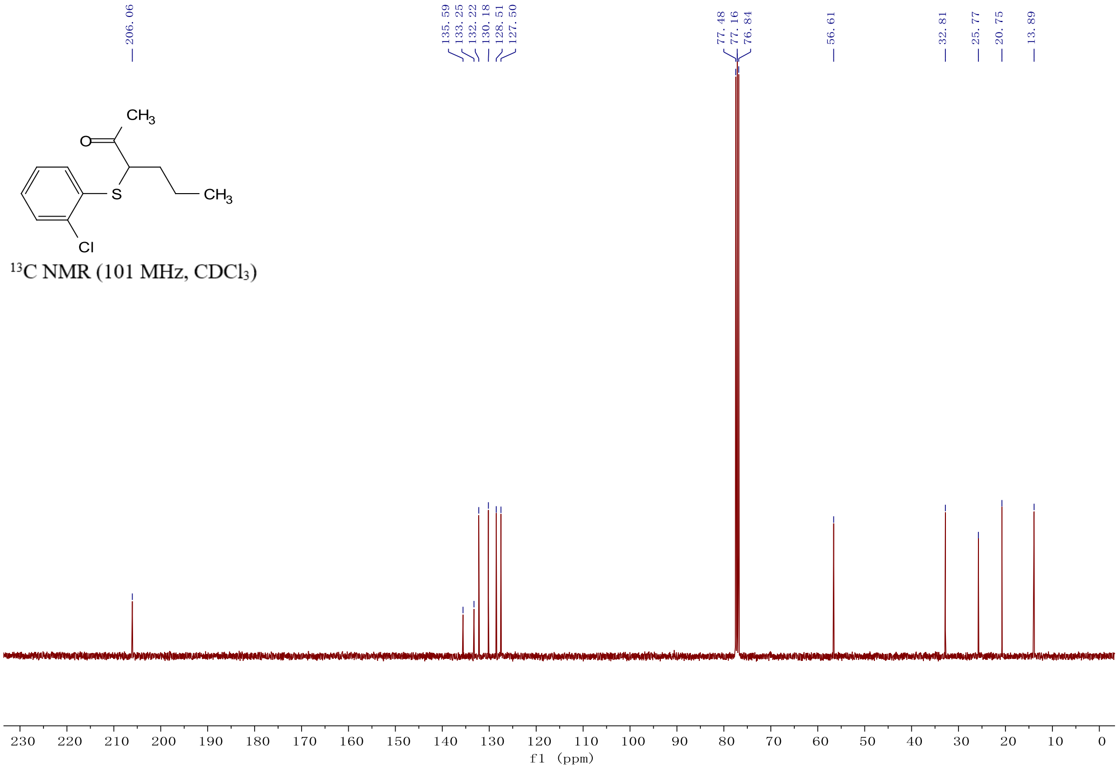

^{13}C NMR (101 MHz, $CDCl_3$)

3-((4-chlorophenyl)thio)hexan-2-one (7ad)

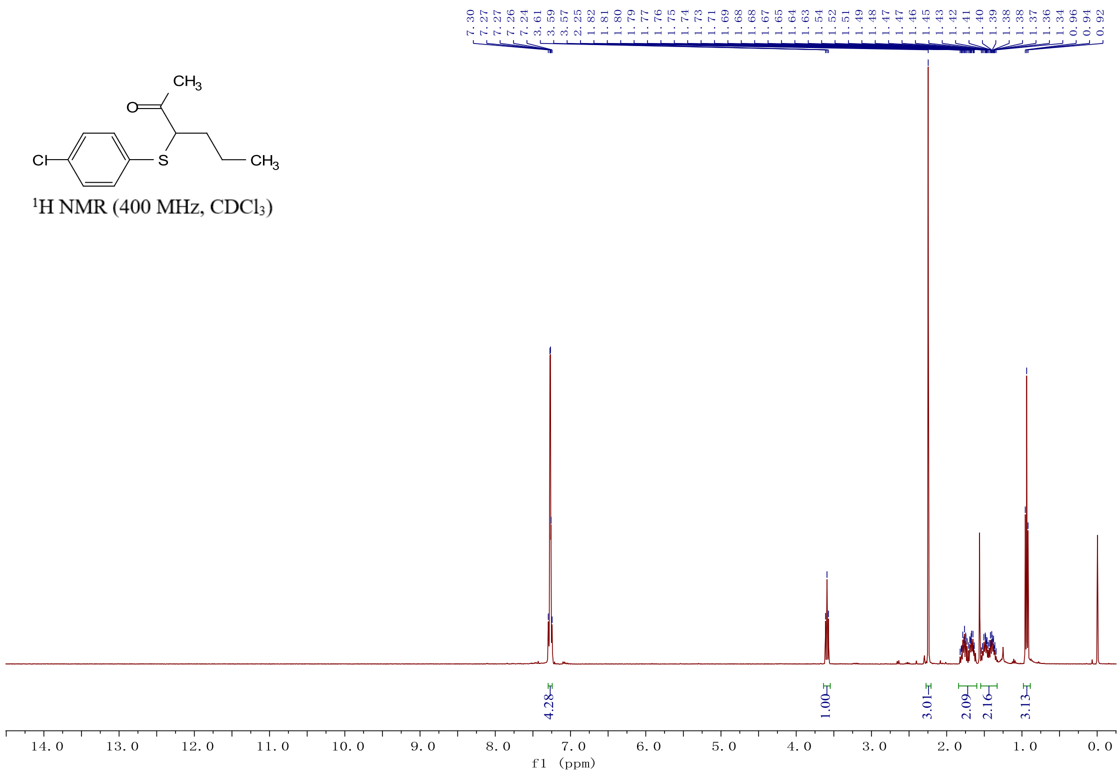

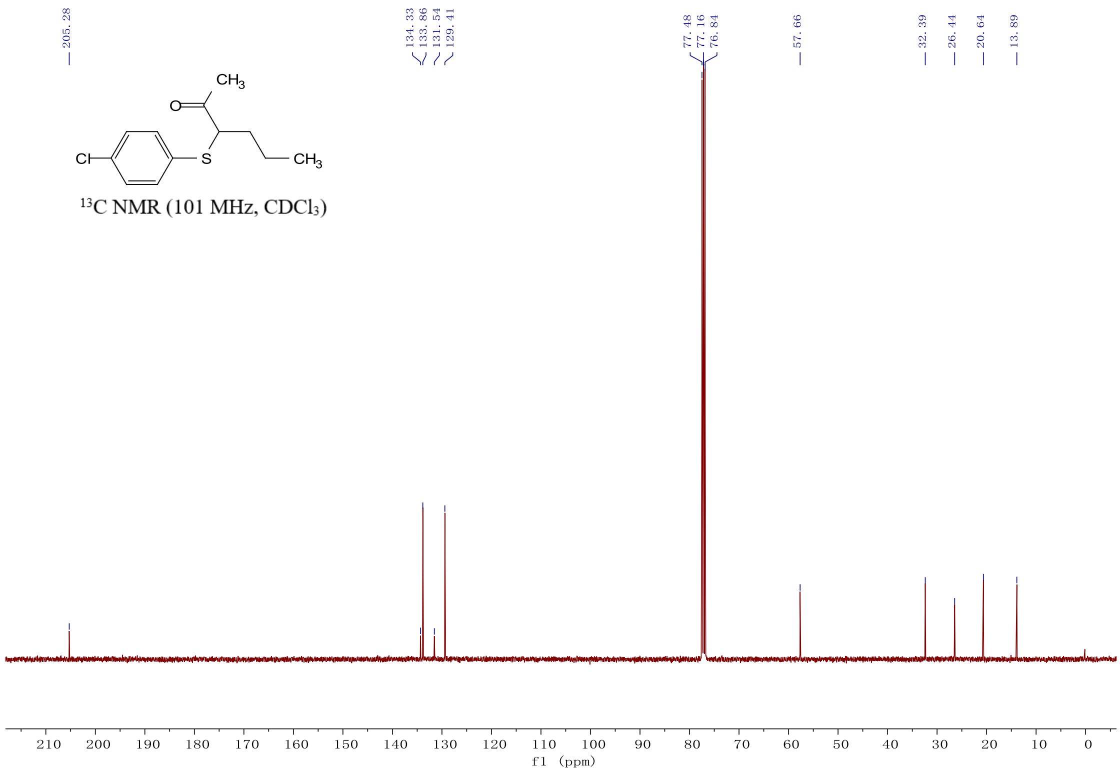

3-((2-bromophenyl)thio)hexan-2-one (7ae)

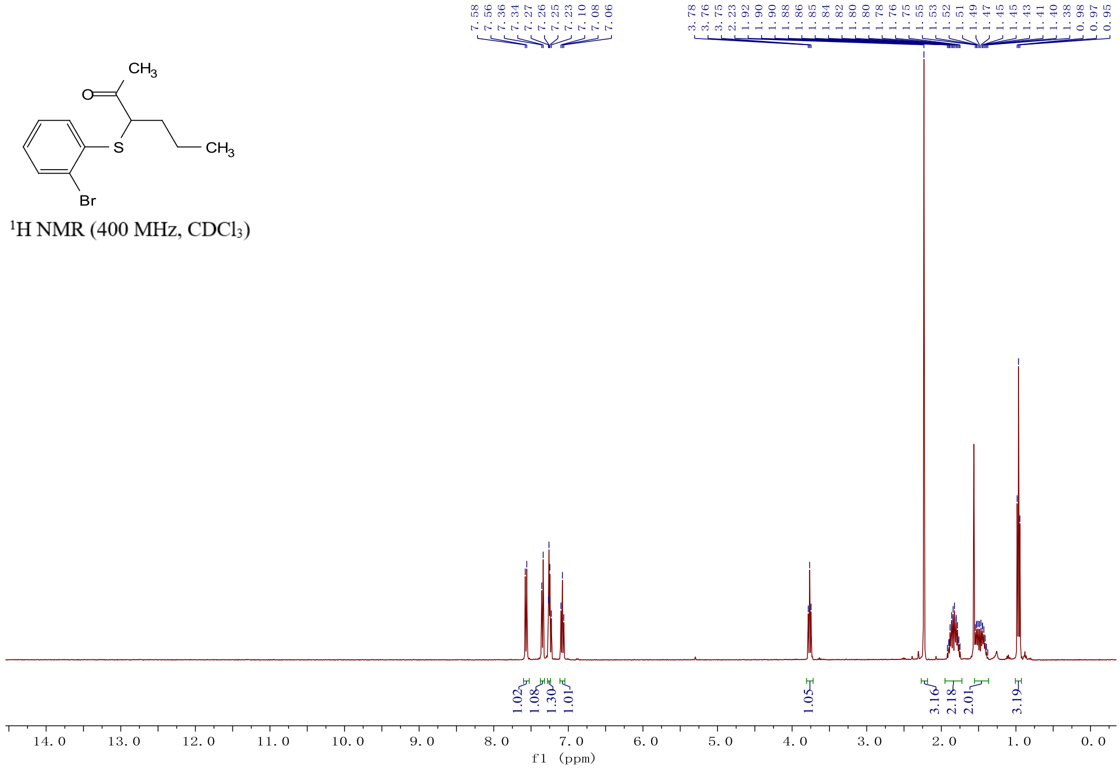

^{1}H NMR (400 MHz, CDCl$_3$)

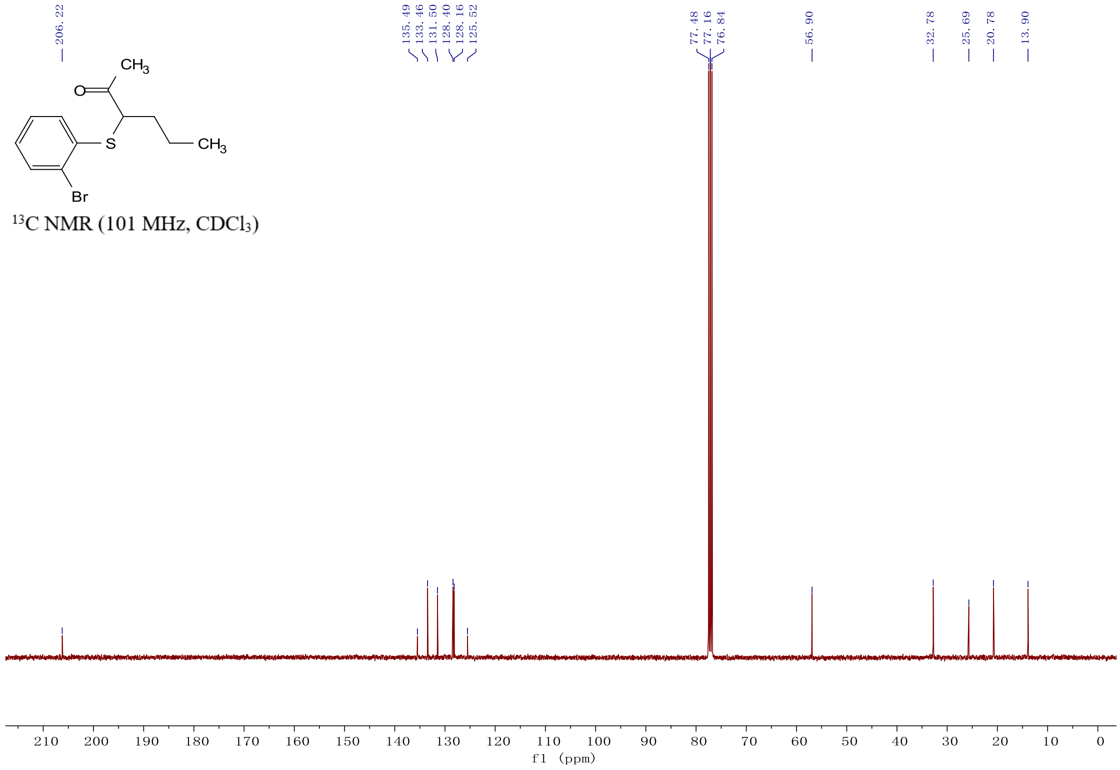

^{13}C NMR (101 MHz, CDCl$_3$)

3-((4-bromophenyl)thio)hexan-2-one (7af)

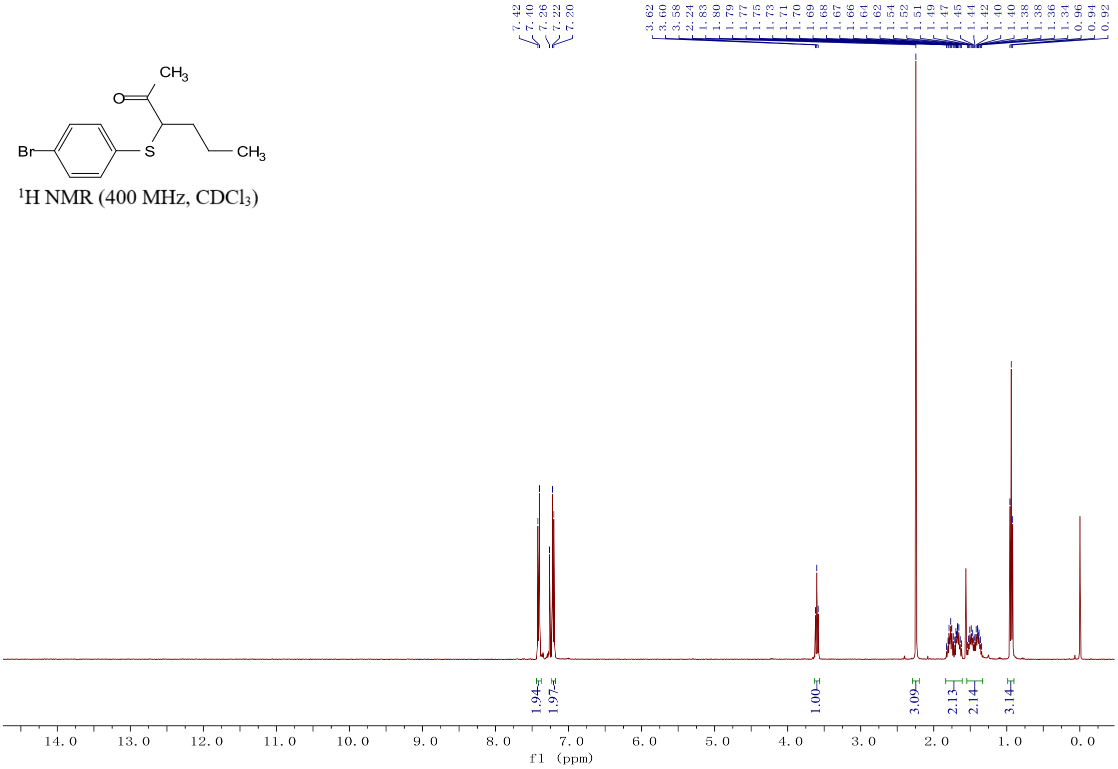

^{1}H NMR (400 MHz, $CDCl_3$)

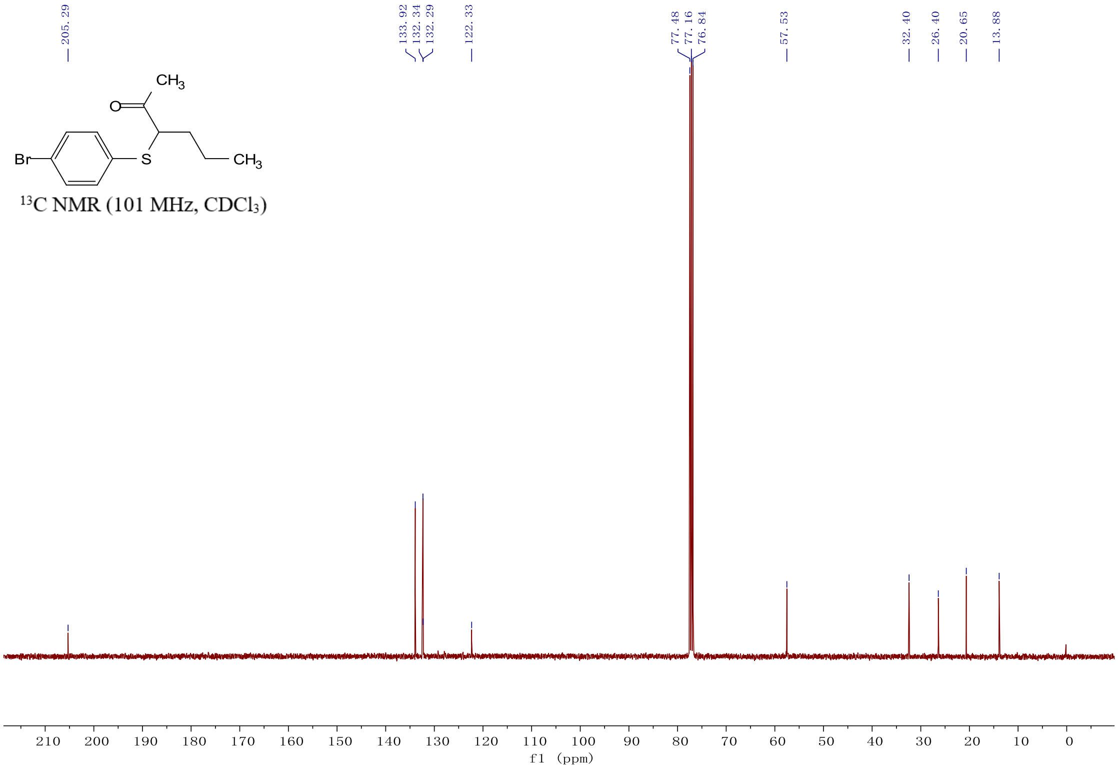

^{13}C NMR (101 MHz, $CDCl_3$)

4-phenyl-3-(phenylthio)butan-2-one (7ag)

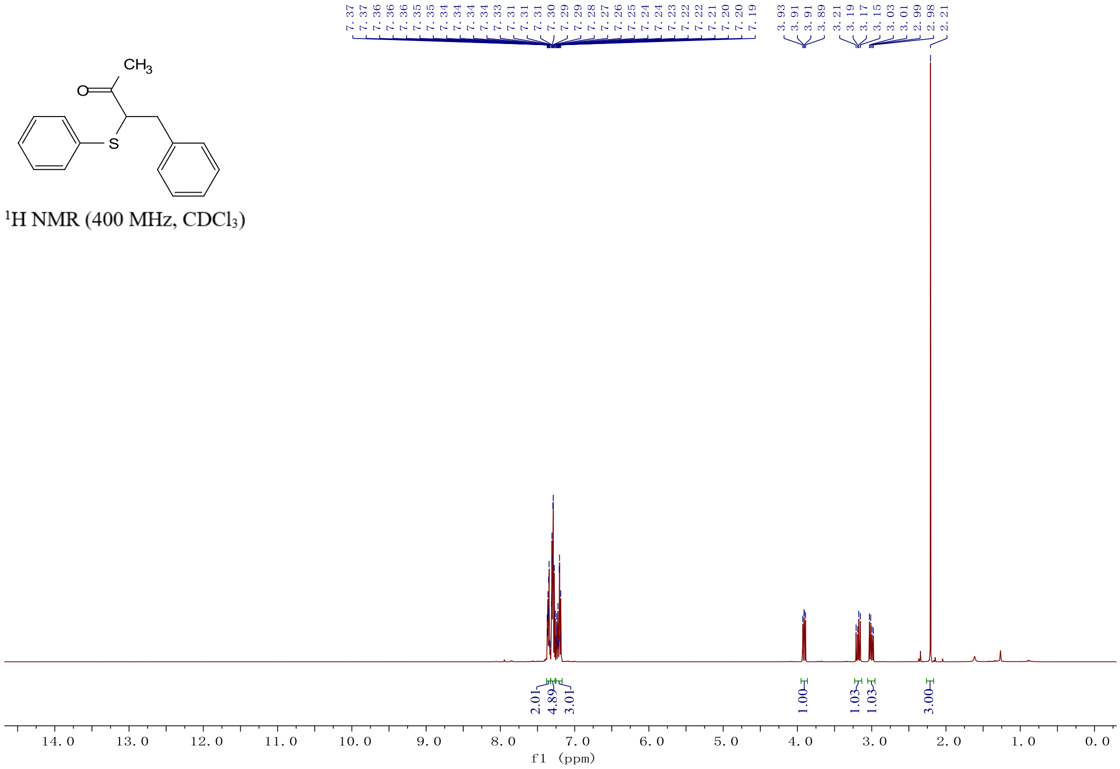

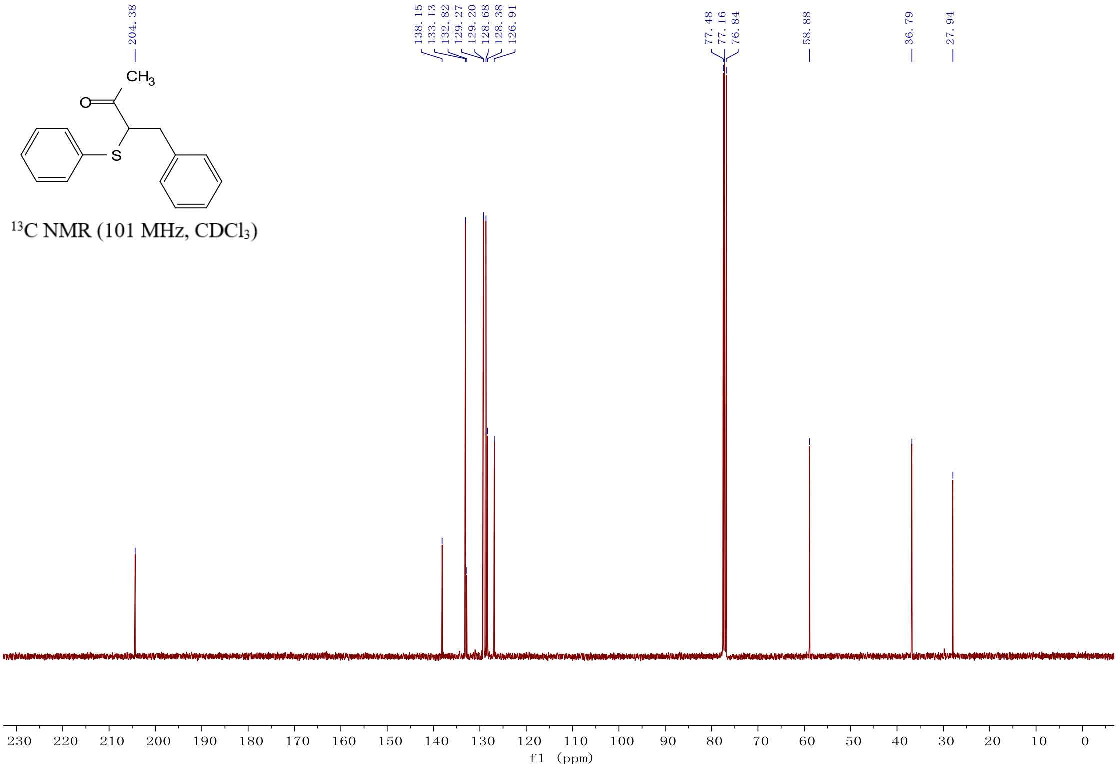

3-((4-chlorophenyl)thio)-4-phenylbutan-2-one (7ah)

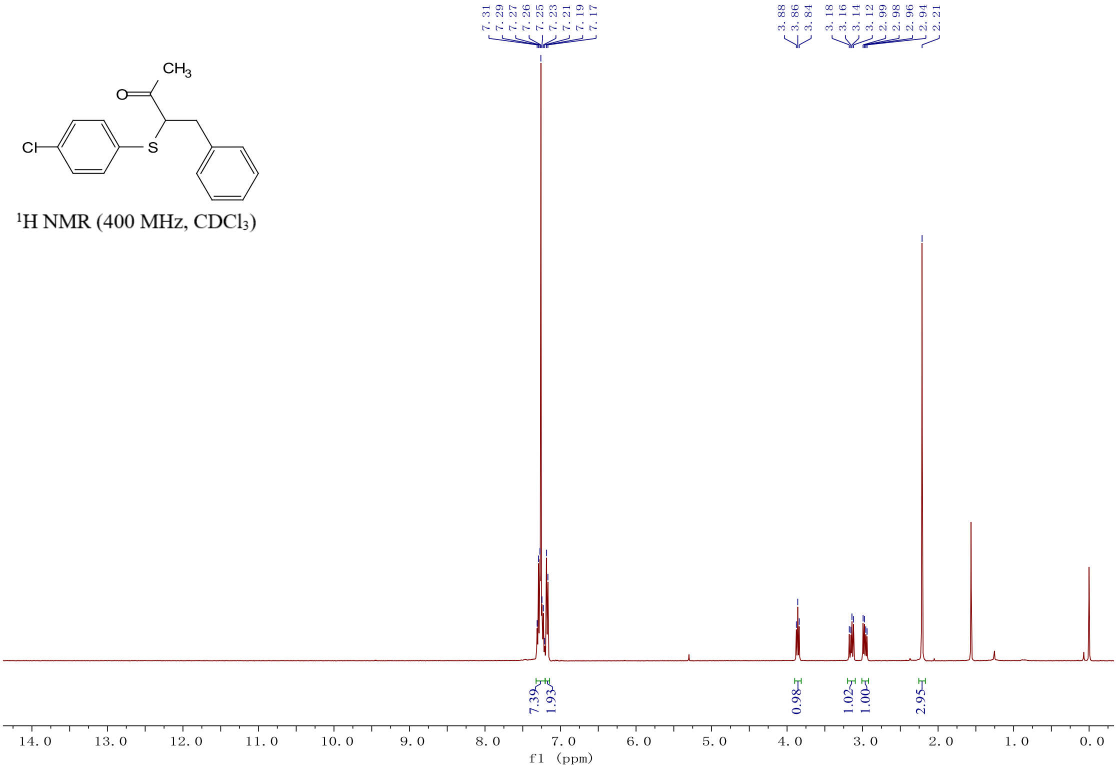

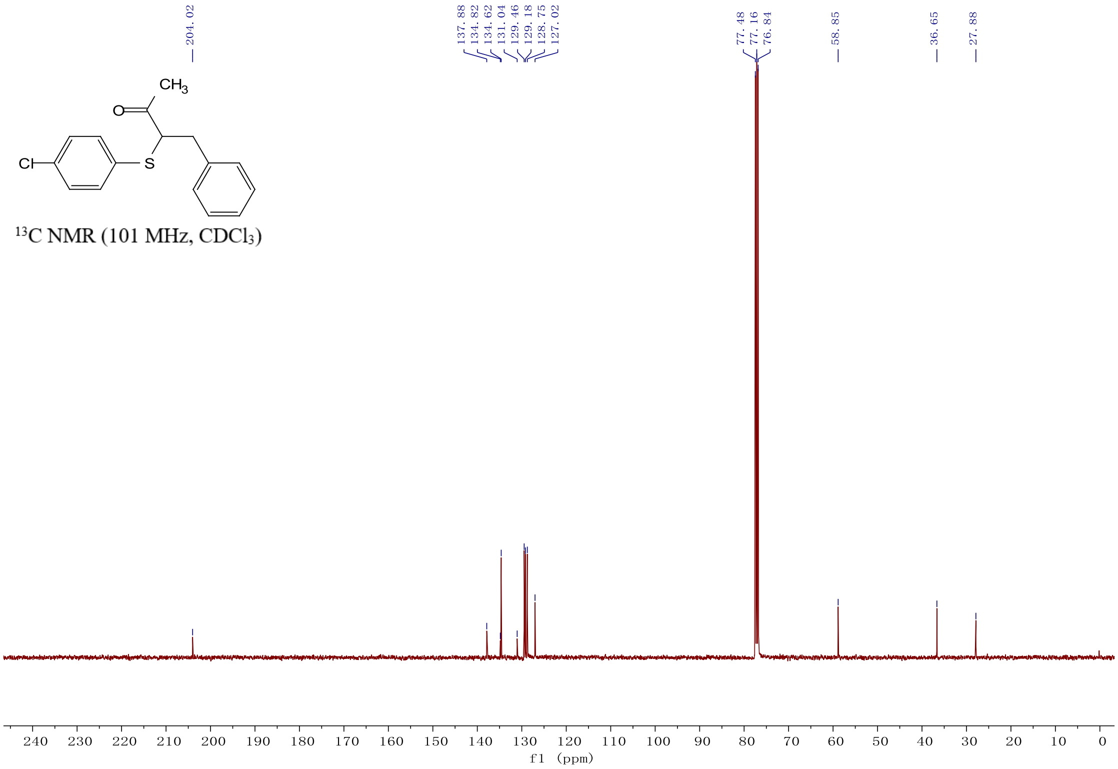

1-phenyl-2-(phenylthio)butan-1-one (7ai)

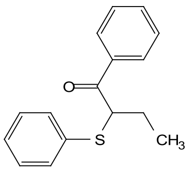

^{1}H NMR (400 MHz, $CDCl_3$)

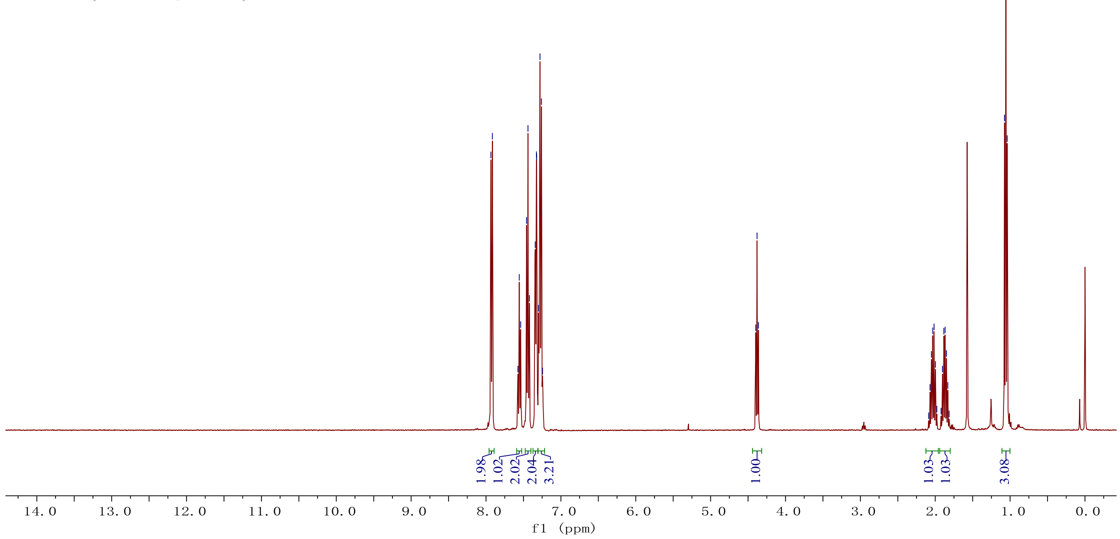

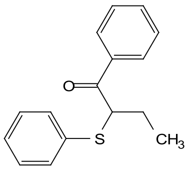

^{13}C NMR (101 MHz, $CDCl_3$)

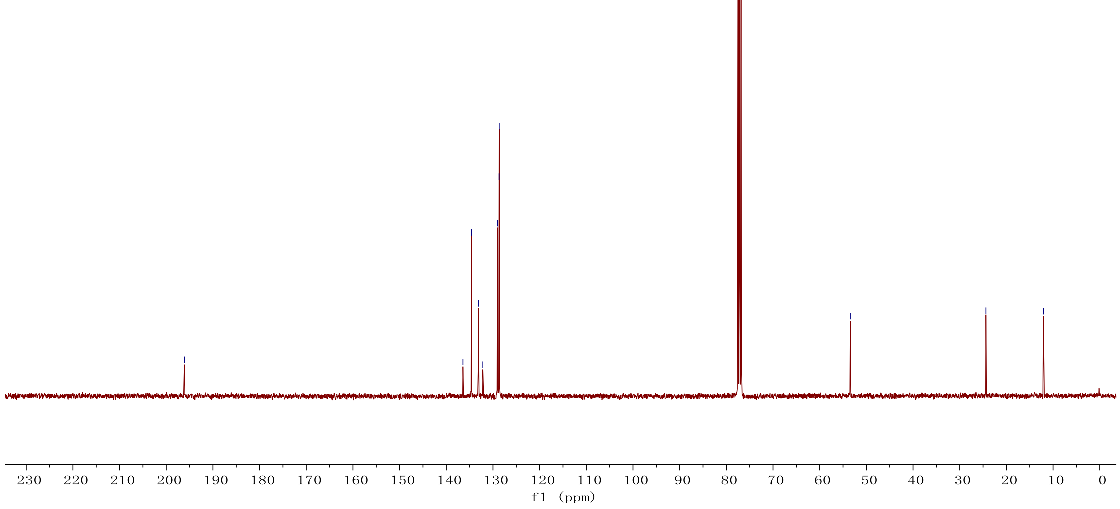

2-(phenylthio)pentanal (7aj)

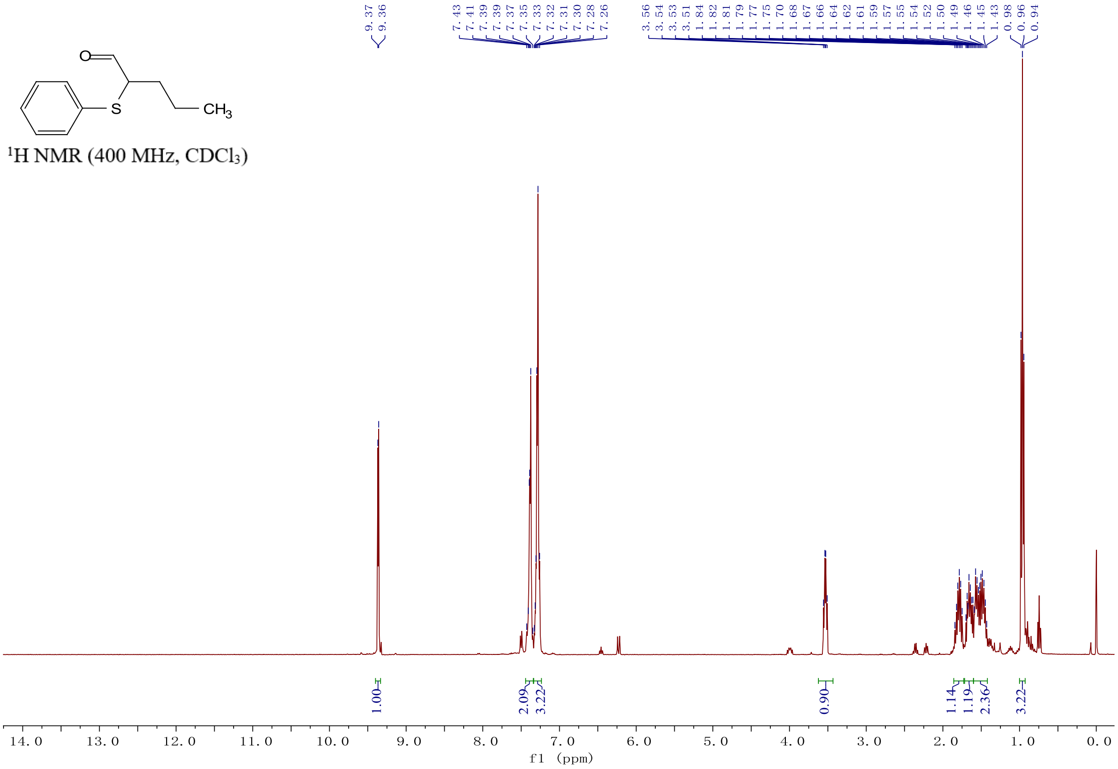

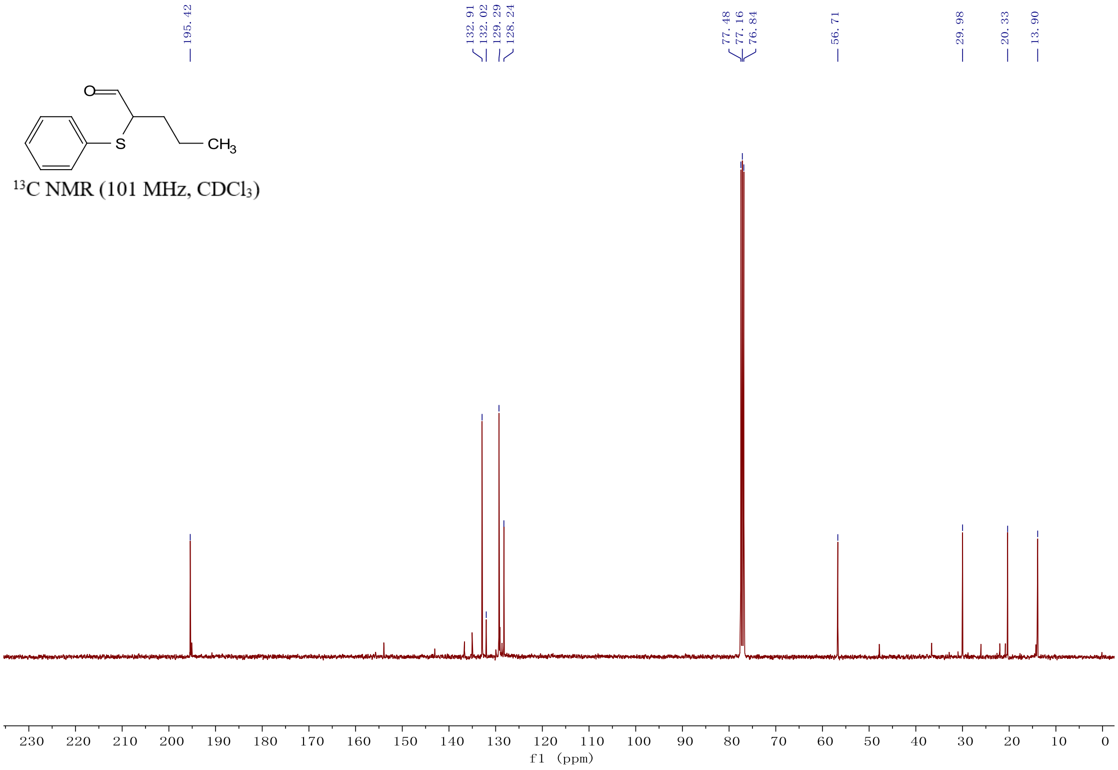

(*Z*)-2-(phenylthio)pentanal oxime/(*E*)-2-(phenylthio)pentanal oxime (*Z*-**7ak**/*E*-**7ak** = 0.5/1)

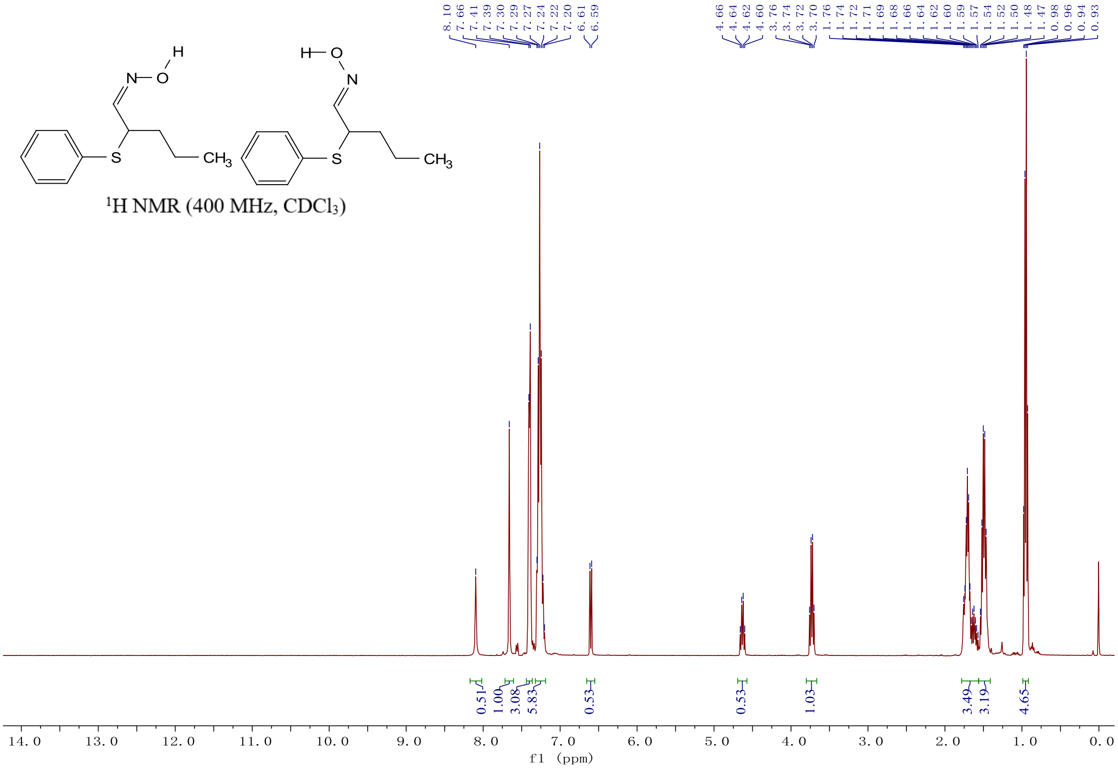

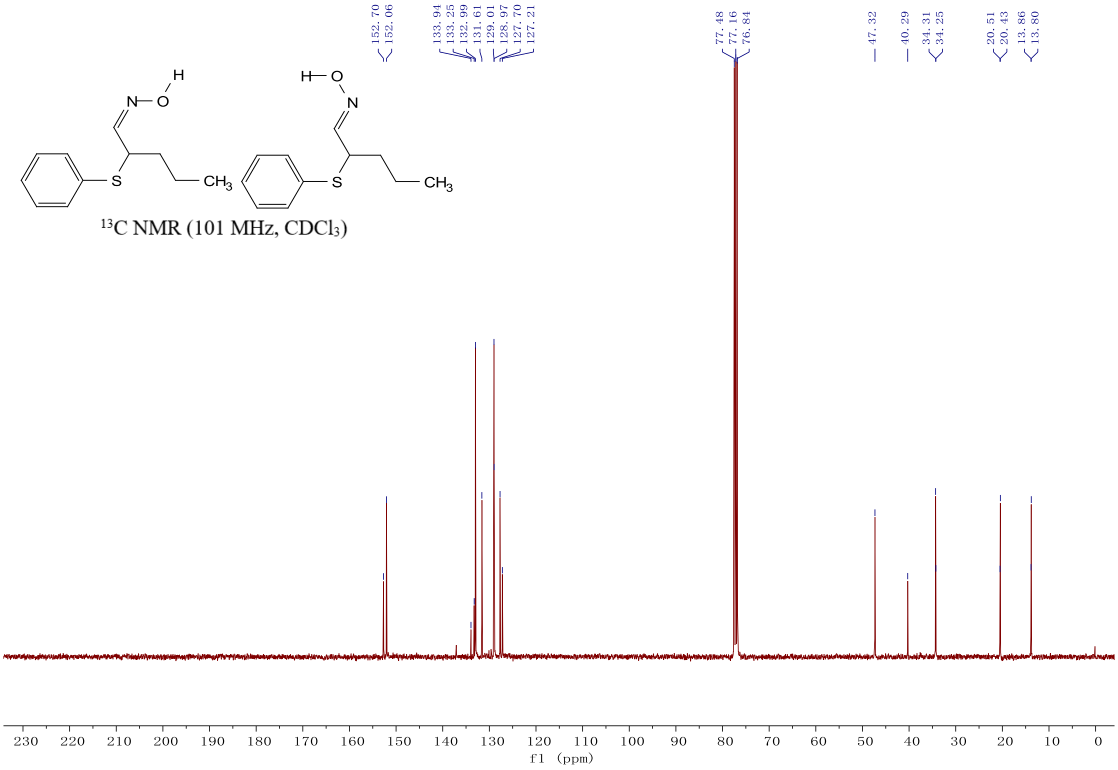

(*Z*)-2-(phenylthio)pentanal oxime/(*E*)-2-(phenylthio)pentanal oxime (*Z*-**7ak**/*E*-**7ak** = 0.5/1)

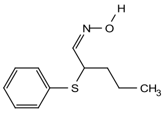

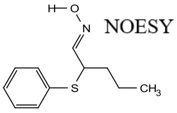

NOESY (400 MHz, $CDCl_3$)

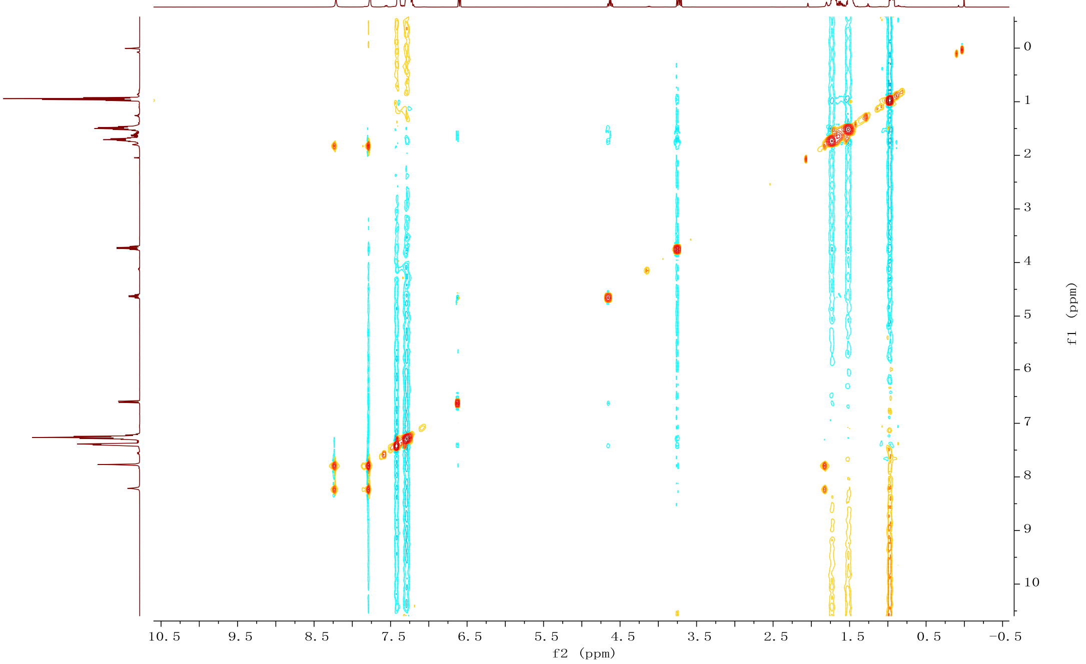

2-(phenylthio)pentanenitrile (7al)

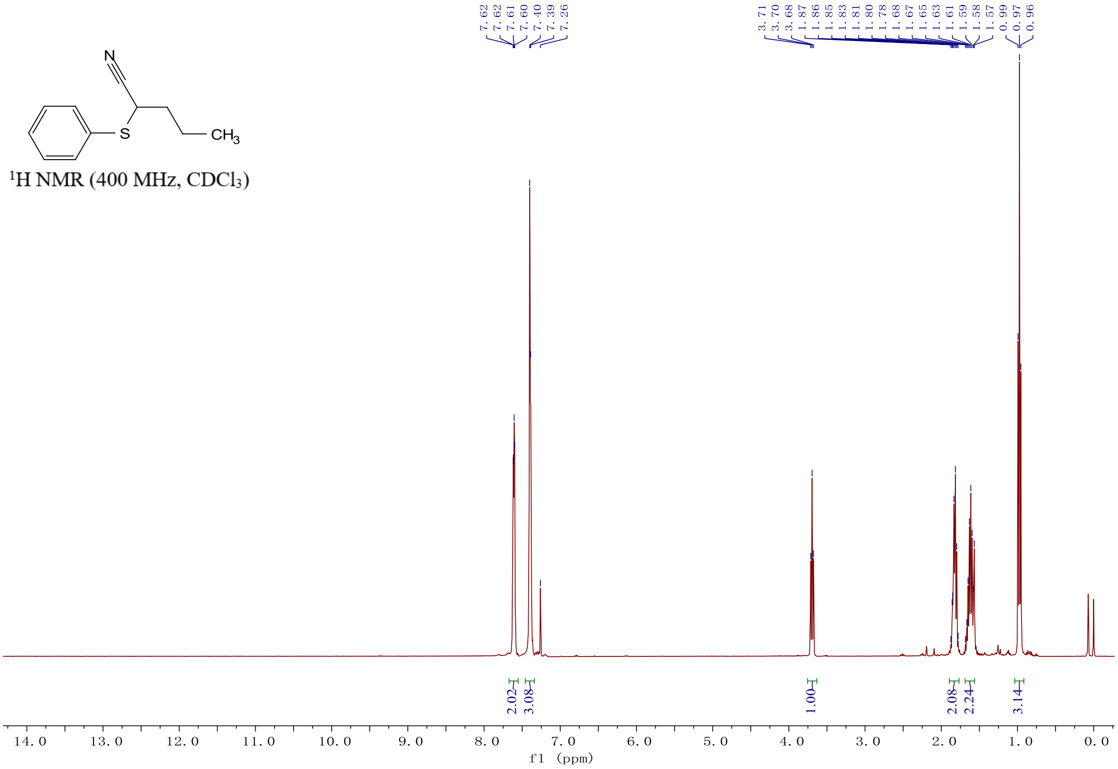

^{1}H NMR (400 MHz, $CDCl_3$)

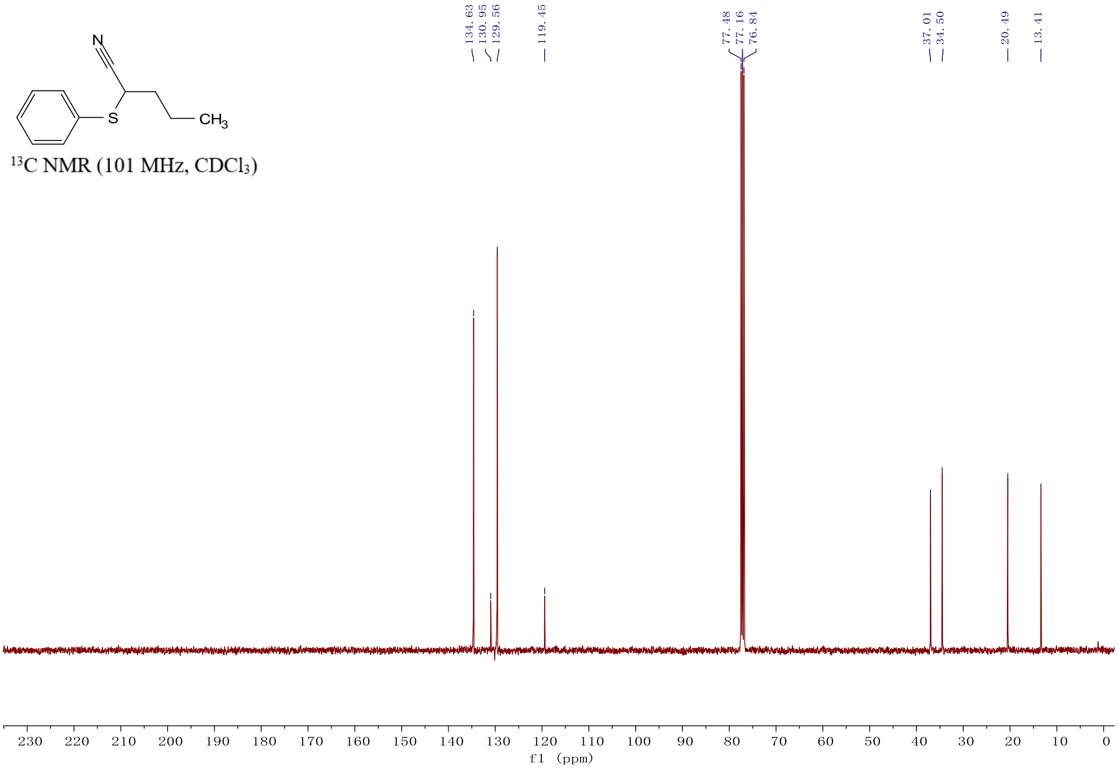

^{13}C NMR (101 MHz, $CDCl_3$)

methyl 2-(phenylthio)butanoate (7am)

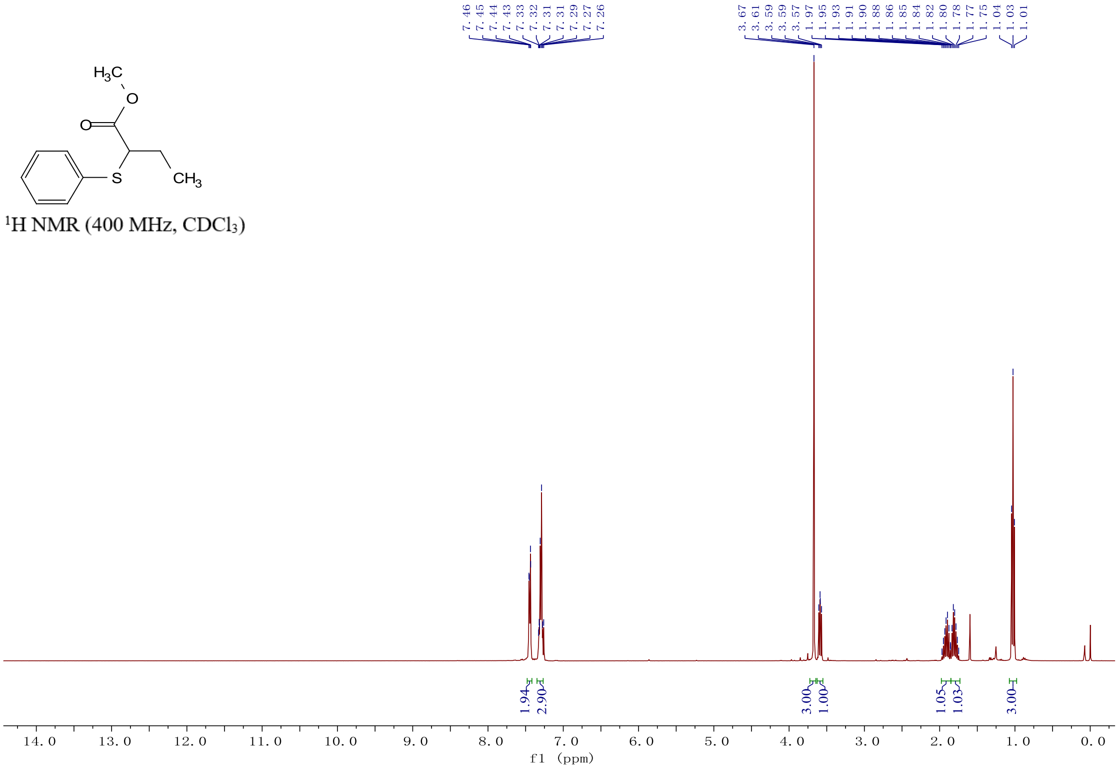

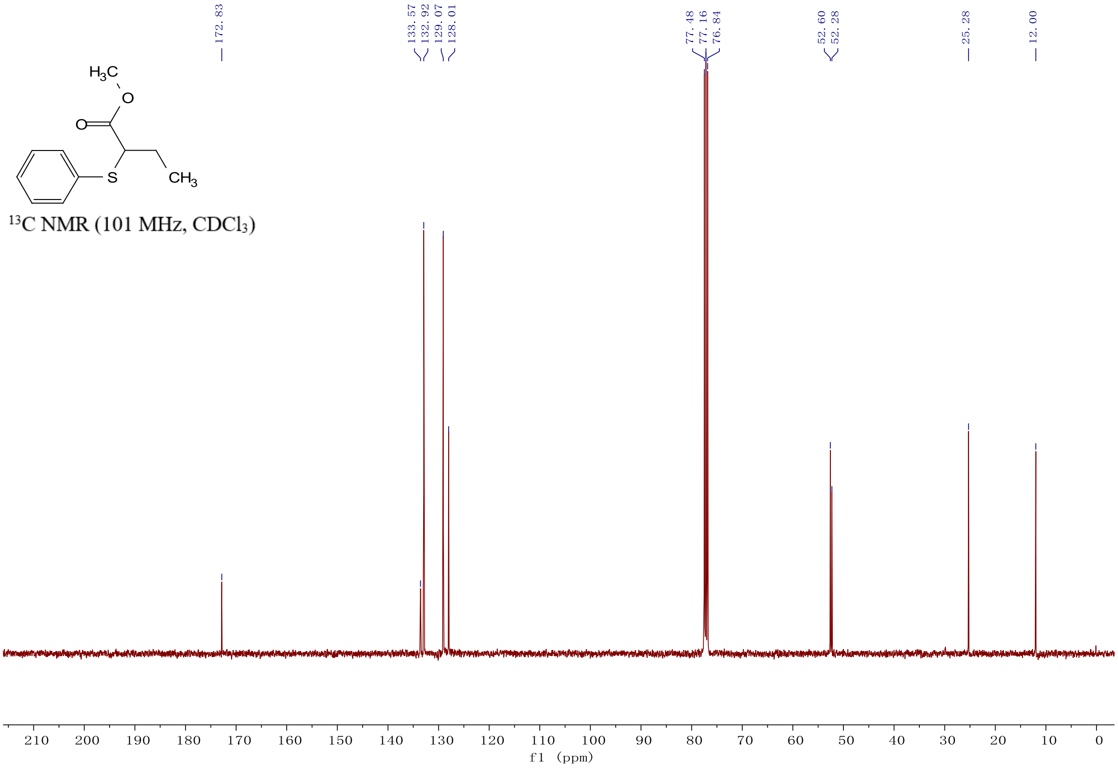

***syn*-3-(phenylthio)hexan-2-ol (*syn*-10aa)**

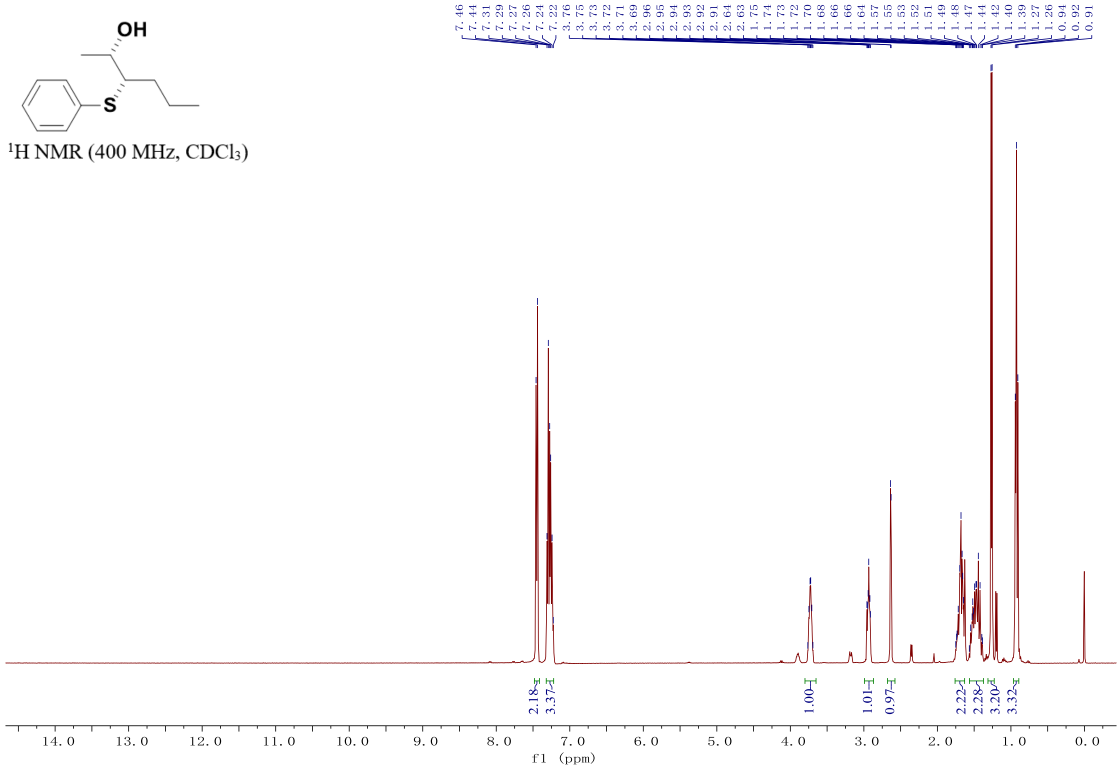

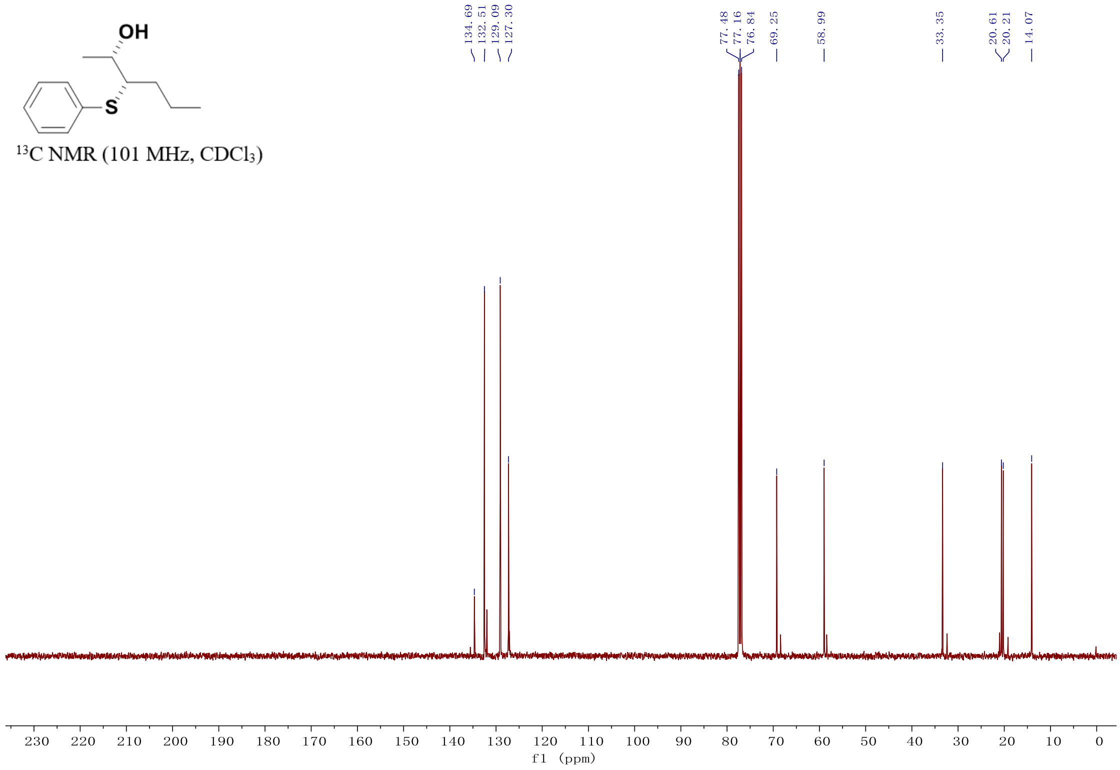

syn-3-((2-fluorophenyl)thio)hexan-2-ol (_syn_-10ab)

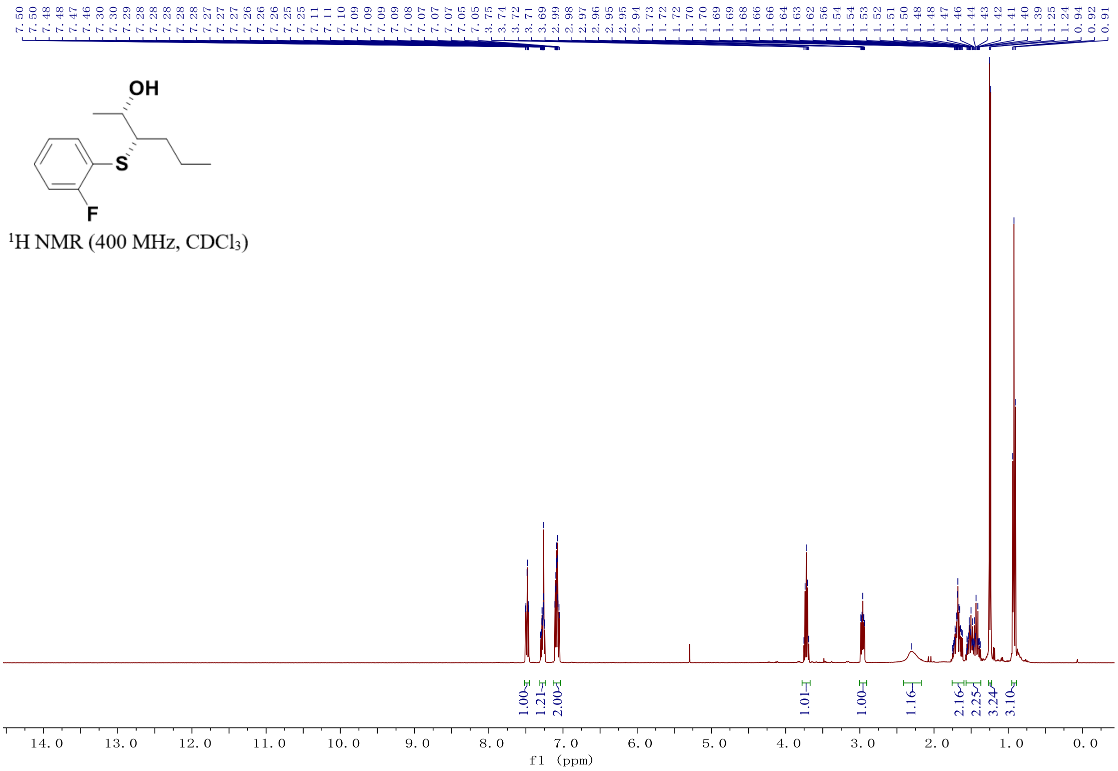

^{1}H NMR (400 MHz, $CDCl_3$)

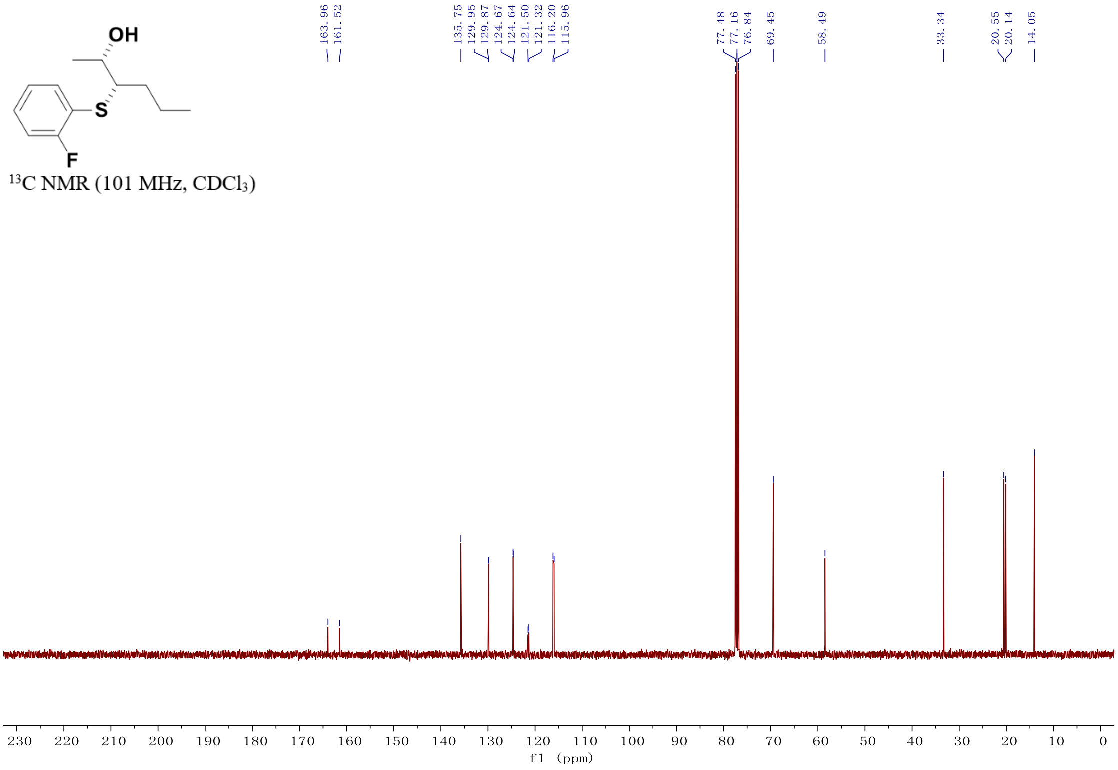

^{13}C NMR (101 MHz, $CDCl_3$)

***syn*-3-((2-fluorophenyl)thio)hexan-2-ol (*syn*-10ab)**

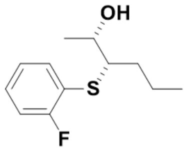

^{19}F NMR (376 MHz, $CDCl_3$)

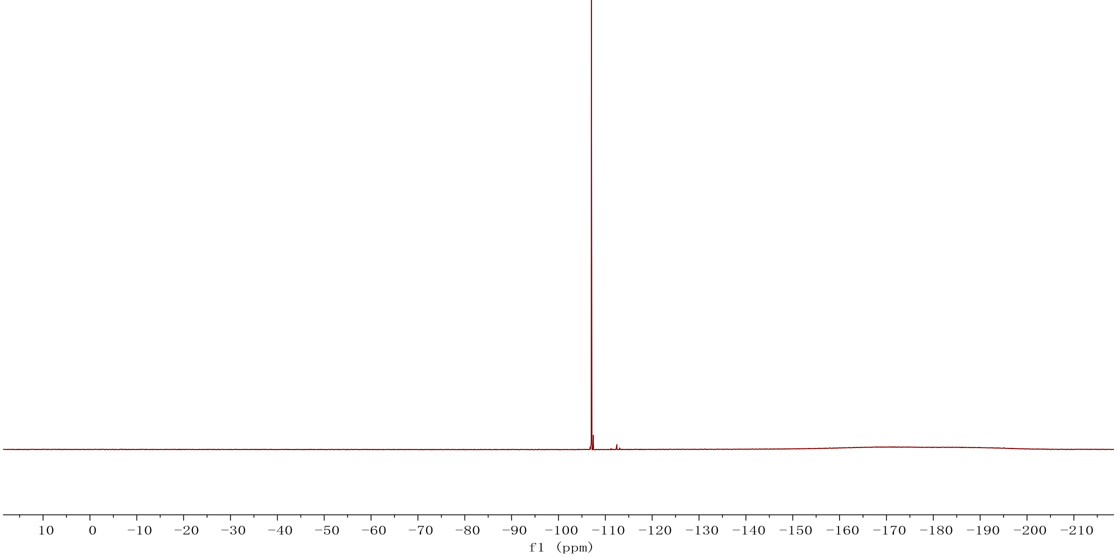

syn-3-((2-chlorophenyl)thio)hexan-2-ol (_syn_-10ac)

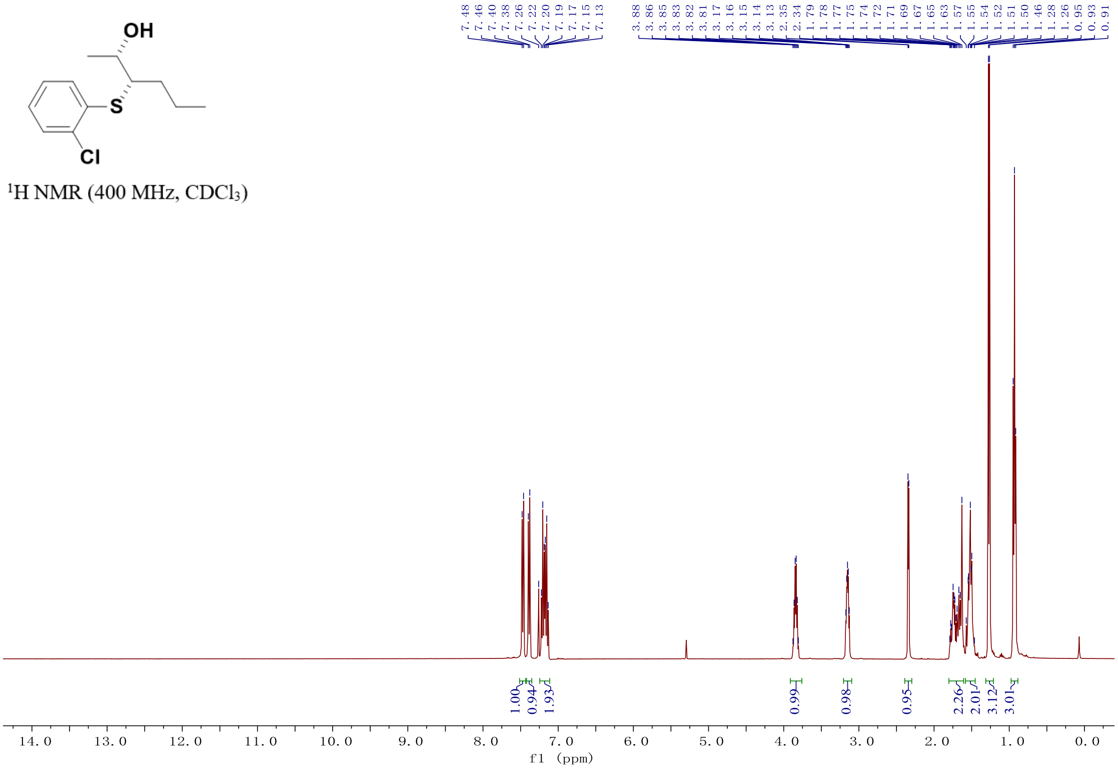

^{1}H NMR (400 MHz, CDCl$_3$)

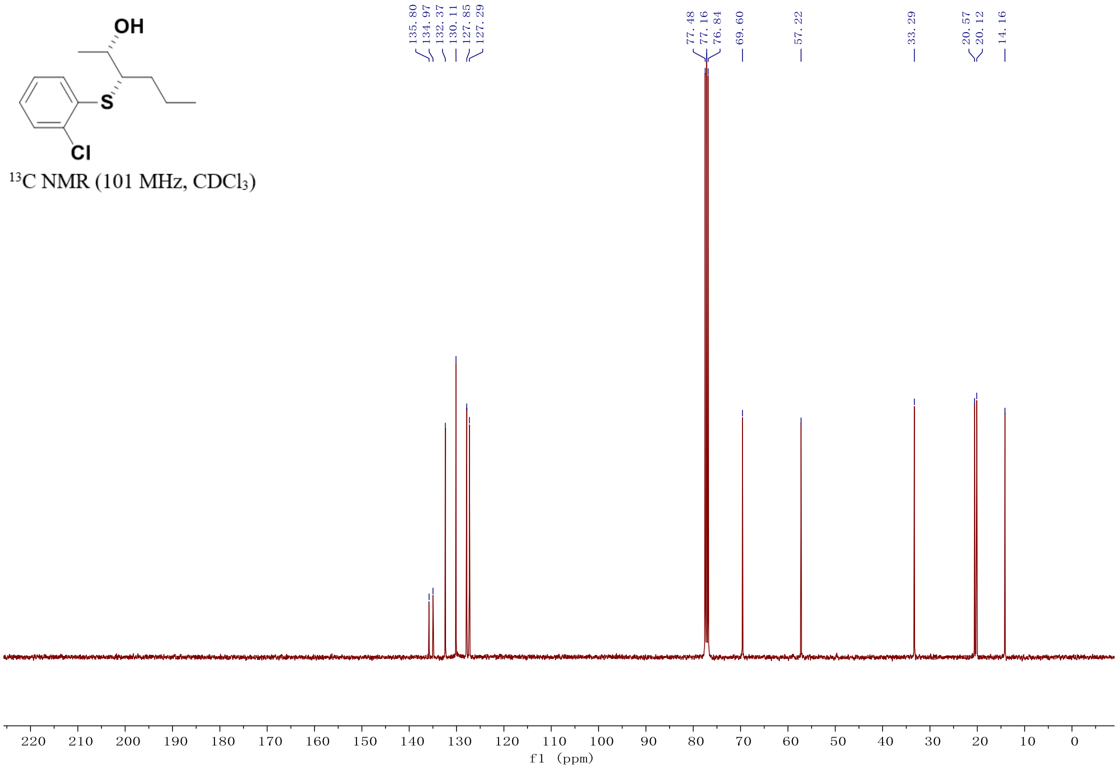

^{13}C NMR (101 MHz, CDCl$_3$)

***syn*-3-((4-chlorophenyl)thio)hexan-2-ol (*syn*-10ad)**

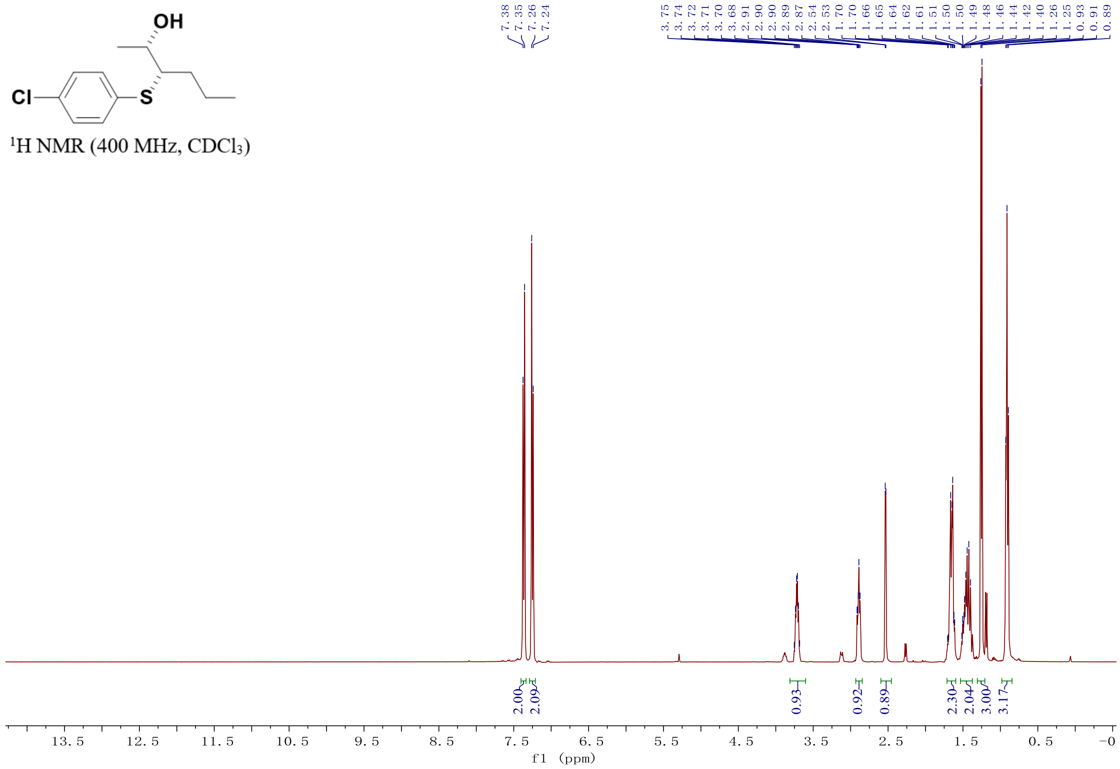

^{1}H NMR (400 MHz, $CDCl_3$)

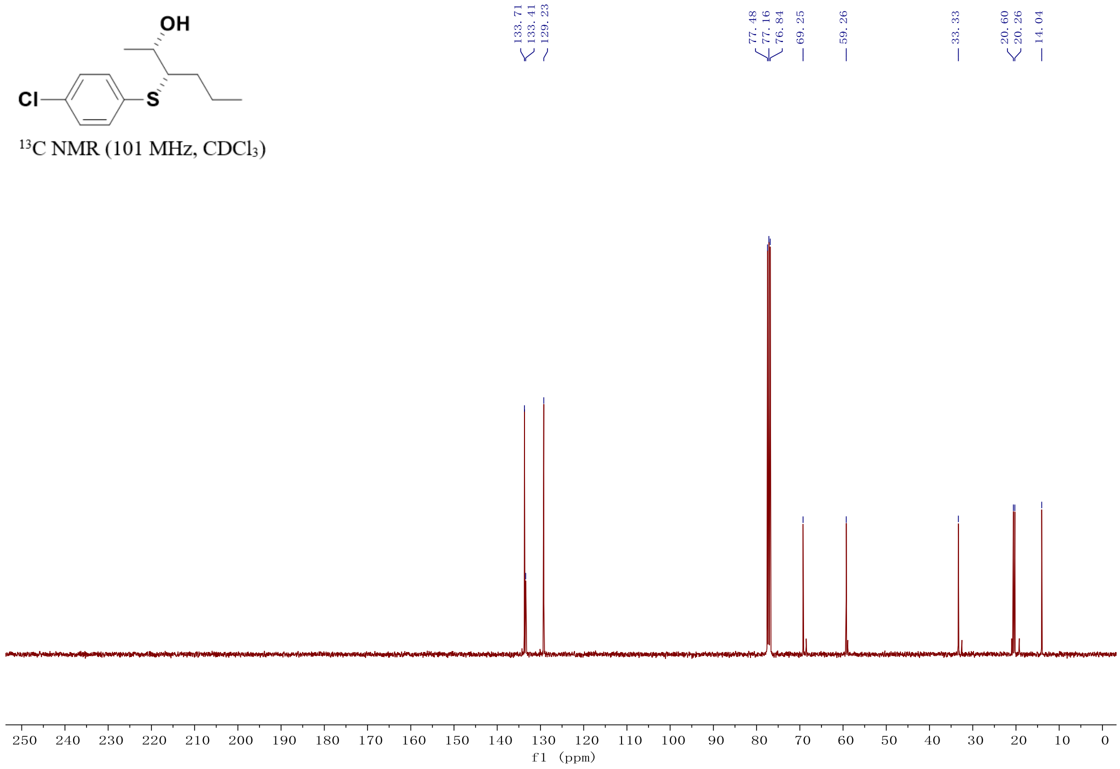

^{13}C NMR (101 MHz, $CDCl_3$)

syn-3-((2-bromophenyl)thio)hexan-2-ol (*syn*-10ae)

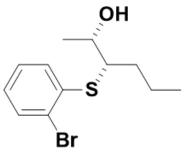

^{1}H NMR (400 MHz, $CDCl_3$)

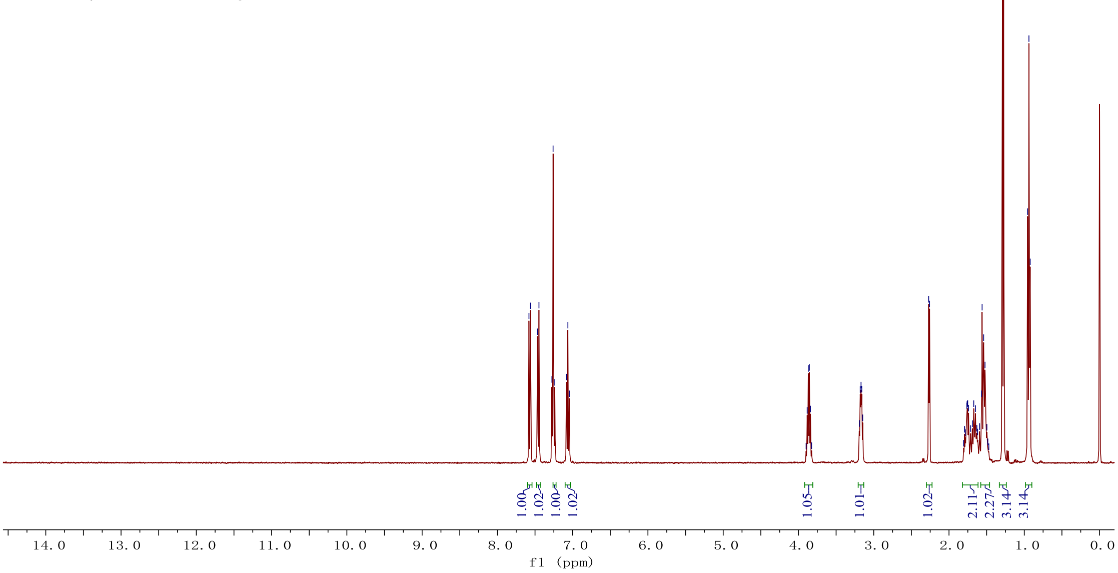

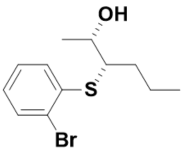

^{13}C NMR (101 MHz, $CDCl_3$)

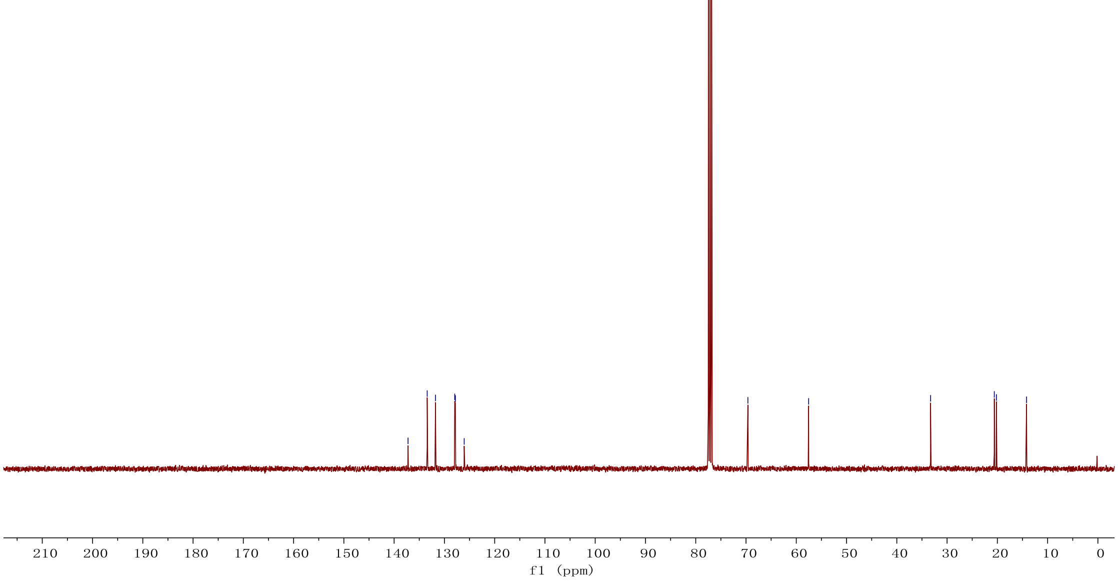

(*Z*)-4-(phenylthio)hex-3-en-2-one (*Z*-6aa)

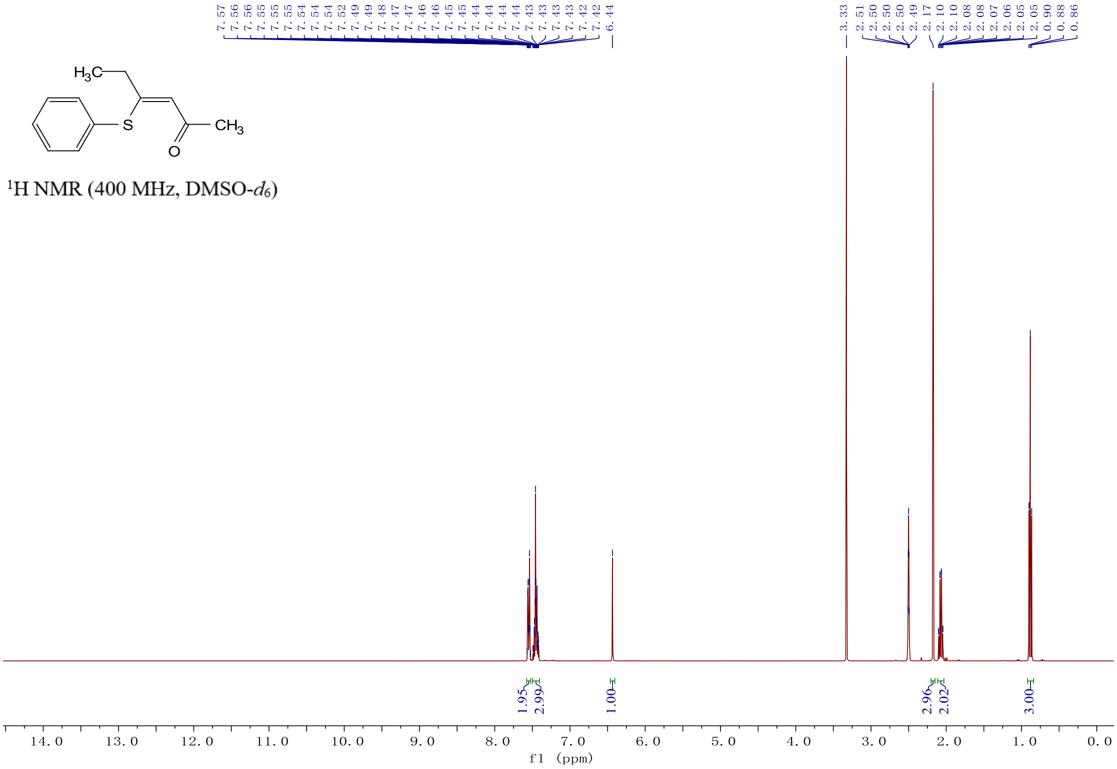

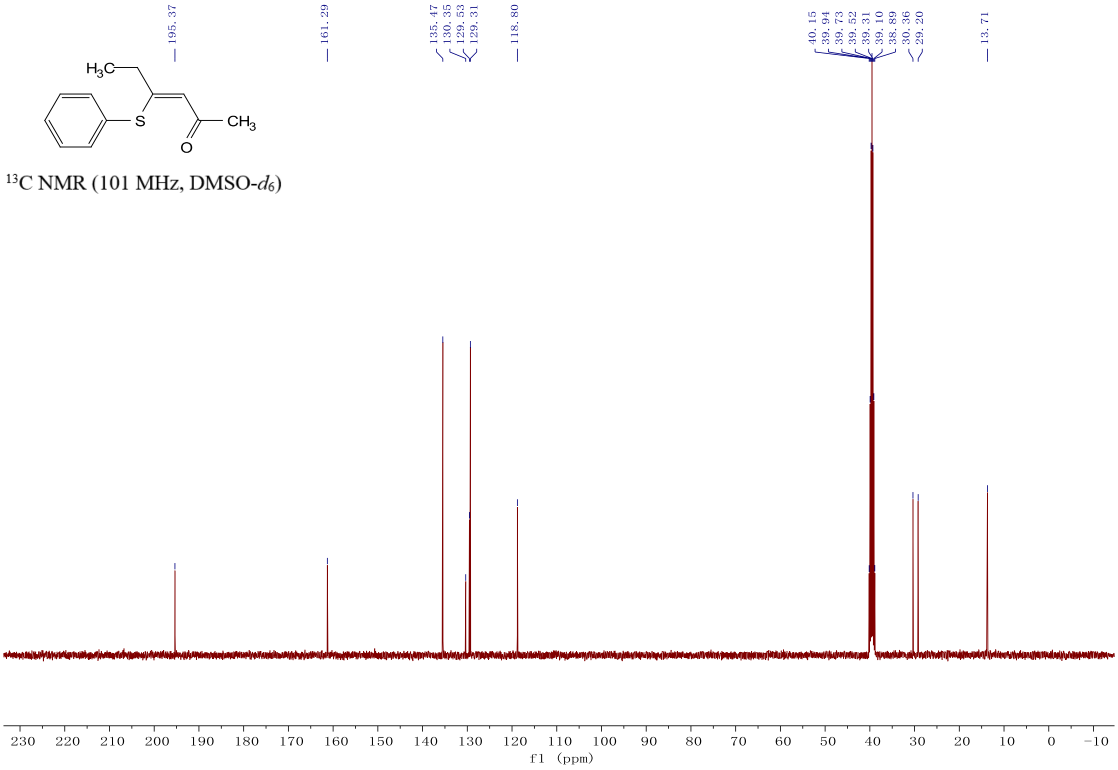

(*Z*)-4-(phenylthio)hex-3-en-2-one (*Z*-6aa)

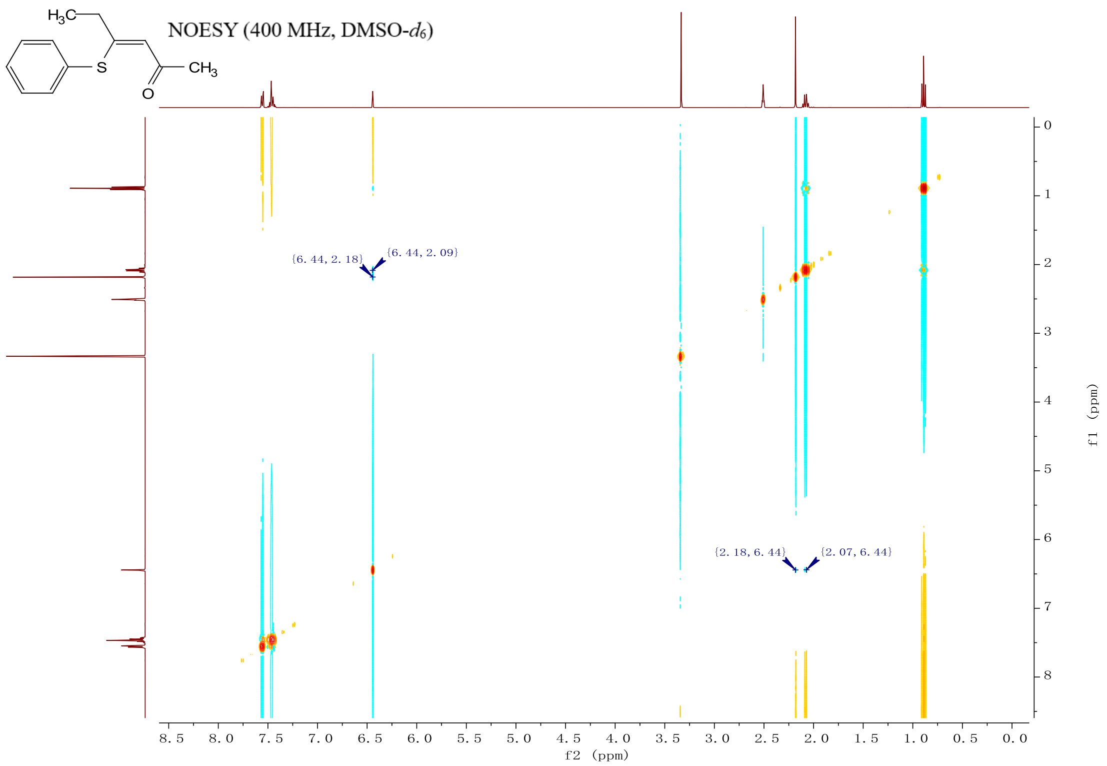

(*E*)-4-(phenylthio)hex-3-en-2-one (*E*-6aa)

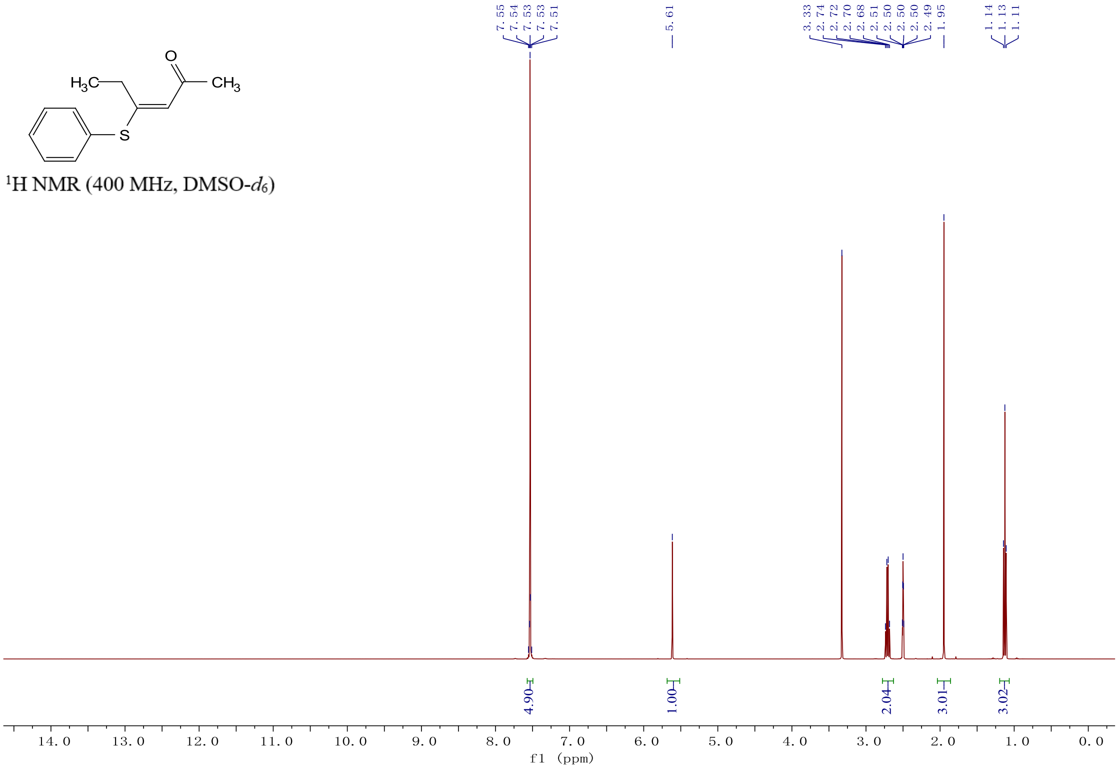

^{1}H NMR (400 MHz, DMSO-d_6)

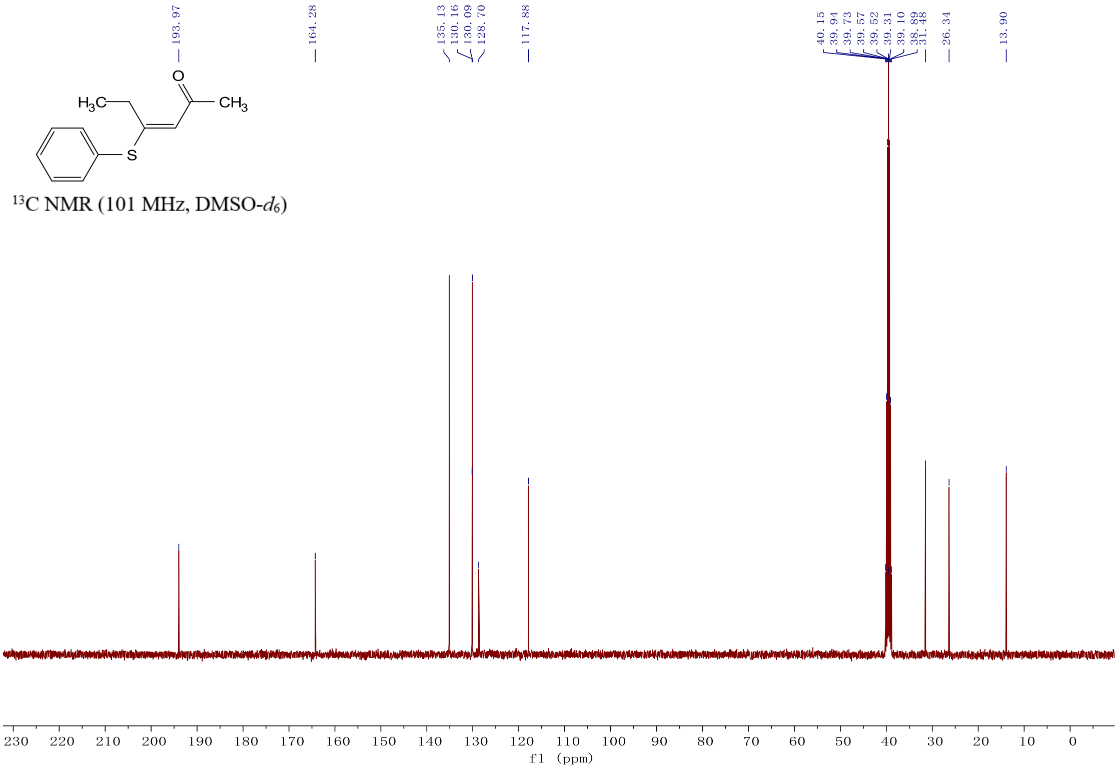

^{13}C NMR (101 MHz, DMSO-d_6)

(*E*)-4-(phenylthio)hex-3-en-2-one (*E*-6aa)

NOESY (400 MHz, DMSO-d_6)

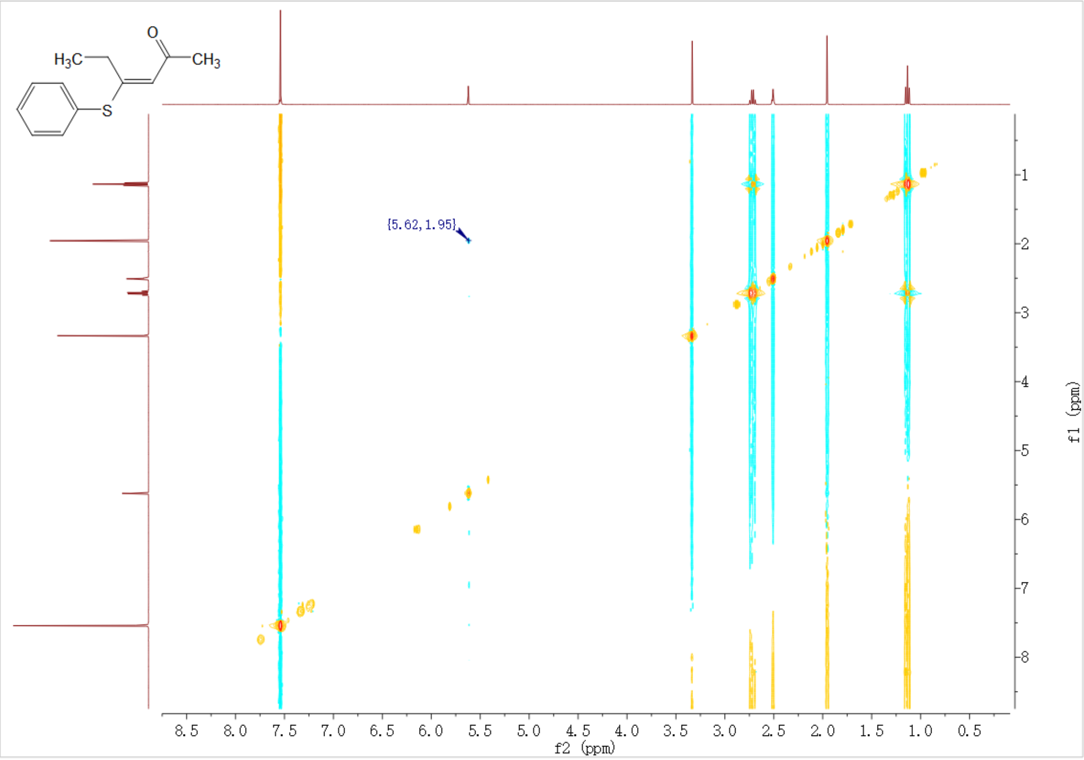

methyl (*Z*)-3-(phenylthio)but-2-enoate (*Z*-6ab)

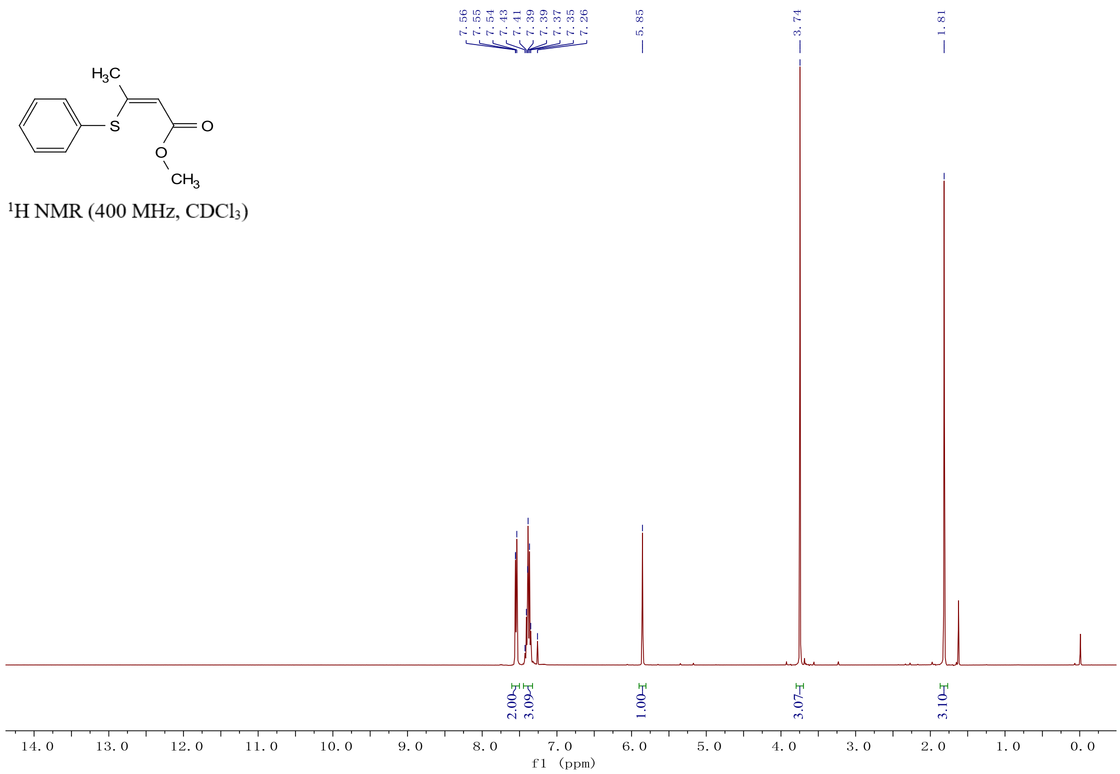

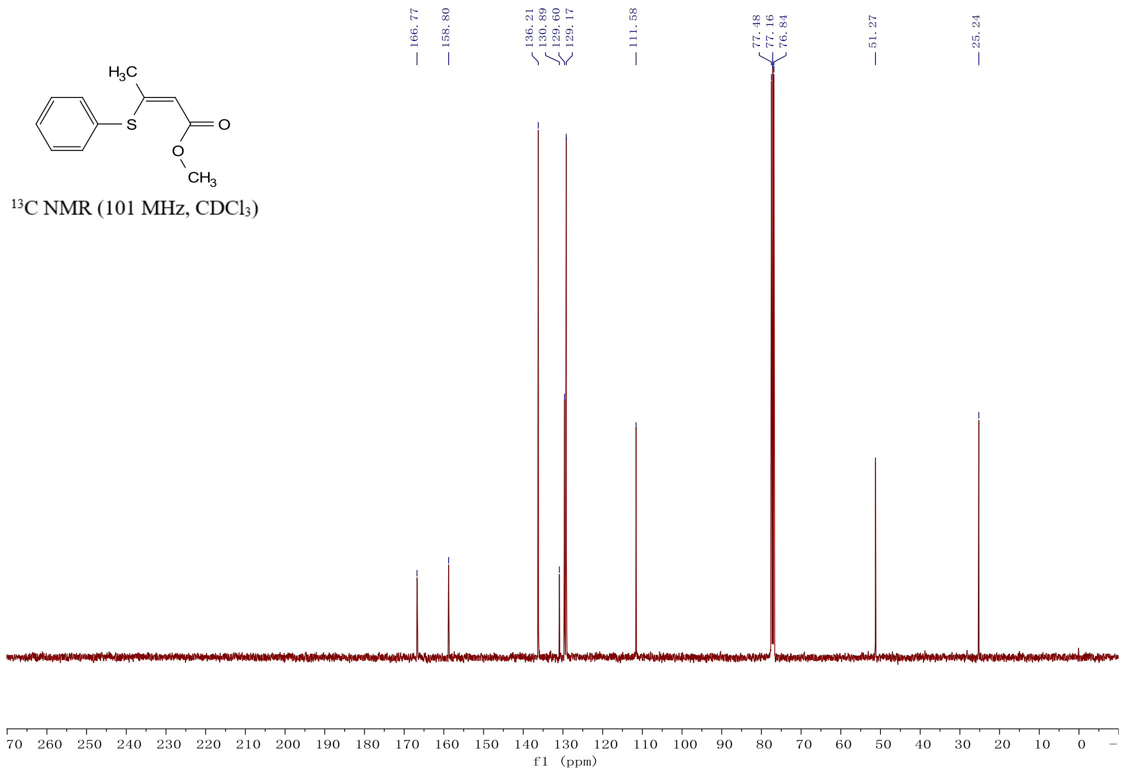

methyl (*Z*)-3-(phenylthio)but-2-enoate (*Z*-6ab)

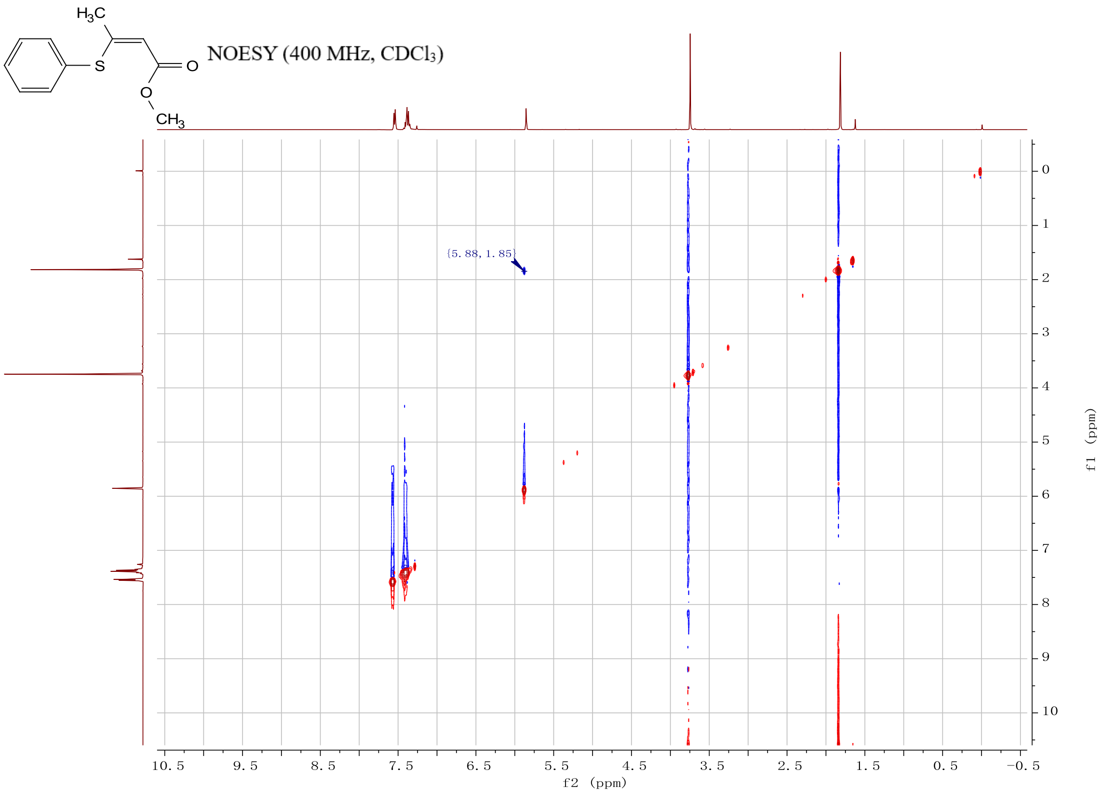

methyl (*E*)-3-(phenylthio)but-2-enoate (*E*-6ab)

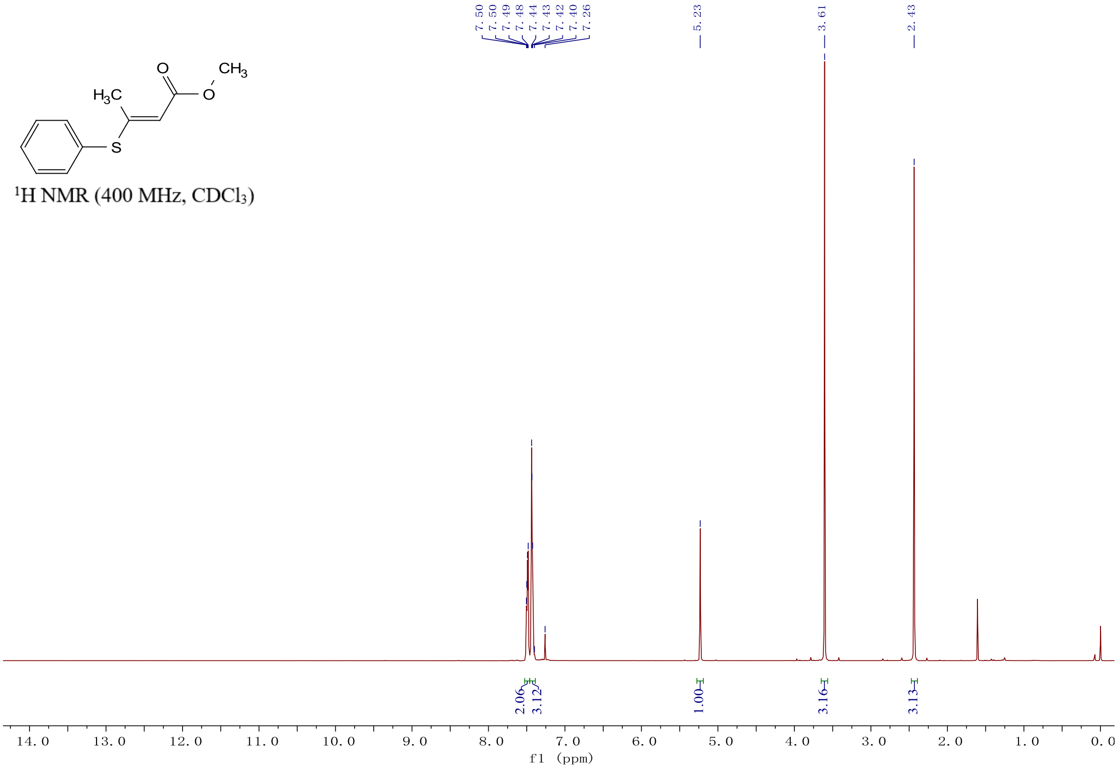

^{1}H NMR (400 MHz, $CDCl_3$)

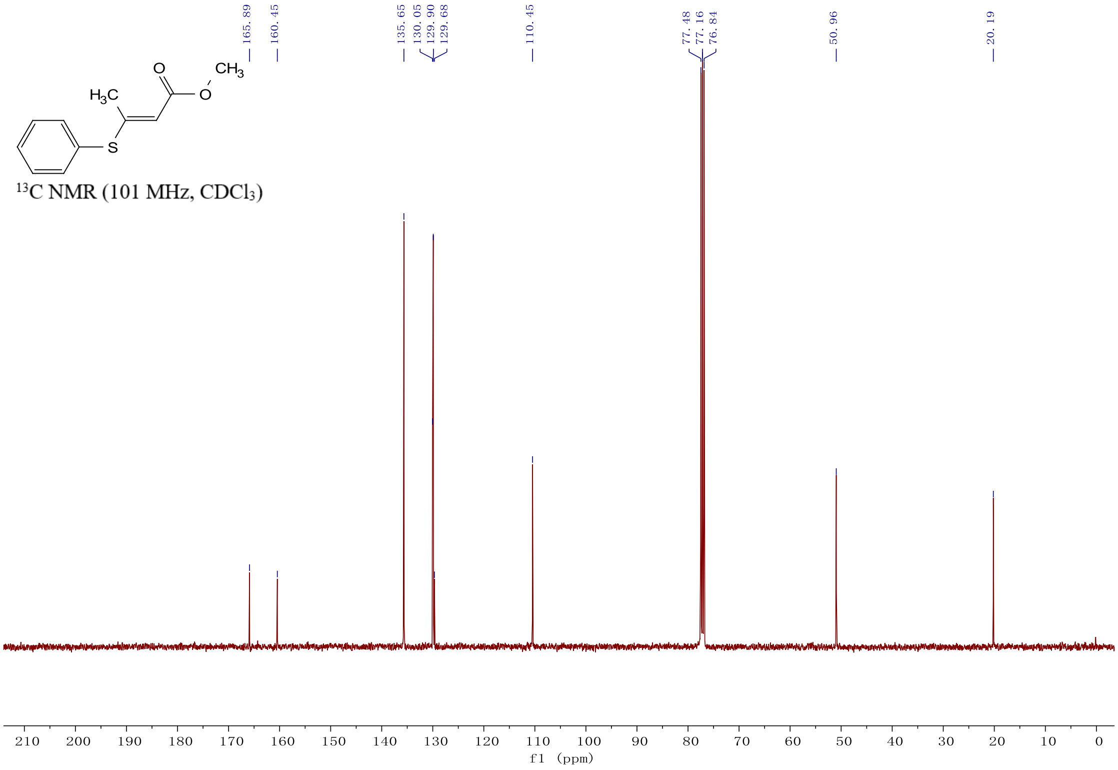

^{13}C NMR (101 MHz, $CDCl_3$)

methyl (*E*)-3-(phenylthio)but-2-enoate (*E*-6ab)

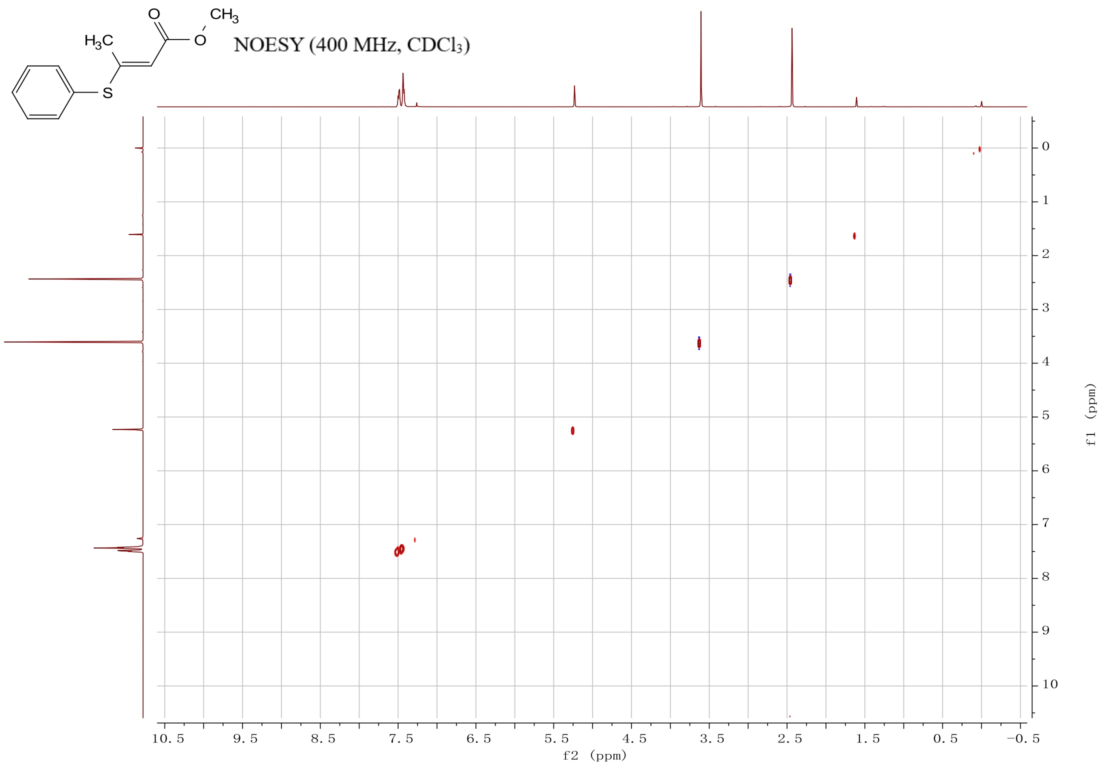

4-(phenylthio)hexan-2-one (8aa)

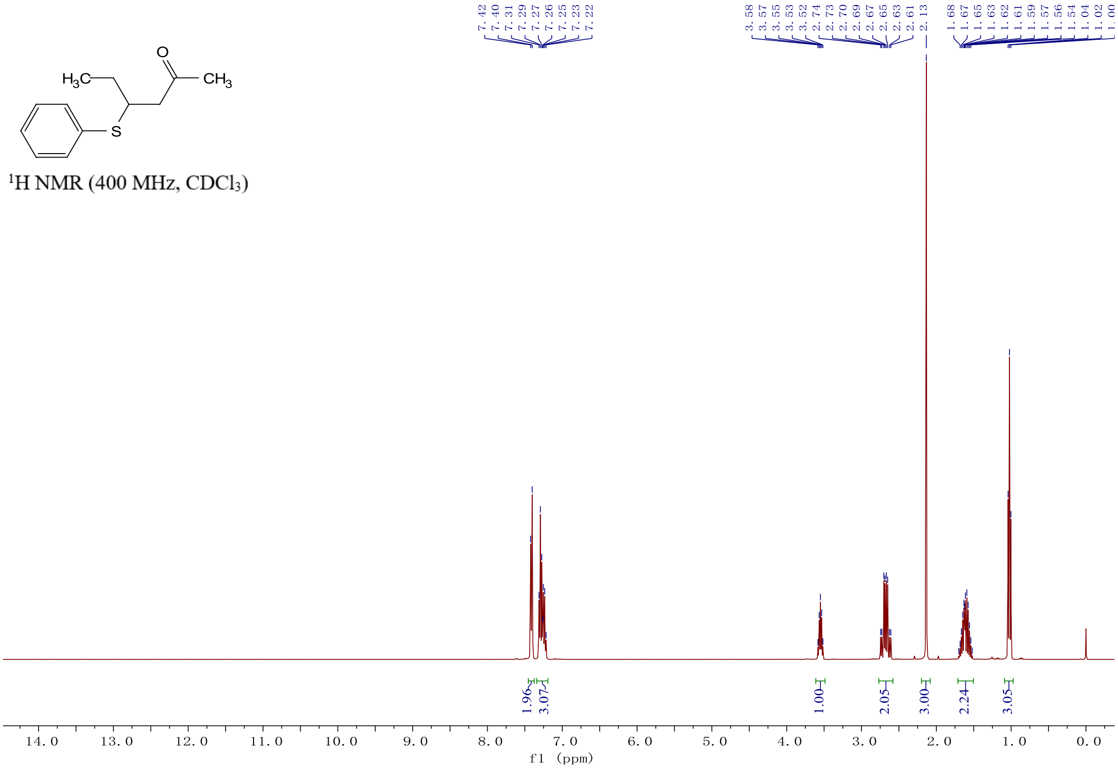

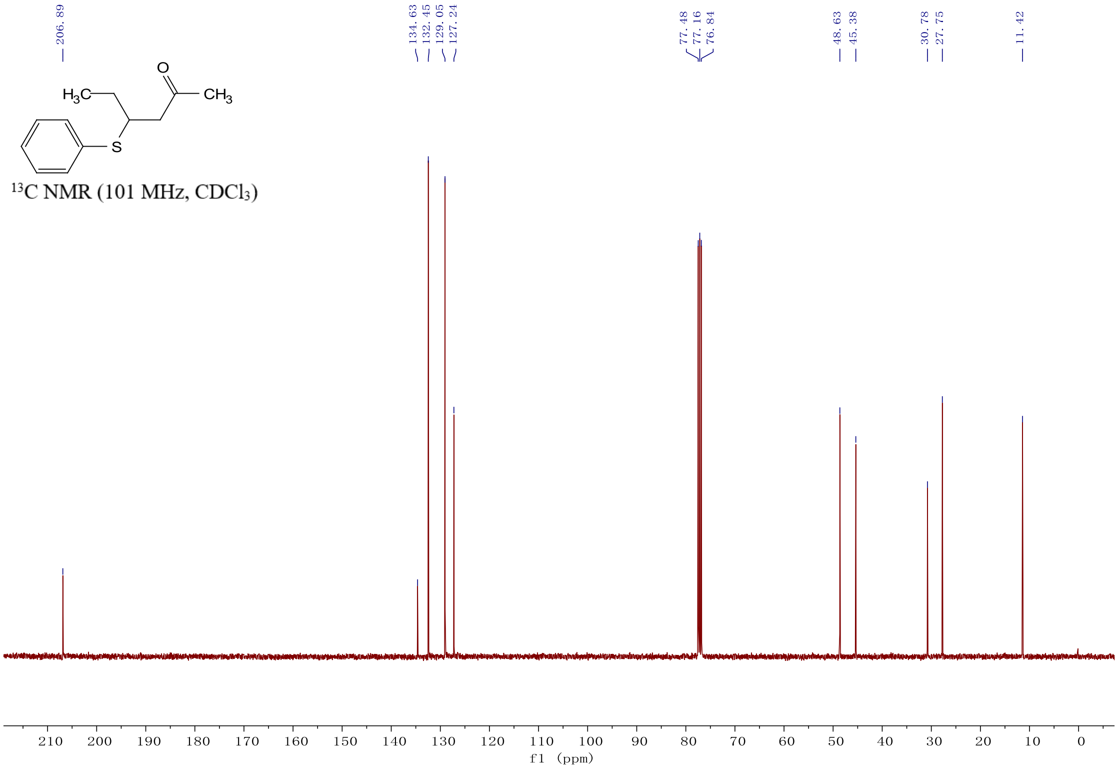

methyl 3-(phenylthio)butanoate (8ab)

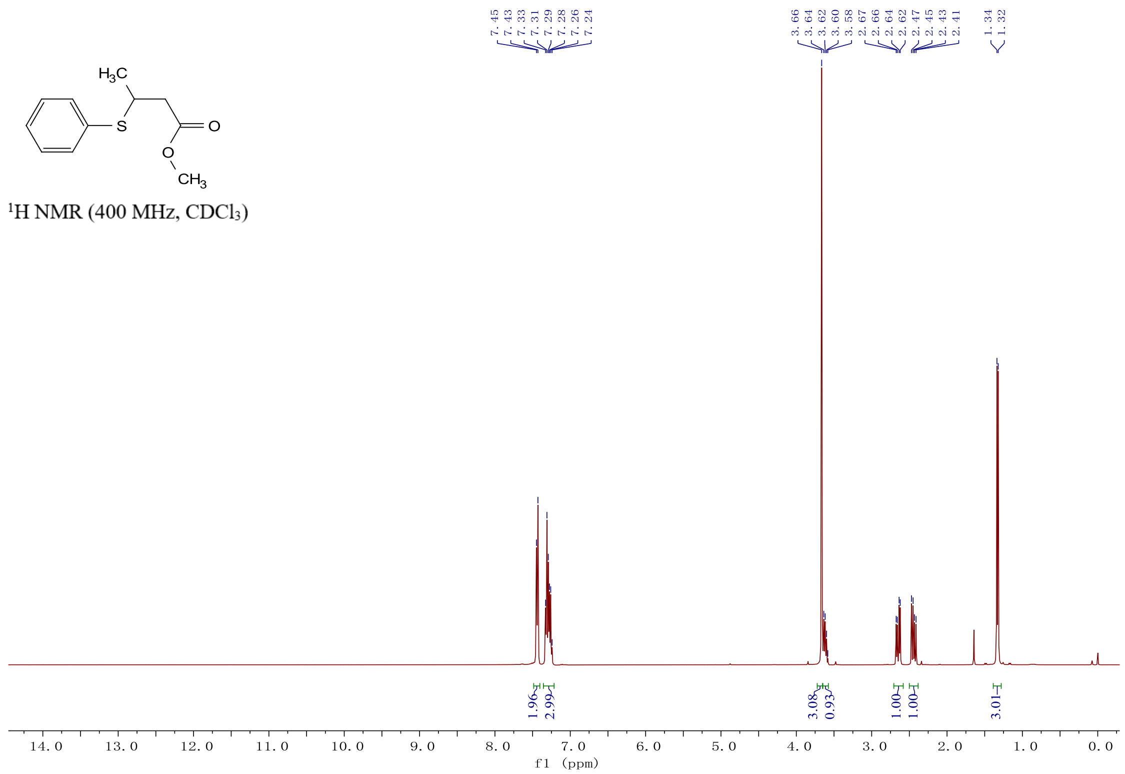

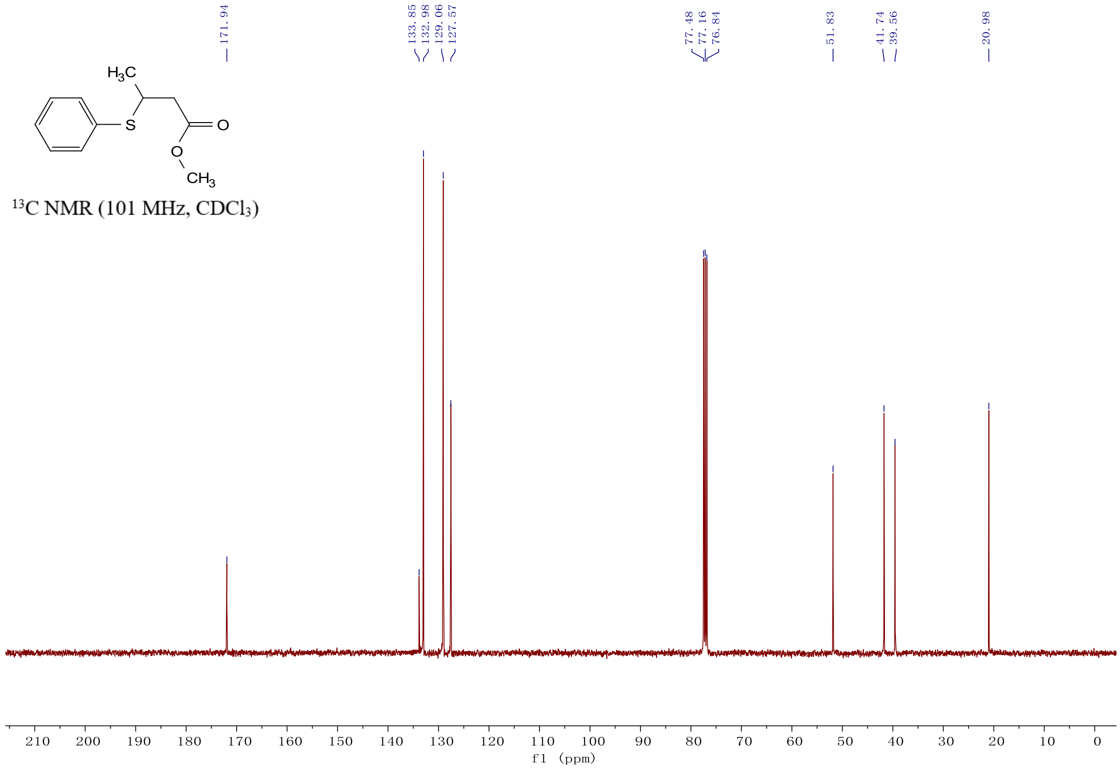

2-(phenylthio)butane-1,4-diol (11aa)

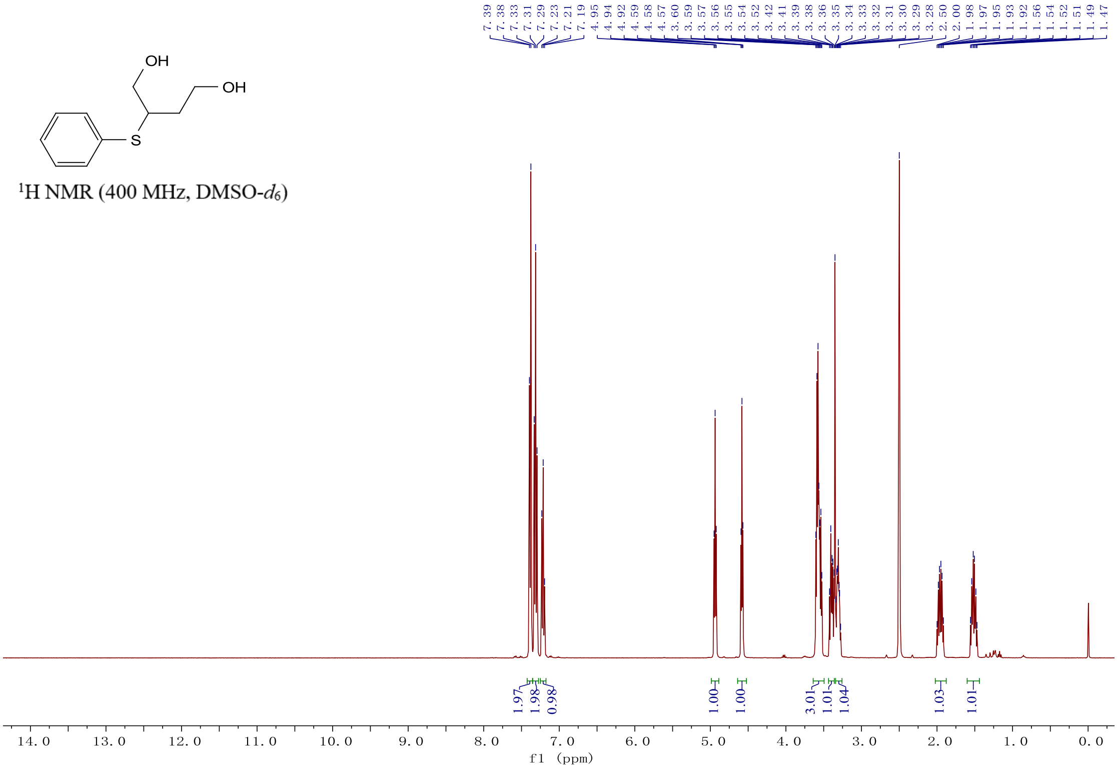

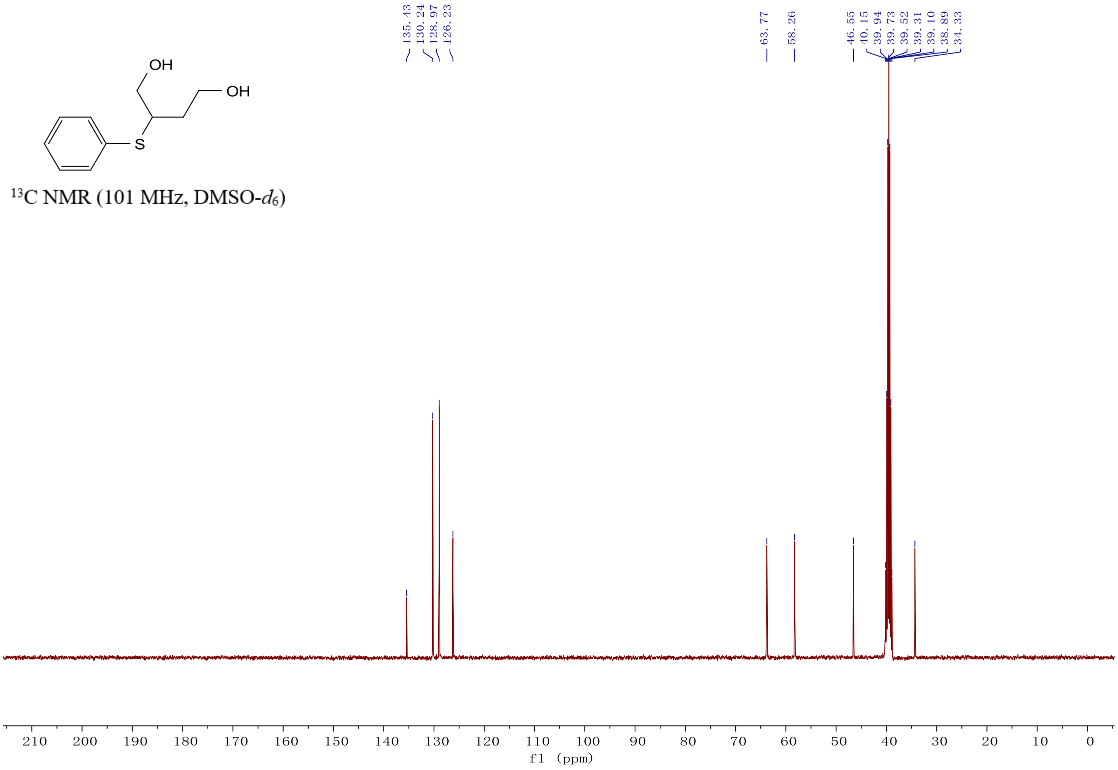

VI. Conditions for HPLC Analysis and Copies of HPLC Spectra

HPLC Analysis for ENE-101 Biocatalysed Reduction of (*Z*)-1 and/or (*E*)-1 into (*S*)-2

HPLC analysis of **2aa**: Chiralpak® IG column (4.6 mm × 250 mm, 5 μm); detected at 254 nm; heptane/EtOH = 90/10; flow rate: 1.0 mL/min.

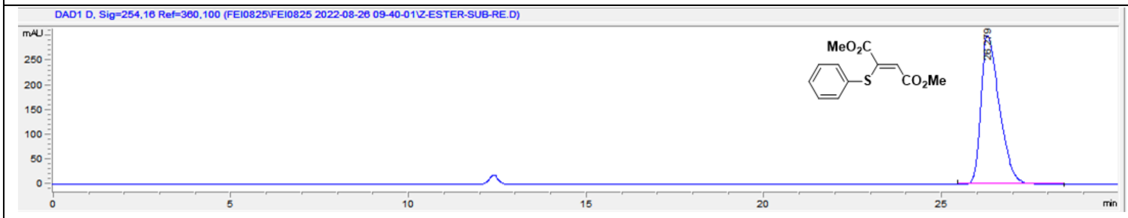

#	Time	Area	Height	Width	Area%	Symmetry
1	26.279	10846.2	300.4	0.5472	100.000	0.532

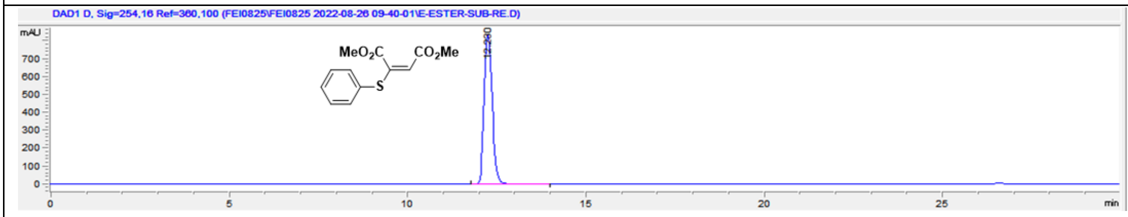

#	Time	Area	Height	Width	Area%	Symmetry
1	12.23	13073.2	834.5	0.2428	100.000	0.747

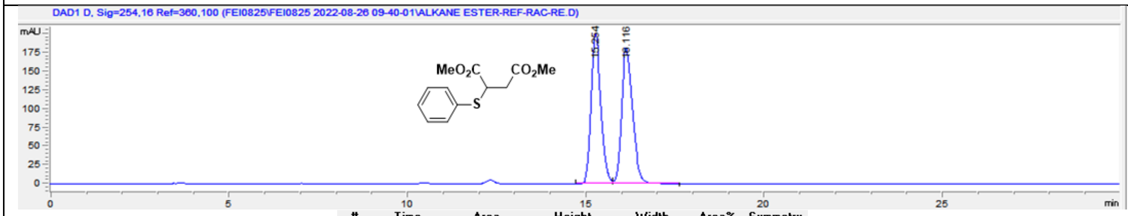

#	Time	Area	Height	Width	Area%	Symmetry
1	15.254	3785	199.8	0.293	49.423	0.753
2	16.116	3873.4	181.4	0.3292	50.577	0.695

Obtained with ENE-101 from (*Z*)-**1aa**

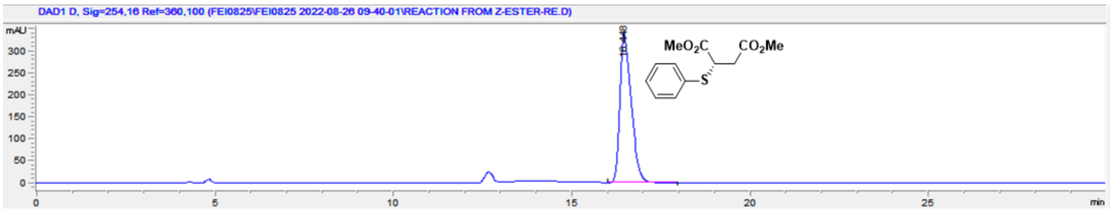

#	Time	Area	Height	Width	Area%	Symmetry
1	16.448	7642.5	341.5	0.3431	100.000	0.544

Obtained with ENE-101 from (*E*)-**1aa**

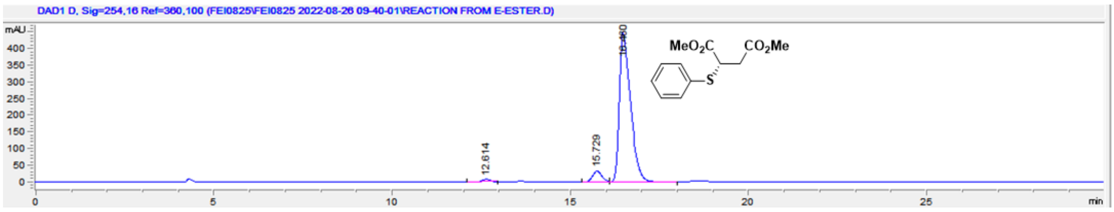

#	Time	Area	Height	Width	Area%	Symmetry
1	12.614	129.9	8.4	0.2411	1.214	0.974
2	15.729	614.8	34.2	0.2798	5.747	0.915
3	16.46	9953.7	450.6	0.3377	93.039	0.485

Obtained with ENE-101 from (*Z*)-**1aa**/(*E*)-**1aa** = 4.1/1

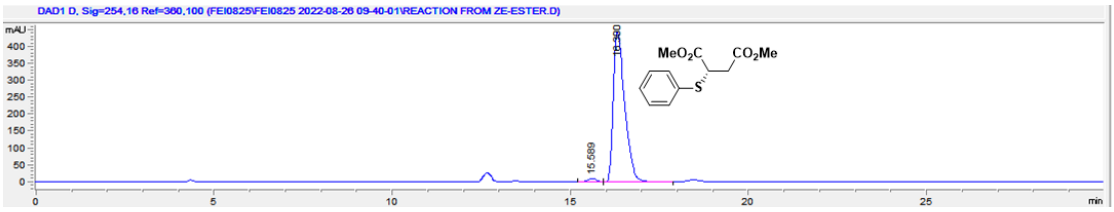

#	Time	Area	Height	Width	Area%	Symmetry
1	15.589	182.4	10.5	0.2683	1.827	0.96
2	16.3	9798.9	443.6	0.3377	98.173	0.484

HPLC analysis of **2ab**: Chiralpak® IG column (4.6 mm × 250 mm, 5 μm); detected at 254 nm; heptane/EtOH = 96/4; flow rate: 1.0 mL/min.

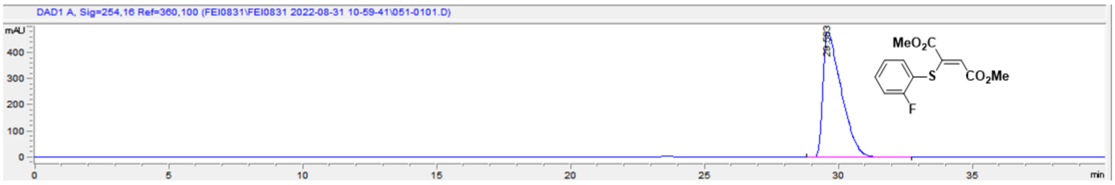

#	Time	Area	Height	Width	Area%	Symmetry
1	29.583	22954.9	481.4	0.7043	100.000	0.335

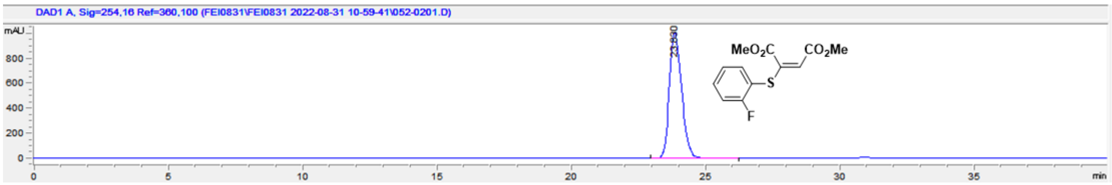

#	Time	Area	Height	Width	Area%	Symmetry
1	23.83	32385.5	1014	0.4926	100.000	0.715

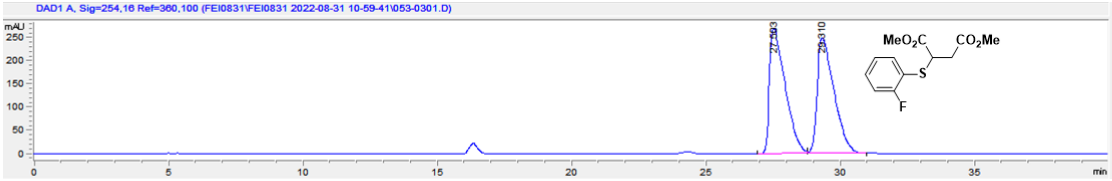

#	Time	Area	Height	Width	Area%	Symmetry
1	27.503	11002.2	271.6	0.5927	50.442	0.344
2	29.31	10809.4	248.6	0.6552	49.558	0.404

Obtained with ENE-101 from (*Z*)-**1ab**

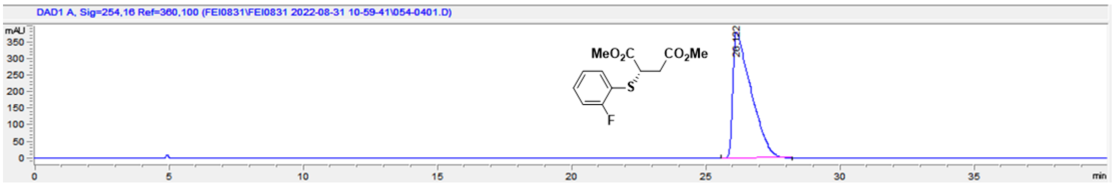

#	Time	Area	Height	Width	Area%	Symmetry
1	26.122	18071.4	381	0.6554	100.000	0.24

Obtained with ENE-101 from (*E*)-**1ab**

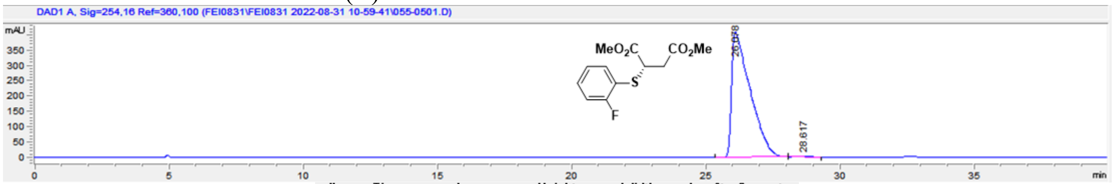

#	Time	Area	Height	Width	Area%	Symmetry
1	26.078	19714.3	411.7	0.6607	99.395	0.229
2	28.617	120	3.5	0.4916	0.605	0.792

Obtained with ENE-101 from (*Z*)-**1ab**/(*E*)-**1ab** = 2.5/1

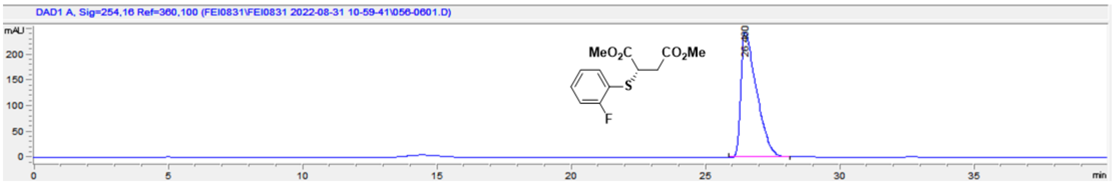

#	Time	Area	Height	Width	Area%	Symmetry
1	26.46	9920.2	243.8	0.6026	100.000	0.356

HPLC analysis of **2ac**: Chiralpak® IG column (4.6 mm × 250 mm, 5 μm); detected at 254 nm; heptane/EtOH = 90/10; flow rate: 1.0 mL/min.

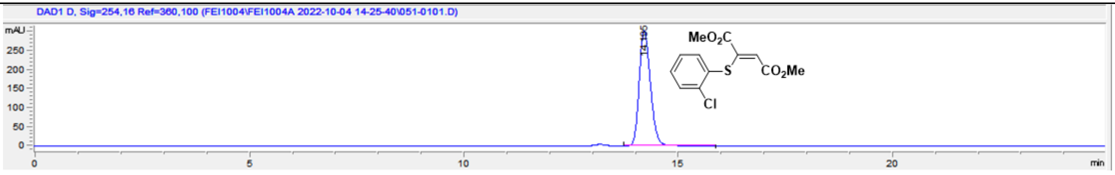

#	Time	Area	Height	Width	Area%	Symmetry
1	14.195	5448.7	301.8	0.2807	100.000	0.737

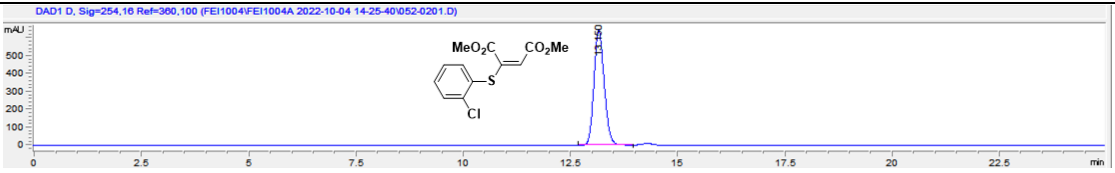

#	Time	Area	Height	Width	Area%	Symmetry
1	13.15	10757.9	643.4	0.2571	100.000	0.811

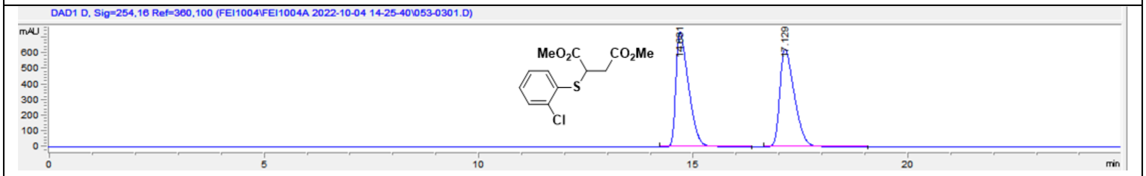

#	Time	Area	Height	Width	Area%	Symmetry
1	14.681	15070.2	734	0.3055	50.599	0.457
2	17.129	14713.5	625	0.3547	49.401	0.53

Obtained with ENE-101 from (*Z*)-**1ac**

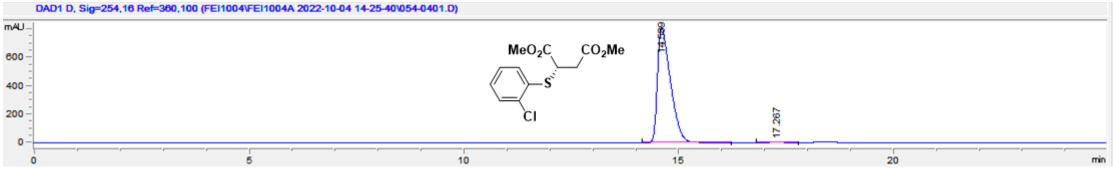

#	Time	Area	Height	Width	Area%	Symmetry
1	14.589	17148.4	809.3	0.3212	99.293	0.43
2	17.267	122.1	5.5	0.3418	0.707	0.921

Obtained with ENE-101 from (*E*)-**1ac**

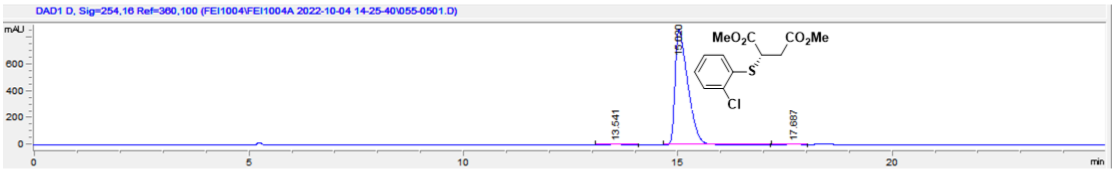

#	Time	Area	Height	Width	Area%	Symmetry
1	13.541	100	5	0.3103	0.558	1.086
2	15.02	17666.6	851.7	0.314	98.659	0.416
3	17.687	140.1	6.3	0.3431	0.782	0.968

Obtained with ENE-101 from (*Z*)-**1ac**/(*E*)-**1ac** = 3.5/1

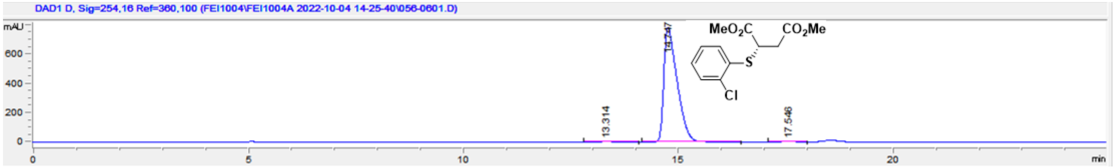

#	Time	Area	Height	Width	Area%	Symmetry
1	13.314	91.4	4.6	0.3072	0.535	0.847
2	14.747	16854	782.4	0.3193	98.747	0.418
3	17.546	122.5	5.6	0.343	0.718	1.002

HPLC analysis of **2ad**: Chiralpak® IG column (4.6 mm × 250 mm, 5 µm); detected at 230 nm; heptane/EtOH = 90/10; flow rate: 1.0 mL/min.

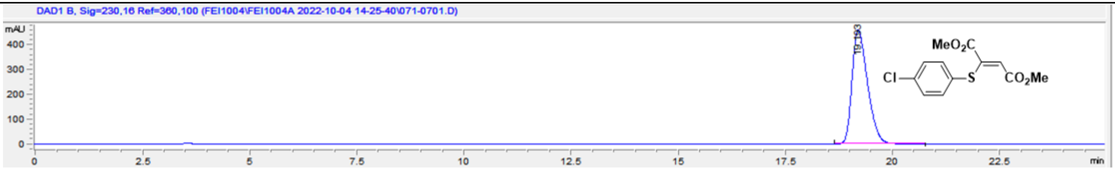

#	Time	Area	Height	Width	Area%	Symmetry
1	19.193	11639.5	460.3	0.391	100.000	0.612

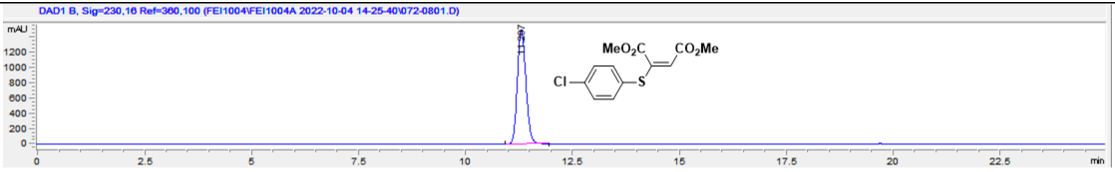

#	Time	Area	Height	Width	Area%	Symmetry
1	11.297	20759.2	1489.4	0.2166	100.000	0.819

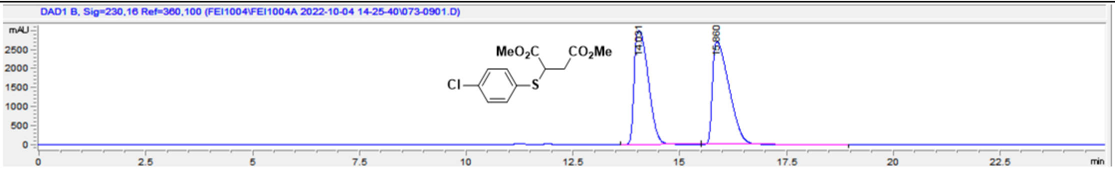

#	Time	Area	Height	Width	Area%	Symmetry
1	14.031	69288.1	3012.6	0.3623	48.420	0.449
2	15.86	73811	2722.3	0.4283	51.580	0.339

Obtained with ENE-101 from (*Z*)-**1ad**

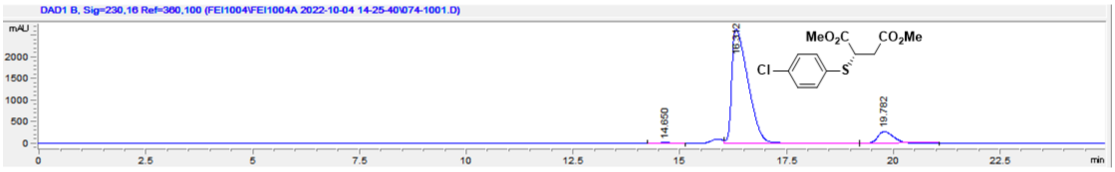

#	Time	Area	Height	Width	Area%	Symmetry
1	14.65	319.6	19.1	0.2593	0.414	0.946
2	16.312	69890.5	2651.2	0.4156	90.450	0.349
3	19.782	7059.3	273.2	0.3995	9.136	0.653

Obtained with ENE-101 from (*E*)-**1ad**

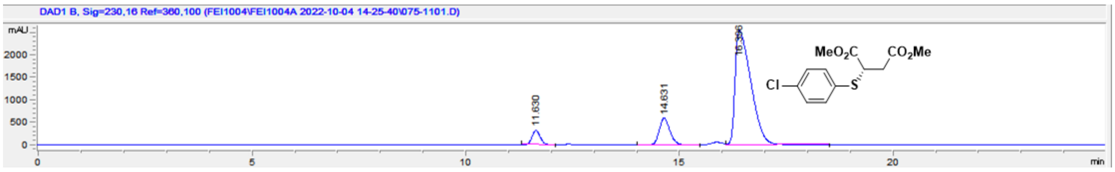

#	Time	Area	Height	Width	Area%	Symmetry
1	11.63	4402.5	322.4	0.2113	5.468	0.858
2	14.631	10340.2	595.1	0.2687	12.844	0.764
3	16.396	65763.7	2549.5	0.4169	81.688	0.364

Obtained with ENE-101 from (*Z*)-**1ad**/(*E*)-**1ad** = 1.2/1

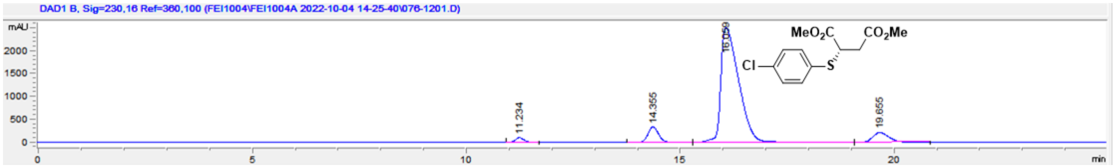

#	Time	Area	Height	Width	Area%	Symmetry
1	11.234	1346.5	99.9	0.2093	1.590	0.856
2	14.355	5732.4	337	0.2644	6.768	0.873
3	16.059	72052.8	2514.3	0.4403	85.076	0.382
4	19.655	5560.3	212.5	0.4033	6.565	0.715

HPLC analysis of **2ae**: Chiralpak® IG column (4.6 mm × 250 mm, 5 μm); detected at 254 nm; heptane/EtOH = 90/10; flow rate: 1.0 mL/min.

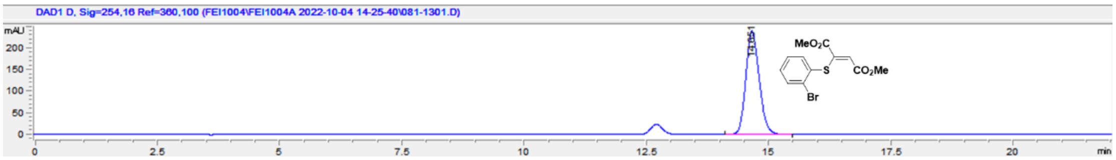

#	Time	Area	Height	Width	Area%	Symmetry
1	14.651	4529.7	240.1	0.2901	100.000	0.799

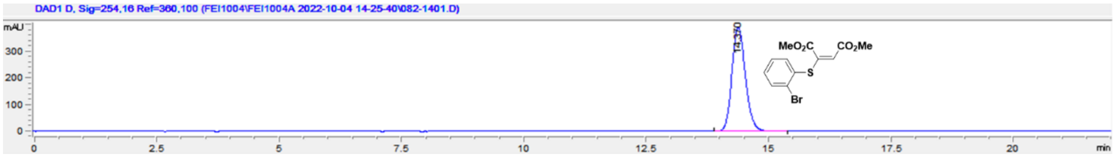

#	Time	Area	Height	Width	Area%	Symmetry
1	14.37	7471.4	390.3	0.2973	100.000	0.818

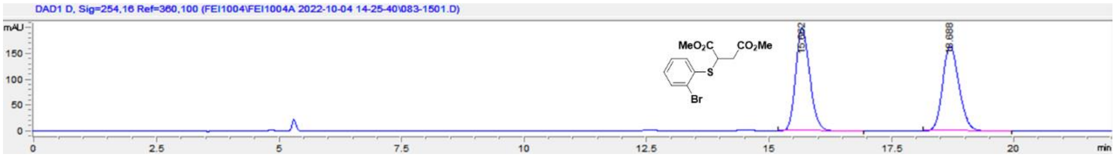

#	Time	Area	Height	Width	Area%	Symmetry
1	15.662	4027	200.6	0.3102	51.118	0.764
2	18.688	3850.8	165.2	0.364	48.882	0.807

Obtained with ENE-101 from (*Z*)-**1ae**

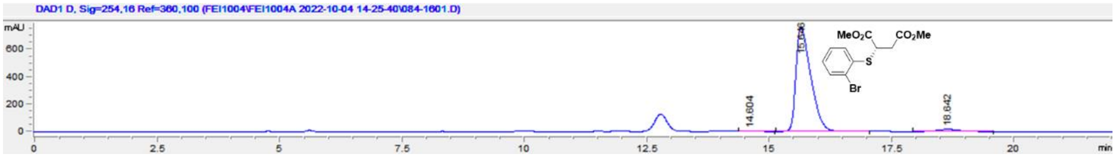

#	Time	Area	Height	Width	Area%	Symmetry
1	14.604	27.6	1.4	0.2939	0.167	0.535
2	15.646	16015	760.3	0.3197	96.838	0.487
3	18.642	495.4	16.1	0.4954	2.996	0.862

Obtained with ENE-101 from (*E*)-**1ae**

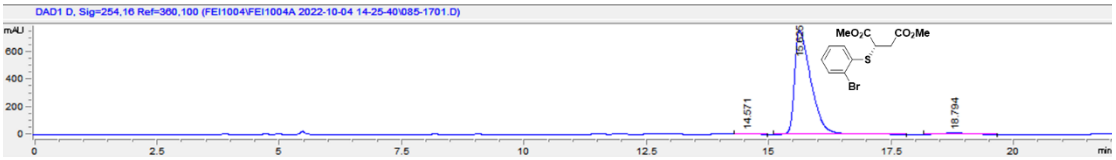

#	Time	Area	Height	Width	Area%	Symmetry
1	14.571	56.4	3.5	0.2518	0.317	0.85
2	15.625	17438	755.5	0.3493	97.954	0.423
3	18.794	307.8	10.5	0.454	1.729	0.945

Obtained with ENE-101 from (*Z*)-**1ae**/(*E*)-**1ae** = 2.7/1

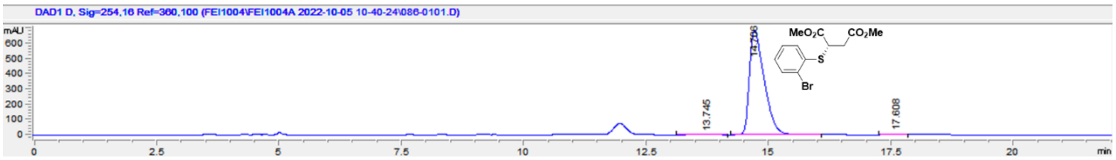

#	Time	Area	Height	Width	Area%	Symmetry
1	13.745	57.6	2.6	0.3218	0.398	1.247
2	14.706	14353	683.2	0.3191	99.269	0.484
3	17.608	48.1	2.9	0.2689	0.333	1.285

HPLC analysis of **2af**: Chiralpak® IG column (4.6 mm × 250 mm, 5 μm); detected at 230 nm; heptane/EtOH = 90/10; flow rate: 1.0 mL/min.

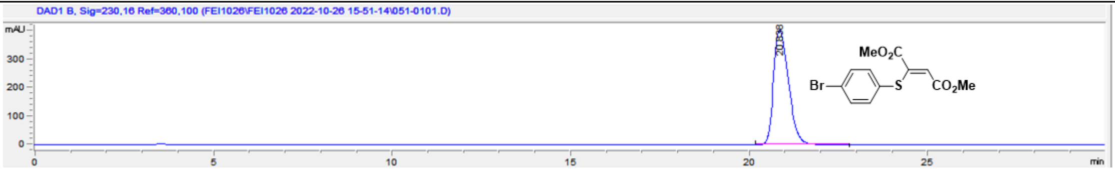

#	Time	Area	Height	Width	Area%	Symmetry
1	20.838	11709.8	402.7	0.4552	100.000	0.646

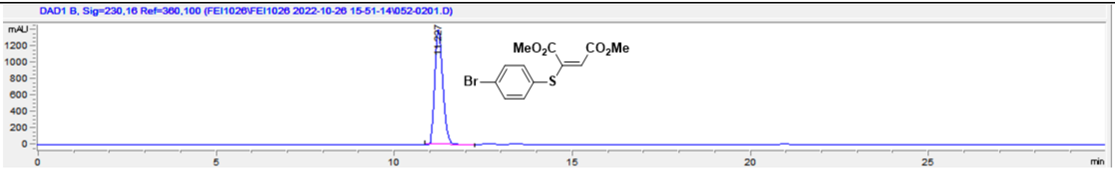

#	Time	Area	Height	Width	Area%	Symmetry
1	11.227	21345.7	1399.2	0.234	100.000	0.749

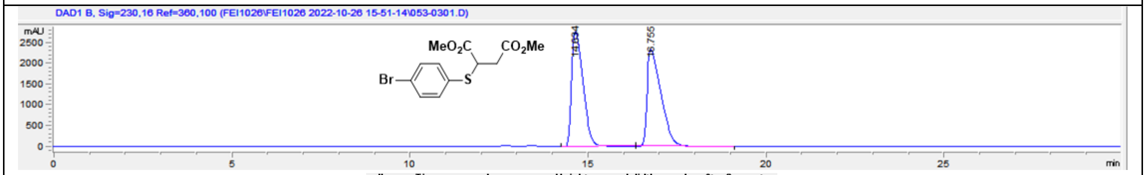

#	Time	Area	Height	Width	Area%	Symmetry
1	14.634	60533.9	2757.5	0.3441	48.690	0.451
2	16.755	63791.6	2332.4	0.4251	51.310	0.369

Obtained with ENE-101 from (*Z*)-**1af**

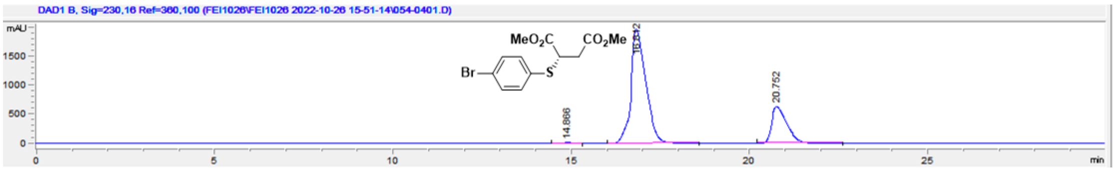

#	Time	Area	Height	Width	Area%	Symmetry
1	14.866	218.4	11.9	0.2879	0.289	0.912
2	16.812	56580.4	1973.7	0.4284	74.892	0.576
3	20.752	18750.2	632.7	0.4637	24.819	0.509

Obtained with ENE-101 from (*E*)-**1af**

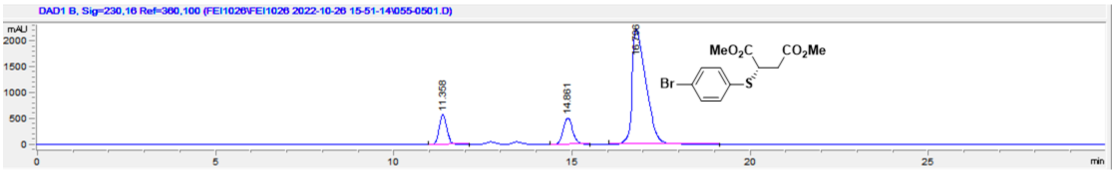

#	Time	Area	Height	Width	Area%	Symmetry
1	11.358	8293.2	575.6	0.2221	10.663	0.852
2	14.861	9587.2	512.4	0.2943	12.326	0.822
3	16.796	59897	2218	0.4051	77.011	0.433

Obtained with ENE-101 from (*Z*)-**1af**/(*E*)-**1af** = 1.3/1

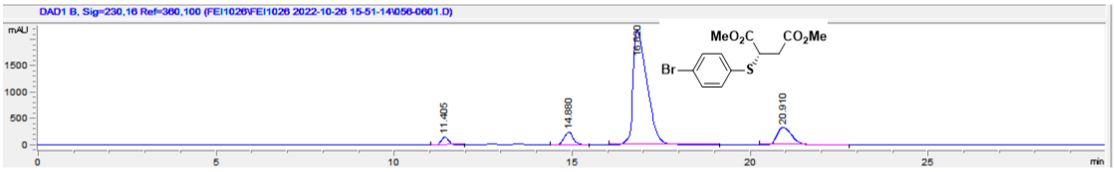

#	Time	Area	Height	Width	Area%	Symmetry
1	11.405	2035	146.2	0.2164	2.725	0.879
2	14.88	4364.9	236.4	0.2854	5.845	0.899
3	16.82	58884.5	2174.8	0.4079	78.851	0.439
4	20.91	9393.6	327.4	0.4427	12.579	0.658

HPLC analysis of **2ag**: Chiralpak® ID column (4.6 mm × 250 mm, 5 μm); detected at 230 nm; heptane/EtOH = 90/10; flow rate: 1.0 mL/min.

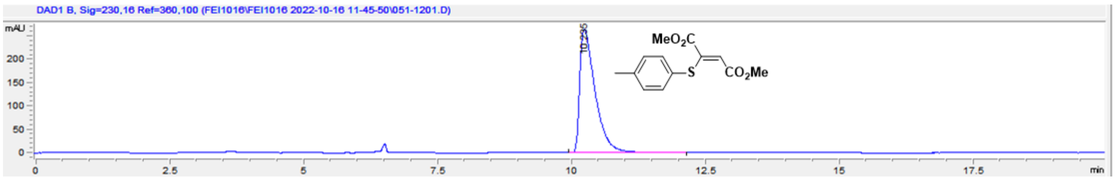

#	Time	Area	Height	Width	Area%	Symmetry
1	10.235	5296.6	264.4	0.2997	100.000	0.384

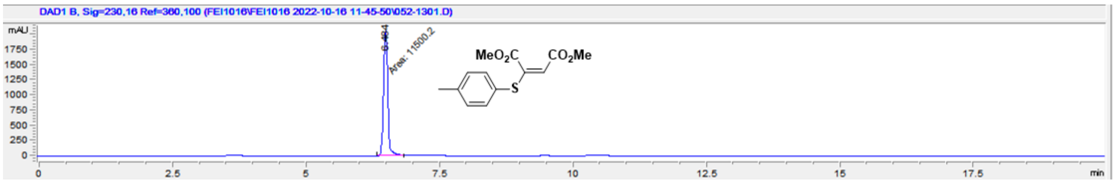

#	Time	Area	Height	Width	Area%	Symmetry
1	6.484	11500.2	2052.3	0.0934	100.000	0.898

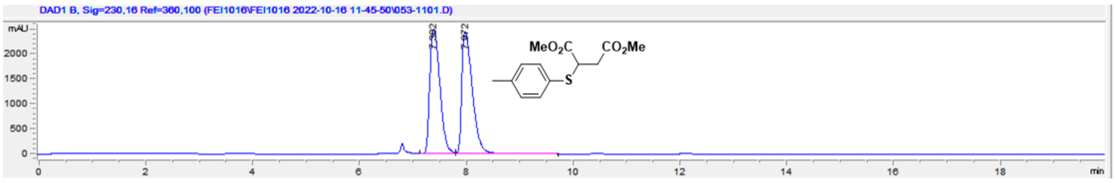

#	Time	Area	Height	Width	Area%	Symmetry
1	7.382	32887.6	2488	0.2082	49.492	0.572
2	7.972	33562.7	2436.4	0.2147	50.508	0.427

Obtained with ENE-101 from (*Z*)-**1ag**

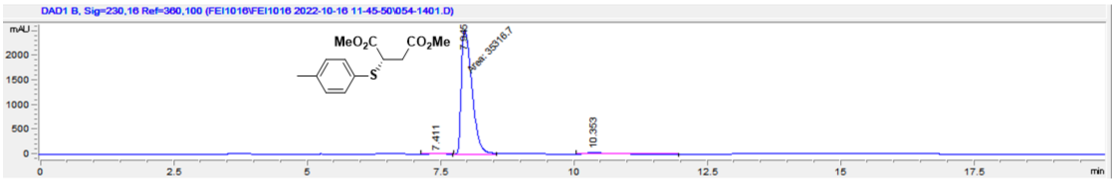

#	Time	Area	Height	Width	Area%	Symmetry
1	7.411	93.7	7.4	0.1926	0.259	0.734
2	7.945	35316.7	2524.2	0.2332	97.773	0.43
3	10.353	710.7	37.7	0.282	1.968	0.529

Obtained with ENE-101 from (*E*)-**1ag**

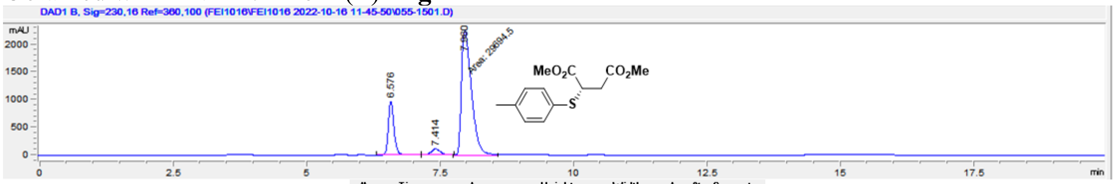

#	Time	Area	Height	Width	Area%	Symmetry
1	6.576	7195	969.5	0.1146	18.820	0.751
2	7.414	1340.5	108.7	0.1899	3.506	0.845
3	7.96	29694.5	2244.9	0.2205	77.673	0.462

Obtained with ENE-101 from (*Z*)-**1ag**/(*E*)-**1ag** = 0.9/1

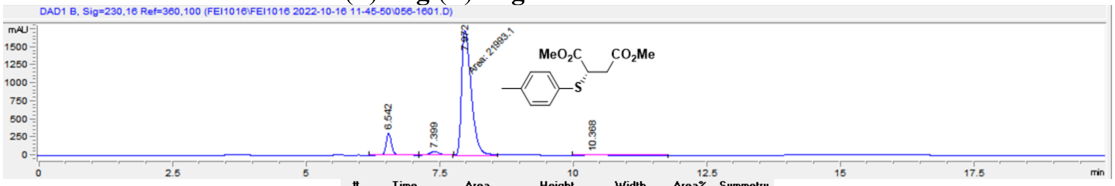

#	Time	Area	Height	Width	Area%	Symmetry
1	6.542	2168.3	304.2	0.1092	8.718	0.813
2	7.399	648	51.3	0.1952	2.605	0.851
3	7.972	21993.1	1732.9	0.2115	88.424	0.522
4	10.368	62.7	3	0.3075	0.252	0.495

HPLC analysis of **2ah**: Chiralpak® IG column (4.6 mm × 250 mm, 5 μm); detected at 230 nm; heptane/EtOH = 90/10; flow rate: 1.0 mL/min.

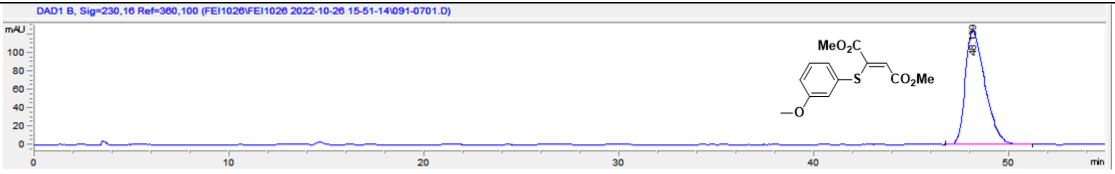

#	Time	Area	Height	Width	Area%	Symmetry
1	48.139	8718.1	124.5	1.0369	100.000	0.584

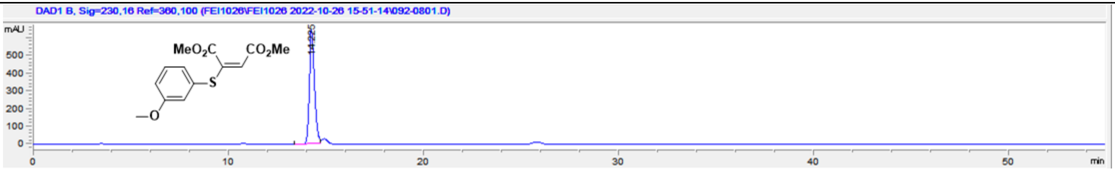

#	Time	Area	Height	Width	Area%	Symmetry
1	14.225	12248.2	645.2	0.2935	100.000	0.768

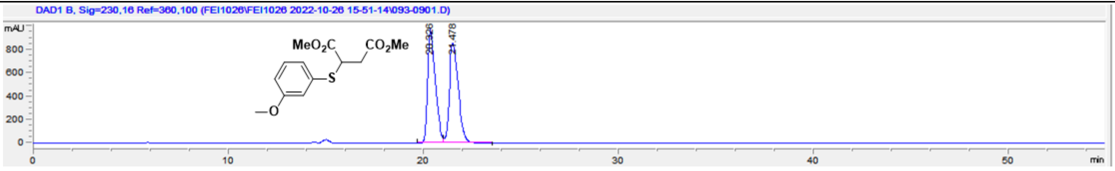

#	Time	Area	Height	Width	Area%	Symmetry
1	20.326	27193.6	974.6	0.4255	50.062	0.531
2	21.478	27126.3	851.2	0.4958	49.938	0.59

Obtained with ENE-101 from (*Z*)-**1ah**

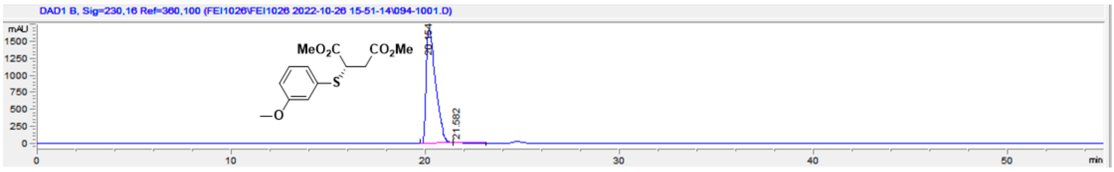

#	Time	Area	Height	Width	Area%	Symmetry
1	20.154	56225.3	1678.1	0.5049	99.280	0.358
2	21.582	407.8	13.1	0.4715	0.720	0.404

Obtained with ENE-101 from (*E*)-**1ah**

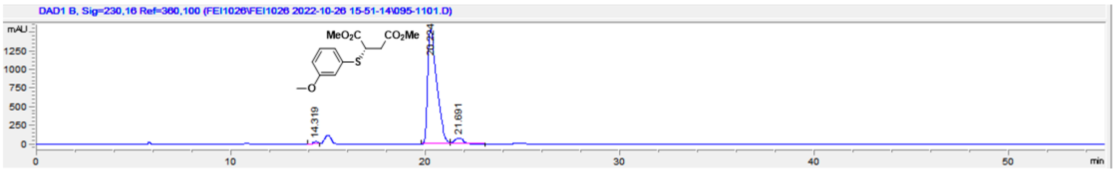

#	Time	Area	Height	Width	Area%	Symmetry
1	14.319	680.7	35.9	0.2915	1.276	0.971
2	20.224	49939.2	1533.8	0.4978	93.634	0.39
3	21.691	2714.8	80.7	0.5187	5.090	0.961

Obtained with ENE-101 from (*Z*)-**1ah**/(*E*)-**1ah** = 2.8/1

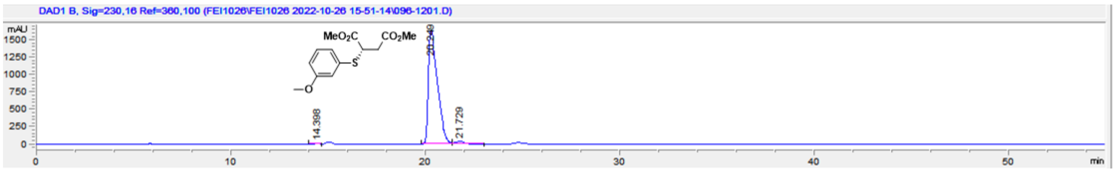

#	Time	Area	Height	Width	Area%	Symmetry
1	14.398	182.8	10	0.2804	0.331	0.937
2	20.249	53637.4	1647.1	0.4859	97.213	0.365
3	21.729	1354.7	40.7	0.506	2.455	0.924

HPLC analysis of **2ai**: Chiralpak® IG column (4.6 mm × 250 mm, 5 μm); detected at 230 nm; heptane/EtOH = 90/10; flow rate: 1.0 mL/min.

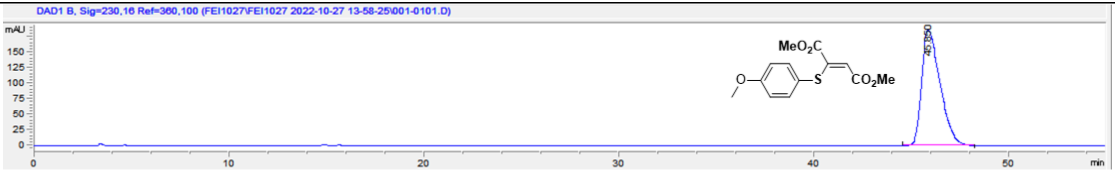

#	Time	Area	Height	Width	Area%	Symmetry
1	45.85	12235.8	185.8	1.0205	100.000	0.531

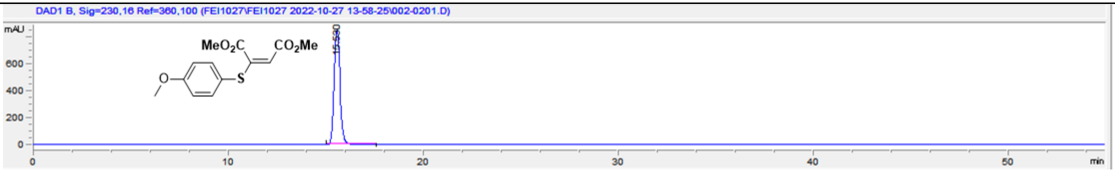

#	Time	Area	Height	Width	Area%	Symmetry
1	15.53	17606.7	850.6	0.3195	100.000	0.762

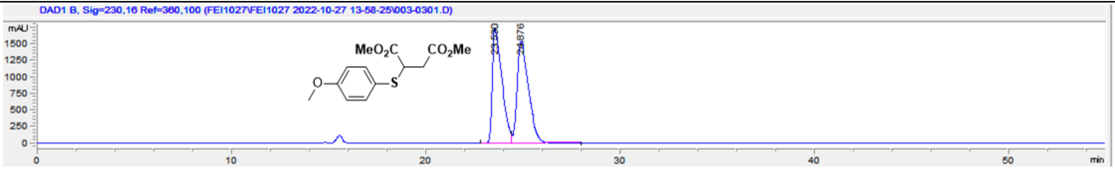

#	Time	Area	Height	Width	Area%	Symmetry
1	23.53	61015.7	1726.6	0.5343	49.454	0.379
2	24.876	62363.7	1544.1	0.6132	50.546	0.48

Obtained with ENE-101 from (*Z*)-**1ai**

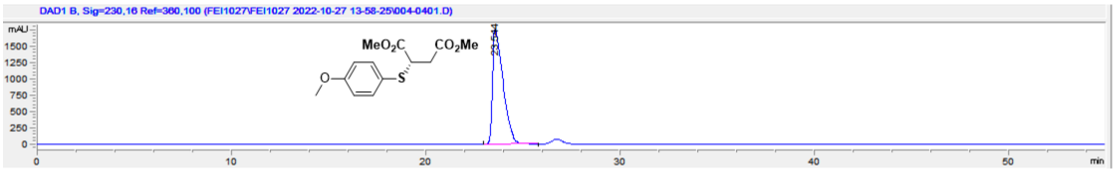

#	Time	Area	Height	Width	Area%	Symmetry
1	23.544	67365	1752.1	0.5706	100.000	0.349

Obtained with ENE-101 from (*E*)-**1ai**

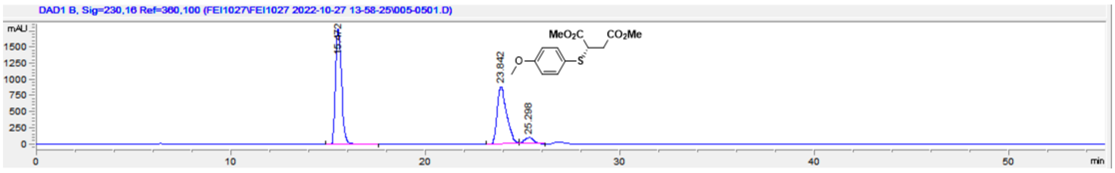

#	Time	Area	Height	Width	Area%	Symmetry
1	15.472	37142.7	1783.7	0.3209	52.417	0.65
2	23.842	30194.3	887.2	0.5171	42.611	0.544
3	25.298	3523.5	98.5	0.5512	4.972	0.958

Obtained with ENE-101 from (*Z*)-**1ai**/(*E*)-**1ai** = 4.4/1

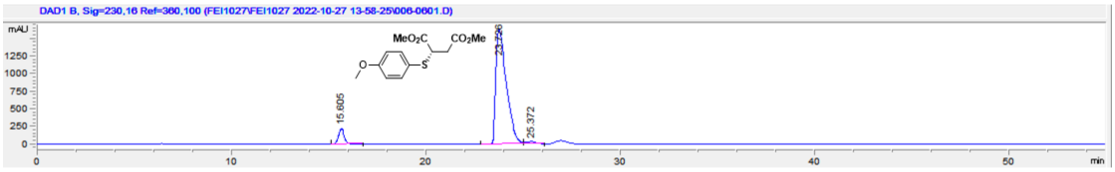

#	Time	Area	Height	Width	Area%	Symmetry
1	15.605	4557.5	222.3	0.3172	6.823	0.867
2	23.726	60911.9	1624	0.5676	91.187	0.375
3	25.372	1329.5	35.9	0.5561	1.990	0.868

HPLC analysis of **2aj**: Chiralcel® OJ-H column (4.6 mm × 250 mm, 5 μm); detected at 230 nm; heptane/EtOH = 90/10; flow rate: 1.0 mL/min.

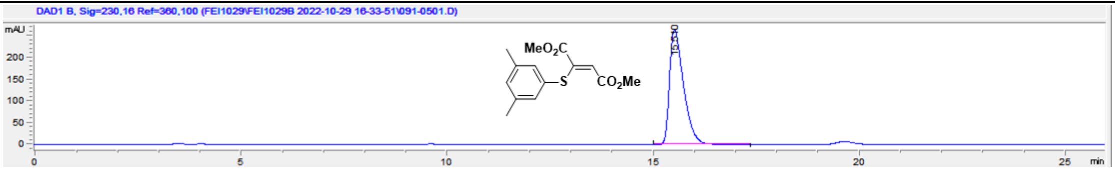

#	Time	Area	Height	Width	Area%	Symmetry
1	15.51	6222.3	262.7	0.3623	100.000	0.51

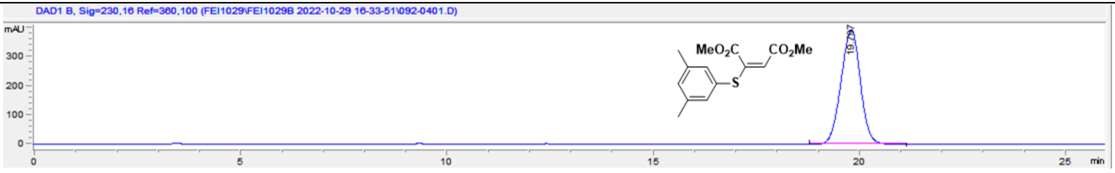

#	Time	Area	Height	Width	Area%	Symmetry
1	19.797	12743.9	391.9	0.5054	100.000	1.087

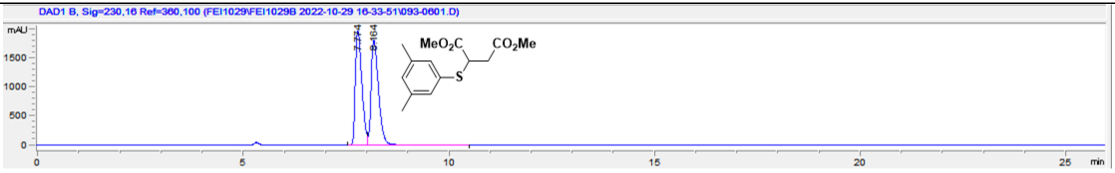

#	Time	Area	Height	Width	Area%	Symmetry
1	7.774	21886.8	1976.2	0.1732	48.955	0.565
2	8.164	22821.6	1814.4	0.1947	51.045	0.529

Obtained with ENE-101 from (*Z*)-**1aj**

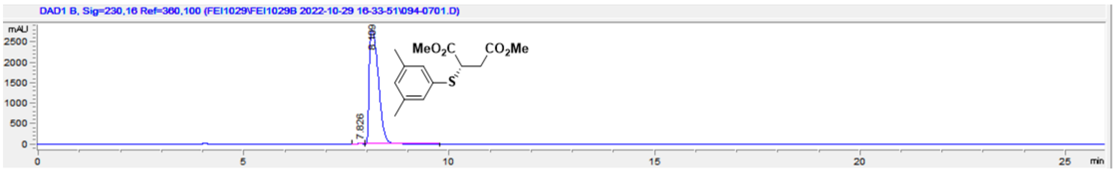

#	Time	Area	Height	Width	Area%	Symmetry
1	7.826	215	24.8	0.1352	0.488	0.96
2	8.109	43851	2802	0.2526	99.512	0.376

Obtained with ENE-101 from (*E*)-**1aj**

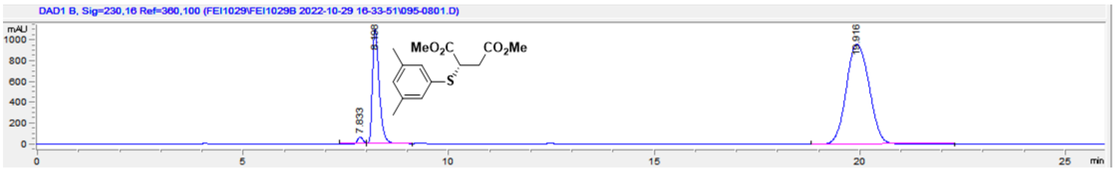

#	Time	Area	Height	Width	Area%	Symmetry
1	7.833	684.9	68.3	0.153	1.352	0.866
2	8.198	12777.1	1099	0.1756	25.229	0.568
3	19.916	37182.9	964.5	0.6198	73.419	0.826

Obtained with ENE-101 from (*Z*)-**1aj**/(*E*)-**1aj** = 4.4/1

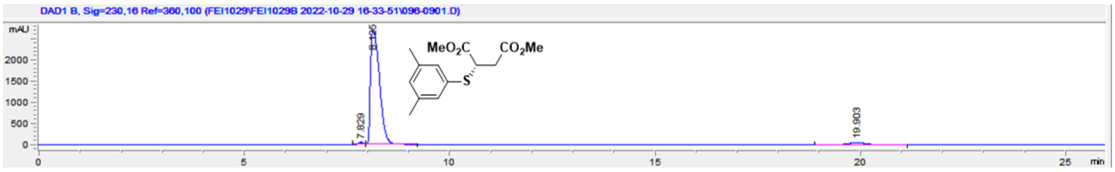

#	Time	Area	Height	Width	Area%	Symmetry
1	7.829	537	60.7	0.1372	1.227	0.883
2	8.125	41688.7	2709.6	0.2475	95.283	0.391
3	19.903	1526.7	49.5	0.4757	3.489	0.891

HPLC analysis of **2ak**: Chiralcel® OD-H column (4.6 mm × 250 mm, 5 μm); detected at 230 nm; Hexane/EtOH = 90/10; flow rate: 1.0 mL/min.

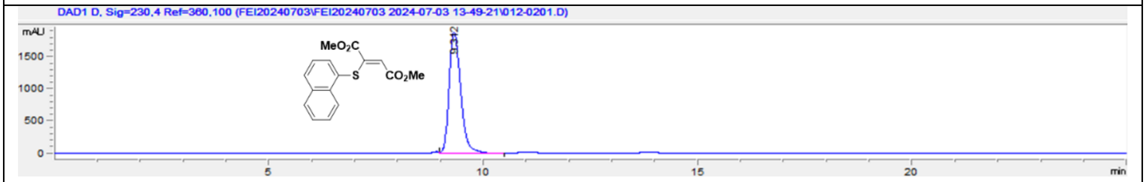

#	Time	Area	Height	Width	Area%	Symmetry
1	9.312	35589.8	1885.5	0.2922	100.000	0.703

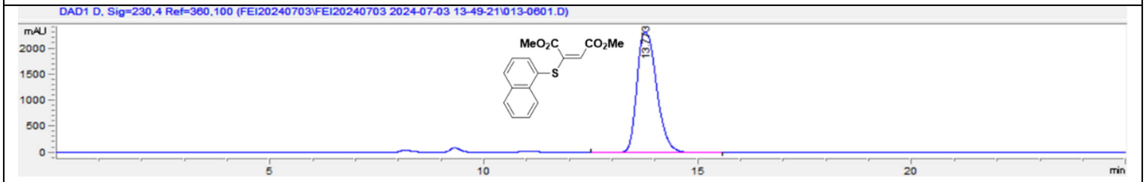

#	Time	Area	Height	Width	Area%	Symmetry
1	13.773	74218.9	2319.9	0.4452	100.000	0.734

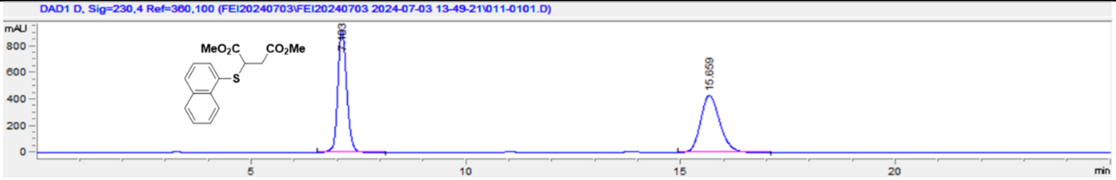

#	Time	Area	Height	Width	Area%	Symmetry
1	7.103	13259.4	926.7	0.2189	49.711	0.811
2	15.659	13413.8	431.6	0.4766	50.289	0.789

Obtained with ENE-101 from (*Z*)-**1ak**

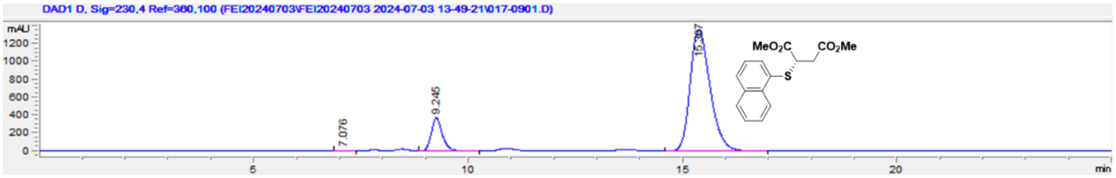

#	Time	Area	Height	Width	Area%	Symmetry
1	7.076	38.2	2.7	0.2223	0.076	0.896
2	9.245	6495.6	368.1	0.2699	12.882	0.751
3	15.367	43888.1	1361.3	0.4981	87.042	0.712

Obtained with ENE-101 from (*E*)-**1ak**

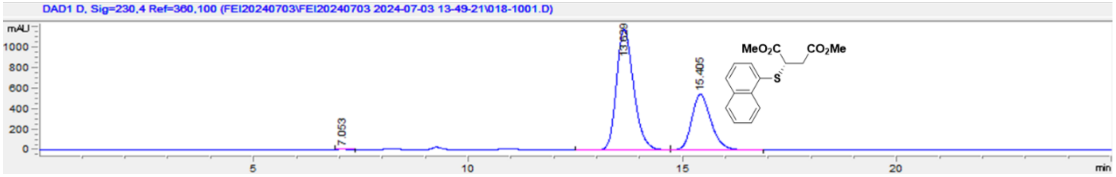

#	Time	Area	Height	Width	Area%	Symmetry
1	7.053	102.4	7.4	0.2089	0.207	0.858
2	13.629	32672.6	1173.6	0.4288	65.994	0.761
3	15.405	16733.6	540.4	0.4753	33.799	0.766

Obtained with ENE-101 from (*Z*)-**1ak**/(*E*)-**1ak** = 0.9/1

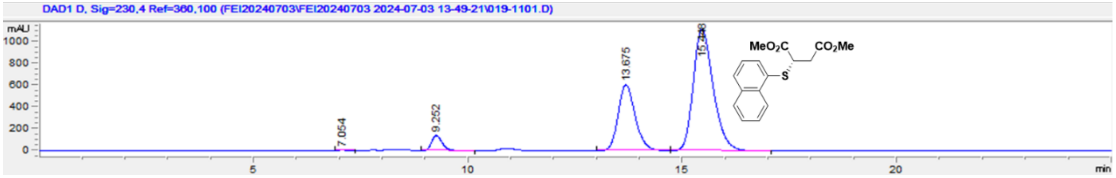

#	Time	Area	Height	Width	Area%	Symmetry
1	7.054	73.1	5.3	0.2023	0.132	0.852
2	9.252	2447.9	139.8	0.2663	4.415	0.751
3	13.675	16738.8	605.1	0.4227	30.190	0.785
4	15.448	36184.5	1115.2	0.4985	65.263	0.726

HPLC analysis of **2al**: Chiralpak® ID column (4.6 mm × 250 mm, 5 µm); detected at 230 nm; heptane/EtOH = 90/10; flow rate: 1.0 mL/min.

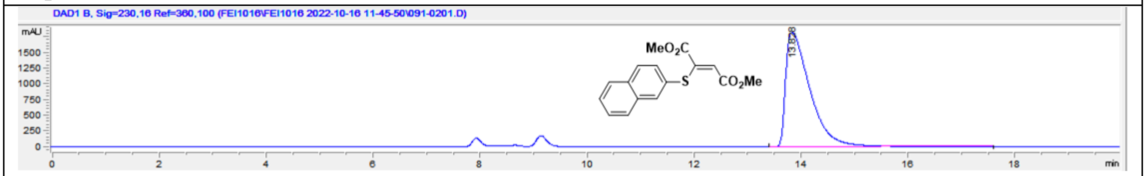

#	Time	Area	Height	Width	Area%	Symmetry
1	13.828	57613	1822.7	0.4768	100.000	0.326

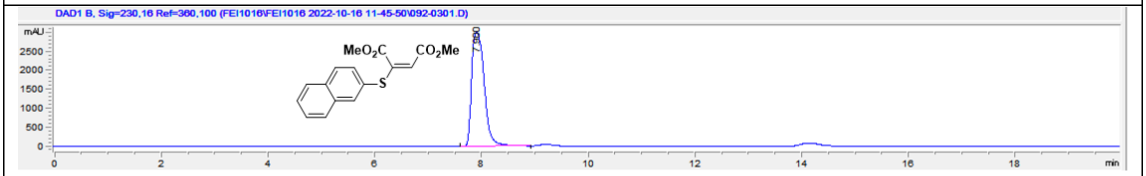

#	Time	Area	Height	Width	Area%	Symmetry
1	7.9	49722.4	3009.3	0.2668	100.000	0.589

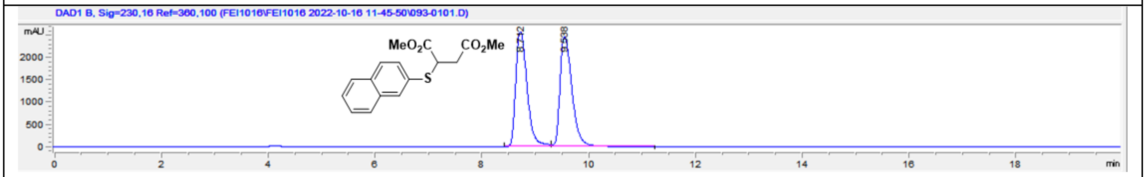

#	Time	Area	Height	Width	Area%	Symmetry
1	8.712	38401.1	2582.3	0.2315	49.593	0.628
2	9.538	39031.6	2476.8	0.2439	50.407	0.557

Obtained with ENE-101 from (*Z*)-**1al**

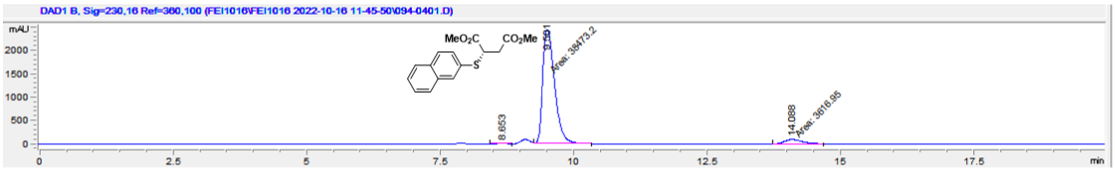

#	Time	Area	Height	Width	Area%	Symmetry
1	8.653	228.7	16.3	0.222	0.540	0.931
2	9.501	38473.2	2447.5	0.262	90.913	0.573
3	14.088	3617	118.5	0.5089	8.547	0.636

Obtained with ENE-101 from (*E*)-**1al**

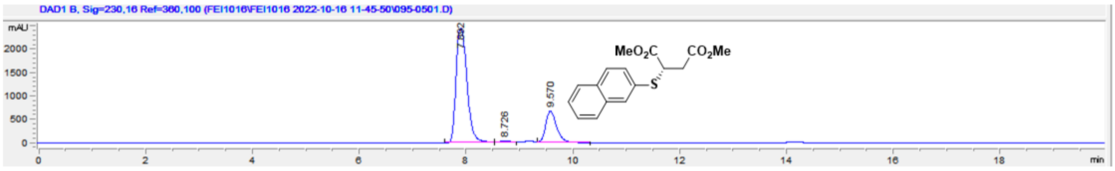

#	Time	Area	Height	Width	Area%	Symmetry
1	7.892	34993	2449.1	0.2247	77.924	0.674
2	8.726	403.4	31.8	0.1958	0.898	1.108
3	9.57	9510.1	673	0.2169	21.178	0.692

Obtained with ENE-101 from (*Z*)-**1al**/(*E*)-**1al** = 1.1/1

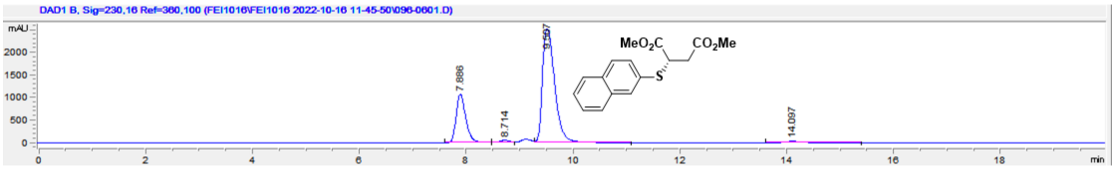

#	Time	Area	Height	Width	Area%	Symmetry
1	7.886	13441.1	1071.6	0.1923	24.431	0.724
2	8.714	613.4	49	0.196	1.115	0.88
3	9.507	40068.1	2513.8	0.248	72.829	0.549
4	14.097	894.2	34.6	0.3916	1.625	0.652

HPLC analysis of **2am**: Chiralpak® IG column (4.6 mm × 250 mm, 5 μm); detected at 214 nm; heptane/EtOH = 90/10; flow rate: 1.0 mL/min.

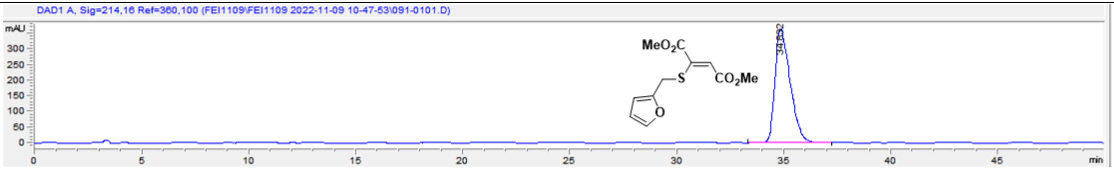

#	Time	Area	Height	Width	Area%	Symmetry
1	34.832	17988	363.1	0.77	100.000	0.555

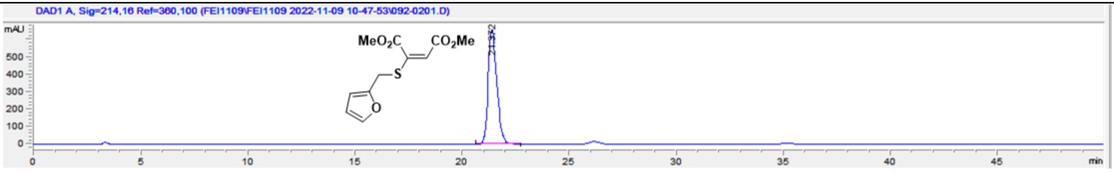

#	Time	Area	Height	Width	Area%	Symmetry
1	21.372	18445.8	654.2	0.4349	100.000	0.714

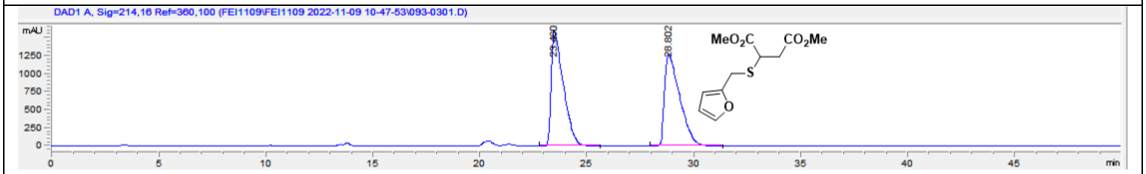

#	Time	Area	Height	Width	Area%	Symmetry
1	23.46	62667.6	1588.6	0.5802	50.075	0.311
2	28.802	62479.5	1266.1	0.7377	49.925	0.334

Obtained with ENE-101 from (*Z*)-**1am**

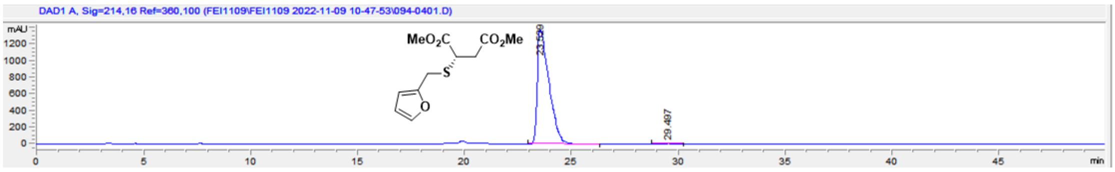

#	Time	Area	Height	Width	Area%	Symmetry
1	23.529	52673.8	1371.6	0.582	99.584	0.338
2	29.497	219.9	5.8	0.5331	0.416	0.919

Obtained with ENE-101 from (*E*)-**1am**

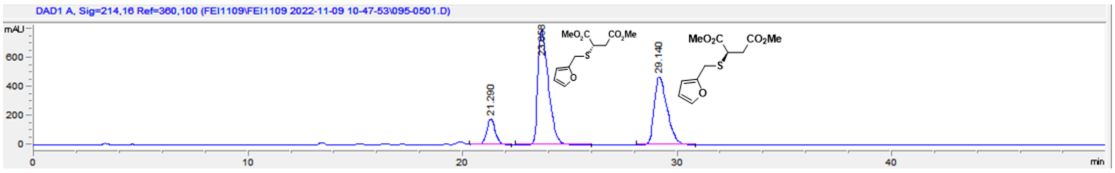

#	Time	Area	Height	Width	Area%	Symmetry
1	21.29	4911.1	175.7	0.4342	9.520	0.904
2	23.658	27092.8	791.5	0.5234	52.519	0.495
3	29.14	19582.4	468.2	0.642	37.960	0.584

Obtained with ENE-101 from (*Z*)-**1am**/(*E*)-**1am** = 0.4/1

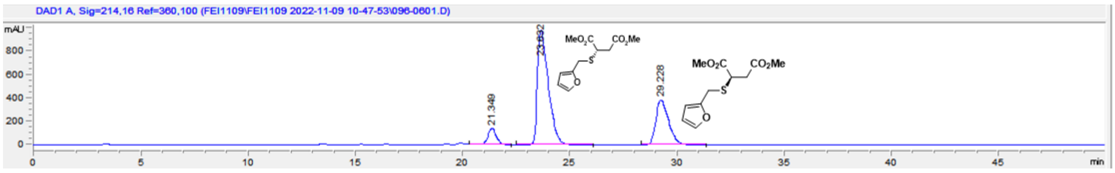

#	Time	Area	Height	Width	Area%	Symmetry
1	21.349	3781.6	136.5	0.4273	6.989	0.942
2	23.632	34507	981.5	0.5362	63.775	0.431
3	29.228	15819.1	383.2	0.6396	29.236	0.627

HPLC analysis of **2an**: Chiralpak® IG column (4.6 mm × 250 mm, 5 µm); detected at 230 nm; heptane/EtOH = 90/10; flow rate: 1.0 mL/min.

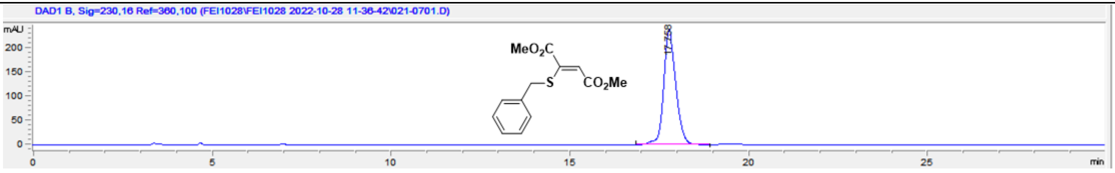

#	Time	Area	Height	Width	Area%	Symmetry
1	17.758	5696.1	236.8	0.3686	100.000	0.777

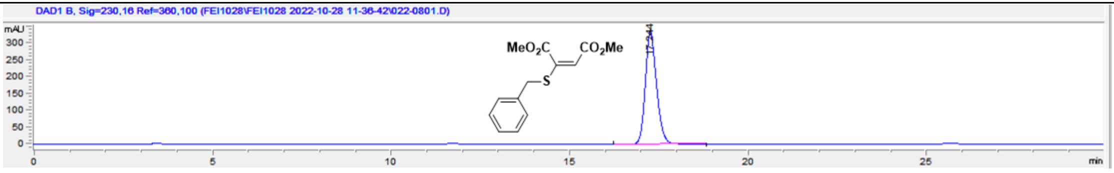

#	Time	Area	Height	Width	Area%	Symmetry
1	17.244	7524.2	339.4	0.3426	100.000	0.758

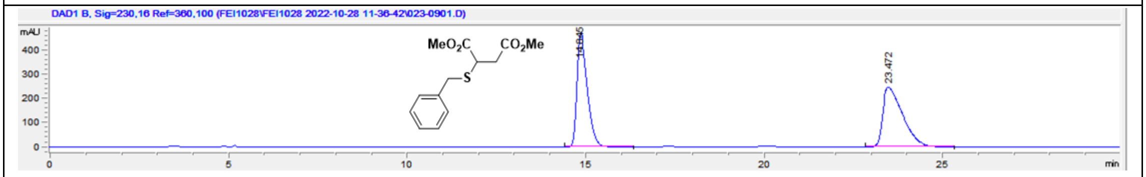

#	Time	Area	Height	Width	Area%	Symmetry
1	14.845	9644.6	474.8	0.309	50.058	0.532
2	23.472	9622.2	249.4	0.5701	49.942	0.394

Obtained with ENE-101 from (*Z*)-**1an**

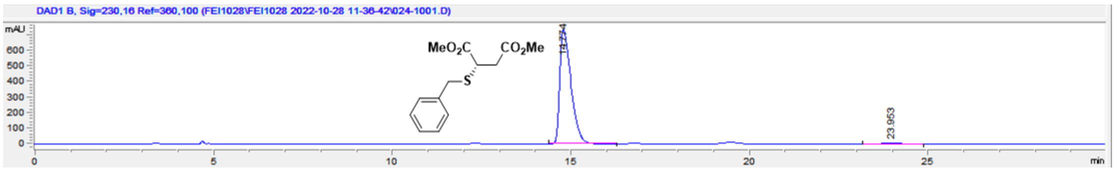

#	Time	Area	Height	Width	Area%	Symmetry
1	14.774	15978.3	732.3	0.3285	98.288	0.427
2	23.953	278.3	8.5	0.4979	1.712	0.878

Obtained with ENE-101 from (*E*)-**1an**

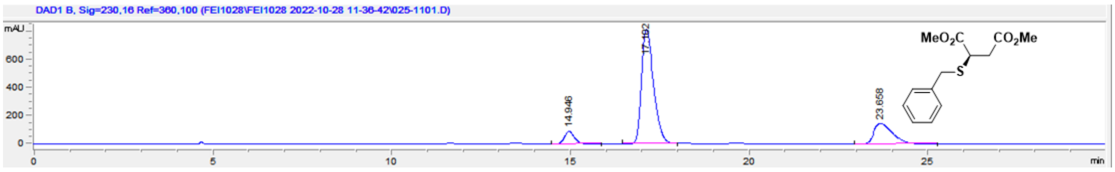

#	Time	Area	Height	Width	Area%	Symmetry
1	14.946	1775.9	91.9	0.3016	6.810	0.818
2	17.102	19042.5	813.7	0.361	73.021	0.583
3	23.658	5259.6	146.3	0.5533	20.169	0.513

HPLC analysis of **2ao**: Chiralpak® IG column (4.6 mm × 250 mm, 5 µm); detected at 214 nm; heptane/EtOH = 90/10; flow rate: 1.0 mL/min.

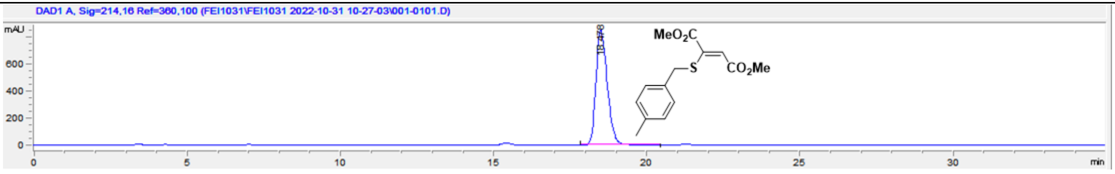

#	Time	Area	Height	Width	Area%	Symmetry
1	18.478	22142.6	853.5	0.4047	100.000	0.687

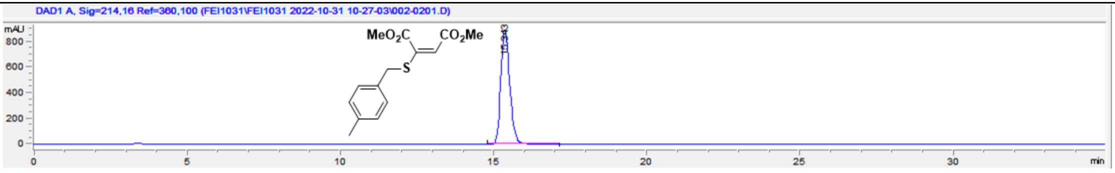

#	Time	Area	Height	Width	Area%	Symmetry
1	15.343	18272.1	891.3	0.3192	100.000	0.72

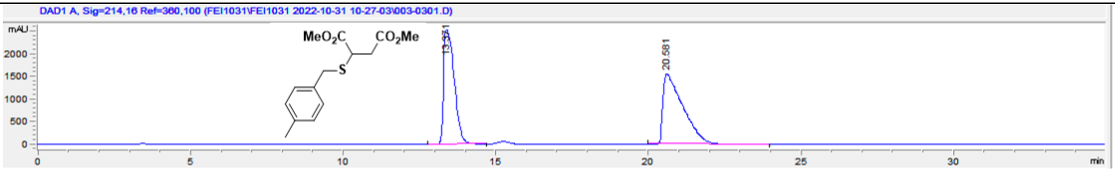

#	Time	Area	Height	Width	Area%	Symmetry
1	13.371	62256.8	2533.1	0.3927	46.803	0.371
2	20.581	70760.8	1561.3	0.6347	53.197	0.205

Obtained with ENE-101 from (*Z*)-**1ao**

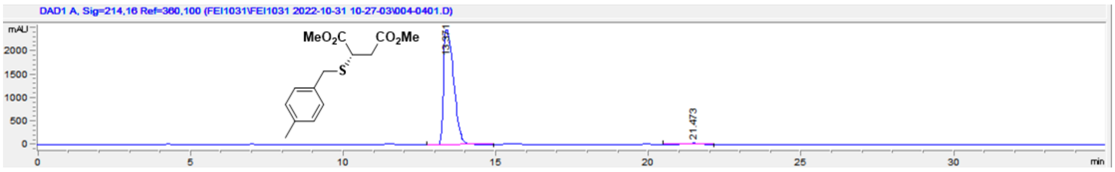

#	Time	Area	Height	Width	Area%	Symmetry
1	13.371	57420.5	2450.2	0.3754	97.944	0.407
2	21.473	1205.2	31.1	0.5647	2.056	1.462

Obtained with ENE-101 from (*E*)-**1ao**

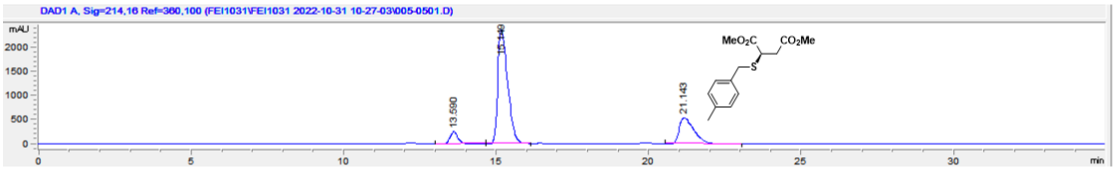

#	Time	Area	Height	Width	Area%	Symmetry
1	13.59	4370.1	252.3	0.2681	5.716	0.843
2	15.149	54033.7	2364.3	0.3546	70.671	0.499
3	21.143	18054	542.6	0.5022	23.613	0.456

HPLC analysis of **2ap**: Chiralpak® IG column (4.6 mm × 250 mm, 5 μm); detected at 214 nm; heptane/EtOH = 90/10; flow rate: 1.0 mL/min.

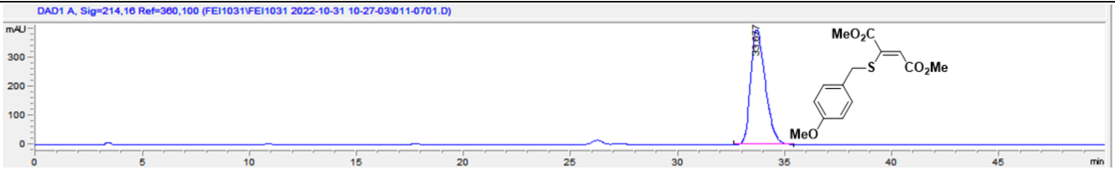

#	Time	Area	Height	Width	Area%	Symmetry
1	33.677	18874.8	395.7	0.7406	100.000	0.663

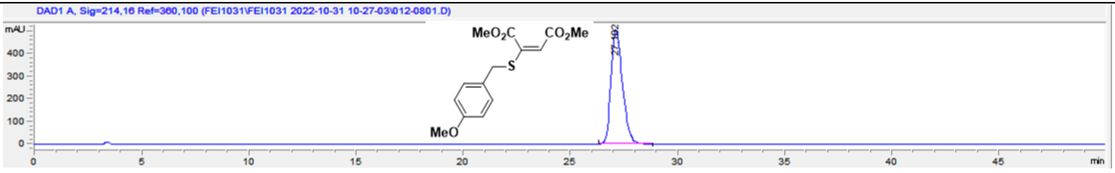

#	Time	Area	Height	Width	Area%	Symmetry
1	27.102	18880.2	505	0.5742	100.000	0.667

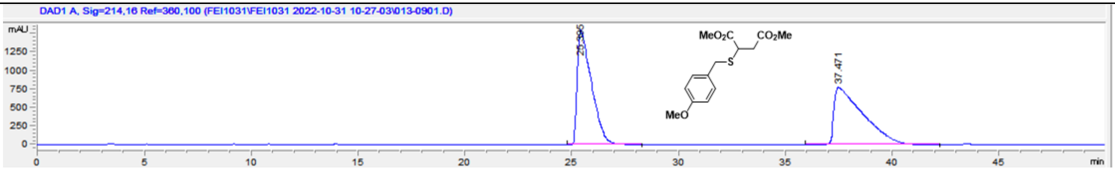

#	Time	Area	Height	Width	Area%	Symmetry
1	25.395	71816.8	1548.7	0.687	49.459	0.283
2	37.471	73386.8	775.1	1.2406	50.541	0.173

Obtained with ENE-101 from (*Z*)-**1ap**

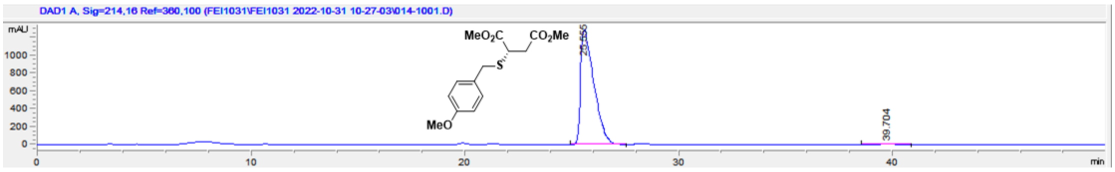

#	Time	Area	Height	Width	Area%	Symmetry
1	25.555	55692.4	1283.8	0.6481	99.228	0.342
2	39.704	433.6	8.3	0.6886	0.772	0.941

Obtained with ENE-101 from (*E*)-**1ap**

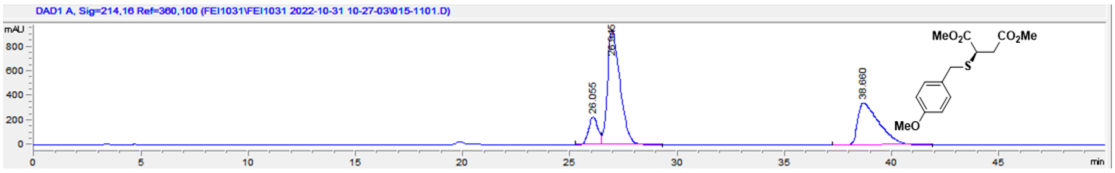

#	Time	Area	Height	Width	Area%	Symmetry
1	26.055	6994.5	225.4	0.4841	10.203	0.871
2	26.945	38010.7	934.5	0.6106	55.448	0.538
3	38.66	23546.3	340.6	1.0286	34.348	0.327

HPLC analysis of **2aq**: Chiralpak® IG column (4.6 mm × 250 mm, 5 μm); detected at 214 nm; heptane/EtOH = 90/10; flow rate: 1.0 mL/min.

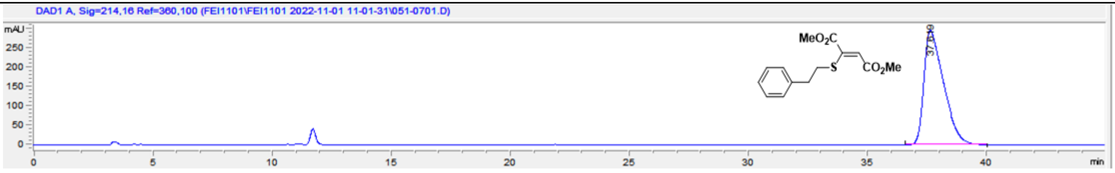

#	Time	Area	Height	Width	Area%	Symmetry
1	37.619	17025.3	296.7	0.8735	100.000	0.463

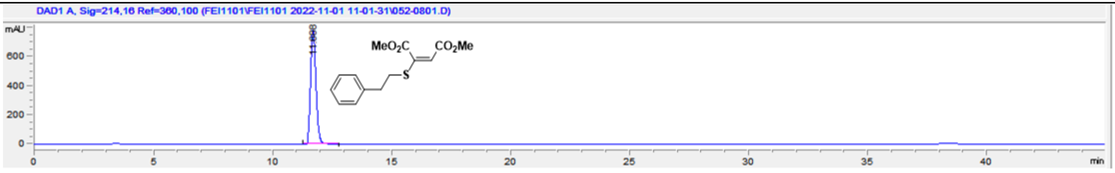

#	Time	Area	Height	Width	Area%	Symmetry
1	11.688	11917.3	785.1	0.2351	100.000	0.794

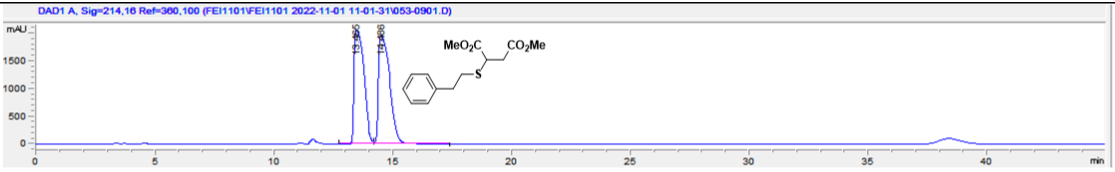

#	Time	Area	Height	Width	Area%	Symmetry
1	13.465	58115.3	2063.2	0.4646	47.282	0.32
2	14.486	64796.3	1960.2	0.5436	52.718	0.273

Obtained with ENE-101 from (*Z*)-**1aq**

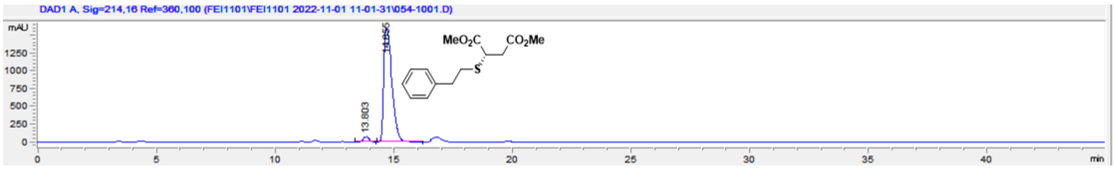

#	Time	Area	Height	Width	Area%	Symmetry
1	13.803	1289.3	75.7	0.2648	3.173	0.918
2	14.655	39341.6	1603.3	0.3903	96.827	0.409

Obtained with ENE-101 from (*E*)-**1aq**

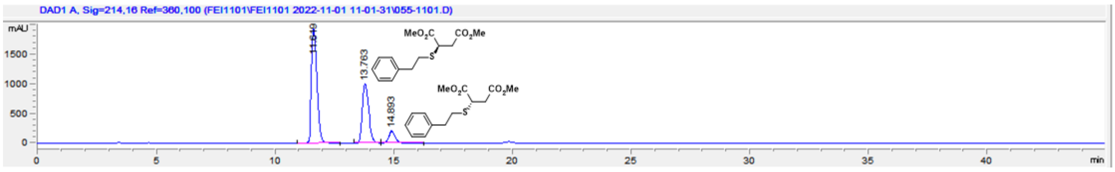

#	Time	Area	Height	Width	Area%	Symmetry
1	11.619	31353.4	1928.5	0.2537	57.401	0.682
2	13.763	19045.4	1004	0.2933	34.868	0.644
3	14.893	4223.3	209.9	0.3107	7.732	0.815

HPLC analysis of **2ar**: Chiralpak® IG column (4.6 mm × 250 mm, 5 μm); detected at 214 nm; hexane/*i*-PrOH = 90/10; flow rate: 1.0 mL/min.

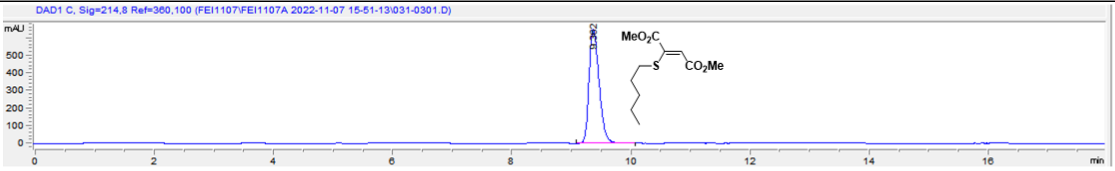

#	Time	Area	Height	Width	Area%	Symmetry
1	9.362	7629.6	650.2	0.1809	100.000	0.675

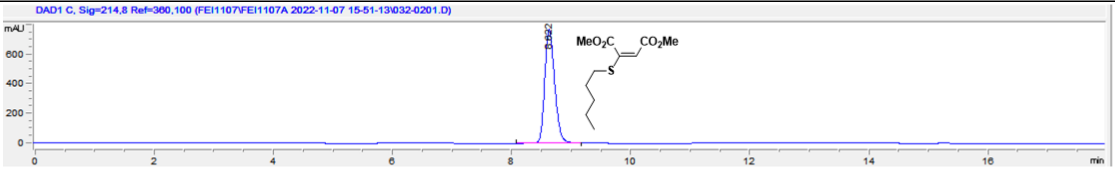

#	Time	Area	Height	Width	Area%	Symmetry
1	8.622	8926.8	771.9	0.1789	100.000	0.72

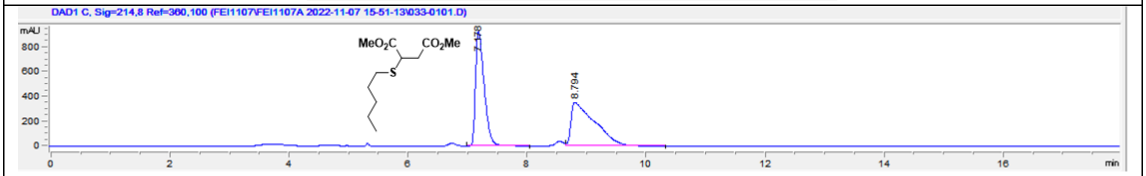

#	Time	Area	Height	Width	Area%	Symmetry
1	7.178	9326.8	928.4	0.1552	48.646	0.479
2	8.794	9845.8	352.9	0.3895	51.354	0.174

Obtained with ENE-101 from (*Z*)-**1ar**

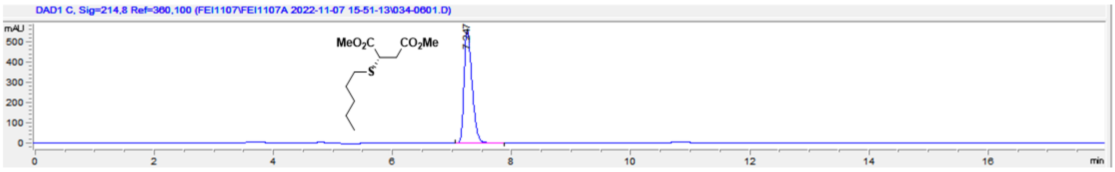

#	Time	Area	Height	Width	Area%	Symmetry
1	7.247	5212.4	564.7	0.1417	100.000	0.599

Obtained with ENE-101 from (*E*)-**1ar**

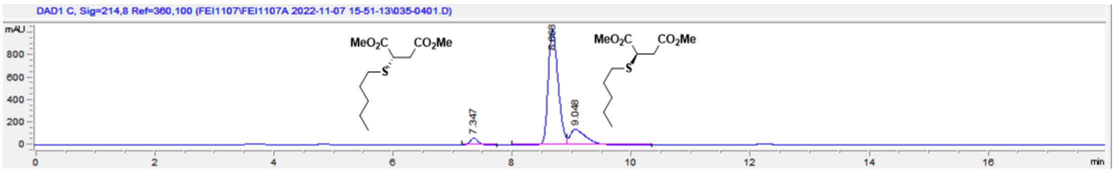

#	Time	Area	Height	Width	Area%	Symmetry
1	7.347	513.3	59.6	0.1345	3.443	0.815
2	8.668	11874.9	1021.9	0.1816	79.655	0.674
3	9.048	2519.7	139.1	0.2594	16.902	0.389

HPLC analysis of **2as**: Chiralpak® IC column (4.6 mm × 250 mm, 5 μm); detected at 214 nm; heptane/EtOH = 90/10; flow rate: 1.0 mL/min.

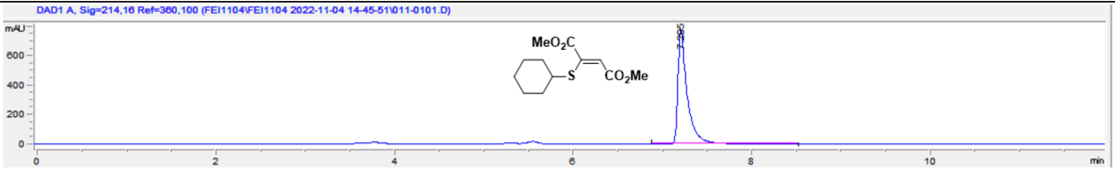

#	Time	Area	Height	Width	Area%	Symmetry
1	7.205	5599.1	781.4	0.1016	100.000	0.413

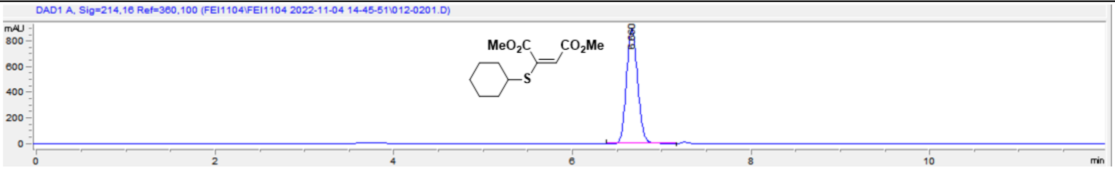

#	Time	Area	Height	Width	Area%	Symmetry
1	6.66	7802.9	895.3	0.1357	100.000	0.851

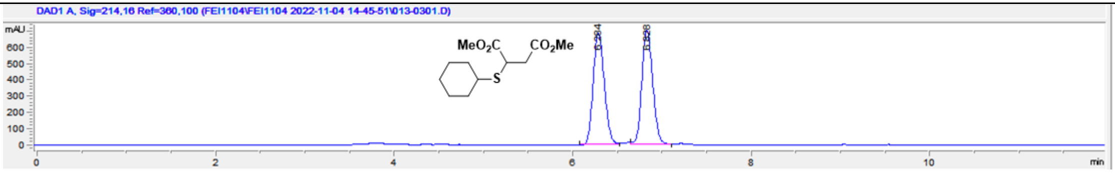

#	Time	Area	Height	Width	Area%	Symmetry
1	6.284	5952.6	689.4	0.1367	50.143	0.812
2	6.828	5918.8	704.1	0.1321	49.857	0.82

Obtained with ENE-101 from (*Z*)-**1as**

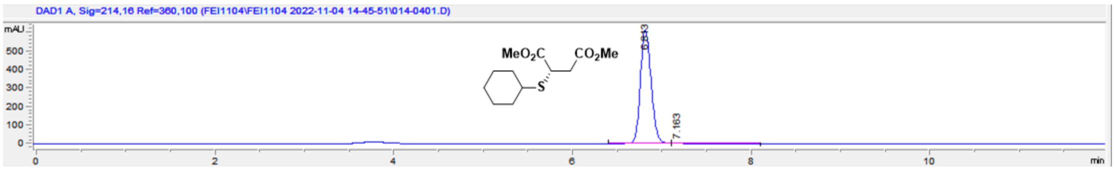

#	Time	Area	Height	Width	Area%	Symmetry
1	6.813	5145.3	614.7	0.1317	98.911	0.811
2	7.163	56.6	4.9	0.1555	1.089	0.276

Obtained with ENE-101 from (*E*)-**1as**

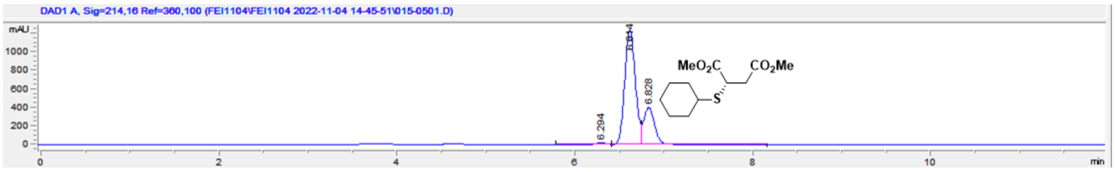

#	Time	Area	Height	Width	Area%	Symmetry
1	6.294	172.3	19.7	0.1361	1.244	1.111
2	6.614	10231.9	1235.7	0.1306	73.900	0.865
3	6.828	3441.4	401.1	0.1301	24.856	0.743

Obtained with ENE-101 from (*Z*)-**1as**/(*E*)-**1as** = 1.1/1

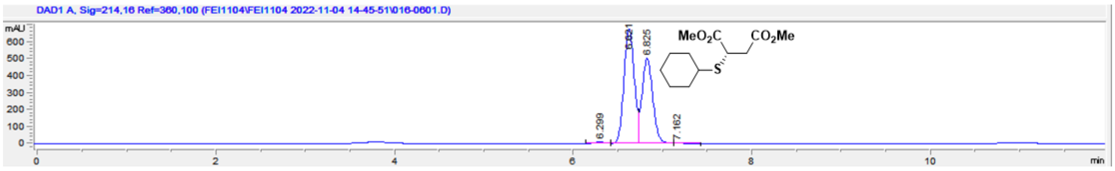

#	Time	Area	Height	Width	Area%	Symmetry
1	6.299	62.1	8.3	0.1194	0.645	1.089
2	6.621	5381.8	673.1	0.1253	55.956	0.925
3	6.825	4153.8	501	0.1267	43.187	0.824
4	7.162	20.4	3.7	0.0828	0.212	0.451

HPLC analysis of **2at**: Chiralpak® IG column (4.6 mm × 250 mm, 5 μm); detected at 230 nm; heptane/EtOH = 90/10; flow rate: 1.0 mL/min.

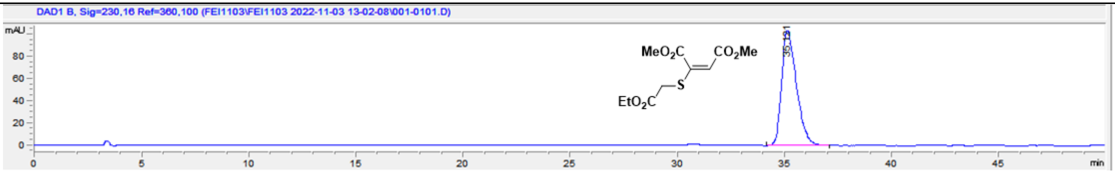

#	Time	Area	Height	Width	Area%	Symmetry
1	35.121	4977.8	103.4	0.7358	100.000	0.603

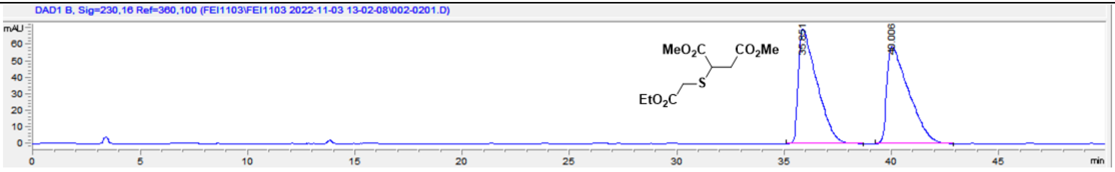

#	Time	Area	Height	Width	Area%	Symmetry
1	35.851	4174.1	69.4	0.8281	50.056	0.297
2	40.006	4164.8	59.1	0.9664	49.944	0.284

Obtained with ENE-101 from (*E*)-**1at**

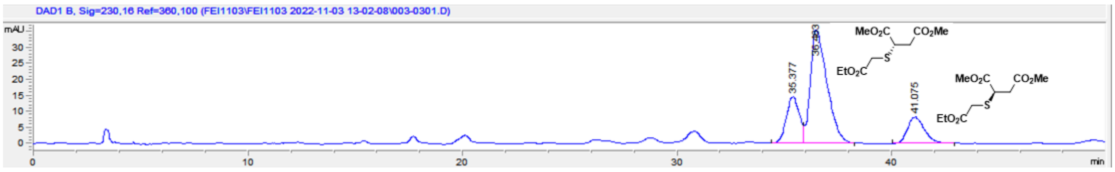

#	Time	Area	Height	Width	Area%	Symmetry
1	35.377	619.2	14.4	0.6568	20.875	0.943
2	36.463	1900.9	35.4	0.7885	64.082	0.501
3	41.075	446.2	8.2	0.7737	15.043	0.754

HPLC analysis of **2au**: Chiralcel® OJ-H column (4.6 mm × 250 mm, 5 μm); detected at 230 nm; heptane/EtOH = 90/10; flow rate: 1.0 mL/min.

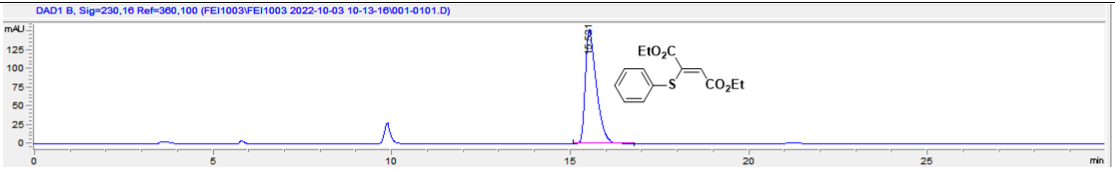

#	Time	Area	Height	Width	Area%	Symmetry
1	15.521	3340.4	152.5	0.3375	100.000	0.539

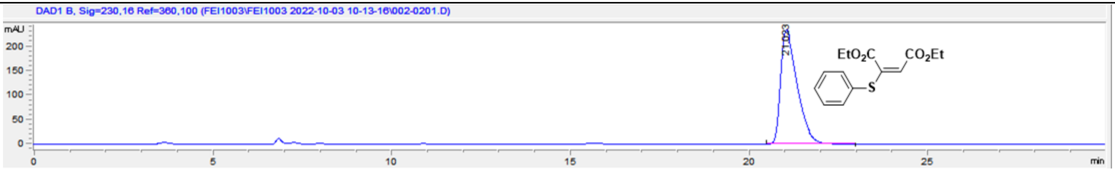

#	Time	Area	Height	Width	Area%	Symmetry
1	21.023	7516.8	233.4	0.4957	100.000	0.473

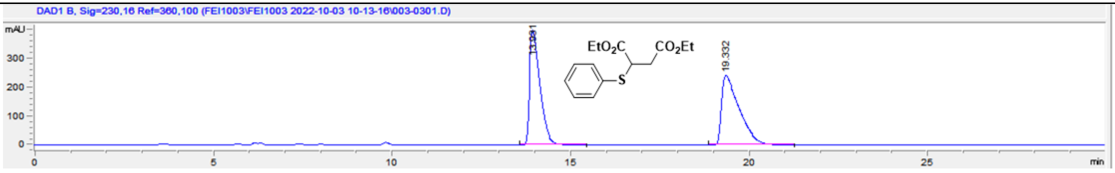

#	Time	Area	Height	Width	Area%	Symmetry
1	13.931	8116.9	399.7	0.3049	49.499	0.444
2	19.332	8281.1	240.3	0.502	50.501	0.288

Obtained with ENE-101 from (*Z*)-**1au**

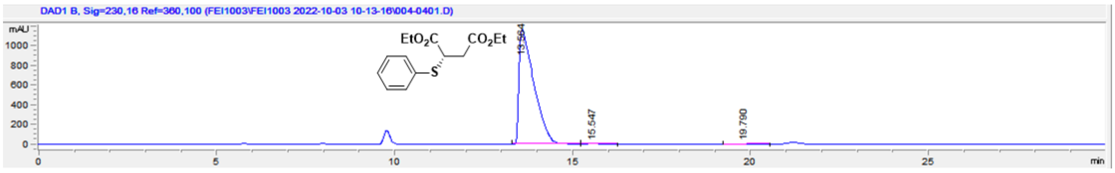

#	Time	Area	Height	Width	Area%	Symmetry
1	13.564	33657.2	1162.5	0.4118	99.067	0.223
2	15.547	195.3	8.7	0.3371	0.575	0.885
3	19.79	121.7	4.9	0.3721	0.358	0.794

Obtained with ENE-101 from (*E*)-**1au**

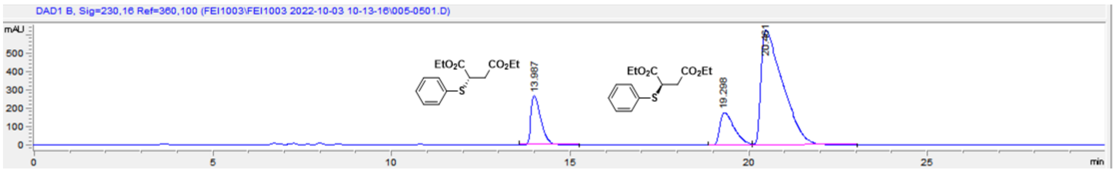

#	Time	Area	Height	Width	Area%	Symmetry
1	13.987	5112.9	267.8	0.2907	14.067	0.519
2	19.298	4874.4	176.4	0.4205	13.411	0.4
3	20.461	26359.2	626.8	0.5986	72.522	0.259

Obtained with ENE-101 from (*Z*)-**1au**/(*E*)-**1au** = 4/1

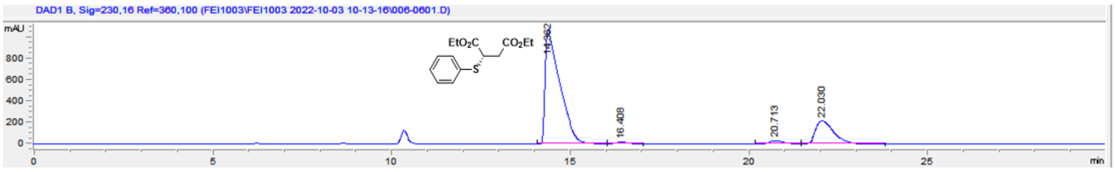

#	Time	Area	Height	Width	Area%	Symmetry
1	14.362	31019.5	1074.9	0.4107	78.082	0.237
2	16.408	340.1	16	0.3246	0.856	0.762
3	20.713	842.5	31.3	0.4081	2.121	0.706
4	22.03	7524.7	215.1	0.5422	18.941	0.507

HPLC analysis of **2av**: Chiralpak® IC column (4.6 mm × 250 mm, 5 μm); detected at 254 nm; heptane/EtOH = 90/10; flow rate: 1.0 mL/min.

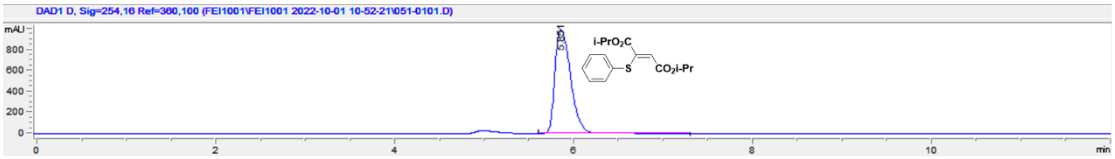

#	Time	Area	Height	Width	Area%	Symmetry
1	5.851	12131.8	992.4	0.1946	100.000	0.548

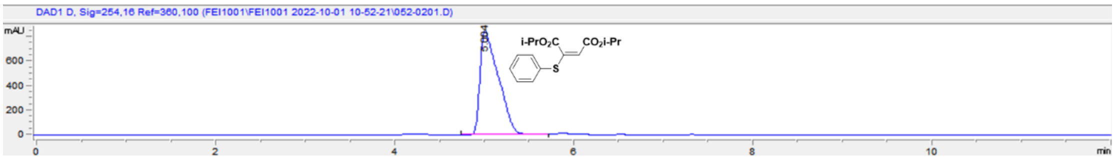

#	Time	Area	Height	Width	Area%	Symmetry
1	5.004	12090.2	848.4	0.2043	100.000	0.347

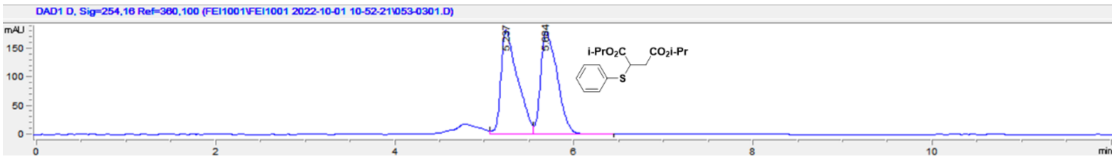

#	Time	Area	Height	Width	Area%	Symmetry
1	5.237	2363.8	182.8	0.1808	50.377	0.414
2	5.684	2328.4	179.8	0.2111	49.623	0.465

Obtained with ENE-101 from (*Z*)-**1av**

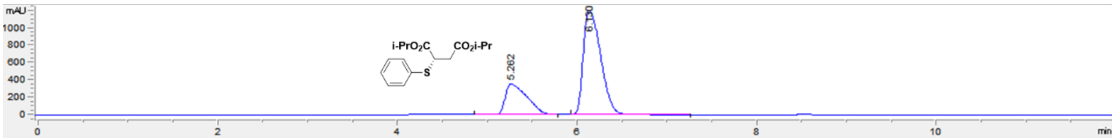

#	Time	Area	Height	Width	Area%	Symmetry
1	5.262	5913.3	353.8	0.285	26.665	0.362
2	6.13	16263.3	1197.9	0.2184	73.335	0.496

Obtained with ENE-101 from (*E*)-**1av**

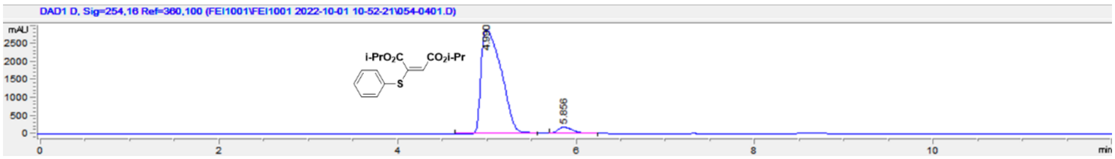

#	Time	Area	Height	Width	Area%	Symmetry
1	4.99	45416.4	2888.3	0.2674	95.637	0.335
2	5.856	2071.8	174.5	0.1845	4.363	0.634

Note: In this case, ^{1}H NMR analysis of the crude reaction mixture also confirmed there was no desired product in this reaction.

HPLC analysis of **2aw**: Chiralpak® IG column (4.6 mm × 250 mm, 5 µm); detected at 254 nm; heptane/EtOH = 90/10; flow rate: 1.0 mL/min.

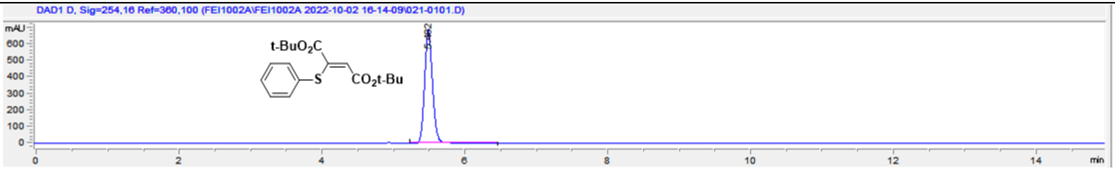

#	Time	Area	Height	Width	Area%	Symmetry
1	5.482	5274	690.8	0.1191	100.000	0.838

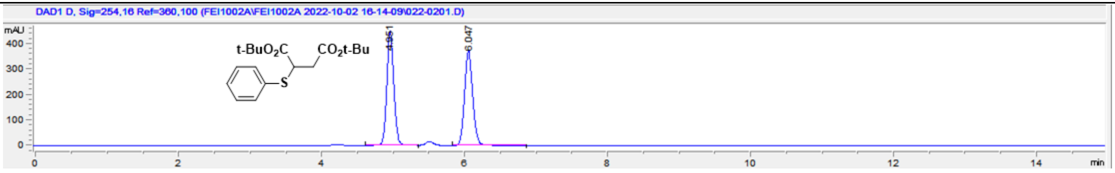

#	Time	Area	Height	Width	Area%	Symmetry
1	4.951	3110.4	450.6	0.1065	51.175	0.876
2	6.047	2967.5	373.7	0.1226	48.825	0.849

Obtained with ENE-101 from (*Z*)-**1aw**

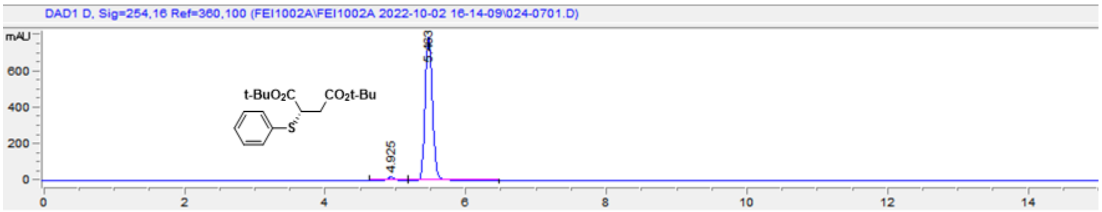

#	Time	Area	Height	Width	Area%	Symmetry
1	4.925	138.6	18.5	0.1133	2.359	1.17
2	5.463	5739.8	787.6	0.113	97.641	0.818

HPLC analysis of **2ax**: Chiralpak® IG column (4.6 mm × 250 mm, 5 µm); detected at 254 nm; heptane/EtOH = 70/30; flow rate: 1.0 mL/min.

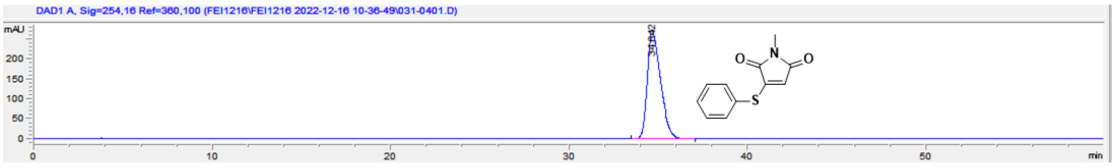

#	Time	Area	Height	Width	Area%	Symmetry
1	34.642	14120	274.3	0.7825	100.000	0.59

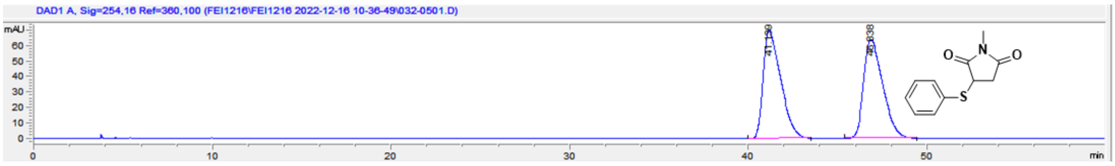

#	Time	Area	Height	Width	Area%	Symmetry
1	41.139	4782.8	70.5	0.991	50.019	0.482
2	46.838	4779.3	64.3	1.1218	49.981	0.575

Obtained with ENE-101 from (*E*)-**1ax**

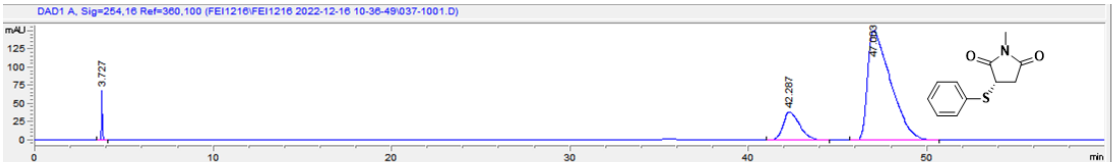

#	Time	Area	Height	Width	Area%	Symmetry
1	3.727	390.2	68	0.089	2.390	0.845
2	42.287	2472.8	38.4	0.951	15.145	0.649
3	47.003	13464.4	151.2	1.2488	82.465	0.343

HPLC analysis of **2ay**: Chiralpak® IG column (4.6 mm × 250 mm, 5 µm); detected at 214 nm; heptane/EtOH = 70/30; flow rate: 1.0 mL/min.

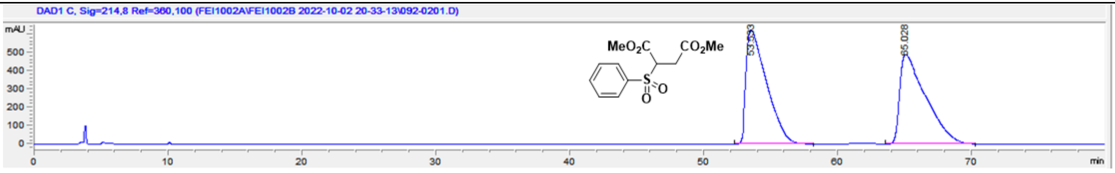

#	Time	Area	Height	Width	Area%	Symmetry
1	53.533	66260.9	623.4	1.52	49.915	0.32
2	65.028	66487.4	486.8	1.8715	50.085	0.269

Obtained with ENE-101 from (*Z*)-**1ay**

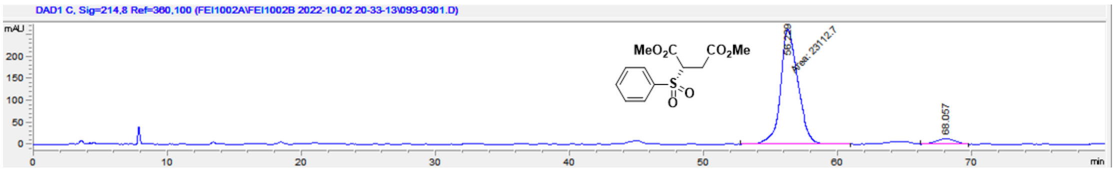

#	Time	Area	Height	Width	Area%	Symmetry
1	56.229	23112.7	261.3	1.4744	94.952	0.693
2	68.057	1228.7	12.3	1.1846	5.048	1.103

Note: In this case, the HPLC spectrum of substrate (*Z*)-**1ay** was not provided because it was not stable and decomposed under the HPLC conditions. This maybe because the nucleophilic addition of EtOH in the eluent to the carbon-carbon double bond in the substrate. However, the full conversion of the substrate to the desired product was also confirmed by ^{1}H NMR analysis of the crude reaction mixture.

HPLC analysis of **2az**: Chiralpak® IG column (4.6 mm × 250 mm, 5 μm); detected at 214 nm; heptane/EtOH = 70/30; flow rate: 1.0 mL/min.

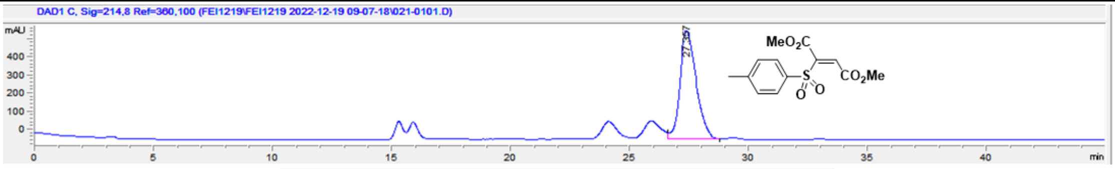

#	Time	Area	Height	Width	Area%	Symmetry
1	27.357	28351.8	600.3	0.725	100.000	0.644

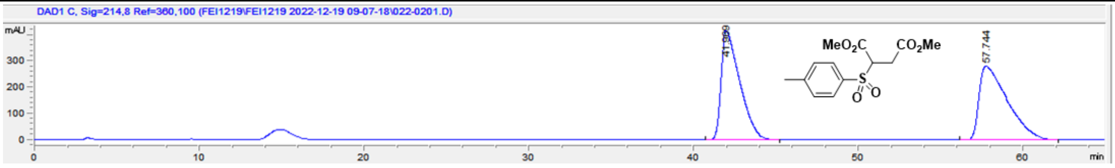

#	Time	Area	Height	Width	Area%	Symmetry
1	41.969	33243.6	418	1.1498	49.931	0.403
2	57.744	33335.4	280.9	1.6405	50.069	0.315

Obtained with ENE-101 from (*Z*)-**1az**

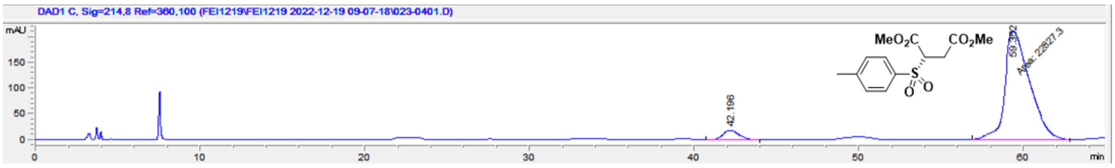

#	Time	Area	Height	Width	Area%	Symmetry
1	42.196	1278.5	18.4	0.8339	5.304	0.931
2	59.352	22827.3	214.3	1.7749	94.696	0.525

Note: In this case, the full conversion of the substrate to the desired product was also confirmed by ^{1}H NMR analysis of the crude reaction mixture.

HPLC analysis of **2ba**: Chiralpak® IC column (4.6 mm × 250 mm, 5 μm); detected at 230 nm; heptane/EtOH = 90/10; flow rate: 1.0 mL/min.

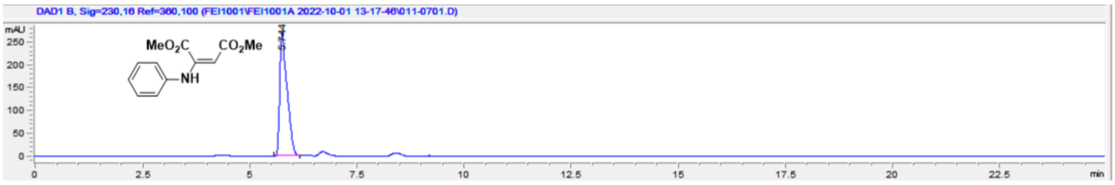

#	Time	Area	Height	Width	Area%	Symmetry
1	5.744	3240.5	275.1	0.1634	100.000	0.408

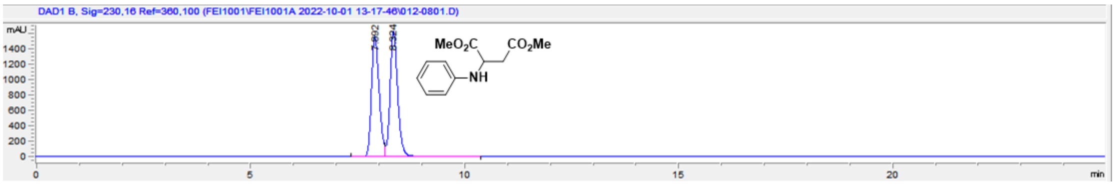

#	Time	Area	Height	Width	Area%	Symmetry
1	7.892	19410.4	1564.5	0.1907	48.917	0.769
2	8.324	20270.2	1631.4	0.191	51.083	0.746

Obtained with ENE-101 from (*E*)-**1ba**

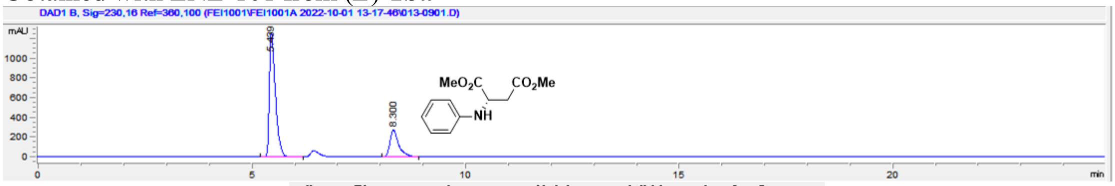

#	Time	Area	Height	Width	Area%	Symmetry
1	5.439	12416.2	1267.3	0.1423	75.911	0.447
2	8.3	3940	273.8	0.2139	24.089	0.612

HPLC analysis of **2bb**: Chiralpak® IG column (4.6 mm × 250 mm, 5 μm); detected at 230 nm; heptane/EtOH = 90/10; flow rate: 1.0 mL/min.

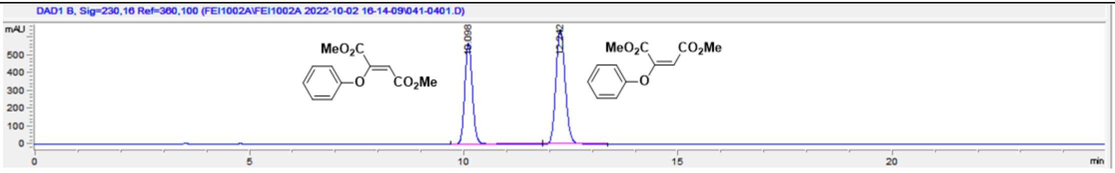

#	Time	Area	Height	Width	Area%	Symmetry
1	10.098	7033.8	570	0.192	41.851	0.859
2	12.242	9773	642.8	0.2374	58.149	0.839

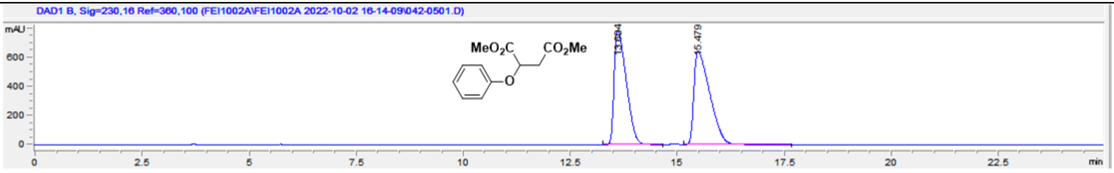

#	Time	Area	Height	Width	Area%	Symmetry
1	13.604	15611.6	788.6	0.311	48.452	0.413
2	15.479	16609.4	641.3	0.4042	51.548	0.344

Obtained with ENE-101 from (*Z*)-**1bb**/(*E*)-**1bb** = 0.8/1

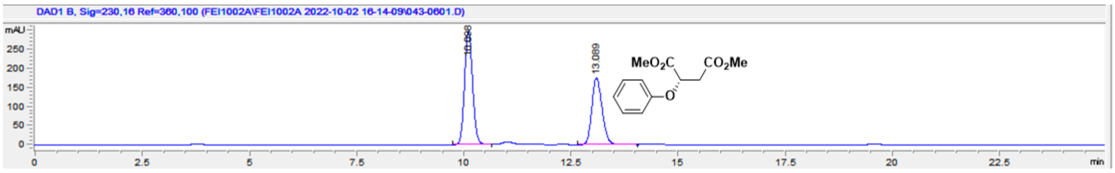

#	Time	Area	Height	Width	Area%	Symmetry
1	10.098	3861.7	297.9	0.2012	57.064	0.867
2	13.089	2905.5	174.5	0.2602	42.936	0.769

HPLC analysis of **2bc**: Chiralpak® IG column (4.6 mm × 250 mm, 5 μm); detected at 214 nm; heptane/EtOH = 90/10; flow rate: 1.0 mL/min.

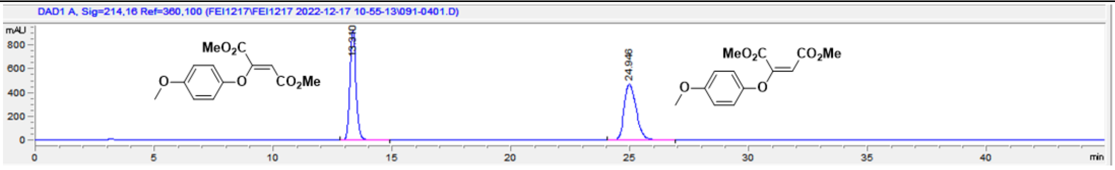

#	Time	Area	Height	Width	Area%	Symmetry
1	13.31	17076.6	926.7	0.283	50.808	0.775
2	24.946	16533.6	471.3	0.5412	49.192	0.696

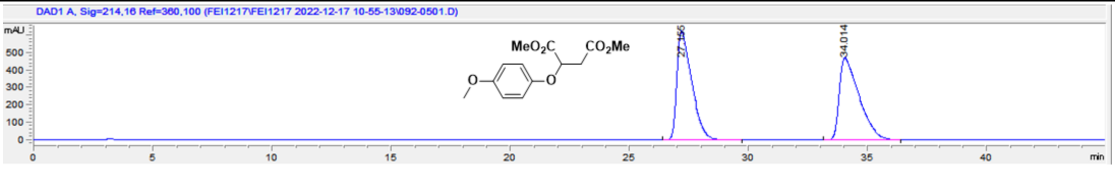

#	Time	Area	Height	Width	Area%	Symmetry
1	27.155	27236.1	628.8	0.6594	49.957	0.42
2	34.014	27282.9	473.5	0.8563	50.043	0.361

Obtained with ENE-101 from (*Z*)-**1bc**/(*E*)-**1bc** = 1/1

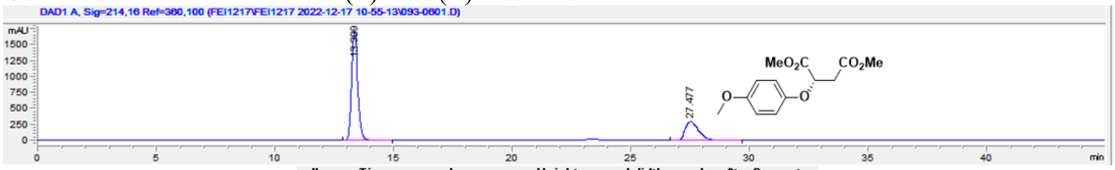

#	Time	Area	Height	Width	Area%	Symmetry
1	13.309	32190.7	1720.4	0.2923	73.361	0.718
2	27.477	11689	295	0.5963	26.639	0.595

HPLC analysis of **2bd**: Chiralpak® IG column (4.6 mm × 250 mm, 5 μm); detected at 214 nm; heptane/EtOH = 90/10; flow rate: 1.0 mL/min.

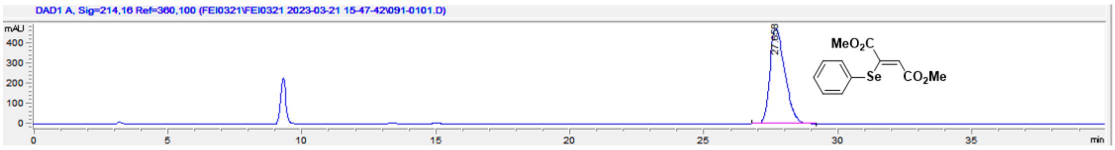

#	Time	Area	Height	Width	Area%	Symmetry
1	27.658	18249.1	472.3	0.5987	100.000	0.611

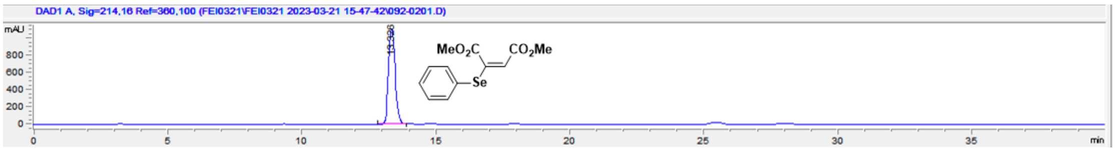

#	Time	Area	Height	Width	Area%	Symmetry
1	13.326	19080.3	1107.4	0.269	100.000	0.78

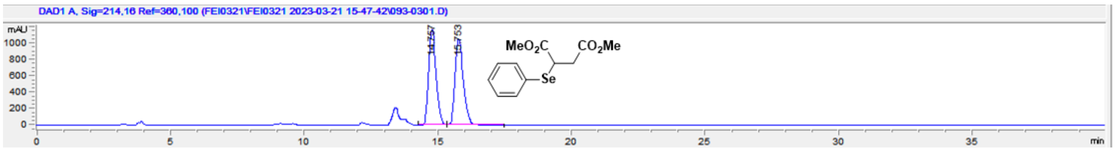

#	Time	Area	Height	Width	Area%	Symmetry
1	14.757	22508.6	1170.9	0.2983	49.931	0.72
2	15.753	22570.4	1052.3	0.3322	50.069	0.658

Obtained with ENE-101 from (*Z*)-**1bd**

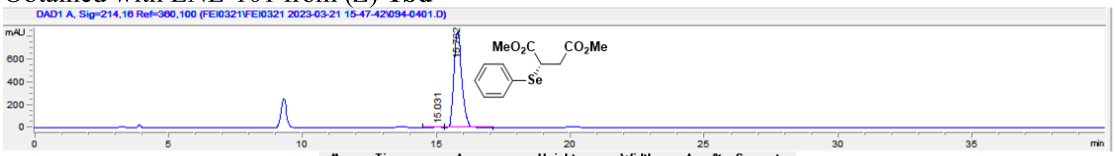

#	Time	Area	Height	Width	Area%	Symmetry
1	15.031	99.7	4.8	0.3093	0.580	1.328
2	15.762	17103.3	840.4	0.3154	99.420	0.712

Obtained with ENE-101 from (*E*)-**1bd**

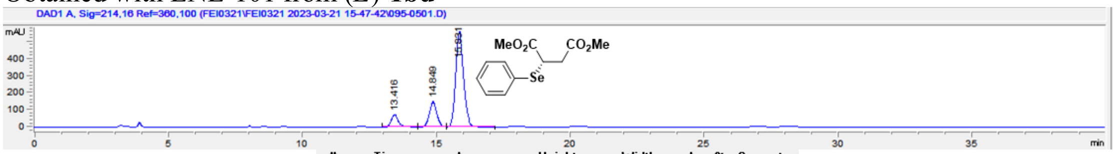

#	Time	Area	Height	Width	Area%	Symmetry
1	13.416	1375.4	74.3	0.2819	8.791	0.792
2	14.849	2862.4	149.6	0.2972	18.294	0.909
3	15.831	11408.7	557.9	0.3166	72.915	0.768

Obtained with ENE-101 from (*Z*)-**1bd**/(*E*)-**1bd** = 5.9/1

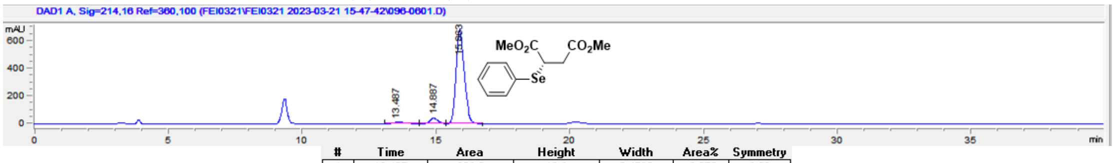

#	Time	Area	Height	Width	Area%	Symmetry
1	13.487	360.6	10.2	0.4723	2.273	0.39
2	14.887	848.1	41.8	0.3104	5.346	0.868
3	15.863	14655.3	685.7	0.3333	92.381	0.738

HPLC Analysis for Two-step One-pot Chemoenzymatic Cascade for the Enantioselective Synthesis of (*S*)-2

HPLC analysis of **2aa**: Chiralpak® IG column (4.6 mm × 250 mm, 5 μm); detected at 254 nm; heptane/EtOH = 90/10; flow rate: 1.0 mL/min.

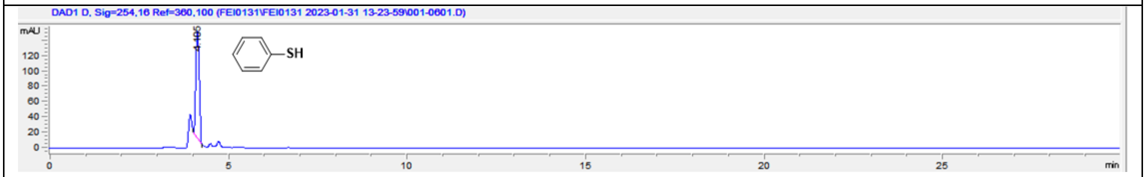

#	Time	Area	Height	Width	Area%	Symmetry
1	4.105	899.8	138	0.106	100.000	0.777

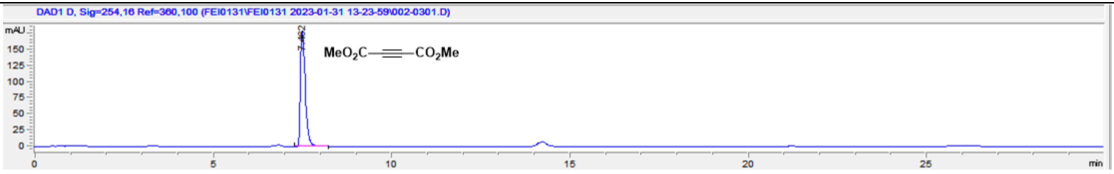

#	Time	Area	Height	Width	Area%	Symmetry
1	7.462	1758.5	179.2	0.1485	100.000	0.418

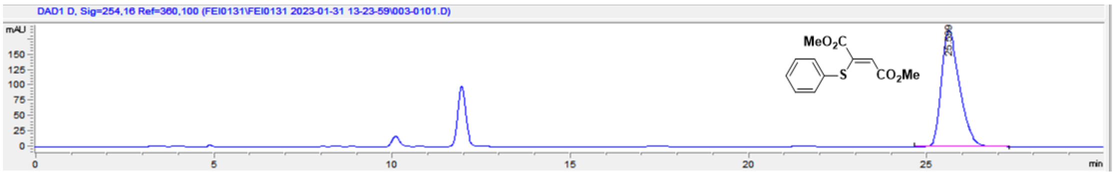

#	Time	Area	Height	Width	Area%	Symmetry
1	25.599	6691.3	189.4	0.5341	100.000	0.627

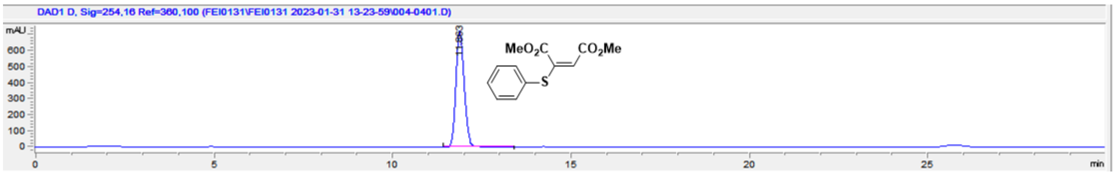

#	Time	Area	Height	Width	Area%	Symmetry
1	11.863	11237.1	723.7	0.2391	100.000	0.771

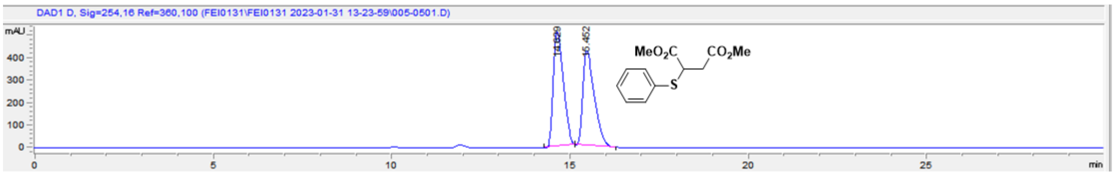

#	Time	Area	Height	Width	Area%	Symmetry
1	14.629	9796.7	511.1	0.2956	50.513	0.582
2	15.452	9597.6	423.1	0.3446	49.487	0.474

Obtained with ENE-101 *via* two-step one-pot chemoenzymatic cascade

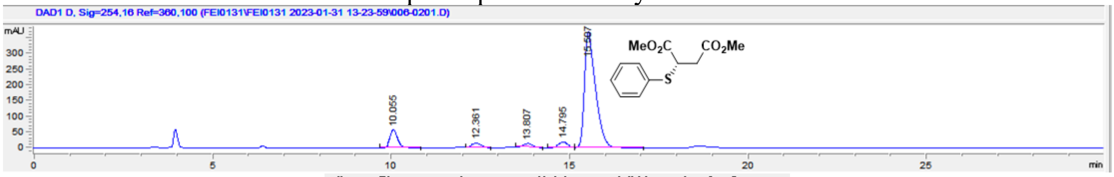

#	Time	Area	Height	Width	Area%	Symmetry
1	10.055	836.9	56.9	0.2255	8.661	0.851
2	12.361	227.3	13.8	0.2597	2.352	0.844
3	13.807	221.6	12.4	0.2872	2.293	0.859
4	14.795	320.4	18.4	0.2735	3.316	0.983
5	15.507	8056.6	369.4	0.3285	83.378	0.501

HPLC analysis of **2ab**: Chiralpak® IG column (4.6 mm × 250 mm, 5 μm); detected at 254 nm; heptane/EtOH = 96/4; flow rate: 1.0 mL/min.

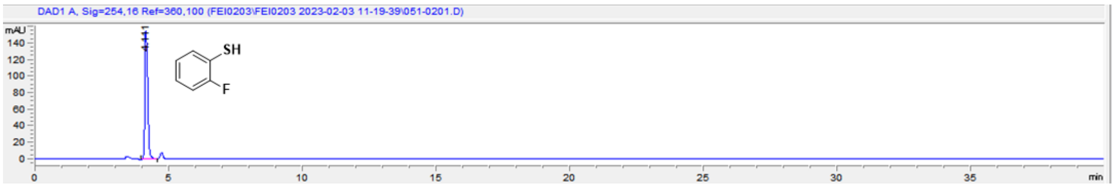

#	Time	Area	Height	Width	Area%	Symmetry
1	4.111	1231.2	154.5	0.129	100.000	0.682

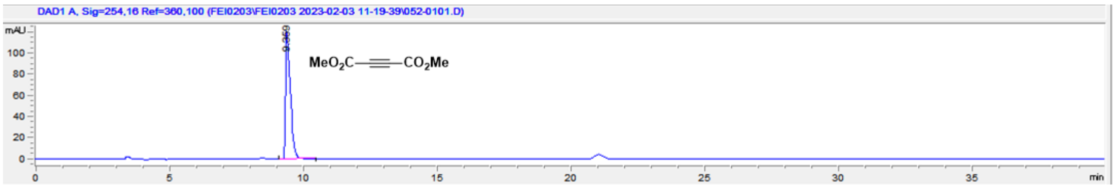

#	Time	Area	Height	Width	Area%	Symmetry
1	9.359	1598.4	120.8	0.1984	100.000	0.35

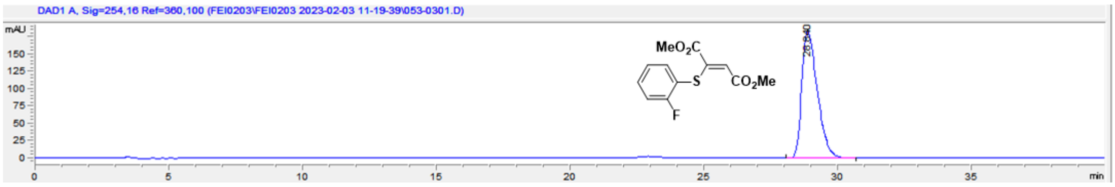

#	Time	Area	Height	Width	Area%	Symmetry
1	28.84	7378.2	183.8	0.6224	100.000	0.564

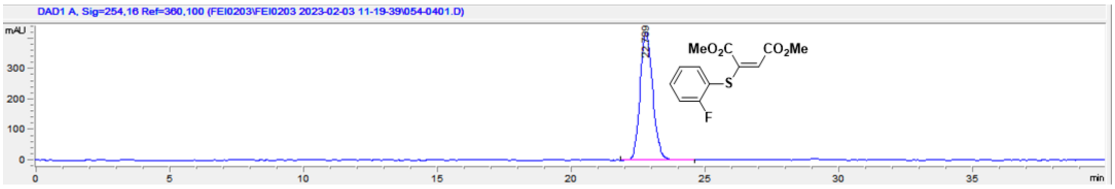

#	Time	Area	Height	Width	Area%	Symmetry
1	22.789	13826.8	422.4	0.5119	100.000	0.837

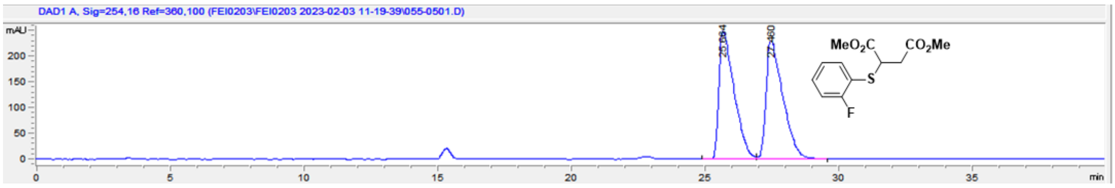

#	Time	Area	Height	Width	Area%	Symmetry
1	25.664	9881.6	248.5	0.5919	49.491	0.354
2	27.46	10084.7	230	0.6374	50.509	0.401

Obtained with ENE-101 *via* two-step one-pot chemoenzymatic cascade

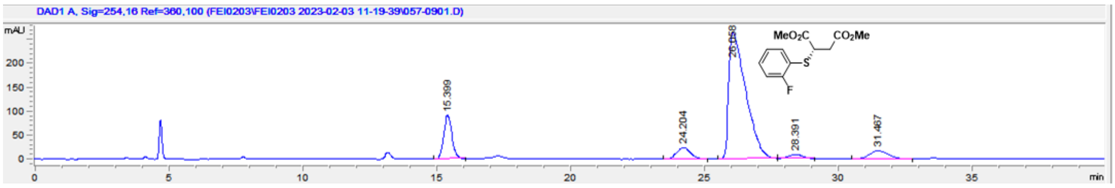

#	Time	Area	Height	Width	Area%	Symmetry
1	15.399	1953.5	90.9	0.3346	12.515	0.866
2	24.204	856	23.5	0.5704	5.484	0.881
3	26.058	11645	266.5	0.6417	74.603	0.308
4	28.391	294.8	8.1	0.5176	1.889	0.988
5	31.467	860	17.2	0.7259	5.509	0.802

HPLC analysis of **2ac**: Chiralpak® IG column (4.6 mm × 250 mm, 5 μm); detected at 254 nm; heptane/EtOH = 90/10; flow rate: 1.0 mL/min.

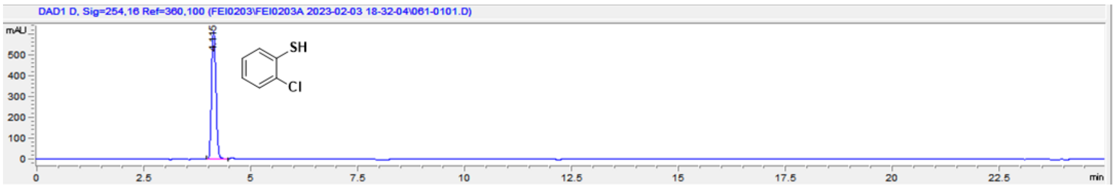

#	Time	Area	Height	Width	Area%	Symmetry
1	4.115	4428.8	613.8	0.1162	100.000	0.774

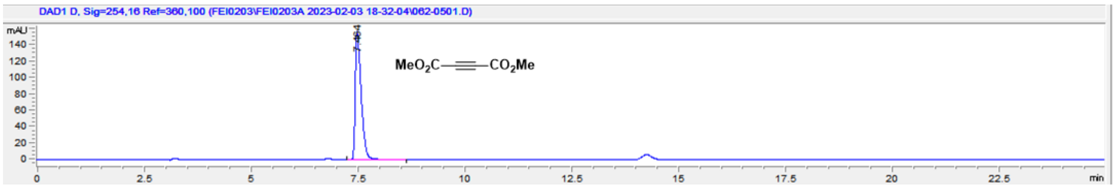

#	Time	Area	Height	Width	Area%	Symmetry
1	7.464	1514.1	156.4	0.1449	100.000	0.432

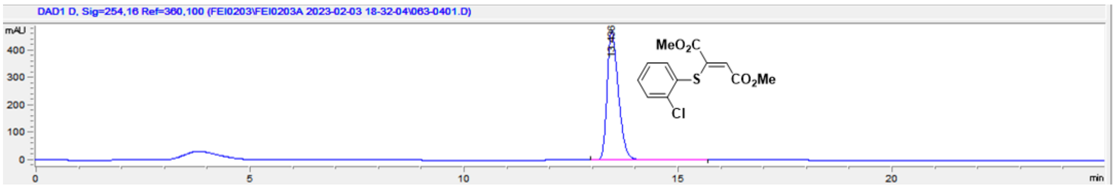

#	Time	Area	Height	Width	Area%	Symmetry
1	13.436	8617.1	468.3	0.2827	100.000	0.633

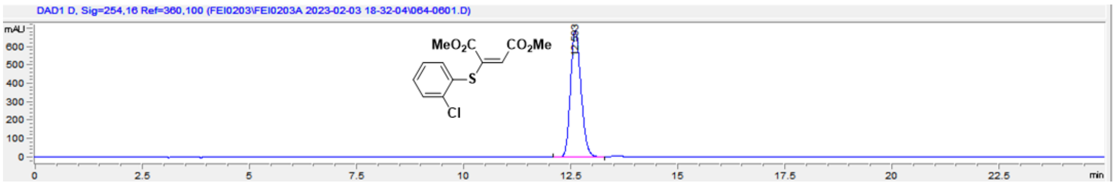

#	Time	Area	Height	Width	Area%	Symmetry
1	12.593	11825.2	688.4	0.2684	100.000	0.768

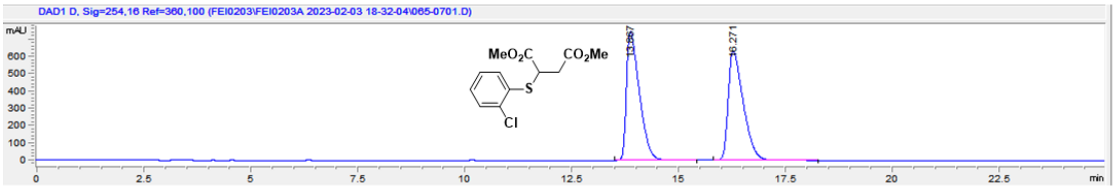

#	Time	Area	Height	Width	Area%	Symmetry
1	13.867	15619.4	745.3	0.3185	50.201	0.42
2	16.271	15494.3	634.4	0.3689	49.799	0.478

Obtained with ENE-101 *via* two-step one-pot chemoenzymatic cascade

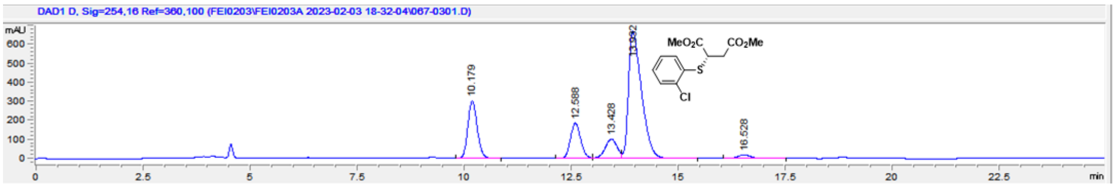

#	Time	Area	Height	Width	Area%	Symmetry
1	10.179	4597.8	303.9	0.2345	18.932	0.824
2	12.588	3115.2	186.8	0.2606	12.827	0.868
3	13.428	2022.3	102.6	0.308	8.327	1.063
4	13.932	14142.8	669.1	0.3226	58.235	0.466
5	16.528	407.8	18.3	0.3462	1.679	0.873

HPLC analysis of **2ad**: Chiralpak® IG column (4.6 mm × 250 mm, 5 μm); detected at 230 nm; heptane/EtOH = 90/10; flow rate: 1.0 mL/min.

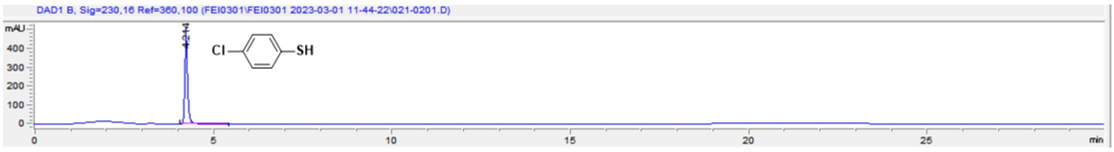

#	Time	Area	Height	Width	Area%	Symmetry
1	4.214	3173.4	508.8	0.0968	100.000	0.74

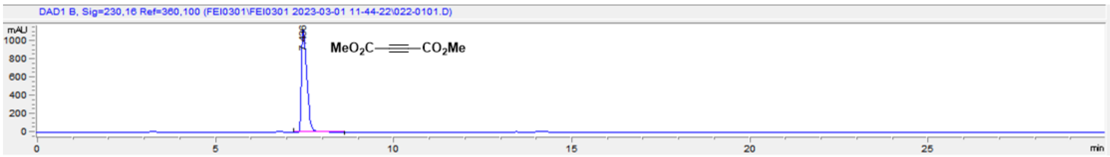

#	Time	Area	Height	Width	Area%	Symmetry
1	7.436	11705.2	1120.1	0.1599	100.000	0.419

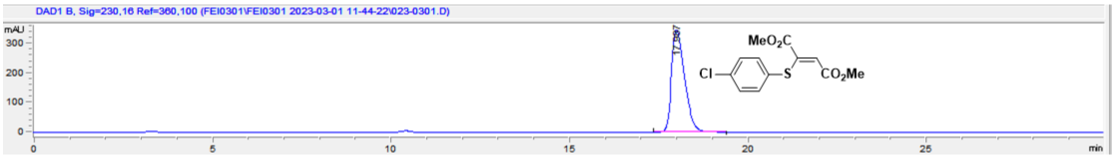

#	Time	Area	Height	Width	Area%	Symmetry
1	17.987	8753.4	347.4	0.382	100.000	0.615

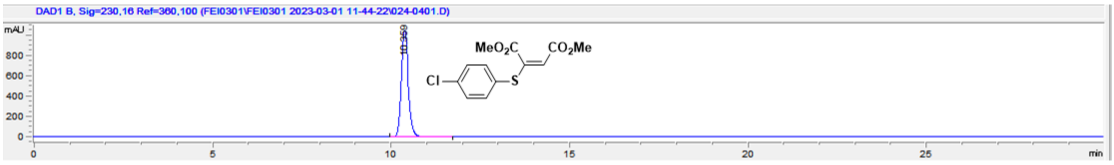

#	Time	Area	Height	Width	Area%	Symmetry
1	10.359	14642.9	1053.1	0.2142	100.000	0.776

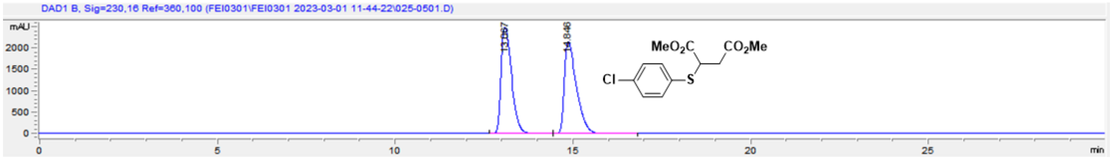

#	Time	Area	Height	Width	Area%	Symmetry
1	13.067	50980.3	2503.2	0.3156	49.827	0.496
2	14.846	51334.6	2151.5	0.3604	50.173	0.405

Obtained with ENE-101 *via* two-step one-pot chemoenzymatic cascade

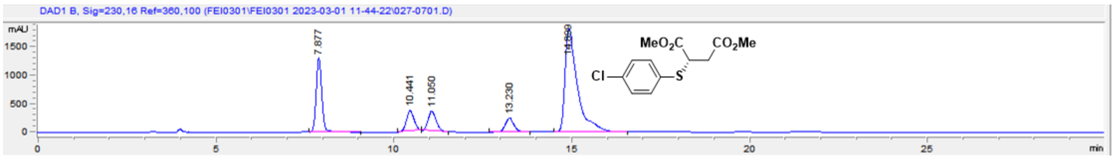

#	Time	Area	Height	Width	Area%	Symmetry
1	7.877	13727	1307.4	0.1625	18.443	0.82
2	10.441	5766.6	370.3	0.2437	7.748	0.901
3	11.05	5800.5	352.1	0.2582	7.793	0.836
4	13.23	4343.1	247.8	0.2685	5.835	0.857
5	14.899	44790.7	1809.7	0.3748	60.180	0.398

HPLC analysis of **2ae**: Chiralpak® IG column (4.6 mm × 250 mm, 5 μm); detected at 254 nm; heptane/EtOH = 90/10; flow rate: 1.0 mL/min.

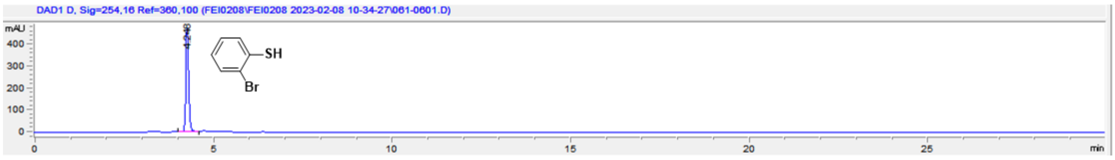

#	Time	Area	Height	Width	Area%	Symmetry
1	4.248	2955.3	471.7	0.0991	100.000	0.905

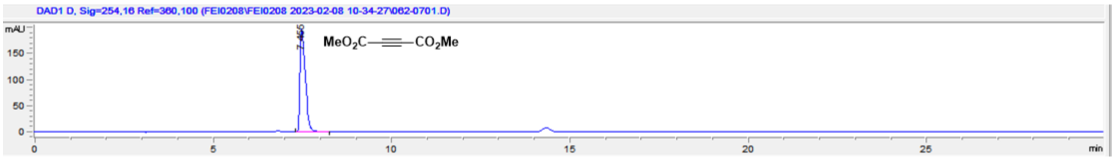

#	Time	Area	Height	Width	Area%	Symmetry
1	7.455	2052	197.1	0.1555	100.000	0.385

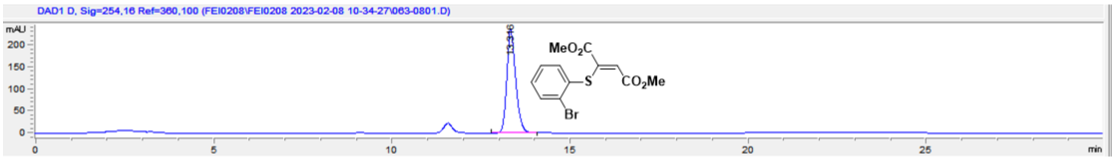

#	Time	Area	Height	Width	Area%	Symmetry
1	13.316	4211.9	235.3	0.2768	100.000	0.746

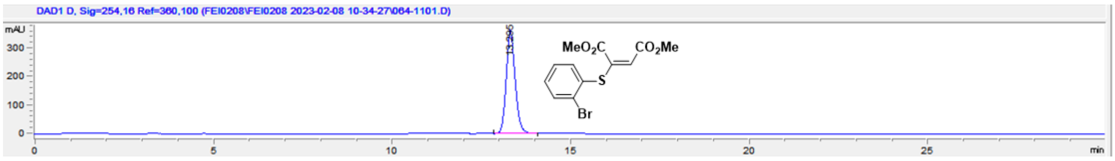

#	Time	Area	Height	Width	Area%	Symmetry
1	13.295	6513	365.3	0.278	100.000	0.825

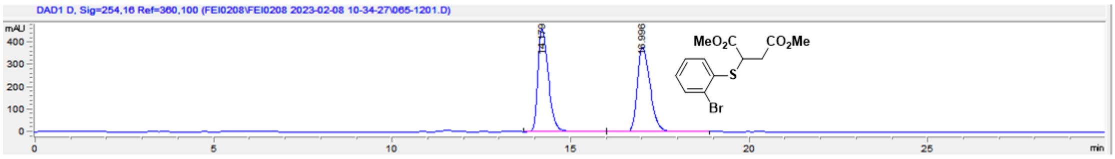

#	Time	Area	Height	Width	Area%	Symmetry
1	14.179	9289.7	461.9	0.3066	50.160	0.558
2	16.996	9230.3	378.8	0.3763	49.840	0.614

Obtained with ENE-101 *via* two-step one-pot chemoenzymatic cascade

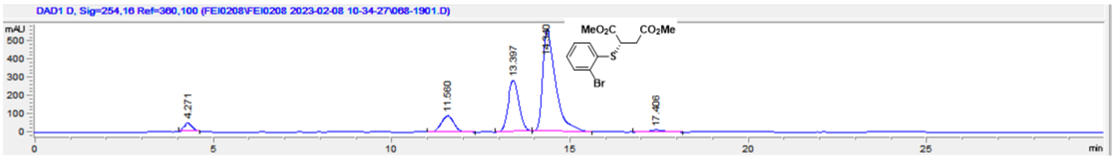

#	Time	Area	Height	Width	Area%	Symmetry
1	4.271	750.6	48.3	0.2213	3.197	0.956
2	11.56	2217.8	89.8	0.3942	9.447	1.03
3	13.397	5979.7	281.8	0.3336	25.471	0.911
4	14.34	14225.6	560.9	0.3859	60.594	0.474
5	17.406	303.4	10.9	0.4334	1.292	0.938

HPLC analysis of **2ag**: Chiralpak® ID column (4.6 mm × 250 mm, 5 μm); detected at 230 nm; heptane/EtOH = 90/10; flow rate: 1.0 mL/min.

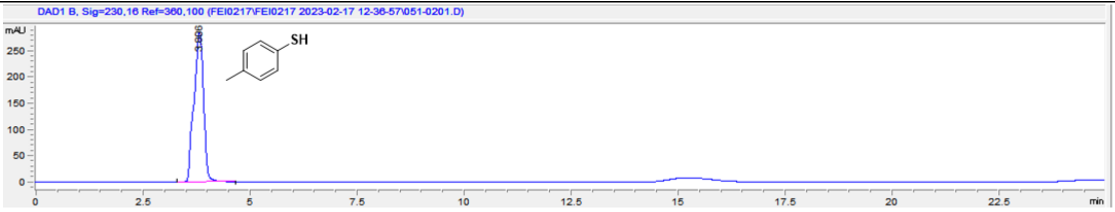

#	Time	Area	Height	Width	Area%	Symmetry
1	3.806	4494.4	285.3	0.2298	100.000	1.284

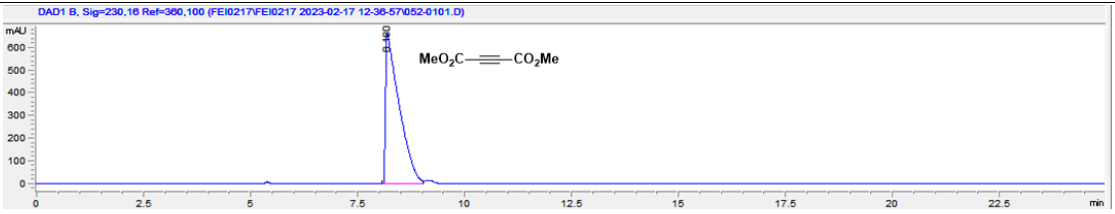

#	Time	Area	Height	Width	Area%	Symmetry
1	8.18	14713.7	665.2	0.2961	100.000	0.128

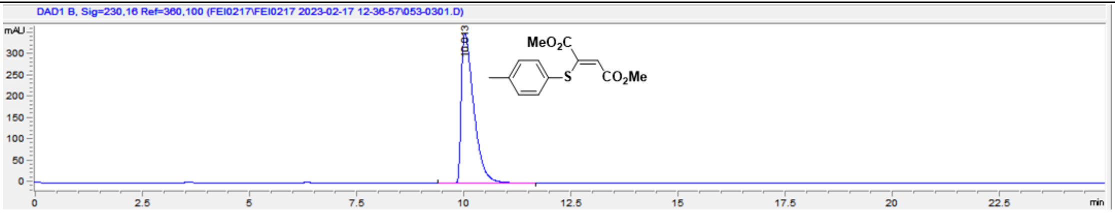

#	Time	Area	Height	Width	Area%	Symmetry
1	10.013	7161.3	353.5	0.3043	100.000	0.373

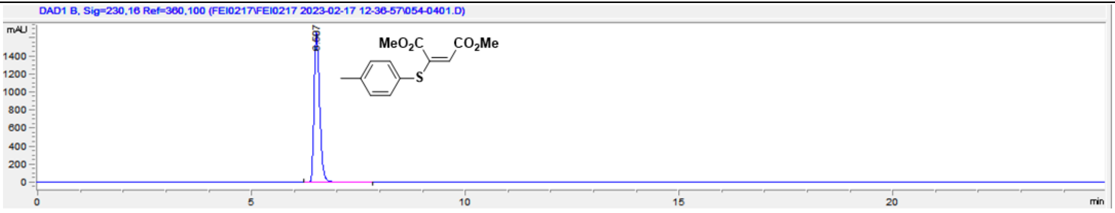

#	Time	Area	Height	Width	Area%	Symmetry
1	6.507	15165.9	1669	0.14	100.000	0.675

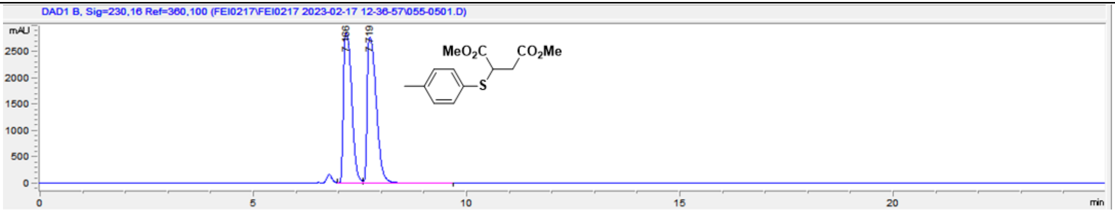

#	Time	Area	Height	Width	Area%	Symmetry
1	7.166	38941	2857.3	0.219	48.420	0.489
2	7.719	41482.6	2773.1	0.2365	51.580	0.382

Obtained with ENE-101 *via* two-step one-pot chemoenzymatic cascade

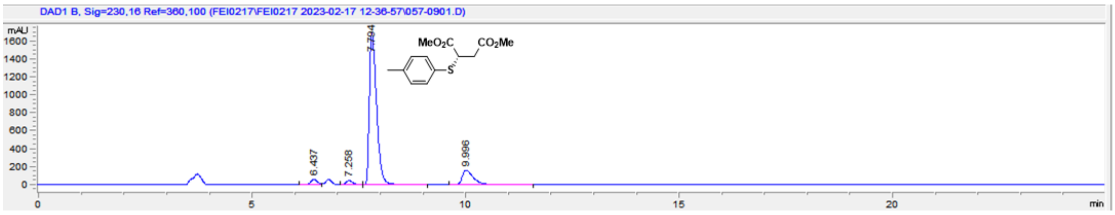

#	Time	Area	Height	Width	Area%	Symmetry
1	6.437	680.8	62.3	0.1675	2.852	0.928
2	7.258	497.9	46.5	0.163	2.086	0.825
3	7.794	19755.7	1694.2	0.18	82.755	0.529
4	9.996	2938	161.9	0.2777	12.307	0.464

HPLC analysis of **2ah**: Chiralpak® IG column (4.6 mm × 250 mm, 5 μm); detected at 230 nm; heptane/EtOH = 90/10; flow rate: 1.0 mL/min.

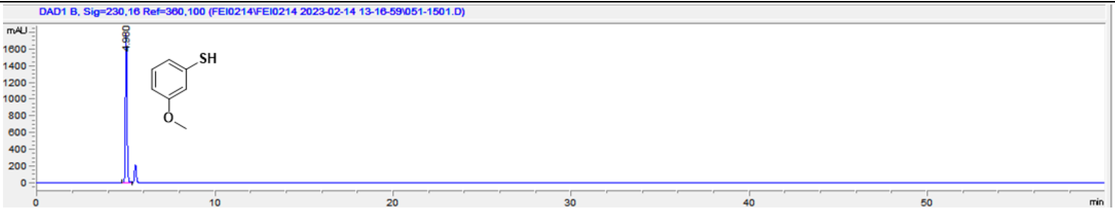

#	Time	Area	Height	Width	Area%	Symmetry
1	4.98	10540.7	1799.1	0.0904	100.000	0.786

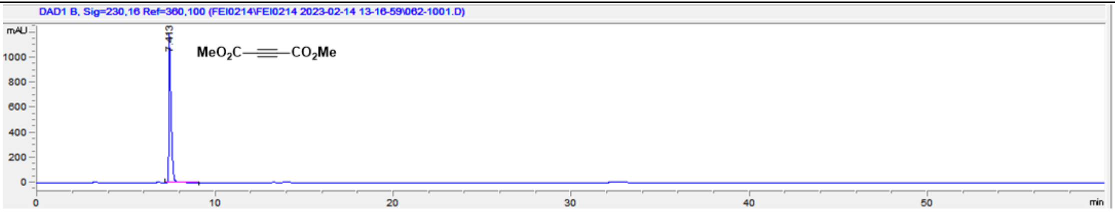

#	Time	Area	Height	Width	Area%	Symmetry
1	7.413	12371.2	1197.5	0.1585	100.000	0.408

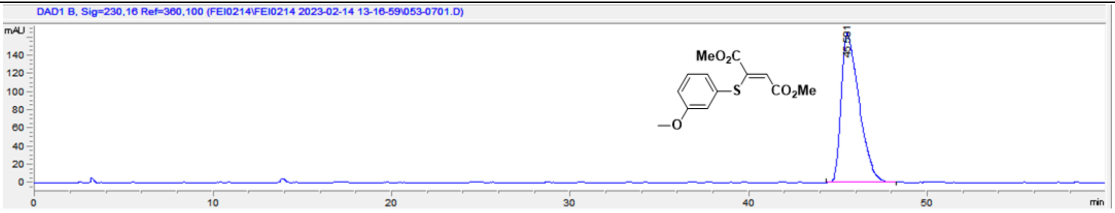

#	Time	Area	Height	Width	Area%	Symmetry
1	45.501	11441.9	164.7	1.0463	100.000	0.484

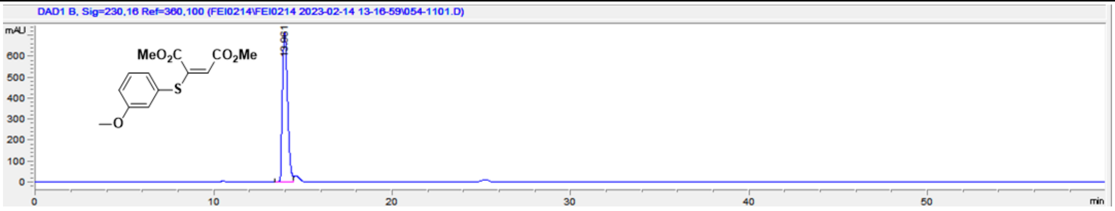

#	Time	Area	Height	Width	Area%	Symmetry
1	13.961	13748.5	715.4	0.2982	100.000	0.751

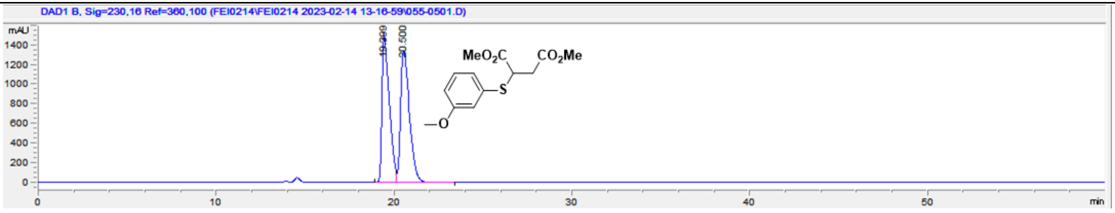

#	Time	Area	Height	Width	Area%	Symmetry
1	19.399	44893	1526.8	0.447	49.456	0.398
2	20.5	45881.3	1341.7	0.509	50.544	0.446

Obtained with ENE-101 *via* two-step one-pot chemoenzymatic cascade

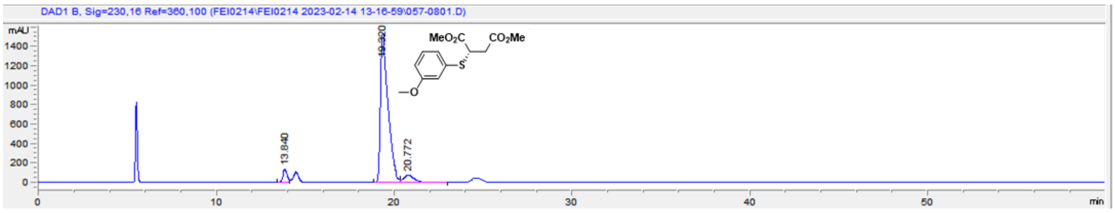

#	Time	Area	Height	Width	Area%	Symmetry
1	13.84	2526.9	137.1	0.285	4.787	0.881
2	19.32	47027.9	1543.6	0.4534	89.088	0.364
3	20.772	3233.1	77.4	0.6331	6.125	0.723

HPLC analysis of **2ai**: Chiralpak® IG column (4.6 mm × 250 mm, 5 μm); detected at 230 nm; heptane/EtOH = 90/10; flow rate: 1.0 mL/min.

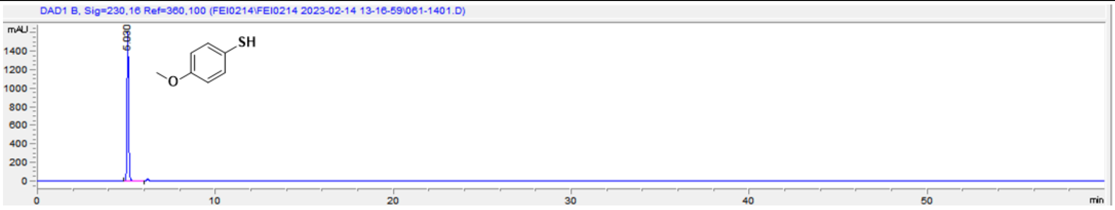

#	Time	Area	Height	Width	Area%	Symmetry
1	5.03	9437.8	1612.7	0.0903	100.000	0.788

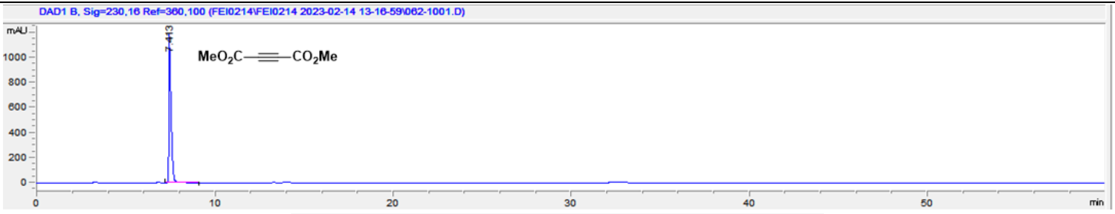

#	Time	Area	Height	Width	Area%	Symmetry
1	7.413	12371.2	1197.5	0.1585	100.000	0.408

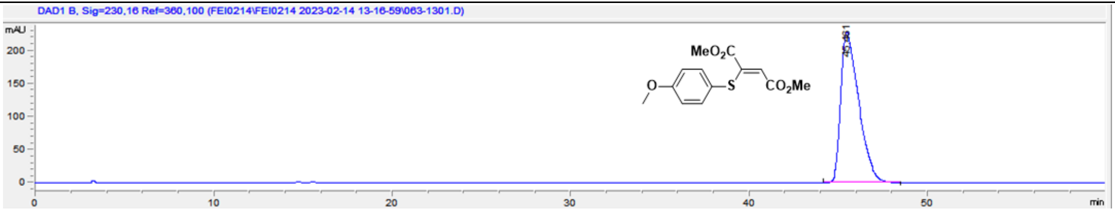

#	Time	Area	Height	Width	Area%	Symmetry
1	45.461	15911.2	227.7	1.0291	100.000	0.469

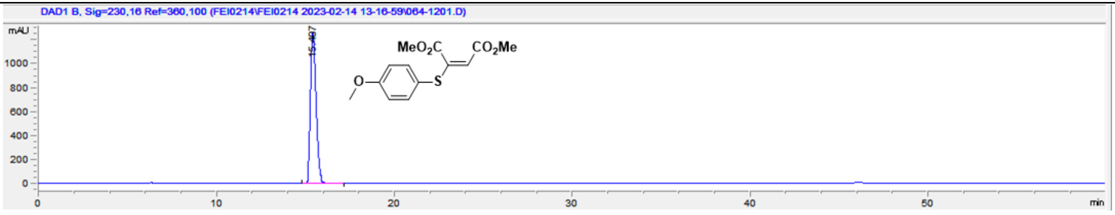

#	Time	Area	Height	Width	Area%	Symmetry
1	15.407	26479.5	1260.9	0.325	100.000	0.705

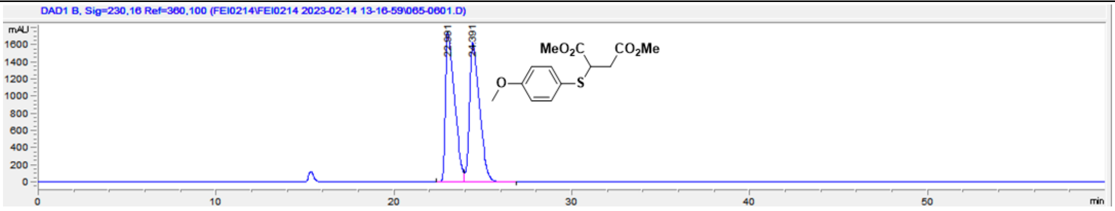

#	Time	Area	Height	Width	Area%	Symmetry
1	22.991	64193.3	1751.7	0.5655	49.563	0.37
2	24.391	65324.1	1627.3	0.6083	50.437	0.48

Obtained with ENE-101 *via* two-step one-pot chemoenzymatic cascade

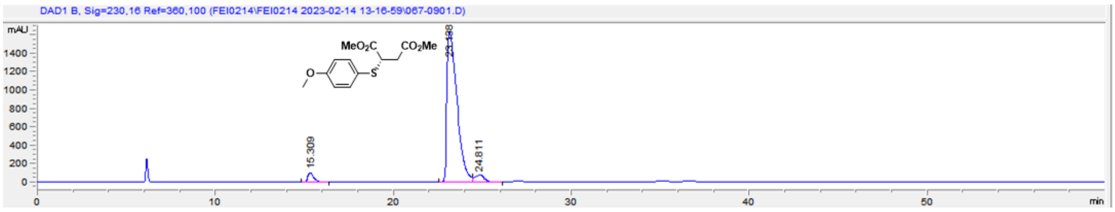

#	Time	Area	Height	Width	Area%	Symmetry
1	15.309	2321.4	102.5	0.3383	3.362	0.728
2	23.138	63744	1634.2	0.5791	92.321	0.353
3	24.811	2980.8	77.8	0.5789	4.317	0.889

HPLC analysis of **2aj**: Chiralcel® OJ-H column (4.6 mm × 250 mm, 5 µm); detected at 230 nm; heptane/EtOH = 90/10; flow rate: 1.0 mL/min.

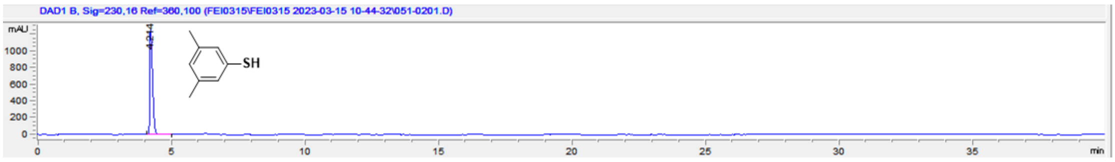

#	Time	Area	Height	Width	Area%	Symmetry
1	4.214	9333.9	1259.1	0.1185	100.000	0.556

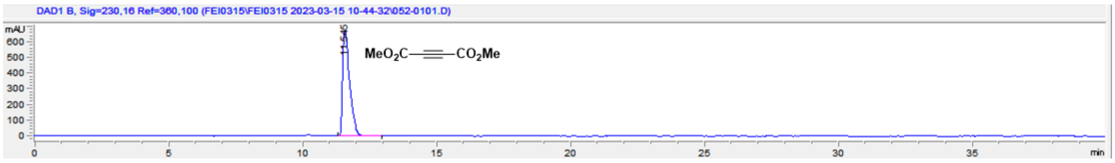

#	Time	Area	Height	Width	Area%	Symmetry
1	11.545	12247.2	681.1	0.2718	100.000	0.35

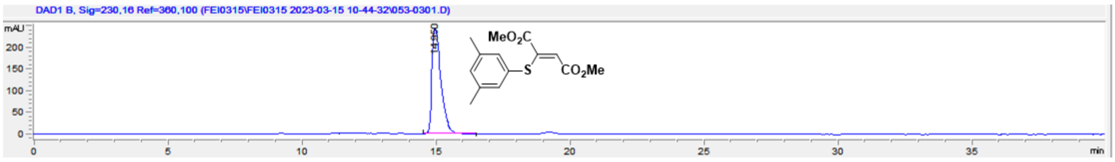

#	Time	Area	Height	Width	Area%	Symmetry
1	14.95	5690.9	242.1	0.3602	100.000	0.51

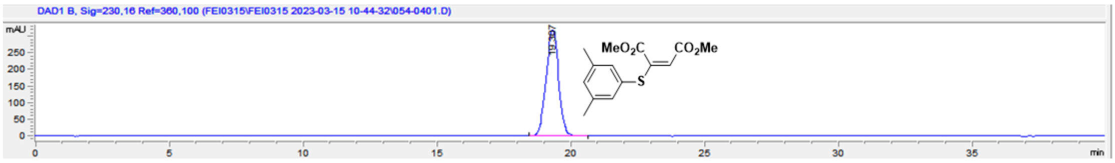

#	Time	Area	Height	Width	Area%	Symmetry
1	19.307	10378.2	320.9	0.5013	100.000	1.084

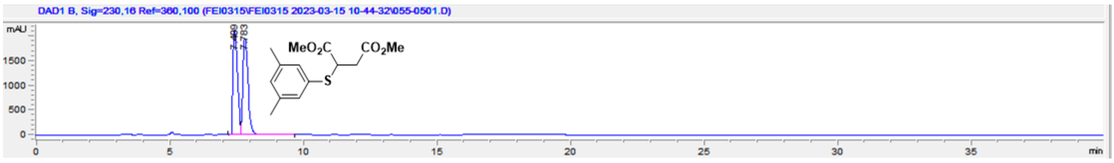

#	Time	Area	Height	Width	Area%	Symmetry
1	7.409	23486.9	2139.3	0.1721	48.271	0.553
2	7.783	25169	1961	0.1977	51.729	0.515

Obtained with ENE-101 *via* two-step one-pot chemoenzymatic cascade

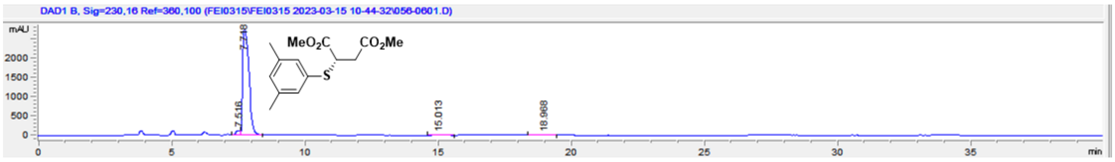

#	Time	Area	Height	Width	Area%	Symmetry
1	7.516	1360.3	123	0.147	3.082	2.77
2	7.718	42554.9	2741.3	0.2491	96.416	0.388
3	15.013	87	4.3	0.2816	0.197	0.832
4	18.968	134.7	5.1	0.4137	0.305	0.993

HPLC analysis of **2ak**: Chiralcel® OD-H column (4.6 mm × 250 mm, 5 μm); detected at 230 nm; Hexane/EtOH = 90/10; flow rate: 1.0 mL/min.

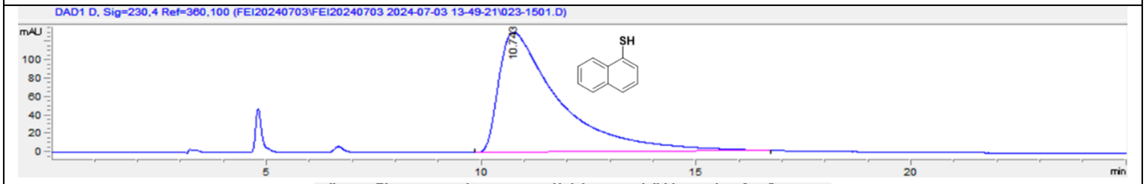

#	Time	Area	Height	Width	Area%	Symmetry
1	10.743	12759.4	130.8	1.2804	100.000	0.3

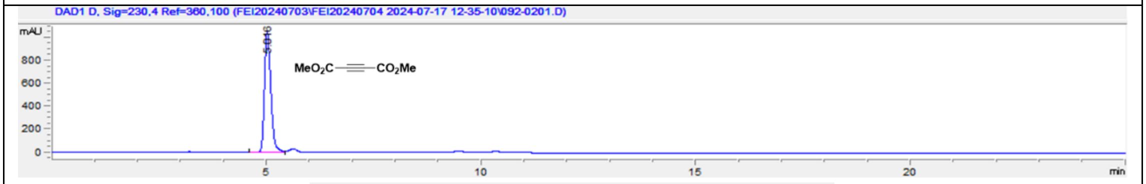

#	Time	Area	Height	Width	Area%	Symmetry
1	5.016	10734.6	1052	0.155	100.000	0.735

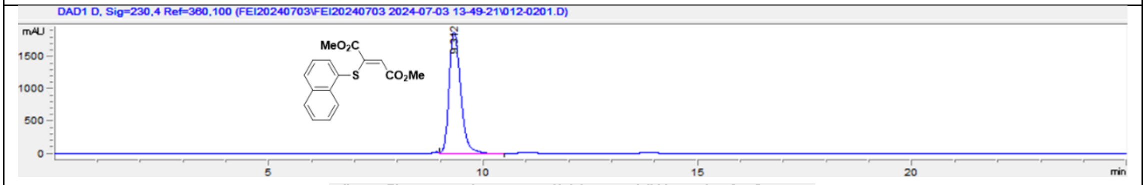

#	Time	Area	Height	Width	Area%	Symmetry
1	9.312	35589.8	1885.5	0.2922	100.000	0.703

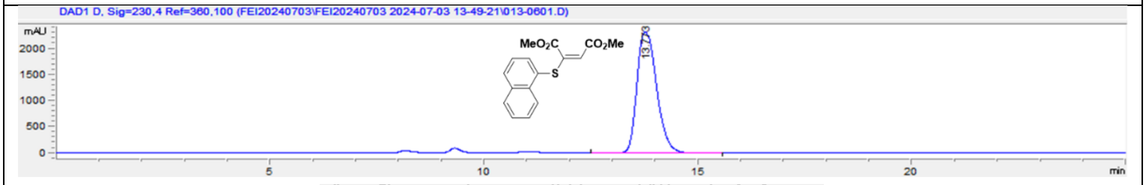

#	Time	Area	Height	Width	Area%	Symmetry
1	13.773	74218.9	2319.9	0.4452	100.000	0.734

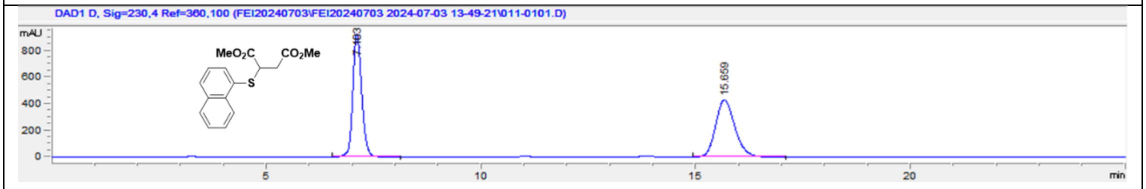

#	Time	Area	Height	Width	Area%	Symmetry
1	7.103	13259.4	926.7	0.2189	49.711	0.811
2	15.659	13413.8	431.6	0.4766	50.289	0.789

Obtained with ENE-101 *via* two-step one-pot chemoenzymatic cascade

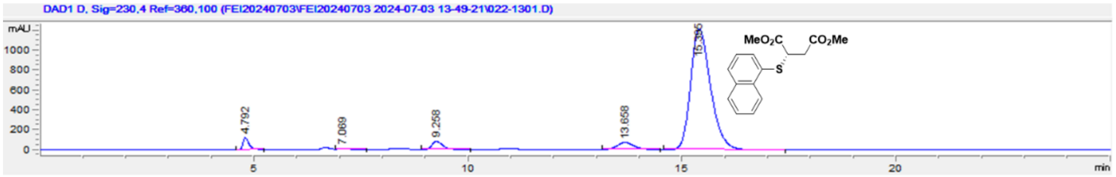

#	Time	Area	Height	Width	Area%	Symmetry
1	4.792	1188.8	119.4	0.1502	2.696	0.598
2	7.069	220.3	12.7	0.2497	0.500	0.609
3	9.258	1469.8	82.9	0.2689	3.333	0.708
4	13.658	1971.3	71.5	0.4219	4.470	0.832
5	15.395	39247.6	1213.4	0.4974	89.001	0.715

HPLC analysis of **2al**: Chiralpak® ID column (4.6 mm × 250 mm, 5 μm); detected at 230 nm; heptane/EtOH = 90/10; flow rate: 1.0 mL/min.

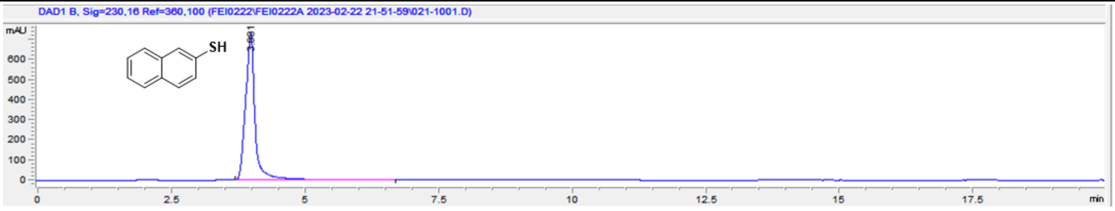

#	Time	Area	Height	Width	Area%	Symmetry
1	3.981	9155.7	735.9	0.1792	100.000	1.029

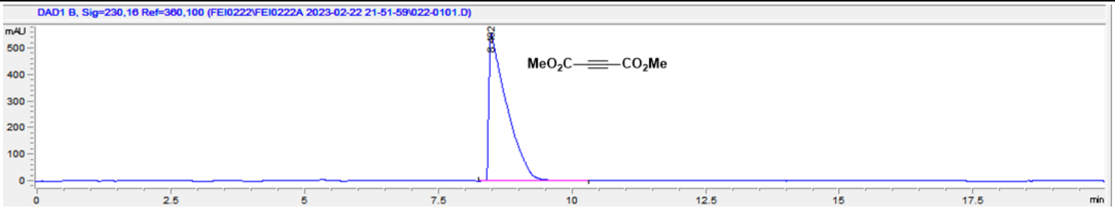

#	Time	Area	Height	Width	Area%	Symmetry
1	8.482	12884.4	556.3	0.3082	100.000	0.123

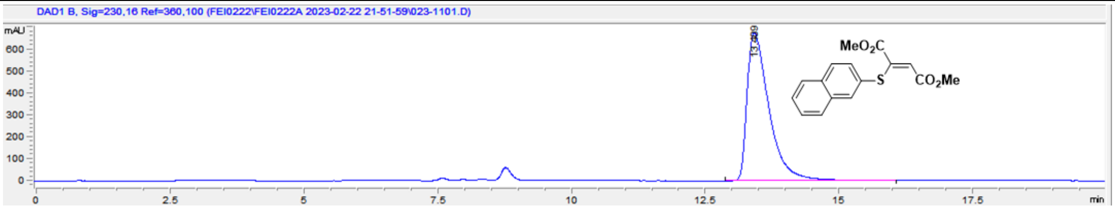

#	Time	Area	Height	Width	Area%	Symmetry
1	13.409	19231.8	675.5	0.4302	100.000	0.449

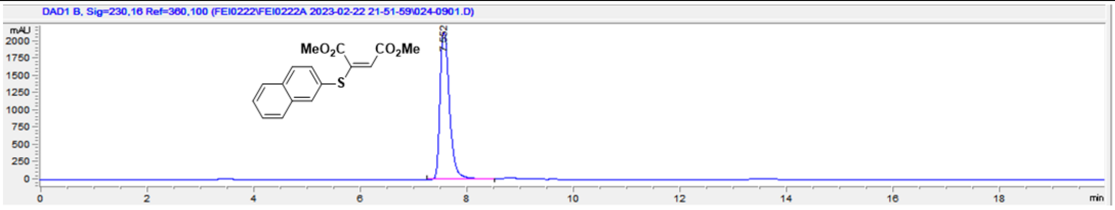

#	Time	Area	Height	Width	Area%	Symmetry
1	7.552	26482.5	2128.1	0.1932	100.000	0.641

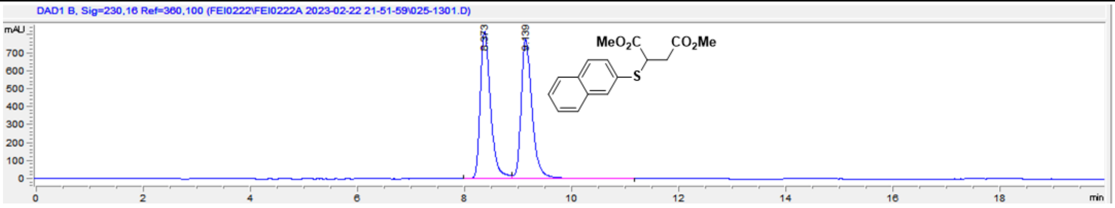

#	Time	Area	Height	Width	Area%	Symmetry
1	8.373	11037.7	819.1	0.2052	49.763	0.657
2	9.139	11142.9	776.6	0.2194	50.237	0.63

Obtained with ENE-101 *via* two-step one-pot chemoenzymatic cascade

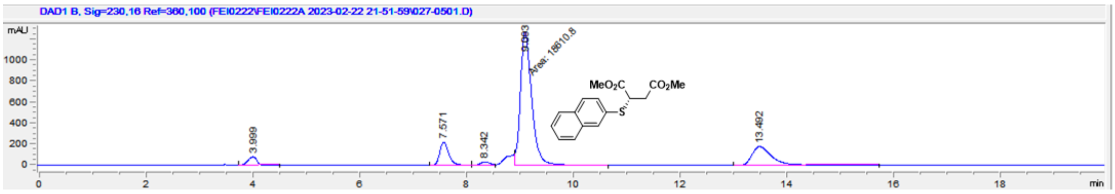

#	Time	Area	Height	Width	Area%	Symmetry
1	3.999	927.7	80.3	0.1887	3.405	1.323
2	7.571	2585.4	219.1	0.1797	9.491	0.711
3	8.342	440.9	33.6	0.201	1.618	0.878
4	9.093	18610.8	1273.4	0.2436	68.318	0.656
5	13.492	4676.9	178.7	0.3934	17.168	0.572

HPLC analysis of **2an**: Chiralpak® IG column (4.6 mm × 250 mm, 5 μm); detected at 230 nm; heptane/EtOH = 90/10; flow rate: 1.0 mL/min.

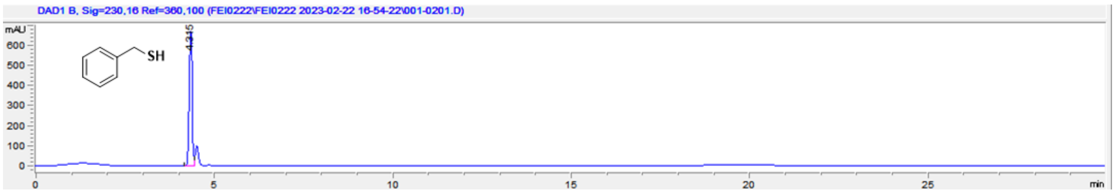

#	Time	Area	Height	Width	Area%	Symmetry
1	4.315	4082.2	675.9	0.0945	100.000	0.86

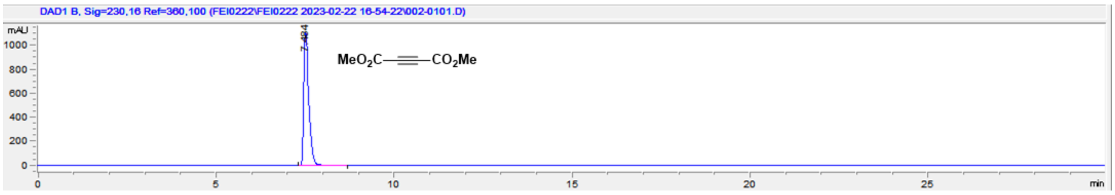

#	Time	Area	Height	Width	Area%	Symmetry
1	7.484	11458.6	1116.1	0.1578	100.000	0.428

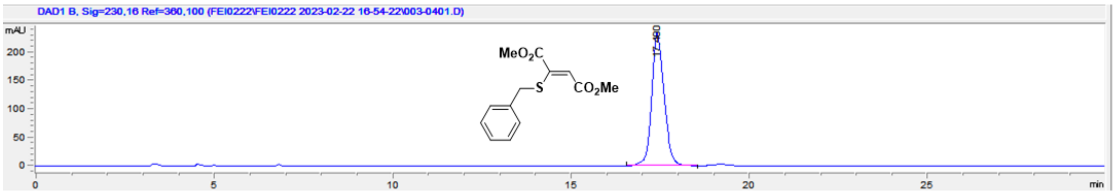

#	Time	Area	Height	Width	Area%	Symmetry
1	17.4	5697.3	235.6	0.3701	100.000	0.769

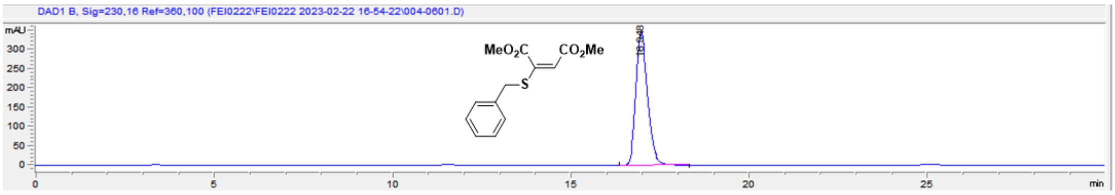

#	Time	Area	Height	Width	Area%	Symmetry
1	16.948	7952.6	346.1	0.3521	100.000	0.721

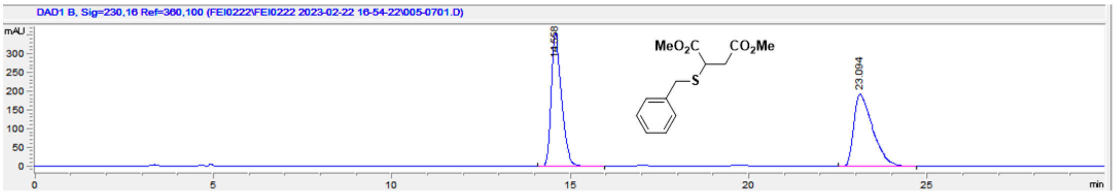

#	Time	Area	Height	Width	Area%	Symmetry
1	14.558	7166.1	357.9	0.3076	50.405	0.594
2	23.094	7051	194.1	0.5398	49.595	0.453

Obtained with ENE-101 *via* two-step one-pot chemoenzymatic cascade

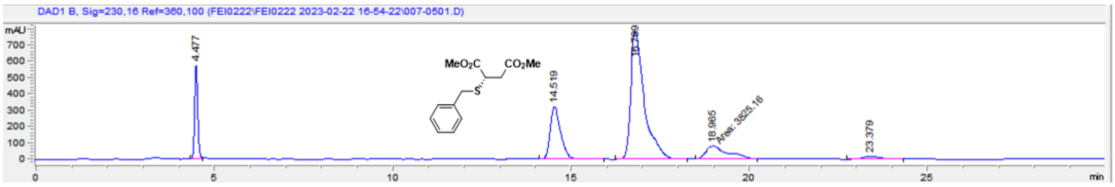

#	Time	Area	Height	Width	Area%	Symmetry
1	4.477	3195.7	574.9	0.0869	9.157	0.826
2	14.519	6524.1	322.9	0.3097	18.693	0.621
3	16.789	20823	785	0.3915	59.664	0.474
4	18.965	3825.2	83.3	0.7655	10.960	0.457
5	23.379	532.6	16.6	0.4932	1.526	0.82

HPLC analysis of **2bd**: Chiralpak® IG column (4.6 mm × 250 mm, 5 μm); detected at 214 nm; heptane/EtOH = 90/10; flow rate: 1.0 mL/min.

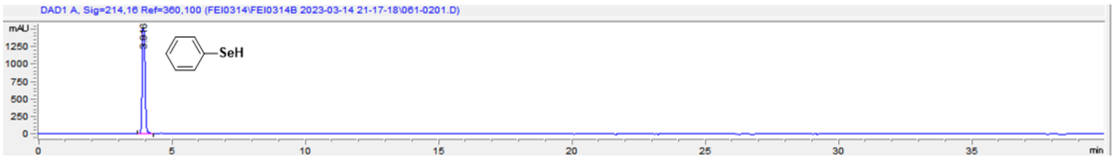

#	Time	Area	Height	Width	Area%	Symmetry
1	3.916	12833.1	1516.3	0.1367	100.000	1

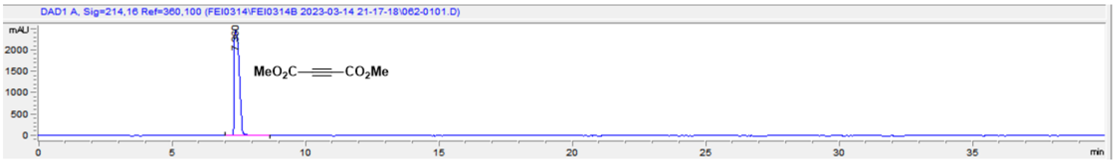

#	Time	Area	Height	Width	Area%	Symmetry
1	7.36	32180.7	2473	0.2118	100.000	0.362

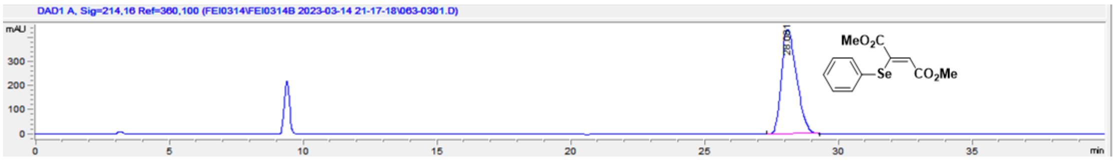

#	Time	Area	Height	Width	Area%	Symmetry
1	28.081	17178.3	437	0.6186	100.000	0.634

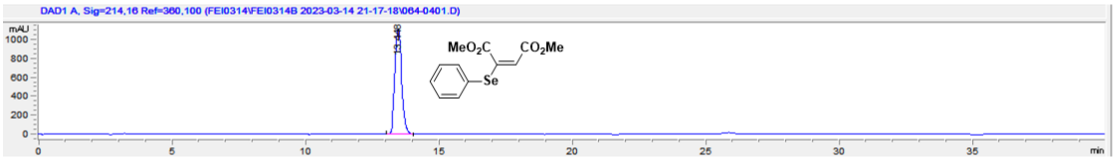

#	Time	Area	Height	Width	Area%	Symmetry
1	13.448	19625.6	1112	0.2759	100.000	0.791

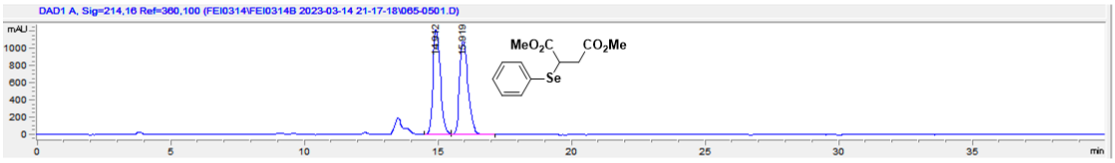

#	Time	Area	Height	Width	Area%	Symmetry
1	14.912	23696	1224.7	0.2997	49.817	0.71
2	15.919	23870.6	1089.2	0.3377	50.183	0.632

Obtained with ENE-101 *via* two-step one-pot chemoenzymatic cascade

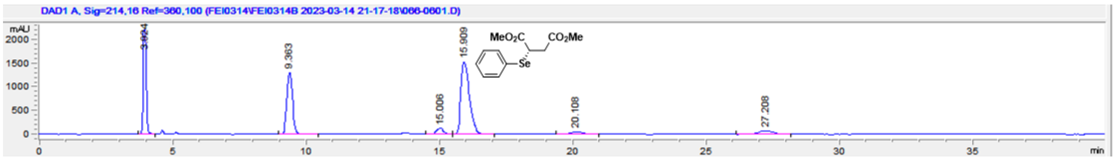

#	Time	Area	Height	Width	Area%	Symmetry
1	3.924	19444.1	2222.3	0.1421	24.517	0.819
2	9.363	18986.1	1308	0.2253	23.940	0.859
3	15.006	2393.7	124.7	0.298	3.018	0.956
4	15.909	33776.4	1529.8	0.3416	42.589	0.558
5	20.108	1714.5	56.2	0.4496	2.162	0.807
6	27.208	2992.3	74.8	0.6244	3.773	0.931

HPLC Analysis for One-step One-pot Chemoenzymatic Cascade for the Enantioselective Synthesis of (*S*)-2

HPLC analysis of **2aa**: Chiralpak® IG column (4.6 mm × 250 mm, 5 μm); detected at 254 nm; heptane/EtOH = 90/10; flow rate: 1.0 mL/min.

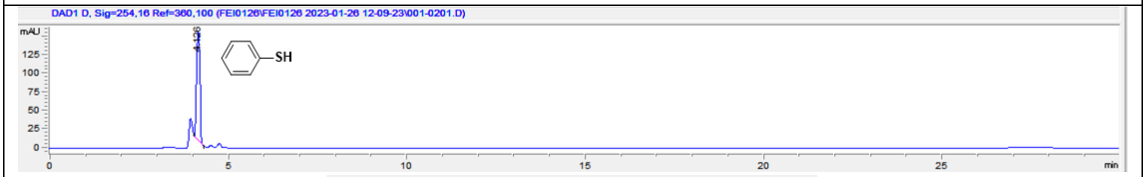

#	Time	Area	Height	Width	Area%	Symmetry
1	4.126	967.4	144.4	0.1102	100.000	0.766

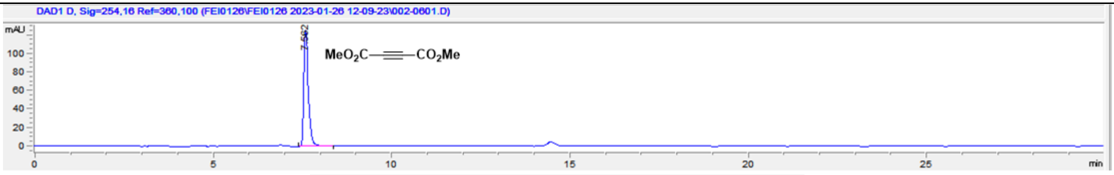

#	Time	Area	Height	Width	Area%	Symmetry
1	7.562	1188.9	125.2	0.1448	100.000	0.485

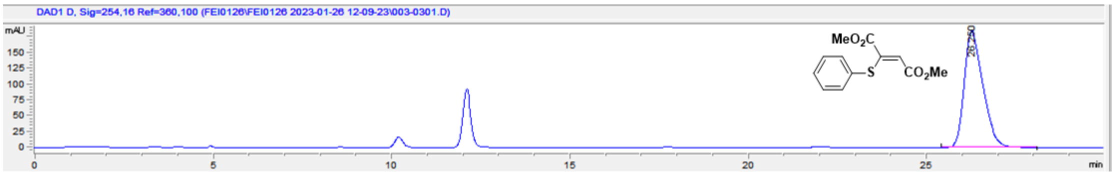

#	Time	Area	Height	Width	Area%	Symmetry
1	26.25	6700.4	183.9	0.557	100.000	0.626

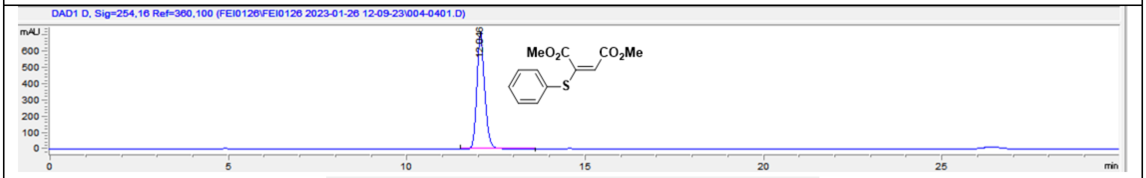

#	Time	Area	Height	Width	Area%	Symmetry
1	12.046	11436.9	716.3	0.2463	100.000	0.763

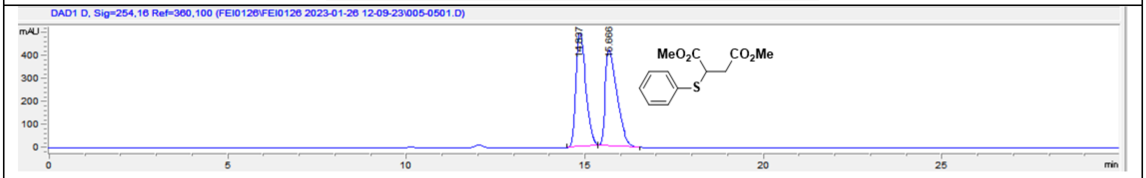

#	Time	Area	Height	Width	Area%	Symmetry
1	14.837	9587.3	498.2	0.2985	50.230	0.585
2	15.666	9499.6	415.9	0.3464	49.770	0.474

Obtained with ENE-101 *via* one-step one-pot chemoenzymatic cascade

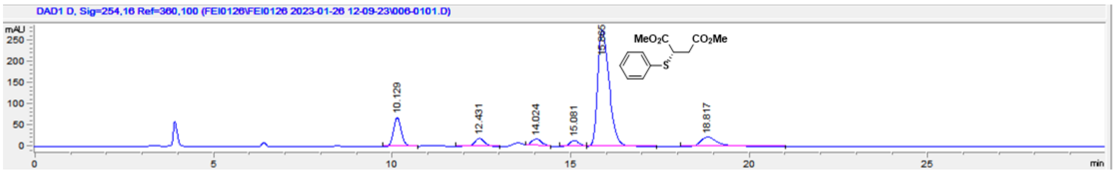

#	Time	Area	Height	Width	Area%	Symmetry
1	10.129	977.3	67.7	0.2243	11.413	0.883
2	12.431	365.6	19	0.2947	4.269	1.033
3	14.024	263	14.6	0.286	3.071	0.833
4	15.081	240.8	13.2	0.2869	2.812	0.942
5	15.865	5993.1	273.1	0.336	69.986	0.573
6	18.817	723.5	21.8	0.4952	8.448	0.613

HPLC analysis of **2ab**: Chiralpak® IG column (4.6 mm × 250 mm, 5 μm); detected at 254 nm; heptane/EtOH = 96/4; flow rate: 1.0 mL/min.

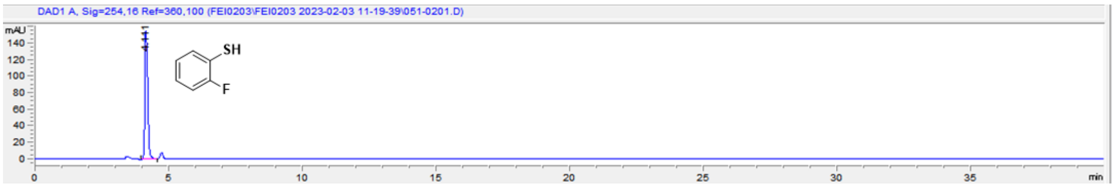

#	Time	Area	Height	Width	Area%	Symmetry
1	4.111	1231.2	154.5	0.129	100.000	0.682

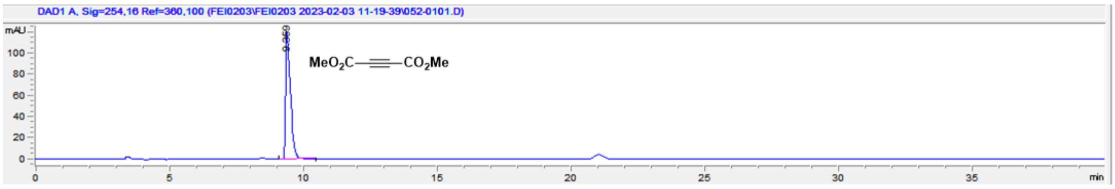

#	Time	Area	Height	Width	Area%	Symmetry
1	9.359	1598.4	120.8	0.1984	100.000	0.35

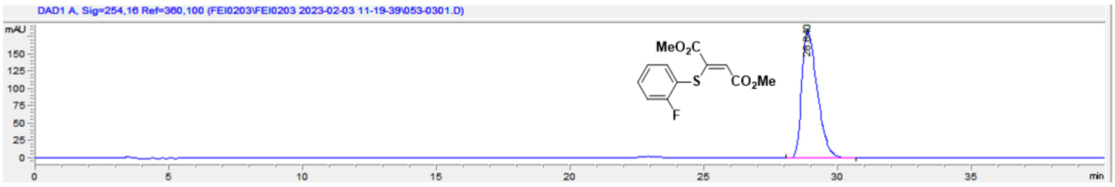

#	Time	Area	Height	Width	Area%	Symmetry
1	28.84	7378.2	183.8	0.6224	100.000	0.564

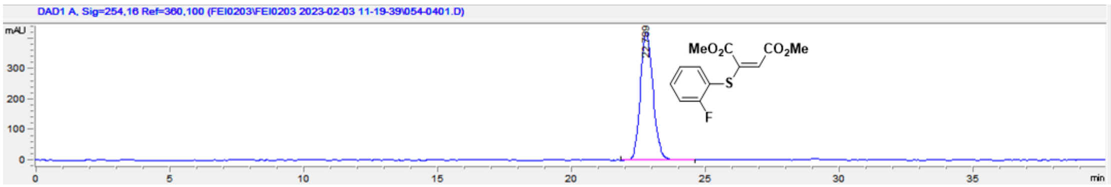

#	Time	Area	Height	Width	Area%	Symmetry
1	22.789	13826.8	422.4	0.5119	100.000	0.837

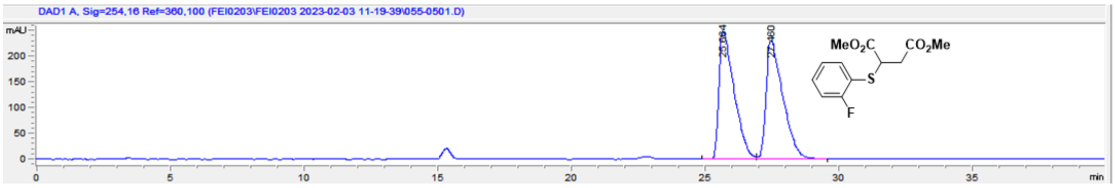

#	Time	Area	Height	Width	Area%	Symmetry
1	25.664	9881.6	248.5	0.5919	49.491	0.354
2	27.46	10084.7	230	0.6374	50.509	0.401

Obtained with ENE-101 *via* one-step one-pot chemoenzymatic cascade

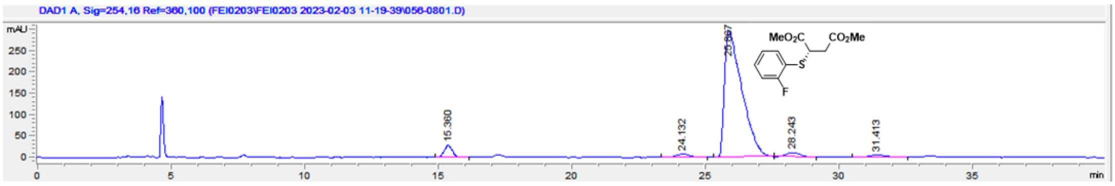

#	Time	Area	Height	Width	Area%	Symmetry
1	15.36	595.7	28.1	0.3276	3.977	0.888
2	24.132	273.3	7.5	0.5527	1.825	0.914
3	25.867	13469.2	297.6	0.6641	89.928	0.3
4	28.243	377	9.9	0.5828	2.517	0.859
5	31.413	262.5	5.4	0.6183	1.753	0.85

HPLC analysis of **2ac**: Chiralpak® IG column (4.6 mm × 250 mm, 5 µm); detected at 254 nm; heptane/EtOH = 90/10; flow rate: 1.0 mL/min.

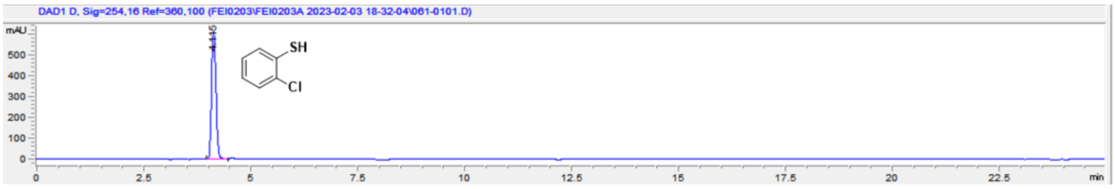

#	Time	Area	Height	Width	Area%	Symmetry
1	4.115	4428.8	613.8	0.1162	100.000	0.774

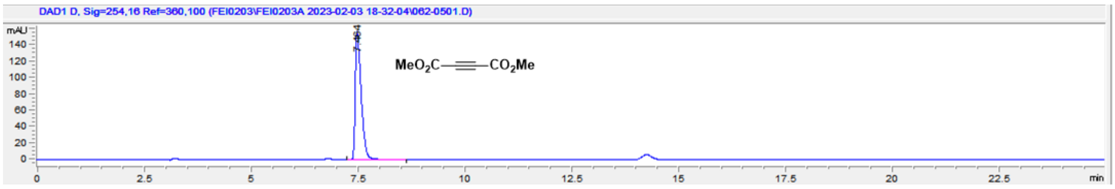

#	Time	Area	Height	Width	Area%	Symmetry
1	7.464	1514.1	156.4	0.1449	100.000	0.432

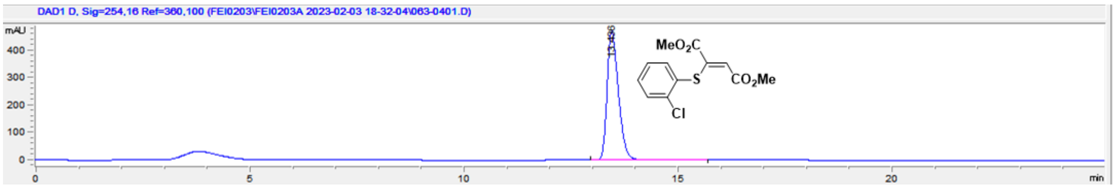

#	Time	Area	Height	Width	Area%	Symmetry
1	13.436	8617.1	468.3	0.2827	100.000	0.633

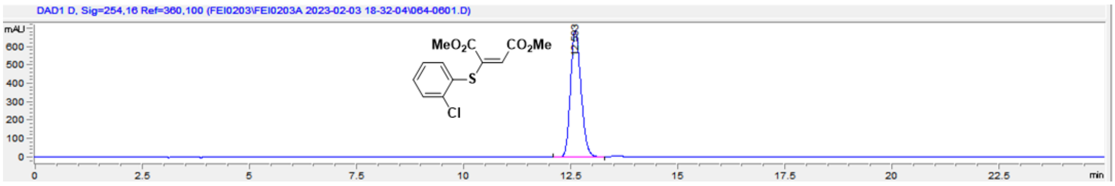

#	Time	Area	Height	Width	Area%	Symmetry
1	12.593	11825.2	688.4	0.2684	100.000	0.768

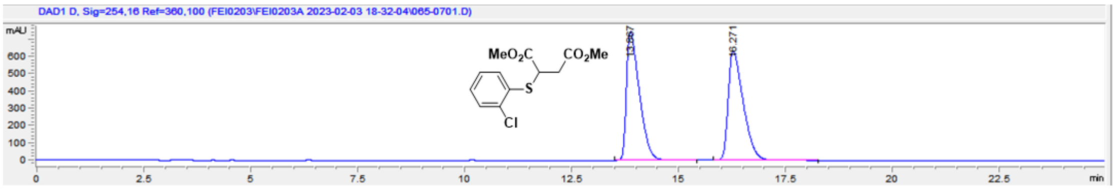

#	Time	Area	Height	Width	Area%	Symmetry
1	13.867	15619.4	745.3	0.3185	50.201	0.42
2	16.271	15494.3	634.4	0.3689	49.799	0.478

Obtained with ENE-101 *via* one-step one-pot chemoenzymatic cascade

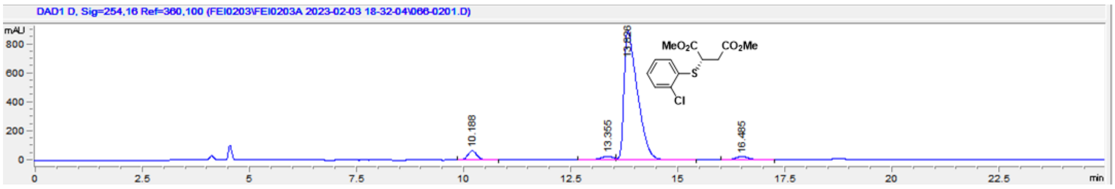

#	Time	Area	Height	Width	Area%	Symmetry
1	10.188	939	62.9	0.2321	4.377	0.897
2	13.355	556	25.9	0.3281	2.592	1.401
3	13.826	19443.3	886.2	0.326	90.628	0.388
4	16.485	515.6	23.3	0.3439	2.403	0.896

HPLC analysis of **2ad**: Chiralpak® IG column (4.6 mm × 250 mm, 5 μm); detected at 230 nm; heptane/EtOH = 90/10; flow rate: 1.0 mL/min.

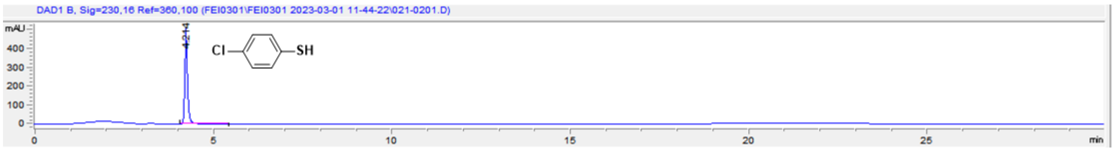

#	Time	Area	Height	Width	Area%	Symmetry
1	4.214	3173.4	508.8	0.0968	100.000	0.74

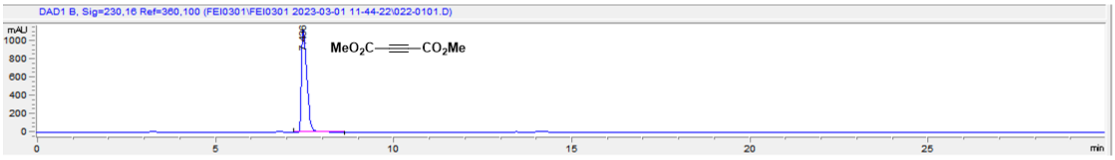

#	Time	Area	Height	Width	Area%	Symmetry
1	7.436	11705.2	1120.1	0.1599	100.000	0.419

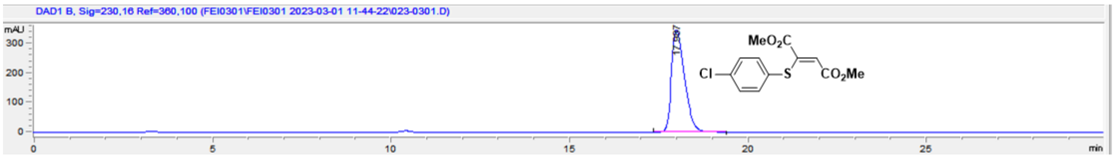

#	Time	Area	Height	Width	Area%	Symmetry
1	17.987	8753.4	347.4	0.382	100.000	0.615

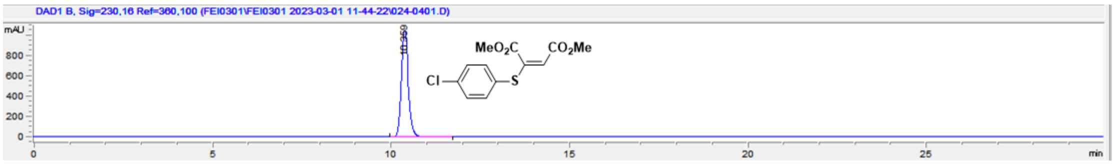

#	Time	Area	Height	Width	Area%	Symmetry
1	10.359	14642.9	1053.1	0.2142	100.000	0.776

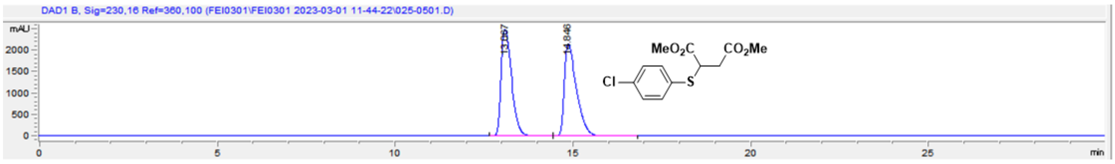

#	Time	Area	Height	Width	Area%	Symmetry
1	13.067	50980.3	2503.2	0.3156	49.827	0.496
2	14.846	51334.6	2151.5	0.3604	50.173	0.405

Obtained with ENE-101 *via* one-step one-pot chemoenzymatic cascade

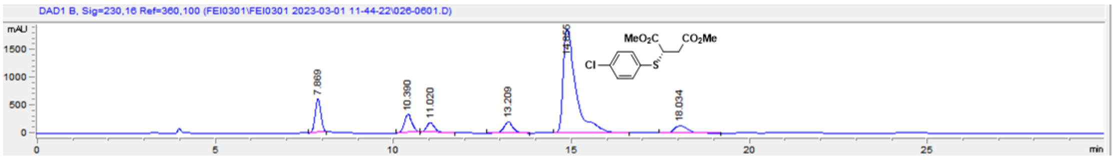

#	Time	Area	Height	Width	Area%	Symmetry
1	7.869	6439.2	611.4	0.1629	9.487	0.925
2	10.39	5126.2	326.4	0.2452	7.552	0.806
3	11.02	2903.1	177.2	0.2552	4.277	0.793
4	13.209	3598.2	201.4	0.2764	5.301	0.892
5	14.855	46661.7	1861.1	0.3705	68.747	0.37
6	18.034	3146.3	129.7	0.3771	4.635	0.769

HPLC analysis of **2ag**: Chiralpak® ID column (4.6 mm × 250 mm, 5 μm); detected at 230 nm; heptane/EtOH = 90/10; flow rate: 1.0 mL/min.

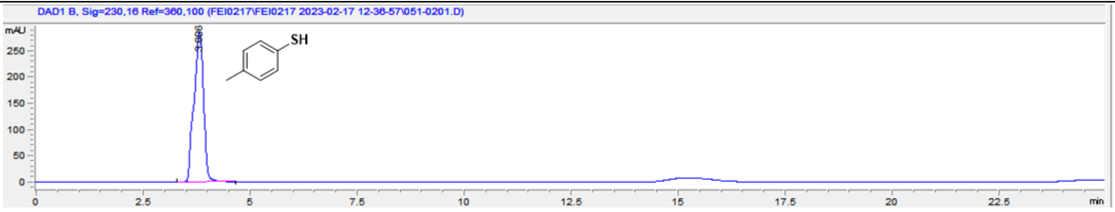

#	Time	Area	Height	Width	Area%	Symmetry
1	3.806	4494.4	285.3	0.2298	100.000	1.284

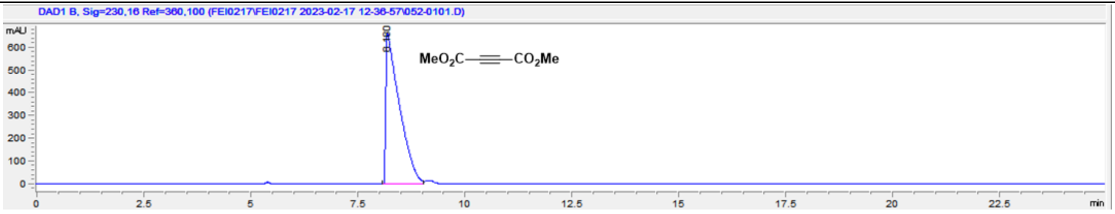

#	Time	Area	Height	Width	Area%	Symmetry
1	8.18	14713.7	665.2	0.2961	100.000	0.128

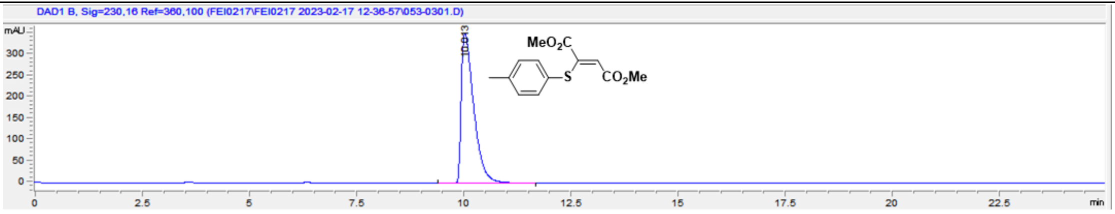

#	Time	Area	Height	Width	Area%	Symmetry
1	10.013	7161.3	353.5	0.3043	100.000	0.373

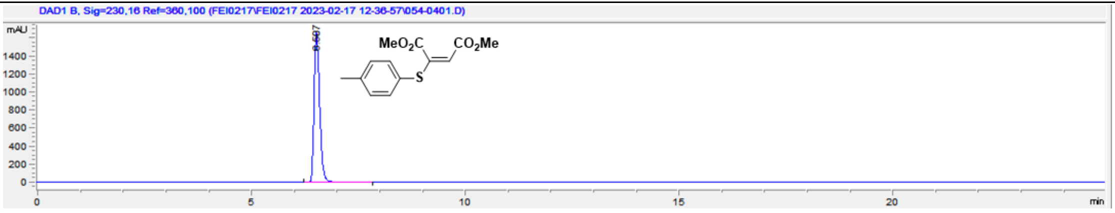

#	Time	Area	Height	Width	Area%	Symmetry
1	6.507	15165.9	1669	0.14	100.000	0.675

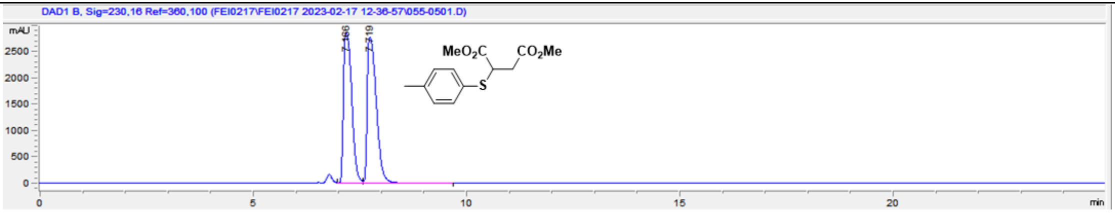

#	Time	Area	Height	Width	Area%	Symmetry
1	7.166	38941	2857.3	0.219	48.420	0.489
2	7.719	41482.6	2773.1	0.2365	51.580	0.382

Obtained with ENE-101 *via* one-step one-pot chemoenzymatic cascade

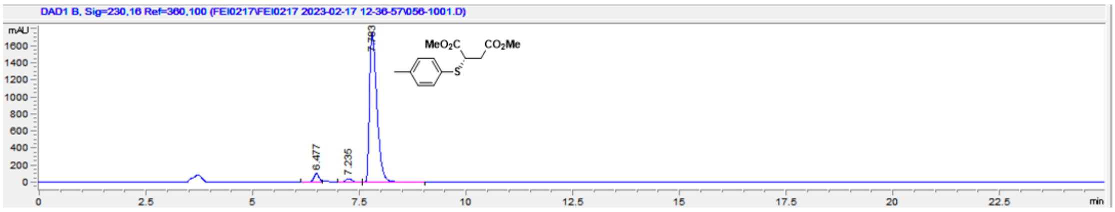

#	Time	Area	Height	Width	Area%	Symmetry
1	6.477	921.2	105.3	0.132	4.067	0.91
2	7.235	493.6	43.5	0.1765	2.179	0.847
3	7.783	21234.4	1757.2	0.185	93.754	0.506

HPLC analysis of **2ah**: Chiralpak® IG column (4.6 mm × 250 mm, 5 μm); detected at 230 nm; heptane/EtOH = 90/10; flow rate: 1.0 mL/min.

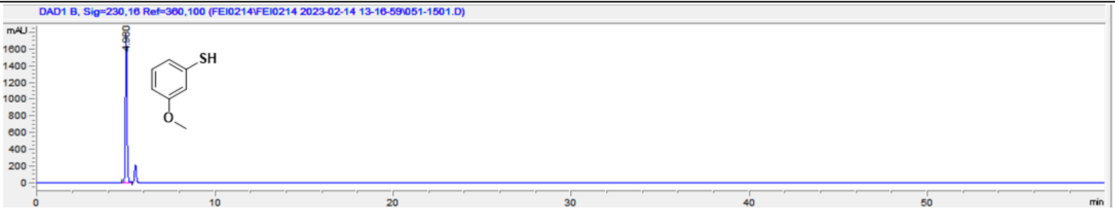

#	Time	Area	Height	Width	Area%	Symmetry
1	4.98	10540.7	1799.1	0.0904	100.000	0.786

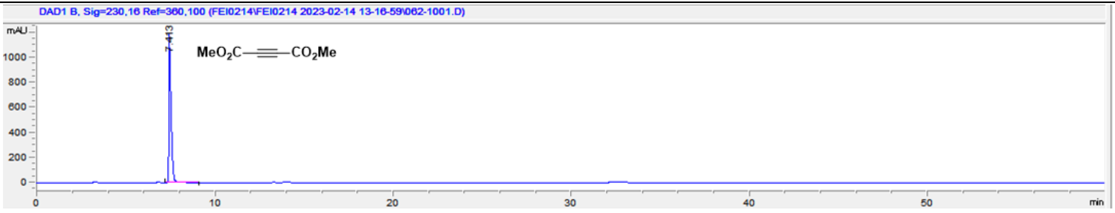

#	Time	Area	Height	Width	Area%	Symmetry
1	7.413	12371.2	1197.5	0.1585	100.000	0.408

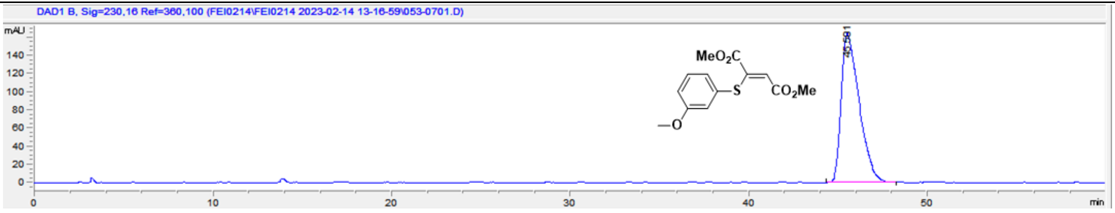

#	Time	Area	Height	Width	Area%	Symmetry
1	45.501	11441.9	164.7	1.0463	100.000	0.484

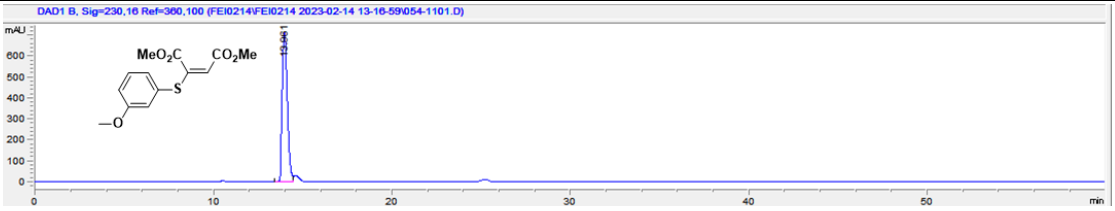

#	Time	Area	Height	Width	Area%	Symmetry
1	13.961	13748.5	715.4	0.2982	100.000	0.751

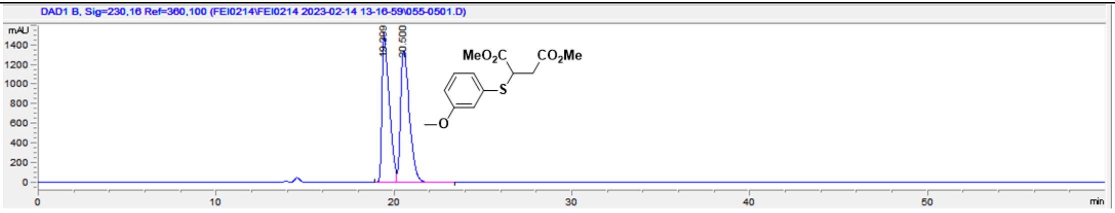

#	Time	Area	Height	Width	Area%	Symmetry
1	19.399	44893	1526.8	0.447	49.456	0.398
2	20.5	45881.3	1341.7	0.509	50.544	0.446

Obtained with ENE-101 *via* one-step one-pot chemoenzymatic cascade

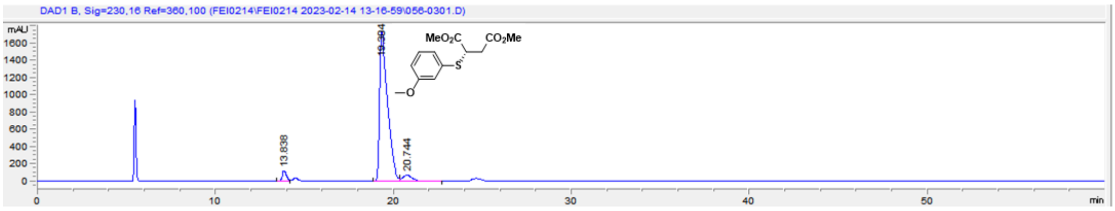

#	Time	Area	Height	Width	Area%	Symmetry
1	13.838	2243.4	121	0.2882	3.709	0.881
2	19.304	55394.5	1735.1	0.4765	91.591	0.342
3	20.744	2842.2	75.4	0.5635	4.699	0.768

HPLC analysis of **2ai**: Chiralpak® IG column (4.6 mm × 250 mm, 5 μm); detected at 230 nm; heptane/EtOH = 90/10; flow rate: 1.0 mL/min.

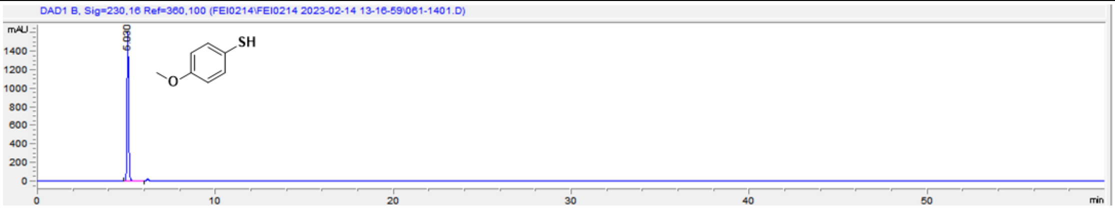

#	Time	Area	Height	Width	Area%	Symmetry
1	5.03	9437.8	1612.7	0.0903	100.000	0.788

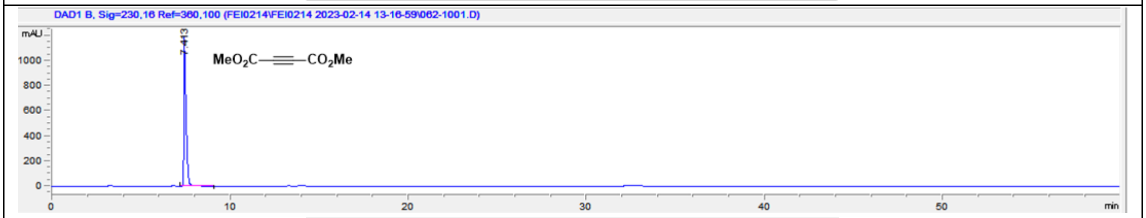

#	Time	Area	Height	Width	Area%	Symmetry
1	7.413	12371.2	1197.5	0.1585	100.000	0.408

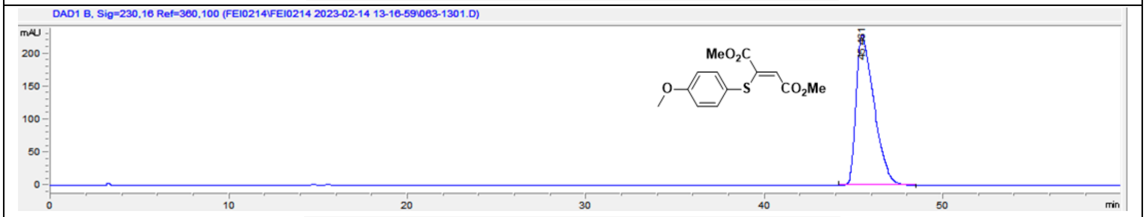

#	Time	Area	Height	Width	Area%	Symmetry
1	45.461	15911.2	227.7	1.0291	100.000	0.469

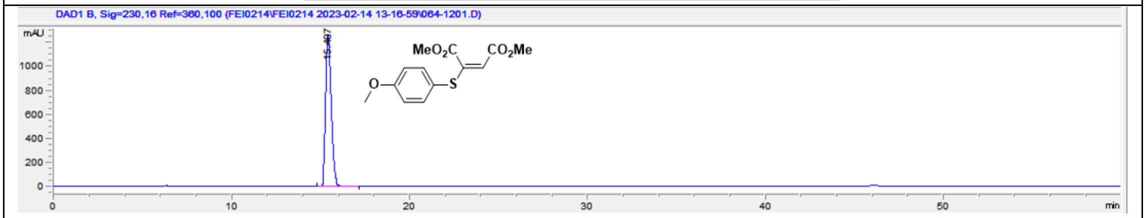

#	Time	Area	Height	Width	Area%	Symmetry
1	15.407	26479.5	1260.9	0.325	100.000	0.705

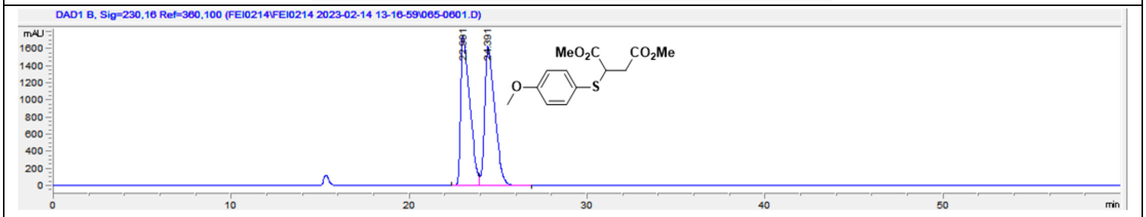

#	Time	Area	Height	Width	Area%	Symmetry
1	22.991	64193.3	1751.7	0.5655	49.563	0.37
2	24.391	65324.1	1627.3	0.6083	50.437	0.48

Obtained with ENE-101 *via* one-step one-pot chemoenzymatic cascade

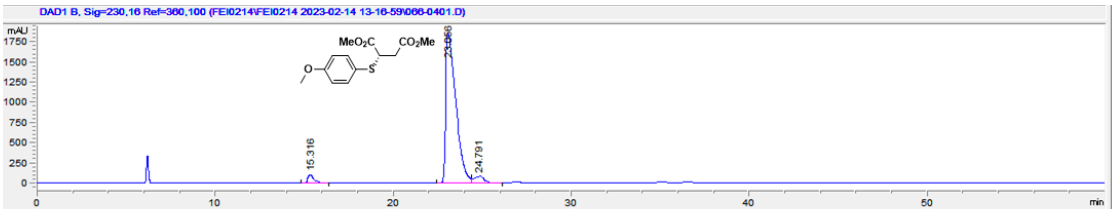

#	Time	Area	Height	Width	Area%	Symmetry
1	15.316	2504.7	109.2	0.3417	3.119	0.68
2	23.056	74458.5	1863.8	0.6041	92.717	0.32
3	24.791	3344.5	85.9	0.5825	4.165	0.898

HPLC analysis of **2aj**: Chiralcel® OJ-H column (4.6 mm × 250 mm, 5 μm); detected at 230 nm; heptane/EtOH = 90/10; flow rate: 1.0 mL/min.

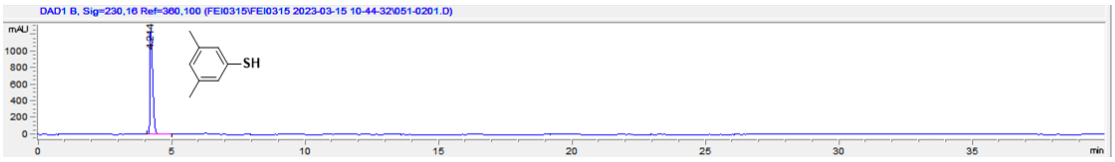

#	Time	Area	Height	Width	Area%	Symmetry
1	4.214	9333.9	1259.1	0.1185	100.000	0.556

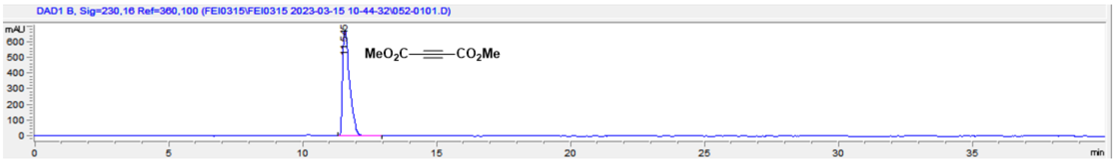

#	Time	Area	Height	Width	Area%	Symmetry
1	11.545	12247.2	681.1	0.2718	100.000	0.35

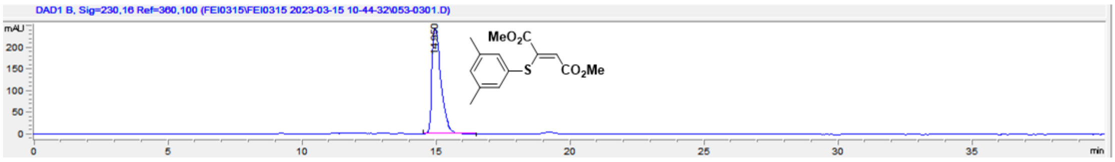

#	Time	Area	Height	Width	Area%	Symmetry
1	14.95	5690.9	242.1	0.3602	100.000	0.51

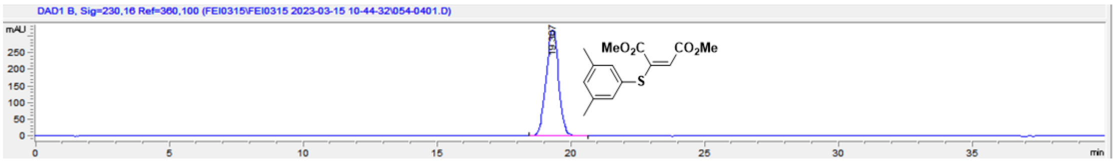

#	Time	Area	Height	Width	Area%	Symmetry
1	19.307	10378.2	320.9	0.5013	100.000	1.084

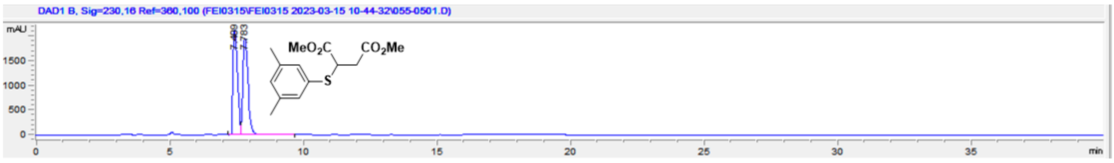

#	Time	Area	Height	Width	Area%	Symmetry
1	7.409	23486.9	2139.3	0.1721	48.271	0.553
2	7.783	25169	1961	0.1977	51.729	0.515

Obtained with ENE-101 *via* one-step one-pot chemoenzymatic cascade

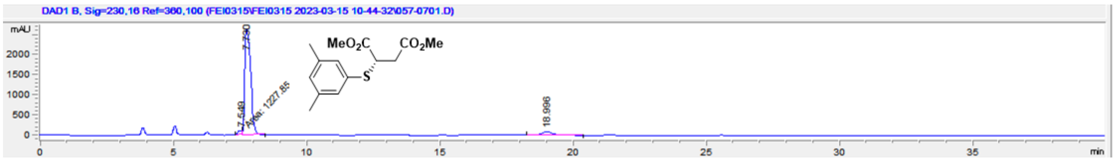

#	Time	Area	Height	Width	Area%	Symmetry
1	7.549	1227.8	102.5	0.1996	2.923	5.622
2	7.73	38270.2	2627.7	0.2339	91.101	0.423
3	18.996	2510.6	83.3	0.4697	5.976	0.904

HPLC analysis of **2ak**: Chiralcel® OD-H column (4.6 mm × 250 mm, 5 μm); detected at 230 nm; Hexane/EtOH = 90/10; flow rate: 1.0 mL/min.

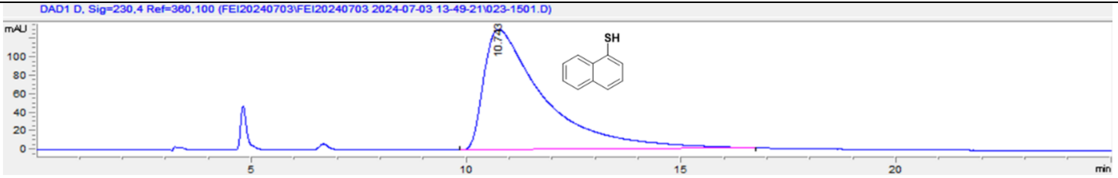

#	Time	Area	Height	Width	Area%	Symmetry
1	10.743	12759.4	130.8	1.2804	100.000	0.3

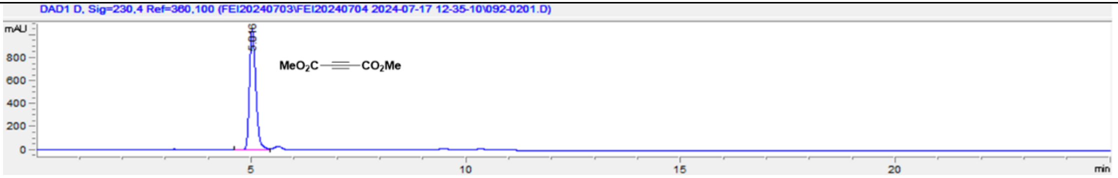

#	Time	Area	Height	Width	Area%	Symmetry
1	5.016	10734.6	1052	0.155	100.000	0.735

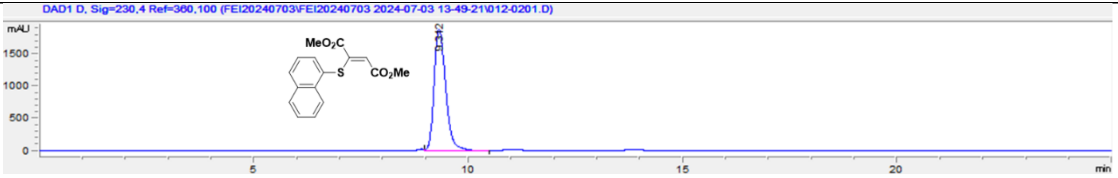

#	Time	Area	Height	Width	Area%	Symmetry
1	9.312	35589.8	1885.5	0.2922	100.000	0.703

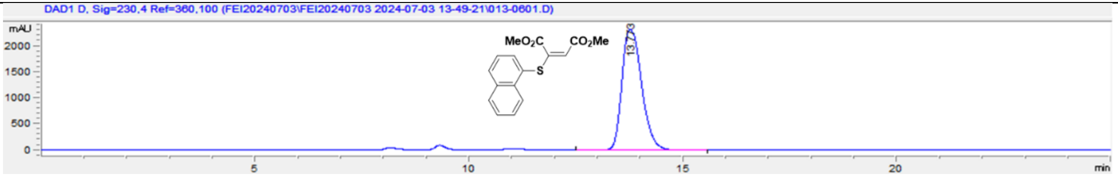

#	Time	Area	Height	Width	Area%	Symmetry
1	13.773	74218.9	2319.9	0.4452	100.000	0.734

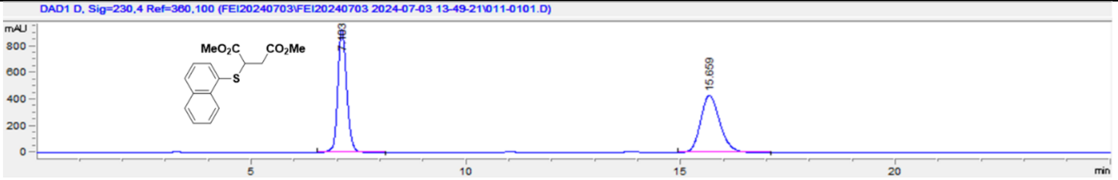

#	Time	Area	Height	Width	Area%	Symmetry
1	7.103	13259.4	926.7	0.2189	49.711	0.811
2	15.659	13413.8	431.6	0.4766	50.289	0.789

Obtained with ENE-101 *via* one-step one-pot chemoenzymatic cascade

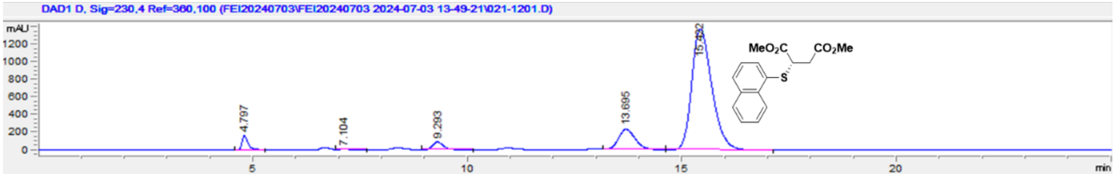

#	Time	Area	Height	Width	Area%	Symmetry
1	4.797	1620.3	161.9	0.1508	2.976	0.594
2	7.104	157	8.2	0.2762	0.288	0.53
3	9.293	1570.8	88.5	0.2691	2.885	0.702
4	13.695	6443	234.8	0.4181	11.834	0.823
5	15.422	44652.6	1375.2	0.5028	82.016	0.701

HPLC analysis of **2al**: Chiralpak® ID column (4.6 mm × 250 mm, 5 μm); detected at 230 nm; heptane/EtOH = 90/10; flow rate: 1.0 mL/min.

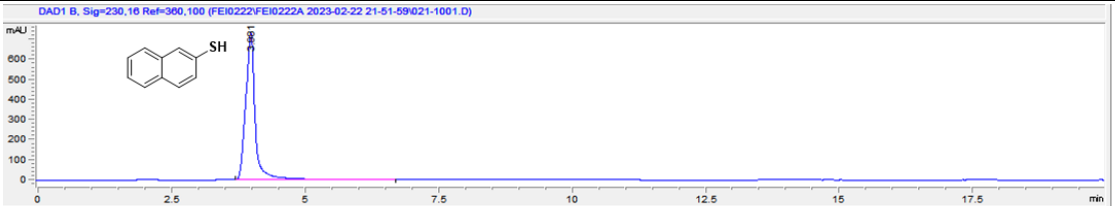

#	Time	Area	Height	Width	Area%	Symmetry
1	3.981	9155.7	735.9	0.1792	100.000	1.029

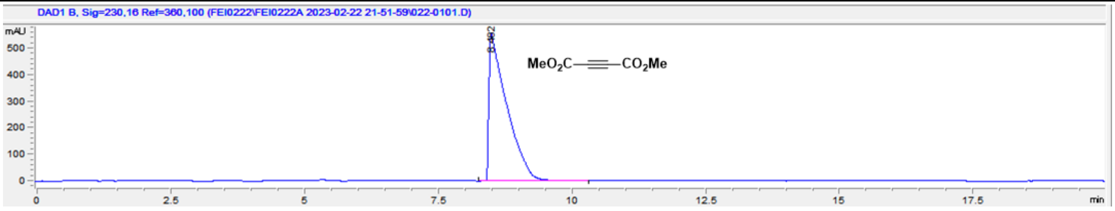

#	Time	Area	Height	Width	Area%	Symmetry
1	8.482	12884.4	556.3	0.3082	100.000	0.123

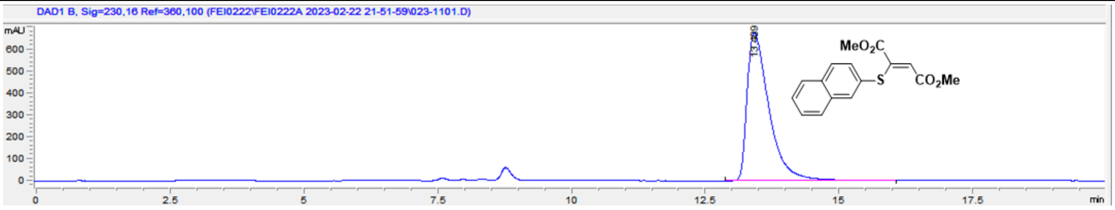

#	Time	Area	Height	Width	Area%	Symmetry
1	13.409	19231.8	675.5	0.4302	100.000	0.449

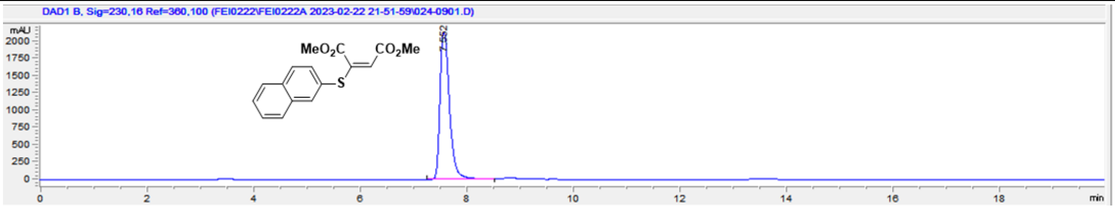

#	Time	Area	Height	Width	Area%	Symmetry
1	7.552	26482.5	2128.1	0.1932	100.000	0.641

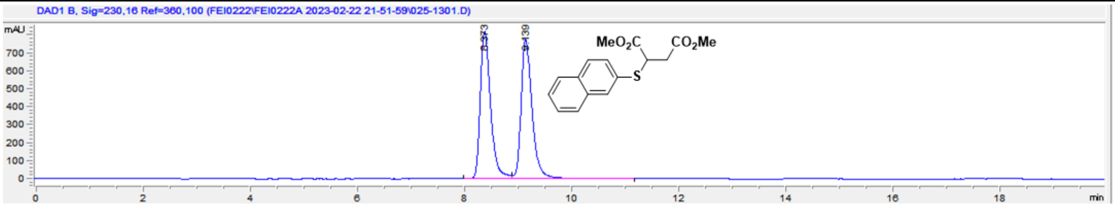

#	Time	Area	Height	Width	Area%	Symmetry
1	8.373	11037.7	819.1	0.2052	49.763	0.657
2	9.139	11142.9	776.6	0.2194	50.237	0.63

Obtained with ENE-101 *via* one-step one-pot chemoenzymatic cascade

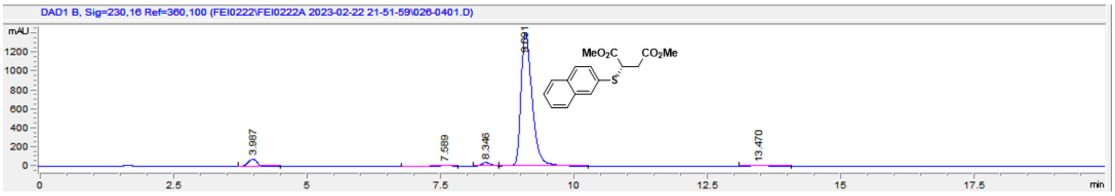

#	Time	Area	Height	Width	Area%	Symmetry
1	3.987	888.9	76.6	0.1913	3.990	1.296
2	7.589	217.6	15	0.2091	0.977	1.064
3	8.346	498	38.9	0.1934	2.235	0.821
4	9.091	20480.9	1413.5	0.221	91.928	0.635
5	13.47	193.9	9	0.3336	0.870	0.732

HPLC analysis of **2an**: Chiralpak® IG column (4.6 mm × 250 mm, 5 μm); detected at 230 nm; heptane/EtOH = 90/10; flow rate: 1.0 mL/min.

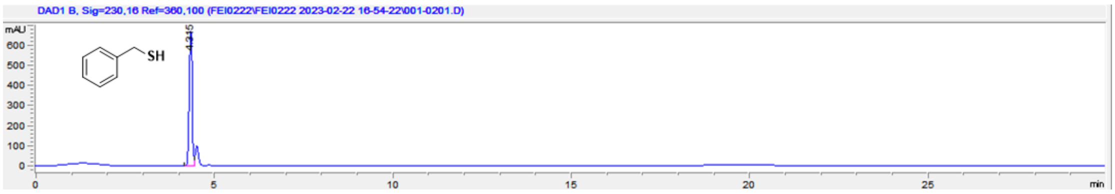

#	Time	Area	Height	Width	Area%	Symmetry
1	4.315	4082.2	675.9	0.0945	100.000	0.86

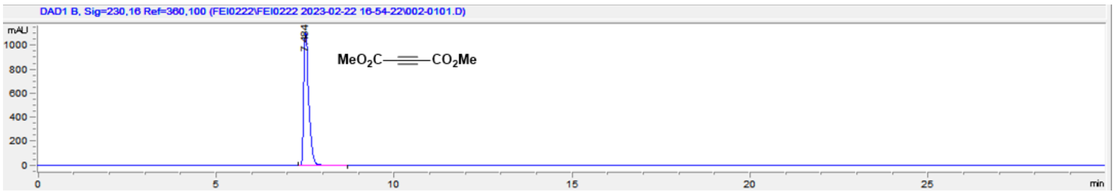

#	Time	Area	Height	Width	Area%	Symmetry
1	7.484	11458.6	1116.1	0.1578	100.000	0.428

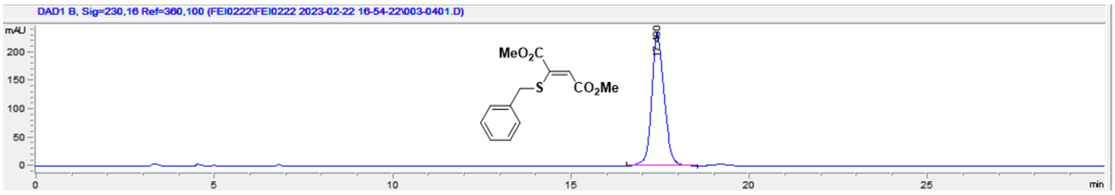

#	Time	Area	Height	Width	Area%	Symmetry
1	17.4	5697.3	235.6	0.3701	100.000	0.769

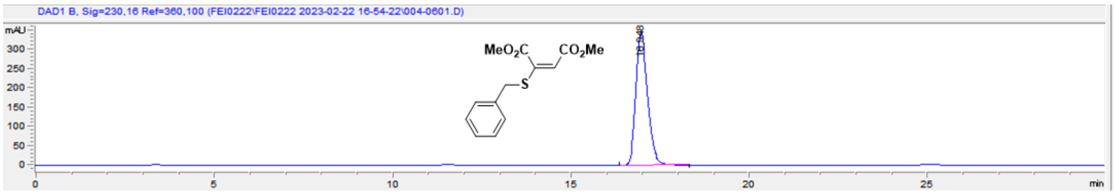

#	Time	Area	Height	Width	Area%	Symmetry
1	16.948	7952.6	346.1	0.3521	100.000	0.721

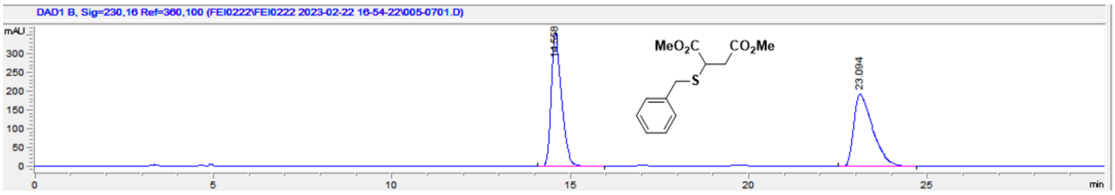

#	Time	Area	Height	Width	Area%	Symmetry
1	14.558	7166.1	357.9	0.3076	50.405	0.594
2	23.094	7051	194.1	0.5398	49.595	0.453

Obtained with ENE-101 *via* one-step one-pot chemoenzymatic cascade

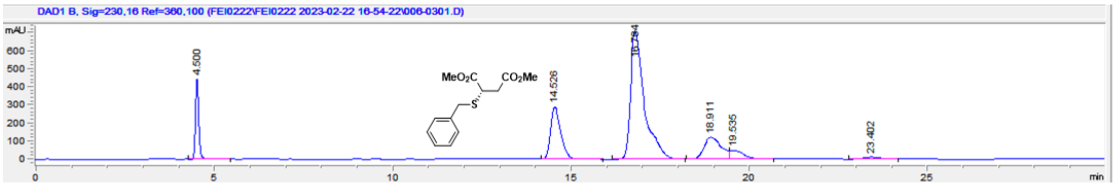

#	Time	Area	Height	Width	Area%	Symmetry
1	4.5	2622	442.5	0.0891	8.000	0.788
2	14.526	5649.7	288.9	0.3001	17.237	0.627
3	16.784	18946.6	707.2	0.3946	57.805	0.462
4	18.911	4030.2	120.1	0.5076	12.296	0.641
5	19.535	1179.5	46	0.3931	3.599	0.288
6	23.402	348.5	11.3	0.4766	1.063	0.861

HPLC analysis of **2bd**: Chiralpak® IG column (4.6 mm × 250 mm, 5 μm); detected at 214 nm; heptane/EtOH = 90/10; flow rate: 1.0 mL/min.

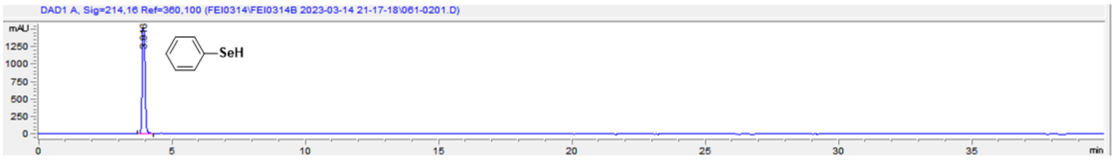

#	Time	Area	Height	Width	Area%	Symmetry
1	3.916	12833.1	1516.3	0.1367	100.000	1

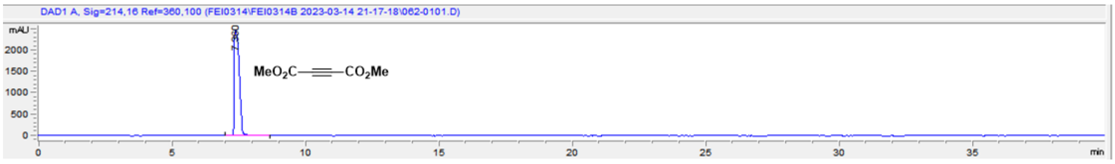

#	Time	Area	Height	Width	Area%	Symmetry
1	7.36	32180.7	2473	0.2118	100.000	0.362

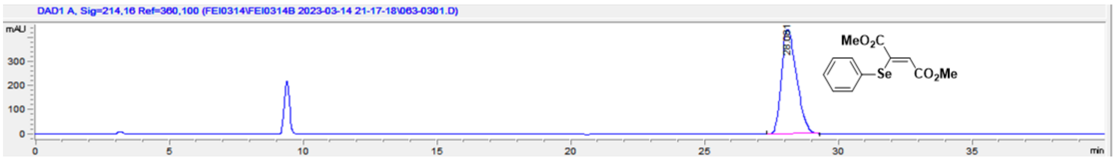

#	Time	Area	Height	Width	Area%	Symmetry
1	28.081	17178.3	437	0.6186	100.000	0.634

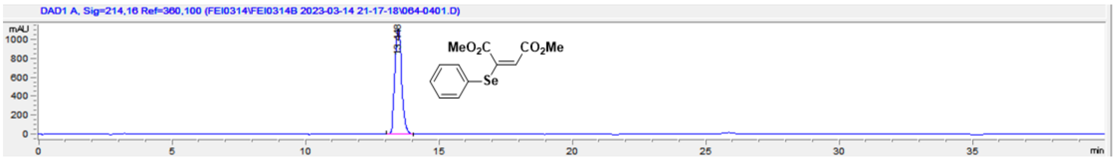

#	Time	Area	Height	Width	Area%	Symmetry
1	13.448	19625.6	1112	0.2759	100.000	0.791

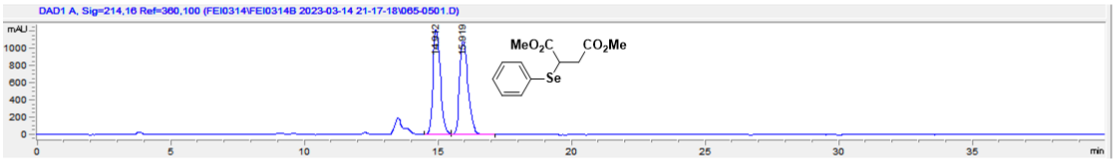

#	Time	Area	Height	Width	Area%	Symmetry
1	14.912	23696	1224.7	0.2997	49.817	0.71
2	15.919	23870.6	1089.2	0.3377	50.183	0.632

Obtained with ENE-101 *via* one-step one-pot chemoenzymatic cascade

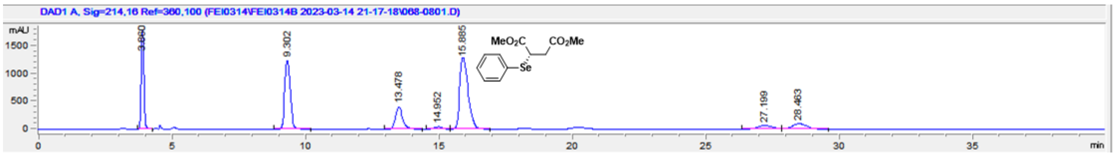

#	Time	Area	Height	Width	Area%	Symmetry
1	3.86	14860.2	1784	0.1352	19.947	0.652
2	9.302	17444.9	1238.4	0.2203	23.417	0.859
3	13.478	7154.6	396.2	0.2767	9.604	0.791
4	14.952	764.6	39.4	0.3025	1.026	0.999
5	15.885	27842.7	1289.4	0.3319	37.374	0.603
6	27.199	2611.3	64.3	0.6117	3.505	0.942
7	28.463	3819.7	98	0.6005	5.127	0.833

HPLC Analysis for ENE-101 Biocatalysed Enantioselective Reduction of 5 or 6 into (*S*)-7 or 8

HPLC analysis of **7aa**: Chiralpak® IG column (4.6 mm × 250 mm, 5 µm); detected at 254 nm; heptane/EtOH = 99/1; flow rate: 0.8 mL/min.

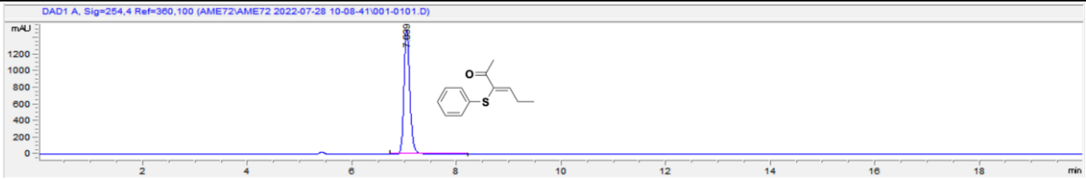

#	Time	Area	Height	Width	Area%	Symmetry
1	7.039	11661.3	1492.9	0.1211	100.000	0.785

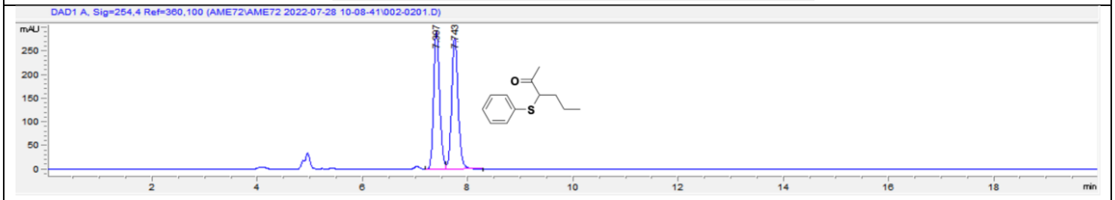

#	Time	Area	Height	Width	Area%	Symmetry
1	7.397	2335.1	291.7	0.1234	49.500	0.824
2	7.743	2382.2	277	0.1323	50.500	0.823

Obtained with ENE-101 from (*Z*)-**5aa**

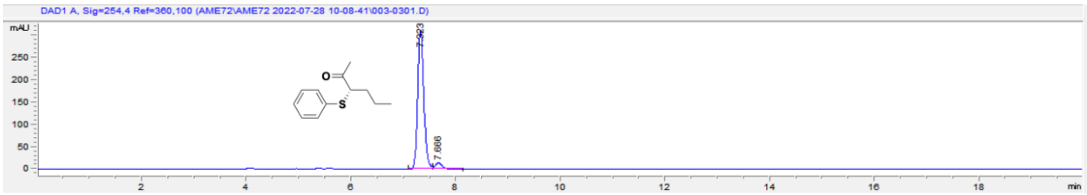

#	Time	Area	Height	Width	Area%	Symmetry
1	7.323	2529.2	311.4	0.1248	95.229	0.814
2	7.666	126.7	13.7	0.1377	4.771	0.843

HPLC analysis of **7ab**: Chiralcel® OJ-H column (4.6 mm × 250 mm, 5 µm); detected at 254 nm; heptane/EtOH = 90/10; flow rate: 0.8 mL/min.

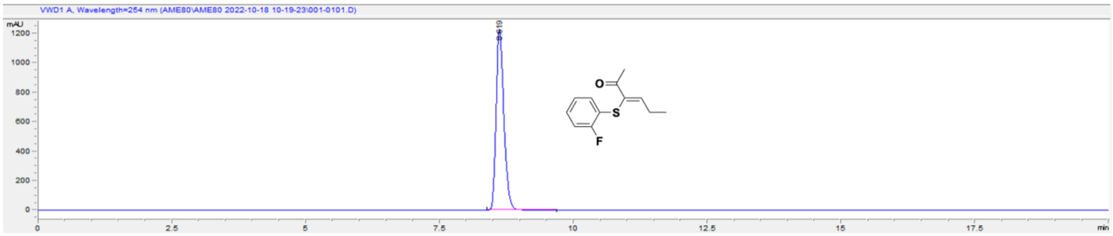

#	Time	Area	Height	Width	Area%	Symmetry
1	8.619	12386.7	1227.9	0.1541	100.000	0.711

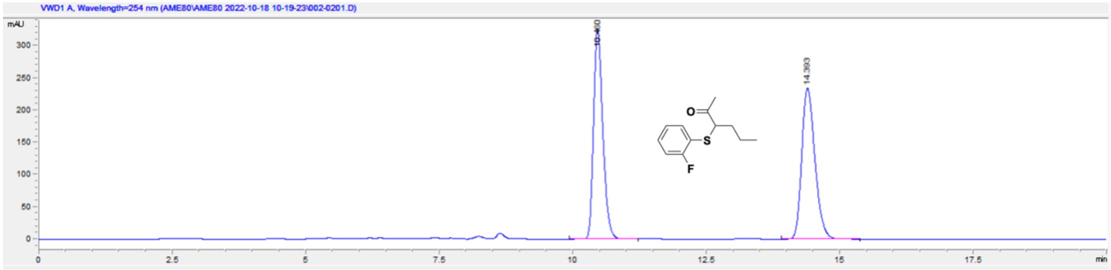

#	Time	Area	Height	Width	Area%	Symmetry
1	10.46	3914.5	324.3	0.186	49.346	0.701
2	14.392	4018.3	234.3	0.2656	50.654	0.731

Obtained with ENE-101 from (*Z*)-**5ab**

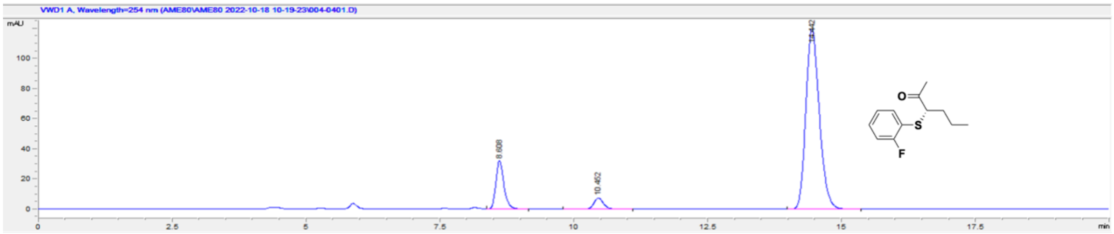

#	Time	Area	Height	Width	Area%	Symmetry
1	8.608	332.9	31.9	0.1595	13.269	0.757
2	10.452	86.2	7.2	0.1845	3.437	0.809
3	14.442	2089.5	119.6	0.2664	83.294	0.773

HPLC analysis of **7ac**: Chiralpak® IG column (4.6 mm × 250 mm, 5 μm); detected at 254 nm; heptane/EtOH = 99/1; flow rate: 0.8 mL/min.

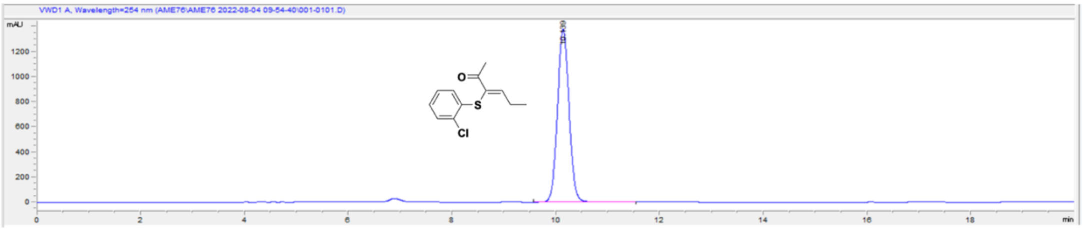

#	Time	Area	Height	Width	Area%	Symmetry
1	10.139	21310.5	1377.3	0.2417	100.000	0.904

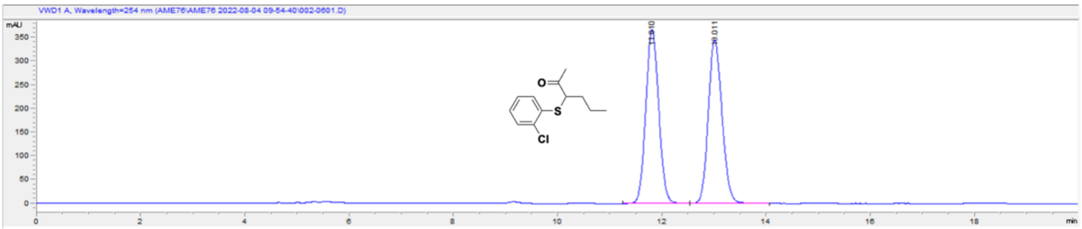

#	Time	Area	Height	Width	Area%	Symmetry
1	11.81	6314.2	367	0.2692	50.045	0.909
2	13.011	6302.8	344.9	0.286	49.955	0.82

Obtained with ENE-101 from (*Z*)-**5ac**

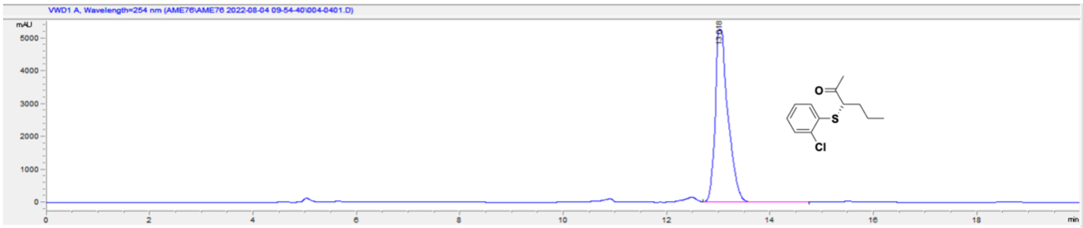

#	Time	Area	Height	Width	Area%	Symmetry
1	13.018	90670.7	5268.3	0.2255	100.000	0.485

HPLC analysis of **7ad**: Chiralpak® IG column (4.6 mm × 250 mm, 5 μm); detected at 254 nm; heptane/EtOH = 95/5; flow rate: 0.8 mL/min.

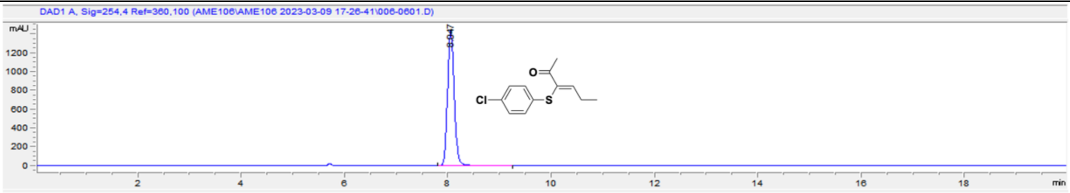

#	Time	Area	Height	Width	Area%	Symmetry
1	8.047	13078.7	1438.3	0.1401	100.000	0.821

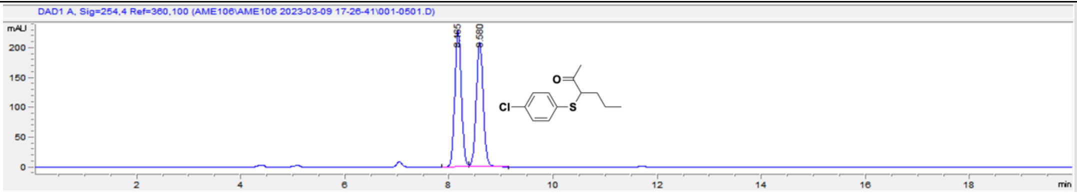

#	Time	Area	Height	Width	Area%	Symmetry
1	8.165	2024.5	229.2	0.1371	50.525	0.931
2	8.58	1982.4	209.6	0.1464	49.475	0.906

Obtained with ENE-101 from (*Z*)-**5ad**

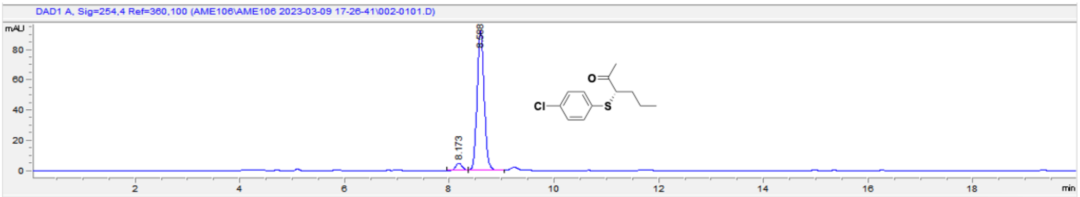

#	Time	Area	Height	Width	Area%	Symmetry
1	8.173	43.9	4.8	0.1397	4.817	0.925
2	8.588	866.8	92.3	0.1455	95.183	0.903

HPLC analysis of **7ae**: Chiralpak® IG column (4.6 mm × 250 mm, 5 μm); detected at 254 nm; heptane/EtOH = 90/10; flow rate: 0.8 mL/min.

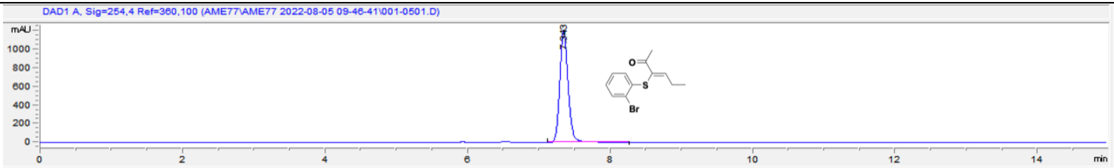

#	Time	Area	Height	Width	Area%	Symmetry
1	7.343	9837.1	1202.5	0.1274	100.000	0.79

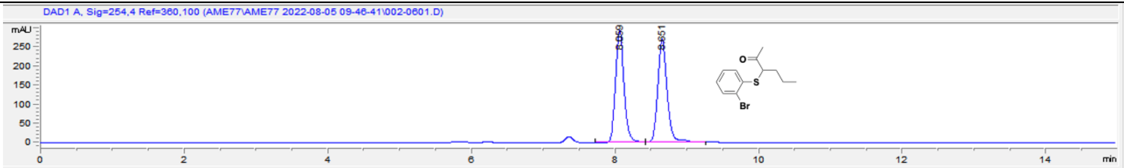

#	Time	Area	Height	Width	Area%	Symmetry
1	8.059	2421.5	293.6	0.1262	49.797	0.855
2	8.651	2441.2	271.2	0.139	50.203	0.8

Obtained with ENE-101 from (*Z*)-**5ae**

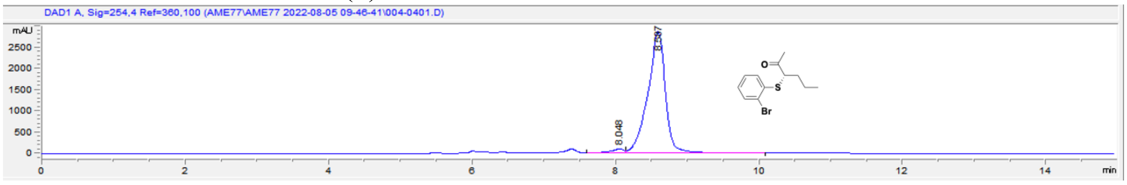

#	Time	Area	Height	Width	Area%	Symmetry
1	8.048	1274.4	97.2	0.1829	2.543	2.019
2	8.587	48845.2	2869.8	0.2446	97.457	1.302

HPLC analysis of **7af**: Chiralpak® OJ-H column (4.6 mm × 250 mm, 5 μm); detected at 254 nm; heptane/EtOH = 99/1; flow rate: 0.8 mL/min.

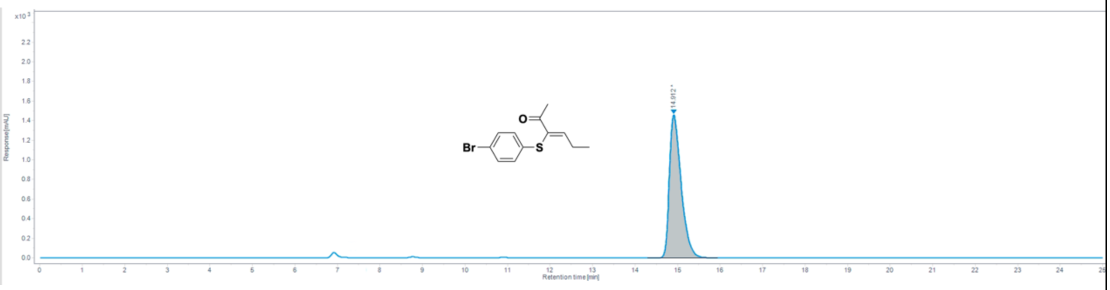

#	RT (min)	Area (mAU·s)	Area%	Height (mAU)	Symmetry	Width (min)
1	14.912	27974.502	100.000	1458.675	0.55805	1.648

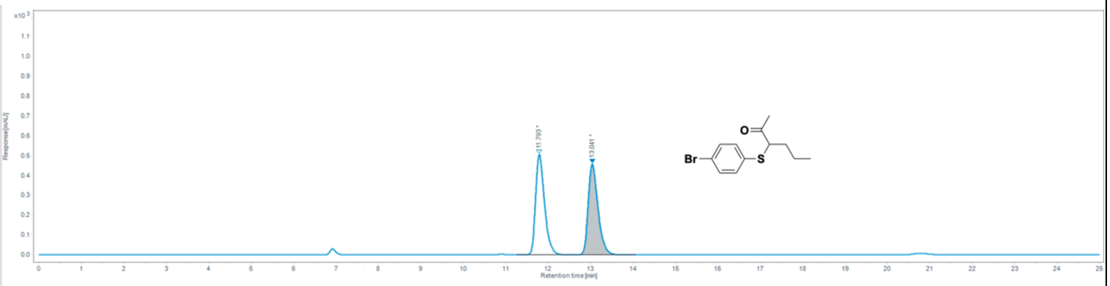

#	RT (min)	Area (mAU·s)	Area%	Height (mAU)	Symmetry	Width (min)
1	11.793	7403.665	49.992	506.507	0.66752	1.409
2	13.041	7406.122	50.008	458.855	0.66163	1.390

Obtained with ENE-101 from (*Z*)-**5af**

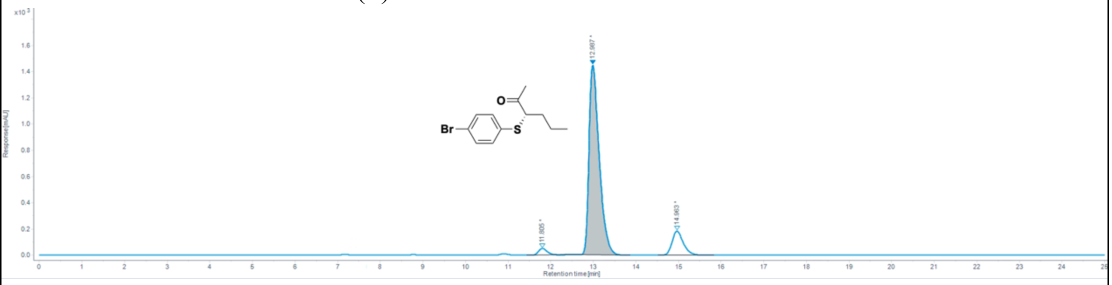

#	RT (min)	Area (mAU·s)	Area%	Height (mAU)	Symmetry	Width (min)
1	11.805	705.032	2.499	51.259	0.76426	1.052
2	12.987	24121.517	85.493	1446.332	0.54476	1.370
3	14.963	3388.211	12.009	183.409	0.72291	1.310

HPLC analysis of **7ag**: Chiralcel® OJ-H column (4.6 mm × 250 mm, 5 μm); detected at 254 nm; heptane/EtOH = 90/10; flow rate: 1.0 mL/min.

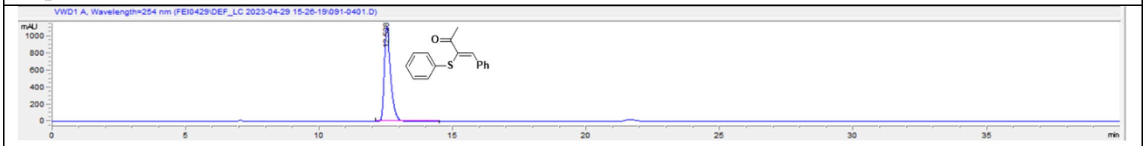

#	Time	Area	Height	Width	Area%	Symmetry
1	12.528	18785.3	1104.2	0.2596	100.000	0.603

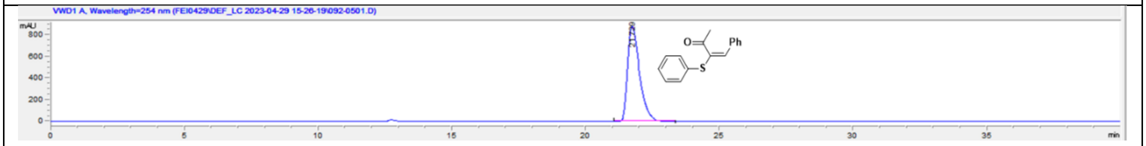

#	Time	Area	Height	Width	Area%	Symmetry
1	21.729	26870.2	878.7	0.4674	100.000	0.552

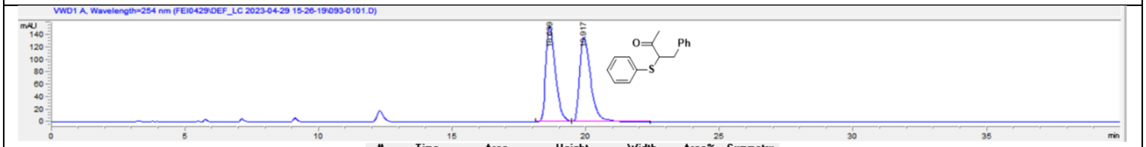

#	Time	Area	Height	Width	Area%	Symmetry
1	18.639	3782.9	153.4	0.3751	49.602	0.635
2	19.917	3843.6	135.8	0.4291	50.398	0.554

Obtained with ENE-101 from (*Z*)-**5ag** (5 h)

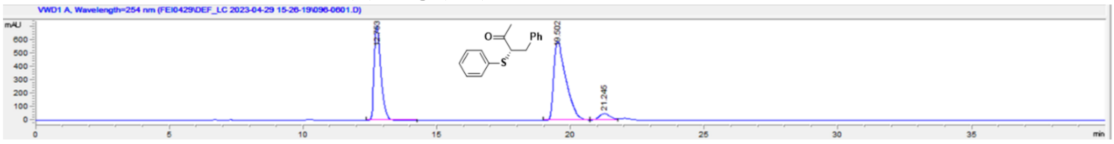

#	Time	Area	Height	Width	Area%	Symmetry
1	12.753	12118.7	707.9	0.2609	37.406	0.648
2	19.502	18778	587.7	0.4834	57.962	0.412
3	21.245	1500.7	50	0.4637	4.632	0.767

Obtained with ENE-101 from (*Z*)-**5ag** (19 h)

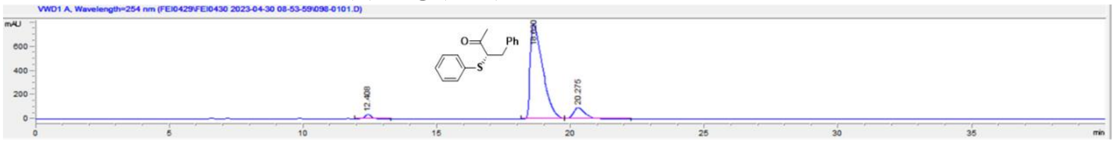

#	Time	Area	Height	Width	Area%	Symmetry
1	12.408	628.4	36	0.262	2.184	0.902
2	18.62	25219.7	781.6	0.4813	87.635	0.377
3	20.275	2930	93.3	0.4771	10.181	0.658

Obtained with ENE-101 from (*E*)-**5ag** (5 h)

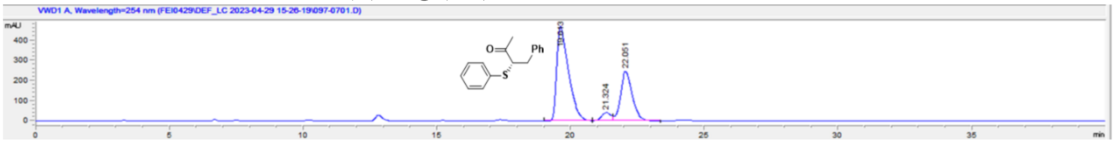

#	Time	Area	Height	Width	Area%	Symmetry
1	19.613	14508.2	469.7	0.471	63.142	0.45
2	21.324	1049.4	40.9	0.3954	4.567	1.003
3	22.051	7419.4	245.9	0.4627	32.290	0.755

Obtained with ENE-101 from (*E*)-**5ag** (19 h)

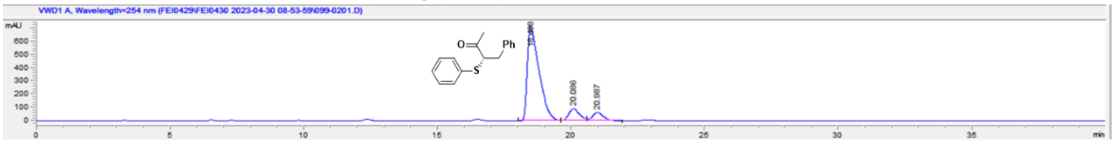

#	Time	Area	Height	Width	Area%	Symmetry
1	18.488	21890	711	0.464	83.822	0.401
2	20.086	2502.2	89.1	0.4354	9.582	0.693
3	20.987	1722.6	61.8	0.4245	6.596	0.847

HPLC analysis of **7ah**: Chiralcel® OJ-H column (4.6 mm × 250 mm, 5 µm); detected at 254 nm; heptane/EtOH = 80/20; flow rate: 0.8 mL/min.

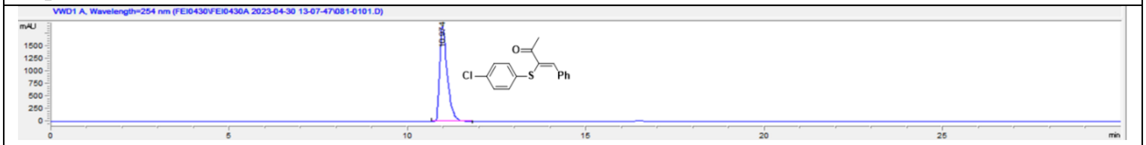

#	Time	Area	Height	Width	Area%	Symmetry
1	10.974	28222.5	1885.8	0.227	100.000	0.55

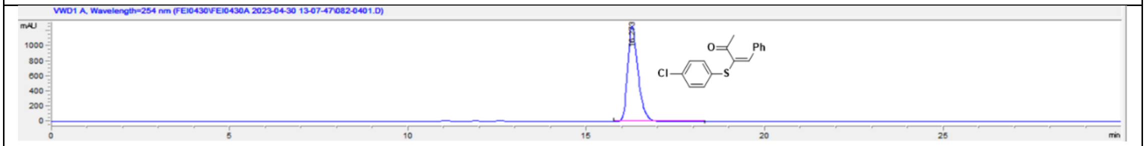

#	Time	Area	Height	Width	Area%	Symmetry
1	16.273	25546.1	1259.4	0.3095	100.000	0.689

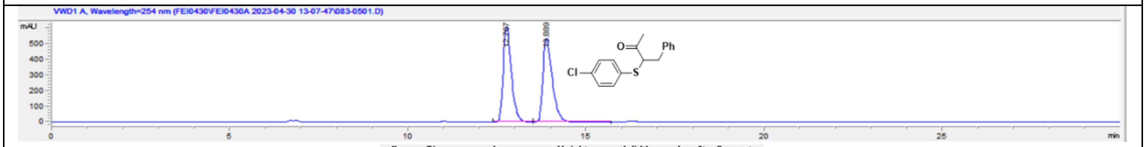

#	Time	Area	Height	Width	Area%	Symmetry
1	12.767	9633.2	607.4	0.2433	49.895	0.654
2	13.889	9673.9	533.2	0.2786	50.105	0.584

Obtained with ENE-101 from (*Z*)-**5ah** (1.5 h)

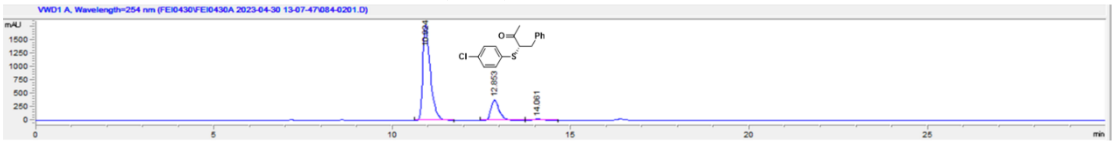

#	Time	Area	Height	Width	Area%	Symmetry
1	10.924	26369	1772.9	0.2259	80.251	0.559
2	12.853	6056.9	380.3	0.244	18.434	0.689
3	14.061	432.3	25.1	0.2622	1.316	0.761

Obtained with ENE-101 from (*Z*)-**5ah** (23 h)

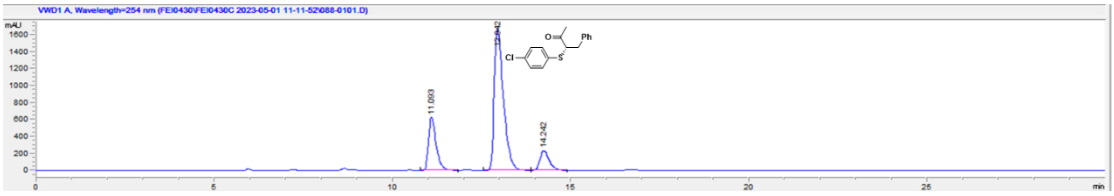

#	Time	Area	Height	Width	Area%	Symmetry
1	11.093	9205.7	623.5	0.2247	21.121	0.609
2	12.942	30183.6	1677.7	0.274	69.251	0.523
3	14.242	4196.2	231.5	0.2785	9.627	0.688

Obtained with ENE-101 from (*E*)-**5ah** (1.5 h)

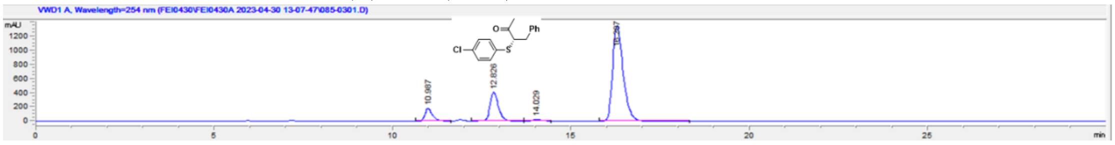

#	Time	Area	Height	Width	Area%	Symmetry
1	10.987	2614.8	177.2	0.2246	7.030	0.643
2	12.826	6694.5	410.3	0.2486	17.999	0.74
3	14.029	415.8	22.5	0.2763	1.118	0.657
4	16.287	27468.3	1345.6	0.311	73.853	0.682

Obtained with ENE-101 from (*E*)-**5ah** (23 h)

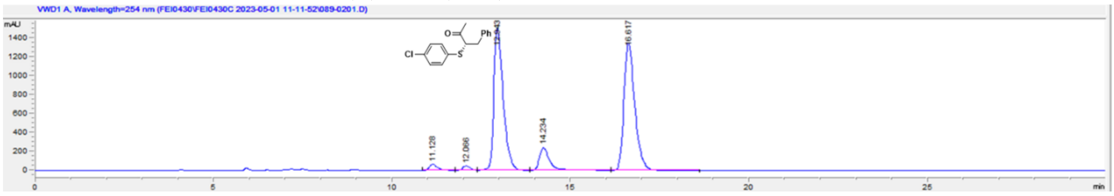

#	Time	Area	Height	Width	Area%	Symmetry
1	11.128	992.3	63.6	0.2373	1.602	0.634
2	12.066	659.2	46.7	0.2172	1.064	0.811
3	12.943	27162.8	1508.8	0.2741	43.856	0.569
4	14.234	4848.8	239.6	0.3031	7.829	0.546
5	16.617	28273.1	1346.4	0.3207	45.649	0.677

HPLC analysis of **7ai**: Chiralcel® OJ-H column (4.6 mm × 250 mm, 5 μm); detected at 254 nm; heptane/EtOH = 80/20; flow rate: 0.8 mL/min.

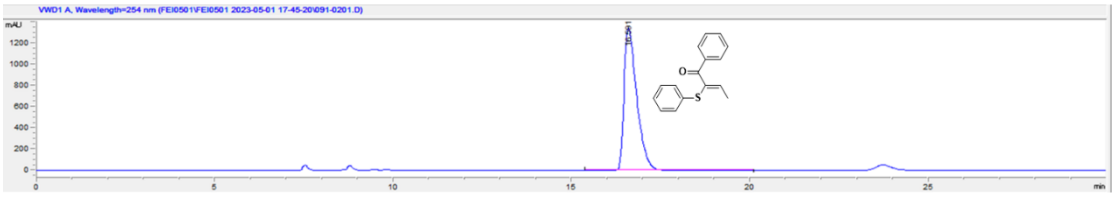

#	Time	Area	Height	Width	Area%	Symmetry
1	16.591	33098.2	1343.3	0.3748	100.000	0.487

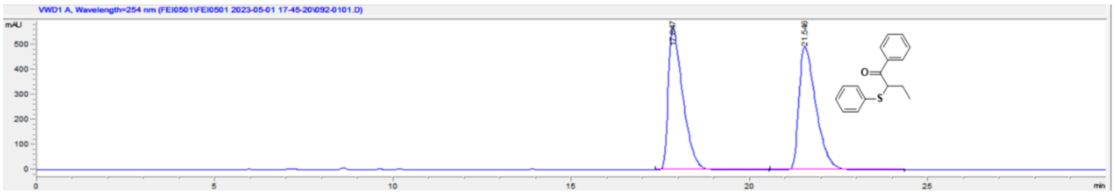

#	Time	Area	Height	Width	Area%	Symmetry
1	17.847	16118.1	567.9	0.4301	49.817	0.446
2	21.546	16236.6	493.1	0.5006	50.183	0.499

Obtained with ENE-101 from (*Z*)-**5ai**

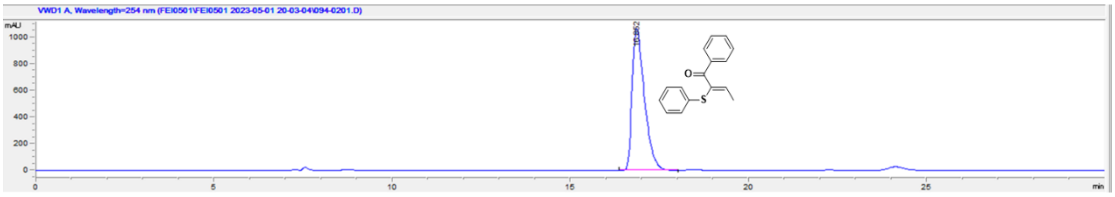

#	Time	Area	Height	Width	Area%	Symmetry
1	16.852	25656.2	1064.8	0.3685	100.000	0.521

HPLC analysis of **7aj**: Chiralpak® IG column (4.6 mm × 250 mm, 5 μm); detected at 254 nm; heptane/EtOH = 90/10; flow rate: 1.0 mL/min.

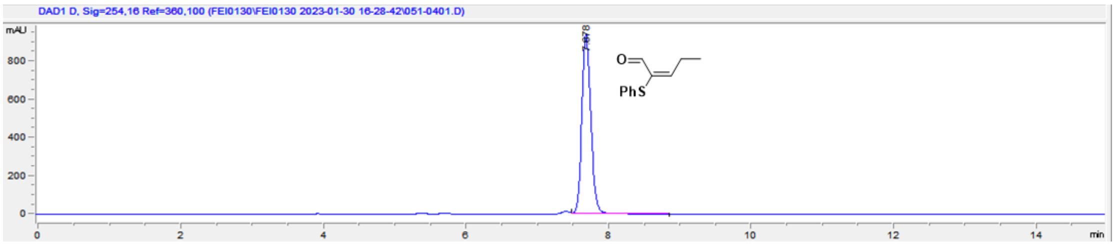

#	Time	Area	Height	Width	Area%	Symmetry
1	7.678	8289.2	941.7	0.1367	100.000	0.811

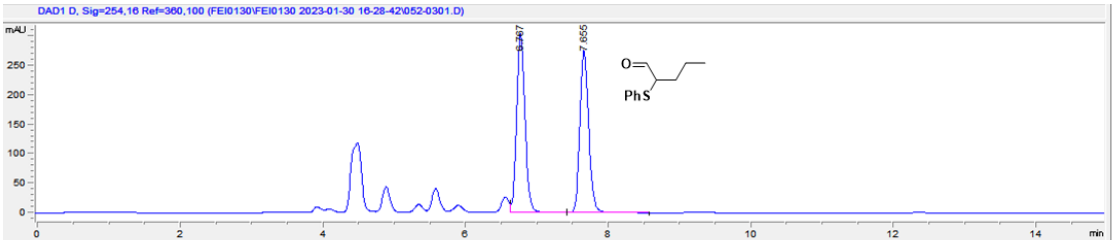

#	Time	Area	Height	Width	Area%	Symmetry
1	6.767	2453.8	303.5	0.1243	50.874	0.806
2	7.655	2369.4	275.1	0.1325	49.126	0.785

Obtained with ENE-101 from (*Z*)-**5aj** (1 h)

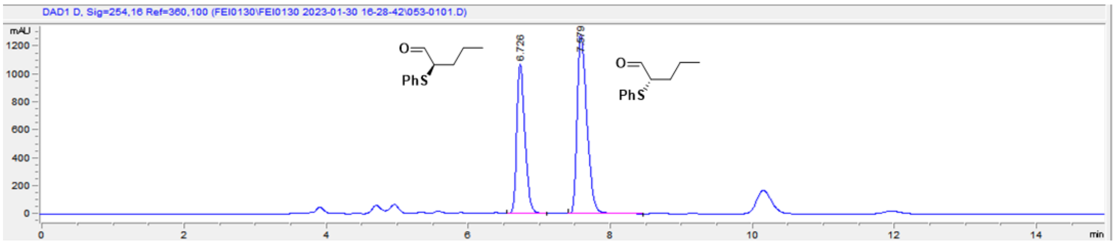

#	Time	Area	Height	Width	Area%	Symmetry
1	6.726	8498.8	1068.5	0.1228	40.931	0.672
2	7.579	12265	1281.4	0.1477	59.069	0.557

Note: In this case, the full conversion of the substrate to the desired product was also confirmed by ^{1}H NMR analysis of the crude reaction mixture.

Obtained with ENE-101 from (*Z*)-**5aj** (2 h)

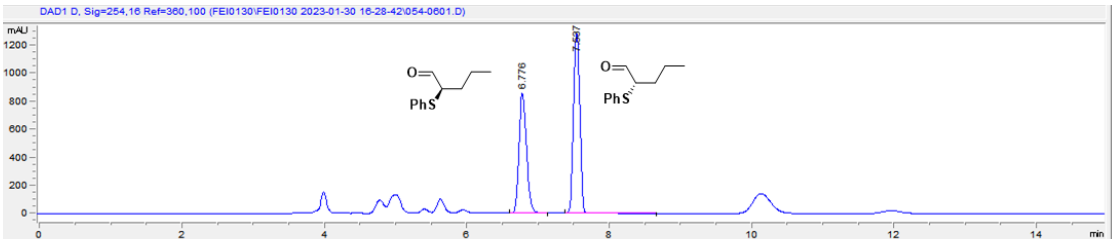

#	Time	Area	Height	Width	Area%	Symmetry
1	6.776	6270.8	856.2	0.1134	43.964	0.75
2	7.537	7992.8	1280.5	0.1028	56.036	0.855

Note: In this case, the full conversion of the substrate to the desired product was also confirmed by ^{1}H NMR analysis of the crude reaction mixture.

HPLC analysis of **7ak**: Chiralpak® IG column (4.6 mm × 250 mm, 5 μm); detected at 254 nm; heptane/EtOH = 95/5; flow rate: 1.0 mL/min.

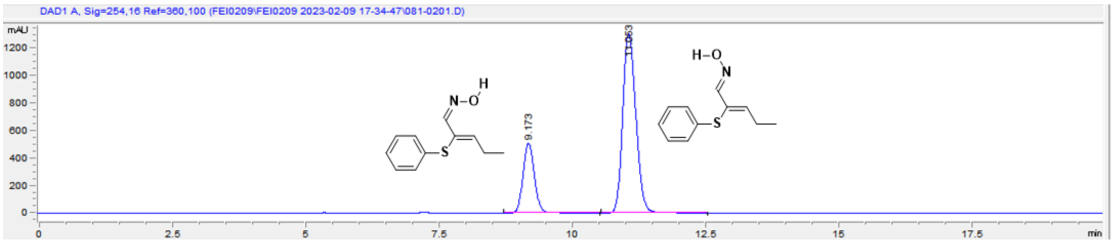

#	Time	Area	Height	Width	Area%	Symmetry
1	9.173	7548.5	506.5	0.2319	25.165	0.865
2	11.053	22447.7	1306.7	0.2684	74.835	0.781

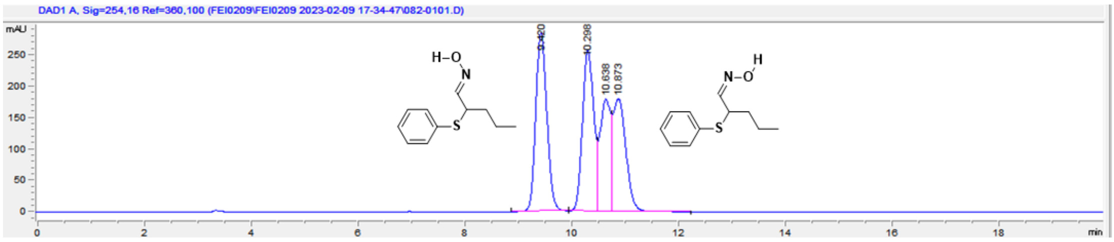

#	Time	Area	Height	Width	Area%	Symmetry
1	9.42	4109.4	284.4	0.2246	30.734	0.847
2	10.298	3812.4	255.6	0.228	28.513	0.879
3	10.638	2471.2	178.5	0.2015	18.482	1.203
4	10.873	2977.9	178.9	0.2482	22.272	0.648

Obtained with ENE-101 from (1*Z*,2*Z*)-**5ak**/(1*E*,2*Z*)-**5ak** = 0.4/1

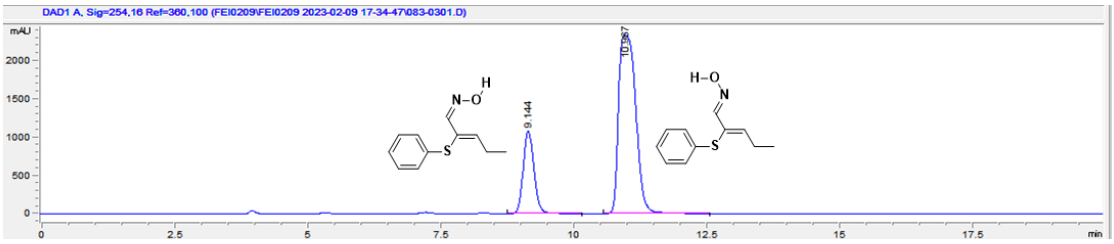

#	Time	Area	Height	Width	Area%	Symmetry
1	9.144	15255.4	1077.8	0.2191	22.565	0.813
2	10.967	52350	2338.6	0.3612	77.435	0.59

Note: In this case, ^{1}H NMR analysis of the crude reaction mixture also confirmed there was no desired products in this reaction.

HPLC analysis of **7al**: Chiralpak® IG column (4.6 mm × 250 mm, 5 μm); detected at 254 nm; heptane/EtOH = 90/10; flow rate: 1.0 mL/min.

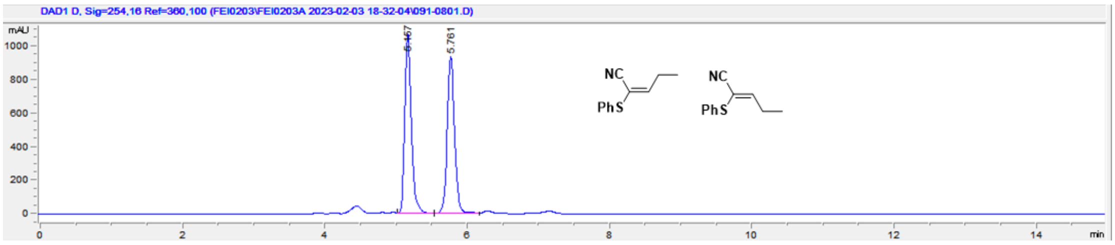

#	Time	Area	Height	Width	Area%	Symmetry
1	5.157	6892.4	1071.1	0.0991	50.545	0.749
2	5.761	6743.7	932.5	0.1104	49.455	0.843

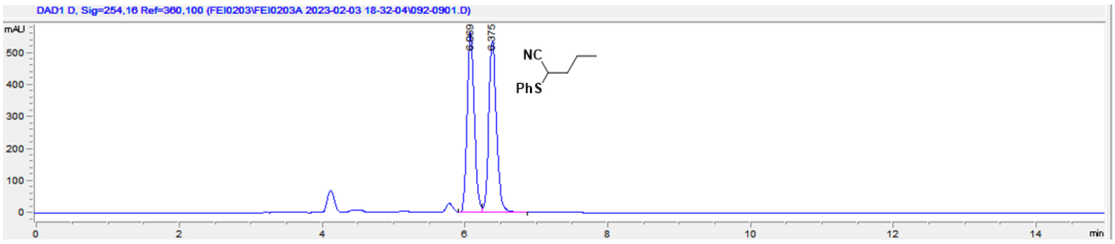

#	Time	Area	Height	Width	Area%	Symmetry
1	6.069	3912.2	560.9	0.1094	49.190	0.805
2	6.375	4041.1	535.3	0.1161	50.810	0.781

Obtained with ENE-101 from (*Z*)-**5al**/(*E*)-**5al** = 1/1

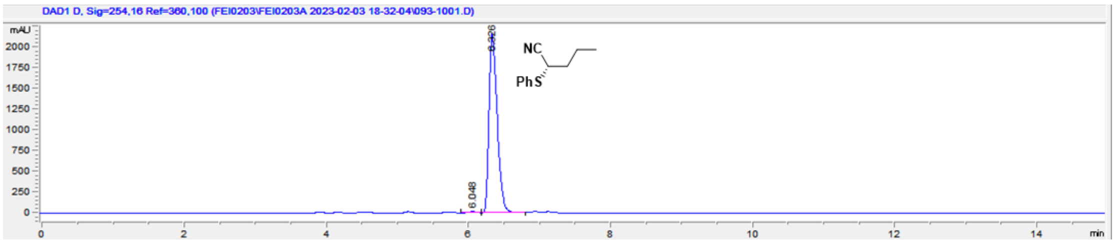

#	Time	Area	Height	Width	Area%	Symmetry
1	6.048	146.9	17.4	0.1366	0.832	0.981
2	6.326	17518.5	2162.9	0.1245	99.168	0.574

HPLC analysis of **7am**: Chiralcel® OJ-H column (4.6 mm × 250 mm, 5 μm); detected at 254 nm; heptane/EtOH = 98/2; flow rate: 1.0 mL/min.

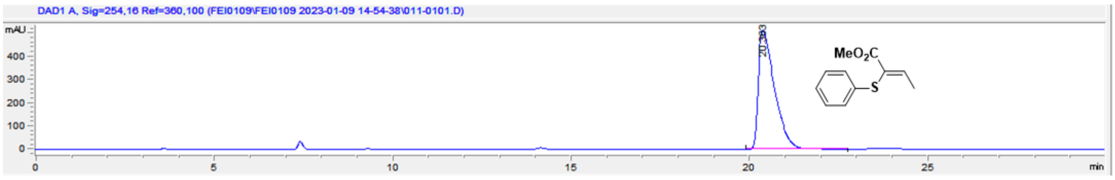

#	Time	Area	Height	Width	Area%	Symmetry
1	20.363	16358.4	512.3	0.4906	100.000	0.365

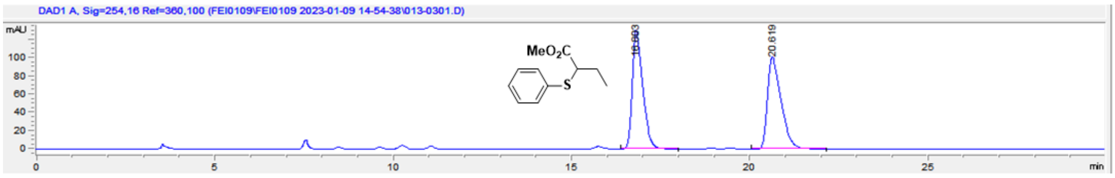

#	Time	Area	Height	Width	Area%	Symmetry
1	16.803	2675.5	130	0.3161	50.166	0.583
2	20.619	2657.8	101.1	0.4007	49.834	0.496

Obtained with ENE-101 from (*Z*)-**5am**

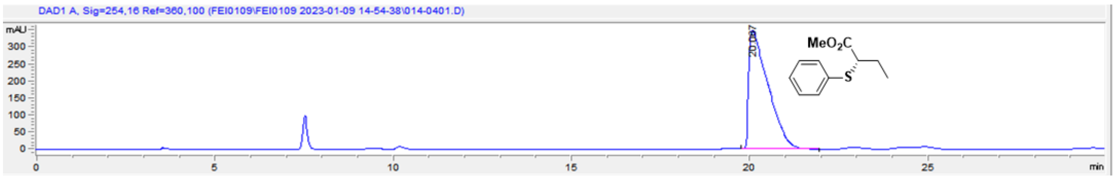

#	Time	Area	Height	Width	Area%	Symmetry
1	20.067	12959.3	347.5	0.5331	100.000	0.215

Note: In this case, the full conversion of the substrate to the desired product was also confirmed by ^{1}H NMR analysis of the crude reaction mixture.

HPLC analysis of **8aa**: Chiralpak® IG column (4.6 mm × 250 mm, 5 μm); detected at 230 nm; heptane/EtOH = 90/10; flow rate: 1.0 mL/min.

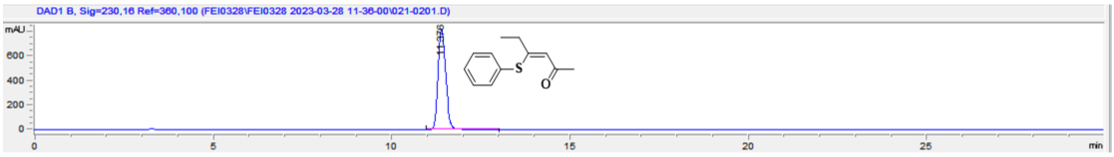

#	Time	Area	Height	Width	Area%	Symmetry
1	11.376	12015.2	818	0.2274	100.000	0.711

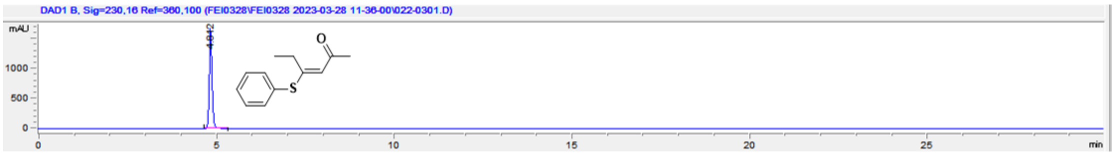

#	Time	Area	Height	Width	Area%	Symmetry
1	4.812	10798.7	1671.1	0.1014	100.000	0.831

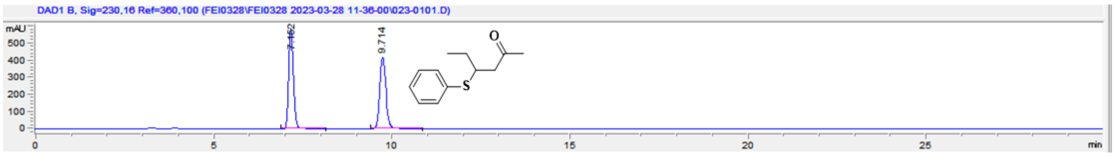

#	Time	Area	Height	Width	Area%	Symmetry
1	7.152	5146.6	586.4	0.1364	50.313	0.795
2	9.714	5082.6	418.1	0.1878	49.687	0.736

Obtained with ENE-101 from (*Z*)-**6aa**

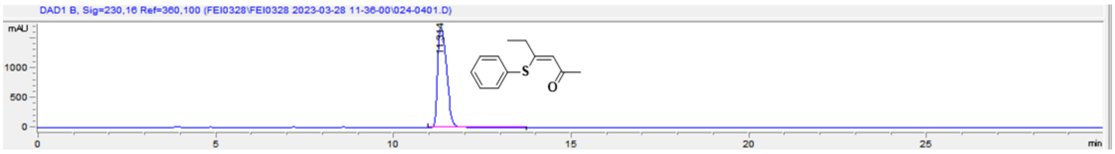

#	Time	Area	Height	Width	Area%	Symmetry
1	11.314	28993.2	1666.9	0.2789	100.000	0.489

Obtained with ENE-101 from (*E*)-**6aa**

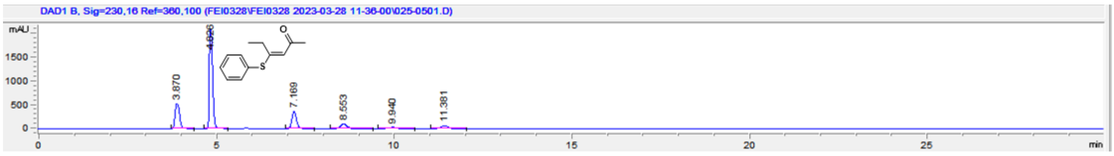

#	Time	Area	Height	Width	Area%	Symmetry
1	3.87	4453.3	527.9	0.1404	18.232	0.679
2	4.826	14231.7	2095	0.1073	58.265	0.782
3	7.169	3131.5	366.2	0.1338	12.820	0.826
4	8.553	1264.3	103.3	0.1868	5.176	0.913
5	9.94	454.7	30	0.2208	1.862	1.28
6	11.381	890.4	64.2	0.2138	3.645	0.895

HPLC analysis of **8ab**: Chiralpak® IG column (4.6 mm × 250 mm, 5 μm); detected at 254 nm; heptane/EtOH = 90/10; flow rate: 1.0 mL/min.

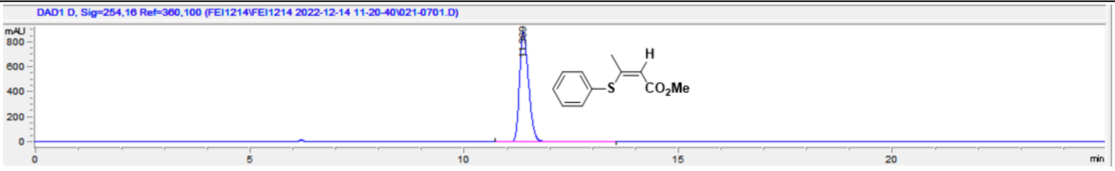

#	Time	Area	Height	Width	Area%	Symmetry
1	11.369	13065.4	885	0.2282	100.000	0.651

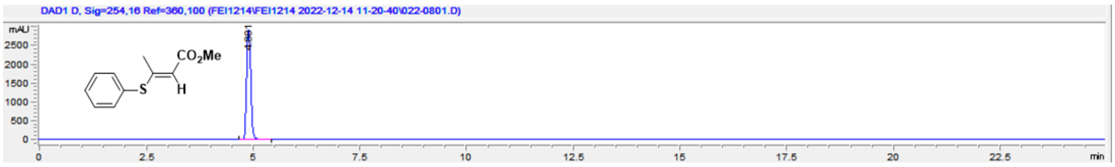

#	Time	Area	Height	Width	Area%	Symmetry
1	4.881	20240.1	2931.2	0.1106	100.000	0.773

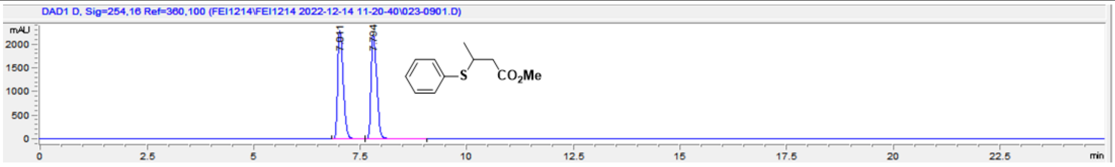

#	Time	Area	Height	Width	Area%	Symmetry
1	7.011	20598.8	2302.1	0.1384	49.948	0.587
2	7.794	20641.7	2201.6	0.1554	50.052	0.558

Obtained with ENE-101 from (*Z*)-**6ab**

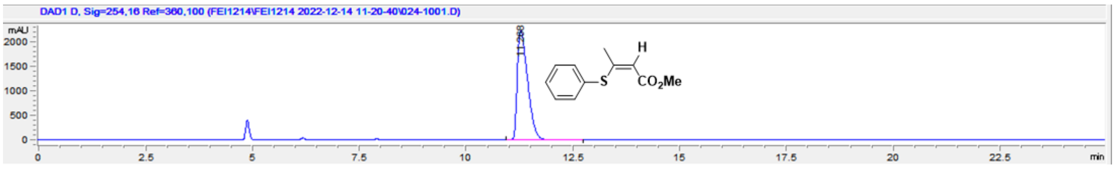

#	Time	Area	Height	Width	Area%	Symmetry
1	11.268	37750.5	2231.6	0.2614	100.000	0.437

Obtained with ENE-101 from (*E*)-**6ab**

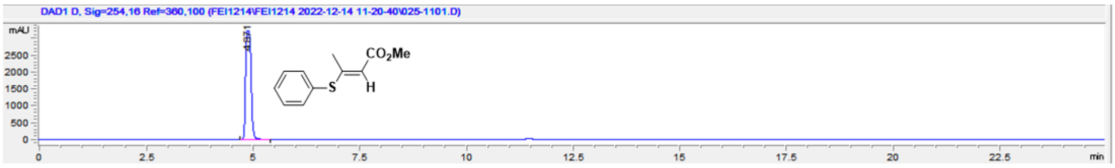

#	Time	Area	Height	Width	Area%	Symmetry
1	4.871	28573.8	3239.3	0.1429	100.000	0.72

HPLC Analysis for One-Step One-pot Hydrogen-Borrowing Cascade for the Enantioselective Synthesis of (*S*)-7 from 9

HPLC analysis of **7aa**: Chiralpak® IG column (4.6 mm × 250 mm, 5 µm); detected at 254 nm; heptane/EtOH = 95/5; flow rate: 0.8 mL/min.

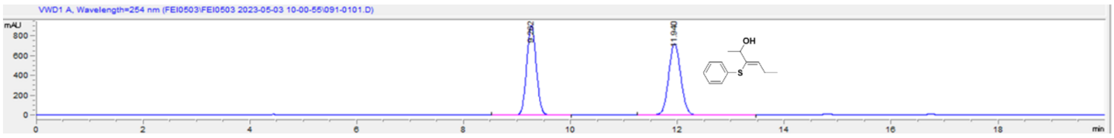

#	Time	Area	Height	Width	Area%	Symmetry
1	9.261	11375.6	908.6	0.1956	49.821	0.915
2	11.94	11457.3	722.7	0.2461	50.179	0.91

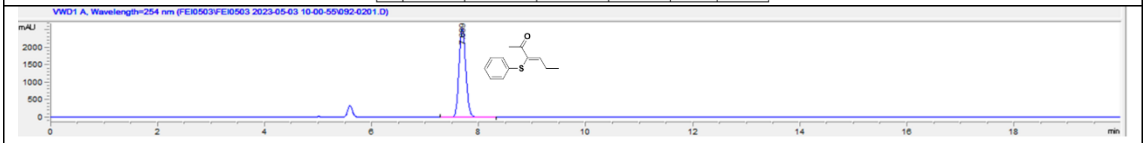

#	Time	Area	Height	Width	Area%	Symmetry
1	7.688	22726.8	2569.1	0.1411	100.000	0.87

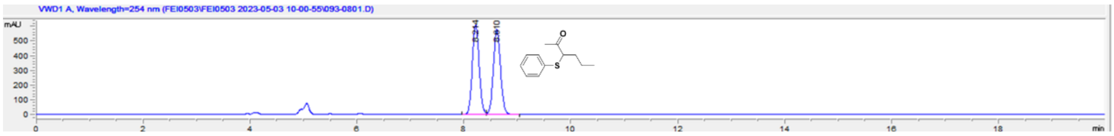

#	Time	Area	Height	Width	Area%	Symmetry
1	8.214	5477.8	612.4	0.1422	50.141	0.897
2	8.61	5447.1	580.9	0.1473	49.859	0.877

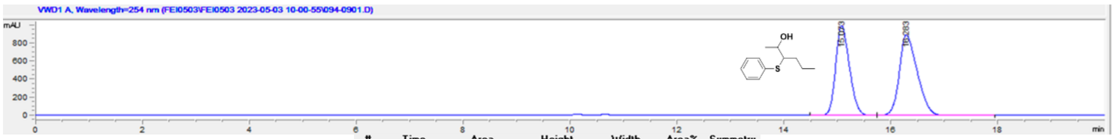

#	Time	Area	Height	Width	Area%	Symmetry
1	15.073	17759.8	995.2	0.2781	43.742	0.739
2	16.283	20709.5	891.2	0.3526	51.007	0.626

Obtained from the hydrogen-borrowing cascade in 1.5 h

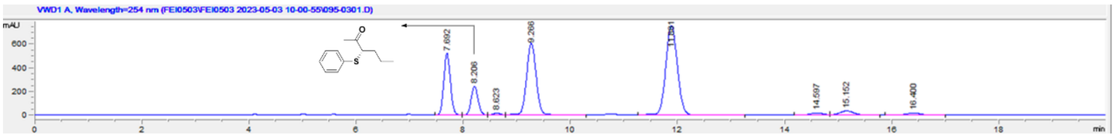

#	Time	Area	Height	Width	Area%	Symmetry
1	7.692	4486	524.1	0.1348	16.535	0.903
2	8.206	2170.8	242.1	0.1424	8.001	0.928
3	8.622	142.4	16.1	0.1408	0.525	0.946
4	9.266	7534.8	608.8	0.1939	27.773	0.907
5	11.88	11447	759.6	0.237	42.193	0.914
6	14.597	327.5	18.8	0.2734	1.207	0.978
7	15.152	670.4	35.7	0.2921	2.471	0.965
8	16.4	351.1	17.6	0.3119	1.294	0.937

Obtained from the hydrogen-borrowing cascade in 12 h

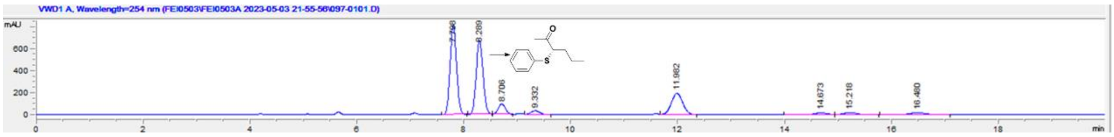

#	Time	Area	Height	Width	Area%	Symmetry
1	7.798	6859.6	815.7	0.1301	37.594	0.92
2	8.288	6013	673.4	0.1391	32.954	0.882
3	8.705	898.2	95.4	0.1507	4.923	0.954
4	9.332	421.3	35.9	0.1863	2.309	0.942
5	11.982	2943.1	195.2	0.2341	16.130	0.953
6	14.673	319.6	18.6	0.268	1.752	0.992
7	15.218	369.8	20.2	0.2859	2.027	0.951
8	16.48	422.1	19.4	0.3348	2.313	0.908

HPLC analysis of **7ae**: Chiralpak® IG column (4.6 mm × 250 mm, 5 μm); detected at 254 nm; heptane/EtOH = 95/5; flow rate: 0.8 mL/min.

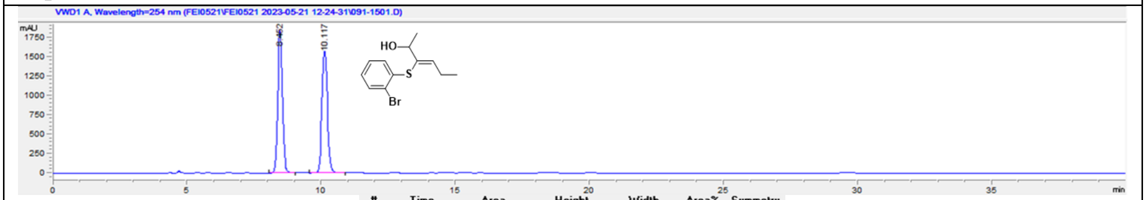

#	Time	Area	Height	Width	Area%	Symmetry
1	8.452	22510.6	1833.6	0.1927	50.139	0.914
2	10.117	22386	1566.3	0.225	49.861	0.876

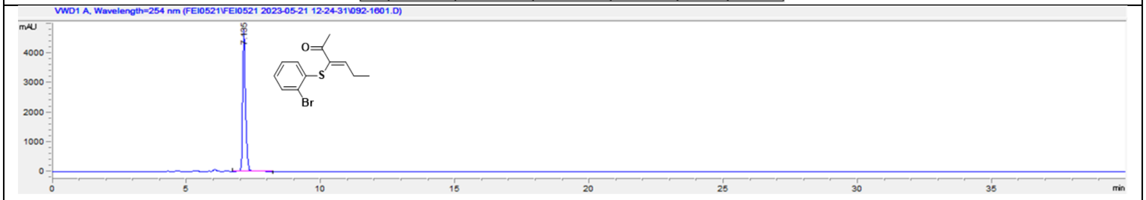

#	Time	Area	Height	Width	Area%	Symmetry
1	7.135	33336.7	4800.9	0.097	100.000	0.821

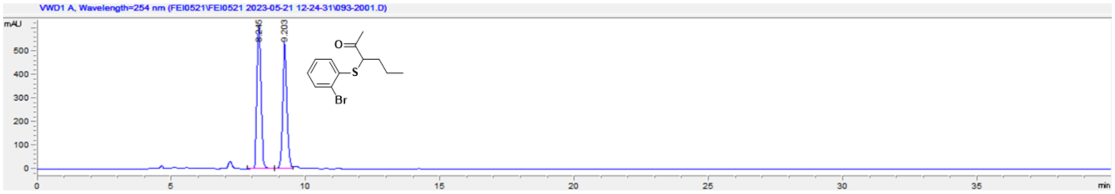

#	Time	Area	Height	Width	Area%	Symmetry
1	8.245	6042.7	605.6	0.1557	50.131	0.846
2	9.203	6011.1	539.3	0.1708	49.869	0.837

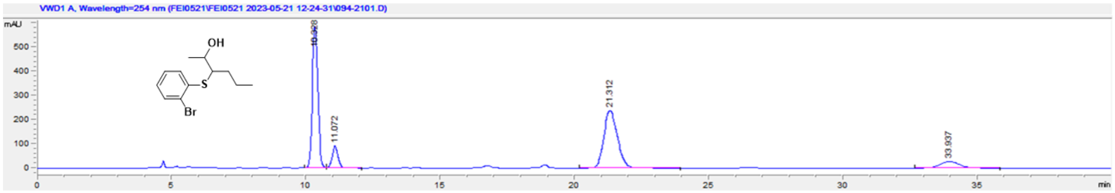

#	Time	Area	Height	Width	Area%	Symmetry
1	10.328	8321	584.2	0.2215	43.014	0.882
2	11.072	1370.1	91.6	0.2328	7.083	0.908
3	21.312	8337.1	238.7	0.5444	43.097	0.778
4	33.937	1316.5	26.9	0.7629	6.806	0.897

Obtained from the hydrogen-borrowing cascade in 12 h

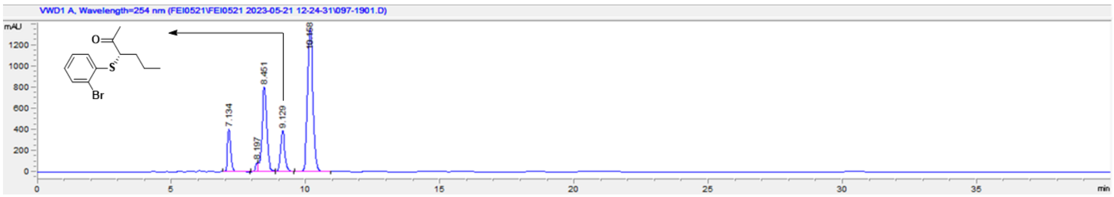

#	Time	Area	Height	Width	Area%	Symmetry
1	7.134	3331.8	402.1	0.1287	8.941	0.846
2	8.197	608.6	83.2	0.1115	1.633	2.259
3	8.451	9926.3	797.1	0.1919	26.639	0.956
4	9.129	4221.2	383.6	0.1721	11.328	0.819
5	10.158	19174.7	1347	0.2214	51.458	0.881

HPLC Analysis for Two-Step One-Pot ENE-101/NaBH$_4$ Cascade, Two-Step One-Pot ENE-101/ADH-153 Cascade, and One-Step One-Pot ENE-101/ADH-153 Cascade

HPLC analysis of **10aa**: Chiralpak® IG column (4.6 mm × 250 mm, 5 μm); detected at 254 nm; heptane/EtOH = 95/5; flow rate: 0.8 mL/min.

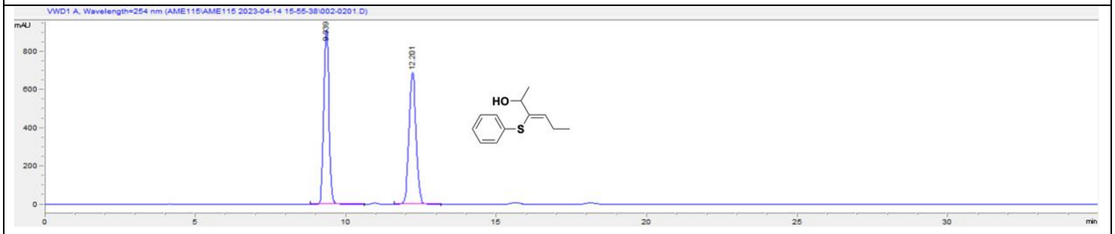

#	Time	Area	Height	Width	Area%	Symmetry
1	9.339	11005	912.2	0.1888	49.429	0.909
2	12.201	11259	689.5	0.2546	50.571	0.92

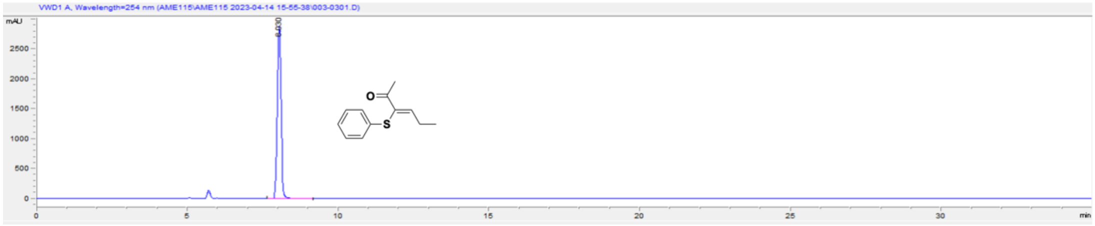

#	Time	Area	Height	Width	Area%	Symmetry
1	8.03	23945.9	2886.4	0.1288	100.000	0.881

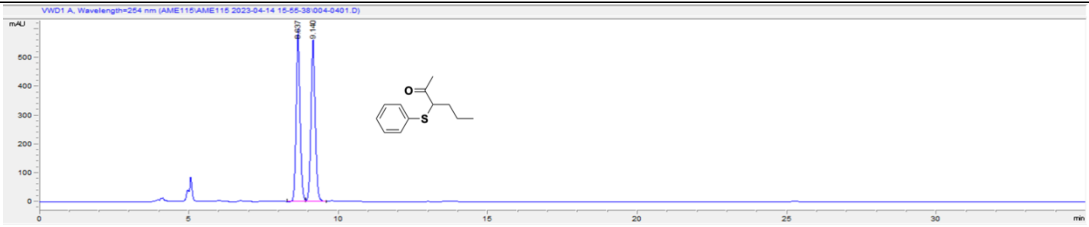

#	Time	Area	Height	Width	Area%	Symmetry
1	8.637	5682.8	600.3	0.1468	49.597	0.874
2	9.14	5775	563.2	0.1589	50.403	0.866

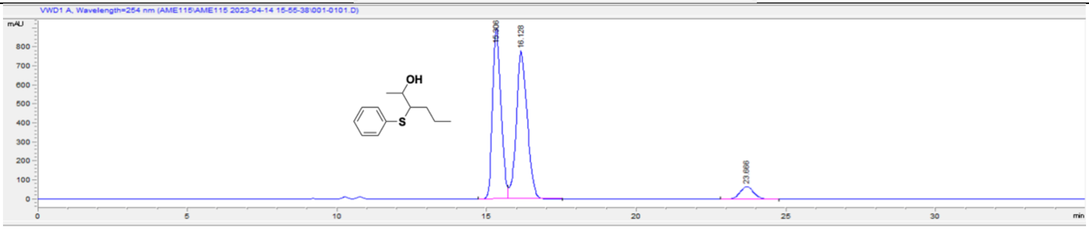

#	Time	Area	Height	Width	Area%	Symmetry
1	15.306	17742.5	896.7	0.3095	45.008	0.745
2	16.128	19645.9	778.4	0.3818	49.837	0.721
3	23.666	2032	65.3	0.4855	5.155	0.908

Obtained from two-step one-pot ENE-101/NaBH$_4$ cascade

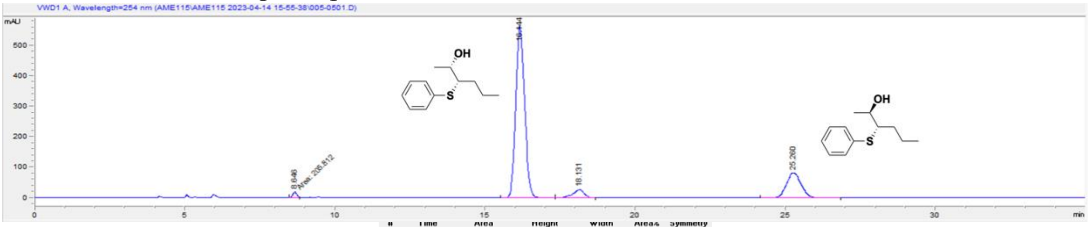

#	Time	Area	Height	Width	Area%	Symmetry
1	8.646	205.8	20.4	0.1685	1.358	0.965
2	16.144	11532.2	562.8	0.3206	76.081	0.813
3	18.131	691.6	26.5	0.3828	4.562	1.434
4	25.26	2728.1	81.3	0.5255	17.998	0.887

Obtained from two-step one-pot ENE-101/ADH-153 cascade

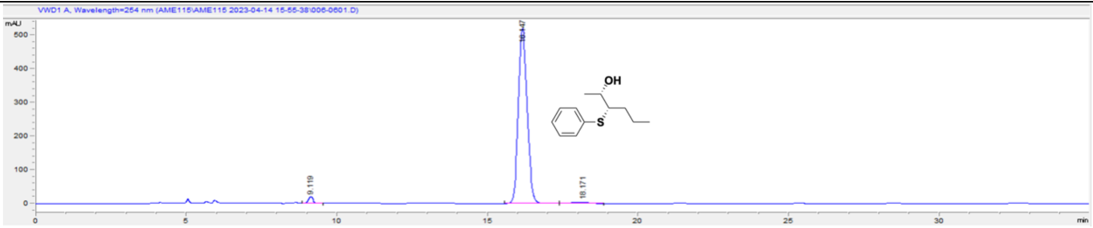

#	Time	Area	Height	Width	Area%	Symmetry
1	9.119	207.7	19.6	0.1642	1.895	0.91
2	16.147	10624.6	519.1	0.3174	96.922	0.815
3	18.171	129.6	3.4	0.5208	1.183	2.154

Obtained from one-step one-pot ENE-101/ADH-153 cascade

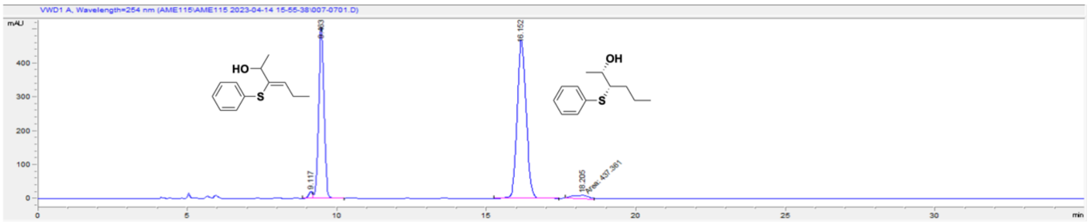

#	Time	Area	Height	Width	Area%	Symmetry
1	9.117	188.3	19.6	0.1482	1.123	1.149
2	9.463	6379.2	503.2	0.1989	38.039	0.926
3	16.152	9765.4	469	0.3245	58.231	0.844
4	18.205	437.4	11.1	0.6565	2.608	1.706

HPLC analysis of **10ab**: Chiralpak® IG column (4.6 mm × 250 mm, 5 µm); detected at 254 nm; heptane/EtOH = 95/5; flow rate: 0.8 mL/min.

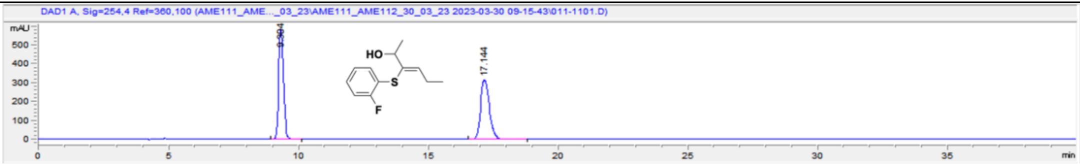

#	Time	Area	Height	Width	Area%	Symmetry
1	9.304	7544.5	585.3	0.2024	50.653	0.858
2	17.144	7349.9	316.4	0.359	49.347	0.73

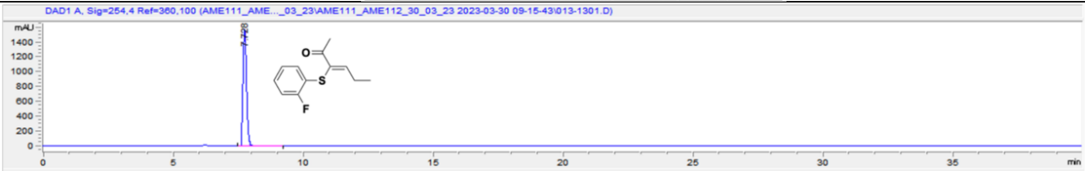

#	Time	Area	Height	Width	Area%	Symmetry
1	7.728	13707.2	1583.4	0.133	100.000	0.797

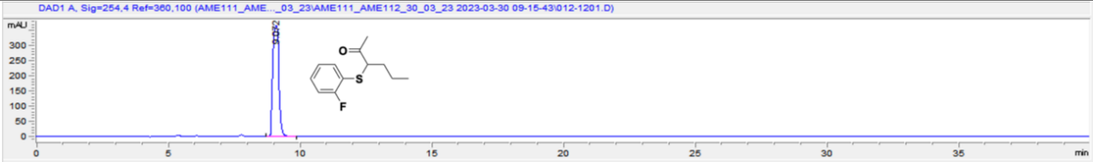

#	Time	Area	Height	Width	Area%	Symmetry
1	9.072	5847.6	366.5	0.2581	100.000	1.058

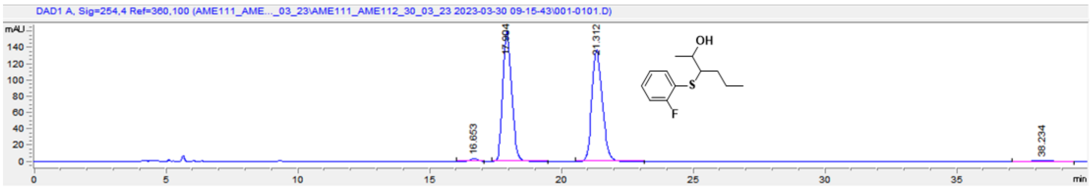

#	Time	Area	Height	Width	Area%	Symmetry
1	16.653	76.3	3.7	0.3139	0.985	1.064
2	17.904	3760.3	160.6	0.3651	48.591	0.768
3	21.312	3821.6	137.1	0.4332	49.383	0.771
4	38.234	80.6	1.6	0.6154	1.041	1.099

Obtained from two-step one-pot ENE-101/ADH-153 cascade

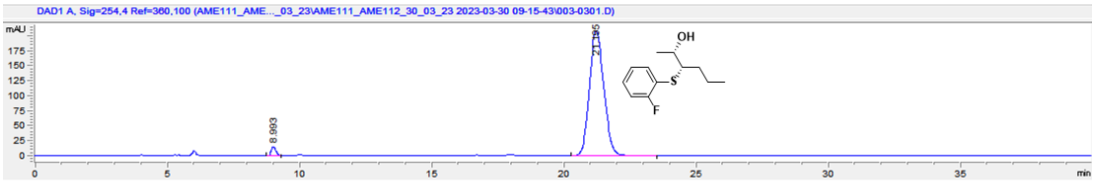

#	Time	Area	Height	Width	Area%	Symmetry
1	8.993	175.8	15.2	0.1751	2.170	0.763
2	21.195	7926.9	209.4	0.5976	97.830	0.808

Obtained from one-step one-pot ENE-101/ADH-153 cascade

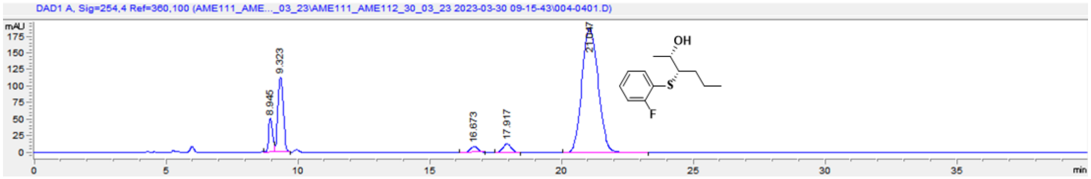

#	Time	Area	Height	Width	Area%	Symmetry
1	8.945	544.6	51.1	0.1642	5.126	0.891
2	9.323	1546.4	113.4	0.2152	14.556	0.89
3	16.673	184.2	9.1	0.3177	1.734	0.991
4	17.917	286	13.2	0.3404	2.692	0.925
5	21.047	8062.6	188.4	0.6792	75.891	0.844

HPLC analysis of **10ac**: Chiralpak® IG column (4.6 mm × 250 mm, 5 μm); detected at 254 nm; heptane/EtOH = 95/5; flow rate: 0.8 mL/min.

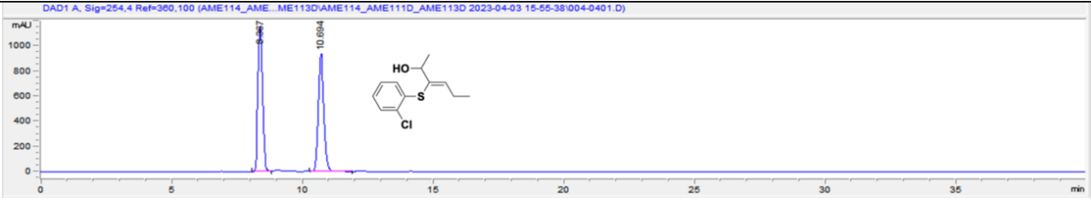

#	Time	Area	Height	Width	Area%	Symmetry
1	8.367	13495.5	1142.8	0.1838	49.384	0.904
2	10.694	13831.9	935.5	0.2305	50.616	0.825

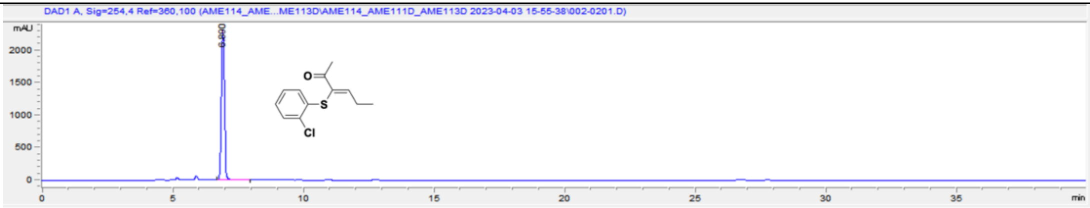

#	Time	Area	Height	Width	Area%	Symmetry
1	6.89	18076.2	2298.7	0.1237	100.000	0.8

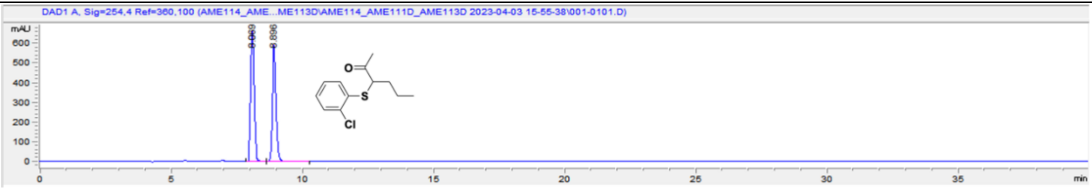

#	Time	Area	Height	Width	Area%	Symmetry
1	8.069	5938.4	665.4	0.1381	50.192	0.826
2	8.896	5893.1	594.5	0.1536	49.808	0.79

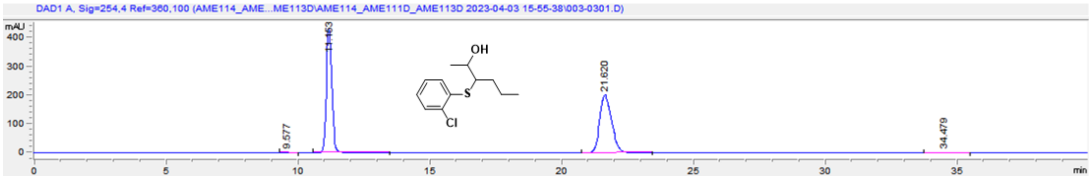

#	Time	Area	Height	Width	Area%	Symmetry
1	9.577	7.2	4.7E-1	0.2382	0.057	0.796
2	11.153	6356.8	433.3	0.2272	50.169	0.863
3	21.62	6276.3	200.6	0.489	49.534	0.734
4	34.479	30.4	7E-1	0.5469	0.240	0.749

Obtained from two-step one-pot ENE-101/ADH-153 cascade

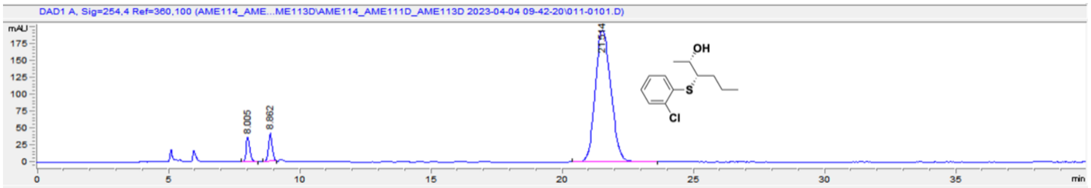

#	Time	Area	Height	Width	Area%	Symmetry
1	8.005	364.4	36.4	0.1568	4.109	0.865
2	8.862	441.1	41.9	0.1648	4.974	0.916
3	21.514	8063	194.5	0.6557	90.917	0.801

Obtained from one-step one-pot ENE-101/ADH-153 cascade

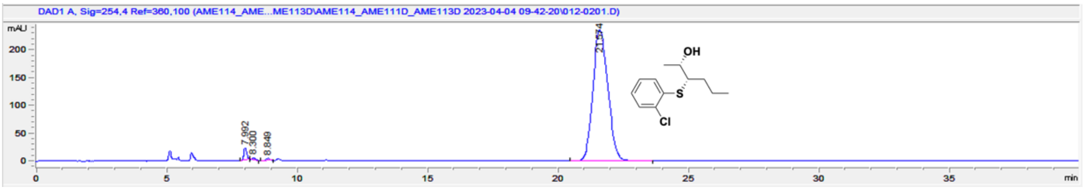

#	Time	Area	Height	Width	Area%	Symmetry
1	7.992	195.9	21.9	0.1424	2.013	0.984
2	8.3	37.1	3.8	0.1551	0.381	0.694
3	8.849	51	4.8	0.167	0.524	0.936
4	21.574	9443.3	235.5	0.6358	97.081	0.791

HPLC analysis of **10ad**: Chiralpak® IG column (4.6 mm × 250 mm, 5 μm); detected at 254 nm; heptane/EtOH = 95/5; flow rate: 0.8 mL/min.

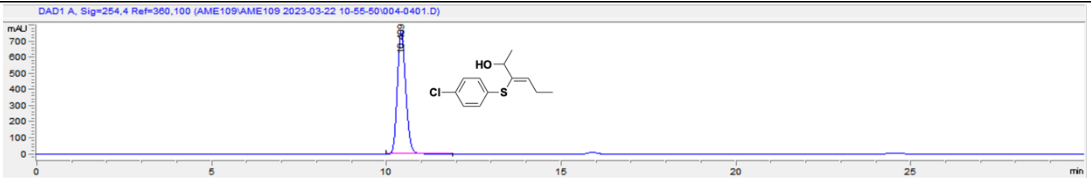

#	Time	Area	Height	Width	Area%	Symmetry
1	10.409	13379.2	770.4	0.2746	100.000	0.801

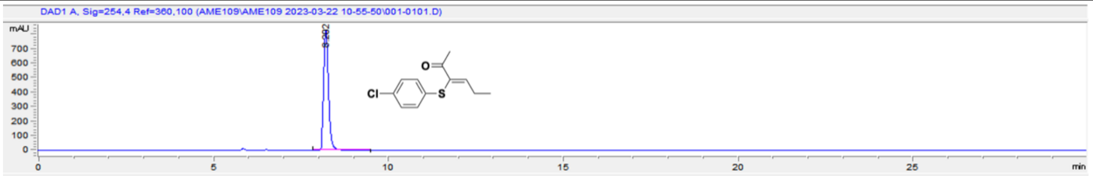

#	Time	Area	Height	Width	Area%	Symmetry
1	8.202	7773.5	830	0.1433	100.000	0.807

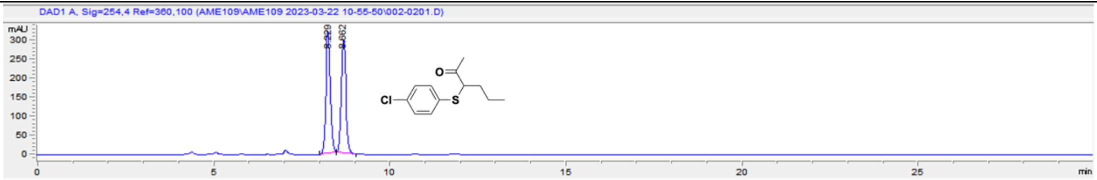

#	Time	Area	Height	Width	Area%	Symmetry
1	8.229	3007.1	323.1	0.1446	51.612	0.893
2	8.662	2819.3	296.8	0.1468	48.388	0.831

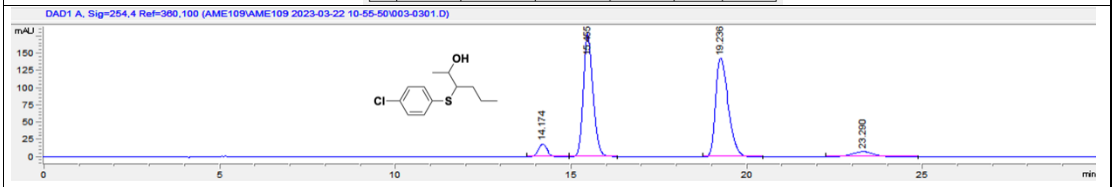

#	Time	Area	Height	Width	Area%	Symmetry
1	14.174	316.1	18.4	0.2668	4.110	0.916
2	15.455	3518.5	180.5	0.3034	45.747	0.775
3	19.236	3539	143.1	0.3844	46.014	0.576
4	23.29	317.6	7.5	0.6501	4.129	1.038

Obtained from two-step one-pot ENE-101/NaBH$_4$ cascade

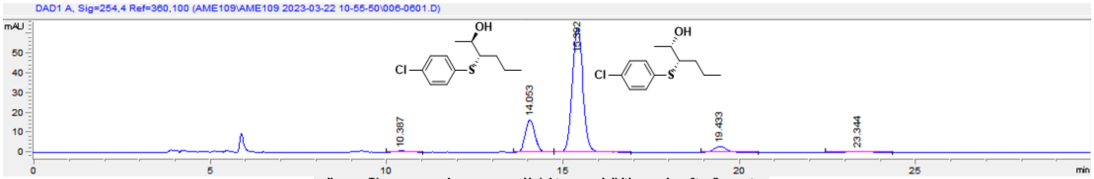

#	Time	Area	Height	Width	Area%	Symmetry
1	10.387	16.1	6.6E-1	0.3284	0.933	0.653
2	14.053	297.7	16.1	0.2872	17.280	0.949
3	15.392	1314.6	62.9	0.3238	76.312	0.87
4	19.433	74.1	3	0.3857	4.300	0.926
5	23.344	20.3	5.5E-1	0.5221	1.176	0.868

Obtained from two-step one-pot ENE-101/ADH-153 cascade

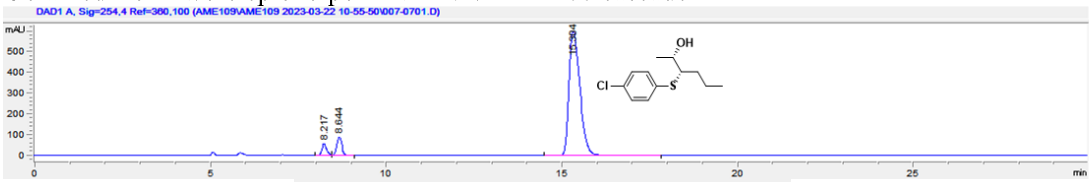

#	Time	Area	Height	Width	Area%	Symmetry
1	8.217	595.2	56.7	0.1584	4.163	0.815
2	8.644	920.9	88.9	0.1609	6.440	0.873
3	15.304	12782.7	601.6	0.3279	89.397	0.557

Obtained from one-step one-pot ENE-101/ADH-153 cascade

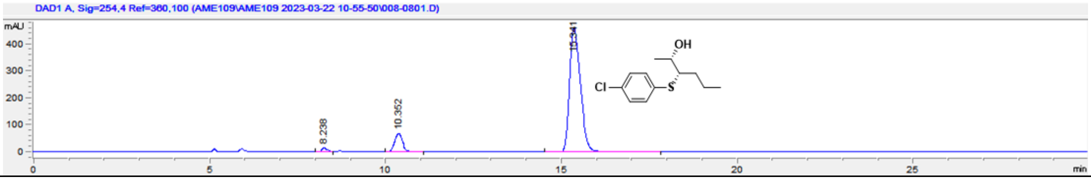

#	Time	Area	Height	Width	Area%	Symmetry
1	8.238	186.5	16.2	0.1745	1.705	0.705
2	10.352	1078.2	68.6	0.2474	9.861	0.873
3	15.341	9669.4	461.6	0.3244	88.434	0.616

HPLC analysis of **10ae**: Chiralpak® IG column (4.6 mm × 250 mm, 5 μm); detected at 254 nm; heptane/EtOH = 95/5; flow rate: 0.8 mL/min.

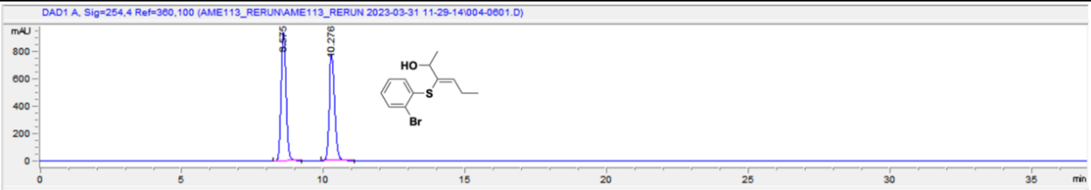

#	Time	Area	Height	Width	Area%	Symmetry
1	8.575	11074.4	943.8	0.1829	50.054	0.881
2	10.276	11050.7	781.2	0.219	49.946	0.847

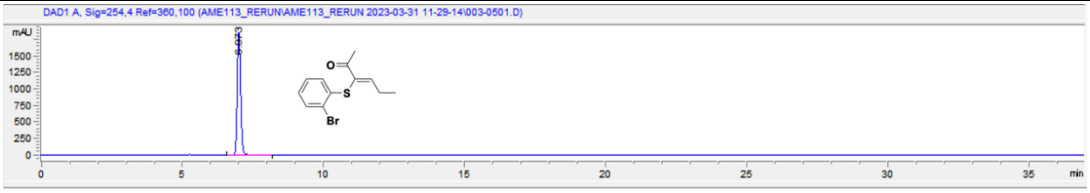

#	Time	Area	Height	Width	Area%	Symmetry
1	6.973	14977.9	1861.1	0.1239	100.000	0.82

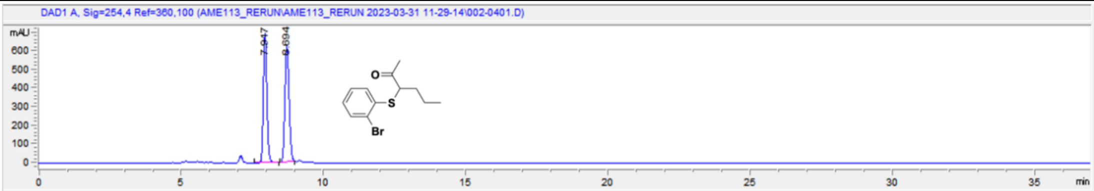

#	Time	Area	Height	Width	Area%	Symmetry
1	7.917	5733.1	693.5	0.1285	47.880	0.803
2	8.694	6240.8	621.6	0.1551	52.120	0.827

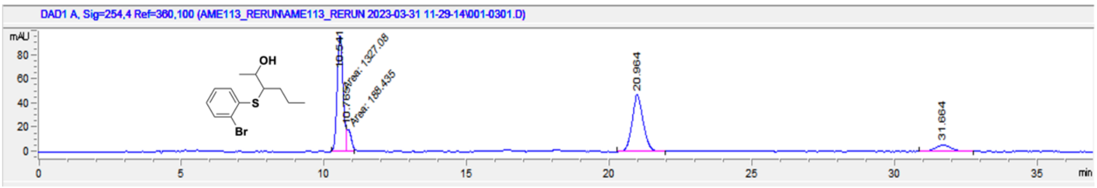

#	Time	Area	Height	Width	Area%	Symmetry
1	10.541	1327.1	95.3	0.232	43.782	0.877
2	10.765	188.4	17.8	0.1765	6.217	0
3	20.964	1306.1	47	0.4244	43.090	0.878
4	31.664	209.5	5.3	0.544	6.911	0.896

Obtained from two-step one-pot ENE-101/NaBH$_4$ cascade

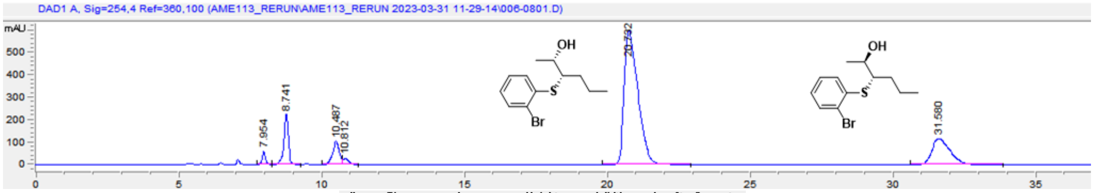

#	Time	Area	Height	Width	Area%	Symmetry
1	7.954	520.8	59.4	0.1364	1.734	0.908
2	8.741	2352.8	222.9	0.1572	7.834	1.085
3	10.487	1696.9	103.6	0.2451	5.650	1.114
4	10.812	353.7	27.4	0.1948	1.178	0.665
5	20.732	20059.4	602.2	0.4906	66.791	0.486
6	31.58	5049.3	115.4	0.6824	16.813	0.589

Obtained from two-step one-pot ENE-101/ADH-153 cascade

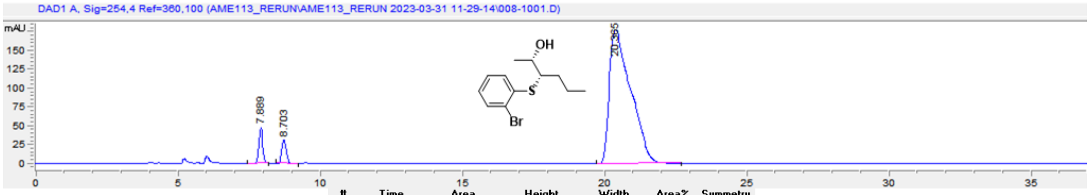

#	Time	Area	Height	Width	Area%	Symmetry
1	7.889	465.1	47.4	0.1525	4.612	0.887
2	8.703	327.9	30.9	0.1639	3.252	0.86
3	20.365	9291.3	178	0.7369	92.136	0.382

Obtained from one-step one-pot ENE-101/ADH-153 cascade

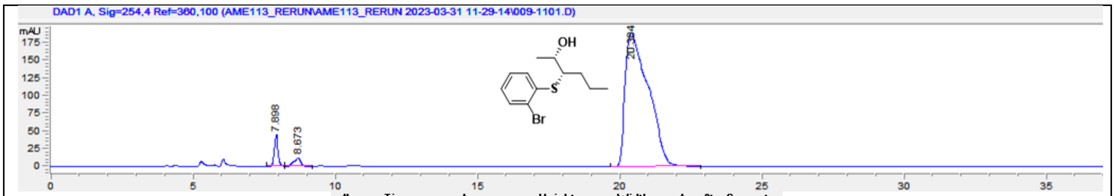

#	Time	Area	Height	Width	Area%	Symmetry
1	7.898	419.7	44.2	0.1468	3.728	0.919
2	8.673	189	11.6	0.2273	1.679	1.682
3	20.384	10649.3	189.2	0.7948	94.593	0.334

HPLC Analysis for Two-step One-pot ENE-101/ADH-19 Cascade

HPLC analysis of **10aa**: Chiralpak® IG column (4.6 mm × 250 mm, 5 µm); detected at 254 nm; heptane/EtOH = 95/5; flow rate: 0.8 mL/min.

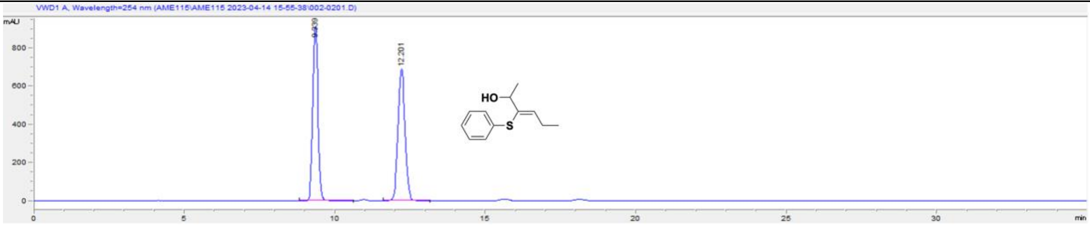

#	Time	Area	Height	Width	Area%	Symmetry
1	9.339	11005	912.2	0.1888	49.429	0.909
2	12.201	11259	689.5	0.2546	50.571	0.92

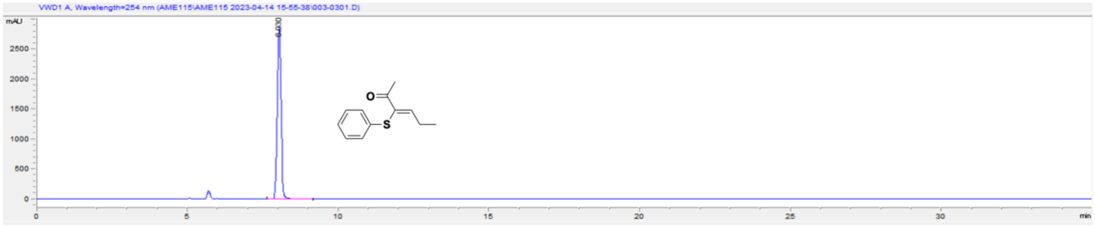

#	Time	Area	Height	Width	Area%	Symmetry
1	8.03	23945.9	2886.4	0.1288	100.000	0.881

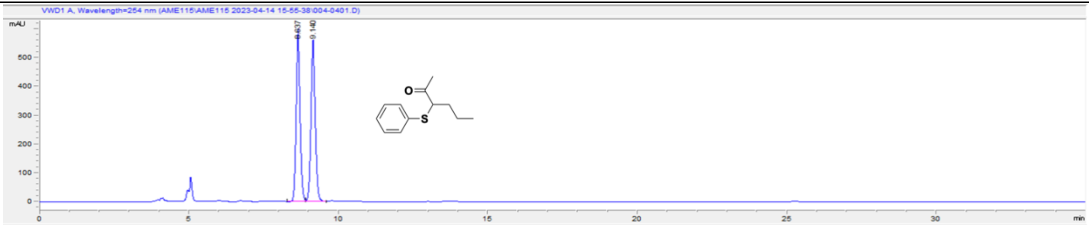

#	Time	Area	Height	Width	Area%	Symmetry
1	8.637	5682.8	600.3	0.1468	49.597	0.874
2	9.14	5775	563.2	0.1589	50.403	0.866

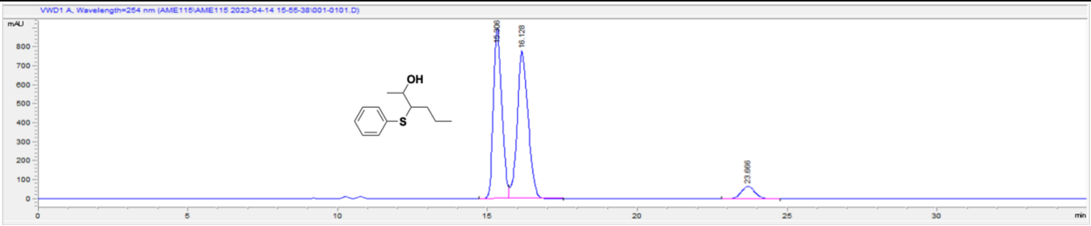

#	Time	Area	Height	Width	Area%	Symmetry
1	15.306	17742.5	896.7	0.3095	45.008	0.745
2	16.128	19645.9	778.4	0.3818	49.837	0.721
3	23.666	2032	65.3	0.4855	5.155	0.908

Obtained from two-step one-pot ENE-101/ADH-19 cascade

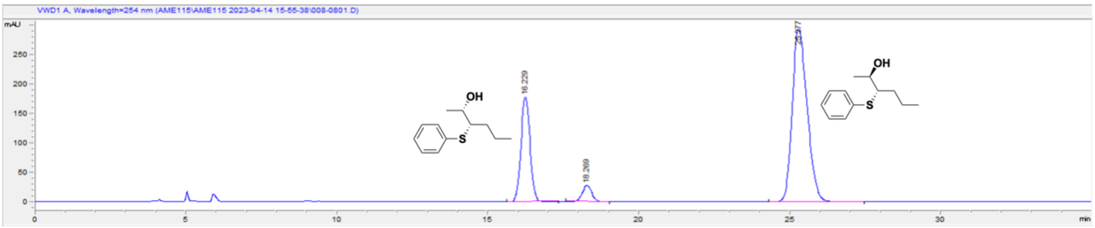

#	Time	Area	Height	Width	Area%	Symmetry
1	16.229	3684.2	177.2	0.3241	25.693	0.918
2	18.269	597.3	27.4	0.342	4.165	0.983
3	25.277	10058	292.8	0.5347	70.142	0.701

HPLC analysis of **10ab**: Chiralpak® IG column (4.6 mm × 250 mm, 5 μm); detected at 254 nm; heptane/EtOH = 95/5; flow rate: 0.8 mL/min.

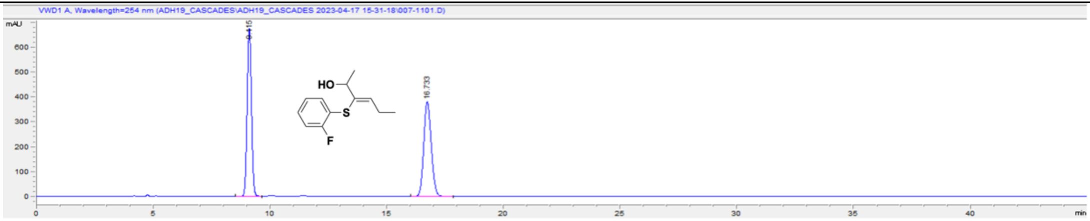

#	Time	Area	Height	Width	Area%	Symmetry
1	9.115	8607.3	677.1	0.1993	50.481	0.937
2	16.733	8443.3	381.6	0.3426	49.519	0.83

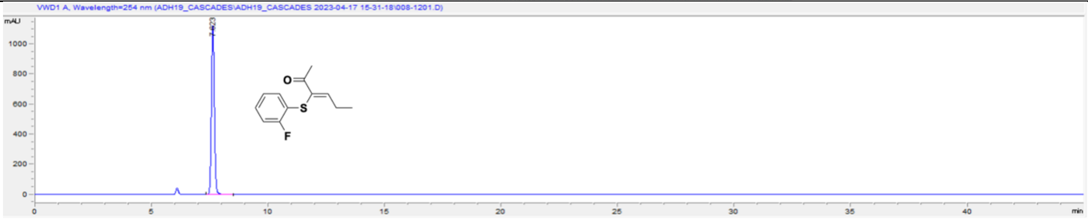

#	Time	Area	Height	Width	Area%	Symmetry
1	7.623	9766.1	1122.7	0.1364	100.000	0.878

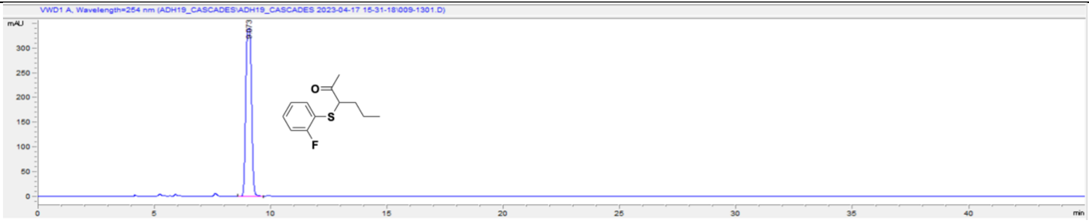

#	Time	Area	Height	Width	Area%	Symmetry
1	9.073	5724.5	340.9	0.2323	100.000	1.279

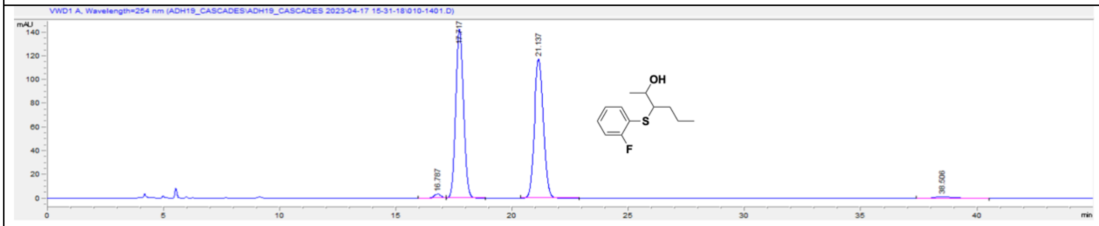

#	Time	Area	Height	Width	Area%	Symmetry
1	16.787	78	3.6	0.3355	1.163	1.103
2	17.717	3282.4	142.4	0.3592	48.935	0.877
3	21.137	3264	117.3	0.4353	48.660	0.902
4	38.506	83.3	1.4	0.7163	1.242	0.723

Obtained from two-step one-pot ENE-101/ADH-19 cascade

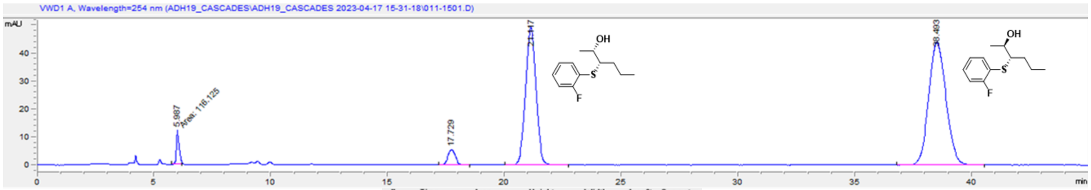

#	Time	Area	Height	Width	Area%	Symmetry
1	5.987	116.1	12.2	0.1589	2.872	0.617
2	17.729	120.2	5.5	0.3469	2.971	0.991
3	21.117	1545.6	49.6	0.4862	38.224	0.967
4	38.493	2261.7	44	0.7972	55.933	0.842

HPLC analysis of **10ac**: Chiralpak® IG column (4.6 mm × 250 mm, 5 μm); detected at 254 nm; heptane/EtOH = 95/5; flow rate: 0.8 mL/min.

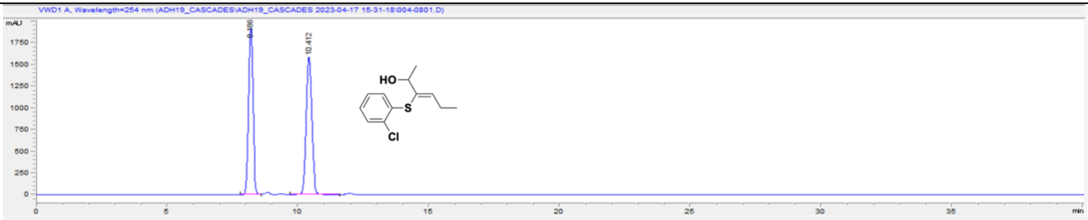

#	Time	Area	Height	Width	Area%	Symmetry
1	8.186	22716.2	1923.6	0.1858	49.413	0.932
2	10.412	23255.8	1581.4	0.2298	50.587	0.878

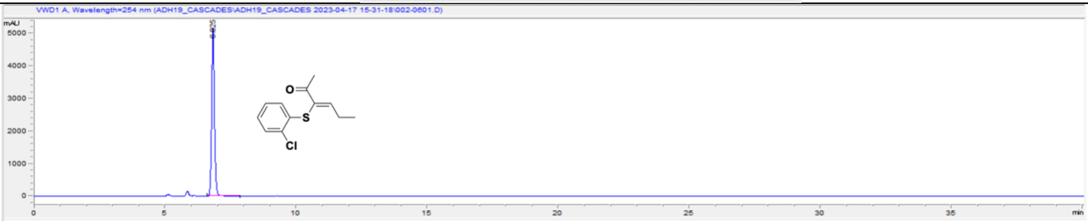

#	Time	Area	Height	Width	Area%	Symmetry
1	6.825	35277.6	5165.8	0.1001	100.000	0.868

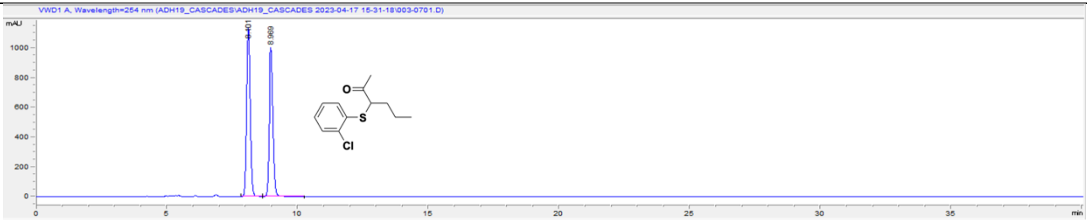

#	Time	Area	Height	Width	Area%	Symmetry
1	8.101	10320.4	1131.7	0.1413	50.154	0.868
2	8.969	10257.2	1001.5	0.1588	49.846	0.82

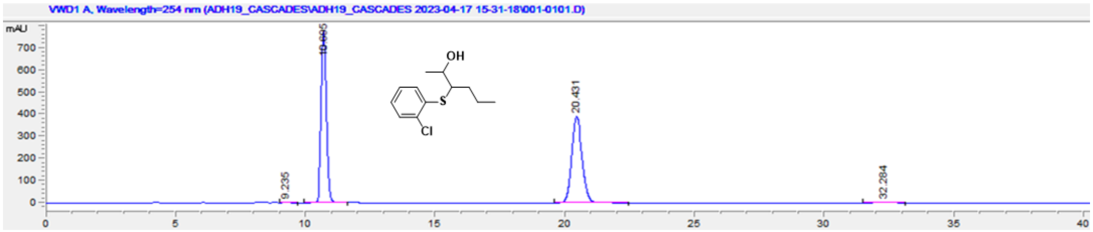

#	Time	Area	Height	Width	Area%	Symmetry
1	9.235	12.1	9.2E-1	0.1981	0.056	0.779
2	10.695	10804.7	771.7	0.2187	49.701	0.898
3	20.431	10876.7	388.8	0.4341	50.032	0.801
4	32.284	46	1.1	0.4918	0.212	0.888

Obtained from two-step one-pot ENE-101/ADH-19 cascade

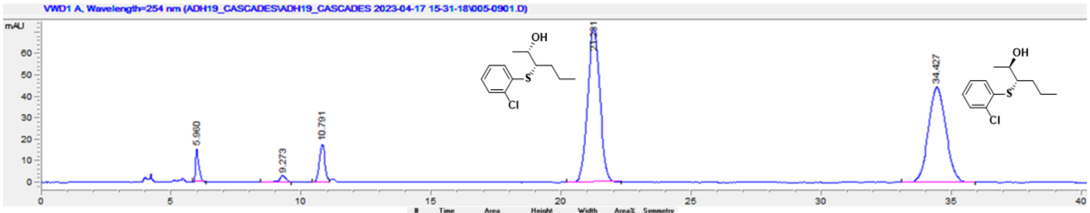

#	Time	Area	Height	Width	Area%	Symmetry
1	5.96	148.2	15.4	0.147	3.030	0.437
2	9.273	52.8	3	0.2636	1.080	1.23
3	10.791	264.7	17.6	0.2354	5.410	0.946
4	21.231	2346.1	71.5	0.5137	47.955	0.956
5	34.427	2080.4	44.6	0.7308	42.525	0.915

HPLC analysis of **10ad**: Chiralpak® IG column (4.6 mm × 250 mm, 5 µm); detected at 254 nm; heptane/EtOH = 95/5; flow rate: 0.8 mL/min.

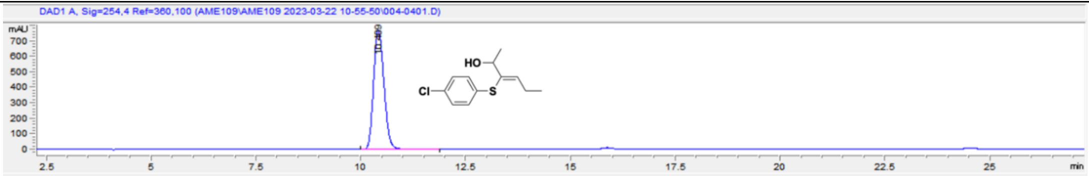

#	Time	Area	Height	Width	Area%	Symmetry
1	10.409	13379.2	770.4	0.2746	100.000	0.801

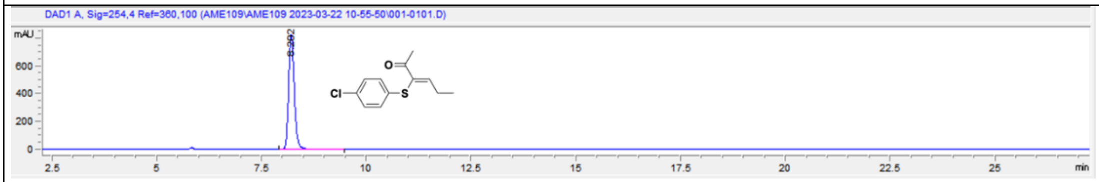

#	Time	Area	Height	Width	Area%	Symmetry
1	8.202	7773.5	830	0.1433	100.000	0.807

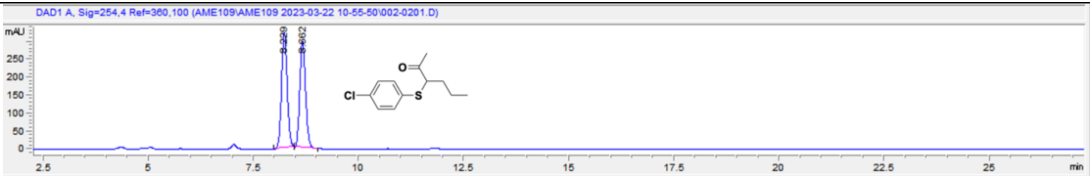

#	Time	Area	Height	Width	Area%	Symmetry
1	8.229	3007.1	323.1	0.1446	51.612	0.893
2	8.662	2819.3	296.8	0.1468	48.388	0.831

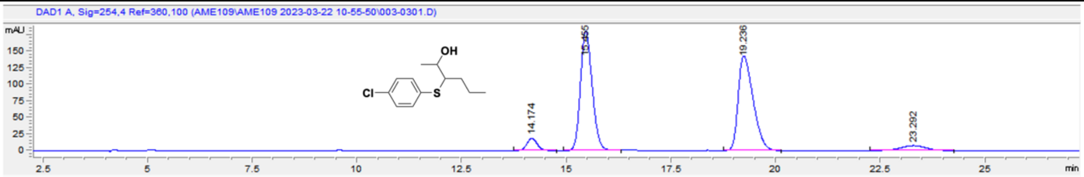

#	Time	Area	Height	Width	Area%	Symmetry
1	14.174	315.9	18.4	0.2667	4.112	0.917
2	15.455	3518.5	180.5	0.3014	45.804	0.773
3	19.236	3534.5	143.1	0.3841	46.013	0.575
4	23.292	312.7	7.5	0.5844	4.071	1.071

Obtained from two-step one-pot ENE-101/ADH-19 cascade

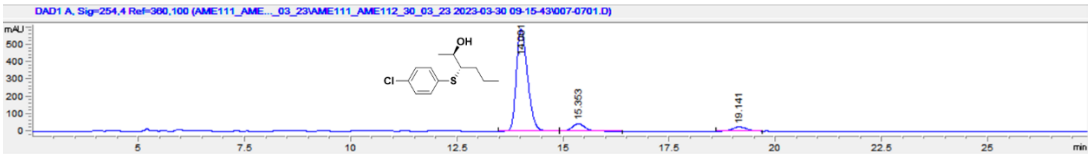

#	Time	Area	Height	Width	Area%	Symmetry
1	14.001	10686.6	587.2	0.2823	88.558	0.671
2	15.353	829.9	41	0.316	6.878	0.926
3	19.141	550.8	23.8	0.3659	4.565	0.89

HPLC analysis of **10ae**: Chiralpak® IG column (4.6 mm × 250 mm, 5 μm); detected at 254 nm; heptane/EtOH = 95/5; flow rate: 0.8 mL/min.

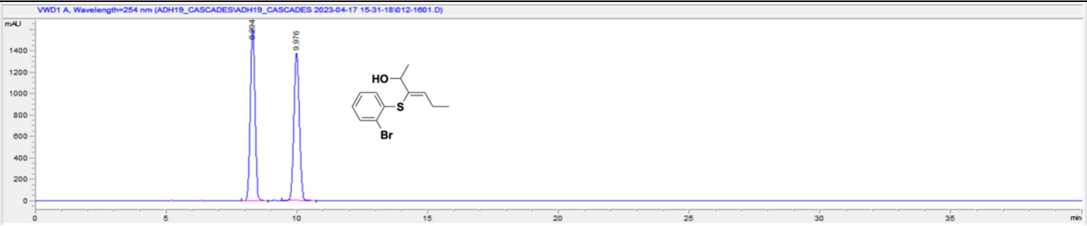

#	Time	Area	Height	Width	Area%	Symmetry
1	8.294	19460.9	1609.3	0.1891	50.330	0.935
2	9.976	19205.7	1380.6	0.2176	49.670	0.904

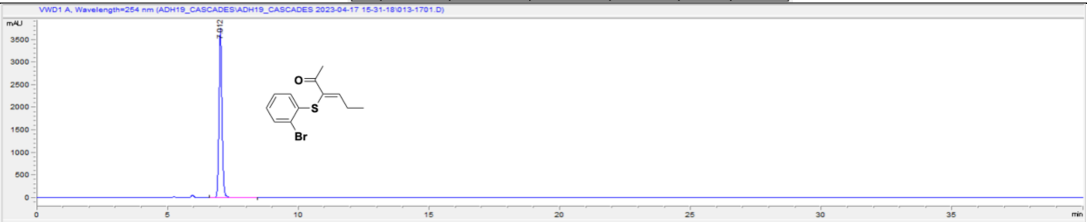

#	Time	Area	Height	Width	Area%	Symmetry
1	7.012	28010.5	3761	0.1073	100.000	0.867

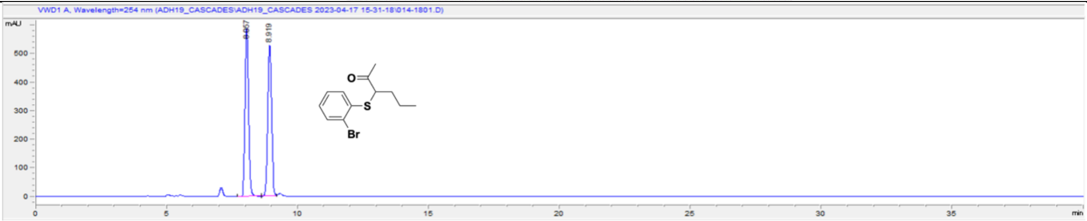

#	Time	Area	Height	Width	Area%	Symmetry
1	8.057	5340.1	588.8	0.1422	50.112	0.899
2	8.919	5316.3	528.8	0.1566	49.888	0.892

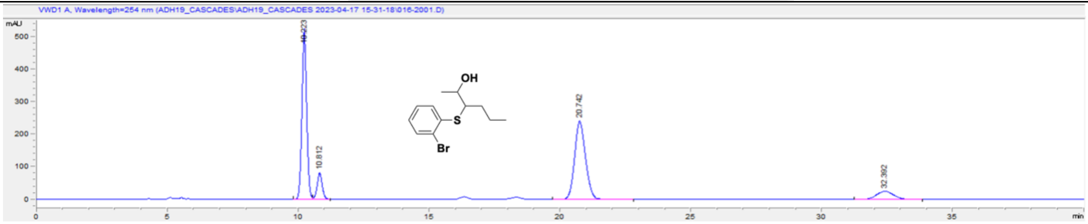

#	Time	Area	Height	Width	Area%	Symmetry
1	10.223	7090.7	525	0.21	43.269	0.927
2	10.812	1182.8	83	0.2245	7.218	0.943
3	20.742	7007.5	242	0.4513	42.761	0.825
4	32.392	1106.4	25.1	0.6913	6.752	0.924

Obtained from two-step one-pot ENE-101/ADH-19 cascade

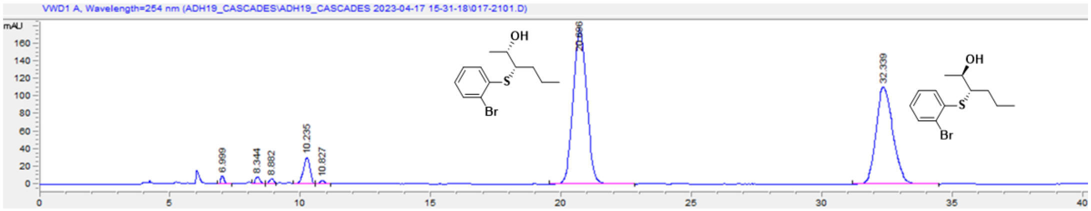

#	Time	Area	Height	Width	Area%	Symmetry
1	6.999	83.3	9.3	0.1406	0.683	0.909
2	8.344	94.2	8	0.1847	0.772	0.898
3	8.882	58.8	5.5	0.1681	0.482	0.925
4	10.235	506.5	30.1	0.2572	4.153	1.184
5	10.827	51.9	3.6	0.2246	0.425	0.938
6	20.696	6461.3	176.8	0.575	52.977	0.921
7	32.339	4940.5	110.4	0.7028	40.508	0.75

HPLC Analysis for One-step One-pot ENE-101/ADH-19 Cascade

HPLC analysis of **10ab**: Chiralpak® IG column (4.6 mm × 250 mm, 5 μm); detected at 254 nm; heptane/EtOH = 95/5; flow rate: 0.8 mL/min.

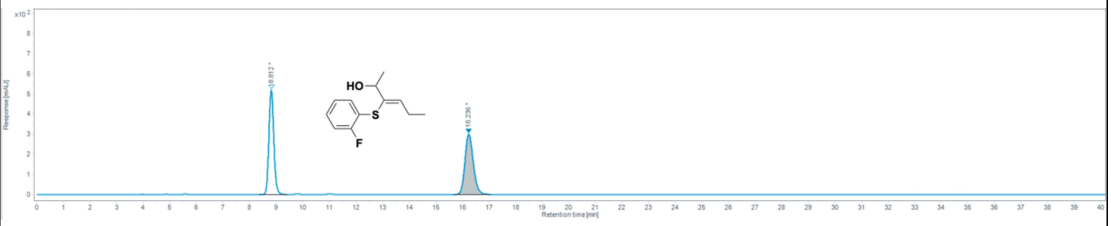

#	RT (min)	Area (mAU·s)	Area%	Height (mAU)	Symmetry	Width (min)
1	8.812	6620.245	50.215	518.977	0.88265	1.062
2	16.236	6563.475	49.785	300.066	0.80626	1.432

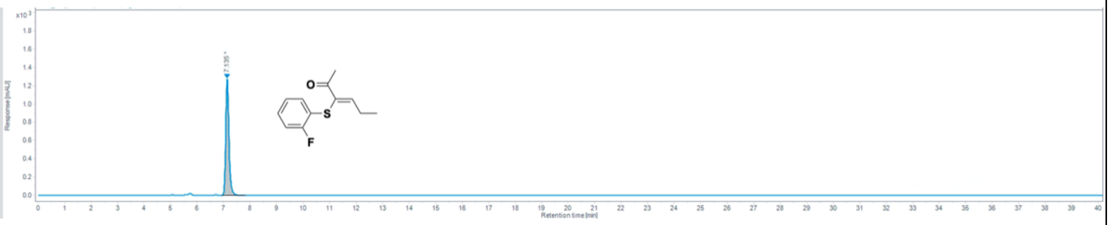

#	RT (min)	Area (mAU·s)	Area%	Height (mAU)	Symmetry	Width (min)
1	7.135	10740.057	100.000	1270.861	0.76315	0.893

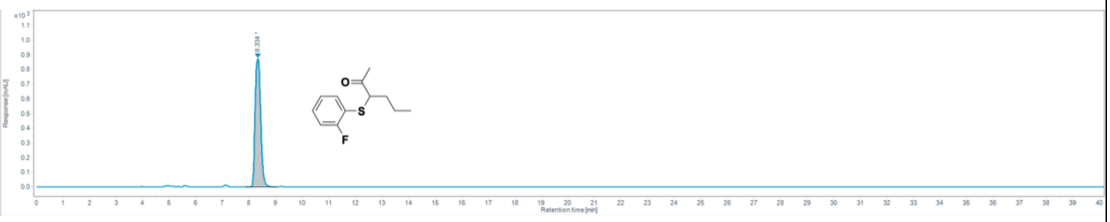

#	RT (min)	Area (mAU·s)	Area%	Height (mAU)	Symmetry	Width (min)
1	8.334	12751.604	100.000	875.687	0.92007	1.170

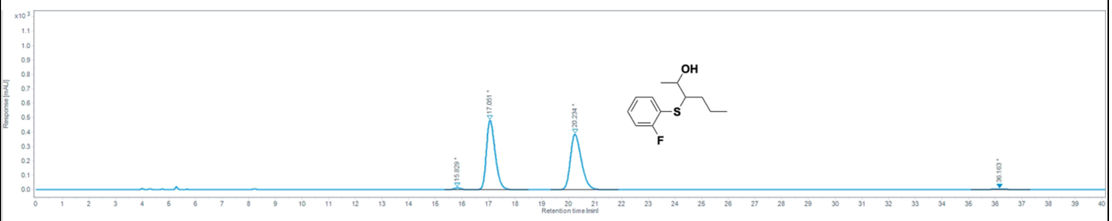

#	RT (min)	Area (mAU·s)	Area%	Height (mAU)	Symmetry	Width (min)
1	15.829	234.287	1.036	12.015	0.95636	0.910
2	17.051	11082.544	48.994	482.030	0.67249	2.221
3	20.234	11070.080	48.939	384.840	0.67190	2.528
4	36.163	233.185	1.031	4.876	0.98374	2.227

Obtained from one-step one-pot ENE-101/ADH-19 cascade

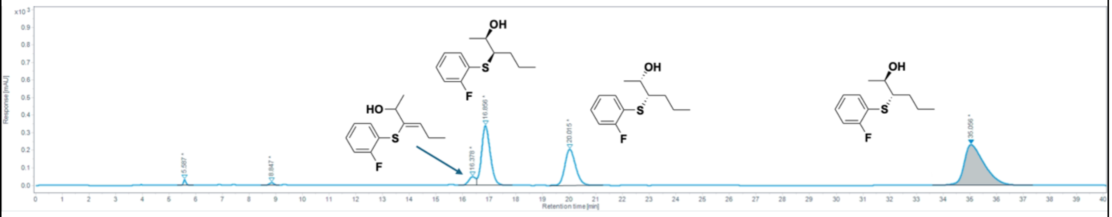

#	RT (min)	Area (mAU·s)	Area%	Height (mAU)	Symmetry	Width (min)
1	5.587	218.093	0.805	31.575	0.79855	0.597
2	8.847	181.452	0.670	14.432	0.96626	0.689
3	16.378	872.729	3.223	48.206	1.32589	0.680
4	16.856	7725.680	28.527	338.412	0.76070	1.337
5	20.015	5806.277	21.440	205.811	0.77250	1.997
6	35.056	12277.630	45.335	231.648	0.51458	3.751

HPLC analysis of **10ac**: Chiralpak® IG column (4.6 mm × 250 mm, 5 µm); detected at 254 nm; heptane/EtOH = 95/5; flow rate: 0.8 mL/min.

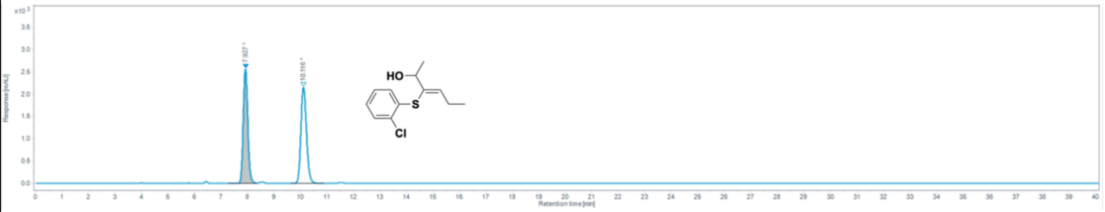

#	RT (min)	Area (mAU·s)	Area%	Height (mAU)	Symmetry	Width (min)
1	7.927	30980.521	49.553	2555.088	0.88060	1.100
2	10.116	31538.839	50.447	2151.056	0.80520	1.251

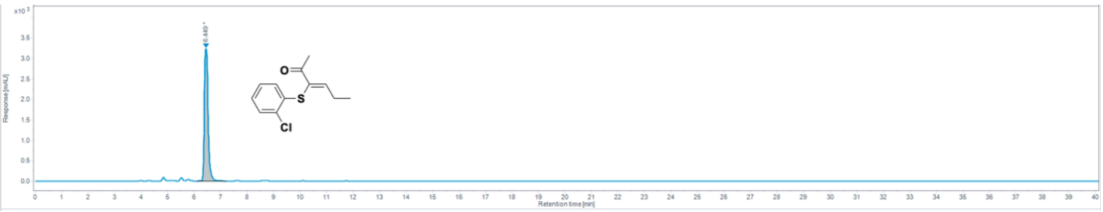

#	RT (min)	Area (mAU·s)	Area%	Height (mAU)	Symmetry	Width (min)
1	6.449	32111.736	100.000	3238.288	0.69886	1.102

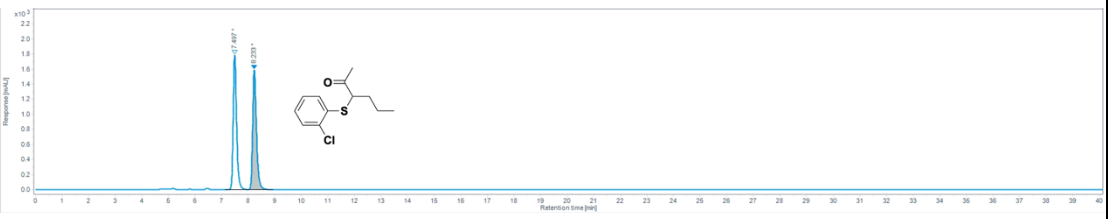

#	RT (min)	Area (mAU·s)	Area%	Height (mAU)	Symmetry	Width (min)
1	7.497	15806.374	49.897	1783.771	0.74099	0.821
2	8.233	15871.897	50.103	1586.164	0.67294	0.967

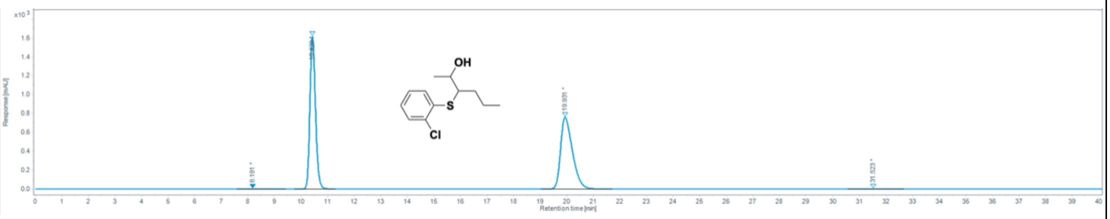

#	RT (min)	Area (mAU·s)	Area%	Height (mAU)	Symmetry	Width (min)
1	8.181	84.131	0.178	2.169	0.52961	1.836
2	10.428	23585.146	49.820	1616.833	0.78114	1.530
3	19.931	23572.211	49.793	760.002	0.57411	2.679
4	31.523	99.149	0.209	2.360	0.87573	2.105

Obtained from one-step one-pot ENE-101/ADH-19 cascade

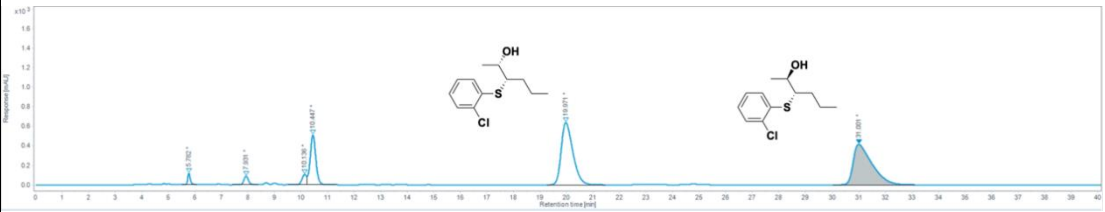

#	RT (min)	Area (mAU·s)	Area%	Height (mAU)	Symmetry	Width (min)
1	5.782	785.433	1.547	116.223	0.75708	0.544
2	7.931	1080.519	2.128	89.022	0.93293	0.974
3	10.136	1198.818	2.361	103.450	1.61702	0.704
4	10.447	7673.662	15.112	511.849	0.91199	1.119
5	19.971	19598.768	38.598	640.035	0.61962	2.212
6	31.001	20439.901	40.254	416.739	0.39057	3.073

HPLC analysis of **10ad**: Chiralpak® IG column (4.6 mm × 250 mm, 5 µm); detected at 254 nm; heptane/EtOH = 95/5; flow rate: 0.8 mL/min.

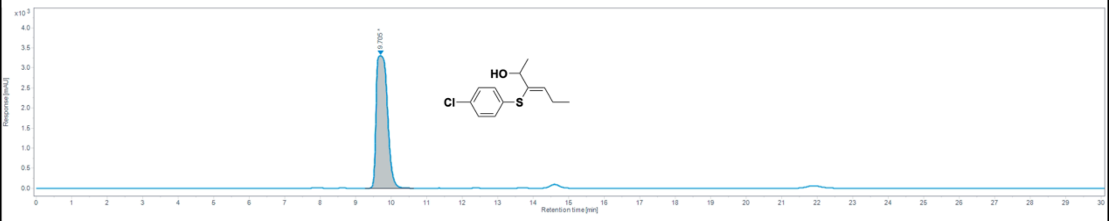

#	RT (min)	Area (mAU·s)	Area%	Height (mAU)	Symmetry	Width (min)
1	9.705	72437.022	100.000	3298.365	0.58804	1.365

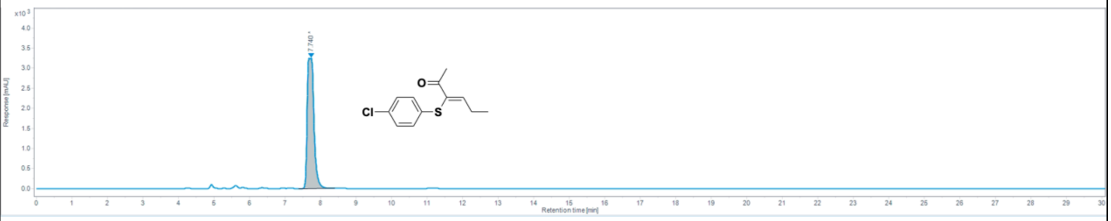

#	RT (min)	Area (mAU·s)	Area%	Height (mAU)	Symmetry	Width (min)
1	7.740	42588.055	100.000	3242.846	1.25056	1.019

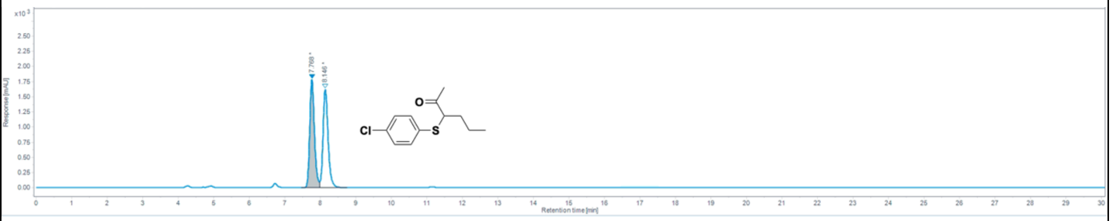

#	RT (min)	Area (mAU·s)	Area%	Height (mAU)	Symmetry	Width (min)
1	7.768	16483.776	50.538	1782.142	0.76816	0.510
2	8.146	16132.721	49.462	1623.186	0.74761	0.760

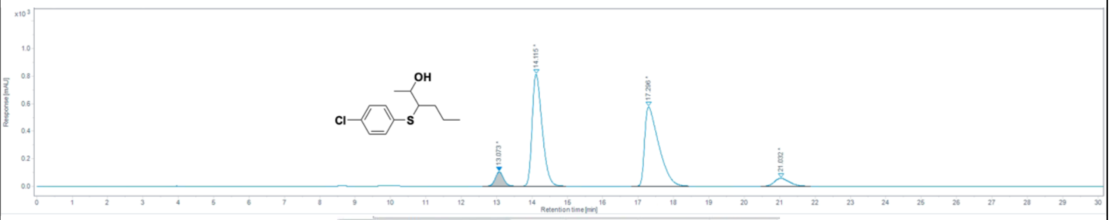

#	RT (min)	Area (mAU·s)	Area%	Height (mAU)	Symmetry	Width (min)
1	13.073	1707.764	4.861	103.514	0.88561	1.118
2	14.115	15874.754	45.182	813.290	0.60941	1.239
3	17.296	15845.146	45.098	574.463	0.36356	1.618
4	21.032	1707.164	4.859	57.817	0.71888	1.408

Obtained from one-step one-pot ENE-101/ADH-19 cascade

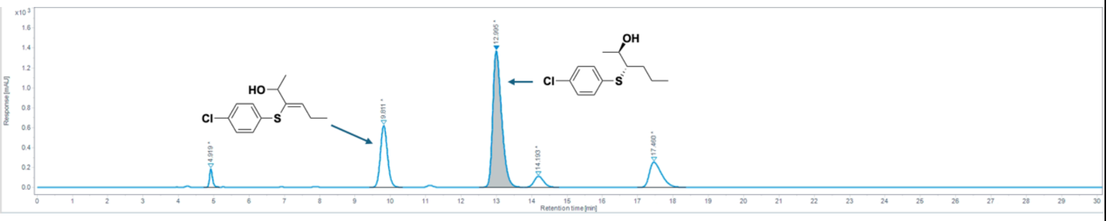

#	RT (min)	Area (mAU·s)	Area%	Height (mAU)	Symmetry	Width (min)
1	4.919	1088.734	2.581	183.800	0.76351	0.443
2	9.811	8648.603	20.502	620.604	0.85383	0.951
3	12.995	24173.556	57.305	1368.748	0.61021	1.255
4	14.193	2127.252	5.043	110.855	0.83064	0.993
5	17.460	6146.151	14.570	253.401	0.56096	1.363

HPLC Analysis for Three-Step One-Pot Biocatalytic-Biocatalytic-Biocatalytic (Bio-Bio-Bio) Cascade for the Enantioselective Synthesis of 10 from 9

HPLC analysis of **10aa**: Chiralpak® IG column (4.6 mm × 250 mm, 5 µm); detected at 254 nm; heptane/EtOH = 95/5; flow rate: 0.8 mL/min.

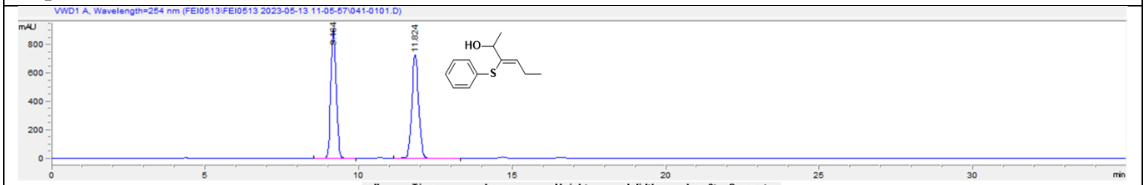

#	Time	Area	Height	Width	Area%	Symmetry
1	9.164	11290.4	910.9	0.1941	49.678	0.911
2	11.824	11436.6	726.7	0.2448	50.322	0.904

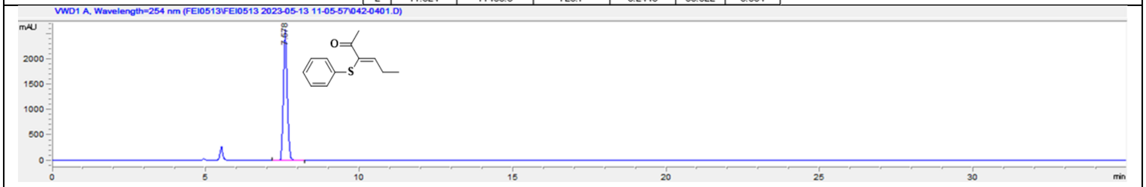

#	Time	Area	Height	Width	Area%	Symmetry
1	7.578	22665.3	2587.4	0.1371	100.000	0.858

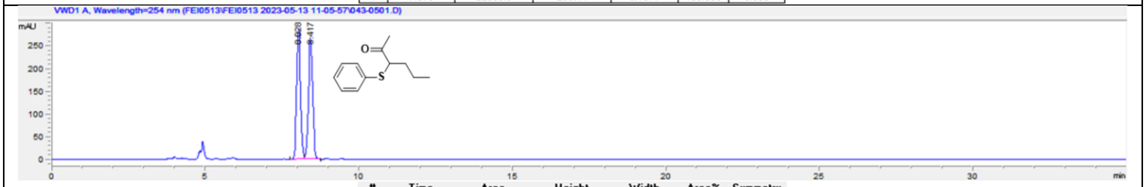

#	Time	Area	Height	Width	Area%	Symmetry
1	8.028	2571.3	288.6	0.1389	50.097	0.936
2	8.417	2561.3	268.4	0.1492	49.903	0.927

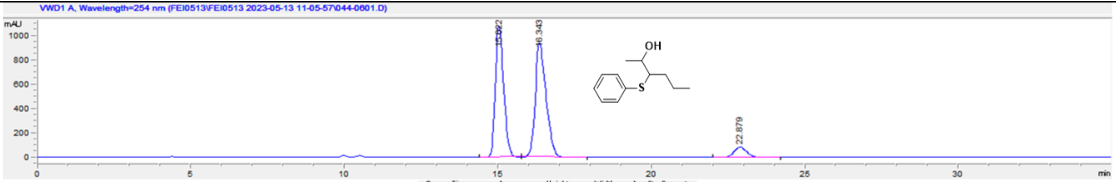

#	Time	Area	Height	Width	Area%	Symmetry
1	15.022	20515	1077.8	0.2949	44.918	0.702
2	16.343	22806	939	0.3649	49.934	0.6
3	22.879	2351.3	82.8	0.4419	5.148	0.895

Obtained with ADH-159

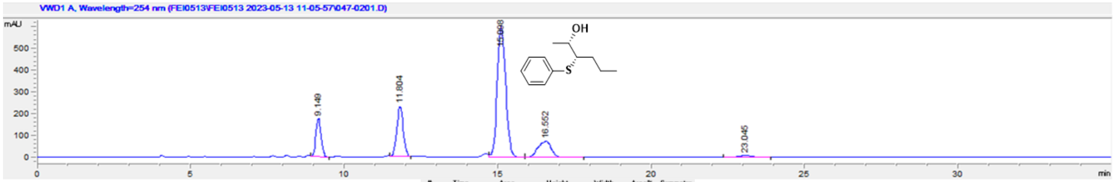

#	Time	Area	Height	Width	Area%	Symmetry
1	9.149	2158.9	176.7	0.192	11.095	0.947
2	11.804	3468.5	231.2	0.2362	17.826	0.965
3	15.098	11408.6	602.8	0.2936	58.632	0.806
4	16.552	2184.7	73.2	0.4968	11.228	1.374
5	23.045	237.2	8.1	0.4541	1.219	0.961

HPLC analysis of **10aa**: Chiralpak® IG column (4.6 mm × 250 mm, 5 μm); detected at 254 nm; heptane/EtOH = 95/5; flow rate: 0.8 mL/min.

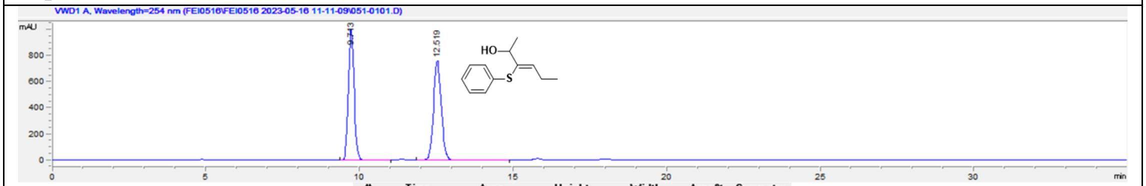

#	Time	Area	Height	Width	Area%	Symmetry
1	9.713	13122.2	1005	0.2047	49.410	0.863
2	12.519	13435.8	761.8	0.2727	50.590	0.894

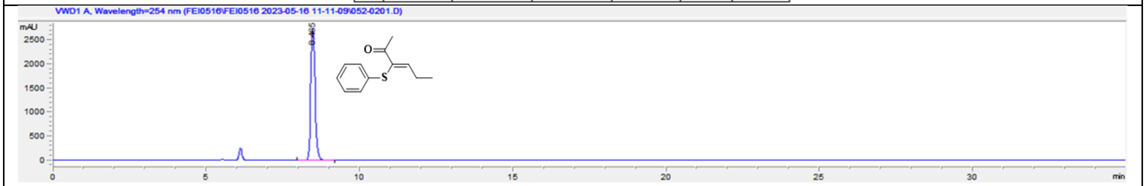

#	Time	Area	Height	Width	Area%	Symmetry
1	8.465	26178.2	2718.7	0.1502	100.000	0.813

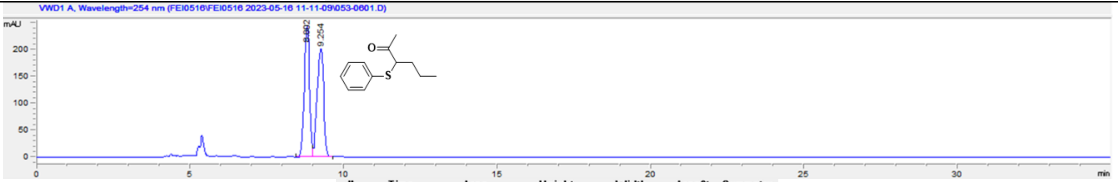

#	Time	Area	Height	Width	Area%	Symmetry
1	8.802	2990.9	243.7	0.1985	49.940	1.17
2	9.254	2998	200.9	0.2498	50.060	1.378

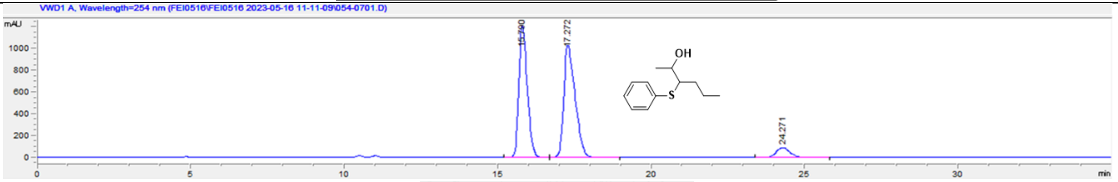

#	Time	Area	Height	Width	Area%	Symmetry
1	15.79	23100.7	1207.5	0.296	44.336	0.671
2	17.272	26253	1032.3	0.387	50.386	0.529
3	24.271	2750.2	89.7	0.4801	5.278	0.863

Obtained with ADH-153

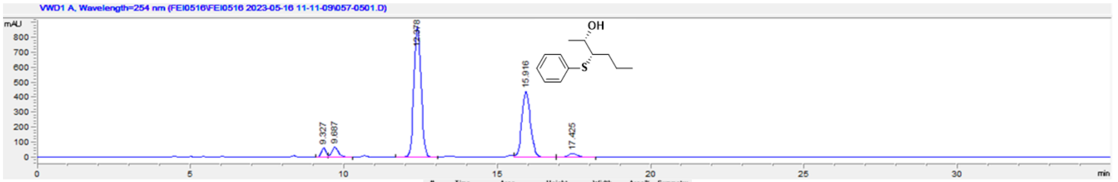

#	Time	Area	Height	Width	Area%	Symmetry
1	9.327	674.7	64.5	0.1629	2.705	0.919
2	9.687	993.6	68.4	0.2219	3.983	0.745
3	12.378	14158.7	877.1	0.2524	56.762	0.888
4	15.916	8590.5	441.2	0.3029	34.439	0.824
5	17.425	526.6	24.3	0.3319	2.111	0.872

HPLC analysis of **10ab**: Chiralpak® IG column (4.6 mm × 250 mm, 5 µm); detected at 254 nm; heptane/EtOH = 95/5; flow rate: 0.8 mL/min.

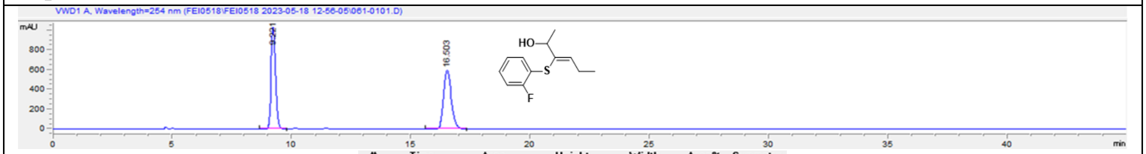

#	Time	Area	Height	Width	Area%	Symmetry
1	9.221	13498.4	1026.9	0.2058	50.582	0.908
2	16.503	13187.6	592.8	0.344	49.418	0.739

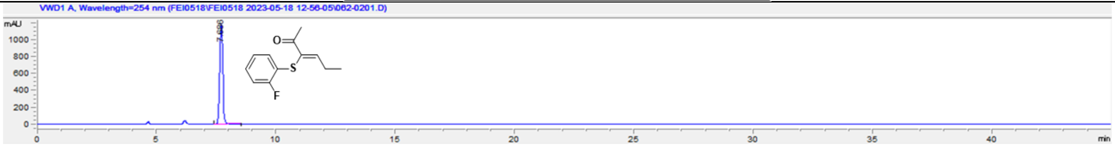

#	Time	Area	Height	Width	Area%	Symmetry
1	7.686	10691.1	1187.7	0.14	100.000	0.837

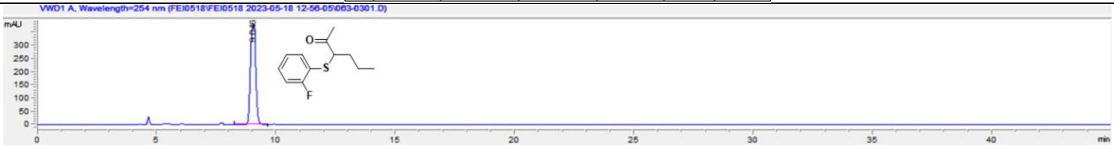

#	Time	Area	Height	Width	Area%	Symmetry
1	9.046	6057.5	378.3	0.2596	100.000	1.12

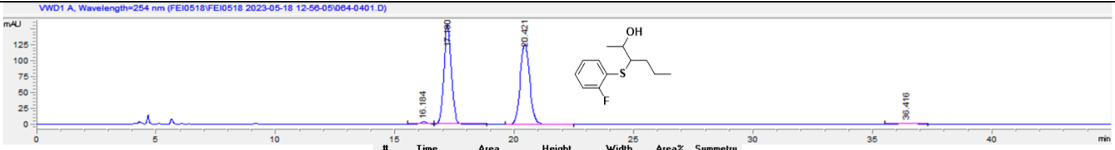

#	Time	Area	Height	Width	Area%	Symmetry
1	16.184	84.2	3.8	0.3353	1.193	0.926
2	17.18	3438.2	157	0.34	48.709	0.87
3	20.421	3469.8	125.4	0.4334	49.156	0.897
4	36.416	66.6	1.5	0.5685	0.943	0.911

Obtained with ADH-159

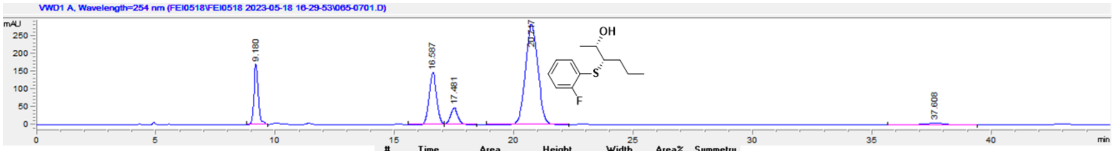

#	Time	Area	Height	Width	Area%	Symmetry
1	9.18	2272.8	170.1	0.2083	13.266	0.87
2	16.587	3210.1	146.1	0.3407	18.737	0.924
3	17.481	1032.3	47.4	0.3413	6.025	0.914
4	20.717	10314.5	282.2	0.5604	60.204	0.912
5	37.608	302.8	4.9	0.8942	1.768	1.322

Obtained with ADH-153

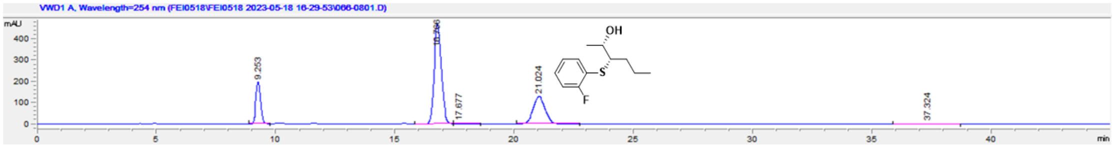

#	Time	Area	Height	Width	Area%	Symmetry
1	9.253	2722.9	195.7	0.2206	15.704	0.945
2	16.766	9990.7	469.3	0.3299	57.622	0.823
3	17.677	62.4	1.9	0.4547	0.360	0.472
4	21.024	4508.3	128.9	0.5419	26.002	0.935
5	37.324	53.9	1	0.691	0.311	0.906

HPLC analysis of **10ac**: Chiralpak® IG column (4.6 mm × 250 mm, 5 μm); detected at 254 nm; heptane/EtOH = 95/5; flow rate: 0.8 mL/min.

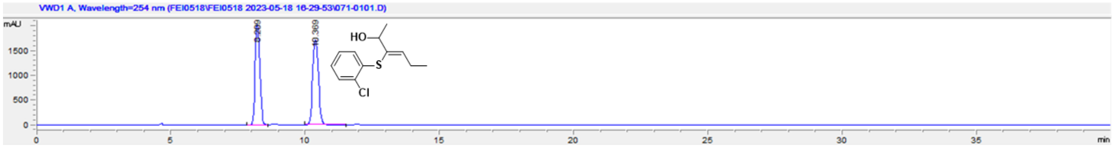

#	Time	Area	Height	Width	Area%	Symmetry
1	8.209	24331.8	2031.1	0.1864	48.966	0.894
2	10.369	25359.2	1726.7	0.2325	51.034	0.875

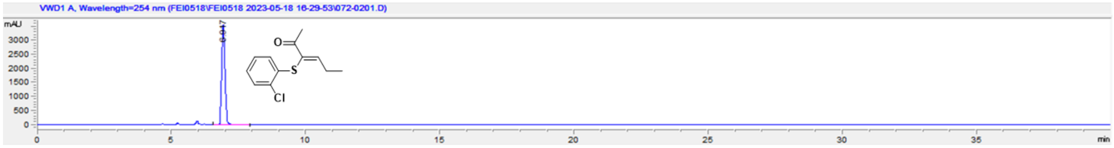

#	Time	Area	Height	Width	Area%	Symmetry
1	6.917	27827.5	3556	0.1204	100.000	0.839

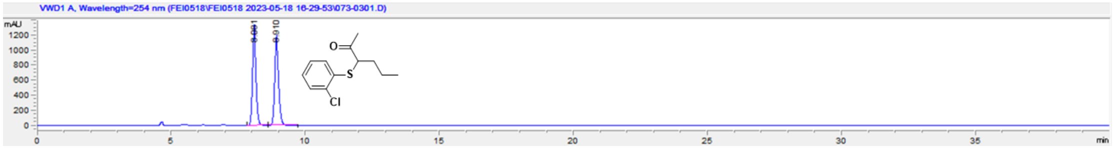

#	Time	Area	Height	Width	Area%	Symmetry
1	8.081	12445.9	1337	0.1465	50.069	0.84
2	8.91	12411.5	1198.3	0.1616	49.931	0.784

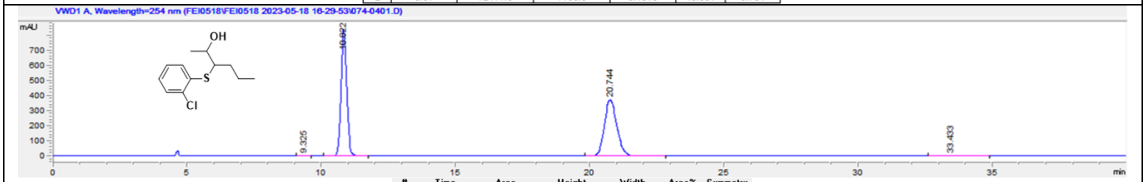

#	Time	Area	Height	Width	Area%	Symmetry
1	9.325	12.3	9.6E-1	0.1992	0.050	0.945
2	10.822	12430.7	841.3	0.2306	50.806	0.873
3	20.744	11969.5	372.6	0.5	48.922	0.796
4	33.433	54.2	1.2	0.5408	0.222	0.839

Obtained with ADH-159

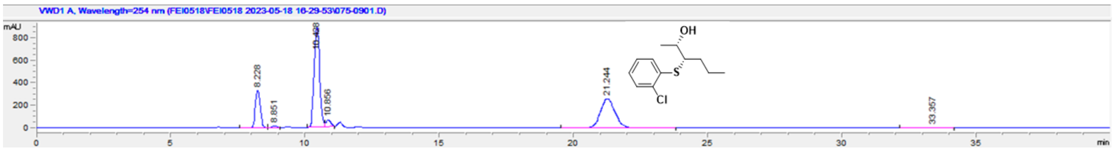

#	Time	Area	Height	Width	Area%	Symmetry
1	8.228	4128	332.8	0.1942	14.965	0.928
2	8.851	221.8	17.2	0.1974	0.804	0.92
3	10.428	12621.4	888.7	0.2239	45.755	0.884
4	10.856	784.7	61.5	0.2013	2.845	0.918
5	21.244	9696.3	259.2	0.5821	35.151	0.844
6	33.357	132.3	3	0.6564	0.480	0.91

HPLC analysis of **10ac**: Chiralpak® IG column (4.6 mm × 250 mm, 5 μm); detected at 254 nm; heptane/EtOH = 95/5; flow rate: 0.8 mL/min.

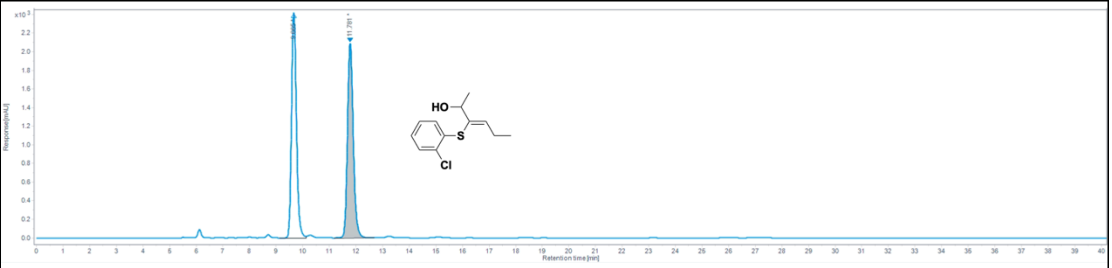

#	RT (min) △	Area (mAU·s)	Area%	Height (mAU)	Symmetry	Width (min)
1	9.665	30948.077	49.489	2417.062	0.83147	0.997
2	11.781	31587.282	50.511	2091.345	0.80878	1.529

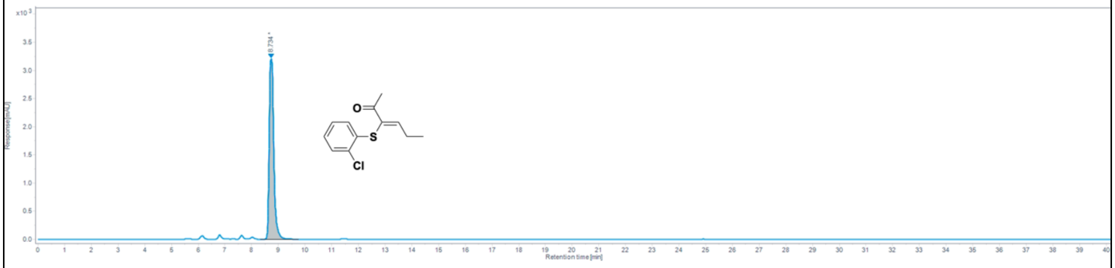

#	RT (min) △	Area (mAU·s)	Area%	Height (mAU)	Symmetry	Width (min)
1	8.734	38501.563	100.000	3205.639	0.74058	1.422

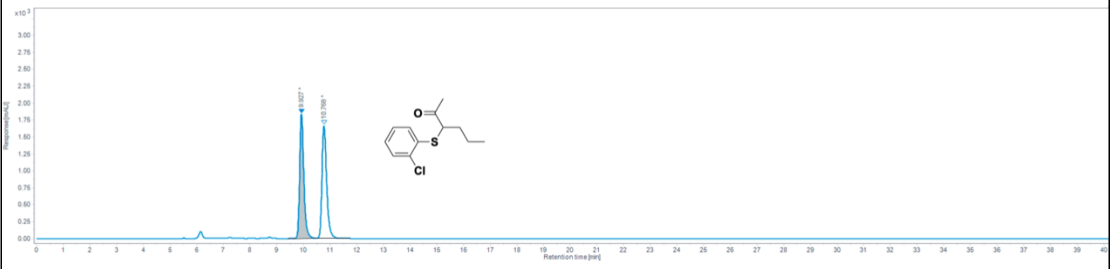

#	RT (min) △	Area (mAU·s)	Area%	Height (mAU)	Symmetry	Width (min)
1	9.927	20079.652	49.912	1838.115	0.73286	1.015
2	10.768	20150.139	50.088	1665.723	0.67209	1.310

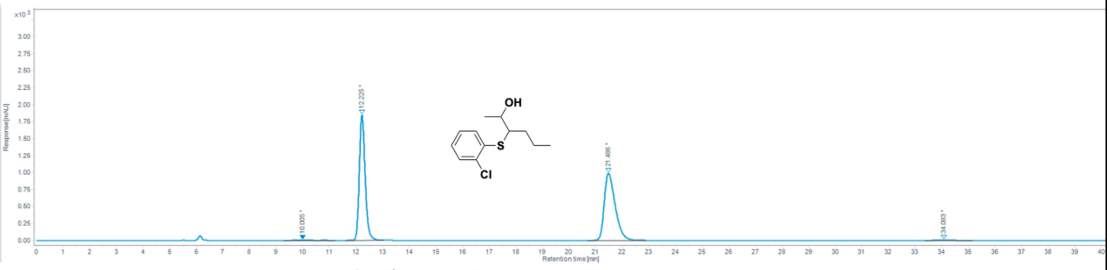

#	RT (min) △	Area (mAU·s)	Area%	Height (mAU)	Symmetry	Width (min)
1	10.005	100.763	0.178	2.324	0.59543	1.936
2	12.225	28161.540	49.812	1845.738	0.77577	1.414
3	21.486	28158.987	49.808	988.586	0.58842	2.192
4	34.083	114.148	0.202	2.838	0.88848	1.777

Obtained with ADH-19

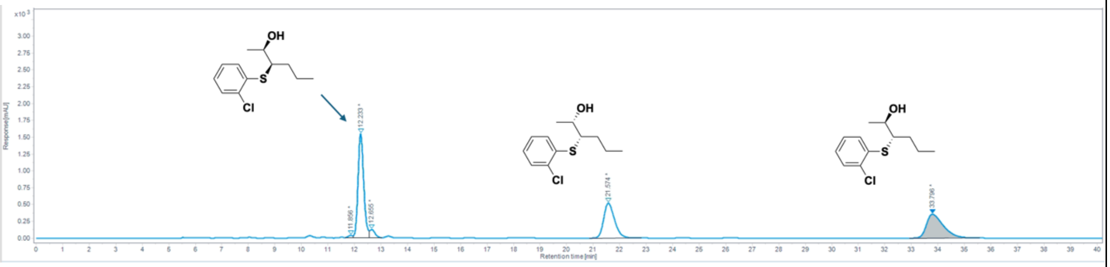

#	RT (min) △	Area (mAU·s)	Area%	Height (mAU)	Symmetry	Width (min)
1	11.856	427.000	0.767	34.187	1.23613	0.324
2	12.233	23093.258	41.488	1547.675	0.80619	0.599
3	12.655	1652.758	2.969	120.039	0.58652	0.490
4	21.574	14680.815	26.375	517.453	0.71257	1.946
5	33.796	15808.525	28.401	353.771	0.52309	2.660

HPLC analysis of **10ad**: Chiralpak® IG column (4.6 mm × 250 mm, 5 μm); detected at 254 nm; heptane/EtOH = 95/5; flow rate: 0.8 mL/min.

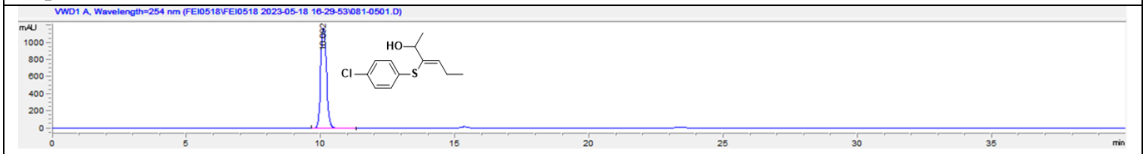

#	Time	Area	Height	Width	Area%	Symmetry
1	10.092	17494.4	1168.6	0.2358	100.000	0.843

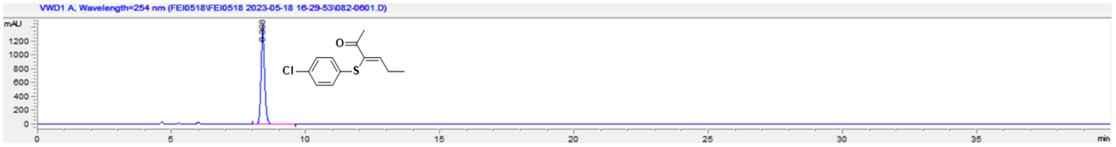

#	Time	Area	Height	Width	Area%	Symmetry
1	8.388	13886	1446.9	0.1498	100.000	0.841

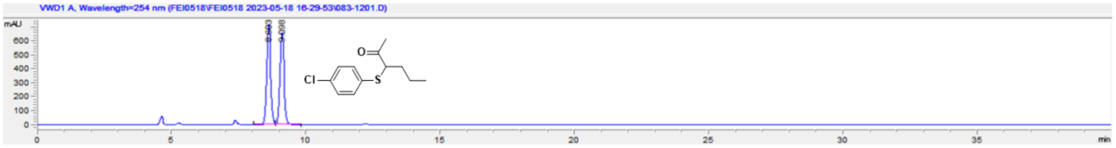

#	Time	Area	Height	Width	Area%	Symmetry
1	8.603	7356.3	718.6	0.1573	51.400	0.9
2	9.098	6955.7	657.2	0.1671	48.600	0.869

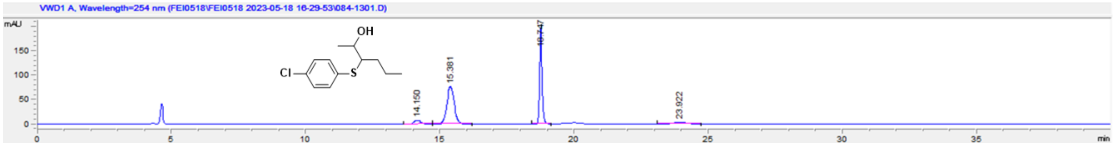

#	Time	Area	Height	Width	Area%	Symmetry
1	14.15	134.5	7.6	0.2768	4.309	0.983
2	15.381	1469.2	77.2	0.2979	47.055	0.91
3	18.747	1386.5	204.3	0.1054	44.405	0.814
4	23.922	132.1	3.8	0.5387	4.231	0.954

Obtained with ADH-159

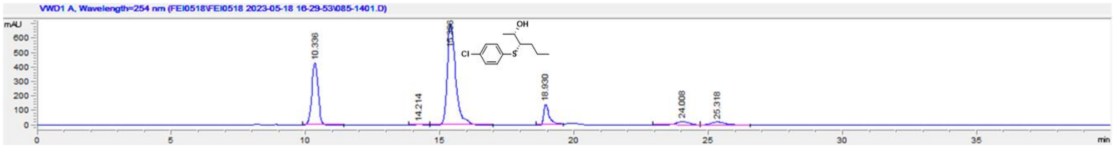

#	Time	Area	Height	Width	Area%	Symmetry
1	10.336	6873.1	427.6	0.2516	26.251	0.992
2	14.214	101.9	4.8	0.3129	0.389	0.635
3	15.386	15345.4	691.8	0.3403	58.610	0.609
4	18.93	2139.1	141.7	0.2169	8.170	0.421
5	24.008	854.4	23.6	0.5506	3.263	0.941
6	25.318	868.3	21.5	0.6047	3.316	0.729

Obtained with ADH-153

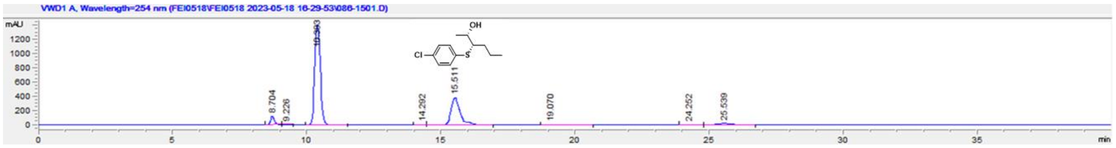

#	Time	Area	Height	Width	Area%	Symmetry
1	8.704	1248.3	120.9	0.1612	4.029	0.853
2	9.226	70.2	5.8	0.1869	0.227	0.833
3	10.383	19963	1408.9	0.2235	64.441	0.845
4	14.292	11.1	6.3E-1	0.2631	0.036	1.023
5	15.511	8810.7	379.8	0.358	28.441	0.686
6	19.07	60.7	2.2	0.3725	0.196	0.454
7	24.252	85.2	2.5	0.5205	0.275	0.979
8	25.539	729.5	19.6	0.5796	2.355	0.859

HPLC analysis of **10ad**: Chiralpak® IG column (4.6 mm × 250 mm, 5 μm); detected at 254 nm; heptane/EtOH = 95/5; flow rate: 0.8 mL/min.

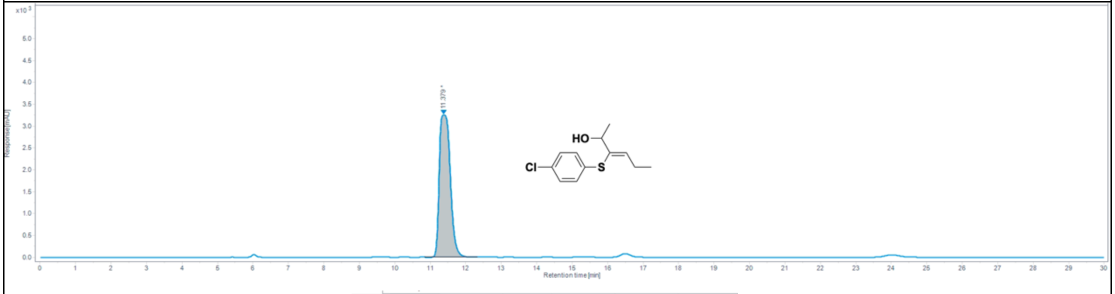

#	RT (min)	Area (mAU·s)	Area%	Height (mAU)	Symmetry	Width (min)
1	11.379	69942.464	100.000	3255.476	0.60138	1.493

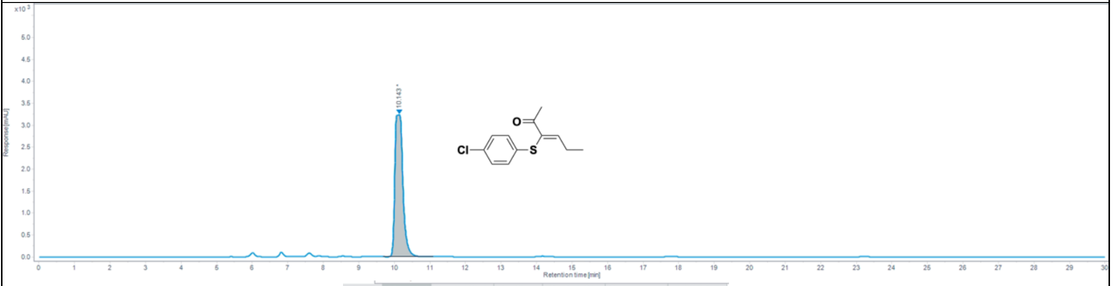

#	RT (min)	Area (mAU·s)	Area%	Height (mAU)	Symmetry	Width (min)
1	10.143	51403.325	100.000	3237.003	1.15811	1.415

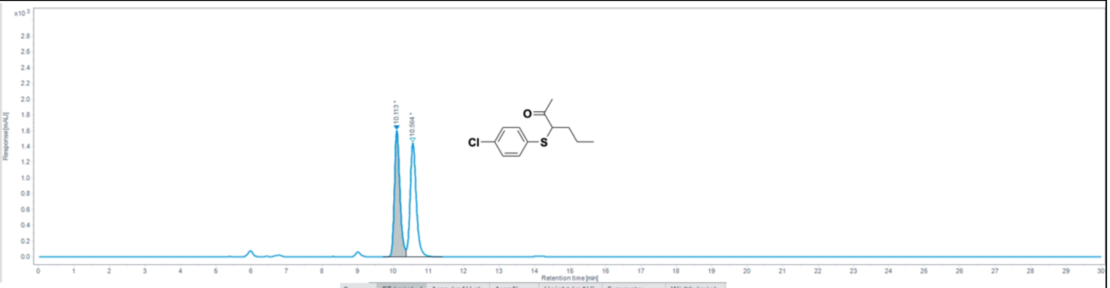

#	RT (min)	Area (mAU·s)	Area%	Height (mAU)	Symmetry	Width (min)
1	10.113	17649.566	50.438	1604.104	0.76074	0.654
2	10.564	17342.896	49.562	1445.080	0.74856	1.044

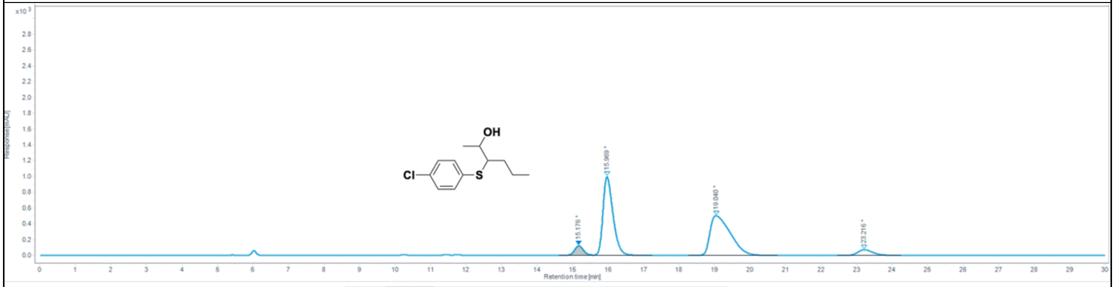

#	RT (min)	Area (mAU·s)	Area%	Height (mAU)	Symmetry	Width (min)
1	15.176	2126.704	4.859	122.017	0.88152	0.968
2	15.969	19665.924	44.933	994.829	0.63640	1.655
3	19.040	19870.132	45.400	502.673	0.39580	2.517
4	23.216	2104.169	4.808	71.505	0.72437	1.786

Obtained with ADH-19

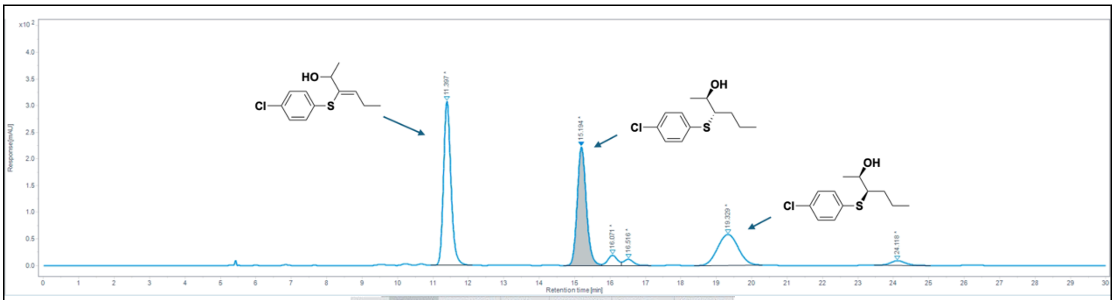

#	RT (min)	Area (mAU·s)	Area%	Height (mAU)	Symmetry	Width (min)
1	11.397	4478.874	38.218	308.004	0.80713	1.163
2	15.194	4003.784	34.164	222.574	0.82087	1.089
3	16.071	387.625	3.308	19.979	0.98905	0.553
4	16.516	268.413	2.290	12.500	0.71535	0.838
5	19.329	2343.753	19.999	57.717	0.96010	1.919
6	24.118	236.909	2.022	8.352	0.88632	1.591

HPLC analysis of **10ae**: Chiralpak® IG column (4.6 mm × 250 mm, 5 μm); detected at 254 nm; heptane/EtOH = 95/5; flow rate: 0.8 mL/min.

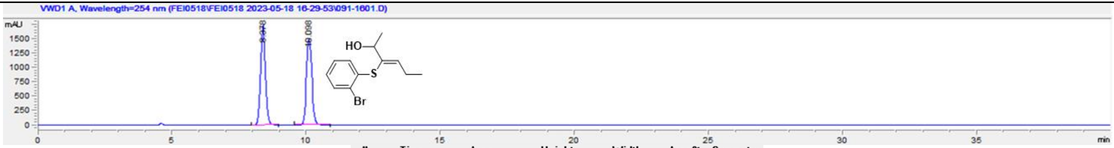

#	Time	Area	Height	Width	Area%	Symmetry
1	8.378	22066.4	1740.6	0.2003	50.895	0.909
2	10.098	21290.6	1486.7	0.2253	49.105	0.875

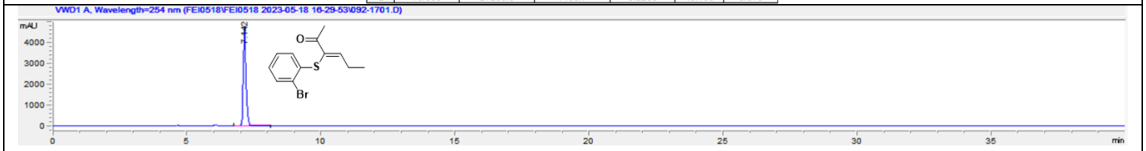

#	Time	Area	Height	Width	Area%	Symmetry
1	7.142	32044.8	4754.7	0.099	100.000	0.863

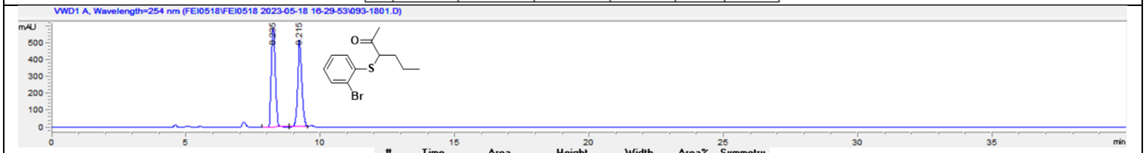

#	Time	Area	Height	Width	Area%	Symmetry
1	8.235	5693.8	593.2	0.1498	49.552	0.854
2	9.215	5796.8	519	0.174	50.448	0.862

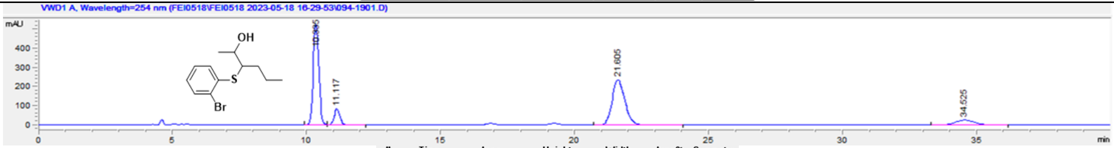

#	Time	Area	Height	Width	Area%	Symmetry
1	10.335	7556.2	525.2	0.2261	42.611	0.899
2	11.117	1230.5	83.1	0.228	6.939	0.916
3	21.605	7730.3	236.6	0.5035	43.593	0.803
4	34.525	1216	25	0.7719	6.858	0.896

Obtained with ADH-159

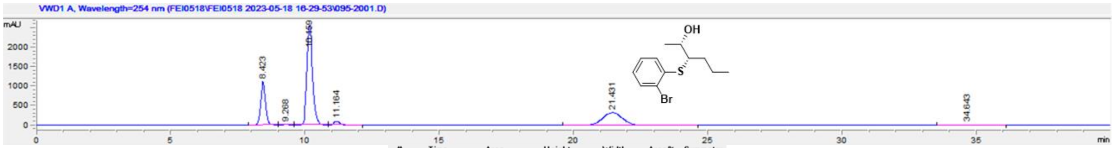

#	Time	Area	Height	Width	Area%	Symmetry
1	8.423	13994.5	1117.1	0.1957	19.962	0.923
2	9.268	252.1	18.4	0.2148	0.360	0.952
3	10.159	38108.4	2575.6	0.2279	54.358	0.805
4	11.164	1432.7	97.2	0.2274	2.044	0.922
5	21.431	16269.2	321.3	0.8004	23.206	0.946
6	34.643	49.4	9.4E-1	0.6855	0.070	1.054

HPLC Analysis of Deuterium-Labeling Experiments

HPLC analysis of deuterium-labeling experiments A: Chiralpak® IG column (4.6 mm × 250 mm, 5 μm); detected at 254 nm; heptane/EtOH = 90/10; flow rate: 1.0 mL/min.

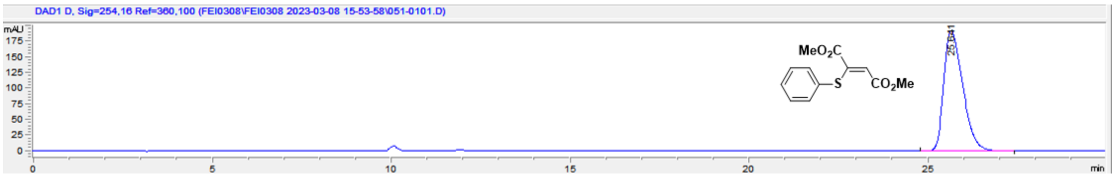

#	Time	Area	Height	Width	Area%	Symmetry
1	25.641	7002.2	192.7	0.558	100.000	0.608

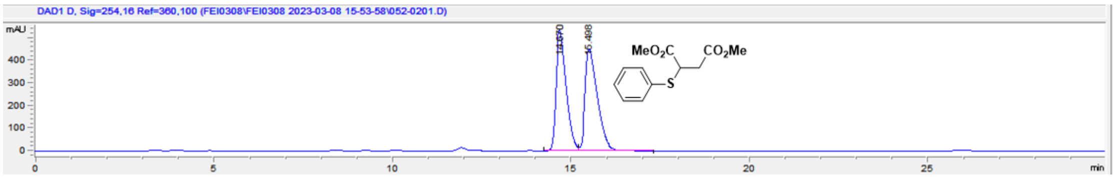

#	Time	Area	Height	Width	Area%	Symmetry
1	14.67	10655.3	533.2	0.3071	49.926	0.55
2	15.498	10687	449	0.3597	50.074	0.47

Obtained in deuterated KPBS (250 mM, pH 7.0)

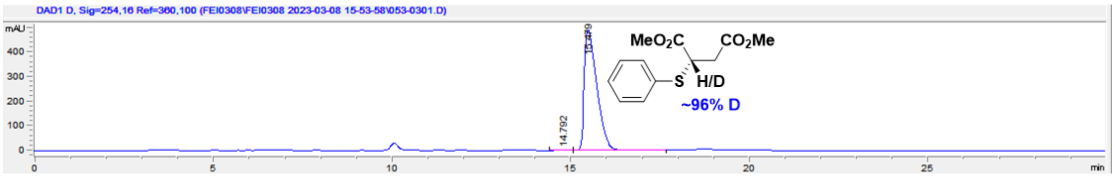

#	Time	Area	Height	Width	Area%	Symmetry
1	14.792	25.3	1.4	0.2604	0.213	0.983
2	15.479	11862.7	491.3	0.3717	99.787	0.426

HPLC analysis of deuterium-labeling experiments B: Chiralpak® IG column (4.6 mm × 250 mm, 5 μm); detected at 254 nm; heptane/EtOH = 90/10; flow rate: 1.0 mL/min.

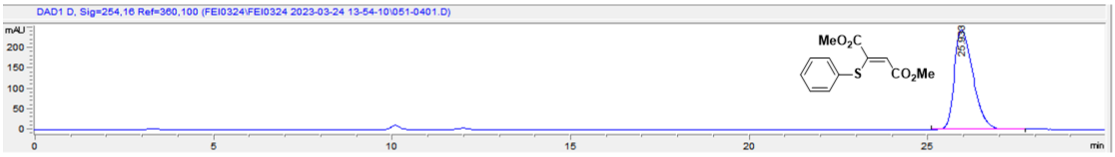

#	Time	Area	Height	Width	Area%	Symmetry
1	25.933	8808.8	243.3	0.5584	100.000	0.599

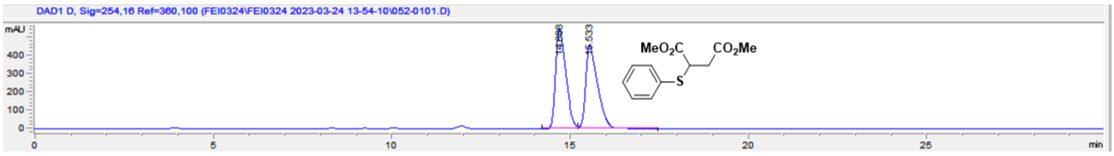

#	Time	Area	Height	Width	Area%	Symmetry
1	14.688	10670.1	544	0.3008	49.691	0.553
2	15.533	10802.8	458.5	0.3569	50.309	0.475

Obtained with D-glucose-d_{12} in KPBS (250 mM, pH 7.0)

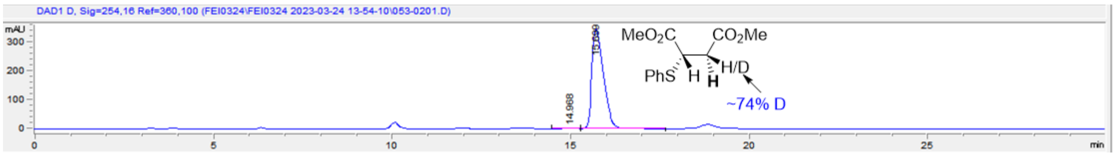

#	Time	Area	Height	Width	Area%	Symmetry
1	14.968	30.4	1.6	0.2841	0.391	1.023
2	15.699	7755.1	345.3	0.342	99.609	0.539

HPLC analysis of deuterium-labeling experiments C: Chiralpak® IG column (4.6 mm × 250 mm, 5 µm); detected at 254 nm; heptane/EtOH = 90/10; flow rate: 1.0 mL/min.

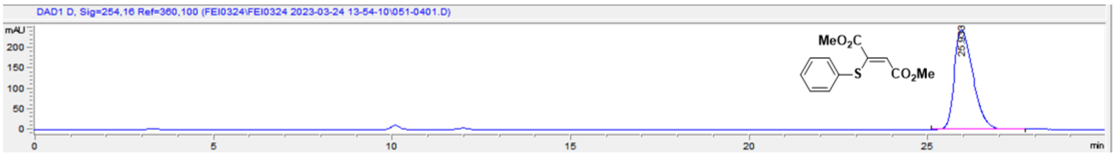

#	Time	Area	Height	Width	Area%	Symmetry
1	25.933	8808.8	243.3	0.5584	100.000	0.599

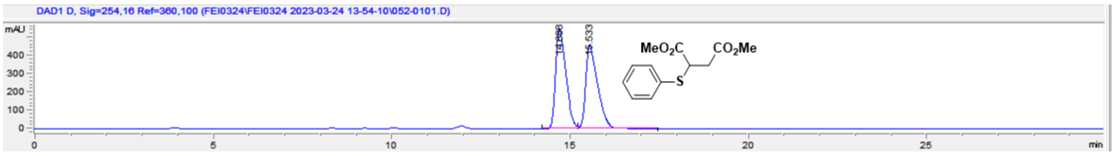

#	Time	Area	Height	Width	Area%	Symmetry
1	14.688	10670.1	544	0.3008	49.691	0.553
2	15.533	10802.8	458.5	0.3569	50.309	0.475

Obtained with D-glucose-d_{12} in deuterated KPBS (250 mM, pH 7.0)

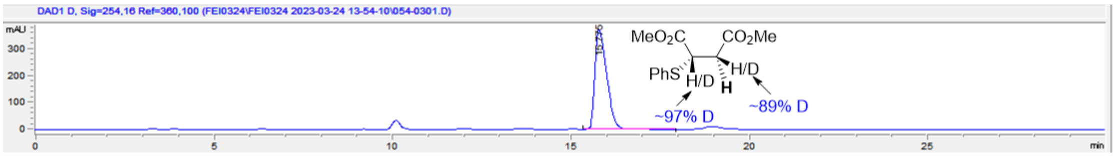

#	Time	Area	Height	Width	Area%	Symmetry
1	15.775	8335.7	372.3	0.3432	100.000	0.506

HPLC analysis of **11aa**: Chiralpak® IG column (4.6 mm × 250 mm, 5 µm); detected at 254 nm; heptane/EtOH = 90/10; flow rate: 1.0 mL/min.

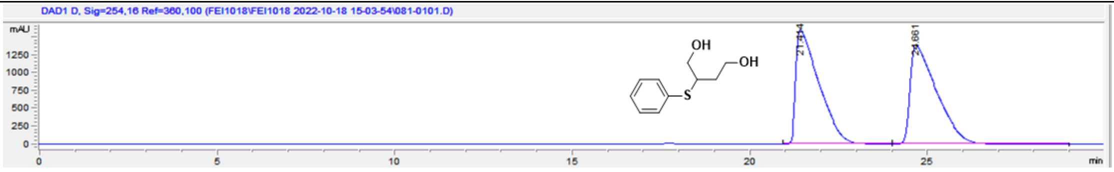

#	Time	Area	Height	Width	Area%	Symmetry
1	21.414	71718.3	1598.9	0.6453	49.774	0.235
2	24.661	72370	1389.3	0.7437	50.226	0.272

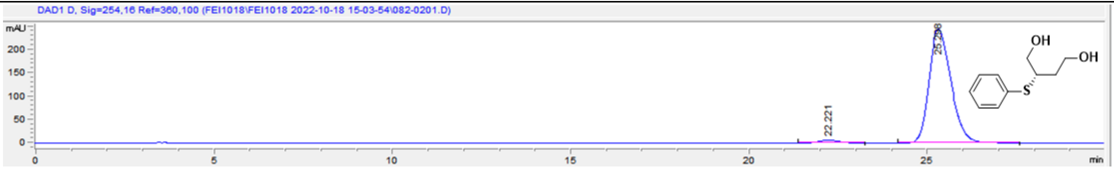

#	Time	Area	Height	Width	Area%	Symmetry
1	22.221	225.1	6.2	0.5433	2.127	0.966
2	25.298	10358.6	244.3	0.6547	97.873	0.705

VII. Supplementary References

1. Domínguez, B., Schell, U., Kratzer, C. & Kalthoff, T. Catalyst and use thereof, US patent 010829743 (2020).

2. Sarrafi, Y., Sadatshahabi, M., Alimohammadi, K. & Tajbakhsh, M. A green and rapid approach for the stereoselective vinylation of phenol, thiol and amine derivatives in water. *Green Chem*. **13**, 2851–2858 (2011).

3. Mosslemin, M. H., Anary-Abbassinejad, M., Hassanabadi, A., Mohebat, M. & Nateghi, M. R. Reaction between triphenylphosphine and acetylenic esters or acetylenic ketones in the presence of mercaptoesters. *Synth. Commun*. **39**, 3482−3492 (2009).

4. Shmidt, M. S., Martini, M. F., Oppezzo, G. A., Blanco, M. M. & Moglioni, A. G. Study of the hydroamination reaction of methyl acetylenedicarboxylate with aromatic amines and its heterocyclization products. *ChemistrySelect* **6**, 1969−1975 (2021).

5. Yang, Z.-H., An, Y.-L., Chen, Y., Shao, Z.-Y. & Zhao, S.-Y. Copper(I) iodide-catalyzed sulfenylation of maleimides and related 3-indolylmaleimides with thiols. *Adv. Synth. Catal*. **358**, 3869−3875 (2016).

6. Jin, Z., Xu, B. & Hammond, G. B. Green synthesis of vicinal dithioethers and alkenyl thioethers from the reaction of alkynes and thiols in water. *Eur. J. Org. Chem.* 168–173 (2010).

7. Xu, F., Shi, W. & Wang, J. 1,2-Thio group migration in Rh(II) carbene reactions. *J. Org. Chem*. **70**, 4191–4194 (2005).

8. Meindertsma, A. F., Pollard, M. M., Feringa, B. L., de Vries, J. G. & Minnaard, A. J. Asymmetric hydrogenation of alkyl(vinyl)thioethers: a promising approach to α-chiral thioethers. *Tetrahedron: Asymmetry* **18**, 2849–2858 (2007).

9. Lewandowska, E. Nucleophilic α-addition to β-nitroacrylates: application to the synthesis of α-thioacrylates. *Tetrahedron* **62**, 4879–4883 (2006).

10. Zhao, F. et al. Chemoenzymatic cascades for the enantioselective synthesis of β-hydroxysulfides bearing a stereocentre at the C−O or C−S bond by ketoreductases. *Angew. Chem. Int. Ed.* **61**, e202202363 (2022).

11. Effenberger, F. & Gaupp, S. Stereoselective substitution of (*R*)-2-(sulfonyloxy)nitriles with sulfur nucleophiles. *Tetrahedron: Asymmetry* **10**, 1765–1775 (1999).

12. Yamashita, H. & Mukaiyama, T. Asymmetric michael addition of thiophenol to maleic acid esters. *Chem. Lett*. **14**, 363–366 (1985).

13. Schrödinger Release 2020-3: Maestro, Schrödinger, LLC, New York, NY, 2020.

14. Friesner, R. A. et al. Glide: a new approach for rapid, accurate docking and scoring. 1. method and assessment of docking accuracy. *J. Med. Chem.* **47**, 1739–1749 (2004).

15. Friesner, R. A. et al. Extra precision glide: docking and scoring incorporating a model of hydrophobic enclosure for protein−ligand complexes. *J. Med. Chem.* **49**, 6177–6196 (2006).

16. Bochevarov, A. D. et al. Jaguar: a high-performance quantum chemistry software program with strengths in life and materials sciences. *Int. J. Quantum Chem.* **113**, 2110–2142 (2013).

17. Contreras, R. R., Fuentealba, P., Galván, M. & Pérez, P. A direct evaluation of regional Fukui functions in molecules. *Chem. Phys. Lett.* **304**, 405–413 (1999).

18. Chamorro, E. & Pérez, P. Condensed-to-atoms electronic Fukui functions within the framework of spin-polarized density-functional theory. *J. Chem. Phys.* **123**, 114107 (2005).
